# *Chlamydia trachomatis* transmission between the oropharynx, urethra and anorectum in men who have sex with men: a mathematical model

**DOI:** 10.1186/s12916-020-01796-3

**Published:** 2020-11-17

**Authors:** Xianglong Xu, Eric P. F. Chow, Jason J. Ong, Christian J. P. A. Hoebe, Zhuoru Zou, Jane S. Hocking, Christopher K. Fairley, Lei Zhang

**Affiliations:** 1 China Australia Joint Research Center for Infectious Diseases, School of Public Health, Xi’an Jiaotong University Health Science Centre, Xi’an, 710061 Shaanxi People’s Republic of China; 2 Melbourne Sexual Health Centre, Alfred Health, Melbourne, Australia; 3 Central Clinical School, Faculty of Medicine, Nursing and Health Sciences, Monash University, Melbourne, Australia; 4 Centre for Epidemiology and Biostatistics, Melbourne School of Population and Global Health, The University of Melbourne, Melbourne, Australia; 5 Department of Sexual Health, Infectious Diseases and Environmental Health, South Limburg Public Health Service, Heerlen, The Netherlands; 6 Department of Medical Microbiology, Care and Public Health Research Institute (CAPHRI), Maastricht University Medical Centre (MUMC+), Maastricht, The Netherlands; 7 Department of Epidemiology and Biostatistics, College of Public Health, Zhengzhou University, Zhengzhou, Henan People’s Republic of China

**Keywords:** Transmission, Behavioural interventions, Sexual practices, Anatomical site, *Chlamydia trachomatis*, Men who have sex with men

## Abstract

**Background:**

It has been presumed that *Chlamydia trachomatis* is transmitted between men only through anal or oral sex, but no mathematical models have tested this presumption.

**Methods:**

To test this presumption, we created 20 compartmental mathematical models of different sexual practices that included both oral and anal sex and calibrated these models to the observed rates of *Chlamydia trachomatis* infection at three anatomical sites from 4888 men who have sex with men (MSM) in Melbourne Sexual Health Centre during 2018–2019.

**Results:**

A model that included only oral and anal sex could replicate the observed rates of single-site infection at the oropharynx, urethra and rectum alone, but could not replicate infection at more than one of these sites (multisite). However, if we included transmission from sexual practices that followed one another in the same sexual episode (e.g. saliva contamination of the penis from oral sex transmitting chlamydia to the rectum by anal sex), we significantly improved the calibration of multisite infection rates substantially.

**Conclusions:**

Our modelling study suggests that transmission routes other than just oral and anal sex are necessary to explain the high rate of *Chlamydia trachomatis* infection at more than one site.

## Background

*Chlamydia trachomatis* (chlamydia) is a common sexually transmitted infection in men who have sex with men (MSM), and its burden is likely to rise over time as condom use falls in the pre-exposure prophylaxis (PrEP) era [1–4]. If the rates of condom use are to fall, new prevention measures will be needed, and these measures can only be developed if we clearly understand the transmission dynamics of chlamydia among MSM [5, 6].

Mathematical models are important for investigating the transmission of infections in populations, particularly when the transmission is complex or may be difficult to study epidemiologically [7, 8]. The transmission of chlamydia may be more complex than has been previously appreciated given transmission occurs between three anatomical sites (oropharynx, urethra and anorectum), and there are a large number of potential sexual practices that may transmit it. Indeed recent epidemiological studies have assessed some potential routes such as kissing, rimming (oral-anal sex) and the use of saliva as a lubricant for anal sex among MSM [9–11]. However, studying the potential contribution of these sexual practices to transmission using epidemiological studies is difficult because of the great number of questions that would be required for potentially many sexual partners. Such studies would also be difficult to analyse because men usually have multiple sexual practices in one sexual episode, making it hard to separate the individual contribution of each sexual practice. Several mathematical models have explored chlamydia transmission in heterosexuals [12–15], and also in MSM [16–20], but none of these studies used anatomical site-specific models. However, chlamydia can infect multiple anatomical sites, including the oropharynx, urethra and anorectum [10, 21–24]. Besides, the majority of MSM had multiple sexual practices in one sexual episode [25], which were not included in previous chlamydia models.

Given that little is known about chlamydia transmission, we progressively added other sexual practices to these two sexual practices to develop a series of mathematical models to determine what sexual practices were necessary to replicate the observed prevalence at each anatomical site (oropharynx, urethra and anorectum) in MSM.

## Methods

After recovery from *Chlamydia trachomatis* infection, an individual can be immediately susceptible again [26]. We constructed a susceptible-infectious-susceptible compartmental model to test which transmission routes were necessary for chlamydia to replicate the observed infection rates at specific anatomical sites in MSM. Our *Chlamydia trachomatis* models are based on two previous anatomical site-specific models [27, 28] (Additional file 1: Fig. S1).

### Model assumptions

**Fig. 1:**
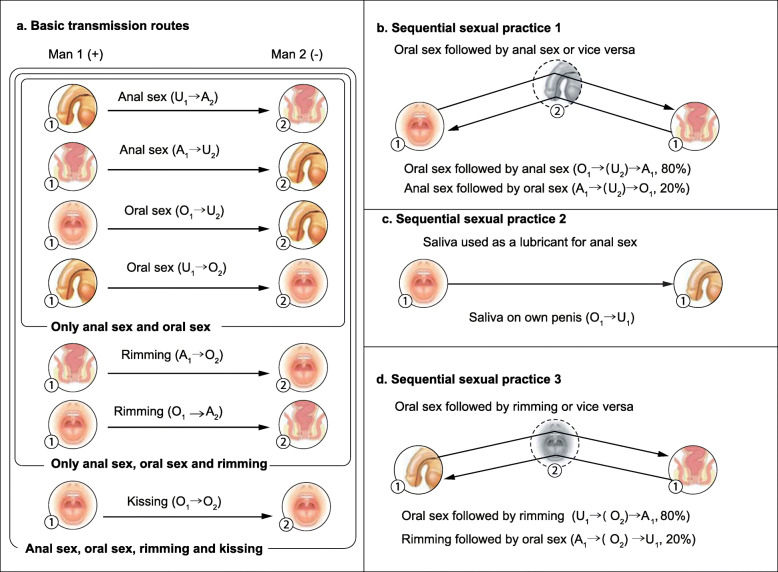
Baseline transmission routes (**a**) to which transmission routes 1–3 (**b**–**d**) have been added; arrow signifies the direction of transmission. Note: Man 1 ①; Man 2 ②

Our chlamydia models included the following assumptions related to multisite infections: (1) multisite infection could develop in a man who is already infected at one site when he has sex with another infected partner; and (2) within the same individual, and the *Chlamydia trachomatis* infection may be transmitted from one infected site to a different non-infected site through sexual practices during the same sexual episode (‘sequential sexual practices’) with another sex partner (outlined in Figs. 1 and 3) during a sequential sexual practice, we assumed that the medium anatomical site would act as a conduit for the transmission of the bacteria but not habour the bacteria at the site. As there currently is no evidence to inform the likelihood of infection at the medium anatomical site, we assumed that the medium anatomical site was not infected during a sequential sexual practice. This avoids adding substantial complexity to the model.

### Model development

Given that little is known about chlamydia transmission other than from anal and oral sex, we systematically established 20 compartmental models to test the effect of adding in different sexual practices as outlined in Fig. 1. The transmission routes we tested included: (a) anal sex and oral sex only, (b) the addition of riming (oral-anal sex), (c) the addition of riming and kissing and (d) including the possibility that an individual with an infection at one site can be infected at another site (from the original site) during a sexual episode with another person whose sexual acts ‘connect’ these two anatomical sites in the same person. These ‘connecting’ sexual practices we have termed as ‘sequential sexual practices’. The first sequential sexual practice was oral sex followed by anal sex (or the reverse) that allowed transmission from either the oral or anal site in the same person via chlamydia contamination on the partners’ penis. For example, if an individual has oropharyngeal chlamydia and has receptive oral sex that was followed by anal sex, then remnant chlamydia containing saliva on the sexual partner’s penis may transmit the infection from the mouth to the anus of the same individual via the partner’s penis. The second sequential sexual practice was when saliva was used as a lubricant for anal sex and therefore may pass the infection from the oropharynx to an individual’s own penis. The third sequential sexual practice was when either urethral or anal infection was passed to the urethra or anus (in the same person) via the partner’s mouth when oral sex was followed by oral-anal sex (rimming) or anal-oral sex was followed by oral-penile sex. These sexual practices may be important for increasing the chance of an individual having the infection at more than one site (multisite infection), because they connect sites in the same man and multiple sexual practices in a single sexual encounter (e.g. oral sex followed by anal sex) is very common in MSM [25]. The models (model 1–20) were represented as a group of ordinary differential equations in Additional file 1. We have not included other possible transmission routes into account. For example, we have assumed that transmission could not occur to the penis of one man from his partner’s saliva during anal sex that follows oral sex (Fig. 1b).

### Model parameterization and calibration

Our model parameters were from previously published biological and behavioural data of chlamydia (Additional file 2: Table S3) [2, 9, 20, 25, 27, 29–38] . We calibrated the models to diagnosis data of chlamydia infections at each anatomical site (i.e. oropharynx, urethra and anorectum) and multisite infection (oropharynx and urethra together, oropharynx and anorectum together, urethra and anorectum together, oropharynx and urethra and anorectum together) (Additional file 2: Table S4, 5).

We used six sources of data for model calibration. The first was data from the Melbourne Sexual Health Centre (MSHC) and included five similar studies on reported multisite infections of chlamydia. Urethral swabs, oropharyngeal swabs and anorectal swabs were tested for chlamydia by using nucleic acid amplification tests (NAAT) in MSHC [39]. To avoid the bias of multiple visits by the same returning patients, the model used data of chlamydia prevalence among 4888 MSM who visited MSHC for the first time and included 2565 MSM in 2018 and 2323 MSM in 2019 (Additional file 2: Table S4). The proportion of MSM who had more than one anatomical site infected with chlamydia was 20.0% (124/620). We also identified five similar studies reported multisite infections of chlamydia using NAAT as the validation data sets, including one study in Australia [40], two studies in the USA [41, 42], one study in the Netherlands [21] and one study in Thailand [22]. (Additional file 2: Table S5). We did not aim to collect all available multisite infections studies for chlamydia to cross-validate our findings.

The collected parameters of chlamydia models were sampled within the confidence interval by the Latin hypercube sampling method and repeated 300 times. By using the sum of squares of the errors to estimate the goodness of fit between simulation output and the epidemiological multisite infections data, we selected ‘optimal runs’ and we sorted the simulation outputs in descending order and selected the top 10% simulations to generate the chlamydia models outputs with 95% confidence intervals. We measured the fitting error by calculating the sum of the squared errors. We compared chlamydia models using the minimal of sum squared errors between the empirical multisite infections data and the corresponding calibration results. We performed an independent *t* test to check for differences in terms of the means of the sum of squared errors between two models. Statistical significance was considered at *p* < 0.05. The detailed calibration for models was provided in Additional file 1 [27, 36–38, 43, 44]. Output parameters for *Chlamydia trachomatis* models were provided in the additional file (Additional file 3: Tables S 8.1-8.219). We used MATLAB R 2019a to solve differential equations and conduct statistical analyses.

### Estimating the composition of chlamydia incidence

We used our calibrated models to estimate the composition of chlamydia incidence caused by oropharyngeal, anorectal and urethral infection. Based on our previously reported method [27], we estimated the new infections at any given time and calculated the ratio between the number of new infections and the number of susceptible. We also used our models to estimate the proportion of incidence caused by sexual practices.

### Uncertainty and sensitivity analysis

We conducted sensitivity analyses for the chlamydia models using the whole parameter set with varying some important parameters, including the duration of infection, frequency of sexual practices and the proportion of men with sequential sexual practices (details in Additional file 1).

## Results

### Replication of site-specific chlamydia prevalence without sequential sexual practices

The three models (models 1 to 3) that included no sequential sexual practices could only replicate single site infection at the oropharyngeal, urethra and anorectum, but could not replicate the high proportion of men with chlamydia at more than one site (multisite infection) (Additional file 4: Fig. S2a).

### Replication of site-specific chlamydia prevalence with sequential sexual practices

We added the three sequential sexual practices that connected all three anatomical sites in the same person to model 1 (anal sex and oral sex only) to build models 4–6. Like models 1–3, we were able to replicate single site infection at the oropharynx, urethra and anorectum in models 4–6. However, we were also able to improve the calibration of multisite infection substantially and significantly both at the oropharynx and anorectum when we added oral sex followed by anal sex (or vice versa) (model 4 vs. model 1 (*p* < 0.01) and model 6 vs. model 1 (*p* < 0.01)). (Additional file 4: Fig. S8a; Additional file 2: Table S6).

**Fig. 2:**
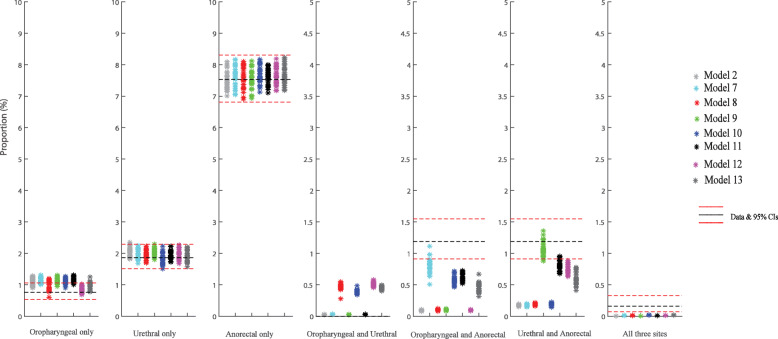
Estimates of the eight models for the percentage of specific anatomical sites positive for *Chlamydia trachomatis* for the 8 models (model 2, 7–13) and the 95% confidence intervals for the observed site-specific positivity among 4888 MSM attending Melbourne Sexual Health Centre in 2018 and 2019. The prevalence of oropharyngeal and urethral infection is zero and that therefore the dashed lines are missing. Model 2 (dark grey asterisk): anal and oral sex and rimming. Model 7 (cyan asterisk): anal sex and oral sex, rimming and sequential oral/anal sex. Model 8 (red asterisk): anal sex and oral sex and rimming and using saliva as a lubricant for anal sex. Model 9 (green asterisk): anal sex and oral sex and rimming and sequential oral sex/riming. Model 10 (blue asterisk): anal sex and oral sex and rimming, sequential oral/anal sex and using saliva as a lubricant for anal sex. Model 11 (black asterisk): anal sex and oral sex and rimming and sequential oral/anal sex and sequential oral sex/riming. Model 12 (magenta asterisk): anal sex and oral sex and rimming and using saliva as a lubricant for anal sex and sequential oral sex/riming. Model 13 (dim grey asterisk): anal sex and oral sex and rimming and sequential oral/anal sex and using saliva as a lubricant for anal sex and sequential oral sex/riming

We added the three sequential sexual practice that connected all three anatomical sites in the same person to model 2 (anal sex, oral sex and rimming) to build models 7–13. Like in models 4–6, we were able to replicate single-site infection at the oropharyngeal, urethra and anorectum. Besides, we were also able to improve the calibration of multisite infection substantially and significantly both at the oropharynx and anorectum when we added oral sex followed by anal sex (or vice versa) to model 2 (models 7, 10, 11 and 13). Similarly, we could substantially improve the calibration of multisite infection at both the urethra and anorectum (Fig. 2a) when we added oral sex followed by rimming (or reverse) to model 2 (models 9, 11–13). According to the sum of squared errors, our best calibration model among all above 13 models (model 1–13) was model 11 (introduction of oral sex followed by anal sex (or vice versa) and oral sex followed by rimming (or vice versa)) (Additional file 2: Table S6).

We added the three sequential sexual practice that connects all three anatomical sites in the same person to model 3 (anal sex, oral sex, rimming and kissing only) to build models 14–20. Like in models 1–13, we were able to replicate single site infection at the oropharyngeal, urethra and anorectum in models 14–20. Furthermore, we were also able to improve the calibration of multisite infection substantially and significantly at both the oropharynx and anorectum when we added oral sex followed by anal sex (or vice versa). Similarly, we were able to substantially improve the calibration of multisite infection at both the urethra and anorectum when we added oral sex followed by rimming (or vice versa) (Additional file 4: Fig. S27a).

We compared the calibration of the models using the sum of squared errors and used the baseline model as model 1 (for models 4–6), model 2 (for model 7–13) and model 3 (for models 14–20). The calibration performance results of all 20 chlamydia models are reported in the additional file (Additional file 2: Table S6). We analysed the comparison of the means of the sum of squared error between model 11 and model 18, and the difference had no statistical significance (*p* = 0.0869).

### Estimate the relative proportions of incident chlamydia at different sites and due to different sexual practices

**Fig. 3:**
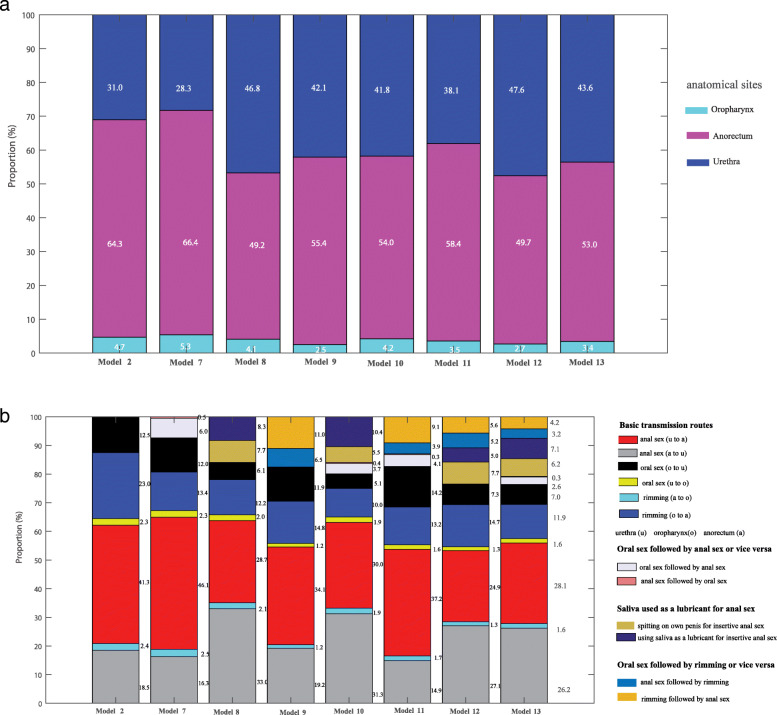
**a** Estimated proportion of incident *Chlamydia trachomatis* cases that occur at the oropharynx, anorectum or urethra in MSM from the eight models (model 2, 7–13) among 4888 MSM attending Melbourne Sexual Health Centre in 2018 and 2019. Model 2: anal and oral sex and rimming. Model 7: anal sex and oral sex, rimming and sequential oral/anal sex. Model 8: anal sex and oral sex and rimming and using saliva as a lubricant for anal sex. Model 9: anal sex and oral sex and rimming and sequential oral sex/riming. Model 10: anal sex and oral sex and rimming, sequential oral/anal sex and using saliva as a lubricant for anal sex. Model 11: anal sex and oral sex and rimming and sequential oral/anal sex and sequential oral sex/riming. Model 12: anal sex and oral sex and rimming and using saliva as a lubricant for anal sex and sequential oral sex/riming. Model 13: anal sex and oral sex and rimming and sequential oral/anal sex and using saliva as a lubricant for anal sex and sequential oral sex/riming and rimming and using saliva as a lubricant for anal sex. Model 9 (green asterisk): anal sex and oral sex and rimming and sequential oral sex/riming; Model 10 (blue asterisk): Anal sex and oral sex and rimming, sequential oral/anal sex and using saliva as a lubricant for anal sex. Model 11 (black asterisk): anal sex and oral sex and rimming and sequential oral/anal sex and sequential oral sex/riming. Model 12 (magenta asterisk): anal sex and oral sex and rimming and using saliva as a lubricant for anal sex and sequential oral sex/riming. Model 13 (dim grey asterisk): anal sex and oral sex and rimming and sequential oral/anal sex and using saliva as a lubricant for anal sex and sequential oral sex/riming. **b** Estimated proportion of incident *Chlamydia trachomat*is cases caused by sexual practices in MSM from the eight models (model 2, 7–13) among 4888 MSM attending Melbourne Sexual Health Centre in 2018 and 2019. Model 2: anal and oral sex and rimming. Model 7: anal sex and oral sex, rimming and sequential oral/anal sex. Model 8: anal sex and oral sex and rimming and using saliva as a lubricant for anal sex. Model 9: anal sex and oral sex and rimming and sequential oral sex/riming. Model 10: anal sex and oral sex and rimming, sequential oral/anal sex and using saliva as a lubricant for anal sex; Model 11: Anal sex and oral sex and rimming and sequential oral/anal sex and sequential oral sex/riming. Model 12: anal sex and oral sex and rimming and using saliva as a lubricant for anal sex and sequential oral sex/riming. Model 13: anal sex and oral sex and rimming and sequential oral/anal sex and using saliva as a lubricant for anal sex and sequential oral sex/riming

We used model 2 and models 7–13 to calculate the proportion of incident cases because these models provided the best calibration. The incidence of chlamydia infection varied by anatomic site (anorectal, range 50.9–63.7%; urethral, range 31.4–43.6%; and oropharyngeal, range 5.2–7.5%), across all eight models (Fig. 3a). The proportion of incident chlamydia caused by anal sex only was 44.7–55.9% (anorectum-to-urethra, range 14.0–30.8%; urethra-to-anorectum, range 24.5–36.1%), by riming only was 15.6–36.1% (anorectum-to-oropharynx, range 2.6–3.6%; oropharynx to anorectum, 13.1–32.5%), by oral sex only was 8.7–19.2% (urethra-to-oropharynx, range 2.6–3.7%; oropharynx-to-urethra, range 5.6–15.6%) and by all sequential activities was 7.3–23.6%, across all eight models (Fig. 3b).

### Sensitivity analysis

We performed sensitivity analyses of the models that provided the best calibration of multisite infection (models 2, 7–13). The results showed that varying key model indicators, including duration of infection, frequency of sexual practices and the proportion of men with sequential sexual practices, would not alter our conclusions relating to model calibration and incidence estimations. Including oral sex followed by anal sex (or reverse), saliva use as a lubricant for anal sex and oral sex followed by rimming (or reverse) were essential for calibrating multisite chlamydia infection. Anorectal infection and anal sex contributed the most to chlamydia incidence. We repeated the same analysis in all five validation datasets, and the results were essentially the same. Similarly, the five validation datasets showed that anorectal infections and anal sex contributed the most to chlamydia incidence (detailed results of sensitivity analysis in Additional file 4).

## Discussion

This is the first modelling study to explore the role that different sexual practices play in chlamydia transmission at each anatomical site (oropharynx, urethra and anorectum) in MSM. We were unable to replicate the high proportion of chlamydia infection at more than one anatomical site without including some sequential sexual practices that transmit chlamydia between anatomical sites in the same person. Including oral sex followed by anal sex (or vice versa) improved the calibration of multisite infection at both the oropharynx and anorectum. Including oral sex followed by rimming (or vice versa) improved the calibration of multisite infection, both for the urethra and anorectum. Our optimal calibration results were obtained with oral sex, anal sex, riming and sequential oral and anal sex together with sequential oral sex and rimming. Our findings suggest that chlamydia transmission may be more complicated than had previously been appreciated, and therefore, the public health messages required to control it, in an environment with falling rates of condoms use may need to be more complex than just using condoms more. These hypothetical models of transmission and other potential ones that we did not explore will need to be confirmed in epidemiological studies, but our data will provide some guidance for the direction of these studies.

Our study has several limitations. First, we had to make a number of assumptions about the parameters when published data were not available or uncertainty. To address this issue, we performed sensitivity analyses for some of these variables. For example, we used different estimates of the duration of asymptomatic urethral and anal infection, frequency of sexual practices. Second, we did not include all possible sequential practices because little is known about them, and there are many possible permutations and combinations. Third, we also did not include transmission to the anatomical site that was acting as the conduit between two other sites during the sequential sexual practice. For example, we assumed that when oral sex is followed by anal sex that the penis could not be infected from the remnant saliva during anal sex. Similarly, we assumed that when anal sex is followed by oral sex that the penis could not be infected from remnant faecal material during oral sex. We acknowledge that this may happen but not only would it add substantial complexity to an already complicated model, but transmission between these sites could also occur through other acts (oral sex or anal sex). These transmission routes also do not address the key epidemiological issue that infection at more than one site in the same man is currently not well explained. We feel that our findings should be interpreted only as indicating that transmission is likely to be more complicated than had previously been appreciated, and transmission is unlikely to be solely from just anal and oral. Finally, our models did not include group sex because little is known about sequential sexual practices with more than one sex partner.

One important issue to consider is whether chlamydia can remain viable and infectious in genital secretions (e.g. saliva) and therefore remain infectious during sequential sexual acts. Chlamydial DNA is detected in genital secretions, including commonly in the saliva (69%) of men who have oropharyngeal chlamydia [45]. Although there have not been studies to assess the viability of chlamydia in saliva, it is commonly reported to be viable in rectal samples (58%) using mRNA and studies using culture suggest it may be viable in the environment [46, 47]. Researchers from The Netherlands particularly who have pioneered the viability assays suggest that inoculation from the vagina to the anus may be the cause for the high and unexplained rate of anal chlamydia in women [46]. Further studies will be required to clarify this important issue.

Our models suggested that oropharyngeal chlamydia incidence was relatively low in MSM and helped explain the epidemiological data showing that oropharyngeal chlamydia was not common [48, 49]. Our findings are consistent with chlamydia’s higher affinity for columnar epithelium rather than the squamous epithelium that mostly covers the oropharynx [50]. It has been speculated that this is why there is a lower prevalence of chlamydia in the throat compared to *Neisseria gonorrhoeae* [40]. Our models indicated that anorectal infection significantly contributed to the overall incidence of chlamydia. Our model suggests that penile-anal sex is the main contributor to new chlamydia infections in MSM. Our findings suggest that consistent condom use for penile-anal sex could prevent more than half of incident cases and so promoting their use is an effective prevention strategy. Our estimated incidence data support the observed epidemiological data that anorectal chlamydia infection was the most common site-specific infection [51, 52]. Our models suggested that the estimated proportion of incident chlamydia caused by all sequential activities while low (7.3–23.6%) was necessary to replicate the observed epidemiology.

There are relatively few epidemiological studies investigating the transmission of chlamydia by sexual practices other than anal and oral sex in MSM. While it remains unclear, for example, whether sexual practices that involve saliva or saliva contamination could contribute to chlamydia transmission [10]. The Health In Men Study reported that insertive oral sex with ejaculation was associated with urethral chlamydia infection, and incident anal chlamydia was associated with receptive rimming [31]. A case-control study of chlamydial urethritis suggested that about 13% of cases were from oral sex or saliva exposure from oral sex [32]. However, a cross-sectional study of MSM attending a sexual health service found that using saliva as a lubricant and rimming was not associated with the presence of chlamydia [10]. There have been only one study assessing kissing, and this study did not find that kissing was associated with oropharyngeal chlamydia [9]. These findings from epidemiological studies are generally consistent with our models.

Our study suggests that the transmission of chlamydia is more complex than had previously been appreciated. Our models found that inclusion of sequential sexual practices (oral sex followed by anal sex and oral sex followed by rimming) could improve the calibration of multisite infection substantially, particularly multisite infection of both the oropharynx and anorectum and multisite infection of both the urethra and anorectum. We thought it was important to assess more complex sexual practices because a previous study reported that most MSM had more than one sexual practice during the same sexual episode [25]. Our findings suggest that more complicated transmission routes may be required to explain multisite chlamydia prevalence, and, thus, chlamydia caused by sequential sexual practices deserve further attention. The findings of our study also could provide some direction for epidemiological research of the new transmission routes of chlamydia that our models suggest oral sex followed by anal sex (or reverse) and oral sex followed by rimming (or reverse) are necessary to replicate the epidemiological data.

## Conclusions

Our study is the first modelling study to explore the role that different sexual practices play in chlamydia transmission at each anatomical site (oropharynx, urethra and anorectum) in MSM. Our modelling study suggests that transmission routes other than just oral and anal sex are necessary to explain the high rate of *Chlamydia trachomatis* infection at more than one site. Our modelling study may be useful to understand the transmission of *Chlamydia trachomatis* between the oropharynx, urethra and anorectum in men who have sex with men, thus making an essential step towards the control of chlamydia transmission at multiple anatomical sites in men.

## Supplementary information

**Additional file 1.** Details of model equations, model calibration and sensitive analysis.

**Additional file 2: Table S1.** Definition of *Chlamydia trachomatis* models based on sexual practices. **Table S2.** Sexual practices included in the 20 *Chlamydia trachomatis* models. **Table S3.** Biological and behavioural data of *Chlamydia trachomatis* for model parameterization in MSM. **Table S4.** The anatomical site-specific infection prevalence of *Chlamydia trachomatis* in MSM attending MSHC in 2018 and 2019. **Table S5.** All anatomical site-specific infection prevalence datasets of *Chlamydia trachomatis* for model calibration and validation. **Table S6.** Output parameters and transmission probability of the best-calibrated model and baseline model based on data from 4888 MSM attending Melbourne Sexual Health Centre. **Table S7.** Sum of squared error of 20 *Chlamydia trachomatis* models on data of 4888 MSM attending Melbourne Sexual Health Centre in 2018 and 2019.

**Additional file 3. **Output parameters for *Chlamydia trachomatis* models. **Tables S 8.1-8.219**.

**Additional file 4. **Additional results: model calibration, estimating the composition of incidence, and sensitivity analysis. **Figs. S2a-32c.**

## Data Availability

All data analysed during this study are included in this article and its additional file.

## Abbreviations

chlamydia: *Chlamydia trachomatis*
MSHC: Melbourne Sexual Health Centre
MSM: Men who have sex with men
