# Supplementary material for: Chlamydia trachomatis transmission between the oropharynx, urethra and anorectum in men who have sex with men: a mathematical model
Source: BMC Med. 2020 Nov 17;18:326. doi: 10.1186/s12916-020-01796-3 (PMC7670797; doi:10.1186/s12916-020-01796-3)
Supplement: Supplementary file 1 — Additional file 1. Details of model equations, model calibration and sensitive analysis. [file 12916_2020_1796_MOESM1_ESM.docx]

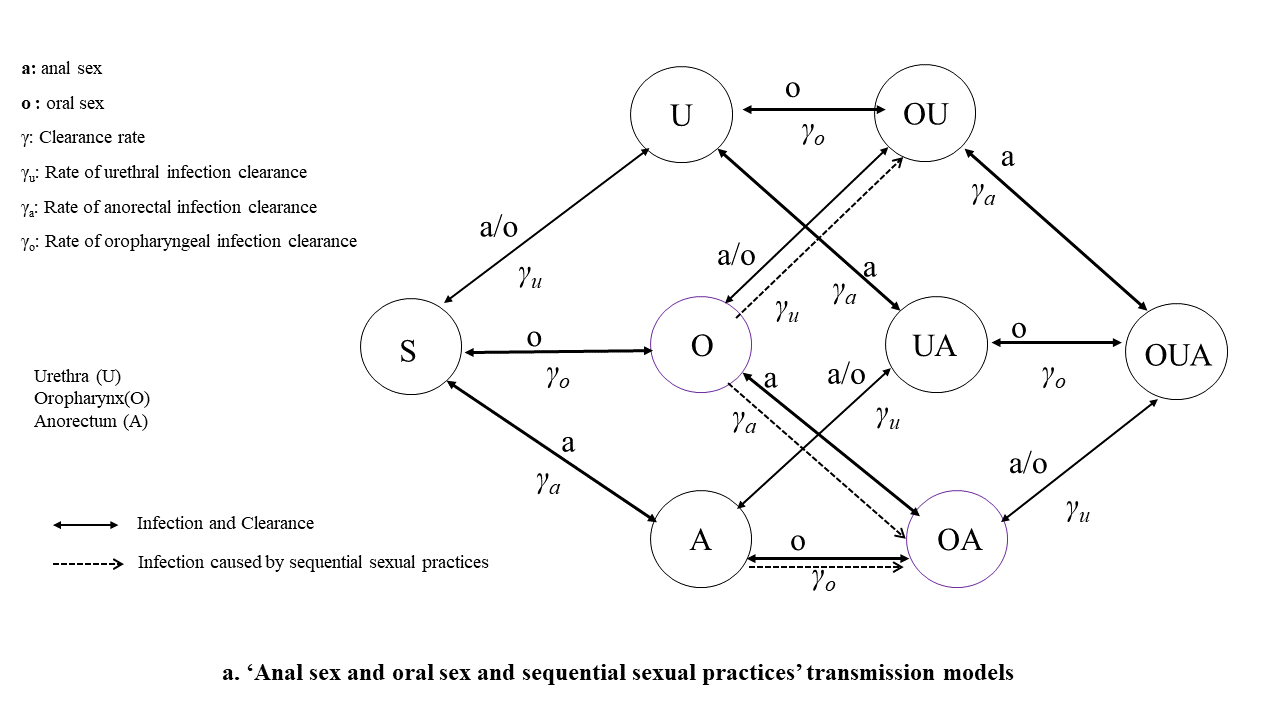


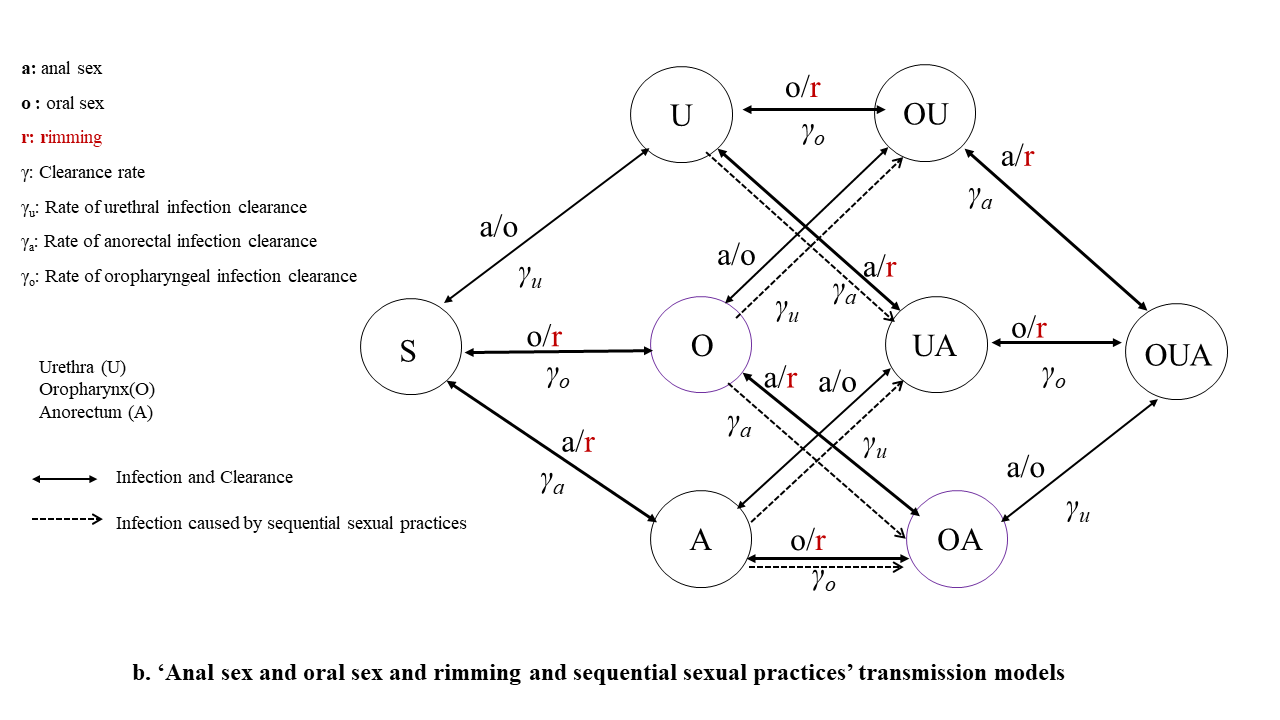


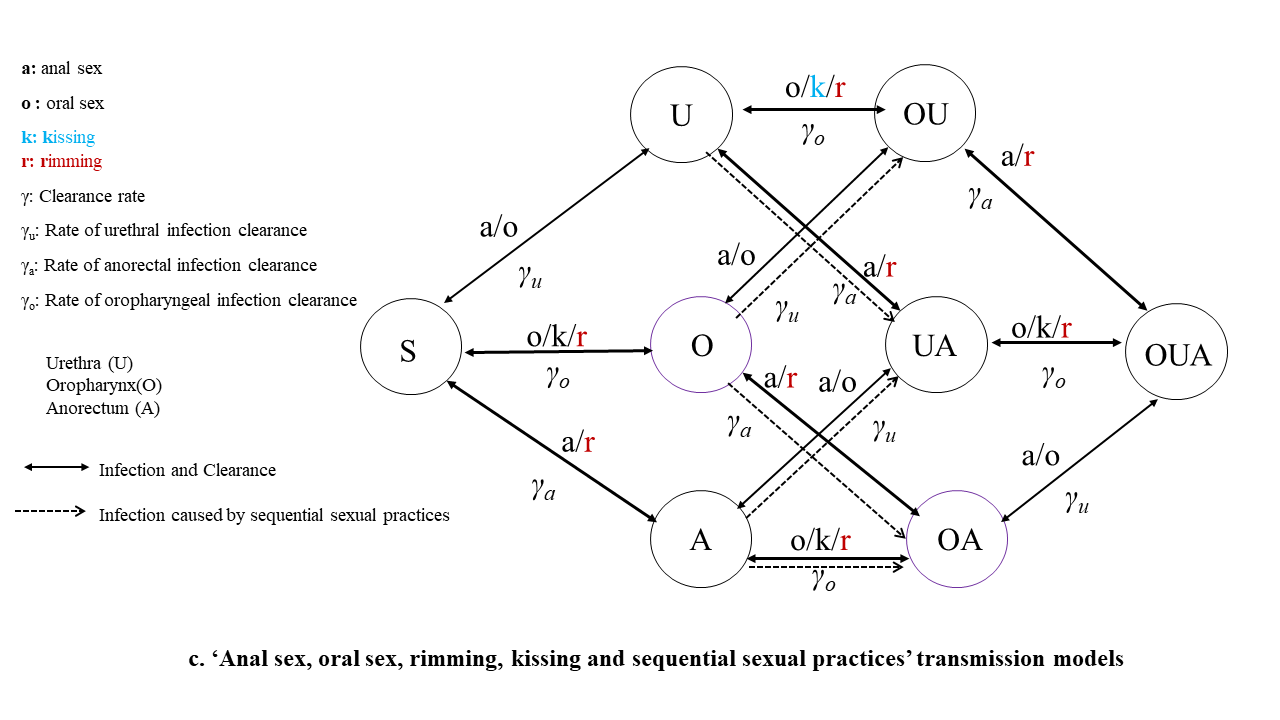


**Figure S1.** Anatomical site-specific transmission model of *Chlamydia trachomatis* in men who have sex with men; a. ‘Anal sex and oral sex and sequential sexual practices’ transmission models; b. ‘Anal sex and oral sex and rimming and sequential sexual practices’ transmission models; c. ‘Anal sex, oral sex, rimming, kissing and sequential sexual practices’ transmission models

**Ordinary differential equations used in *Chlamydia trachomatis* transmission models**

Our *Chlamydia trachomatis* transmission models expressed as a system of ordinary differential equations (ODE). Our models are based on our previous study [1]. Parameter symbols were used in ordinary differential equations including S: Susceptible MSM; I : Infected MSM; U: MSM had only urethra infection; O: MSM had only oropharynx infection; A: MSM had only anorectum infection; OU: MSM had only oropharynx and urethra infections; UA: MSM had only urethra and anorectum infections; OA: MSM had only oropharynx and anorectum infections; OUA: MSM had only oropharynx, urethra and anorectum infections; Io: Number of infected at oropharynx; Iu: Number of infected at the urethra; Ia: Number of infected at the anorectum; Iou: Number of infected at the oropharynx and urethra; Iua: Number of infected at the anorectum, and urethra; Ioa: Number of infected at oropharynx and urethra; Ioua: Number of infected at oropharynx, anorectum, and urethra; P: Prevalence; Po: The prevalence of infected only at the oropharynx; Pa: The prevalence of infected only at the anorectum; Pu :The prevalence of infected only at the urethra; Pou: The prevalence of infected only at oropharynx and urethra; Pua: The prevalence of infected only at the anorectum, and urethra; Poa: The prevalence of infected only at the oropharynx and urethra; Poua: The prevalence of infected at oropharynx, anorectum, and urethra; λ: rate of conversion from susceptibles to infected individuals, it is a function of per-act transmission probability, frequency of sex acts, condom use and condom efficacy, the product of λ and site-specific prevalence defines the ‘force of infection’ at the specific site; $\lambda_{ij}$: the rate of conversion from the site i to j (λoo: kissing (oropharynx to oropharynx); λao: rimming (anorectum to oropharynx); λoa: rimming (oropharynx to anorectum); λau: anal sex (anorectum to urethra); λua: anal sex (urethra to anorectum); λuo: oral sex (urethra to oropharynx); λou: oral sex (oropharynx to urethra)); The rate of conversion at various sites due to sequential sexual practices (λooa: conversion at anorectum due to sequential oral sex followed by anal sex; λaoa: conversion at oropharynx due to sequential anal sex followed by oral sex; λoou: conversion at urethra due to spiting saliva on own penile; λua2: conversion at anus due to using saliva as a lubricant for penile-anal sex; λuua: conversion at anorectum due to sequential oral sex followed by riming; λaua: conversion at urethra due to sequential riming followed by oral sex) ; $\beta_{ij}$: The per-act transmission probability from the site i to j; $\varepsilon_{c}$: The efficacy of condom in preventing transmission of infection; C: The percentage of condom use in anal sex; $f_{ij}$:The frequency of sexual practices from the site i to j (including oral sex, anal sex, kissing, and rimming); The frequency of sexual acts (f) was estimated based on the number of partners and the number of sexual acts per partner. For example, i(oropharynx), j(urethra), then fij is the frequency of oral sex (including oropharynx-to- urethra, and urethra-to- oropharynx). For the whole MSM population, a man with infection at the site “i(oropharynx)” infected another man’s site “j(urethra)”, or a man with infection at site “j(urethra)” infected another man’s site “i(oropharynx)”. γ: The rate of infection clearance; γ_u_: The rate of oropharyngeal infection clearance; γ_a_: The rate of anorectal infection clearance; γ_o_: The rate of urethral infection clearance; incid.au: New infection caused by anal sex (anorectum to urethra); incid.ao: New infection caused by rimming (anorectum to oropharynx); incid.ua: New infection caused by anal sex (urethra to anorectum); incid.uo: New infection caused by oral sex (urethra to oropharynx); incid.oa: New infection caused by rimming (oropharynx to anorectum); incid.ou: New infection caused by oral sex (oropharynx to urethra); incid.oo: New infection caused by kissing (oropharynx to oropharynx); incid.ooa: New infection caused by sequential oral sex followed by anal sex; incid.aoa: New infection caused by sequential anal sex followed by oral sex; incid.oou: New infection caused by spit saliva on own penile; incid.ua2: New infection caused by saliva using as a lubricant for penile-anal sex; incid.uua: New infection caused by sequential oral sex followed by riming; incid.aua: New infection caused by sequential riming followed by oral sex; incid.o: New infection occurred at the oropharynx; incid.a: New infection occurred at the anorectum; incid.u: New infection occurred at the urethra.

The ODE is as follows:

$$\frac{dS}{dt}=\frac{-\lambda SI}{N}+\gamma\cdot I$$

$$\frac{dI}{dt}=\frac{\lambda SI}{N}-\gamma\cdot I$$

$\lambda_{ij}=({1-\left( 1-\beta_{ij}\cdot(1-\varepsilon_{c}\cdot C) \right)}^{\frac{f_{ij}}{2}})$

$$N=S+I_{o}+I_{u}+I_{a}+I_{ou}+I_{ua}+I_{oa}+I_{oua}$$

$$P_{o}=I_{o}/N$$

$$P_{u}=I_{u}/N$$

$$P_{a}=I_{a}/N$$

$$P_{ou}=I_{ou}/N$$

$$P_{ua}=I_{ua}/N$$

$$P_{oa}=I_{oa}/N$$

$$P_{oua}=I_{oua}/N$$

$$P_{o\_all}=P_{o}+P_{ou}+P_{oa}+P_{oua}$$

$$P_{a\_all}=P_{a}+P_{oa}+P_{ua}+P_{oua}$$

$$P_{u\_all}=P_{u}+P_{ou}+P_{ua}+P_{oua}$$

$$P_{ou\_all}=P_{ou}+P_{oua}$$

$$P_{ua\_all}=P_{ua}+P_{oua}$$

$$P_{oa\_all}=P_{oa}+P_{oua}$$

***‘Anal sex and oral sex and sequential sexual practices’ transmission models***

$$a1=\lambda_{uo}{\boldsymbol{\cdot}P}_{u\_all}$$

$$a2=\lambda_{ua}{\boldsymbol{\cdot}P}_{u\_all}$$

$$a3=\lambda_{ou}{\boldsymbol{\cdot}P}_{o\_all}+\lambda_{au}\boldsymbol{\cdot}P_{a\_all}$$

$$\frac{dP_{s}}{dt}=-a1\cdot P_{s}+\gamma_{o}\cdot P_{o}-a2\cdot P_{s}+\gamma_{a}\cdot P_{a}-a3\cdot P_{s}+\gamma_{u}\cdot P_{u}$$

$$\frac{dP_{o}}{dt}=a1\cdot P_{s}-\gamma_{o}\cdot P_{o}-a2\cdot P_{o}+\gamma_{a}\cdot P_{oa}-a3\cdot P_{o}+\gamma_{u}\cdot P_{ou}-\boldsymbol{\lambda}_{\boldsymbol{ooa}}\boldsymbol{\cdot}\boldsymbol{P}_{\boldsymbol{o}}\boldsymbol{-}\boldsymbol{\lambda}_{\boldsymbol{oou}}\boldsymbol{\cdot}\boldsymbol{P}_{\boldsymbol{o}}$$

$$\frac{dP_{u}}{dt}=a3\cdot P_{s}-\gamma_{u}\cdot P_{u}-a1\cdot P_{u}+\gamma_{o}\cdot P_{ou}-a2\cdot P_{u}+\gamma_{a}\cdot P_{ua}-\boldsymbol{\lambda}_{\boldsymbol{ua}\boldsymbol{2}}\boldsymbol{\cdot}\boldsymbol{P}_{\boldsymbol{o}}$$

$$\frac{dP_{a}}{dt}=a2\cdot P_{s}-\gamma_{a}\cdot P_{a}-a1\cdot P_{a}+\gamma_{o}\cdot P_{oa}-a3\cdot P_{a}+\gamma_{u}\cdot P_{ua}\boldsymbol{-}\boldsymbol{\lambda}_{\boldsymbol{aoa}}\boldsymbol{\cdot}\boldsymbol{P}_{\boldsymbol{a}}\boldsymbol{+}\boldsymbol{\lambda}_{\boldsymbol{ua}\boldsymbol{2}}\boldsymbol{\cdot}\boldsymbol{P}_{\boldsymbol{o}}$$

$$\frac{dP_{ou}}{dt}=a1\cdot P_{u}-\gamma_{o}\cdot P_{ou}+a3\cdot P_{o}-\gamma_{u}\cdot P_{ou}-a2\cdot P_{ou}+\gamma_{a}\cdot P_{oua}+\boldsymbol{\lambda}_{\boldsymbol{oou}}\boldsymbol{\cdot}\boldsymbol{P}_{\boldsymbol{o}}$$

$$\frac{dP_{oa}}{dt}= a2\cdot P_{o}-\gamma_{a}\cdot P_{oa}+a1\cdot P_{a}-\gamma_{o}\cdot P_{oa}-a3\cdot P_{oa}+\gamma_{u}\cdot P_{oua}\boldsymbol{+}\boldsymbol{\lambda}_{\boldsymbol{ooa}}\boldsymbol{\cdot}\boldsymbol{P}_{\boldsymbol{o}}\boldsymbol{+}\boldsymbol{\lambda}_{\boldsymbol{aoa}}\boldsymbol{\cdot}\boldsymbol{P}_{\boldsymbol{a}}$$

$$\frac{dP_{ua}}{dt}=a2\cdot P_{u}-\gamma_{a}\cdot P_{ua}+a3\cdot P_{a}-\gamma_{u}\cdot P_{ua}-a1\cdot P_{ua}+\gamma_{o}\cdot P_{oua}$$

$$\frac{dP_{oua}}{dt}=a2\cdot P_{ou}-\gamma_{a}\cdot P_{oua}+a1\cdot P_{ua}-\gamma_{o}\cdot P_{oua}+a3\cdot P_{oa}-\gamma_{u}\cdot P_{oua}$$

**Estimating attributed to sexual practices**

$$incid.au=\lambda_{au}\cdot P_{a\_all}\cdot(Ps+ Po+ Pa+ Poa)$$

$$incid.ua=\lambda_{ua \cdot}P_{u\_all}\cdot(Ps+ Po+ Pu+ Pou)$$

$$incid.uo=\lambda_{uo \cdot}P_{u\_all}\cdot(Ps+ Pu+ Pa+ Pua)$$

$$incid.ou=\lambda_{ou}{\cdot P}_{o\_all}\cdot(Ps+ Po+ Pa+ Poa)$$

$$incid.ooa=\lambda_{ooa \cdot}P_{o}$$

$$incid.aoa=\lambda_{aoa}\cdot P_{a}$$

$$incid.oou=\lambda_{oou\cdot}P_{o}$$

$$incid.ua2=\lambda_{ua2}{\cdot P}_{o}$$

**Incidence attributed to** **oropharyngeal, anorectal, and urethral infection**

$$incid.o= incid.uo+ incid.aoa$$

$$incid.a= incid.ua+ incid.ooa+ incid.ua2$$

$$incid.u= incid.oou +incid.au+ incid.ou$$

***‘Anal sex and oral sex and rimming and sequential sexual practices’ transmission models***

$$a1=\lambda_{ao}\boldsymbol{\cdot}P_{a\_all}+\lambda_{uo}{\boldsymbol{\cdot}P}_{u\_all}$$

$$a2=\lambda_{oa}\boldsymbol{\cdot}P_{o\_all}+\lambda_{ua}{\boldsymbol{\cdot}P}_{u\_all}$$

$$a3=\lambda_{ou}{\boldsymbol{\cdot}P}_{o\_all}+\lambda_{au}{\boldsymbol{\cdot}P}_{a\_all}$$

$$\frac{dP_{s}}{dt}=-a1\cdot P_{s}+\gamma_{o}\cdot P_{o}-a2\cdot P_{s}+\gamma_{a}\cdot P_{a}-a3\cdot P_{s}+\gamma_{u}\cdot P_{u}$$

$$\frac{dP_{o}}{dt}=a1\cdot P_{s}-\gamma_{o}\cdot P_{o}-a2\cdot P_{o}+\gamma_{a}\cdot P_{oa}-a3\cdot P_{o}+\gamma_{u}\cdot P_{ou}-\boldsymbol{\lambda}_{\boldsymbol{ooa}}\boldsymbol{\cdot}\boldsymbol{P}_{\boldsymbol{o}}\boldsymbol{-}\boldsymbol{\lambda}_{\boldsymbol{oou}}\boldsymbol{\cdot}\boldsymbol{P}_{\boldsymbol{o}}$$

$$\frac{dP_{u}}{dt}=a3\cdot P_{s}-\gamma_{u}\cdot P_{u}-a1\cdot P_{u}+\gamma_{o}\cdot P_{ou}-a2\cdot P_{u}+\gamma_{a}\cdot P_{ua}-\boldsymbol{\lambda}_{\boldsymbol{uua}}\boldsymbol{\cdot}\boldsymbol{P}_{\boldsymbol{u}}-\boldsymbol{\lambda}_{\boldsymbol{ua}\boldsymbol{2}}\boldsymbol{\cdot}\boldsymbol{P}_{\boldsymbol{o}}$$

$$\frac{dP_{a}}{dt}=a2\cdot P_{s}-\gamma_{a}\cdot P_{a}-a1\cdot P_{a}+\gamma_{o}\cdot P_{oa}-a3\cdot P_{a}+\gamma_{u}\cdot P_{ua}\boldsymbol{-}\boldsymbol{\lambda}_{\boldsymbol{aoa}}\boldsymbol{\cdot}\boldsymbol{P}_{\boldsymbol{a}}\boldsymbol{-}\boldsymbol{\lambda}_{\boldsymbol{aua}}\boldsymbol{\cdot}\boldsymbol{P}_{\boldsymbol{a}}\boldsymbol{+}\boldsymbol{\lambda}_{\boldsymbol{ua}\boldsymbol{2}}\boldsymbol{\cdot}\boldsymbol{P}_{\boldsymbol{o}}$$

$$\frac{dP_{ou}}{dt}=a1\cdot P_{u}-\gamma_{o}\cdot P_{ou}+a3\cdot P_{o}-\gamma_{u}\cdot P_{ou}-a2\cdot P_{ou}+\gamma_{a}\cdot P_{oua}+\boldsymbol{\lambda}_{\boldsymbol{oou}}\boldsymbol{\cdot}\boldsymbol{P}_{\boldsymbol{o}}$$

$$\frac{dP_{oa}}{dt}= a2\cdot P_{o}-\gamma_{a}\cdot P_{oa}+a1\cdot P_{a}-\gamma_{o}\cdot P_{oa}-a3\cdot P_{oa}+\gamma_{u}\cdot P_{oua}\boldsymbol{+}\boldsymbol{\lambda}_{\boldsymbol{ooa}}\boldsymbol{\cdot}\boldsymbol{P}_{\boldsymbol{o}}\boldsymbol{+}\boldsymbol{\lambda}_{\boldsymbol{aoa}}\boldsymbol{\cdot}\boldsymbol{P}_{\boldsymbol{a}}$$

$$\frac{dP_{ua}}{dt}=a2\cdot P_{u}-\gamma_{a}\cdot P_{ua}+a3\cdot P_{a}-\gamma_{u}\cdot P_{ua}-a1\cdot P_{ua}+\gamma_{o}\cdot P_{oua}\boldsymbol{+}\boldsymbol{\lambda}_{\boldsymbol{uua}}\boldsymbol{\cdot}\boldsymbol{P}_{\boldsymbol{u}}\boldsymbol{+}\boldsymbol{\lambda}_{\boldsymbol{aua}}\boldsymbol{\cdot}\boldsymbol{P}_{\boldsymbol{a}}$$

$$\frac{dP_{oua}}{dt}=a2\cdot P_{ou}-\gamma_{a}\cdot P_{oua}+a1\cdot P_{ua}-\gamma_{o}\cdot P_{oua}+a3\cdot P_{oa}-\gamma_{u}\cdot P_{oua}$$

**Estimating attributed to sexual practices**

$$incid.au=\lambda_{au}\cdot P_{a\_all}\cdot(Ps+ Po+ Pa+ Poa)$$

$$incid.ao=\lambda_{ao\cdot}P_{a\_all}\cdot(Ps+ Pu+ Pa+ Pua)$$

$$incid.ua=\lambda_{ua \cdot}P_{u\_all}\cdot(Ps+ Po+ Pu+ Pou)$$

$$incid.uo=\lambda_{uo \cdot}P_{u\_all}\cdot(Ps+ Pu+ Pa+ Pua)$$

$$incid.oa=\lambda_{oa \cdot}P_{o\_all}\cdot(Ps+ Po+ Pu+ Pou)$$

$$incid.ou=\lambda_{ou}{\cdot P}_{o\_all}\cdot(Ps+ Po+ Pa+ Poa)$$

$$incid.ooa=\lambda_{ooa \cdot}P_{o}$$

$$incid.aoa=\lambda_{aoa}\cdot P_{a}$$

$$incid.oou=\lambda_{oou\cdot}P_{o}$$

$$incid.ua2=\lambda_{ua2}{\cdot P}_{o}$$

**Incidence attributed to** **oropharyngeal, anorectal, and urethral infection**

$$incid.o= incid.ao+ incid.uo+ incid.aoa$$

$$incid.a= incid.ua+ incid.oa+ incid.ooa+ incid.ua2+ incid.uua$$

$$incid.u= incid.oou +incid.aua+incid.au+ incid.ou$$

***‘Anal sex, oral sex, rimming, kissing and sequential sexual practices’ transmission models***

$${a1=\lambda}_{oo}\boldsymbol{\cdot}P_{o\_all}+\lambda_{ao}{\boldsymbol{\cdot}P}_{a\_all}+\lambda_{uo}{\boldsymbol{\cdot}P}_{u\_all}$$

$$a2=\lambda_{oa}{\boldsymbol{\cdot}P}_{o\_all}+\lambda_{ua}{\boldsymbol{\cdot}P}_{u\_all}$$

$$a3=\lambda_{ou}{\boldsymbol{\cdot}P}_{o\_all}+\lambda_{au}{\boldsymbol{\cdot}P}_{a\_all}$$

$$\frac{dP_{s}}{dt}=-a1\cdot P_{s}+\gamma_{o}\cdot P_{o}-a2\cdot P_{s}+\gamma_{a}\cdot P_{a}-a3\cdot P_{s}+\gamma_{u}\cdot P_{u}- (beta.ohu2*Po\_all)$$

$$\frac{dP_{o}}{dt}=a1\cdot P_{s}-\gamma_{o}\cdot P_{o}-a2\cdot P_{o}+\gamma_{a}\cdot P_{oa}-a3\cdot P_{o}+\gamma_{u}\cdot P_{ou}-\boldsymbol{\lambda}_{\boldsymbol{ooa}}\boldsymbol{\cdot}\boldsymbol{P}_{\boldsymbol{o}}\boldsymbol{-}\boldsymbol{\lambda}_{\boldsymbol{oou}}\boldsymbol{\cdot}\boldsymbol{P}_{\boldsymbol{o}}$$

$$\frac{dP_{u}}{dt}=a3\cdot P_{s}-\gamma_{u}\cdot P_{u}-a1\cdot P_{u}+\gamma_{o}\cdot P_{ou}-a2\cdot P_{u}+\gamma_{a}\cdot P_{ua}-\boldsymbol{\lambda}_{\boldsymbol{uua}}\boldsymbol{\cdot}\boldsymbol{P}_{\boldsymbol{u}}-\boldsymbol{\lambda}_{\boldsymbol{ua}\boldsymbol{2}}\boldsymbol{\cdot}\boldsymbol{P}_{\boldsymbol{o}}$$

$$\frac{dP_{a}}{dt}=a2\cdot P_{s}-\gamma_{a}\cdot P_{a}-a1\cdot P_{a}+\gamma_{o}\cdot P_{oa}-a3\cdot P_{a}+\gamma_{u}\cdot P_{ua}\boldsymbol{-}\boldsymbol{\lambda}_{\boldsymbol{aoa}}\boldsymbol{\cdot}\boldsymbol{P}_{\boldsymbol{a}}\boldsymbol{-}\boldsymbol{\lambda}_{\boldsymbol{aua}}\boldsymbol{\cdot}\boldsymbol{P}_{\boldsymbol{a}}\boldsymbol{+}\boldsymbol{\lambda}_{\boldsymbol{ua}\boldsymbol{2}}\boldsymbol{\cdot}\boldsymbol{P}_{\boldsymbol{o}}$$

$$\frac{dP_{ou}}{dt}=a1\cdot P_{u}-\gamma_{o}\cdot P_{ou}+a3\cdot P_{o}-\gamma_{u}\cdot P_{ou}-a2\cdot P_{ou}+\gamma_{a}\cdot P_{oua}+\boldsymbol{\lambda}_{\boldsymbol{oou}}\boldsymbol{\cdot}\boldsymbol{P}_{\boldsymbol{o}}$$

$$\frac{dP_{oa}}{dt}= a2\cdot P_{o}-\gamma_{a}\cdot P_{oa}+a1\cdot P_{a}-\gamma_{o}\cdot P_{oa}-a3\cdot P_{oa}+\gamma_{u}\cdot P_{oua}\boldsymbol{+}\boldsymbol{\lambda}_{\boldsymbol{ooa}}\boldsymbol{\cdot}\boldsymbol{P}_{\boldsymbol{o}}\boldsymbol{+}\boldsymbol{\lambda}_{\boldsymbol{aoa}}\boldsymbol{\cdot}\boldsymbol{P}_{\boldsymbol{a}}$$

$$\frac{dP_{ua}}{dt}=a2\cdot P_{u}-\gamma_{a}\cdot P_{ua}+a3\cdot P_{a}-\gamma_{u}\cdot P_{ua}-a1\cdot P_{ua}+\gamma_{o}\cdot P_{oua}\boldsymbol{+}\boldsymbol{\lambda}_{\boldsymbol{uua}}\boldsymbol{\cdot}\boldsymbol{P}_{\boldsymbol{u}}\boldsymbol{+}\boldsymbol{\lambda}_{\boldsymbol{aua}}\boldsymbol{\cdot}\boldsymbol{P}_{\boldsymbol{a}}$$

$$\frac{dP_{oua}}{dt}=a2\cdot P_{ou}-\gamma_{a}\cdot P_{oua}+a1\cdot P_{ua}-\gamma_{o}\cdot P_{oua}+a3\cdot P_{oa}-\gamma_{u}\cdot P_{oua}$$

**Estimating attributed to sexual practices**

$$incid.au=\lambda_{au}\cdot P_{a\_all}\cdot(Ps+ Po+ Pa+ Poa)$$

$$incid.ao=\lambda_{ao\cdot}P_{a\_all}\cdot(Ps+ Pu+ Pa+ Pua)$$

$$incid.ua=\lambda_{ua \cdot}P_{u\_all}\cdot(Ps+ Po+ Pu+ Pou)$$

$$incid.uo=\lambda_{uo \cdot}P_{u\_all}\cdot(Ps+ Pu+ Pa+ Pua)$$

$$incid.oa=\lambda_{oa \cdot}P_{o\_all}\cdot(Ps+ Po+ Pu+ Pou)$$

$$incid.ou=\lambda_{ou}{\cdot P}_{o\_all}\cdot(Ps+ Po+ Pa+ Poa)$$

$$incid.oo=\lambda_{oo}{\cdot P}_{o\_all}\cdot(Ps+ Pu+ Pa+ Pua)$$

$$incid.ooa=\lambda_{ooa \cdot}P_{o}$$

$$incid.aoa=\lambda_{aoa}\cdot P_{a}$$

$$incid.oou=\lambda_{oou}\cdot P_{o}$$

$$incid.ua2=\lambda_{ua2}{\cdot P}_{o}$$

**Incidence attributed to** **oropharyngeal, anorectal, and urethral infection**

$$incid.o= incid.ao+ incid.uo+ incid.oo+incid.aoa$$

$$incid.a= incid.ua+ incid.oa+ incid.ooa+ incid.ua2+ incid.uua$$

$$incid.u= incid.oou +incid.aua+incid.au+ incid.ou$$

***Chlamydia trachomatis* model calibration**

We calibrated our models to the anatomical site-specific prevalence of chlamydia. Our model parameters were from published biological and behavioural data of chlamydia (Supplemental Table S3). The collected parameters of chlamydia models were sampled within the confidence interval by the Latin hypercube sampling method and repeated 300 times. For each set of parameters, we used ‘Nelder-Mead simplex direct search’ method to optimise the model when calibrating to the empirical prevalence. We used the least square method to estimate the goodness of fit between simulation output and observed chlamydia prevalence data. We then sorted the goodness of fit in descending order (best fit at the front), and the top 10% of 300 simulations were regarded as the best simulations, which were used to generate the chlamydia models outputs with 95% confidence intervals. The confidence intervals for those parameters of chlamydia models could not be obtained from published literature was estimated based on assumptions of binomial probability distributions [2-4]. Sparse Equations and Least Squares algorithm were used to calculate the sum of squared errors to assess the simulation performance of chlamydia models [5]. Based on each selected set of parameters of chlamydia model fitting, we used the ‘trust-region-reflective’ method for optimisation of a nonlinear function, we also fitthe weighted prevalence data using the built-in MATLAB least-squares fitting routine fmincon in the optimisation toolbox [6].

**‘Anal sex and oral sex and sequential sexual practices’ transmission model calibration**

To parameterize these 4 models (model 1, 4-6) , 23 parameters values and 95% CI are needed (Table S3, S5): **2** parameters related to condom (consistent condom usage, condom efficacy), **2** parameters related to sexual practices (frequency of oral sex, frequency of anal sex) , **3** parameters related to sequential sexual practices (Proportion of 'oral sex and anal sex' in the same sex episode, Proportion of saliva use during anal sex, the saliva is coming from the insertive (top) partner, proportion of receptive oral sex (from urethra to oropharynx) followed by partner's insertive anal sex (from urethra to anorectum)), **6** parameters related to the *Chlamydia trachomatis* infection recovery process (infection duration of *Chlamydia trachomatis* at the throat (asymptomatic infection), infection duration of *Chlamydia trachomatis* at urethral (symptomatic infection), infection duration of *Chlamydia trachomatis* at urethral (asymptomatic infection), proportion of urethral infections that are asymptomatic, proportion of anal *Chlamydia trachomatis* infections that are asymptomatic, infection duration of *Chlamydia trachomatis* at the anus), **3** parameters related to screening for sexually transmitted infections (proportion of MSM received throat swab in the past 12 months, proportion of MSM received anal swab in the past 12 months, proportion of MSM received urine test in the past 12 months), and **7** site-specific infection prevalence parameters (oropharynx only, urethral only, rectal only, oropharynx and urethra both, oropharynx and rectum both, urethra and rectum both, infection at all three sites).

**‘Anal sex and oral sex and rimming and sequential sexual practices’ transmission model calibration**

To parameterize these 8 models (model 2, 7-13) , 26 parameters values and 95% CI are needed (Table S3, S5): **2** parameters related to condom (consistent condom usage, condom efficacy), **3** parameters related to sexual practices (frequency of oral sex, frequency of rimming, frequency of anal sex) , **5** parameters related to sequential sexual practices (Proportion of 'oral sex and anal sex' in the same sex episode, Proportion of 'oral sex and rimming' in the same sex episode, Proportion of saliva use during anal sex, the saliva is coming from the insertive (top) partner, proportion of receptive oral sex (from urethra to oropharynx) followed by partner's insertive anal sex (from urethra to anorectum), proportion of insertive oral sex (from oropharynx to urethra) followed by partner's insertive rimming (from oropharynx to anorectum)), **6** parameters related to the *Chlamydia trachomatis* infection recovery process (infection duration of *Chlamydia trachomatis* at the throat (asymptomatic infection), infection duration of *Chlamydia trachomatis* at urethral (symptomatic infection), infection duration of *Chlamydia trachomatis* at urethral (asymptomatic infection), proportion of urethral infections that are asymptomatic, proportion of anal *Chlamydia trachomatis* infections that are asymptomatic, infection duration of *Chlamydia trachomatis* at the anus), **3** parameters related to screening for sexually transmitted infections (proportion of MSM received throat swab in the past 12 months, proportion of MSM received anal swab in the past 12 months, proportion of MSM received urine test in the past 12 months), and **7** site-specific infection prevalence parameters (oropharynx only, urethral only, rectal only, oropharynx and urethra both, oropharynx and rectum both, urethra and rectum both, infection at all three sites).

**‘Anal sex, oral sex, rimming, kissing and sequential sexual practices’ transmission models calibration**

To parameterize these 8 models (model 3, 14-20), 27 parameters values and 95%CI are needed (Table S3, S5): **2** parameters related to condom (consistent condom usage, condom efficacy), **4** parameters related to sexual practices (frequency of kissing, frequency of oral sex, frequency of rimming, frequency of anal sex), **5** parameters related to sequential sexual practices (Proportion of 'oral sex and anal sex' in the same sex episode, Proportion of 'oral sex and rimming' in the same sex episode, Proportion of saliva use during anal sex, the saliva is coming from the insertive (top) partner, proportion of receptive oral sex (from urethra to oropharynx) followed by partner's insertive anal sex (from urethra to anorectum), proportion of insertive oral sex (from oropharynx to urethra) followed by partner's insertive rimming (from oropharynx to anorectum)), **6** parameters related to the *Chlamydia trachomatis* infection recovery process (infection duration of *Chlamydia trachomatis* at the throat (asymptomatic infection), infection duration of *Chlamydia trachomatis* at urethral (symptomatic infection), infection duration of *Chlamydia trachomatis* at urethral (asymptomatic infection), proportion of urethral infections that are asymptomatic, proportion of anal *Chlamydia trachomatis* infections that are asymptomatic, infection duration of *Chlamydia trachomatis* at the anus), **3** parameters related to screening for sexually transmitted infections (proportion of MSM received throat swab in the past 12 months, proportion of MSM received anal swab in the past 12 months, proportion of MSM received urine test in the past 12 months), and **7** site-specific infection prevalence parameters (oropharynx only, urethral only, rectal only, oropharynx and urethra both, oropharynx and rectum both, urethra and rectum both, infection at all three sites).

**Uncertainty and sensitivity analysis**

We performed sensitivity analyses of the models that provided the best calibration of multisite infection (models 2, 7-13) using the whole parameter set. Details were described in ‘Anal sex and oral sex and rimming and sequential sexual practices’ transmission model calibration. First, we varied the duration of infection (half-length of asymptomatic urethral and anal duration). Second, we performed sensitivity analyses with varying the frequency of sexual practices (double-length or half-length days of sexual practices, including kissing, oral sex, rimming, and anal sex). Furthermore, we performed sensitivity analyses with varying the proportion of oral-penile sex followed by penile-anal sex and proportion of penile-oral sex followed by oral-anal sex (rimming) (proportion=0%, 95%, 98%, 100%).

1. Zhang L, Regan DG, Chow EPF, Gambhir M, Cornelisse V, Grulich A, Ong J, Lewis DA, Hocking J, Fairley CK: **Neisseria gonorrhoeae Transmission Among Men Who Have Sex With Men: An Anatomical Site-Specific Mathematical Model Evaluating the Potential Preventive Impact of Mouthwash**. *Sex Transm Dis* 2017, **44**(10):586-592.

2. Newcombe RG: **Two-sided confidence intervals for the single proportion: comparison of seven methods**. *Stat Med* 1998, **17**(8):857-872.

3. Hu FB: **Diet and exercise for new-onset type 2 diabetes?** *Lancet* 2011, **378**(9786):101-102.

4. Wilson EB: **Probable Inference, the Law of Succession, and Statistical Inference**. *Journal of the American Statistical Association* 1927, **22**(158):209-212.

5. Paige CC, Saunders MA: **LSQR: An Algorithm for Sparse Linear Equations and Sparse Least Squares**. *ACM Trans Math Softw* 1982, **8**(1):43-71.

6. Onwubu SC, Mdluli PS, Singh S, Collins OC: **The Application of the Logistic Equation Model to Predict the Remineralization Characteristics of Desensitizing Paste**. *Int J Dent* 2019, **2019**:7528154.
