# Supplementary material for: Chlamydia trachomatis transmission between the oropharynx, urethra and anorectum in men who have sex with men: a mathematical model
Source: BMC Med. 2020 Nov 17;18:326. doi: 10.1186/s12916-020-01796-3 (PMC7670797; doi:10.1186/s12916-020-01796-3)
Supplement: Supplementary file 2 — Additional file 2: Table S1. Definition of Chlamydia trachomatis models based on sexual practices. Table S2. Sexual practices included in the 20 Chlamydia trachomatis models. Table S3. Biological and behavioural data of Chlamydia trachomatis for model parameterization in MSM. Table S4. The anatomical site-specific infection prevalence of Chlamydia trachomatis in MSM attending MSHC in 2018 and 2019. Table S5. All anatomical site-specific infection prevalence datasets of Chlamydia trachomatis for model calibration and validation. Table S6. Output parameters and transmission probability of the best-calibrated model and baseline model based on data from 4888 MSM attending Melbourne Sexual Health Centre. Table S7. Sum of squared error of 20 Chlamydia trachomatis models on data of 4888 MSM attending Melbourne Sexual Health Centre in 2018 and 2019. [file 12916_2020_1796_MOESM2_ESM.docx]

**Table S1**. Definition of *Chlamydia trachomatis* models based on sexual practices

| **Models** | **Sexual practices** |
| --- | --- |
| **No transmission from sequential sexual practices** | |
| Model 1 | Oral sex and anal sex |
| Model 2 | Oral sex and anal sex and rimming |
| Model 3 | Oral sex and anal sex and rimming and kissing |
| Adding extra sequential sexual practices to three models without sequential sexual practices | |
| **‘Anal sex and oral sex and sequential sexual practices’ transmission models** | |
| Model 4 | Anal sex and oral sex and sequential oral/anal sex |
| Model 5 | Anal sex and oral sex and using saliva as a lubricant for anal sex |
| Model 6 | Anal sex and oral sex, sequential oral/anal sex and using saliva as a lubricant for anal sex |
| **‘Anal sex and oral sex and rimming and sequential sexual practices’ transmission models** | |
| Model 7 | Anal sex and oral sex, rimming and sequential oral/anal sex |
| Model 8 | Anal sex and oral sex and rimming and using saliva as a lubricant for anal sex |
| Model 9 | Anal sex and oral sex and rimming and sequential oral sex/riming |
| Model 10 | Anal sex and oral sex and rimming, sequential oral/anal sex and using saliva as a lubricant for anal sex |
| Model 11 | Anal sex and oral sex and rimming and sequential oral/anal sex and sequential oral sex/riming |
| Model 12 | Anal sex and oral sex and rimming and using saliva as a lubricant for anal sex and sequential oral sex/riming |
| Model 13 | Anal sex and oral sex and rimming and sequential oral/anal sex and using saliva as a lubricant for anal sex and sequential oral sex/riming |
| **‘Anal sex, oral sex, rimming, kissing and sequential sexual practices’ transmission models** | |
| Model 14 | Anal sex and oral sex and rimming and kissing and sequential oral/anal sex |
| Model 15 | Anal sex and oral sex and rimming and kissing and using saliva as a lubricant for anal sex |
| Model 16 | Anal sex and oral sex and rimming and kissing and sequential oral sex/riming |
| Model 17 | Anal sex and oral sex and rimming and kissing and sequential oral/anal sex and using saliva as a lubricant for anal sex |
| Model 18 | Anal sex and oral sex and rimming and kissing and sequential oral/anal sex and sequential oral sex/riming |
| Model 19 | Anal sex and oral sex and rimming and kissing and using saliva as a lubricant for anal sex and sequential oral sex/riming |
| Model 20 | Anal sex and oral sex and rimming and kissing and sequential oral/anal sex, using saliva as a lubricant for anal sex and sequential oral sex/riming |

**Table S2**. Sexual practices included in the 20 *Chlamydia trachomatis* models

|  | **Sequential sexual practices** | | | | | | | |
| --- | --- | --- | --- | --- | --- | --- | --- | --- |
|  | **No sequential sexual practices** | **Oral sex followed by anal sex (or reverse)** | **Saliva use as a lubricant for anal sex** | **Both of previous ones** | **Oral sex followed by rimming (or reverse)** | **Oral sex followed by rimming and Oral sex followed by anal sex (or reverse)** | **Oral sex followed by rimming and Saliva use as a lubricant for anal sex** | **All three sequential sexual practices** |
| **Simple practices** |  | Connects mouth and anus | Connects mouth and penis | Connects mouth to both anus and penis | Connects penis and anus | Connects anus to both penis and mouth | Connects all three anatomical sites |  |
| **Oral and anal sex only** | 1 | 4 | 5 | 6 | NA | NA | NA | NA |
| **Plus rimming** | 2 | 7 | 8 | 10 | 9 | 11 | 12 | 13 |
| **Plus kissing** | 3 | 14 | 15 | 17 | 16 | 18 | 19 | 20 |

**Table S 3**. Biological and behavioural data of *Chlamydia trachomatis* for model parameterization in MSM

| **Parameters** | **Value (95% confidence interval)** | **References/Remarks** |
| --- | --- | --- |
| %, Consistent condom usage in anal sex in past 12 months | 46.90(34.50- 59.30) | [1] |
| %, Condom efficacy in preventing transmission | 87.50(80.00-95.00) | [1, 2] |
| times, Frequency of kissing in the past 12 months | 6.31(0.00-13.12) | [1] |
| times, Frequency of oral sex in the past 12 months | 13.53(0.00-28.11) | [1] |
| times, Frequency of rimming in the past 12 months | 38.57(0.00-80.15) | [1] |
| times, Frequency of anal sex in the past 12 months | 26.44(0.00-54.94) | [1] |
| weeks, Infection duration of *Chlamydia trachomatis* at the throat (asymptomatic infection) | 95.29(24.87-245.44) | [3, 4]. |
| weeks, Infection duration of *Chlamydia trachomatis* at urethral (symptomatic infection) | 1.57(1.00-2.00) | Footnote *a*, [5] |
| weeks, Infection duration of *Chlamydia trachomatis* at urethral (asymptomatic infection) | 57.72(19.60-158.08) | [6] |
| %, Proportion of urethral infections that are asymptomatic | 85.00(75.00-95.00) | Footnote *b*, [7] |
| %, Proportion of anal *Chlamydia trachomatis* infections that are asymptomatic | 87.50(60.41-97.80) | Footnote *c*, [8] |
| weeks, Infection duration of *Chlamydia trachomatis* at the anus | 82.68(50.02-134.16) | [6] |
| %, Proportion of MSM received throat swab in the past 12 months | 79.65(63.70-95.60) | Footnote *d*, [9] |
| %, Proportion of MSM received anal swab in the past 12 months | 79.65(63.70-95.60) | Footnote *d*, [9] |
| %, Proportion of MSM received urine test in the past 12 months | 79.65(63.70-95.60) | Footnote *d*, [9] |
| %, Proportion of 'oral sex and anal sex' in the same sex episode | 29.41(24.82-34.00) | Footnote *e*, [10] |
| %, Proportion of 'oral sex and rimming' in the same sex episode | 70.5 (67.94-72.94) | Footnote *f,* [11] |
| %, Proportion of saliva use during anal sex, the saliva is coming from the insertive (top) partner | 68.52(65.92-71.01) | [11] |
| %, Proportion of receptive oral sex (individual’s mouth in contact with a partner’s penis) followed by partner's insertive anal sex (individual’ penis in contact with partner’ anus) | 80.00(80.00-80.00) | Footnote *g* [12] |
| %, Proportion of insertive oral sex (individual’s penis in contact with a partner’s mouth) followed by partner's insertive rimming (individual’s mouth in contact with a partner’s anus) | 80.00(80.00-80.00) | **Estimate from Melbourne Sexual Health Centre** |

**Footnote**:

1. The duration time is estimated by the time between the first symptom and treatment. Among Men, the mean time was 11 Days =1.57 weeks, range (7-14) days.
2. We estimated the value from a previous study. A case-control study of MSM attending Seattle & King County STD Clinic between 2001 and 2013 found that clinicians diagnosed a total of 6464 urethral infections, only 61% of chlamydial infections were symptomatic and asymptomatic chlamydial infections were 39%.
3. 16 MSM was infected with anal *Chlamydia trachomatis*, of which 14 were asymptomatic.
4. We used the proportion of gay and bisexual men attending sexual health clinic for a chlamydia test in 2017 (95.60%) as the upper bound. We used the proportion of gay and bisexual men attending general practice clinic tested for chlamydia in 2017 (63.70%) as the lower bound. The mean value of proportion = (upper bound+ lower bound)/2.
5. The proportion of 'oral sex and anal sex' in the same sex episode was not available from published data. We used the prevalence of insertive anal sex (34.00%) as the upper bound. We also used the value of the prevalence of anal sex (34.00%) multiply the prevalence of oral sex (73.00%) as the lower bound. The mean value of proportion = (upper bound+ lower bound)/2.
6. The proportion of 'oral sex and rimming' in the same sex episode was not available from published data. About 70.5% (95%CI67.94-72.94) reported rimming. According to the advice of sexually transmitted infections specialist at the Melbourne Sexual Health Centre, rimming is always accompanied by oral sex. We are using the mean value and 95%CI for rimming as the mean and bound for the proportion of 'oral sex and rimming' in the same sex episode.
7. The proportion of receptive oral sex (individual’ mouth in contact with partner’ penis) followed by partner's insertive anal sex (individual’ penis in contact with partner’ anus) was not available from published data. The sequence of receptive oral sex followed by partner's insertive anal sex was most common among oral sex and anal sex combinations. Based on the advice of sexually transmitted infections specialist at the Melbourne Sexual Health Centre, we made the assumption.
8. The confidence intervals for those parameters of chlamydia models could not be obtained from published literature was estimated based on assumptions of binomial probability distributions [13-15] .

**Table S4**. The anatomical site-specific infection prevalence of *Chlamydia trachomatis* in MSM attending MSHC in 2018 and 2019

|  | **2018(N=2565)** | **2019(N=2323)** | **2018 and 2019 (N=4888)** |
| --- | --- | --- | --- |
|  | N, % | N, % | N, % |
| Oropharynx only | 14 (0.54) | 23 (0.99) | 37 (0.76) |
| Urethral only | 40 (1.55) | 51 (2.19) | 91(1.86) |
| Rectal only | 189 (7.36) | 179 (7.70) | 368(7.53) |
| Oropharynx and urethra co-infection | 0 | 0 | 0 |
| Oropharynx and rectum co-infection | 24 (0.93) | 34 (1.46) | 58(1.19) |
| Urethra and rectum co-infection | 33 (1.28) | 25 (1.07) | 58(1.19) |
| Oropharynx and urethra and rectum | 2 (0.10) | 6 (0.31) | 8(0.16) |

**Table S 5.** All anatomical site-specific infection prevalence datasets of *Chlamydia trachomatis* for model calibration and validation

| **Continents** | | | **Value (95%** **confidence interval)** | | | | | | |
| --- | --- | --- | --- | --- | --- | --- | --- | --- | --- |
|  |  |  | **Single-site infections** | | | **Multisite infections** | | | |
|  |  |  | Oropharynx only | Urethral only | Rectal  only | Oropharynx and urethra both | Oropharynx and rectum both | Urethra and rectum both | Oropharynx and urethra and rectum both |
| Country/region | Duration | Sample size |  |  |  |  |  |  |  |
| **Calibration dataset** | | | | | | | | | |
| *Footnote a,* Australia/Melbourne | 2018 and 2019 | 4888 | 0.76  (0.54-1.06) | 1.86  (1.51-2.29) | 7.53  (6.81-8.31) | *Footnote b,*  0.00  (0.00-0.00) | 1.19  (0.91-1.55) | 1.19  (0.91-1.55) | 0.16  (0.07-0.33) |
| **Validation dataset** | | | | | | | | | |
| Validation dataset 1  Australia/Melbourne (Asymptomatic MSM)[16] | 2016–2017 | 1,011 | 0.97  (0.31-2.64) | 1.34  (0.75-2.35) | 7.23  (5.72-9.09) | *Footnote b,*  0.00  (0.-0.00) | 1.96  (0.91-3.98) | 0.42  (0.14-1.15) | *Footnote b,*  0.00  (0.-0.00) |
| Validation dataset 2  North America/ USA[17, 18] |  | 393 | 0.51  (0.09-2.03) | 2.29  (1.12-4.46) | 6.36  (4.24-9.37) | *Footnote b,*  0.00  (0.-0.00) | 1.53  (0.62-3.47) | 0.76  (0.20-2.40) | *Footnote b,*  0.00  (0.00-0.00) |
| Validation dataset 3  Europe/Dutch[19] | 2008-2017 |  | 0.51  (0.48-0.54) | 2.30  (2.23-2.37) | 6.29  (6.17-6.41) | 0.06  (0.05-0.07) | 0.54  (0.51-0.58) | 1.02  (0.97-1.07) | 0.10  (0.09-0.12) |
| Validation dataset 4  Asia /Thailand[20] | 2015-2016 | 1610 | 1.74  (1.18-2.54) | 4.84  (3.87-6.03) | 11.86  (10.34-13.56) | 0.06  (0.00-0.40) | 1.76  (0.69-1.80) | 1.99  (1.39-2.83) | 0.06  (0.00-0.40) |
| Validation dataset 5  North America/ USA (HIV+)[17, 18] |  | 179 | *Footnote* b, 0.00  (0.-0.00) | 1.68  (0.44-5.22) | 12.85  (8.49-18.86) | *Footnote b,*  0.00  (0.-0.00) | *Footnote b,*  0.00  (0.-0.00) | 1.12  (0.19-4.41) | *Footnote b,*  0.00  (0.-0.00) |

**Footnote:**

1. MSHC started screening for throat chlamydia in mid-2017, so we used chlamydia data for 2018-19.
2. Since the positivity is zero, we assumed the upper bound of 95% confidence interval was 1/1000000 (one person in a million) and the lower bound of 95% was 0.0000000 in the simulation.

**Table S 6**. Sum of squared error of 20 *Chlamydia trachomatis* models on data of 4888 MSM attending Melbourne Sexual Health Centre in 2018 and 2019

| **Models** | **Sexual practices** | **Sum of Squared Error (×10^-3^), *Footnote* a** | **Statistical analysis, *Footnote* c** |
| --- | --- | --- | --- |
|  |  | **Mean, 95% Confidence Interval, *Footnote* b** |  |
| **No transmission sequential sexual practices** | | | |
| Model 1 | Oral sex and anal sex | 0.197 (0.170-0.204) | Reference group |
| Model 2 | Oral sex and anal sex and rimming | 0.201 (0.157-0.215) | Model 2 vs. Model 1 (p=0.1595) |
| Model 3 | Oral sex and anal sex and rimming and kissing | 0.205 (0.155-0.215) | Model 3 vs. Model 1 (p=0.1031) |
| **‘Anal sex and oral sex and sequential sexual practices’ transmission models** | | | |
| Model 1 | Oral sex and anal sex | 0.197 (0.170-0.204) | Reference group |
| Model 4 | Anal sex and oral sex and ***sequential oral/anal sex*** | 0.093 (0.074-0.103) | Model 4 vs. Model 1 (p < 0.01) |
| Model 5 | Anal sex and oral sex and ***using saliva as a lubricant for anal sex*** | 0.199 (0.178-0.212) | Model 5 vs. Model 1 (p = 0.4775) |
| Model 6 | Anal sex and oral sex, ***sequential oral/anal sex and using saliva as a lubricant for anal sex*** | 0.116 (0.096-0.125) | Model 6 vs. Model 1 (p < 0.01) |
| **‘Anal sex and oral sex and rimming and sequential sexual practices’ transmission models** | | | |
| Model 2 | Oral sex and anal sex and rimming | 0.201 (0.157-0.215) | Reference group |
| Model 7 | Anal sex and oral sex, rimming and ***sequential oral/anal sex*** | 0.102 (0.080-0.115) | Model 7 vs. Model 2 (p < 0.001) |
| Model 8 | Anal sex and oral sex and rimming and ***using saliva as a lubricant for anal sex*** | 0.219 (0.183-0.232) | Model 8 vs. Model 2 (p < 0.001) |
| Model 9 | Anal sex and oral sex and rimming and ***sequential oral sex/riming*** | 0.102 (0.093-0.112) | Model 9 vs. Model 2 (p < 0.001) |
| Model 10 | Anal sex and oral sex and rimming, ***sequential oral/anal sex and using saliva as a lubricant for anal sex*** | 0.129 (0.107-0.140) | Model 10 vs. Model 2 (p < 0.001) |
| Model 11 | Anal sex and oral sex and rimming and ***sequential oral/anal sex and sequential oral sex/riming*** | **0.043 (0.028-0.046), *Footnote* d** | Model 11 vs. Model 2 (p < 0.001) |
| Model 12 | Anal sex and oral sex and rimming and ***using saliva as a lubricant for anal sex and sequential oral sex/riming*** | 0.136 (0.124-0.144) | Model 12 vs. Model 2 (p < 0.001) |
| Model 13 | Anal sex and oral sex and rimming and ***sequential oral/anal sex and using saliva as a lubricant for anal sex and sequential oral sex/riming*** | 0.089 (0.067-0.100 | Model 13 vs. Model 2(p < 0.001) |
| **‘Anal sex, oral sex, rimming, kissing and sequential sexual practices’ transmission models** | | | |
| Model 3 | Oral sex and anal sex and rimming and kissing | 0.205 (0.155-0.215) | Reference group |
| Model 14 | Anal sex and oral sex and rimming and kissing and ***sequential oral/anal sex*** | 0.106 (0.089-0.115) | Model 14 vs. Model 3 (p < 0.001) |
| Model 15 | Anal sex and oral sex and rimming and kissing and ***using saliva as a lubricant for anal sex*** | 0.217 (0.186-0.237) | Model 15 vs. Model 3 (p < 0.001) |
| Model 16 | Anal sex and oral sex and rimming and kissing and ***sequential oral sex/riming*** | 0.104 (0.088-0.114) | Model 16 vs. Model 3 (p < 0.001) |
| Model 17 | Anal sex and oral sex and rimming and kissing and ***sequential oral/anal sex and using saliva as a lubricant for anal sex*** | 0.145 (0.115-0.154) | Model 17 vs. Model 3 (p < 0.001) |
| Model 18 | Anal sex and oral sex and rimming and kissing and ***sequential oral/anal sex and sequential oral sex/riming*** | **0.043 (0.034-0.049)** | Model 18 vs. Model 3 (p < 0.001) |
| Model 19 | Anal sex and oral sex and rimming and kissing and ***using saliva as a lubricant for anal sex and sequential oral sex/riming*** | 0.123 (0.096-0.132) | Model 19 vs. Model 3 (p < 0.001) |
| Model 20 | Anal sex and oral sex and rimming and kissing and ***sequential oral/anal sex, using saliva as a lubricant for anal sex and sequential oral sex/riming*** | 0.072 (0.056-0.077) | Model 20 vs. Model 3 (p < 0.001) |

**Footnote:**

1. Sum of squared error measures the total deviation of the calibrated seven prevalence values (single-site infections including only oropharynx infection, only anorectal infection, only urethral infection, and multi-site infections including multi-site infection at oropharyngeal and urethral only, multi-site infection at oropharynx and rectum only, multi-site infection at urethral and rectal only, and multi-site infection at urethral and anorectal and oropharyngeal) and the fit to the observed seven prevalence values.
2. Values close to 0 indicate that the model has a smaller error.
3. T-test was used to determine if there was a significant difference between the means of the sum of squared error between two models.
4. Model 11 and model 18 had no statistically significant differences in the means of the sum of squared error (p= 0.0869).

**Table S7**. Output parameters and transmission probability of the best-calibrated model and baseline model based on data from 4888 MSM attending Melbourne Sexual Health Centre

| **Parameters** | **Model 2(Oral sex and anal sex and rimming)** | | | **Model 11(Anal sex and oral sex and rimming and sequential oral/anal sex and sequential oral sex/riming)** | | |
| --- | --- | --- | --- | --- | --- | --- |
|  | **Mean** | **95%CI** | | **Mean** | **95%CI** | |
| %, Consistent condom usage in anal sex in past 12 months | 44.92 | 35.37 | 56.36 | 47.90 | 43.65 | 51.78 |
| %, Condom efficacy in preventing transmission | 87.18 | 80.81 | 93.81 | 87.21 | 83.82 | 90.01 |
| Times, Frequency of kissing in the past 12 months | - | - | - | - | - | - |
| Times, Frequency of oral sex in the past 12 months | 15.27 | 2.44 | 27.70 | 18.81 | 14.28 | 24.89 |
| Times, Frequency of rimming in the past 12 months | 28.08 | 4.93 | 76.16 | 41.23 | 23.91 | 57.13 |
| Times, Frequency of anal sex in the past 12 months | 13.43 | 1.16 | 39.12 | 13.93 | 7.83 | 18.96 |
| weeks, Infection duration of chlamydia trachomatis at throat (asymptomatic infection, weeks) | 112.41 | 32.72 | 218.24 | 143.90 | 93.41 | 188.23 |
| weeks, Infection duration at urethral (symptomatic infection) | 1.49 | 1.05 | 1.93 | 1.53 | 1.31 | 1.69 |
| %, proportion of urethral infections that are asymptomatic | 85.56 | 81.38 | 89.29 | 85.33 | 83.15 | 87.03 |
| %, proportion of anal infections that are asymptomatic | 80.49 | 65.95 | 96.31 | 82.23 | 74.30 | 87.30 |
| weeks, Infection duration at urethral (asymptomatic infection) | 28.32 | 6.20 | 47.55 | 16.95 | 10.61 | 23.17 |
| weeks, Infection duration of chlamydia trachomatis at anus (asymptomatic infection) | 50.38 | 48.20 | 51.73 | 49.55 | 48.86 | 50.25 |
| weeks, Infection duration of chlamydia trachomatis at anus (symptomatic infection) | 1.59 | 1.14 | 1.95 | 1.56 | 1.29 | 1.76 |
| %, proportion of MSM received throat swab in the past 12 months | 81.12 | 65.11 | 91.11 | 78.94 | 72.09 | 85.64 |
| %, proportion of MSM received anal swab in the past 12 months | 78.05 | 64.78 | 92.65 | 78.87 | 70.06 | 84.87 |
| %, proportion of MSM received urine test in the past 12 months | 77.76 | 64.71 | 92.37 | 79.58 | 71.12 | 88.29 |
| %, proportion of 'oral sex and anal sex' in the same sex episode | - | - | - | 29.82 | 27.50 | 31.91 |
| %, proportion of 'oral sex and rimming' in the same sex episode | - | - | - | 71.50 | 70.96 | 72.07 |
| %, proportion of saliva use during anal sex | - | - | - | 68.45 | 67.35 | 69.98 |
| %, proportion of receptive oral sex followed by partner's insertive anal sex | - | - | - | 80.00 | 80.00 | 80.00 |
| %, proportion of insertive oral sex followed by partner's insertive rimming | - | - | - | 80.00 | 80.00 | 80.00 |
| %, Infection prevalence of chlamydia trachomatis only at Oropharynx | 0.84 | 0.63 | 1.02 | 0.81 | 0.69 | 0.90 |
| %, Infection prevalence of chlamydia trachomatis only at Urethral | 1.95 | 1.59 | 2.25 | 1.97 | 1.82 | 2.17 |
| %, Infection prevalence of chlamydia trachomatis only at Rectum | 7.54 | 6.89 | 8.25 | 7.46 | 7.12 | 7.88 |
| %, Infection prevalence of chlamydia trachomatis at Oropharynx & Urethra | 0.00 | 0.00 | 0.00 | 0.00 | 0.00 | 0.00 |
| %, Infection prevalence of chlamydia trachomatis at Oropharynx & Rectum | 1.20 | 0.95 | 1.51 | 1.22 | 1.11 | 1.33 |
| %, Infection prevalence of chlamydia trachomatis at Urethra & Rectum | 1.18 | 0.97 | 1.53 | 1.23 | 1.08 | 1.40 |
| %, Infection prevalence of chlamydia trachomatis at Oropharynx & Urethra & Rectum | 0.22 | 0.09 | 0.31 | 0.19 | 0.15 | 0.24 |
| Transmission probability of anal sex (Urethra to Anorectum) | 0.074 | 0.017 | 0.367 | 0.044 | 0.019 | 0.190 |
| Transmission probability of rimming (Anorectum to Oropharynx) | 0.014 | 0.001 | 0.051 | 0.024 | 0.008 | 0.055 |
| Transmission probability of anal sex (Anorectum to Urethra) | 0.538 | 0.289 | 0.736 | 0.743 | 0.316 | 0.836 |
| Transmission probability of oral sex (Urethra to Oropharynx) | 0.030 | 0.005 | 0.102 | 0.032 | 0.014 | 0.093 |
| Transmission probability of rimming (Oropharynx to Anorectum) | 0.550 | 0.058 | 0.959 | 0.362 | 0.148 | 0.662 |
| Transmission probability of oral sex (Oropharynx to Urethra) | 0.138 | 0.033 | 0.377 | 0.057 | 0.016 | 0.187 |

1. Zhang L, Regan DG, Chow EPF, Gambhir M, Cornelisse V, Grulich A, Ong J, Lewis DA, Hocking J, Fairley CK: **Neisseria gonorrhoeae Transmission Among Men Who Have Sex With Men: An Anatomical Site-Specific Mathematical Model Evaluating the Potential Preventive Impact of Mouthwash**. *Sex Transm Dis* 2017, **44**(10):586-592.

2. Tuli K, Kerndt PR: **Preventing sexually transmitted infections among incarcerated men who have sex with men: a cost-effectiveness analysis**. *Sex Transm Dis* 2009, **36**(2 Suppl):S41-48.

3. Templeton DJ, Jin F, Imrie J, Prestage GP, Donovan B, Cunningham PH, Kaldor JM, Kippax S, Grulich AE: **Prevalence, incidence and risk factors for pharyngeal chlamydia in the community based Health in Men (HIM) cohort of homosexual men in Sydney, Australia**. *Sex Transm Infect* 2008, **84**(5):361-363.

4. Chow EP, Camilleri S, Ward C, Huffam S, Chen MY, Bradshaw CS, Fairley CK: **Duration of gonorrhoea and chlamydia infection at the pharynx and rectum among men who have sex with men: a systematic review**. *Sex Health* 2016, **13**(3):199-204.

5. Andersen B, Gundgaard J, Kretzschmar M, Olsen J, Welte R, Oster-Gaard L: **Prediction of costs, effectiveness, and disease control of a population-based program using home sampling for diagnosis of urogenital Chlamydia trachomatis Infections**. *Sex Transm Dis* 2006, **33**(7):407-415.

6. Jin F, Prestage GP, Mao L, Kippax SC, Pell CM, Donovan B, Cunningham PH, Templeton DJ, Kaldor JM, Grulich AE: **Incidence and risk factors for urethral and anal gonorrhoea and chlamydia in a cohort of HIV-negative homosexual men: the Health in Men Study**. *Sex Transm Infect* 2007, **83**(2):113-119.

7. Barbee LA, Khosropour CM, Dombrowski JC, Manhart LE, Golden MR: **An estimate of the proportion of symptomatic gonococcal, chlamydial and non-gonococcal non-chlamydial urethritis attributable to oral sex among men who have sex with men: a case-control study**. *Sex Transm Infect* 2016, **92**(2):155-160.

8. Rebe K, Lewis D, Myer L, de Swardt G, Struthers H, Kamkuemah M, McIntyre J: **A Cross Sectional Analysis of Gonococcal and Chlamydial Infections among Men-Who-Have-Sex-with-Men in Cape Town, South Africa**. *PLoS One* 2015, **10**(9):e0138315.

9. **HIV, viral hepatitis and sexually transmissible infections in Australia: annual surveillance report 2018.** [<https://kirby.unsw.edu.au/report/hiv-viral-hepatitis-and-sexually-transmissible-infections-australia-annual-surveillance>]

10. Fairley CK, Cornelisse VJ, Hocking JS, Chow EPF: **Models of gonorrhoea transmission from the mouth and saliva**. *The Lancet Infectious Diseases* 2019.

11. Chow EPF, Cornelisse VJ, Read TRH, Lee D, Walker S, Hocking JS, Chen MY, Bradshaw CS, Fairley CK: **Saliva use as a lubricant for anal sex is a risk factor for rectal gonorrhoea among men who have sex with men, a new public health message: a cross-sectional survey**. *Sex Transm Infect* 2016, **92**(7):532-536.

12. Rosenberger JG, Reece M, Schick V, Herbenick D, Novak DS, Van Der Pol B, Fortenberry JD: **Sexual behaviors and situational characteristics of most recent male-partnered sexual event among gay and bisexually identified men in the United States**. *J Sex Med* 2011, **8**(11):3040-3050.

13. Newcombe RG: **Two-sided confidence intervals for the single proportion: comparison of seven methods**. *Stat Med* 1998, **17**(8):857-872.

14. Hu FB: **Diet and exercise for new-onset type 2 diabetes?** *Lancet* 2011, **378**(9786):101-102.

15. Wilson EB: **Probable Inference, the Law of Succession, and Statistical Inference**. *Journal of the American Statistical Association* 1927, **22**(158):209-212.

16. Read TRH, Murray GL, Danielewski JA, Fairley CK, Doyle M, Worthington K, Su J, Mokany E, Tan LT, Lee D *et al*: **Symptoms, Sites, and Significance of Mycoplasma genitalium in Men Who Have Sex with Men**. *Emerg Infect Dis* 2019, **25**(4):719-727.

17. Footman A, Dionne-Odom J, Aaron KJ, Raper JL, Van Der Pol B: **Performance of 4 Molecular Assays for Detection of Chlamydia and Gonorrhea in a Sample of Human Immunodeficiency Virus-Positive Men Who Have Sex With Men**. *Sex Transm Dis* 2020, **47**(3):158-161.

18. BVD P: **Extragenital CT/GC in MSM**. In: *The 20th International Union against Sexually Transmitted Infections- Asia Pacific Conference (IUSTI-AP): 2019; Shanghai*; 2019.

19. van Liere G, Dukers-Muijrers N, Wessel SK, Gotz HM, Hoebe C: **What is the optimal testing strategy for oropharyngeal Neisseria gonorrhoeae in men who have sex with men? Comparing selective testing versus routine universal testing from Dutch STI clinic data (2008-2017)**. *Clin Infect Dis* 2019.

20. Hiransuthikul A, Sungsing T, Jantarapakde J, Trachunthong D, Mills S, Vannakit R, Phanuphak P, Phanuphak N: **Correlations of chlamydia and gonorrhoea among pharyngeal, rectal and urethral sites among Thai men who have sex with men: multicentre community-led test and treat cohort in Thailand**. *BMJ Open* 2019, **9**(6):e028162.
