# Supplementary material for: Chlamydia trachomatis transmission between the oropharynx, urethra and anorectum in men who have sex with men: a mathematical model
Source: BMC Med. 2020 Nov 17;18:326. doi: 10.1186/s12916-020-01796-3 (PMC7670797; doi:10.1186/s12916-020-01796-3)
Supplement: Supplementary file 3 — Additional file 3. Output parameters for Chlamydia trachomatis models. Tables S 8.1-8.219. [file 12916_2020_1796_MOESM3_ESM.docx]

**Output parameters for *Chlamydia trachomatis* models**

**Transmission models without considering sequential sexual practices (model 1-3)**

Model output parameters (Calibration Dataset): Unpublished calibration data from 4888 MSM attending Melbourne Sexual Health Centre

**Table S 8.1**. Model 1’ output parameters with 95% confidence intervals

| **Mean** | **95%CI: lower** | **95%CI: upper** | **Parameters** |
| --- | --- | --- | --- |
| 43.91 | 35.89 | 56.88 | %, Consistent condom usage in anal sex in past 12 months |
| 88.12 | 81.32 | 94.44 | %, Condom efficacy in preventing transmission |
| - | - | - | Times, Frequency of kissing in the past 12 months |
| 16.49 | 5.43 | 27.52 | Times, Frequency of oral sex in the past 12 months |
| - | - | - | Times, Frequency of rimming in the past 12 months |
| 7.19 | 1.63 | 23.03 | Times, Frequency of anal sex in the past 12 months |
| 118.64 | 31.37 | 215.74 | weeks, Infection duration of chlamydia trachomatis at throat (asymptomatic infection) |
| 1.44 | 1.14 | 1.92 | weeks, Infection duration at urethral (symptomatic infection) |
| 85.13 | 80.84 | 88.06 | %, proportion of urethral infections that are asymptomatic |
| 82.40 | 65.34 | 95.55 | %, proportion of anal infections that are asymptomatic |
| 31.23 | 3.91 | 49.83 | weeks, Infection duration at urethral (asymptomatic infection) |
| 49.98 | 48.46 | 51.53 | weeks, Infection duration of chlamydia trachomatis at anus (asymptomatic infection) |
| 1.51 | 1.10 | 1.89 | weeks, Infection duration of chlamydia trachomatis at anus (symptomatic infection) |
| 76.52 | 66.42 | 92.98 | %, proportion of MSM received throat swab in the past 12 months |
| 82.80 | 66.38 | 91.53 | %, proportion of MSM received anal swab in the past 12 months |
| 80.09 | 67.22 | 93.58 | %, proportion of MSM received urine test in the past 12 months |
| - | - | - | %, proportion of 'oral sex and anal sex' in the same sex episode |
| - | - | - | %, proportion of 'oral sex and rimming' in the same sex episode |
| - | - | - | %, proportion of saliva use during anal sex |
| - | - | - | %, proportion of receptive oral sex followed by partner's insertive anal sex |
| - | - | - | %, proportion of insertive oral sex followed by partner's insertive rimming |
| 0.71 | 0.57 | 0.97 | %, Infection prevalence of chlamydia trachomatis only at Oropharynx |
| 1.84 | 1.57 | 2.16 | %, Infection prevalence of chlamydia trachomatis only at Urethral |
| 7.35 | 6.88 | 8.06 | %, Infection prevalence of chlamydia trachomatis only at Rectum |
| 0.00 | 0.00 | 0.00 | %, Infection prevalence of chlamydia trachomatis at Oropharynx & Urethra |
| 1.26 | 0.97 | 1.51 | %, Infection prevalence of chlamydia trachomatis at Oropharynx & Rectum |
| 1.21 | 0.99 | 1.47 | %, Infection prevalence of chlamydia trachomatis at Urethra & Rectum |
| 0.20 | 0.11 | 0.31 | %, Infection prevalence of chlamydia trachomatis at Oropharynx & Urethra & Rectum |

**Table S8.2.**  Model 2’ output parameters with 95% confidence intervals

| **Mean** | **95%CI: lower** | **95%CI: upper** | **Parameters** |
| --- | --- | --- | --- |
| 44.92 | 35.37 | 56.36 | %, Consistent condom usage in anal sex in past 12 months |
| 87.18 | 80.81 | 93.81 | %, Condom efficacy in preventing transmission |
| - | - | - | Times, Frequency of kissing in the past 12 months |
| 15.27 | 2.44 | 27.70 | Times, Frequency of oral sex in the past 12 months |
| 28.08 | 4.93 | 76.16 | Times, Frequency of rimming in the past 12 months |
| 13.43 | 1.16 | 39.12 | Times, Frequency of anal sex in the past 12 months |
| 112.41 | 32.72 | 218.24 | weeks, Infection duration of chlamydia trachomatis at throat (asymptomatic infection) |
| 1.49 | 1.05 | 1.93 | weeks, Infection duration at urethral (symptomatic infection) |
| 85.56 | 81.38 | 89.29 | %, proportion of urethral infections that are asymptomatic |
| 80.49 | 65.95 | 96.31 | %, proportion of anal infections that are asymptomatic |
| 28.32 | 6.20 | 47.55 | weeks, Infection duration at urethral (asymptomatic infection) |
| 50.38 | 48.20 | 51.73 | weeks, Infection duration of chlamydia trachomatis at anus (asymptomatic infection) |
| 1.59 | 1.14 | 1.95 | weeks, Infection duration of chlamydia trachomatis at anus (symptomatic infection) |
| 81.12 | 65.11 | 91.11 | %, proportion of MSM received throat swab in the past 12 months |
| 78.05 | 64.78 | 92.65 | %, proportion of MSM received anal swab in the past 12 months |
| 77.76 | 64.71 | 92.37 | %, proportion of MSM received urine test in the past 12 months |
| - | - | - | %, proportion of 'oral sex and anal sex' in the same sex episode |
| - | - | - | %, proportion of 'oral sex and rimming' in the same sex episode |
| - | - | - | %, proportion of saliva use during anal sex |
| - | - | - | %, proportion of receptive oral sex followed by partner's insertive anal sex |
| - | - | - | %, proportion of insertive oral sex followed by partner's insertive rimming |
| 0.84 | 0.63 | 1.02 | %, Infection prevalence of chlamydia trachomatis only at Oropharynx |
| 1.95 | 1.59 | 2.25 | %, Infection prevalence of chlamydia trachomatis only at Urethral |
| 7.54 | 6.89 | 8.25 | %, Infection prevalence of chlamydia trachomatis only at Rectum |
| 0.00 | 0.00 | 0.00 | %, Infection prevalence of chlamydia trachomatis at Oropharynx & Urethra |
| 1.20 | 0.95 | 1.51 | %, Infection prevalence of chlamydia trachomatis at Oropharynx & Rectum |
| 1.18 | 0.97 | 1.53 | %, Infection prevalence of chlamydia trachomatis at Urethra & Rectum |
| 0.22 | 0.09 | 0.31 | %, Infection prevalence of chlamydia trachomatis at Oropharynx & Urethra & Rectum |

**Table S8.3**. Model 3’ output parameters with 95% confidence intervals

| **Mean** | **95%CI: lower** | **95%CI: upper** | **Parameters** |
| --- | --- | --- | --- |
| 45.36 | 35.44 | 56.03 | %, Consistent condom usage in anal sex in past 12 months |
| 89.18 | 80.65 | 93.70 | %, Condom efficacy in preventing transmission |
| 5.07 | 0.84 | 12.10 | Times, Frequency of kissing in the past 12 months |
| 15.14 | 2.49 | 27.82 | Times, Frequency of oral sex in the past 12 months |
| 30.29 | 3.04 | 75.07 | Times, Frequency of rimming in the past 12 months |
| 16.99 | 2.11 | 47.78 | Times, Frequency of anal sex in the past 12 months |
| 119.84 | 31.07 | 210.10 | weeks, Infection duration of chlamydia trachomatis at throat(asymptomatic infection) |
| 1.38 | 1.04 | 1.94 | weeks, Infection duration at urethral (symptomatic infection) |
| 84.52 | 81.26 | 89.33 | %, proportion of urethral infections that are asymptomatic |
| 81.53 | 66.81 | 95.69 | %, proportion of anal infections that are asymptomatic |
| 22.64 | 7.23 | 50.70 | weeks, Infection duration at urethral (asymptomatic infection) |
| 50.02 | 48.43 | 51.75 | weeks, Infection duration of chlamydia trachomatis at anus (asymptomatic infection) |
| 1.51 | 1.03 | 1.99 | weeks, Infection duration of chlamydia trachomatis at anus (symptomatic infection) |
| 81.74 | 65.60 | 94.05 | %, proportion of MSM received throat swab in the past 12 months |
| 77.50 | 64.47 | 92.09 | %, proportion of MSM received anal swab in the past 12 months |
| 80.67 | 67.25 | 92.54 | %, proportion of MSM received urine test in the past 12 months |
| - | - | - | %, proportion of 'oral sex and anal sex' in the same sex episode |
| - | - | - | %, proportion of 'oral sex and rimming' in the same sex episode |
| - | - | - | %, proportion of saliva use during anal sex |
| - | - | - | %, proportion of receptive oral sex followed by partner's insertive anal sex |
| - | - | - | %, proportion of insertive oral sex followed by partner's insertive rimming |
| 0.83 | 0.63 | 1.01 | %, Infection prevalence of chlamydia trachomatis only at Oropharynx |
| 1.87 | 1.54 | 2.20 | %, Infection prevalence of chlamydia trachomatis only at Urethral |
| 7.59 | 6.85 | 8.11 | %, Infection prevalence of chlamydia trachomatis only at Rectum |
| 0.00 | 0.00 | 0.00 | %, Infection prevalence of chlamydia trachomatis at Oropharynx & Urethra |
| 1.14 | 0.93 | 1.47 | %, Infection prevalence of chlamydia trachomatis at Oropharynx & Rectum |
| 1.16 | 0.94 | 1.46 | %, Infection prevalence of chlamydia trachomatis at Urethra & Rectum |
| 0.20 | 0.09 | 0.30 | %, Infection prevalence of chlamydia trachomatis at Oropharynx & Urethra & Rectum |

Model output parameters (Published validation dataset1): Data from 1,011 asymptomatic MSM attending Melbourne Sexual Health Centre

**Table S8.4**. Model 1’ output parameters with 95% confidence intervals

| **Mean** | **95%CI: lower** | **95%CI: upper** | **Parameters** |
| --- | --- | --- | --- |
| 45.19 | 35.36 | 58.18 | %, Consistent condom usage in anal sex in past 12 months |
| 87.83 | 81.03 | 94.07 | %, Condom efficacy in preventing transmission |
| - | - | - | Times, Frequency of kissing in the past 12 months |
| 12.68 | 2.55 | 26.07 | Times, Frequency of oral sex in the past 12 months |
| - | - | - | Times, Frequency of rimming in the past 12 months |
| 15.45 | 1.45 | 39.80 | Times, Frequency of anal sex in the past 12 months |
| 91.57 | 31.22 | 196.45 | weeks, Infection duration of chlamydia trachomatis at throat (asymptomatic infection) |
| 1.55 | 1.11 | 1.92 | weeks, Infection duration at urethral (symptomatic infection) |
| 85.65 | 80.77 | 89.60 | %, proportion of urethral infections that are asymptomatic |
| 83.76 | 62.96 | 94.87 | %, proportion of anal infections that are asymptomatic |
| 24.84 | 5.46 | 42.87 | weeks, Infection duration at urethral (asymptomatic infection) |
| 50.33 | 48.21 | 51.60 | weeks, Infection duration of chlamydia trachomatis at anus (asymptomatic infection) |
| 1.49 | 1.09 | 1.90 | weeks, Infection duration of chlamydia trachomatis at anus (symptomatic infection) |
| 78.13 | 66.06 | 92.67 | %, proportion of MSM received throat swab in the past 12 months |
| 79.43 | 66.63 | 92.14 | %, proportion of MSM received anal swab in the past 12 months |
| 79.37 | 65.38 | 92.75 | %, proportion of MSM received urine test in the past 12 months |
| - | - | - | %, proportion of 'oral sex and anal sex' in the same sex episode |
| - | - | - | %, proportion of 'oral sex and rimming' in the same sex episode |
| - | - | - | %, proportion of saliva use during anal sex |
| - | - | - | %, proportion of receptive oral sex followed by partner's insertive anal sex |
| - | - | - | %, proportion of insertive oral sex followed by partner's insertive rimming |
| 1.25 | 0.36 | 2.39 | %, Infection prevalence of chlamydia trachomatis only at Oropharynx |
| 1.62 | 0.85 | 2.31 | %, Infection prevalence of chlamydia trachomatis only at Urethral |
| 7.45 | 5.92 | 8.93 | %, Infection prevalence of chlamydia trachomatis only at Rectum |
| 0.00 | 0.00 | 0.00 | %, Infection prevalence of chlamydia trachomatis at Oropharynx & Urethra |
| 2.36 | 0.97 | 3.95 | %, Infection prevalence of chlamydia trachomatis at Oropharynx & Rectum |
| 0.62 | 0.19 | 1.10 | %, Infection prevalence of chlamydia trachomatis at Urethra & Rectum |
| 0.00 | 0.00 | 0.00 | %, Infection prevalence of chlamydia trachomatis at Oropharynx & Urethra & Rectum |

**Table S8.5**. Model 2’ output parameters with 95% confidence intervals

| **Mean** | **95%CI: lower** | **95%CI: upper** | **Parameters** |
| --- | --- | --- | --- |
| 46.44 | 35.20 | 56.27 | %, Consistent condom usage in anal sex in past 12 months |
| 88.68 | 80.42 | 94.51 | %, Condom efficacy in preventing transmission |
| - | - | - | Times, Frequency of kissing in the past 12 months |
| 17.36 | 5.55 | 27.42 | Times, Frequency of oral sex in the past 12 months |
| 36.93 | 1.70 | 77.84 | Times, Frequency of rimming in the past 12 months |
| 20.02 | 5.12 | 47.66 | Times, Frequency of anal sex in the past 12 months |
| 146.01 | 35.86 | 235.71 | weeks, Infection duration of chlamydia trachomatis at throat(asymptomatic infection) |
| 1.59 | 1.09 | 1.91 | weeks, Infection duration at urethral (symptomatic infection) |
| 84.02 | 81.26 | 88.83 | %, proportion of urethral infections that are asymptomatic |
| 81.25 | 63.24 | 96.84 | %, proportion of anal infections that are asymptomatic |
| 24.12 | 7.11 | 45.96 | weeks, Infection duration at urethral (asymptomatic infection) |
| 49.85 | 48.16 | 51.77 | weeks, Infection duration of chlamydia trachomatis at anus (asymptomatic infection) |
| 1.60 | 1.13 | 1.95 | weeks, Infection duration of chlamydia trachomatis at anus (symptomatic infection) |
| 84.97 | 68.72 | 93.50 | %, proportion of MSM received throat swab in the past 12 months |
| 78.46 | 64.79 | 93.84 | %, proportion of MSM received anal swab in the past 12 months |
| 77.92 | 65.79 | 90.93 | %, proportion of MSM received urine test in the past 12 months |
| - | - | - | %, proportion of 'oral sex and anal sex' in the same sex episode |
| - | - | - | %, proportion of 'oral sex and rimming' in the same sex episode |
| - | - | - | %, proportion of saliva use during anal sex |
| - | - | - | %, proportion of receptive oral sex followed by partner's insertive anal sex |
| - | - | - | %, proportion of insertive oral sex followed by partner's insertive rimming |
| 1.67 | 0.36 | 2.51 | %, Infection prevalence of chlamydia trachomatis only at Oropharynx |
| 1.48 | 0.76 | 2.25 | %, Infection prevalence of chlamydia trachomatis only at Urethral |
| 6.99 | 5.76 | 8.67 | %, Infection prevalence of chlamydia trachomatis only at Rectum |
| 0.00 | 0.00 | 0.00 | %, Infection prevalence of chlamydia trachomatis at Oropharynx & Urethra |
| 2.24 | 1.12 | 3.69 | %, Infection prevalence of chlamydia trachomatis at Oropharynx & Rectum |
| 0.66 | 0.19 | 1.12 | %, Infection prevalence of chlamydia trachomatis at Urethra & Rectum |
| 0.00 | 0.00 | 0.00 | %, Infection prevalence of chlamydia trachomatis at Oropharynx & Urethra & Rectum |

**Table S8.6.** Model 3’ output parameters with 95% confidence intervals

| **Mean** | **95%CI: lower** | **95%CI: upper** | **Parameters** |
| --- | --- | --- | --- |
| 43.82 | 36.01 | 58.44 | %, Consistent condom usage in anal sex in past 12 months |
| 89.33 | 80.48 | 94.68 | %, Condom efficacy in preventing transmission |
| 7.04 | 1.54 | 11.63 | Times, Frequency of kissing in the past 12 months |
| 16.66 | 1.77 | 27.78 | Times, Frequency of oral sex in the past 12 months |
| 40.35 | 1.86 | 77.20 | Times, Frequency of rimming in the past 12 months |
| 30.07 | 2.70 | 51.56 | Times, Frequency of anal sex in the past 12 months |
| 130.69 | 46.76 | 238.73 | weeks, Infection duration of chlamydia trachomatis at throat(asymptomatic infection) |
| 1.45 | 1.09 | 1.95 | weeks, Infection duration at urethral (symptomatic infection) |
| 100.00 | 100.00 | 100.00 | %, proportion of urethral infections that are asymptomatic |
| 100.00 | 100.00 | 100.00 | %, proportion of anal infections that are asymptomatic |
| 25.16 | 4.76 | 46.03 | weeks, Infection duration at urethral (asymptomatic infection) |
| 49.94 | 48.06 | 51.70 | weeks, Infection duration of chlamydia trachomatis at anus (asymptomatic infection) |
| 1.44 | 1.12 | 1.97 | weeks, Infection duration of chlamydia trachomatis at anus (symptomatic infection) |
| 80.84 | 67.45 | 94.21 | %, proportion of MSM received throat swab in the past 12 months |
| 82.23 | 63.90 | 94.90 | %, proportion of MSM received anal swab in the past 12 months |
| 76.88 | 66.13 | 92.76 | %, proportion of MSM received urine test in the past 12 months |
| - | - | - | %, proportion of 'oral sex and anal sex' in the same sex episode |
| - | - | - | %, proportion of 'oral sex and rimming' in the same sex episode |
| - | - | - | %, proportion of saliva use during anal sex |
| - | - | - | %, proportion of receptive oral sex followed by partner's insertive anal sex |
| - | - | - | %, proportion of insertive oral sex followed by partner's insertive rimming |
| 1.39 | 0.43 | 2.45 | %, Infection prevalence of chlamydia trachomatis only at Oropharynx |
| 1.59 | 0.90 | 2.29 | %, Infection prevalence of chlamydia trachomatis only at Urethral |
| 6.98 | 5.78 | 8.61 | %, Infection prevalence of chlamydia trachomatis only at Rectum |
| 0.00 | 0.00 | 0.00 | %, Infection prevalence of chlamydia trachomatis at Oropharynx & Urethra |
| 2.09 | 1.18 | 3.90 | %, Infection prevalence of chlamydia trachomatis at Oropharynx & Rectum |
| 0.52 | 0.20 | 1.09 | %, Infection prevalence of chlamydia trachomatis at Urethra & Rectum |
| 0.00 | 0.00 | 0.00 | %, Infection prevalence of chlamydia trachomatis at Oropharynx & Urethra & Rectum |

Model output parameters (Published validation dataset 2): Data from 393 MSM attending STD & HIV care clinics in the USA

**Table S8.7.** Model 1’ output parameters with 95% confidence intervals

| **Mean** | **95%CI: lower** | **95%CI: upper** | **Parameters** |
| --- | --- | --- | --- |
| 44.20 | 36.01 | 58.69 | %, Consistent condom usage in anal sex in past 12 months |
| 87.91 | 80.33 | 94.74 | %, Condom efficacy in preventing transmission |
| - | - | - | Times, Frequency of kissing in the past 12 months |
| 15.37 | 3.78 | 27.62 | Times, Frequency of oral sex in the past 12 months |
| - | - | - | Times, Frequency of rimming in the past 12 months |
| 19.61 | 1.83 | 37.70 | Times, Frequency of anal sex in the past 12 months |
| 95.55 | 33.70 | 231.51 | weeks, Infection duration of chlamydia trachomatis at throat(asymptomatic infection) |
| 1.49 | 1.20 | 1.96 | weeks, Infection duration at urethral (symptomatic infection) |
| 85.41 | 80.51 | 89.60 | %, proportion of urethral infections that are asymptomatic |
| 80.93 | 66.09 | 96.05 | %, proportion of anal infections that are asymptomatic |
| 24.92 | 4.29 | 49.37 | weeks, Infection duration at urethral (asymptomatic infection) |
| 49.77 | 48.18 | 51.77 | weeks, Infection duration of chlamydia trachomatis at anus (asymptomatic infection) |
| 1.47 | 1.08 | 1.92 | weeks, Infection duration of chlamydia trachomatis at anus (symptomatic infection) |
| 79.88 | 64.58 | 94.29 | %, proportion of MSM received throat swab in the past 12 months |
| 78.86 | 65.50 | 92.13 | %, proportion of MSM received anal swab in the past 12 months |
| 76.76 | 66.18 | 93.14 | %, proportion of MSM received urine test in the past 12 months |
| - | - | - | %, proportion of 'oral sex and anal sex' in the same sex episode |
| - | - | - | %, proportion of 'oral sex and rimming' in the same sex episode |
| - | - | - | %, proportion of saliva use during anal sex |
| - | - | - | %, proportion of receptive oral sex followed by partner's insertive anal sex |
| - | - | - | %, proportion of insertive oral sex followed by partner's insertive rimming |
| 1.13 | 0.20 | 1.94 | %, Infection prevalence of chlamydia trachomatis only at Oropharynx |
| 2.96 | 1.31 | 4.28 | %, Infection prevalence of chlamydia trachomatis only at Urethral |
| 5.77 | 4.31 | 8.61 | %, Infection prevalence of chlamydia trachomatis only at Rectum |
| 0.00 | 0.00 | 0.00 | %, Infection prevalence of chlamydia trachomatis at Oropharynx & Urethra |
| 1.82 | 0.89 | 3.39 | %, Infection prevalence of chlamydia trachomatis at Oropharynx & Rectum |
| 1.23 | 0.32 | 2.19 | %, Infection prevalence of chlamydia trachomatis at Urethra & Rectum |
| 0.00 | 0.00 | 0.00 | %, Infection prevalence of chlamydia trachomatis at Oropharynx & Urethra & Rectum |

**TableS9. 8.** Model 2’ output parameters with 95% confidence intervals

| **Mean** | **95%CI: lower** | **95%CI: upper** | **Parameters** |
| --- | --- | --- | --- |
| 44.71 | 36.92 | 57.55 | %, Consistent condom usage in anal sex in past 12 months |
| 88.49 | 81.87 | 93.32 | %, Condom efficacy in preventing transmission |
| - | - | - | Times, Frequency of kissing in the past 12 months |
| 11.31 | 1.88 | 25.31 | Times, Frequency of oral sex in the past 12 months |
| 45.50 | 9.10 | 78.05 | Times, Frequency of rimming in the past 12 months |
| 15.34 | 1.63 | 48.13 | Times, Frequency of anal sex in the past 12 months |
| 132.30 | 31.94 | 219.48 | weeks, Infection duration of chlamydia trachomatis at throat(asymptomatic infection) |
| 1.51 | 1.09 | 1.92 | weeks, Infection duration at urethral (symptomatic infection) |
| 85.06 | 81.84 | 89.91 | %, proportion of urethral infections that are asymptomatic |
| 75.53 | 65.81 | 96.28 | %, proportion of anal infections that are asymptomatic |
| 34.35 | 7.19 | 51.79 | weeks, Infection duration at urethral (asymptomatic infection) |
| 50.10 | 48.16 | 51.92 | weeks, Infection duration of chlamydia trachomatis at anus (asymptomatic infection) |
| 1.44 | 1.06 | 1.94 | weeks, Infection duration of chlamydia trachomatis at anus (symptomatic infection) |
| 82.39 | 64.74 | 95.06 | %, proportion of MSM received throat swab in the past 12 months |
| 79.20 | 66.13 | 92.28 | %, proportion of MSM received anal swab in the past 12 months |
| 76.43 | 65.79 | 95.09 | %, proportion of MSM received urine test in the past 12 months |
| - | - | - | %, proportion of 'oral sex and anal sex' in the same sex episode |
| - | - | - | %, proportion of 'oral sex and rimming' in the same sex episode |
| - | - | - | %, proportion of saliva use during anal sex |
| - | - | - | %, proportion of receptive oral sex followed by partner's insertive anal sex |
| - | - | - | %, proportion of insertive oral sex followed by partner's insertive rimming |
| 1.08 | 0.29 | 1.84 | %, Infection prevalence of chlamydia trachomatis only at Oropharynx |
| 2.80 | 1.23 | 4.25 | %, Infection prevalence of chlamydia trachomatis only at Urethral |
| 6.94 | 4.83 | 8.85 | %, Infection prevalence of chlamydia trachomatis only at Rectum |
| 0.00 | 0.00 | 0.00 | %, Infection prevalence of chlamydia trachomatis at Oropharynx & Urethra |
| 1.92 | 0.70 | 3.28 | %, Infection prevalence of chlamydia trachomatis at Oropharynx & Rectum |
| 1.13 | 0.39 | 2.22 | %, Infection prevalence of chlamydia trachomatis at Urethra & Rectum |
| 0.00 | 0.00 | 0.00 | %, Infection prevalence of chlamydia trachomatis at Oropharynx & Urethra & Rectum |

**Table S8.9.** Model 3’ output parameters with 95% confidence intervals

| **Mean** | **95%CI: lower** | **95%CI: upper** | **Parameters** |
| --- | --- | --- | --- |
| 48.15 | 37.95 | 58.12 | %, Consistent condom usage in anal sex in past 12 months |
| 87.99 | 81.65 | 92.86 | %, Condom efficacy in preventing transmission |
| 5.82 | 1.10 | 12.47 | Times, Frequency of kissing in the past 12 months |
| 10.29 | 0.73 | 25.63 | Times, Frequency of oral sex in the past 12 months |
| 44.07 | 10.17 | 76.62 | Times, Frequency of rimming in the past 12 months |
| 16.73 | 1.84 | 42.71 | Times, Frequency of anal sex in the past 12 months |
| 166.22 | 31.94 | 237.92 | weeks, Infection duration of chlamydia trachomatis at throat(asymptomatic infection) |
| 1.46 | 1.04 | 1.95 | weeks, Infection duration at urethral (symptomatic infection) |
| 84.97 | 81.29 | 89.64 | %, proportion of urethral infections that are asymptomatic |
| 77.42 | 64.50 | 96.74 | %, proportion of anal infections that are asymptomatic |
| 32.67 | 5.66 | 51.79 | weeks, Infection duration at urethral (asymptomatic infection) |
| 49.55 | 48.22 | 51.86 | weeks, Infection duration of chlamydia trachomatis at anus (asymptomatic infection) |
| 1.53 | 1.16 | 1.94 | weeks, Infection duration of chlamydia trachomatis at anus (symptomatic infection) |
| 80.40 | 64.69 | 95.06 | %, proportion of MSM received throat swab in the past 12 months |
| 78.69 | 65.21 | 92.03 | %, proportion of MSM received anal swab in the past 12 months |
| 78.62 | 64.94 | 94.90 | %, proportion of MSM received urine test in the past 12 months |
| - | - | - | %, proportion of 'oral sex and anal sex' in the same sex episode |
| - | - | - | %, proportion of 'oral sex and rimming' in the same sex episode |
| - | - | - | %, proportion of saliva use during anal sex |
| - | - | - | %, proportion of receptive oral sex followed by partner's insertive anal sex |
| - | - | - | %, proportion of insertive oral sex followed by partner's insertive rimming |
| 0.76 | 0.14 | 1.96 | %, Infection prevalence of chlamydia trachomatis only at Oropharynx |
| 2.25 | 1.20 | 4.25 | %, Infection prevalence of chlamydia trachomatis only at Urethral |
| 6.91 | 4.44 | 9.11 | %, Infection prevalence of chlamydia trachomatis only at Rectum |
| 0.00 | 0.00 | 0.00 | %, Infection prevalence of chlamydia trachomatis at Oropharynx & Urethra |
| 2.03 | 0.76 | 3.31 | %, Infection prevalence of chlamydia trachomatis at Oropharynx & Rectum |
| 1.33 | 0.36 | 2.27 | %, Infection prevalence of chlamydia trachomatis at Urethra & Rectum |
| 0.00 | 0.00 | 0.00 | %, Infection prevalence of chlamydia trachomatis at Oropharynx & Urethra & Rectum |

Model output parameters (Published validation dataset3): Data from MSM surveillance data of all Dutch STI clinics

**Table S8.10**. Model 1’ output parameters with 95% confidence intervals

| **Mean** | **95%CI: lower** | **95%CI: upper** | **Parameters** |
| --- | --- | --- | --- |
| 47.74 | 35.41 | 56.65 | %, Consistent condom usage in anal sex in past 12 months |
| 85.46 | 80.85 | 92.13 | %, Condom efficacy in preventing transmission |
| - | - | - | Times, Frequency of kissing in the past 12 months |
| 15.54 | 2.66 | 23.96 | Times, Frequency of oral sex in the past 12 months |
| - | - | - | Times, Frequency of rimming in the past 12 months |
| 10.61 | 4.23 | 23.24 | Times, Frequency of anal sex in the past 12 months |
| 146.28 | 39.04 | 232.37 | weeks, Infection duration of chlamydia trachomatis at throat (asymptomatic infection) |
| 1.48 | 1.12 | 1.97 | weeks, Infection duration at urethral (symptomatic infection) |
| 100.00 | 100.00 | 100.00 | %, proportion of urethral infections that are asymptomatic |
| 100.00 | 100.00 | 100.00 | %, proportion of anal infections that are asymptomatic |
| 32.77 | 10.09 | 49.11 | weeks, Infection duration at urethral (asymptomatic infection) |
| 49.78 | 48.24 | 51.76 | weeks, Infection duration of chlamydia trachomatis at anus (asymptomatic infection) |
| 1.32 | 1.03 | 1.93 | weeks, Infection duration of chlamydia trachomatis at anus (symptomatic infection) |
| 78.72 | 65.20 | 94.12 | %, proportion of MSM received throat swab in the past 12 months |
| 80.37 | 67.39 | 90.38 | %, proportion of MSM received anal swab in the past 12 months |
| 75.03 | 65.46 | 91.12 | %, proportion of MSM received urine test in the past 12 months |
| - | - | - | %, proportion of 'oral sex and anal sex' in the same sex episode |
| - | - | - | %, proportion of 'oral sex and rimming' in the same sex episode |
| - | - | - | %, proportion of saliva use during anal sex |
| - | - | - | %, proportion of receptive oral sex followed by partner's insertive anal sex |
| - | - | - | %, proportion of insertive oral sex followed by partner's insertive rimming |
| 0.51 | 0.48 | 0.53 | %, Infection prevalence of chlamydia trachomatis only at Oropharynx |
| 2.29 | 2.24 | 2.36 | %, Infection prevalence of chlamydia trachomatis only at Urethral |
| 6.29 | 6.18 | 6.40 | %, Infection prevalence of chlamydia trachomatis only at Rectum |
| 0.06 | 0.05 | 0.07 | %, Infection prevalence of chlamydia trachomatis at Oropharynx & Urethra |
| 0.53 | 0.51 | 0.56 | %, Infection prevalence of chlamydia trachomatis at Oropharynx & Rectum |
| 1.02 | 0.98 | 1.06 | %, Infection prevalence of chlamydia trachomatis at Urethra & Rectum |
| 0.10 | 0.09 | 0.12 | %, Infection prevalence of chlamydia trachomatis at Oropharynx & Urethra & Rectum |

**Table S8.11.** Model 2’ output parameters with 95% confidence intervals

| **Mean** | **95%CI: lower** | **95%CI: upper** | **Parameters** |
| --- | --- | --- | --- |
| 44.97 | 34.87 | 56.82 | %, Consistent condom usage in anal sex in past 12 months |
| 87.44 | 80.56 | 93.54 | %, Condom efficacy in preventing transmission |
| - | - | - | Times, Frequency of kissing in the past 12 months |
| 10.72 | 1.88 | 26.08 | Times, Frequency of oral sex in the past 12 months |
| 19.24 | 2.69 | 67.13 | Times, Frequency of rimming in the past 12 months |
| 12.82 | 2.12 | 48.88 | Times, Frequency of anal sex in the past 12 months |
| 100.44 | 31.72 | 236.78 | weeks, Infection duration of chlamydia trachomatis at throat (asymptomatic infection) |
| 1.53 | 1.14 | 1.96 | weeks, Infection duration at urethral (symptomatic infection) |
| 100.00 | 100.00 | 100.00 | %, proportion of urethral infections that are asymptomatic |
| 100.00 | 100.00 | 100.00 | %, proportion of anal infections that are asymptomatic |
| 29.41 | 14.12 | 50.45 | weeks, Infection duration at urethral (asymptomatic infection) |
| 50.08 | 48.25 | 51.73 | weeks, Infection duration of chlamydia trachomatis at anus (asymptomatic infection) |
| 1.52 | 1.13 | 1.91 | weeks, Infection duration of chlamydia trachomatis at anus (symptomatic infection) |
| 83.86 | 65.06 | 95.48 | %, proportion of MSM received throat swab in the past 12 months |
| 75.02 | 65.30 | 93.58 | %, proportion of MSM received anal swab in the past 12 months |
| 75.54 | 64.95 | 92.25 | %, proportion of MSM received urine test in the past 12 months |
| - | - | - | %, proportion of 'oral sex and anal sex' in the same sex episode |
| - | - | - | %, proportion of 'oral sex and rimming' in the same sex episode |
| - | - | - | %, proportion of saliva use during anal sex |
| - | - | - | %, proportion of receptive oral sex followed by partner's insertive anal sex |
| - | - | - | %, proportion of insertive oral sex followed by partner's insertive rimming |
| 0.50 | 0.49 | 0.53 | %, Infection prevalence of chlamydia trachomatis only at Oropharynx |
| 2.32 | 2.24 | 2.36 | %, Infection prevalence of chlamydia trachomatis only at Urethral |
| 6.25 | 6.17 | 6.38 | %, Infection prevalence of chlamydia trachomatis only at Rectum |
| 0.06 | 0.05 | 0.07 | %, Infection prevalence of chlamydia trachomatis at Oropharynx & Urethra |
| 0.54 | 0.51 | 0.58 | %, Infection prevalence of chlamydia trachomatis at Oropharynx & Rectum |
| 1.03 | 0.99 | 1.07 | %, Infection prevalence of chlamydia trachomatis at Urethra & Rectum |
| 0.10 | 0.09 | 0.12 | %, Infection prevalence of chlamydia trachomatis at Oropharynx & Urethra & Rectum |

**Table S8.12.** Model 3’ output parameters with 95% confidence intervals

| **Mean** | **95%CI: lower** | **95%CI: upper** | **Parameters** |
| --- | --- | --- | --- |
| 48.95 | 35.37 | 58.33 | %, Consistent condom usage in anal sex in past 12 months |
| 86.49 | 80.19 | 93.86 | %, Condom efficacy in preventing transmission |
| 7.49 | 2.15 | 12.39 | Times, Frequency of kissing in the past 12 months |
| 7.69 | 1.04 | 23.11 | Times, Frequency of oral sex in the past 12 months |
| 17.77 | 1.91 | 75.23 | Times, Frequency of rimming in the past 12 months |
| 13.46 | 0.47 | 50.79 | Times, Frequency of anal sex in the past 12 months |
| 122.89 | 45.61 | 234.07 | weeks, Infection duration of chlamydia trachomatis at throat(asymptomatic infection) |
| 1.53 | 1.16 | 1.96 | weeks, Infection duration at urethral (symptomatic infection) |
| 100.00 | 100.00 | 100.00 | %, proportion of urethral infections that are asymptomatic |
| 100.00 | 100.00 | 100.00 | %, proportion of anal infections that are asymptomatic |
| 31.33 | 14.93 | 49.24 | weeks, Infection duration at urethral (asymptomatic infection) |
| 50.14 | 48.27 | 51.75 | weeks, Infection duration of chlamydia trachomatis at anus (asymptomatic infection) |
| 1.39 | 1.07 | 1.97 | weeks, Infection duration of chlamydia trachomatis at anus (symptomatic infection) |
| 77.57 | 66.36 | 94.77 | %, proportion of MSM received throat swab in the past 12 months |
| 77.07 | 64.78 | 93.65 | %, proportion of MSM received anal swab in the past 12 months |
| 76.06 | 65.04 | 92.27 | %, proportion of MSM received urine test in the past 12 months |
| - | - | - | %, proportion of 'oral sex and anal sex' in the same sex episode |
| - | - | - | %, proportion of 'oral sex and rimming' in the same sex episode |
| - | - | - | %, proportion of saliva use during anal sex |
| - | - | - | %, proportion of receptive oral sex followed by partner's insertive anal sex |
| - | - | - | %, proportion of insertive oral sex followed by partner's insertive rimming |
| 0.51 | 0.48 | 0.54 | %, Infection prevalence of chlamydia trachomatis only at Oropharynx |
| 2.31 | 2.24 | 2.36 | %, Infection prevalence of chlamydia trachomatis only at Urethral |
| 6.32 | 6.18 | 6.39 | %, Infection prevalence of chlamydia trachomatis only at Rectum |
| 0.06 | 0.05 | 0.07 | %, Infection prevalence of chlamydia trachomatis at Oropharynx & Urethra |
| 0.54 | 0.51 | 0.58 | %, Infection prevalence of chlamydia trachomatis at Oropharynx & Rectum |
| 1.03 | 0.98 | 1.06 | %, Infection prevalence of chlamydia trachomatis at Urethra & Rectum |
| 0.11 | 0.09 | 0.12 | %, Infection prevalence of chlamydia trachomatis at Oropharynx & Urethra & Rectum |

Model output parameters (Published validation dataset4): Data from 1,610 community MSM in Thailand

**Table S8.13.** Model 1’ output parameters with 95% confidence intervals

| **Mean** | **95%CI: lower** | **95%CI: upper** | **Parameters** |
| --- | --- | --- | --- |
| 44.48 | 35.57 | 57.26 | %, Consistent condom usage in anal sex in past 12 months |
| 87.86 | 80.59 | 94.38 | %, Condom efficacy in preventing transmission |
| - | - | - | Times, Frequency of kissing in the past 12 months |
| 20.89 | 4.35 | 26.86 | Times, Frequency of oral sex in the past 12 months |
| - | - | - | Times, Frequency of rimming in the past 12 months |
| 13.87 | 1.68 | 42.97 | Times, Frequency of anal sex in the past 12 months |
| 146.28 | 34.04 | 237.39 | weeks, Infection duration of chlamydia trachomatis at throat(asymptomatic infection) |
| 1.33 | 1.03 | 1.91 | weeks, Infection duration at urethral (symptomatic infection) |
| 100.00 | 100.00 | 100.00 | %, proportion of urethral infections that are asymptomatic |
| 100.00 | 100.00 | 100.00 | %, proportion of anal infections that are asymptomatic |
| 29.15 | 4.73 | 50.25 | weeks, Infection duration at urethral (asymptomatic infection) |
| 50.16 | 48.19 | 51.78 | weeks, Infection duration of chlamydia trachomatis at anus (asymptomatic infection) |
| 1.40 | 1.03 | 1.94 | weeks, Infection duration of chlamydia trachomatis at anus (symptomatic infection) |
| 77.06 | 64.34 | 94.19 | %, proportion of MSM received throat swab in the past 12 months |
| 78.12 | 64.83 | 92.36 | %, proportion of MSM received anal swab in the past 12 months |
| 78.98 | 66.34 | 93.64 | %, proportion of MSM received urine test in the past 12 months |
| - | - | - | %, proportion of 'oral sex and anal sex' in the same sex episode |
| - | - | - | %, proportion of 'oral sex and rimming' in the same sex episode |
| - | - | - | %, proportion of saliva use during anal sex |
| - | - | - | %, proportion of receptive oral sex followed by partner's insertive anal sex |
| - | - | - | %, proportion of insertive oral sex followed by partner's insertive rimming |
| 1.70 | 1.25 | 2.41 | %, Infection prevalence of chlamydia trachomatis only at Oropharynx |
| 5.23 | 4.15 | 5.83 | %, Infection prevalence of chlamydia trachomatis only at Urethral |
| 11.89 | 10.44 | 13.37 | %, Infection prevalence of chlamydia trachomatis only at Rectum |
| 0.22 | 0.03 | 0.38 | %, Infection prevalence of chlamydia trachomatis at Oropharynx & Urethra |
| 1.12 | 0.79 | 1.68 | %, Infection prevalence of chlamydia trachomatis at Oropharynx & Rectum |
| 2.17 | 1.66 | 2.68 | %, Infection prevalence of chlamydia trachomatis at Urethra & Rectum |
| 0.21 | 0.03 | 0.38 | %, Infection prevalence of chlamydia trachomatis at Oropharynx & Urethra & Rectum |

**Table S8.14**. Model 2’ output parameters with 95% confidence intervals

| **Mean** | **95%CI: lower** | **95%CI: upper** | **Parameters** |
| --- | --- | --- | --- |
| 46.18 | 36.36 | 55.59 | %, Consistent condom usage in anal sex in past 12 months |
| 87.71 | 81.29 | 93.06 | %, Condom efficacy in preventing transmission |
| - | - | - | Times, Frequency of kissing in the past 12 months |
| 17.81 | 2.29 | 26.93 | Times, Frequency of oral sex in the past 12 months |
| 30.17 | 4.25 | 69.51 | Times, Frequency of rimming in the past 12 months |
| 33.51 | 2.31 | 51.95 | Times, Frequency of anal sex in the past 12 months |
| 146.84 | 33.42 | 238.52 | weeks, Infection duration of chlamydia trachomatis at throat(asymptomatic infection) |
| 1.58 | 1.06 | 1.90 | weeks, Infection duration at urethral (symptomatic infection) |
| 100.00 | 100.00 | 100.00 | %, proportion of urethral infections that are asymptomatic |
| 100.00 | 100.00 | 100.00 | %, proportion of anal infections that are asymptomatic |
| 22.18 | 3.95 | 47.21 | weeks, Infection duration at urethral (asymptomatic infection) |
| 50.33 | 48.21 | 51.71 | weeks, Infection duration of chlamydia trachomatis at anus (asymptomatic infection) |
| 1.44 | 1.15 | 1.95 | weeks, Infection duration of chlamydia trachomatis at anus (symptomatic infection) |
| 81.60 | 67.26 | 95.40 | %, proportion of MSM received throat swab in the past 12 months |
| 80.36 | 64.40 | 94.33 | %, proportion of MSM received anal swab in the past 12 months |
| 78.51 | 65.88 | 93.38 | %, proportion of MSM received urine test in the past 12 months |
| - | - | - | %, proportion of 'oral sex and anal sex' in the same sex episode |
| - | - | - | %, proportion of 'oral sex and rimming' in the same sex episode |
| - | - | - | %, proportion of saliva use during anal sex |
| - | - | - | %, proportion of receptive oral sex followed by partner's insertive anal sex |
| - | - | - | %, proportion of insertive oral sex followed by partner's insertive rimming |
| 1.68 | 1.19 | 2.47 | %, Infection prevalence of chlamydia trachomatis only at Oropharynx |
| 5.18 | 3.94 | 5.90 | %, Infection prevalence of chlamydia trachomatis only at Urethral |
| 11.90 | 10.44 | 12.88 | %, Infection prevalence of chlamydia trachomatis only at Rectum |
| 0.21 | 0.03 | 0.37 | %, Infection prevalence of chlamydia trachomatis at Oropharynx & Urethra |
| 1.31 | 0.75 | 1.64 | %, Infection prevalence of chlamydia trachomatis at Oropharynx & Rectum |
| 2.09 | 1.55 | 2.79 | %, Infection prevalence of chlamydia trachomatis at Urethra & Rectum |
| 0.22 | 0.05 | 0.38 | %, Infection prevalence of chlamydia trachomatis at Oropharynx & Urethra & Rectum |

**Table S8.15**. Model 3’ output parameters with 95% confidence intervals

| **Mean** | **95%CI: lower** | **95%CI: upper** | **Parameters** |
| --- | --- | --- | --- |
| 48.71 | 36.37 | 58.50 | %, Consistent condom usage in anal sex in past 12 months |
| 88.76 | 80.42 | 93.61 | %, Condom efficacy in preventing transmission |
| 6.21 | 2.51 | 12.65 | Times, Frequency of kissing in the past 12 months |
| 10.18 | 1.63 | 27.70 | Times, Frequency of oral sex in the past 12 months |
| 36.43 | 2.50 | 73.10 | Times, Frequency of rimming in the past 12 months |
| 29.01 | 3.52 | 52.91 | Times, Frequency of anal sex in the past 12 months |
| 122.43 | 30.00 | 235.88 | weeks, Infection duration of chlamydia trachomatis at throat(asymptomatic infection) |
| 1.49 | 1.03 | 1.96 | weeks, Infection duration at urethral (symptomatic infection) |
| 100.00 | 100.00 | 100.00 | %, proportion of urethral infections that are asymptomatic |
| 100.00 | 100.00 | 100.00 | %, proportion of anal infections that are asymptomatic |
| 26.38 | 8.34 | 51.11 | weeks, Infection duration at urethral (asymptomatic infection) |
| 49.99 | 48.16 | 51.53 | weeks, Infection duration of chlamydia trachomatis at anus (asymptomatic infection) |
| 1.50 | 1.03 | 1.93 | weeks, Infection duration of chlamydia trachomatis at anus (symptomatic infection) |
| 78.27 | 66.85 | 94.51 | %, proportion of MSM received throat swab in the past 12 months |
| 82.84 | 64.67 | 94.93 | %, proportion of MSM received anal swab in the past 12 months |
| 80.26 | 65.38 | 90.25 | %, proportion of MSM received urine test in the past 12 months |
| - | - | - | %, proportion of 'oral sex and anal sex' in the same sex episode |
| - | - | - | %, proportion of 'oral sex and rimming' in the same sex episode |
| - | - | - | %, proportion of saliva use during anal sex |
| - | - | - | %, proportion of receptive oral sex followed by partner's insertive anal sex |
| - | - | - | %, proportion of insertive oral sex followed by partner's insertive rimming |
| 1.98 | 1.47 | 2.49 | %, Infection prevalence of chlamydia trachomatis only at Oropharynx |
| 4.65 | 3.91 | 5.89 | %, Infection prevalence of chlamydia trachomatis only at Urethral |
| 11.88 | 10.43 | 13.11 | %, Infection prevalence of chlamydia trachomatis only at Rectum |
| 0.23 | 0.05 | 0.38 | %, Infection prevalence of chlamydia trachomatis at Oropharynx & Urethra |
| 1.07 | 0.72 | 1.74 | %, Infection prevalence of chlamydia trachomatis at Oropharynx & Rectum |
| 1.92 | 1.45 | 2.64 | %, Infection prevalence of chlamydia trachomatis at Urethra & Rectum |
| 0.17 | 0.01 | 0.38 | %, Infection prevalence of chlamydia trachomatis at Oropharynx & Urethra & Rectum |

Model output parameters (Published validation dataset5): Data from 179 MSM with HIV in the USA

**Table S8.16.** Model 1’ output parameters with 95% confidence intervals

| **Mean** | **95%CI: lower** | **95%CI: upper** | **Parameters** |
| --- | --- | --- | --- |
| 47.10 | 35.21 | 58.70 | %, Consistent condom usage in anal sex in past 12 months |
| 87.06 | 80.62 | 93.07 | %, Condom efficacy in preventing transmission |
| - | - | - | Times, Frequency of kissing in the past 12 months |
| 14.76 | 2.51 | 24.57 | Times, Frequency of oral sex in the past 12 months |
| - | - | - | Times, Frequency of rimming in the past 12 months |
| 12.02 | 1.86 | 33.60 | Times, Frequency of anal sex in the past 12 months |
| 115.06 | 27.90 | 227.64 | weeks, Infection duration of chlamydia trachomatis at throat (asymptomatic infection) |
| 1.47 | 1.08 | 1.97 | weeks, Infection duration at urethral (symptomatic infection) |
| 83.63 | 80.47 | 89.65 | %, proportion of urethral infections that are asymptomatic |
| 84.25 | 65.24 | 95.23 | %, proportion of anal infections that are asymptomatic |
| 33.29 | 10.32 | 51.10 | weeks, Infection duration at urethral (asymptomatic infection) |
| 49.67 | 48.26 | 51.38 | weeks, Infection duration of chlamydia trachomatis at anus (asymptomatic infection) |
| 1.59 | 1.26 | 1.99 | weeks, Infection duration of chlamydia trachomatis at anus (symptomatic infection) |
| 84.21 | 64.83 | 94.99 | %, proportion of MSM received throat swab in the past 12 months |
| 77.29 | 64.94 | 93.19 | %, proportion of MSM received anal swab in the past 12 months |
| 84.79 | 65.21 | 92.77 | %, proportion of MSM received urine test in the past 12 months |
| - | - | - | %, proportion of 'oral sex and anal sex' in the same sex episode |
| - | - | - | %, proportion of 'oral sex and rimming' in the same sex episode |
| - | - | - | %, proportion of saliva use during anal sex |
| - | - | - | %, proportion of receptive oral sex followed by partner's insertive anal sex |
| - | - | - | %, proportion of insertive oral sex followed by partner's insertive rimming |
| 0.00 | 0.00 | 0.00 | %, Infection prevalence of chlamydia trachomatis only at Oropharynx |
| 2.43 | 0.84 | 4.33 | %, Infection prevalence of chlamydia trachomatis only at Urethral |
| 15.21 | 8.81 | 17.84 | %, Infection prevalence of chlamydia trachomatis only at Rectum |
| 0.00 | 0.00 | 0.00 | %, Infection prevalence of chlamydia trachomatis at Oropharynx & Urethra |
| 0.00 | 0.00 | 0.00 | %, Infection prevalence of chlamydia trachomatis at Oropharynx & Rectum |
| 2.83 | 0.76 | 4.17 | %, Infection prevalence of chlamydia trachomatis at Urethra & Rectum |
| 0.00 | 0.00 | 0.00 | %, Infection prevalence of chlamydia trachomatis at Oropharynx & Urethra & Rectum |

**Table S8.17**. Model 2’ output parameters with 95% confidence intervals

| **Mean** | **95%CI: lower** | **95%CI: upper** | **Parameters** |
| --- | --- | --- | --- |
| 44.46 | 35.06 | 56.26 | %, Consistent condom usage in anal sex in past 12 months |
| 86.07 | 80.48 | 93.98 | %, Condom efficacy in preventing transmission |
| - | - | - | Times, Frequency of kissing in the past 12 months |
| 10.95 | 2.66 | 27.30 | Times, Frequency of oral sex in the past 12 months |
| 29.17 | 2.56 | 76.63 | Times, Frequency of rimming in the past 12 months |
| 13.73 | 1.72 | 48.68 | Times, Frequency of anal sex in the past 12 months |
| 125.37 | 34.04 | 232.11 | weeks, Infection duration of chlamydia trachomatis at throat (asymptomatic infection) |
| 1.37 | 1.05 | 1.97 | weeks, Infection duration at urethral (symptomatic infection) |
| 84.05 | 80.31 | 89.60 | %, proportion of urethral infections that are asymptomatic |
| 83.31 | 67.07 | 94.44 | %, proportion of anal infections that are asymptomatic |
| 27.95 | 4.02 | 51.02 | weeks, Infection duration at urethral (asymptomatic infection) |
| 49.80 | 48.43 | 51.67 | weeks, Infection duration of chlamydia trachomatis at anus (asymptomatic infection) |
| 1.38 | 1.07 | 1.84 | weeks, Infection duration of chlamydia trachomatis at anus (symptomatic infection) |
| 83.28 | 64.94 | 95.31 | %, proportion of MSM received throat swab in the past 12 months |
| 79.54 | 67.32 | 94.19 | %, proportion of MSM received anal swab in the past 12 months |
| 79.23 | 64.86 | 92.58 | %, proportion of MSM received urine test in the past 12 months |
| - | - | - | %, proportion of 'oral sex and anal sex' in the same sex episode |
| - | - | - | %, proportion of 'oral sex and rimming' in the same sex episode |
| - | - | - | %, proportion of saliva use during anal sex |
| - | - | - | %, proportion of receptive oral sex followed by partner's insertive anal sex |
| - | - | - | %, proportion of insertive oral sex followed by partner's insertive rimming |
| 0.00 | 0.00 | 0.00 | %, Infection prevalence of chlamydia trachomatis only at Oropharynx |
| 2.97 | 0.66 | 4.77 | %, Infection prevalence of chlamydia trachomatis only at Urethral |
| 13.21 | 9.17 | 18.54 | %, Infection prevalence of chlamydia trachomatis only at Rectum |
| 0.00 | 0.00 | 0.00 | %, Infection prevalence of chlamydia trachomatis at Oropharynx & Urethra |
| 0.00 | 0.00 | 0.00 | %, Infection prevalence of chlamydia trachomatis at Oropharynx & Rectum |
| 2.36 | 0.82 | 4.09 | %, Infection prevalence of chlamydia trachomatis at Urethra & Rectum |
| 0.00 | 0.00 | 0.00 | %, Infection prevalence of chlamydia trachomatis at Oropharynx & Urethra & Rectum |

**Table S8.18.** Model 3’ output parameters with 95% confidence intervals

| **Mean** | **95%CI: lower** | **95%CI: upper** | **Parameters** |
| --- | --- | --- | --- |
| 44.72 | 34.86 | 57.44 | %, Consistent condom usage in anal sex in past 12 months |
| 85.81 | 80.51 | 94.01 | %, Condom efficacy in preventing transmission |
| 6.16 | 0.81 | 12.43 | Times, Frequency of kissing in the past 12 months |
| 14.21 | 1.30 | 27.72 | Times, Frequency of oral sex in the past 12 months |
| 29.43 | 6.87 | 76.83 | Times, Frequency of rimming in the past 12 months |
| 11.17 | 2.57 | 50.14 | Times, Frequency of anal sex in the past 12 months |
| 103.34 | 28.69 | 224.19 | weeks, Infection duration of chlamydia trachomatis at throat(asymptomatic infection) |
| 1.50 | 1.11 | 1.97 | weeks, Infection duration at urethral (symptomatic infection) |
| 84.67 | 80.42 | 88.74 | %, proportion of urethral infections that are asymptomatic |
| 83.16 | 64.12 | 96.40 | %, proportion of anal infections that are asymptomatic |
| 32.33 | 4.80 | 50.09 | weeks, Infection duration at urethral (asymptomatic infection) |
| 50.10 | 48.13 | 51.70 | weeks, Infection duration of chlamydia trachomatis at anus (asymptomatic infection) |
| 1.52 | 1.05 | 1.97 | weeks, Infection duration of chlamydia trachomatis at anus (symptomatic infection) |
| 84.16 | 67.99 | 94.41 | %, proportion of MSM received throat swab in the past 12 months |
| 77.88 | 65.87 | 91.62 | %, proportion of MSM received anal swab in the past 12 months |
| 77.49 | 64.87 | 93.20 | %, proportion of MSM received urine test in the past 12 months |
| - | - | - | %, proportion of 'oral sex and anal sex' in the same sex episode |
| - | - | - | %, proportion of 'oral sex and rimming' in the same sex episode |
| - | - | - | %, proportion of saliva use during anal sex |
| - | - | - | %, proportion of receptive oral sex followed by partner's insertive anal sex |
| - | - | - | %, proportion of insertive oral sex followed by partner's insertive rimming |
| 0.00 | 0.00 | 0.00 | %, Infection prevalence of chlamydia trachomatis only at Oropharynx |
| 2.83 | 0.58 | 4.84 | %, Infection prevalence of chlamydia trachomatis only at Urethral |
| 12.52 | 8.73 | 17.74 | %, Infection prevalence of chlamydia trachomatis only at Rectum |
| 0.00 | 0.00 | 0.00 | %, Infection prevalence of chlamydia trachomatis at Oropharynx & Urethra |
| 0.00 | 0.00 | 0.00 | %, Infection prevalence of chlamydia trachomatis at Oropharynx & Rectum |
| 2.41 | 0.72 | 4.37 | %, Infection prevalence of chlamydia trachomatis at Urethra & Rectum |
| 0.00 | 0.00 | 0.00 | %, Infection prevalence of chlamydia trachomatis at Oropharynx & Urethra & Rectum |

‘Anal sex, oral sex and sequential sexual practices’ transmission models (Model 1, 4-6)

Model output parameters (Calibration Dataset): Unpublished calibration data from 4888 MSM attending Melbourne Sexual Health Centre

**Table S8.19.** Model 1’ output parameters with 95% confidence intervals

| **Mean** | **95%CI: lower** | **95%CI: upper** | **Parameters** |
| --- | --- | --- | --- |
| 43.91 | 35.89 | 56.88 | %, Consistent condom usage in anal sex in past 12 months |
| 88.12 | 81.32 | 94.44 | %, Condom efficacy in preventing transmission |
| - | - | - | Times, Frequency of kissing in the past 12 months |
| 16.49 | 5.43 | 27.52 | Times, Frequency of oral sex in the past 12 months |
| - | - | - | Times, Frequency of rimming in the past 12 months |
| 7.19 | 1.63 | 23.03 | Times, Frequency of anal sex in the past 12 months |
| 118.64 | 31.37 | 215.74 | weeks, Infection duration of chlamydia trachomatis at throat (asymptomatic infection) |
| 1.44 | 1.14 | 1.92 | weeks, Infection duration at urethral (symptomatic infection) |
| 85.13 | 80.84 | 88.06 | %, proportion of urethral infections that are asymptomatic |
| 82.40 | 65.34 | 95.55 | %, proportion of anal infections that are asymptomatic |
| 31.23 | 3.91 | 49.83 | weeks, Infection duration at urethral (asymptomatic infection) |
| 49.98 | 48.46 | 51.53 | weeks, Infection duration of chlamydia trachomatis at anus (asymptomatic infection) |
| 1.51 | 1.10 | 1.89 | weeks, Infection duration of chlamydia trachomatis at anus (symptomatic infection) |
| 76.52 | 66.42 | 92.98 | %, proportion of MSM received throat swab in the past 12 months |
| 82.80 | 66.38 | 91.53 | %, proportion of MSM received anal swab in the past 12 months |
| 80.09 | 67.22 | 93.58 | %, proportion of MSM received urine test in the past 12 months |
| - | - | - | %, proportion of 'oral sex and anal sex' in the same sex episode |
| - | - | - | %, proportion of 'oral sex and rimming' in the same sex episode |
| - | - | - | %, proportion of saliva use during anal sex |
| - | - | - | %, proportion of receptive oral sex followed by partner's insertive anal sex |
| - | - | - | %, proportion of insertive oral sex followed by partner's insertive rimming |
| 0.71 | 0.57 | 0.97 | %, Infection prevalence of chlamydia trachomatis only at Oropharynx |
| 1.84 | 1.57 | 2.16 | %, Infection prevalence of chlamydia trachomatis only at Urethral |
| 7.35 | 6.88 | 8.06 | %, Infection prevalence of chlamydia trachomatis only at Rectum |
| 0.00 | 0.00 | 0.00 | %, Infection prevalence of chlamydia trachomatis at Oropharynx & Urethra |
| 1.26 | 0.97 | 1.51 | %, Infection prevalence of chlamydia trachomatis at Oropharynx & Rectum |
| 1.21 | 0.99 | 1.47 | %, Infection prevalence of chlamydia trachomatis at Urethra & Rectum |
| 0.20 | 0.11 | 0.31 | %, Infection prevalence of chlamydia trachomatis at Oropharynx & Urethra & Rectum |

**Table S8.20**. Model 4’ output parameters with 95% confidence intervals

| **Mean** | **95%CI: lower** | **95%CI: upper** | **Parameters** |
| --- | --- | --- | --- |
| 46.24 | 36.48 | 54.87 | %, Consistent condom usage in anal sex in past 12 months |
| 87.30 | 81.87 | 92.51 | %, Condom efficacy in preventing transmission |
| - | - | - | Times, Frequency of kissing in the past 12 months |
| 16.81 | 7.18 | 26.46 | Times, Frequency of oral sex in the past 12 months |
| - | - | - | Times, Frequency of rimming in the past 12 months |
| 8.18 | 2.22 | 17.62 | Times, Frequency of anal sex in the past 12 months |
| 144.80 | 53.03 | 204.52 | weeks, Infection duration of chlamydia trachomatis at throat (asymptomatic infection) |
| 1.54 | 1.20 | 1.87 | weeks, Infection duration at urethral (symptomatic infection) |
| 85.40 | 81.17 | 87.85 | %, proportion of urethral infections that are asymptomatic |
| 84.91 | 67.42 | 94.63 | %, proportion of anal infections that are asymptomatic |
| 31.10 | 6.23 | 49.27 | weeks, Infection duration at urethral (asymptomatic infection) |
| 50.18 | 48.81 | 51.44 | weeks, Infection duration of chlamydia trachomatis at anus (asymptomatic infection) |
| 1.55 | 1.14 | 1.87 | weeks, Infection duration of chlamydia trachomatis at anus (symptomatic infection) |
| 79.60 | 67.41 | 91.34 | %, proportion of MSM received throat swab in the past 12 months |
| 78.81 | 67.78 | 89.56 | %, proportion of MSM received anal swab in the past 12 months |
| 76.20 | 67.16 | 91.36 | %, proportion of MSM received urine test in the past 12 months |
| 29.91 | 26.06 | 32.82 | %, proportion of 'oral sex and anal sex' in the same sex episode |
| - | - | - | %, proportion of 'oral sex and rimming' in the same sex episode |
| - | - | - | %, proportion of saliva use during anal sex |
| 80.00 | 80.00 | 80.00 | %, proportion of receptive oral sex followed by partner's insertive anal sex |
| - | - | - | %, proportion of insertive oral sex followed by partner's insertive rimming |
| 0.75 | 0.60 | 0.94 | %, Infection prevalence of chlamydia trachomatis only at Oropharynx |
| 1.91 | 1.57 | 2.15 | %, Infection prevalence of chlamydia trachomatis only at Urethral |
| 7.43 | 6.96 | 7.91 | %, Infection prevalence of chlamydia trachomatis only at Rectum |
| 0.00 | 0.00 | 0.00 | %, Infection prevalence of chlamydia trachomatis at Oropharynx & Urethra |
| 1.12 | 0.97 | 1.50 | %, Infection prevalence of chlamydia trachomatis at Oropharynx & Rectum |
| 1.25 | 0.99 | 1.44 | %, Infection prevalence of chlamydia trachomatis at Urethra & Rectum |
| 0.22 | 0.12 | 0.31 | %, Infection prevalence of chlamydia trachomatis at Oropharynx & Urethra & Rectum |

**Table S8.21**. Model 5’ output parameters with 95% confidence intervals

| **Mean** | **95%CI: lower** | **95%CI: upper** | **Parameters** |
| --- | --- | --- | --- |
| 46.04 | 39.80 | 54.34 | %, Consistent condom usage in anal sex in past 12 months |
| 87.27 | 81.90 | 91.96 | %, Condom efficacy in preventing transmission |
| - | - | - | Times, Frequency of kissing in the past 12 months |
| 16.33 | 7.73 | 25.59 | Times, Frequency of oral sex in the past 12 months |
| - | - | - | Times, Frequency of rimming in the past 12 months |
| 11.45 | 4.55 | 17.11 | Times, Frequency of anal sex in the past 12 months |
| 114.71 | 65.89 | 195.94 | weeks, Infection duration of chlamydia trachomatis at throat (asymptomatic infection) |
| 1.60 | 1.34 | 1.85 | weeks, Infection duration at urethral (symptomatic infection) |
| 83.63 | 81.21 | 87.41 | %, proportion of urethral infections that are asymptomatic |
| 80.97 | 68.32 | 93.53 | %, proportion of anal infections that are asymptomatic |
| 26.14 | 7.88 | 47.30 | weeks, Infection duration at urethral (asymptomatic infection) |
| 49.95 | 48.88 | 51.35 | weeks, Infection duration of chlamydia trachomatis at anus (asymptomatic infection) |
| 1.43 | 1.15 | 1.78 | weeks, Infection duration of chlamydia trachomatis at anus (symptomatic infection) |
| 78.98 | 67.74 | 90.41 | %, proportion of MSM received throat swab in the past 12 months |
| 77.22 | 68.44 | 86.62 | %, proportion of MSM received anal swab in the past 12 months |
| 81.02 | 68.13 | 91.10 | %, proportion of MSM received urine test in the past 12 months |
| - | - | - | %, proportion of 'oral sex and anal sex' in the same sex episode |
| - | - | - | %, proportion of 'oral sex and rimming' in the same sex episode |
| 68.47 | 66.85 | 69.62 | %, proportion of saliva use during anal sex |
| - | - | - | %, proportion of receptive oral sex followed by partner's insertive anal sex |
| - | - | - | %, proportion of insertive oral sex followed by partner's insertive rimming |
| 0.75 | 0.62 | 0.93 | %, Infection prevalence of chlamydia trachomatis only at Oropharynx |
| 1.86 | 1.62 | 2.12 | %, Infection prevalence of chlamydia trachomatis only at Urethral |
| 7.49 | 7.01 | 7.91 | %, Infection prevalence of chlamydia trachomatis only at Rectum |
| 0.00 | 0.00 | 0.00 | %, Infection prevalence of chlamydia trachomatis at Oropharynx & Urethra |
| 1.25 | 1.02 | 1.49 | %, Infection prevalence of chlamydia trachomatis at Oropharynx & Rectum |
| 1.21 | 1.02 | 1.37 | %, Infection prevalence of chlamydia trachomatis at Urethra & Rectum |
| 0.20 | 0.12 | 0.28 | %, Infection prevalence of chlamydia trachomatis at Oropharynx & Urethra & Rectum |

**Table S8.22**. Model 6’ output parameters with 95% confidence intervals

| **Mean** | **95%CI: lower** | **95%CI: upper** | **Parameters** |
| --- | --- | --- | --- |
| 46.48 | 41.51 | 52.64 | %, Consistent condom usage in anal sex in past 12 months |
| 87.45 | 83.43 | 91.16 | %, Condom efficacy in preventing transmission |
| - | - | - | Times, Frequency of kissing in the past 12 months |
| 16.36 | 11.33 | 23.98 | Times, Frequency of oral sex in the past 12 months |
| - | - | - | Times, Frequency of rimming in the past 12 months |
| 11.54 | 6.25 | 16.44 | Times, Frequency of anal sex in the past 12 months |
| 119.88 | 84.43 | 175.54 | weeks, Infection duration of chlamydia trachomatis at throat (asymptomatic infection) |
| 1.59 | 1.38 | 1.81 | weeks, Infection duration at urethral (symptomatic infection) |
| 84.14 | 81.76 | 86.69 | %, proportion of urethral infections that are asymptomatic |
| 82.42 | 69.79 | 91.90 | %, proportion of anal infections that are asymptomatic |
| 24.10 | 9.28 | 35.45 | weeks, Infection duration at urethral (asymptomatic infection) |
| 50.48 | 49.34 | 51.08 | weeks, Infection duration of chlamydia trachomatis at anus (asymptomatic infection) |
| 1.51 | 1.23 | 1.73 | weeks, Infection duration of chlamydia trachomatis at anus (symptomatic infection) |
| 78.47 | 69.44 | 85.57 | %, proportion of MSM received throat swab in the past 12 months |
| 78.97 | 70.89 | 85.32 | %, proportion of MSM received anal swab in the past 12 months |
| 77.68 | 68.95 | 90.41 | %, proportion of MSM received urine test in the past 12 months |
| 30.85 | 27.45 | 32.06 | %, proportion of 'oral sex and anal sex' in the same sex episode |
| - | - | - | %, proportion of 'oral sex and rimming' in the same sex episode |
| 68.02 | 67.12 | 69.15 | %, proportion of saliva use during anal sex |
| 80.00 | 80.00 | 80.00 | %, proportion of receptive oral sex followed by partner's insertive anal sex |
| - | - | - | %, proportion of insertive oral sex followed by partner's insertive rimming |
| 0.80 | 0.67 | 0.90 | %, Infection prevalence of chlamydia trachomatis only at Oropharynx |
| 1.86 | 1.67 | 2.10 | %, Infection prevalence of chlamydia trachomatis only at Urethral |
| 7.54 | 7.31 | 7.85 | %, Infection prevalence of chlamydia trachomatis only at Rectum |
| 0.00 | 0.00 | 0.00 | %, Infection prevalence of chlamydia trachomatis at Oropharynx & Urethra |
| 1.23 | 1.05 | 1.42 | %, Infection prevalence of chlamydia trachomatis at Oropharynx & Rectum |
| 1.19 | 1.03 | 1.36 | %, Infection prevalence of chlamydia trachomatis at Urethra & Rectum |
| 0.19 | 0.15 | 0.26 | %, Infection prevalence of chlamydia trachomatis at Oropharynx & Urethra & Rectum |

Model output parameters (Published validation dataset1): Data from 1,011 asymptomatic MSM attending Melbourne Sexual Health Centre

**Table S8.23.** Model 1’ output parameters with 95% confidence intervals

| **Mean** | **95%CI: lower** | **95%CI: upper** | **Parameters** |
| --- | --- | --- | --- |
| 45.19 | 35.36 | 58.18 | %, Consistent condom usage in anal sex in past 12 months |
| 87.83 | 81.03 | 94.07 | %, Condom efficacy in preventing transmission |
| - | - | - | Times, Frequency of kissing in the past 12 months |
| 12.68 | 2.55 | 26.07 | Times, Frequency of oral sex in the past 12 months |
| - | - | - | Times, Frequency of rimming in the past 12 months |
| 15.45 | 1.45 | 39.80 | Times, Frequency of anal sex in the past 12 months |
| 91.57 | 31.22 | 196.45 | weeks, Infection duration of chlamydia trachomatis at throat (asymptomatic infection) |
| 1.55 | 1.11 | 1.92 | weeks, Infection duration at urethral (symptomatic infection) |
| 85.65 | 80.77 | 89.60 | %, proportion of urethral infections that are asymptomatic |
| 83.76 | 62.96 | 94.87 | %, proportion of anal infections that are asymptomatic |
| 24.84 | 5.46 | 42.87 | weeks, Infection duration at urethral (asymptomatic infection) |
| 50.33 | 48.21 | 51.60 | weeks, Infection duration of chlamydia trachomatis at anus (asymptomatic infection) |
| 1.49 | 1.09 | 1.90 | weeks, Infection duration of chlamydia trachomatis at anus (symptomatic infection) |
| 78.13 | 66.06 | 92.67 | %, proportion of MSM received throat swab in the past 12 months |
| 79.43 | 66.63 | 92.14 | %, proportion of MSM received anal swab in the past 12 months |
| 79.37 | 65.38 | 92.75 | %, proportion of MSM received urine test in the past 12 months |
| - | - | - | %, proportion of 'oral sex and anal sex' in the same sex episode |
| - | - | - | %, proportion of 'oral sex and rimming' in the same sex episode |
| - | - | - | %, proportion of saliva use during anal sex |
| - | - | - | %, proportion of receptive oral sex followed by partner's insertive anal sex |
| - | - | - | %, proportion of insertive oral sex followed by partner's insertive rimming |
| 1.25 | 0.36 | 2.39 | %, Infection prevalence of chlamydia trachomatis only at Oropharynx |
| 1.62 | 0.85 | 2.31 | %, Infection prevalence of chlamydia trachomatis only at Urethral |
| 7.45 | 5.92 | 8.93 | %, Infection prevalence of chlamydia trachomatis only at Rectum |
| 0.00 | 0.00 | 0.00 | %, Infection prevalence of chlamydia trachomatis at Oropharynx & Urethra |
| 2.36 | 0.97 | 3.95 | %, Infection prevalence of chlamydia trachomatis at Oropharynx & Rectum |
| 0.62 | 0.19 | 1.10 | %, Infection prevalence of chlamydia trachomatis at Urethra & Rectum |
| 0.00 | 0.00 | 0.00 | %, Infection prevalence of chlamydia trachomatis at Oropharynx & Urethra & Rectum |

**Table S8.24**. Model 4’ output parameters with 95% confidence intervals

| **Mean** | **95%CI: lower** | **95%CI: upper** | **Parameters** |
| --- | --- | --- | --- |
| 43.92 | 36.48 | 55.57 | %, Consistent condom usage in anal sex in past 12 months |
| 86.84 | 83.62 | 92.47 | %, Condom efficacy in preventing transmission |
| - | - | - | Times, Frequency of kissing in the past 12 months |
| 13.82 | 5.16 | 25.82 | Times, Frequency of oral sex in the past 12 months |
| - | - | - | Times, Frequency of rimming in the past 12 months |
| 6.49 | 1.89 | 14.84 | Times, Frequency of anal sex in the past 12 months |
| 132.19 | 49.89 | 177.64 | weeks, Infection duration of chlamydia trachomatis at throat (asymptomatic infection) |
| 1.54 | 1.15 | 1.79 | weeks, Infection duration at urethral (symptomatic infection) |
| 84.27 | 81.69 | 88.44 | %, proportion of urethral infections that are asymptomatic |
| 79.34 | 66.88 | 93.20 | %, proportion of anal infections that are asymptomatic |
| 23.92 | 9.24 | 36.49 | weeks, Infection duration at urethral (asymptomatic infection) |
| 49.75 | 48.41 | 50.84 | weeks, Infection duration of chlamydia trachomatis at anus (asymptomatic infection) |
| 1.56 | 1.14 | 1.91 | weeks, Infection duration of chlamydia trachomatis at anus (symptomatic infection) |
| 82.11 | 69.56 | 92.11 | %, proportion of MSM received throat swab in the past 12 months |
| 77.46 | 67.44 | 88.48 | %, proportion of MSM received anal swab in the past 12 months |
| 78.58 | 67.45 | 89.77 | %, proportion of MSM received urine test in the past 12 months |
| 30.02 | 25.40 | 33.12 | %, proportion of 'oral sex and anal sex' in the same sex episode |
| - | - | - | %, proportion of 'oral sex and rimming' in the same sex episode |
| - | - | - | %, proportion of saliva use during anal sex |
| 80.00 | 80.00 | 80.00 | %, proportion of receptive oral sex followed by partner's insertive anal sex |
| - | - | - | %, proportion of insertive oral sex followed by partner's insertive rimming |
| 1.42 | 0.80 | 2.31 | %, Infection prevalence of chlamydia trachomatis only at Oropharynx |
| 1.62 | 0.95 | 2.15 | %, Infection prevalence of chlamydia trachomatis only at Urethral |
| 7.26 | 6.35 | 8.75 | %, Infection prevalence of chlamydia trachomatis only at Rectum |
| 0.00 | 0.00 | 0.00 | %, Infection prevalence of chlamydia trachomatis at Oropharynx & Urethra |
| 2.63 | 1.25 | 3.67 | %, Infection prevalence of chlamydia trachomatis at Oropharynx & Rectum |
| 0.68 | 0.28 | 1.07 | %, Infection prevalence of chlamydia trachomatis at Urethra & Rectum |
| 0.00 | 0.00 | 0.00 | %, Infection prevalence of chlamydia trachomatis at Oropharynx & Urethra & Rectum |

**Table S8.25.** Model 5’ output parameters with 95% confidence intervals

| **Mean** | **95%CI: lower** | **95%CI: upper** | **Parameters** |
| --- | --- | --- | --- |
| 44.52 | 37.97 | 52.74 | %, Consistent condom usage in anal sex in past 12 months |
| 88.00 | 84.67 | 91.58 | %, Condom efficacy in preventing transmission |
| - | - | - | Times, Frequency of kissing in the past 12 months |
| 17.53 | 9.66 | 24.56 | Times, Frequency of oral sex in the past 12 months |
| - | - | - | Times, Frequency of rimming in the past 12 months |
| 10.92 | 5.08 | 14.66 | Times, Frequency of anal sex in the past 12 months |
| 114.30 | 63.55 | 146.99 | weeks, Infection duration of chlamydia trachomatis at throat (asymptomatic infection) |
| 1.44 | 1.23 | 1.71 | weeks, Infection duration at urethral (symptomatic infection) |
| 85.16 | 82.55 | 87.85 | %, proportion of urethral infections that are asymptomatic |
| 80.47 | 68.19 | 90.42 | %, proportion of anal infections that are asymptomatic |
| 21.84 | 13.00 | 35.41 | weeks, Infection duration at urethral (asymptomatic infection) |
| 49.77 | 48.79 | 50.85 | weeks, Infection duration of chlamydia trachomatis at anus (asymptomatic infection) |
| 1.51 | 1.18 | 1.84 | weeks, Infection duration of chlamydia trachomatis at anus (symptomatic infection) |
| 81.85 | 72.23 | 90.74 | %, proportion of MSM received throat swab in the past 12 months |
| 79.40 | 71.23 | 85.27 | %, proportion of MSM received anal swab in the past 12 months |
| 77.92 | 69.48 | 86.85 | %, proportion of MSM received urine test in the past 12 months |
| - | - | - | %, proportion of 'oral sex and anal sex' in the same sex episode |
| - | - | - | %, proportion of 'oral sex and rimming' in the same sex episode |
| 68.37 | 66.74 | 70.07 | %, proportion of saliva use during anal sex |
| - | - | - | %, proportion of receptive oral sex followed by partner's insertive anal sex |
| - | - | - | %, proportion of insertive oral sex followed by partner's insertive rimming |
| 1.58 | 1.02 | 2.22 | %, Infection prevalence of chlamydia trachomatis only at Oropharynx |
| 1.36 | 0.98 | 1.99 | %, Infection prevalence of chlamydia trachomatis only at Urethral |
| 7.36 | 6.85 | 8.41 | %, Infection prevalence of chlamydia trachomatis only at Rectum |
| 0.00 | 0.00 | 0.00 | %, Infection prevalence of chlamydia trachomatis at Oropharynx & Urethra |
| 2.39 | 1.33 | 3.57 | %, Infection prevalence of chlamydia trachomatis at Oropharynx & Rectum |
| 0.63 | 0.34 | 1.00 | %, Infection prevalence of chlamydia trachomatis at Urethra & Rectum |
| 0.00 | 0.00 | 0.00 | %, Infection prevalence of chlamydia trachomatis at Oropharynx & Urethra & Rectum |

**Table S8.26.** Model 6’ output parameters with 95% confidence intervals

| **Mean** | **95%CI: lower** | **95%CI: upper** | **Parameters** |
| --- | --- | --- | --- |
| 48.96 | 37.26 | 58.43 | %, Consistent condom usage in anal sex in past 12 months |
| 86.17 | 80.47 | 93.61 | %, Condom efficacy in preventing transmission |
| - | - | - | Times, Frequency of kissing in the past 12 months |
| 15.57 | 1.08 | 25.76 | Times, Frequency of oral sex in the past 12 months |
| - | - | - | Times, Frequency of rimming in the past 12 months |
| 20.14 | 3.37 | 35.72 | Times, Frequency of anal sex in the past 12 months |
| 149.59 | 53.67 | 230.37 | weeks, Infection duration of chlamydia trachomatis at throat (asymptomatic infection) |
| 1.49 | 1.10 | 1.95 | weeks, Infection duration at urethral (symptomatic infection) |
| 84.74 | 81.24 | 89.26 | %, proportion of urethral infections that are asymptomatic |
| 83.15 | 66.14 | 96.66 | %, proportion of anal infections that are asymptomatic |
| 22.93 | 3.26 | 47.00 | weeks, Infection duration at urethral (asymptomatic infection) |
| 50.51 | 48.23 | 51.69 | weeks, Infection duration of chlamydia trachomatis at anus (asymptomatic infection) |
| 1.54 | 1.06 | 1.96 | weeks, Infection duration of chlamydia trachomatis at anus (symptomatic infection) |
| 76.86 | 64.34 | 91.71 | %, proportion of MSM received throat swab in the past 12 months |
| 77.52 | 65.59 | 93.73 | %, proportion of MSM received anal swab in the past 12 months |
| 81.93 | 65.56 | 93.75 | %, proportion of MSM received urine test in the past 12 months |
| 29.81 | 26.06 | 33.62 | %, proportion of 'oral sex and anal sex' in the same sex episode |
| - | - | - | %, proportion of 'oral sex and rimming' in the same sex episode |
| 68.31 | 66.13 | 70.87 | %, proportion of saliva use during anal sex |
| 80.00 | 80.00 | 80.00 | %, proportion of receptive oral sex followed by partner's insertive anal sex |
| - | - | - | %, proportion of insertive oral sex followed by partner's insertive rimming |
| 1.66 | 0.40 | 2.47 | %, Infection prevalence of chlamydia trachomatis only at Oropharynx |
| 1.45 | 0.90 | 2.17 | %, Infection prevalence of chlamydia trachomatis only at Urethral |
| 7.22 | 6.07 | 8.54 | %, Infection prevalence of chlamydia trachomatis only at Rectum |
| 0.00 | 0.00 | 0.00 | %, Infection prevalence of chlamydia trachomatis at Oropharynx & Urethra |
| 2.52 | 1.28 | 3.86 | %, Infection prevalence of chlamydia trachomatis at Oropharynx & Rectum |
| 0.77 | 0.25 | 1.10 | %, Infection prevalence of chlamydia trachomatis at Urethra & Rectum |
| 0.00 | 0.00 | 0.00 | %, Infection prevalence of chlamydia trachomatis at Oropharynx & Urethra & Rectum |

Model output parameters (Published validation dataset 2): Data from 393 MSM attending STD & HIV care clinics in the USA

**TableS9. 27.** Model 1’ output parameters with 95% confidence intervals

| **Mean** | **95%CI: lower** | **95%CI: upper** | **Parameters** |
| --- | --- | --- | --- |
| 44.20 | 36.01 | 58.69 | %, Consistent condom usage in anal sex in past 12 months |
| 87.91 | 80.33 | 94.74 | %, Condom efficacy in preventing transmission |
| - | - | - | Times, Frequency of kissing in the past 12 months |
| 15.37 | 3.78 | 27.62 | Times, Frequency of oral sex in the past 12 months |
| - | - | - | Times, Frequency of rimming in the past 12 months |
| 19.61 | 1.83 | 37.70 | Times, Frequency of anal sex in the past 12 months |
| 95.55 | 33.70 | 231.51 | weeks, Infection duration of chlamydia trachomatis at throat (asymptomatic infection) |
| 1.49 | 1.20 | 1.96 | weeks, Infection duration at urethral (symptomatic infection) |
| 85.41 | 80.51 | 89.60 | %, proportion of urethral infections that are asymptomatic |
| 80.93 | 66.09 | 96.05 | %, proportion of anal infections that are asymptomatic |
| 24.92 | 4.29 | 49.37 | weeks, Infection duration at urethral (asymptomatic infection) |
| 49.77 | 48.18 | 51.77 | weeks, Infection duration of chlamydia trachomatis at anus (asymptomatic infection) |
| 1.47 | 1.08 | 1.92 | weeks, Infection duration of chlamydia trachomatis at anus (symptomatic infection) |
| 79.88 | 64.58 | 94.29 | %, proportion of MSM received throat swab in the past 12 months |
| 78.86 | 65.50 | 92.13 | %, proportion of MSM received anal swab in the past 12 months |
| 76.76 | 66.18 | 93.14 | %, proportion of MSM received urine test in the past 12 months |
| - | - | - | %, proportion of 'oral sex and anal sex' in the same sex episode |
| - | - | - | %, proportion of 'oral sex and rimming' in the same sex episode |
| - | - | - | %, proportion of saliva use during anal sex |
| - | - | - | %, proportion of receptive oral sex followed by partner's insertive anal sex |
| - | - | - | %, proportion of insertive oral sex followed by partner's insertive rimming |
| 1.13 | 0.20 | 1.94 | %, Infection prevalence of chlamydia trachomatis only at Oropharynx |
| 2.96 | 1.31 | 4.28 | %, Infection prevalence of chlamydia trachomatis only at Urethral |
| 5.77 | 4.31 | 8.61 | %, Infection prevalence of chlamydia trachomatis only at Rectum |
| 0.00 | 0.00 | 0.00 | %, Infection prevalence of chlamydia trachomatis at Oropharynx & Urethra |
| 1.82 | 0.89 | 3.39 | %, Infection prevalence of chlamydia trachomatis at Oropharynx & Rectum |
| 1.23 | 0.32 | 2.19 | %, Infection prevalence of chlamydia trachomatis at Urethra & Rectum |
| 0.00 | 0.00 | 0.00 | %, Infection prevalence of chlamydia trachomatis at Oropharynx & Urethra & Rectum |

**Table S8.28**. Model 4’ output parameters with 95% confidence intervals

| **Mean** | **95%CI: lower** | **95%CI: upper** | **Parameters** |
| --- | --- | --- | --- |
| 48.18 | 37.14 | 58.15 | %, Consistent condom usage in anal sex in past 12 months |
| 83.72 | 80.85 | 93.40 | %, Condom efficacy in preventing transmission |
| 6.34 | 2.77 | 11.41 | Times, Frequency of kissing in the past 12 months |
| 16.21 | 3.82 | 26.15 | Times, Frequency of oral sex in the past 12 months |
| 48.25 | 10.70 | 74.45 | Times, Frequency of rimming in the past 12 months |
| 14.99 | 3.00 | 32.06 | Times, Frequency of anal sex in the past 12 months |
| 155.53 | 43.82 | 218.06 | weeks, Infection duration of chlamydia trachomatis at throat (asymptomatic infection) |
| 1.52 | 1.22 | 1.89 | weeks, Infection duration at urethral (symptomatic infection) |
| 85.34 | 81.05 | 89.37 | %, proportion of urethral infections that are asymptomatic |
| 82.89 | 66.96 | 95.92 | %, proportion of anal infections that are asymptomatic |
| 29.24 | 6.49 | 49.15 | weeks, Infection duration at urethral (asymptomatic infection) |
| 49.29 | 48.21 | 51.68 | weeks, Infection duration of chlamydia trachomatis at anus (asymptomatic infection) |
| 1.55 | 1.09 | 1.88 | weeks, Infection duration of chlamydia trachomatis at anus (symptomatic infection) |
| 77.88 | 66.32 | 92.66 | %, proportion of MSM received throat swab in the past 12 months |
| 79.86 | 66.53 | 87.82 | %, proportion of MSM received anal swab in the past 12 months |
| 78.63 | 66.86 | 91.94 | %, proportion of MSM received urine test in the past 12 months |
| 28.78 | 25.99 | 32.76 | %, proportion of 'oral sex and anal sex' in the same sex episode |
| - | - | - | %, proportion of 'oral sex and rimming' in the same sex episode |
| - | - | - | %, proportion of saliva use during anal sex |
| 80.00 | 80.00 | 80.00 | %, proportion of receptive oral sex followed by partner's insertive anal sex |
| - | - | - | %, proportion of insertive oral sex followed by partner's insertive rimming |
| 1.43 | 0.24 | 1.87 | %, Infection prevalence of chlamydia trachomatis only at Oropharynx |
| 2.67 | 1.40 | 4.20 | %, Infection prevalence of chlamydia trachomatis only at Urethral |
| 6.55 | 4.59 | 8.37 | %, Infection prevalence of chlamydia trachomatis only at Rectum |
| 0.00 | 0.00 | 0.00 | %, Infection prevalence of chlamydia trachomatis at Oropharynx & Urethra |
| 2.30 | 1.03 | 3.13 | %, Infection prevalence of chlamydia trachomatis at Oropharynx & Rectum |
| 1.29 | 0.46 | 2.01 | %, Infection prevalence of chlamydia trachomatis at Urethra & Rectum |
| 0.00 | 0.00 | 0.00 | %, Infection prevalence of chlamydia trachomatis at Oropharynx & Urethra & Rectum |

**Table S8.29.** Model 5’ output parameters with 95% confidence intervals

| **Mean** | **95%CI: lower** | **95%CI: upper** | **Parameters** |
| --- | --- | --- | --- |
| 47.24 | 37.61 | 55.24 | %, Consistent condom usage in anal sex in past 12 months |
| 86.82 | 82.48 | 92.23 | %, Condom efficacy in preventing transmission |
| - | - | - | Times, Frequency of kissing in the past 12 months |
| 15.83 | 7.11 | 24.16 | Times, Frequency of oral sex in the past 12 months |
| - | - | - | Times, Frequency of rimming in the past 12 months |
| 17.20 | 6.40 | 27.75 | Times, Frequency of anal sex in the past 12 months |
| 114.07 | 55.93 | 185.00 | weeks, Infection duration of chlamydia trachomatis at throat (asymptomatic infection) |
| 1.50 | 1.35 | 1.84 | weeks, Infection duration at urethral (symptomatic infection) |
| 85.19 | 81.98 | 88.79 | %, proportion of urethral infections that are asymptomatic |
| 82.44 | 69.84 | 92.94 | %, proportion of anal infections that are asymptomatic |
| 29.42 | 9.77 | 46.00 | weeks, Infection duration at urethral (asymptomatic infection) |
| 50.16 | 48.33 | 51.32 | weeks, Infection duration of chlamydia trachomatis at anus (asymptomatic infection) |
| 1.38 | 1.15 | 1.82 | weeks, Infection duration of chlamydia trachomatis at anus (symptomatic infection) |
| 78.47 | 69.30 | 89.69 | %, proportion of MSM received throat swab in the past 12 months |
| 74.62 | 68.22 | 86.56 | %, proportion of MSM received anal swab in the past 12 months |
| 79.86 | 68.45 | 91.15 | %, proportion of MSM received urine test in the past 12 months |
| - | - | - | %, proportion of 'oral sex and anal sex' in the same sex episode |
| - | - | - | %, proportion of 'oral sex and rimming' in the same sex episode |
| 68.38 | 66.78 | 70.39 | %, proportion of saliva use during anal sex |
| - | - | - | %, proportion of receptive oral sex followed by partner's insertive anal sex |
| - | - | - | %, proportion of insertive oral sex followed by partner's insertive rimming |
| 1.03 | 0.41 | 1.65 | %, Infection prevalence of chlamydia trachomatis only at Oropharynx |
| 2.60 | 1.41 | 4.07 | %, Infection prevalence of chlamydia trachomatis only at Urethral |
| 6.50 | 5.06 | 8.09 | %, Infection prevalence of chlamydia trachomatis only at Rectum |
| 0.00 | 0.00 | 0.00 | %, Infection prevalence of chlamydia trachomatis at Oropharynx & Urethra |
| 1.69 | 1.14 | 2.71 | %, Infection prevalence of chlamydia trachomatis at Oropharynx & Rectum |
| 1.04 | 0.54 | 1.74 | %, Infection prevalence of chlamydia trachomatis at Urethra & Rectum |
| 0.00 | 0.00 | 0.00 | %, Infection prevalence of chlamydia trachomatis at Oropharynx & Urethra & Rectum |

**Table S8.30.** Model 6’ output parameters with 95% confidence intervals

| **Mean** | **95%CI: lower** | **95%CI: upper** | **Parameters** |
| --- | --- | --- | --- |
| 46.01 | 35.63 | 57.46 | %, Consistent condom usage in anal sex in past 12 months |
| 88.71 | 80.30 | 93.85 | %, Condom efficacy in preventing transmission |
| - | - | - | Times, Frequency of kissing in the past 12 months |
| 11.57 | 0.64 | 27.75 | Times, Frequency of oral sex in the past 12 months |
| - | - | - | Times, Frequency of rimming in the past 12 months |
| 23.40 | 4.40 | 43.00 | Times, Frequency of anal sex in the past 12 months |
| 133.68 | 34.02 | 234.02 | weeks, Infection duration of chlamydia trachomatis at throat (asymptomatic infection) |
| 1.50 | 1.04 | 1.95 | weeks, Infection duration at urethral (symptomatic infection) |
| 84.64 | 80.39 | 89.07 | %, proportion of urethral infections that are asymptomatic |
| 81.19 | 63.52 | 95.75 | %, proportion of anal infections that are asymptomatic |
| 29.49 | 4.21 | 49.21 | weeks, Infection duration at urethral (asymptomatic infection) |
| 49.88 | 48.19 | 51.63 | weeks, Infection duration of chlamydia trachomatis at anus (asymptomatic infection) |
| 1.49 | 1.04 | 1.92 | weeks, Infection duration of chlamydia trachomatis at anus (symptomatic infection) |
| 78.17 | 64.73 | 91.77 | %, proportion of MSM received throat swab in the past 12 months |
| 76.33 | 65.22 | 91.26 | %, proportion of MSM received anal swab in the past 12 months |
| 77.39 | 65.67 | 92.97 | %, proportion of MSM received urine test in the past 12 months |
| 29.01 | 24.92 | 33.16 | %, proportion of 'oral sex and anal sex' in the same sex episode |
| - | - | - | %, proportion of 'oral sex and rimming' in the same sex episode |
| 68.49 | 66.54 | 70.66 | %, proportion of saliva use during anal sex |
| 80.00 | 80.00 | 80.00 | %, proportion of receptive oral sex followed by partner's insertive anal sex |
| - | - | - | %, proportion of insertive oral sex followed by partner's insertive rimming |
| 0.98 | 0.30 | 1.98 | %, Infection prevalence of chlamydia trachomatis only at Oropharynx |
| 2.49 | 1.28 | 4.09 | %, Infection prevalence of chlamydia trachomatis only at Urethral |
| 6.64 | 4.42 | 9.16 | %, Infection prevalence of chlamydia trachomatis only at Rectum |
| 0.00 | 0.00 | 0.00 | %, Infection prevalence of chlamydia trachomatis at Oropharynx & Urethra |
| 2.06 | 0.77 | 3.30 | %, Infection prevalence of chlamydia trachomatis at Oropharynx & Rectum |
| 1.51 | 0.40 | 2.31 | %, Infection prevalence of chlamydia trachomatis at Urethra & Rectum |
| 0.00 | 0.00 | 0.00 | %, Infection prevalence of chlamydia trachomatis at Oropharynx & Urethra & Rectum |

Model output parameters (Published validation dataset3): Data from MSM surveillance data of all Dutch STI clinics

**Table S8.31.** Model 1’ output parameters with 95% confidence intervals

| **Mean** | **95%CI: lower** | **95%CI: upper** | **Parameters** |
| --- | --- | --- | --- |
| 47.74 | 35.41 | 56.65 | %, Consistent condom usage in anal sex in past 12 months |
| 85.46 | 80.85 | 92.13 | %, Condom efficacy in preventing transmission |
| - | - | - | Times, Frequency of kissing in the past 12 months |
| 15.54 | 2.66 | 23.96 | Times, Frequency of oral sex in the past 12 months |
| - | - | - | Times, Frequency of rimming in the past 12 months |
| 10.61 | 4.23 | 23.24 | Times, Frequency of anal sex in the past 12 months |
| 146.28 | 39.04 | 232.37 | weeks, Infection duration of chlamydia trachomatis at throat (asymptomatic infection) |
| 1.48 | 1.12 | 1.97 | weeks, Infection duration at urethral (symptomatic infection) |
| 100.00 | 100.00 | 100.00 | %, proportion of urethral infections that are asymptomatic |
| 100.00 | 100.00 | 100.00 | %, proportion of anal infections that are asymptomatic |
| 32.77 | 10.09 | 49.11 | weeks, Infection duration at urethral (asymptomatic infection) |
| 49.78 | 48.24 | 51.76 | weeks, Infection duration of chlamydia trachomatis at anus (asymptomatic infection) |
| 1.32 | 1.03 | 1.93 | weeks, Infection duration of chlamydia trachomatis at anus (symptomatic infection) |
| 78.72 | 65.20 | 94.12 | %, proportion of MSM received throat swab in the past 12 months |
| 80.37 | 67.39 | 90.38 | %, proportion of MSM received anal swab in the past 12 months |
| 75.03 | 65.46 | 91.12 | %, proportion of MSM received urine test in the past 12 months |
| - | - | - | %, proportion of 'oral sex and anal sex' in the same sex episode |
| - | - | - | %, proportion of 'oral sex and rimming' in the same sex episode |
| - | - | - | %, proportion of saliva use during anal sex |
| - | - | - | %, proportion of receptive oral sex followed by partner's insertive anal sex |
| - | - | - | %, proportion of insertive oral sex followed by partner's insertive rimming |
| 0.51 | 0.48 | 0.53 | %, Infection prevalence of chlamydia trachomatis only at Oropharynx |
| 2.29 | 2.24 | 2.36 | %, Infection prevalence of chlamydia trachomatis only at Urethral |
| 6.29 | 6.18 | 6.40 | %, Infection prevalence of chlamydia trachomatis only at Rectum |
| 0.06 | 0.05 | 0.07 | %, Infection prevalence of chlamydia trachomatis at Oropharynx & Urethra |
| 0.53 | 0.51 | 0.56 | %, Infection prevalence of chlamydia trachomatis at Oropharynx & Rectum |
| 1.02 | 0.98 | 1.06 | %, Infection prevalence of chlamydia trachomatis at Urethra & Rectum |
| 0.10 | 0.09 | 0.12 | %, Infection prevalence of chlamydia trachomatis at Oropharynx & Urethra & Rectum |

**Table S8.32.** Model 4’ output parameters with 95% confidence intervals

| **Mean** | **95%CI: lower** | **95%CI: upper** | **Parameters** |
| --- | --- | --- | --- |
| 44.55 | 37.37 | 53.30 | %, Consistent condom usage in anal sex in past 12 months |
| 87.18 | 81.37 | 90.91 | %, Condom efficacy in preventing transmission |
| - | - | - | Times, Frequency of kissing in the past 12 months |
| 13.43 | 4.77 | 23.22 | Times, Frequency of oral sex in the past 12 months |
| - | - | - | Times, Frequency of rimming in the past 12 months |
| 11.70 | 5.90 | 19.23 | Times, Frequency of anal sex in the past 12 months |
| 167.20 | 61.49 | 221.08 | weeks, Infection duration of chlamydia trachomatis at throat (asymptomatic infection) |
| 1.58 | 1.16 | 1.93 | weeks, Infection duration at urethral (symptomatic infection) |
| 100.00 | 100.00 | 100.00 | %, proportion of urethral infections that are asymptomatic |
| 100.00 | 100.00 | 100.00 | %, proportion of anal infections that are asymptomatic |
| 28.40 | 12.27 | 47.37 | weeks, Infection duration at urethral (asymptomatic infection) |
| 50.54 | 48.64 | 51.57 | weeks, Infection duration of chlamydia trachomatis at anus (asymptomatic infection) |
| 1.49 | 1.17 | 1.91 | weeks, Infection duration of chlamydia trachomatis at anus (symptomatic infection) |
| 79.36 | 69.58 | 92.24 | %, proportion of MSM received throat swab in the past 12 months |
| 79.95 | 68.19 | 89.47 | %, proportion of MSM received anal swab in the past 12 months |
| 77.99 | 67.13 | 89.86 | %, proportion of MSM received urine test in the past 12 months |
| 30.96 | 26.40 | 33.19 | %, proportion of 'oral sex and anal sex' in the same sex episode |
| - | - | - | %, proportion of 'oral sex and rimming' in the same sex episode |
| - | - | - | %, proportion of saliva use during anal sex |
| 80.00 | 80.00 | 80.00 | %, proportion of receptive oral sex followed by partner's insertive anal sex |
| - | - | - | %, proportion of insertive oral sex followed by partner's insertive rimming |
| 0.51 | 0.49 | 0.53 | %, Infection prevalence of chlamydia trachomatis only at Oropharynx |
| 2.30 | 2.25 | 2.35 | %, Infection prevalence of chlamydia trachomatis only at Urethral |
| 6.28 | 6.19 | 6.39 | %, Infection prevalence of chlamydia trachomatis only at Rectum |
| 0.06 | 0.05 | 0.07 | %, Infection prevalence of chlamydia trachomatis at Oropharynx & Urethra |
| 0.54 | 0.52 | 0.56 | %, Infection prevalence of chlamydia trachomatis at Oropharynx & Rectum |
| 1.01 | 0.98 | 1.05 | %, Infection prevalence of chlamydia trachomatis at Urethra & Rectum |
| 0.11 | 0.09 | 0.12 | %, Infection prevalence of chlamydia trachomatis at Oropharynx & Urethra & Rectum |

**Table S8.33.** Model 5’ output parameters with 95% confidence intervals

| **Mean** | **95%CI: lower** | **95%CI: upper** | **Parameters** |
| --- | --- | --- | --- |
| 43.89 | 37.47 | 52.77 | %, Consistent condom usage in anal sex in past 12 months |
| 86.58 | 82.99 | 90.20 | %, Condom efficacy in preventing transmission |
| - | - | - | Times, Frequency of kissing in the past 12 months |
| 9.53 | 4.94 | 21.59 | Times, Frequency of oral sex in the past 12 months |
| - | - | - | Times, Frequency of rimming in the past 12 months |
| 16.07 | 10.46 | 18.66 | Times, Frequency of anal sex in the past 12 months |
| 112.65 | 73.95 | 208.53 | weeks, Infection duration of chlamydia trachomatis at throat (asymptomatic infection) |
| 1.50 | 1.17 | 1.83 | weeks, Infection duration at urethral (symptomatic infection) |
| 100.00 | 100.00 | 100.00 | %, proportion of urethral infections that are asymptomatic |
| 100.00 | 100.00 | 100.00 | %, proportion of anal infections that are asymptomatic |
| 28.89 | 13.13 | 46.78 | weeks, Infection duration at urethral (asymptomatic infection) |
| 50.06 | 48.76 | 51.40 | weeks, Infection duration of chlamydia trachomatis at anus (asymptomatic infection) |
| 1.49 | 1.16 | 1.89 | weeks, Infection duration of chlamydia trachomatis at anus (symptomatic infection) |
| 79.70 | 69.94 | 88.79 | %, proportion of MSM received throat swab in the past 12 months |
| 77.10 | 68.60 | 88.21 | %, proportion of MSM received anal swab in the past 12 months |
| 78.95 | 68.96 | 89.32 | %, proportion of MSM received urine test in the past 12 months |
| - | - | - | %, proportion of 'oral sex and anal sex' in the same sex episode |
| - | - | - | %, proportion of 'oral sex and rimming' in the same sex episode |
| 68.25 | 66.68 | 70.07 | %, proportion of saliva use during anal sex |
| - | - | - | %, proportion of receptive oral sex followed by partner's insertive anal sex |
| - | - | - | %, proportion of insertive oral sex followed by partner's insertive rimming |
| 0.51 | 0.49 | 0.53 | %, Infection prevalence of chlamydia trachomatis only at Oropharynx |
| 2.30 | 2.25 | 2.34 | %, Infection prevalence of chlamydia trachomatis only at Urethral |
| 6.30 | 6.22 | 6.38 | %, Infection prevalence of chlamydia trachomatis only at Rectum |
| 0.06 | 0.05 | 0.07 | %, Infection prevalence of chlamydia trachomatis at Oropharynx & Urethra |
| 0.53 | 0.52 | 0.56 | %, Infection prevalence of chlamydia trachomatis at Oropharynx & Rectum |
| 1.01 | 0.98 | 1.04 | %, Infection prevalence of chlamydia trachomatis at Urethra & Rectum |
| 0.11 | 0.09 | 0.11 | %, Infection prevalence of chlamydia trachomatis at Oropharynx & Urethra & Rectum |

**Table S8.34.** Model 6’ output parameters with 95% confidence intervals

| **Mean** | **95%CI: lower** | **95%CI: upper** | **Parameters** |
| --- | --- | --- | --- |
| 43.13 | 35.78 | 56.16 | %, Consistent condom usage in anal sex in past 12 months |
| 87.54 | 80.66 | 93.99 | %, Condom efficacy in preventing transmission |
| - | - | - | Times, Frequency of kissing in the past 12 months |
| 4.06 | 0.26 | 14.30 | Times, Frequency of oral sex in the past 12 months |
| - | - | - | Times, Frequency of rimming in the past 12 months |
| 22.96 | 11.39 | 34.20 | Times, Frequency of anal sex in the past 12 months |
| 164.40 | 75.10 | 218.22 | weeks, Infection duration of chlamydia trachomatis at throat (asymptomatic infection) |
| 1.51 | 1.23 | 1.92 | weeks, Infection duration at urethral (symptomatic infection) |
| 100.00 | 100.00 | 100.00 | %, proportion of urethral infections that are asymptomatic |
| 100.00 | 100.00 | 100.00 | %, proportion of anal infections that are asymptomatic |
| 33.37 | 14.92 | 45.59 | weeks, Infection duration at urethral (asymptomatic infection) |
| 50.36 | 48.51 | 51.83 | weeks, Infection duration of chlamydia trachomatis at anus (asymptomatic infection) |
| 1.62 | 1.24 | 1.95 | weeks, Infection duration of chlamydia trachomatis at anus (symptomatic infection) |
| 78.42 | 66.44 | 90.15 | %, proportion of MSM received throat swab in the past 12 months |
| 74.20 | 65.46 | 92.42 | %, proportion of MSM received anal swab in the past 12 months |
| 75.59 | 66.44 | 92.70 | %, proportion of MSM received urine test in the past 12 months |
| 30.63 | 26.95 | 33.58 | %, proportion of 'oral sex and anal sex' in the same sex episode |
| - | - | - | %, proportion of 'oral sex and rimming' in the same sex episode |
| 68.18 | 66.28 | 70.77 | %, proportion of saliva use during anal sex |
| 80.00 | 80.00 | 80.00 | %, proportion of receptive oral sex followed by partner's insertive anal sex |
| - | - | - | %, proportion of insertive oral sex followed by partner's insertive rimming |
| 0.51 | 0.49 | 0.54 | %, Infection prevalence of chlamydia trachomatis only at Oropharynx |
| 2.29 | 2.24 | 2.35 | %, Infection prevalence of chlamydia trachomatis only at Urethral |
| 6.30 | 6.18 | 6.41 | %, Infection prevalence of chlamydia trachomatis only at Rectum |
| 0.06 | 0.05 | 0.07 | %, Infection prevalence of chlamydia trachomatis at Oropharynx & Urethra |
| 0.53 | 0.51 | 0.58 | %, Infection prevalence of chlamydia trachomatis at Oropharynx & Rectum |
| 1.01 | 0.97 | 1.06 | %, Infection prevalence of chlamydia trachomatis at Urethra & Rectum |
| 0.10 | 0.09 | 0.12 | %, Infection prevalence of chlamydia trachomatis at Oropharynx & Urethra & Rectum |

Model output parameters (Published validation dataset 4): Data from 1,610 community MSM in Thailand

**Table S8.35.** Model 1’ output parameters with 95% confidence intervals

| **Mean** | **95%CI: lower** | **95%CI: upper** | **Parameters** |
| --- | --- | --- | --- |
| 44.48 | 35.57 | 57.26 | %, Consistent condom usage in anal sex in past 12 months |
| 87.86 | 80.59 | 94.38 | %, Condom efficacy in preventing transmission |
| - | - | - | Times, Frequency of kissing in the past 12 months |
| 20.89 | 4.35 | 26.86 | Times, Frequency of oral sex in the past 12 months |
| - | - | - | Times, Frequency of rimming in the past 12 months |
| 13.87 | 1.68 | 42.97 | Times, Frequency of anal sex in the past 12 months |
| 146.28 | 34.04 | 237.39 | weeks, Infection duration of chlamydia trachomatis at throat (asymptomatic infection) |
| 1.33 | 1.03 | 1.91 | weeks, Infection duration at urethral (symptomatic infection) |
| 100.00 | 100.00 | 100.00 | %, proportion of urethral infections that are asymptomatic |
| 100.00 | 100.00 | 100.00 | %, proportion of anal infections that are asymptomatic |
| 29.15 | 4.73 | 50.25 | weeks, Infection duration at urethral (asymptomatic infection) |
| 50.16 | 48.19 | 51.78 | weeks, Infection duration of chlamydia trachomatis at anus (asymptomatic infection) |
| 1.40 | 1.03 | 1.94 | weeks, Infection duration of chlamydia trachomatis at anus (symptomatic infection) |
| 77.06 | 64.34 | 94.19 | %, proportion of MSM received throat swab in the past 12 months |
| 78.12 | 64.83 | 92.36 | %, proportion of MSM received anal swab in the past 12 months |
| 78.98 | 66.34 | 93.64 | %, proportion of MSM received urine test in the past 12 months |
| - | - | - | %, proportion of 'oral sex and anal sex' in the same sex episode |
| - | - | - | %, proportion of 'oral sex and rimming' in the same sex episode |
| - | - | - | %, proportion of saliva use during anal sex |
| - | - | - | %, proportion of receptive oral sex followed by partner's insertive anal sex |
| - | - | - | %, proportion of insertive oral sex followed by partner's insertive rimming |
| 1.70 | 1.25 | 2.41 | %, Infection prevalence of chlamydia trachomatis only at Oropharynx |
| 5.23 | 4.15 | 5.83 | %, Infection prevalence of chlamydia trachomatis only at Urethral |
| 11.89 | 10.44 | 13.37 | %, Infection prevalence of chlamydia trachomatis only at Rectum |
| 0.22 | 0.03 | 0.38 | %, Infection prevalence of chlamydia trachomatis at Oropharynx & Urethra |
| 1.12 | 0.79 | 1.68 | %, Infection prevalence of chlamydia trachomatis at Oropharynx & Rectum |
| 2.17 | 1.66 | 2.68 | %, Infection prevalence of chlamydia trachomatis at Urethra & Rectum |
| 0.21 | 0.03 | 0.38 | %, Infection prevalence of chlamydia trachomatis at Oropharynx & Urethra & Rectum |

**Table S8.36.** Model 4’ output parameters with 95% confidence intervals

| **Mean** | **95%CI: lower** | **95%CI: upper** | **Parameters** |
| --- | --- | --- | --- |
| 46.67 | 37.27 | 56.76 | %, Consistent condom usage in anal sex in past 12 months |
| 85.28 | 81.86 | 93.20 | %, Condom efficacy in preventing transmission |
| - | - | - | Times, Frequency of kissing in the past 12 months |
| 16.79 | 4.30 | 24.56 | Times, Frequency of oral sex in the past 12 months |
| - | - | - | Times, Frequency of rimming in the past 12 months |
| 19.75 | 3.50 | 35.07 | Times, Frequency of anal sex in the past 12 months |
| 181.39 | 45.32 | 230.33 | weeks, Infection duration of chlamydia trachomatis at throat(asymptomatic infection) |
| 1.48 | 1.06 | 1.87 | weeks, Infection duration at urethral (symptomatic infection) |
| 100.00 | 100.00 | 100.00 | %, proportion of urethral infections that are asymptomatic |
| 100.00 | 100.00 | 100.00 | %, proportion of anal infections that are asymptomatic |
| 24.85 | 4.93 | 47.97 | weeks, Infection duration at urethral (asymptomatic infection) |
| 49.91 | 48.24 | 51.75 | weeks, Infection duration of chlamydia trachomatis at anus (asymptomatic infection) |
| 1.53 | 1.09 | 1.87 | weeks, Infection duration of chlamydia trachomatis at anus (symptomatic infection) |
| 77.24 | 65.63 | 93.13 | %, proportion of MSM received throat swab in the past 12 months |
| 78.51 | 68.23 | 90.62 | %, proportion of MSM received anal swab in the past 12 months |
| 78.73 | 67.01 | 92.35 | %, proportion of MSM received urine test in the past 12 months |
| 30.41 | 26.58 | 32.73 | %, proportion of 'oral sex and anal sex' in the same sex episode |
| - | - | - | %, proportion of 'oral sex and rimming' in the same sex episode |
| - | - | - | %, proportion of saliva use during anal sex |
| 80.00 | 80.00 | 80.00 | %, proportion of receptive oral sex followed by partner's insertive anal sex |
| - | - | - | %, proportion of insertive oral sex followed by partner's insertive rimming |
| 1.82 | 1.32 | 2.23 | %, Infection prevalence of chlamydia trachomatis only at Oropharynx |
| 5.03 | 4.16 | 5.74 | %, Infection prevalence of chlamydia trachomatis only at Urethral |
| 11.19 | 10.51 | 12.76 | %, Infection prevalence of chlamydia trachomatis only at Rectum |
| 0.21 | 0.05 | 0.35 | %, Infection prevalence of chlamydia trachomatis at Oropharynx & Urethra |
| 1.29 | 0.81 | 1.68 | %, Infection prevalence of chlamydia trachomatis at Oropharynx & Rectum |
| 2.30 | 1.75 | 2.60 | %, Infection prevalence of chlamydia trachomatis at Urethra & Rectum |
| 0.20 | 0.04 | 0.33 | %, Infection prevalence of chlamydia trachomatis at Oropharynx & Urethra & Rectum |

**Table S8.37**. Model 5’ output parameters with 95% confidence intervals

| **Mean** | **95%CI: lower** | **95%CI: upper** | **Parameters** |
| --- | --- | --- | --- |
| 48.00 | 37.56 | 55.88 | %, Consistent condom usage in anal sex in past 12 months |
| 89.06 | 82.71 | 92.41 | %, Condom efficacy in preventing transmission |
| - | - | - | Times, Frequency of kissing in the past 12 months |
| 15.80 | 5.64 | 23.71 | Times, Frequency of oral sex in the past 12 months |
| - | - | - | Times, Frequency of rimming in the past 12 months |
| 24.54 | 6.96 | 32.74 | Times, Frequency of anal sex in the past 12 months |
| 116.27 | 54.42 | 221.85 | weeks, Infection duration of chlamydia trachomatis at throat (asymptomatic infection) |
| 1.54 | 1.10 | 1.84 | weeks, Infection duration at urethral (symptomatic infection) |
| 100.00 | 100.00 | 100.00 | %, proportion of urethral infections that are asymptomatic |
| 100.00 | 100.00 | 100.00 | %, proportion of anal infections that are asymptomatic |
| 25.04 | 9.75 | 42.76 | weeks, Infection duration at urethral (asymptomatic infection) |
| 49.42 | 48.32 | 51.59 | weeks, Infection duration of chlamydia trachomatis at anus (asymptomatic infection) |
| 1.57 | 1.20 | 1.84 | weeks, Infection duration of chlamydia trachomatis at anus (symptomatic infection) |
| 81.28 | 67.90 | 91.59 | %, proportion of MSM received throat swab in the past 12 months |
| 77.98 | 69.35 | 89.65 | %, proportion of MSM received anal swab in the past 12 months |
| 79.31 | 67.66 | 91.00 | %, proportion of MSM received urine test in the past 12 months |
| - | - | - | %, proportion of 'oral sex and anal sex' in the same sex episode |
| - | - | - | %, proportion of 'oral sex and rimming' in the same sex episode |
| 68.53 | 66.69 | 70.27 | %, proportion of saliva use during anal sex |
| - | - | - | %, proportion of receptive oral sex followed by partner's insertive anal sex |
| - | - | - | %, proportion of insertive oral sex followed by partner's insertive rimming |
| 1.88 | 1.40 | 2.18 | %, Infection prevalence of chlamydia trachomatis only at Oropharynx |
| 4.99 | 4.21 | 5.63 | %, Infection prevalence of chlamydia trachomatis only at Urethral |
| 11.69 | 10.63 | 12.61 | %, Infection prevalence of chlamydia trachomatis only at Rectum |
| 0.18 | 0.07 | 0.34 | %, Infection prevalence of chlamydia trachomatis at Oropharynx & Urethra |
| 1.11 | 0.86 | 1.61 | %, Infection prevalence of chlamydia trachomatis at Oropharynx & Rectum |
| 2.12 | 1.79 | 2.55 | %, Infection prevalence of chlamydia trachomatis at Urethra & Rectum |
| 0.17 | 0.05 | 0.30 | %, Infection prevalence of chlamydia trachomatis at Oropharynx & Urethra & Rectum |

**Table S8. 38**. Model 6’ output parameters with 95% confidence intervals

| **Mean** | **95%CI: lower** | **95%CI: upper** | **Parameters** |
| --- | --- | --- | --- |
| 46.12 | 36.96 | 55.37 | %, Consistent condom usage in anal sex in past 12 months |
| 85.56 | 81.66 | 91.68 | %, Condom efficacy in preventing transmission |
| - | - | - | Times, Frequency of kissing in the past 12 months |
| 12.68 | 0.38 | 23.89 | Times, Frequency of oral sex in the past 12 months |
| - | - | - | Times, Frequency of rimming in the past 12 months |
| 20.12 | 4.04 | 50.64 | Times, Frequency of anal sex in the past 12 months |
| 144.72 | 56.65 | 227.75 | weeks, Infection duration of chlamydia trachomatis at throat (asymptomatic infection) |
| 1.58 | 1.13 | 1.90 | weeks, Infection duration at urethral (symptomatic infection) |
| 100.00 | 100.00 | 100.00 | %, proportion of urethral infections that are asymptomatic |
| 100.00 | 100.00 | 100.00 | %, proportion of anal infections that are asymptomatic |
| 26.57 | 9.46 | 46.37 | weeks, Infection duration at urethral (asymptomatic infection) |
| 49.71 | 48.50 | 51.61 | weeks, Infection duration of chlamydia trachomatis at anus (asymptomatic infection) |
| 1.51 | 1.10 | 1.89 | weeks, Infection duration of chlamydia trachomatis at anus (symptomatic infection) |
| 72.80 | 65.80 | 91.06 | %, proportion of MSM received throat swab in the past 12 months |
| 80.57 | 65.77 | 90.89 | %, proportion of MSM received anal swab in the past 12 months |
| 79.69 | 65.47 | 91.68 | %, proportion of MSM received urine test in the past 12 months |
| 29.87 | 25.39 | 33.16 | %, proportion of 'oral sex and anal sex' in the same sex episode |
| - | - | - | %, proportion of 'oral sex and rimming' in the same sex episode |
| 68.04 | 66.21 | 70.36 | %, proportion of saliva use during anal sex |
| 80.00 | 80.00 | 80.00 | %, proportion of receptive oral sex followed by partner's insertive anal sex |
| - | - | - | %, proportion of insertive oral sex followed by partner's insertive rimming |
| 1.74 | 1.23 | 2.47 | %, Infection prevalence of chlamydia trachomatis only at Oropharynx |
| 4.91 | 4.08 | 5.88 | %, Infection prevalence of chlamydia trachomatis only at Urethral |
| 11.82 | 10.47 | 13.25 | %, Infection prevalence of chlamydia trachomatis only at Rectum |
| 0.19 | 0.02 | 0.35 | %, Infection prevalence of chlamydia trachomatis at Oropharynx & Urethra |
| 1.15 | 0.73 | 1.58 | %, Infection prevalence of chlamydia trachomatis at Oropharynx & Rectum |
| 2.11 | 1.58 | 2.74 | %, Infection prevalence of chlamydia trachomatis at Urethra & Rectum |
| 0.18 | 0.01 | 0.38 | %, Infection prevalence of chlamydia trachomatis at Oropharynx & Urethra & Rectum |

Model output parameters (Published validation dataset 5): Data from 179 MSM with HIV in the USA

**Table S8.39**. Model 1’ output parameters with 95% confidence intervals

| **Mean** | **95%CI: lower** | **95%CI: upper** | **Parameters** |
| --- | --- | --- | --- |
| 47.10 | 35.21 | 58.70 | %, Consistent condom usage in anal sex in past 12 months |
| 87.06 | 80.62 | 93.07 | %, Condom efficacy in preventing transmission |
| - | - | - | Times, Frequency of kissing in the past 12 months |
| 14.76 | 2.51 | 24.57 | Times, Frequency of oral sex in the past 12 months |
| - | - | - | Times, Frequency of rimming in the past 12 months |
| 12.02 | 1.86 | 33.60 | Times, Frequency of anal sex in the past 12 months |
| 115.06 | 27.90 | 227.64 | weeks, Infection duration of chlamydia trachomatis at throat (asymptomatic infection) |
| 1.47 | 1.08 | 1.97 | weeks, Infection duration at urethral (symptomatic infection) |
| 83.63 | 80.47 | 89.65 | %, proportion of urethral infections that are asymptomatic |
| 84.25 | 65.24 | 95.23 | %, proportion of anal infections that are asymptomatic |
| 33.29 | 10.32 | 51.10 | weeks, Infection duration at urethral (asymptomatic infection) |
| 49.67 | 48.26 | 51.38 | weeks, Infection duration of chlamydia trachomatis at anus (asymptomatic infection) |
| 1.59 | 1.26 | 1.99 | weeks, Infection duration of chlamydia trachomatis at anus (symptomatic infection) |
| 84.21 | 64.83 | 94.99 | %, proportion of MSM received throat swab in the past 12 months |
| 77.29 | 64.94 | 93.19 | %, proportion of MSM received anal swab in the past 12 months |
| 84.79 | 65.21 | 92.77 | %, proportion of MSM received urine test in the past 12 months |
| - | - | - | %, proportion of 'oral sex and anal sex' in the same sex episode |
| - | - | - | %, proportion of 'oral sex and rimming' in the same sex episode |
| - | - | - | %, proportion of saliva use during anal sex |
| - | - | - | %, proportion of receptive oral sex followed by partner's insertive anal sex |
| - | - | - | %, proportion of insertive oral sex followed by partner's insertive rimming |
| 0.00 | 0.00 | 0.00 | %, Infection prevalence of chlamydia trachomatis only at Oropharynx |
| 2.43 | 0.84 | 4.33 | %, Infection prevalence of chlamydia trachomatis only at Urethral |
| 15.21 | 8.81 | 17.84 | %, Infection prevalence of chlamydia trachomatis only at Rectum |
| 0.00 | 0.00 | 0.00 | %, Infection prevalence of chlamydia trachomatis at Oropharynx & Urethra |
| 0.00 | 0.00 | 0.00 | %, Infection prevalence of chlamydia trachomatis at Oropharynx & Rectum |
| 2.83 | 0.76 | 4.17 | %, Infection prevalence of chlamydia trachomatis at Urethra & Rectum |
| 0.00 | 0.00 | 0.00 | %, Infection prevalence of chlamydia trachomatis at Oropharynx & Urethra & Rectum |

**Table S8.40**. Model 4’ output parameters with 95% confidence intervals

| **Mean** | **95%CI: lower** | **95%CI: upper** | **Parameters** |
| --- | --- | --- | --- |
| 44.16 | 36.19 | 55.86 | %, Consistent condom usage in anal sex in past 12 months |
| 86.93 | 81.34 | 92.04 | %, Condom efficacy in preventing transmission |
| - | - | - | Times, Frequency of kissing in the past 12 months |
| 14.22 | 3.28 | 24.66 | Times, Frequency of oral sex in the past 12 months |
| - | - | - | Times, Frequency of rimming in the past 12 months |
| 12.52 | 2.37 | 18.66 | Times, Frequency of anal sex in the past 12 months |
| 116.65 | 30.58 | 207.51 | weeks, Infection duration of chlamydia trachomatis at throat (asymptomatic infection) |
| 1.46 | 1.11 | 1.94 | weeks, Infection duration at urethral (symptomatic infection) |
| 85.83 | 81.01 | 89.56 | %, proportion of urethral infections that are asymptomatic |
| 78.48 | 66.29 | 94.49 | %, proportion of anal infections that are asymptomatic |
| 32.35 | 11.16 | 46.97 | weeks, Infection duration at urethral (asymptomatic infection) |
| 49.96 | 48.51 | 51.22 | weeks, Infection duration of chlamydia trachomatis at anus (asymptomatic infection) |
| 1.59 | 1.30 | 1.90 | weeks, Infection duration of chlamydia trachomatis at anus (symptomatic infection) |
| 75.29 | 65.25 | 94.21 | %, proportion of MSM received throat swab in the past 12 months |
| 79.24 | 66.32 | 92.15 | %, proportion of MSM received anal swab in the past 12 months |
| 78.92 | 66.62 | 90.56 | %, proportion of MSM received urine test in the past 12 months |
| 30.22 | 25.60 | 32.95 | %, proportion of 'oral sex and anal sex' in the same sex episode |
| - | - | - | %, proportion of 'oral sex and rimming' in the same sex episode |
| - | - | - | %, proportion of saliva use during anal sex |
| 80.00 | 80.00 | 80.00 | %, proportion of receptive oral sex followed by partner's insertive anal sex |
| - | - | - | %, proportion of insertive oral sex followed by partner's insertive rimming |
| 0.00 | 0.00 | 0.00 | %, Infection prevalence of chlamydia trachomatis only at Oropharynx |
| 2.68 | 0.98 | 4.32 | %, Infection prevalence of chlamydia trachomatis only at Urethral |
| 13.32 | 8.79 | 17.00 | %, Infection prevalence of chlamydia trachomatis only at Rectum |
| 0.00 | 0.00 | 0.00 | %, Infection prevalence of chlamydia trachomatis at Oropharynx & Urethra |
| 0.00 | 0.00 | 0.00 | %, Infection prevalence of chlamydia trachomatis at Oropharynx & Rectum |
| 2.83 | 0.88 | 4.13 | %, Infection prevalence of chlamydia trachomatis at Urethra & Rectum |
| 0.00 | 0.00 | 0.00 | %, Infection prevalence of chlamydia trachomatis at Oropharynx & Urethra & Rectum |

**Table S8. 41.** Model 5’ output parameters with 95% confidence intervals

| **Mean** | **95%CI: lower** | **95%CI: upper** | **Parameters** |
| --- | --- | --- | --- |
| 43.82 | 37.94 | 51.56 | %, Consistent condom usage in anal sex in past 12 months |
| 86.59 | 81.84 | 91.21 | %, Condom efficacy in preventing transmission |
| - | - | - | Times, Frequency of kissing in the past 12 months |
| 15.44 | 7.50 | 23.86 | Times, Frequency of oral sex in the past 12 months |
| - | - | - | Times, Frequency of rimming in the past 12 months |
| 9.59 | 4.31 | 15.93 | Times, Frequency of anal sex in the past 12 months |
| 98.50 | 55.30 | 180.33 | weeks, Infection duration of chlamydia trachomatis at throat (asymptomatic infection) |
| 1.51 | 1.20 | 1.88 | weeks, Infection duration at urethral (symptomatic infection) |
| 85.57 | 83.30 | 88.40 | %, proportion of urethral infections that are asymptomatic |
| 81.20 | 72.49 | 93.44 | %, proportion of anal infections that are asymptomatic |
| 32.65 | 14.18 | 42.47 | weeks, Infection duration at urethral (asymptomatic infection) |
| 49.78 | 48.90 | 50.75 | weeks, Infection duration of chlamydia trachomatis at anus (asymptomatic infection) |
| 1.59 | 1.33 | 1.84 | weeks, Infection duration of chlamydia trachomatis at anus (symptomatic infection) |
| 82.66 | 68.62 | 89.31 | %, proportion of MSM received throat swab in the past 12 months |
| 79.70 | 68.56 | 87.57 | %, proportion of MSM received anal swab in the past 12 months |
| 80.36 | 71.27 | 89.01 | %, proportion of MSM received urine test in the past 12 months |
| - | - | - | %, proportion of 'oral sex and anal sex' in the same sex episode |
| - | - | - | %, proportion of 'oral sex and rimming' in the same sex episode |
| 68.43 | 66.93 | 69.59 | %, proportion of saliva use during anal sex |
| - | - | - | %, proportion of receptive oral sex followed by partner's insertive anal sex |
| - | - | - | %, proportion of insertive oral sex followed by partner's insertive rimming |
| 0.00 | 0.00 | 0.00 | %, Infection prevalence of chlamydia trachomatis only at Oropharynx |
| 2.82 | 1.47 | 4.12 | %, Infection prevalence of chlamydia trachomatis only at Urethral |
| 12.54 | 9.99 | 15.78 | %, Infection prevalence of chlamydia trachomatis only at Rectum |
| 0.00 | 0.00 | 0.00 | %, Infection prevalence of chlamydia trachomatis at Oropharynx & Urethra |
| 0.00 | 0.00 | 0.00 | %, Infection prevalence of chlamydia trachomatis at Oropharynx & Rectum |
| 2.21 | 1.35 | 3.40 | %, Infection prevalence of chlamydia trachomatis at Urethra & Rectum |
| 0.00 | 0.00 | 0.00 | %, Infection prevalence of chlamydia trachomatis at Oropharynx & Urethra & Rectum |

**Table S8. 42**. Model 6’ output parameters with 95% confidence intervals

| **Mean** | **95%CI: lower** | **95%CI: upper** | **Parameters** |
| --- | --- | --- | --- |
| 46.52 | 36.11 | 55.44 | %, Consistent condom usage in anal sex in past 12 months |
| 87.19 | 82.24 | 91.58 | %, Condom efficacy in preventing transmission |
| - | - | - | Times, Frequency of kissing in the past 12 months |
| 17.16 | 6.13 | 26.36 | Times, Frequency of oral sex in the past 12 months |
| - | - | - | Times, Frequency of rimming in the past 12 months |
| 10.17 | 6.31 | 19.56 | Times, Frequency of anal sex in the past 12 months |
| 143.34 | 42.99 | 199.98 | weeks, Infection duration of chlamydia trachomatis at throat (asymptomatic infection) |
| 1.48 | 1.14 | 1.75 | weeks, Infection duration at urethral (symptomatic infection) |
| 84.26 | 81.13 | 87.97 | %, proportion of urethral infections that are asymptomatic |
| 83.15 | 65.11 | 93.32 | %, proportion of anal infections that are asymptomatic |
| 27.98 | 7.21 | 46.55 | weeks, Infection duration at urethral (asymptomatic infection) |
| 50.34 | 48.53 | 51.38 | weeks, Infection duration of chlamydia trachomatis at anus (asymptomatic infection) |
| 1.49 | 1.10 | 1.94 | weeks, Infection duration of chlamydia trachomatis at anus (symptomatic infection) |
| 79.36 | 65.98 | 91.91 | %, proportion of MSM received throat swab in the past 12 months |
| 77.19 | 67.32 | 89.72 | %, proportion of MSM received anal swab in the past 12 months |
| 80.36 | 67.49 | 92.15 | %, proportion of MSM received urine test in the past 12 months |
| 29.66 | 26.12 | 33.23 | %, proportion of 'oral sex and anal sex' in the same sex episode |
| - | - | - | %, proportion of 'oral sex and rimming' in the same sex episode |
| 68.48 | 66.77 | 70.57 | %, proportion of saliva use during anal sex |
| 80.00 | 80.00 | 80.00 | %, proportion of receptive oral sex followed by partner's insertive anal sex |
| - | - | - | %, proportion of insertive oral sex followed by partner's insertive rimming |
| 0.00 | 0.00 | 0.00 | %, Infection prevalence of chlamydia trachomatis only at Oropharynx |
| 2.92 | 1.37 | 4.48 | %, Infection prevalence of chlamydia trachomatis only at Urethral |
| 13.16 | 10.26 | 16.81 | %, Infection prevalence of chlamydia trachomatis only at Rectum |
| 0.00 | 0.00 | 0.00 | %, Infection prevalence of chlamydia trachomatis at Oropharynx & Urethra |
| 0.00 | 0.00 | 0.00 | %, Infection prevalence of chlamydia trachomatis at Oropharynx & Rectum |
| 2.55 | 0.63 | 3.85 | %, Infection prevalence of chlamydia trachomatis at Urethra & Rectum |
| 0.00 | 0.00 | 0.00 | %, Infection prevalence of chlamydia trachomatis at Oropharynx & Urethra & Rectum |

‘Anal sex, oral sex, rimming and sequential sexual practices’ transmission models (Model 2, 7-13)

Model output parameters (Calibration Dataset): Unpublished calibration data from 4888 MSM attending Melbourne Sexual Health Centre

**Table S8. 43**. Model 2’ output parameters with 95% confidence intervals

| **Mean** | **95%CI: lower** | **95%CI: upper** | **Parameters** |
| --- | --- | --- | --- |
| 44.92 | 35.37 | 56.36 | %, Consistent condom usage in anal sex in past 12 months |
| 87.18 | 80.81 | 93.81 | %, Condom efficacy in preventing transmission |
| - | - | - | Times, Frequency of kissing in the past 12 months |
| 15.27 | 2.44 | 27.70 | Times, Frequency of oral sex in the past 12 months |
| 28.08 | 4.93 | 76.16 | Times, Frequency of rimming in the past 12 months |
| 13.43 | 1.16 | 39.12 | Times, Frequency of anal sex in the past 12 months |
| 112.41 | 32.72 | 218.24 | weeks, Infection duration of chlamydia trachomatis at throat (asymptomatic infection) |
| 1.49 | 1.05 | 1.93 | weeks, Infection duration at urethral (symptomatic infection) |
| 85.56 | 81.38 | 89.29 | %, proportion of urethral infections that are asymptomatic |
| 80.49 | 65.95 | 96.31 | %, proportion of anal infections that are asymptomatic |
| 28.32 | 6.20 | 47.55 | weeks, Infection duration at urethral (asymptomatic infection) |
| 50.38 | 48.20 | 51.73 | weeks, Infection duration of chlamydia trachomatis at anus (asymptomatic infection) |
| 1.59 | 1.14 | 1.95 | weeks, Infection duration of chlamydia trachomatis at anus (symptomatic infection) |
| 81.12 | 65.11 | 91.11 | %, proportion of MSM received throat swab in the past 12 months |
| 78.05 | 64.78 | 92.65 | %, proportion of MSM received anal swab in the past 12 months |
| 77.76 | 64.71 | 92.37 | %, proportion of MSM received urine test in the past 12 months |
| 28.29 | 24.94 | 33.34 | %, proportion of 'oral sex and anal sex' in the same sex episode |
| 71.51 | 69.87 | 72.77 | %, proportion of 'oral sex and rimming' in the same sex episode |
| 68.77 | 66.34 | 70.85 | %, proportion of saliva use during anal sex |
| 80.00 | 80.00 | 80.00 | %, proportion of receptive oral sex followed by partner's insertive anal sex |
| 80.00 | 80.00 | 80.00 | %, proportion of insertive oral sex followed by partner's insertive rimming |
| 0.84 | 0.63 | 1.02 | %, Infection prevalence of chlamydia trachomatis only at Oropharynx |
| 1.95 | 1.59 | 2.25 | %, Infection prevalence of chlamydia trachomatis only at Urethral |
| 7.54 | 6.89 | 8.25 | %, Infection prevalence of chlamydia trachomatis only at Rectum |
| 0.00 | 0.00 | 0.00 | %, Infection prevalence of chlamydia trachomatis at Oropharynx & Urethra |
| 1.20 | 0.95 | 1.51 | %, Infection prevalence of chlamydia trachomatis at Oropharynx & Rectum |
| 1.18 | 0.97 | 1.53 | %, Infection prevalence of chlamydia trachomatis at Urethra & Rectum |
| 0.22 | 0.09 | 0.31 | %, Infection prevalence of chlamydia trachomatis at Oropharynx & Urethra & Rectum |

**Table S8. 44.** Model 7’ output parameters with 95% confidence intervals

| **Mean** | **95%CI: lower** | **95%CI: upper** | **Parameters** |
| --- | --- | --- | --- |
| 44.44 | 38.20 | 54.56 | %, Consistent condom usage in anal sex in past 12 months |
| 86.38 | 82.28 | 92.32 | %, Condom efficacy in preventing transmission |
| - | - | - | Times, Frequency of kissing in the past 12 months |
| 18.60 | 6.66 | 26.96 | Times, Frequency of oral sex in the past 12 months |
| 47.38 | 13.42 | 70.44 | Times, Frequency of rimming in the past 12 months |
| 9.79 | 2.58 | 20.74 | Times, Frequency of anal sex in the past 12 months |
| 132.06 | 55.98 | 210.04 | weeks, Infection duration of chlamydia trachomatis at throat (asymptomatic infection) |
| 1.50 | 1.10 | 1.86 | weeks, Infection duration at urethral (symptomatic infection) |
| 86.33 | 82.36 | 88.50 | %, proportion of urethral infections that are asymptomatic |
| 81.03 | 67.10 | 91.00 | %, proportion of anal infections that are asymptomatic |
| 24.50 | 7.13 | 40.98 | weeks, Infection duration at urethral (asymptomatic infection) |
| 50.28 | 48.57 | 51.49 | weeks, Infection duration of chlamydia trachomatis at anus (asymptomatic infection) |
| 1.49 | 1.23 | 1.93 | weeks, Infection duration of chlamydia trachomatis at anus (symptomatic infection) |
| 73.30 | 67.13 | 89.00 | %, proportion of MSM received throat swab in the past 12 months |
| 76.93 | 67.95 | 89.75 | %, proportion of MSM received anal swab in the past 12 months |
| 80.05 | 67.83 | 90.69 | %, proportion of MSM received urine test in the past 12 months |
| 30.44 | 26.26 | 32.71 | %, proportion of 'oral sex and anal sex' in the same sex episode |
| 71.27 | 70.14 | 72.60 | %, proportion of 'oral sex and rimming' in the same sex episode |
| 69.04 | 67.02 | 70.46 | %, proportion of saliva use during anal sex |
| 80.00 | 80.00 | 80.00 | %, proportion of receptive oral sex followed by partner's insertive anal sex |
| 80.00 | 80.00 | 80.00 | %, proportion of insertive oral sex followed by partner's insertive rimming |
| 0.81 | 0.65 | 0.96 | %, Infection prevalence of chlamydia trachomatis only at Oropharynx |
| 1.83 | 1.63 | 2.23 | %, Infection prevalence of chlamydia trachomatis only at Urethral |
| 7.63 | 6.96 | 8.09 | %, Infection prevalence of chlamydia trachomatis only at Rectum |
| 0.00 | 0.00 | 0.00 | %, Infection prevalence of chlamydia trachomatis at Oropharynx & Urethra |
| 1.25 | 1.00 | 1.49 | %, Infection prevalence of chlamydia trachomatis at Oropharynx & Rectum |
| 1.30 | 1.03 | 1.52 | %, Infection prevalence of chlamydia trachomatis at Urethra & Rectum |
| 0.21 | 0.12 | 0.30 | %, Infection prevalence of chlamydia trachomatis at Oropharynx & Urethra & Rectum |

**Table S8. 45.** Model 8’ output parameters with 95% confidence intervals

| **Mean** | **95%CI: lower** | **95%CI: upper** | **Parameters** |
| --- | --- | --- | --- |
| 46.09 | 40.57 | 53.59 | %, Consistent condom usage in anal sex in past 12 months |
| 88.09 | 82.54 | 91.89 | %, Condom efficacy in preventing transmission |
| - | - | - | Times, Frequency of kissing in the past 12 months |
| 20.08 | 10.39 | 26.25 | Times, Frequency of oral sex in the past 12 months |
| 40.97 | 15.05 | 65.85 | Times, Frequency of rimming in the past 12 months |
| 13.19 | 4.58 | 20.54 | Times, Frequency of anal sex in the past 12 months |
| 118.70 | 62.39 | 198.08 | weeks, Infection duration of chlamydia trachomatis at throat (asymptomatic infection) |
| 1.39 | 1.14 | 1.79 | weeks, Infection duration at urethral (symptomatic infection) |
| 84.87 | 82.65 | 88.18 | %, proportion of urethral infections that are asymptomatic |
| 81.00 | 72.45 | 90.50 | %, proportion of anal infections that are asymptomatic |
| 15.53 | 7.60 | 27.93 | weeks, Infection duration at urethral (asymptomatic infection) |
| 49.52 | 48.69 | 51.08 | weeks, Infection duration of chlamydia trachomatis at anus (asymptomatic infection) |
| 1.57 | 1.25 | 1.93 | weeks, Infection duration of chlamydia trachomatis at anus (symptomatic infection) |
| 82.67 | 69.54 | 88.85 | %, proportion of MSM received throat swab in the past 12 months |
| 75.46 | 67.89 | 87.11 | %, proportion of MSM received anal swab in the past 12 months |
| 79.01 | 68.09 | 90.36 | %, proportion of MSM received urine test in the past 12 months |
| 28.58 | 26.69 | 32.41 | %, proportion of 'oral sex and anal sex' in the same sex episode |
| 71.44 | 70.46 | 72.52 | %, proportion of 'oral sex and rimming' in the same sex episode |
| 68.96 | 67.15 | 70.38 | %, proportion of saliva use during anal sex |
| 80.00 | 80.00 | 80.00 | %, proportion of receptive oral sex followed by partner's insertive anal sex |
| 80.00 | 80.00 | 80.00 | %, proportion of insertive oral sex followed by partner's insertive rimming |
| 0.83 | 0.65 | 0.95 | %, Infection prevalence of chlamydia trachomatis only at Oropharynx |
| 2.05 | 1.71 | 2.23 | %, Infection prevalence of chlamydia trachomatis only at Urethral |
| 7.57 | 6.99 | 7.95 | %, Infection prevalence of chlamydia trachomatis only at Rectum |
| 0.00 | 0.00 | 0.00 | %, Infection prevalence of chlamydia trachomatis at Oropharynx & Urethra |
| 1.24 | 1.04 | 1.43 | %, Infection prevalence of chlamydia trachomatis at Oropharynx & Rectum |
| 1.24 | 1.05 | 1.50 | %, Infection prevalence of chlamydia trachomatis at Urethra & Rectum |
| 0.20 | 0.13 | 0.28 | %, Infection prevalence of chlamydia trachomatis at Oropharynx & Urethra & Rectum |

**Table S8. 46.** Model 9’ output parameters with 95% confidence intervals

| **Mean** | **95%CI: lower** | **95%CI: upper** | **Parameters** |
| --- | --- | --- | --- |
| 47.17 | 41.79 | 53.03 | %, Consistent condom usage in anal sex in past 12 months |
| 87.02 | 83.59 | 91.03 | %, Condom efficacy in preventing transmission |
| - | - | - | Times, Frequency of kissing in the past 12 months |
| 18.37 | 12.09 | 25.58 | Times, Frequency of oral sex in the past 12 months |
| 34.58 | 19.81 | 59.34 | Times, Frequency of rimming in the past 12 months |
| 13.90 | 7.27 | 19.46 | Times, Frequency of anal sex in the past 12 months |
| 142.64 | 75.77 | 195.30 | weeks, Infection duration of chlamydia trachomatis at throat (asymptomatic infection) |
| 1.53 | 1.21 | 1.78 | weeks, Infection duration at urethral (symptomatic infection) |
| 85.81 | 82.72 | 87.96 | %, proportion of urethral infections that are asymptomatic |
| 81.04 | 73.17 | 88.09 | %, proportion of anal infections that are asymptomatic |
| 16.29 | 10.15 | 25.90 | weeks, Infection duration at urethral (asymptomatic infection) |
| 49.74 | 48.76 | 50.67 | weeks, Infection duration of chlamydia trachomatis at anus (asymptomatic infection) |
| 1.51 | 1.24 | 1.83 | weeks, Infection duration of chlamydia trachomatis at anus (symptomatic infection) |
| 77.58 | 71.17 | 87.69 | %, proportion of MSM received throat swab in the past 12 months |
| 79.09 | 67.90 | 86.56 | %, proportion of MSM received anal swab in the past 12 months |
| 81.67 | 69.72 | 89.35 | %, proportion of MSM received urine test in the past 12 months |
| 29.84 | 27.01 | 32.27 | %, proportion of 'oral sex and anal sex' in the same sex episode |
| 71.55 | 70.74 | 72.25 | %, proportion of 'oral sex and rimming' in the same sex episode |
| 69.00 | 67.20 | 70.28 | %, proportion of saliva use during anal sex |
| 80.00 | 80.00 | 80.00 | %, proportion of receptive oral sex followed by partner's insertive anal sex |
| 80.00 | 80.00 | 80.00 | %, proportion of insertive oral sex followed by partner's insertive rimming |
| 0.78 | 0.66 | 0.92 | %, Infection prevalence of chlamydia trachomatis only at Oropharynx |
| 1.99 | 1.74 | 2.22 | %, Infection prevalence of chlamydia trachomatis only at Urethral |
| 7.45 | 7.05 | 7.93 | %, Infection prevalence of chlamydia trachomatis only at Rectum |
| 0.00 | 0.00 | 0.00 | %, Infection prevalence of chlamydia trachomatis at Oropharynx & Urethra |
| 1.18 | 1.07 | 1.36 | %, Infection prevalence of chlamydia trachomatis at Oropharynx & Rectum |
| 1.26 | 1.06 | 1.47 | %, Infection prevalence of chlamydia trachomatis at Urethra & Rectum |
| 0.20 | 0.14 | 0.27 | %, Infection prevalence of chlamydia trachomatis at Oropharynx & Urethra & Rectum |

**Table S8. 47.** Model 10’ output parameters with 95% confidence intervals

| **Mean** | **95%CI: lower** | **95%CI: upper** | **Parameters** |
| --- | --- | --- | --- |
| 46.88 | 43.49 | 52.29 | %, Consistent condom usage in anal sex in past 12 months |
| 86.83 | 83.66 | 90.21 | %, Condom efficacy in preventing transmission |
| - | - | - | Times, Frequency of kissing in the past 12 months |
| 20.28 | 13.37 | 24.82 | Times, Frequency of oral sex in the past 12 months |
| 42.83 | 21.22 | 57.83 | Times, Frequency of rimming in the past 12 months |
| 12.83 | 7.31 | 19.12 | Times, Frequency of anal sex in the past 12 months |
| 139.78 | 77.90 | 190.34 | weeks, Infection duration of chlamydia trachomatis at throat (asymptomatic infection) |
| 1.47 | 1.27 | 1.73 | weeks, Infection duration at urethral (symptomatic infection) |
| 85.29 | 82.95 | 87.27 | %, proportion of urethral infections that are asymptomatic |
| 79.06 | 73.53 | 87.80 | %, proportion of anal infections that are asymptomatic |
| 17.54 | 10.50 | 24.25 | weeks, Infection duration at urethral (asymptomatic infection) |
| 49.71 | 48.81 | 50.45 | weeks, Infection duration of chlamydia trachomatis at anus (asymptomatic infection) |
| 1.53 | 1.29 | 1.79 | weeks, Infection duration of chlamydia trachomatis at anus (symptomatic infection) |
| 77.89 | 71.43 | 87.24 | %, proportion of MSM received throat swab in the past 12 months |
| 77.60 | 69.81 | 85.29 | %, proportion of MSM received anal swab in the past 12 months |
| 79.17 | 70.19 | 88.83 | %, proportion of MSM received urine test in the past 12 months |
| 29.69 | 27.33 | 32.15 | %, proportion of 'oral sex and anal sex' in the same sex episode |
| 71.42 | 70.78 | 72.16 | %, proportion of 'oral sex and rimming' in the same sex episode |
| 69.09 | 67.34 | 70.11 | %, proportion of saliva use during anal sex |
| 80.00 | 80.00 | 80.00 | %, proportion of receptive oral sex followed by partner's insertive anal sex |
| 80.00 | 80.00 | 80.00 | %, proportion of insertive oral sex followed by partner's insertive rimming |
| 0.77 | 0.66 | 0.90 | %, Infection prevalence of chlamydia trachomatis only at Oropharynx |
| 2.00 | 1.82 | 2.18 | %, Infection prevalence of chlamydia trachomatis only at Urethral |
| 7.55 | 7.07 | 7.91 | %, Infection prevalence of chlamydia trachomatis only at Rectum |
| 0.00 | 0.00 | 0.00 | %, Infection prevalence of chlamydia trachomatis at Oropharynx & Urethra |
| 1.20 | 1.09 | 1.34 | %, Infection prevalence of chlamydia trachomatis at Oropharynx & Rectum |
| 1.28 | 1.08 | 1.44 | %, Infection prevalence of chlamydia trachomatis at Urethra & Rectum |
| 0.19 | 0.14 | 0.24 | %, Infection prevalence of chlamydia trachomatis at Oropharynx & Urethra & Rectum |

**Table S8. 48.** Model 11’ output parameters with 95% confidence intervals

| **Mean** | **95%CI: lower** | **95%CI: upper** | **Parameters** |
| --- | --- | --- | --- |
| 47.90 | 43.65 | 51.78 | %, Consistent condom usage in anal sex in past 12 months |
| 87.21 | 83.82 | 90.01 | %, Condom efficacy in preventing transmission |
| - | - | - | Times, Frequency of kissing in the past 12 months |
| 18.81 | 14.28 | 24.89 | Times, Frequency of oral sex in the past 12 months |
| 41.23 | 23.91 | 57.13 | Times, Frequency of rimming in the past 12 months |
| 13.93 | 7.83 | 18.96 | Times, Frequency of anal sex in the past 12 months |
| 143.90 | 93.41 | 188.23 | weeks, Infection duration of chlamydia trachomatis at throat (asymptomatic infection) |
| 1.53 | 1.31 | 1.69 | weeks, Infection duration at urethral (symptomatic infection) |
| 85.33 | 83.15 | 87.03 | %, proportion of urethral infections that are asymptomatic |
| 82.23 | 74.30 | 87.30 | %, proportion of anal infections that are asymptomatic |
| 16.95 | 10.61 | 23.17 | weeks, Infection duration at urethral (asymptomatic infection) |
| 49.55 | 48.86 | 50.25 | weeks, Infection duration of chlamydia trachomatis at anus (asymptomatic infection) |
| 1.56 | 1.29 | 1.76 | weeks, Infection duration of chlamydia trachomatis at anus (symptomatic infection) |
| 78.94 | 72.09 | 85.64 | %, proportion of MSM received throat swab in the past 12 months |
| 78.87 | 70.06 | 84.87 | %, proportion of MSM received anal swab in the past 12 months |
| 79.58 | 71.12 | 88.29 | %, proportion of MSM received urine test in the past 12 months |
| 29.82 | 27.50 | 31.91 | %, proportion of 'oral sex and anal sex' in the same sex episode |
| 71.50 | 70.96 | 72.07 | %, proportion of 'oral sex and rimming' in the same sex episode |
| 68.45 | 67.35 | 69.98 | %, proportion of saliva use during anal sex |
| 80.00 | 80.00 | 80.00 | %, proportion of receptive oral sex followed by partner's insertive anal sex |
| 80.00 | 80.00 | 80.00 | %, proportion of insertive oral sex followed by partner's insertive rimming |
| 0.81 | 0.69 | 0.90 | %, Infection prevalence of chlamydia trachomatis only at Oropharynx |
| 1.97 | 1.82 | 2.17 | %, Infection prevalence of chlamydia trachomatis only at Urethral |
| 7.46 | 7.12 | 7.88 | %, Infection prevalence of chlamydia trachomatis only at Rectum |
| 0.00 | 0.00 | 0.00 | %, Infection prevalence of chlamydia trachomatis at Oropharynx & Urethra |
| 1.22 | 1.11 | 1.33 | %, Infection prevalence of chlamydia trachomatis at Oropharynx & Rectum |
| 1.23 | 1.08 | 1.40 | %, Infection prevalence of chlamydia trachomatis at Urethra & Rectum |
| 0.19 | 0.15 | 0.24 | %, Infection prevalence of chlamydia trachomatis at Oropharynx & Urethra & Rectum |

**Table S8. 49.** Model 12’ output parameters with 95% confidence intervals

| **Mean** | **95%CI: lower** | **95%CI: upper** | **Parameters** |
| --- | --- | --- | --- |
| 47.94 | 44.69 | 50.34 | %, Consistent condom usage in anal sex in past 12 months |
| 87.51 | 85.03 | 89.53 | %, Condom efficacy in preventing transmission |
| - | - | - | Times, Frequency of kissing in the past 12 months |
| 19.57 | 15.14 | 22.90 | Times, Frequency of oral sex in the past 12 months |
| 35.69 | 25.96 | 50.45 | Times, Frequency of rimming in the past 12 months |
| 15.24 | 9.59 | 18.08 | Times, Frequency of anal sex in the past 12 months |
| 139.72 | 107.96 | 176.64 | weeks, Infection duration of chlamydia trachomatis at throat (asymptomatic infection) |
| 1.54 | 1.34 | 1.63 | weeks, Infection duration at urethral (symptomatic infection) |
| 85.27 | 83.61 | 86.93 | %, proportion of urethral infections that are asymptomatic |
| 81.40 | 75.41 | 86.32 | %, proportion of anal infections that are asymptomatic |
| 15.45 | 11.10 | 20.98 | weeks, Infection duration at urethral (asymptomatic infection) |
| 49.43 | 48.88 | 50.03 | weeks, Infection duration of chlamydia trachomatis at anus (asymptomatic infection) |
| 1.59 | 1.39 | 1.69 | weeks, Infection duration of chlamydia trachomatis at anus (symptomatic infection) |
| 81.66 | 73.41 | 85.02 | %, proportion of MSM received throat swab in the past 12 months |
| 76.94 | 71.57 | 83.32 | %, proportion of MSM received anal swab in the past 12 months |
| 78.46 | 73.18 | 86.79 | %, proportion of MSM received urine test in the past 12 months |
| 29.83 | 28.26 | 31.71 | %, proportion of 'oral sex and anal sex' in the same sex episode |
| 71.56 | 71.05 | 71.97 | %, proportion of 'oral sex and rimming' in the same sex episode |
| 68.78 | 67.46 | 69.75 | %, proportion of saliva use during anal sex |
| 80.00 | 80.00 | 80.00 | %, proportion of receptive oral sex followed by partner's insertive anal sex |
| 80.00 | 80.00 | 80.00 | %, proportion of insertive oral sex followed by partner's insertive rimming |
| 0.82 | 0.72 | 0.88 | %, Infection prevalence of chlamydia trachomatis only at Oropharynx |
| 2.06 | 1.88 | 2.15 | %, Infection prevalence of chlamydia trachomatis only at Urethral |
| 7.58 | 7.16 | 7.83 | %, Infection prevalence of chlamydia trachomatis only at Rectum |
| 0.00 | 0.00 | 0.00 | %, Infection prevalence of chlamydia trachomatis at Oropharynx & Urethra |
| 1.21 | 1.14 | 1.28 | %, Infection prevalence of chlamydia trachomatis at Oropharynx & Rectum |
| 1.19 | 1.09 | 1.36 | %, Infection prevalence of chlamydia trachomatis at Urethra & Rectum |
| 0.18 | 0.16 | 0.22 | %, Infection prevalence of chlamydia trachomatis at Oropharynx & Urethra & Rectum |

**Table S8. 50.** Model 13’ output parameters with 95% confidence intervals

| **Mean** | **95%CI: lower** | **95%CI: upper** | **Parameters** |
| --- | --- | --- | --- |
| 46.84 | 45.22 | 49.94 | %, Consistent condom usage in anal sex in past 12 months |
| 86.89 | 85.14 | 89.09 | %, Condom efficacy in preventing transmission |
| - | - | - | Times, Frequency of kissing in the past 12 months |
| 18.35 | 15.32 | 22.19 | Times, Frequency of oral sex in the past 12 months |
| 36.21 | 28.00 | 46.35 | Times, Frequency of rimming in the past 12 months |
| 15.61 | 10.72 | 18.09 | Times, Frequency of anal sex in the past 12 months |
| 136.92 | 110.11 | 174.56 | weeks, Infection duration of chlamydia trachomatis at throat (asymptomatic infection) |
| 1.50 | 1.37 | 1.61 | weeks, Infection duration at urethral (symptomatic infection) |
| 85.75 | 83.65 | 86.86 | %, proportion of urethral infections that are asymptomatic |
| 79.47 | 75.72 | 86.35 | %, proportion of anal infections that are asymptomatic |
| 17.41 | 11.55 | 20.56 | weeks, Infection duration at urethral (asymptomatic infection) |
| 49.35 | 48.94 | 50.03 | weeks, Infection duration of chlamydia trachomatis at anus (asymptomatic infection) |
| 1.59 | 1.39 | 1.68 | weeks, Infection duration of chlamydia trachomatis at anus (symptomatic infection) |
| 80.21 | 73.31 | 84.65 | %, proportion of MSM received throat swab in the past 12 months |
| 78.12 | 72.28 | 83.12 | %, proportion of MSM received anal swab in the past 12 months |
| 78.95 | 73.68 | 86.04 | %, proportion of MSM received urine test in the past 12 months |
| 30.45 | 28.72 | 31.58 | %, proportion of 'oral sex and anal sex' in the same sex episode |
| 71.45 | 71.09 | 71.92 | %, proportion of 'oral sex and rimming' in the same sex episode |
| 68.40 | 67.54 | 69.52 | %, proportion of saliva use during anal sex |
| 80.00 | 80.00 | 80.00 | %, proportion of receptive oral sex followed by partner's insertive anal sex |
| 80.00 | 80.00 | 80.00 | %, proportion of insertive oral sex followed by partner's insertive rimming |
| 0.81 | 0.73 | 0.88 | %, Infection prevalence of chlamydia trachomatis only at Oropharynx |
| 2.01 | 1.90 | 2.13 | %, Infection prevalence of chlamydia trachomatis only at Urethral |
| 7.59 | 7.23 | 7.82 | %, Infection prevalence of chlamydia trachomatis only at Rectum |
| 0.00 | 0.00 | 0.00 | %, Infection prevalence of chlamydia trachomatis at Oropharynx & Urethra |
| 1.20 | 1.14 | 1.27 | %, Infection prevalence of chlamydia trachomatis at Oropharynx & Rectum |
| 1.24 | 1.13 | 1.33 | %, Infection prevalence of chlamydia trachomatis at Urethra & Rectum |
| 0.19 | 0.16 | 0.22 | %, Infection prevalence of chlamydia trachomatis at Oropharynx & Urethra & Rectum |

Model output parameters (Published validation dataset 1): Data from 1,011 asymptomatic MSM attending Melbourne Sexual Health Centre

**Table S8. 51.** Model 2’ output parameters with 95% confidence intervals

| **Mean** | **95%CI: lower** | **95%CI: upper** | **Parameters** |
| --- | --- | --- | --- |
| 46.44 | 35.20 | 56.27 | %, Consistent condom usage in anal sex in past 12 months |
| 88.68 | 80.42 | 94.51 | %, Condom efficacy in preventing transmission |
| - | - | - | Times, Frequency of kissing in the past 12 months |
| 17.36 | 5.55 | 27.42 | Times, Frequency of oral sex in the past 12 months |
| 36.93 | 1.70 | 77.84 | Times, Frequency of rimming in the past 12 months |
| 20.02 | 5.12 | 47.66 | Times, Frequency of anal sex in the past 12 months |
| 146.01 | 35.86 | 235.71 | weeks, Infection duration of chlamydia trachomatis at throat (asymptomatic infection) |
| 1.59 | 1.09 | 1.91 | weeks, Infection duration at urethral (symptomatic infection) |
| 84.02 | 81.26 | 88.83 | %, proportion of urethral infections that are asymptomatic |
| 81.25 | 63.24 | 96.84 | %, proportion of anal infections that are asymptomatic |
| 24.12 | 7.11 | 45.96 | weeks, Infection duration at urethral (asymptomatic infection) |
| 49.85 | 48.16 | 51.77 | weeks, Infection duration of chlamydia trachomatis at anus (asymptomatic infection) |
| 1.60 | 1.13 | 1.95 | weeks, Infection duration of chlamydia trachomatis at anus (symptomatic infection) |
| 84.97 | 68.72 | 93.50 | %, proportion of MSM received throat swab in the past 12 months |
| 78.46 | 64.79 | 93.84 | %, proportion of MSM received anal swab in the past 12 months |
| 77.92 | 65.79 | 90.93 | %, proportion of MSM received urine test in the past 12 months |
| 28.88 | 25.16 | 33.25 | %, proportion of 'oral sex and anal sex' in the same sex episode |
| 71.77 | 69.93 | 72.60 | %, proportion of 'oral sex and rimming' in the same sex episode |
| 69.01 | 66.67 | 70.74 | %, proportion of saliva use during anal sex |
| 80.00 | 80.00 | 80.00 | %, proportion of receptive oral sex followed by partner's insertive anal sex |
| 80.00 | 80.00 | 80.00 | %, proportion of insertive oral sex followed by partner's insertive rimming |
| 1.67 | 0.36 | 2.51 | %, Infection prevalence of chlamydia trachomatis only at Oropharynx |
| 1.48 | 0.76 | 2.25 | %, Infection prevalence of chlamydia trachomatis only at Urethral |
| 6.99 | 5.76 | 8.67 | %, Infection prevalence of chlamydia trachomatis only at Rectum |
| 0.00 | 0.00 | 0.00 | %, Infection prevalence of chlamydia trachomatis at Oropharynx & Urethra |
| 2.24 | 1.12 | 3.69 | %, Infection prevalence of chlamydia trachomatis at Oropharynx & Rectum |
| 0.66 | 0.19 | 1.12 | %, Infection prevalence of chlamydia trachomatis at Urethra & Rectum |
| 0.00 | 0.00 | 0.00 | %, Infection prevalence of chlamydia trachomatis at Oropharynx & Urethra & Rectum |

**Table S8. 52.** Model 7’ output parameters with 95% confidence intervals

| **Mean** | **95%CI: lower** | **95%CI: upper** | **Parameters** |
| --- | --- | --- | --- |
| 42.64 | 35.98 | 54.12 | %, Consistent condom usage in anal sex in past 12 months |
| 86.48 | 81.23 | 92.64 | %, Condom efficacy in preventing transmission |
| - | - | - | Times, Frequency of kissing in the past 12 months |
| 16.34 | 8.72 | 24.12 | Times, Frequency of oral sex in the past 12 months |
| 54.66 | 20.15 | 76.80 | Times, Frequency of rimming in the past 12 months |
| 14.02 | 5.82 | 27.50 | Times, Frequency of anal sex in the past 12 months |
| 131.03 | 51.07 | 227.86 | weeks, Infection duration of chlamydia trachomatis at throat (asymptomatic infection) |
| 1.44 | 1.12 | 1.72 | weeks, Infection duration at urethral (symptomatic infection) |
| 84.70 | 81.78 | 88.40 | %, proportion of urethral infections that are asymptomatic |
| 81.82 | 65.74 | 94.37 | %, proportion of anal infections that are asymptomatic |
| 24.41 | 11.10 | 44.88 | weeks, Infection duration at urethral (asymptomatic infection) |
| 50.12 | 48.54 | 51.51 | weeks, Infection duration of chlamydia trachomatis at anus (asymptomatic infection) |
| 1.47 | 1.21 | 1.89 | weeks, Infection duration of chlamydia trachomatis at anus (symptomatic infection) |
| 81.40 | 70.33 | 92.87 | %, proportion of MSM received throat swab in the past 12 months |
| 77.47 | 67.82 | 90.61 | %, proportion of MSM received anal swab in the past 12 months |
| 76.29 | 66.90 | 87.42 | %, proportion of MSM received urine test in the past 12 months |
| 29.66 | 26.65 | 32.57 | %, proportion of 'oral sex and anal sex' in the same sex episode |
| 71.48 | 70.36 | 72.45 | %, proportion of 'oral sex and rimming' in the same sex episode |
| 68.37 | 67.18 | 70.15 | %, proportion of saliva use during anal sex |
| 80.00 | 80.00 | 80.00 | %, proportion of receptive oral sex followed by partner's insertive anal sex |
| 80.00 | 80.00 | 80.00 | %, proportion of insertive oral sex followed by partner's insertive rimming |
| 1.41 | 0.43 | 2.33 | %, Infection prevalence of chlamydia trachomatis only at Oropharynx |
| 1.45 | 0.83 | 2.12 | %, Infection prevalence of chlamydia trachomatis only at Urethral |
| 6.99 | 5.90 | 8.44 | %, Infection prevalence of chlamydia trachomatis only at Rectum |
| 0.00 | 0.00 | 0.00 | %, Infection prevalence of chlamydia trachomatis at Oropharynx & Urethra |
| 2.79 | 1.46 | 3.66 | %, Infection prevalence of chlamydia trachomatis at Oropharynx & Rectum |
| 0.72 | 0.33 | 1.09 | %, Infection prevalence of chlamydia trachomatis at Urethra & Rectum |
| 0.00 | 0.00 | 0.00 | %, Infection prevalence of chlamydia trachomatis at Oropharynx & Urethra & Rectum |

**Table S8. 53.** Model 8’ output parameters with 95% confidence intervals

| **Mean** | **95%CI: lower** | **95%CI: upper** | **Parameters** |
| --- | --- | --- | --- |
| 46.26 | 38.44 | 51.98 | %, Consistent condom usage in anal sex in past 12 months |
| 87.81 | 82.45 | 92.00 | %, Condom efficacy in preventing transmission |
| - | - | - | Times, Frequency of kissing in the past 12 months |
| 18.90 | 14.67 | 23.04 | Times, Frequency of oral sex in the past 12 months |
| 50.99 | 26.05 | 72.43 | Times, Frequency of rimming in the past 12 months |
| 19.87 | 11.60 | 26.57 | Times, Frequency of anal sex in the past 12 months |
| 105.78 | 55.38 | 208.31 | weeks, Infection duration of chlamydia trachomatis at throat (asymptomatic infection) |
| 1.41 | 1.18 | 1.61 | weeks, Infection duration at urethral (symptomatic infection) |
| 84.58 | 82.58 | 86.73 | %, proportion of urethral infections that are asymptomatic |
| 78.39 | 68.53 | 92.85 | %, proportion of anal infections that are asymptomatic |
| 16.57 | 12.90 | 29.76 | weeks, Infection duration at urethral (asymptomatic infection) |
| 50.04 | 48.93 | 50.88 | weeks, Infection duration of chlamydia trachomatis at anus (asymptomatic infection) |
| 1.63 | 1.44 | 1.78 | weeks, Infection duration of chlamydia trachomatis at anus (symptomatic infection) |
| 82.81 | 72.71 | 89.99 | %, proportion of MSM received throat swab in the past 12 months |
| 80.67 | 70.06 | 90.16 | %, proportion of MSM received anal swab in the past 12 months |
| 81.72 | 71.73 | 85.50 | %, proportion of MSM received urine test in the past 12 months |
| 29.87 | 27.08 | 32.22 | %, proportion of 'oral sex and anal sex' in the same sex episode |
| 71.52 | 70.60 | 72.21 | %, proportion of 'oral sex and rimming' in the same sex episode |
| 68.74 | 67.42 | 69.74 | %, proportion of saliva use during anal sex |
| 80.00 | 80.00 | 80.00 | %, proportion of receptive oral sex followed by partner's insertive anal sex |
| 80.00 | 80.00 | 80.00 | %, proportion of insertive oral sex followed by partner's insertive rimming |
| 1.32 | 0.61 | 2.15 | %, Infection prevalence of chlamydia trachomatis only at Oropharynx |
| 1.57 | 1.02 | 1.95 | %, Infection prevalence of chlamydia trachomatis only at Urethral |
| 7.34 | 6.24 | 8.13 | %, Infection prevalence of chlamydia trachomatis only at Rectum |
| 0.00 | 0.00 | 0.00 | %, Infection prevalence of chlamydia trachomatis at Oropharynx & Urethra |
| 2.68 | 2.02 | 3.38 | %, Infection prevalence of chlamydia trachomatis at Oropharynx & Rectum |
| 0.71 | 0.48 | 1.04 | %, Infection prevalence of chlamydia trachomatis at Urethra & Rectum |
| 0.00 | 0.00 | 0.00 | %, Infection prevalence of chlamydia trachomatis at Oropharynx & Urethra & Rectum |

**Table S8. 54.** Model 9’ output parameters with 95% confidence intervals

| **Mean** | **95%CI: lower** | **95%CI: upper** | **Parameters** |
| --- | --- | --- | --- |
| 45.19 | 38.05 | 56.76 | %, Consistent condom usage in anal sex in past 12 months |
| 89.05 | 81.23 | 94.41 | %, Condom efficacy in preventing transmission |
| - | - | - | Times, Frequency of kissing in the past 12 months |
| 17.36 | 5.15 | 26.51 | Times, Frequency of oral sex in the past 12 months |
| 31.02 | 3.38 | 74.71 | Times, Frequency of rimming in the past 12 months |
| 24.17 | 3.70 | 50.11 | Times, Frequency of anal sex in the past 12 months |
| 157.57 | 68.02 | 240.09 | weeks, Infection duration of chlamydia trachomatis at throat (asymptomatic infection) |
| 1.51 | 1.06 | 1.95 | weeks, Infection duration at urethral (symptomatic infection) |
| 85.58 | 81.03 | 89.36 | %, proportion of urethral infections that are asymptomatic |
| 80.38 | 68.33 | 95.05 | %, proportion of anal infections that are asymptomatic |
| 21.90 | 4.34 | 50.36 | weeks, Infection duration at urethral (asymptomatic infection) |
| 49.36 | 48.12 | 51.19 | weeks, Infection duration of chlamydia trachomatis at anus (asymptomatic infection) |
| 1.38 | 1.05 | 1.85 | weeks, Infection duration of chlamydia trachomatis at anus (symptomatic infection) |
| 80.31 | 70.88 | 92.55 | %, proportion of MSM received throat swab in the past 12 months |
| 75.84 | 64.55 | 90.64 | %, proportion of MSM received anal swab in the past 12 months |
| 80.84 | 67.66 | 92.53 | %, proportion of MSM received urine test in the past 12 months |
| 28.01 | 25.18 | 33.23 | %, proportion of 'oral sex and anal sex' in the same sex episode |
| 71.31 | 70.13 | 72.40 | %, proportion of 'oral sex and rimming' in the same sex episode |
| 69.23 | 66.14 | 70.72 | %, proportion of saliva use during anal sex |
| 80.00 | 80.00 | 80.00 | %, proportion of receptive oral sex followed by partner's insertive anal sex |
| 80.00 | 80.00 | 80.00 | %, proportion of insertive oral sex followed by partner's insertive rimming |
| 1.56 | 0.50 | 2.50 | %, Infection prevalence of chlamydia trachomatis only at Oropharynx |
| 1.66 | 0.91 | 2.24 | %, Infection prevalence of chlamydia trachomatis only at Urethral |
| 8.04 | 5.88 | 8.84 | %, Infection prevalence of chlamydia trachomatis only at Rectum |
| 0.00 | 0.00 | 0.00 | %, Infection prevalence of chlamydia trachomatis at Oropharynx & Urethra |
| 2.17 | 1.11 | 3.70 | %, Infection prevalence of chlamydia trachomatis at Oropharynx & Rectum |
| 0.59 | 0.26 | 1.07 | %, Infection prevalence of chlamydia trachomatis at Urethra & Rectum |
| 0.00 | 0.00 | 0.00 | %, Infection prevalence of chlamydia trachomatis at Oropharynx & Urethra & Rectum |

**Table S8. 55.** Model 10’ output parameters with 95% confidence intervals

| **Mean** | **95%CI: lower** | **95%CI: upper** | **Parameters** |
| --- | --- | --- | --- |
| 45.20 | 39.44 | 54.00 | %, Consistent condom usage in anal sex in past 12 months |
| 88.67 | 81.67 | 94.06 | %, Condom efficacy in preventing transmission |
| - | - | - | Times, Frequency of kissing in the past 12 months |
| 15.96 | 6.60 | 25.85 | Times, Frequency of oral sex in the past 12 months |
| 40.44 | 14.39 | 72.40 | Times, Frequency of rimming in the past 12 months |
| 27.28 | 8.60 | 48.95 | Times, Frequency of anal sex in the past 12 months |
| 165.26 | 78.89 | 224.57 | weeks, Infection duration of chlamydia trachomatis at throat (asymptomatic infection) |
| 1.47 | 1.09 | 1.86 | weeks, Infection duration at urethral (symptomatic infection) |
| 86.16 | 81.56 | 89.01 | %, proportion of urethral infections that are asymptomatic |
| 85.02 | 69.52 | 94.33 | %, proportion of anal infections that are asymptomatic |
| 25.92 | 6.31 | 46.26 | weeks, Infection duration at urethral (asymptomatic infection) |
| 50.20 | 48.73 | 50.97 | weeks, Infection duration of chlamydia trachomatis at anus (asymptomatic infection) |
| 1.40 | 1.11 | 1.84 | weeks, Infection duration of chlamydia trachomatis at anus (symptomatic infection) |
| 82.06 | 72.62 | 91.62 | %, proportion of MSM received throat swab in the past 12 months |
| 79.20 | 65.49 | 89.62 | %, proportion of MSM received anal swab in the past 12 months |
| 80.47 | 67.93 | 91.77 | %, proportion of MSM received urine test in the past 12 months |
| 29.61 | 26.38 | 32.74 | %, proportion of 'oral sex and anal sex' in the same sex episode |
| 71.52 | 70.30 | 72.30 | %, proportion of 'oral sex and rimming' in the same sex episode |
| 68.56 | 66.34 | 70.69 | %, proportion of saliva use during anal sex |
| 80.00 | 80.00 | 80.00 | %, proportion of receptive oral sex followed by partner's insertive anal sex |
| 80.00 | 80.00 | 80.00 | %, proportion of insertive oral sex followed by partner's insertive rimming |
| 1.29 | 0.53 | 2.34 | %, Infection prevalence of chlamydia trachomatis only at Oropharynx |
| 1.66 | 0.98 | 2.08 | %, Infection prevalence of chlamydia trachomatis only at Urethral |
| 7.04 | 5.94 | 8.24 | %, Infection prevalence of chlamydia trachomatis only at Rectum |
| 0.00 | 0.00 | 0.00 | %, Infection prevalence of chlamydia trachomatis at Oropharynx & Urethra |
| 2.51 | 1.20 | 3.59 | %, Infection prevalence of chlamydia trachomatis at Oropharynx & Rectum |
| 0.61 | 0.27 | 1.03 | %, Infection prevalence of chlamydia trachomatis at Urethra & Rectum |
| 0.00 | 0.00 | 0.00 | %, Infection prevalence of chlamydia trachomatis at Oropharynx & Urethra & Rectum |

**Table S8. 56.** Model 11’ output parameters with 95% confidence intervals

| **Mean** | **95%CI: lower** | **95%CI: upper** | **Parameters** |
| --- | --- | --- | --- |
| 46.20 | 40.78 | 52.73 | %, Consistent condom usage in anal sex in past 12 months |
| 87.80 | 82.19 | 92.47 | %, Condom efficacy in preventing transmission |
| - | - | - | Times, Frequency of kissing in the past 12 months |
| 16.80 | 7.15 | 24.13 | Times, Frequency of oral sex in the past 12 months |
| 45.96 | 17.91 | 72.42 | Times, Frequency of rimming in the past 12 months |
| 16.12 | 8.44 | 30.55 | Times, Frequency of anal sex in the past 12 months |
| 154.17 | 99.76 | 213.26 | weeks, Infection duration of chlamydia trachomatis at throat (asymptomatic infection) |
| 1.34 | 1.10 | 1.80 | weeks, Infection duration at urethral (symptomatic infection) |
| 84.72 | 81.97 | 88.65 | %, proportion of urethral infections that are asymptomatic |
| 79.01 | 69.99 | 92.81 | %, proportion of anal infections that are asymptomatic |
| 24.99 | 10.70 | 45.46 | weeks, Infection duration at urethral (asymptomatic infection) |
| 50.02 | 48.94 | 50.92 | weeks, Infection duration of chlamydia trachomatis at anus (asymptomatic infection) |
| 1.40 | 1.13 | 1.79 | weeks, Infection duration of chlamydia trachomatis at anus (symptomatic infection) |
| 80.38 | 73.63 | 89.93 | %, proportion of MSM received throat swab in the past 12 months |
| 77.46 | 66.96 | 87.49 | %, proportion of MSM received anal swab in the past 12 months |
| 80.11 | 69.66 | 90.08 | %, proportion of MSM received urine test in the past 12 months |
| 29.58 | 26.50 | 32.36 | %, proportion of 'oral sex and anal sex' in the same sex episode |
| 71.40 | 70.47 | 72.27 | %, proportion of 'oral sex and rimming' in the same sex episode |
| 68.52 | 66.71 | 70.56 | %, proportion of saliva use during anal sex |
| 80.00 | 80.00 | 80.00 | %, proportion of receptive oral sex followed by partner's insertive anal sex |
| 80.00 | 80.00 | 80.00 | %, proportion of insertive oral sex followed by partner's insertive rimming |
| 1.37 | 0.60 | 2.10 | %, Infection prevalence of chlamydia trachomatis only at Oropharynx |
| 1.51 | 0.99 | 1.95 | %, Infection prevalence of chlamydia trachomatis only at Urethral |
| 7.10 | 6.03 | 8.13 | %, Infection prevalence of chlamydia trachomatis only at Rectum |
| 0.00 | 0.00 | 0.00 | %, Infection prevalence of chlamydia trachomatis at Oropharynx & Urethra |
| 2.54 | 1.32 | 3.55 | %, Infection prevalence of chlamydia trachomatis at Oropharynx & Rectum |
| 0.65 | 0.37 | 1.03 | %, Infection prevalence of chlamydia trachomatis at Urethra & Rectum |
| 0.00 | 0.00 | 0.00 | %, Infection prevalence of chlamydia trachomatis at Oropharynx & Urethra & Rectum |

**Table S8. 57.** Model 12’ output parameters with 95% confidence intervals

| **Mean** | **95%CI: lower** | **95%CI: upper** | **Parameters** |
| --- | --- | --- | --- |
| 46.81 | 42.06 | 52.16 | %, Consistent condom usage in anal sex in past 12 months |
| 88.20 | 82.51 | 92.14 | %, Condom efficacy in preventing transmission |
| - | - | - | Times, Frequency of kissing in the past 12 months |
| 17.85 | 8.41 | 23.29 | Times, Frequency of oral sex in the past 12 months |
| 40.13 | 18.03 | 64.12 | Times, Frequency of rimming in the past 12 months |
| 24.56 | 9.90 | 30.03 | Times, Frequency of anal sex in the past 12 months |
| 162.15 | 106.46 | 210.81 | weeks, Infection duration of chlamydia trachomatis at throat (asymptomatic infection) |
| 1.48 | 1.13 | 1.76 | weeks, Infection duration at urethral (symptomatic infection) |
| 84.63 | 82.33 | 88.17 | %, proportion of urethral infections that are asymptomatic |
| 82.62 | 71.76 | 91.14 | %, proportion of anal infections that are asymptomatic |
| 16.13 | 11.11 | 23.32 | weeks, Infection duration at urethral (asymptomatic infection) |
| 49.74 | 49.04 | 50.85 | weeks, Infection duration of chlamydia trachomatis at anus (asymptomatic infection) |
| 1.55 | 1.15 | 1.79 | weeks, Infection duration of chlamydia trachomatis at anus (symptomatic infection) |
| 82.06 | 74.02 | 89.22 | %, proportion of MSM received throat swab in the past 12 months |
| 75.71 | 67.26 | 86.84 | %, proportion of MSM received anal swab in the past 12 months |
| 77.10 | 70.18 | 89.03 | %, proportion of MSM received urine test in the past 12 months |
| 29.55 | 26.82 | 32.29 | %, proportion of 'oral sex and anal sex' in the same sex episode |
| 71.29 | 70.62 | 72.05 | %, proportion of 'oral sex and rimming' in the same sex episode |
| 68.68 | 66.91 | 70.17 | %, proportion of saliva use during anal sex |
| 80.00 | 80.00 | 80.00 | %, proportion of receptive oral sex followed by partner's insertive anal sex |
| 80.00 | 80.00 | 80.00 | %, proportion of insertive oral sex followed by partner's insertive rimming |
| 1.61 | 0.68 | 2.02 | %, Infection prevalence of chlamydia trachomatis only at Oropharynx |
| 1.56 | 1.01 | 1.93 | %, Infection prevalence of chlamydia trachomatis only at Urethral |
| 7.23 | 6.20 | 8.10 | %, Infection prevalence of chlamydia trachomatis only at Rectum |
| 0.00 | 0.00 | 0.00 | %, Infection prevalence of chlamydia trachomatis at Oropharynx & Urethra |
| 2.39 | 1.46 | 3.43 | %, Infection prevalence of chlamydia trachomatis at Oropharynx & Rectum |
| 0.66 | 0.43 | 1.01 | %, Infection prevalence of chlamydia trachomatis at Urethra & Rectum |
| 0.00 | 0.00 | 0.00 | %, Infection prevalence of chlamydia trachomatis at Oropharynx & Urethra & Rectum |

**Table S8. 58.** Model 13’ output parameters with 95% confidence intervals

| **Mean** | **95%CI: lower** | **95%CI: upper** | **Parameters** |
| --- | --- | --- | --- |
| 47.01 | 42.31 | 51.79 | %, Consistent condom usage in anal sex in past 12 months |
| 87.20 | 82.92 | 91.12 | %, Condom efficacy in preventing transmission |
| - | - | - | Times, Frequency of kissing in the past 12 months |
| 15.39 | 9.20 | 21.59 | Times, Frequency of oral sex in the past 12 months |
| 43.90 | 20.52 | 58.98 | Times, Frequency of rimming in the past 12 months |
| 20.19 | 13.51 | 28.81 | Times, Frequency of anal sex in the past 12 months |
| 161.33 | 124.68 | 196.08 | weeks, Infection duration of chlamydia trachomatis at throat (asymptomatic infection) |
| 1.41 | 1.20 | 1.71 | weeks, Infection duration at urethral (symptomatic infection) |
| 85.25 | 82.53 | 87.47 | %, proportion of urethral infections that are asymptomatic |
| 80.03 | 73.24 | 88.56 | %, proportion of anal infections that are asymptomatic |
| 16.05 | 11.41 | 21.83 | weeks, Infection duration at urethral (asymptomatic infection) |
| 50.05 | 49.22 | 50.68 | weeks, Infection duration of chlamydia trachomatis at anus (asymptomatic infection) |
| 1.45 | 1.24 | 1.74 | weeks, Infection duration of chlamydia trachomatis at anus (symptomatic infection) |
| 82.14 | 75.87 | 87.74 | %, proportion of MSM received throat swab in the past 12 months |
| 76.08 | 68.03 | 86.58 | %, proportion of MSM received anal swab in the past 12 months |
| 79.51 | 71.38 | 88.65 | %, proportion of MSM received urine test in the past 12 months |
| 29.96 | 27.38 | 32.04 | %, proportion of 'oral sex and anal sex' in the same sex episode |
| 71.33 | 70.64 | 71.99 | %, proportion of 'oral sex and rimming' in the same sex episode |
| 68.59 | 67.24 | 69.94 | %, proportion of saliva use during anal sex |
| 80.00 | 80.00 | 80.00 | %, proportion of receptive oral sex followed by partner's insertive anal sex |
| 80.00 | 80.00 | 80.00 | %, proportion of insertive oral sex followed by partner's insertive rimming |
| 1.23 | 0.81 | 1.85 | %, Infection prevalence of chlamydia trachomatis only at Oropharynx |
| 1.49 | 1.03 | 1.86 | %, Infection prevalence of chlamydia trachomatis only at Urethral |
| 7.35 | 6.46 | 7.99 | %, Infection prevalence of chlamydia trachomatis only at Rectum |
| 0.00 | 0.00 | 0.00 | %, Infection prevalence of chlamydia trachomatis at Oropharynx & Urethra |
| 2.59 | 1.65 | 3.37 | %, Infection prevalence of chlamydia trachomatis at Oropharynx & Rectum |
| 0.78 | 0.50 | 0.98 | %, Infection prevalence of chlamydia trachomatis at Urethra & Rectum |
| 0.00 | 0.00 | 0.00 | %, Infection prevalence of chlamydia trachomatis at Oropharynx & Urethra & Rectum |

Model output parameters (Published validation dataset2): Data from 393 MSM attending STD & HIV care clinics in the USA

**Table S8. 59.** Model 2’ output parameters with 95% confidence intervals

| **Mean** | **95%CI: lower** | **95%CI: upper** | **Parameters** |
| --- | --- | --- | --- |
| 44.71 | 36.92 | 57.55 | %, Consistent condom usage in anal sex in past 12 months |
| 88.49 | 81.87 | 93.32 | %, Condom efficacy in preventing transmission |
| - | - | - | Times, Frequency of kissing in the past 12 months |
| 11.31 | 1.88 | 25.31 | Times, Frequency of oral sex in the past 12 months |
| 45.50 | 9.10 | 78.05 | Times, Frequency of rimming in the past 12 months |
| 15.34 | 1.63 | 48.13 | Times, Frequency of anal sex in the past 12 months |
| 132.30 | 31.94 | 219.48 | weeks, Infection duration of chlamydia trachomatis at throat(asymptomatic infection) |
| 1.51 | 1.09 | 1.92 | weeks, Infection duration at urethral (symptomatic infection) |
| 85.06 | 81.84 | 89.91 | %, proportion of urethral infections that are asymptomatic |
| 75.53 | 65.81 | 96.28 | %, proportion of anal infections that are asymptomatic |
| 34.35 | 7.19 | 51.79 | weeks, Infection duration at urethral (asymptomatic infection) |
| 50.10 | 48.16 | 51.92 | weeks, Infection duration of chlamydia trachomatis at anus (asymptomatic infection) |
| 1.44 | 1.06 | 1.94 | weeks, Infection duration of chlamydia trachomatis at anus (symptomatic infection) |
| 82.39 | 64.74 | 95.06 | %, proportion of MSM received throat swab in the past 12 months |
| 79.20 | 66.13 | 92.28 | %, proportion of MSM received anal swab in the past 12 months |
| 76.43 | 65.79 | 95.09 | %, proportion of MSM received urine test in the past 12 months |
| 29.22 | 25.36 | 33.37 | %, proportion of 'oral sex and anal sex' in the same sex episode |
| 71.37 | 69.90 | 72.73 | %, proportion of 'oral sex and rimming' in the same sex episode |
| 68.13 | 66.21 | 70.70 | %, proportion of saliva use during anal sex |
| 80.00 | 80.00 | 80.00 | %, proportion of receptive oral sex followed by partner's insertive anal sex |
| 80.00 | 80.00 | 80.00 | %, proportion of insertive oral sex followed by partner's insertive rimming |
| 1.08 | 0.29 | 1.84 | %, Infection prevalence of chlamydia trachomatis only at Oropharynx |
| 2.80 | 1.23 | 4.25 | %, Infection prevalence of chlamydia trachomatis only at Urethral |
| 6.94 | 4.83 | 8.85 | %, Infection prevalence of chlamydia trachomatis only at Rectum |
| 0.00 | 0.00 | 0.00 | %, Infection prevalence of chlamydia trachomatis at Oropharynx & Urethra |
| 1.92 | 0.70 | 3.28 | %, Infection prevalence of chlamydia trachomatis at Oropharynx & Rectum |
| 1.13 | 0.39 | 2.22 | %, Infection prevalence of chlamydia trachomatis at Urethra & Rectum |
| 0.00 | 0.00 | 0.00 | %, Infection prevalence of chlamydia trachomatis at Oropharynx & Urethra & Rectum |

**Table S8. 60.** Model 7’ output parameters with 95% confidence intervals

| **Mean** | **95%CI: lower** | **95%CI: upper** | **Parameters** |
| --- | --- | --- | --- |
| 46.25 | 38.07 | 56.82 | %, Consistent condom usage in anal sex in past 12 months |
| 87.02 | 82.60 | 91.87 | %, Condom efficacy in preventing transmission |
| - | - | - | Times, Frequency of kissing in the past 12 months |
| 10.59 | 4.72 | 23.18 | Times, Frequency of oral sex in the past 12 months |
| 47.50 | 16.88 | 74.43 | Times, Frequency of rimming in the past 12 months |
| 13.44 | 2.73 | 40.60 | Times, Frequency of anal sex in the past 12 months |
| 134.01 | 50.93 | 210.14 | weeks, Infection duration of chlamydia trachomatis at throat(asymptomatic infection) |
| 1.44 | 1.17 | 1.78 | weeks, Infection duration at urethral (symptomatic infection) |
| 86.44 | 83.07 | 89.65 | %, proportion of urethral infections that are asymptomatic |
| 86.30 | 69.45 | 95.70 | %, proportion of anal infections that are asymptomatic |
| 28.44 | 8.30 | 48.93 | weeks, Infection duration at urethral (asymptomatic infection) |
| 50.31 | 48.25 | 51.51 | weeks, Infection duration of chlamydia trachomatis at anus (asymptomatic infection) |
| 1.41 | 1.10 | 1.85 | weeks, Infection duration of chlamydia trachomatis at anus (symptomatic infection) |
| 74.46 | 65.71 | 89.39 | %, proportion of MSM received throat swab in the past 12 months |
| 72.49 | 67.05 | 81.87 | %, proportion of MSM received anal swab in the past 12 months |
| 79.98 | 67.99 | 93.47 | %, proportion of MSM received urine test in the past 12 months |
| 28.67 | 25.62 | 32.72 | %, proportion of 'oral sex and anal sex' in the same sex episode |
| 71.55 | 70.27 | 72.57 | %, proportion of 'oral sex and rimming' in the same sex episode |
| 68.45 | 67.17 | 70.55 | %, proportion of saliva use during anal sex |
| 80.00 | 80.00 | 80.00 | %, proportion of receptive oral sex followed by partner's insertive anal sex |
| 80.00 | 80.00 | 80.00 | %, proportion of insertive oral sex followed by partner's insertive rimming |
| 1.01 | 0.32 | 1.76 | %, Infection prevalence of chlamydia trachomatis only at Oropharynx |
| 2.71 | 1.54 | 4.00 | %, Infection prevalence of chlamydia trachomatis only at Urethral |
| 6.39 | 5.17 | 8.56 | %, Infection prevalence of chlamydia trachomatis only at Rectum |
| 0.00 | 0.00 | 0.00 | %, Infection prevalence of chlamydia trachomatis at Oropharynx & Urethra |
| 2.42 | 1.16 | 3.13 | %, Infection prevalence of chlamydia trachomatis at Oropharynx & Rectum |
| 1.22 | 0.55 | 2.10 | %, Infection prevalence of chlamydia trachomatis at Urethra & Rectum |
| 0.00 | 0.00 | 0.00 | %, Infection prevalence of chlamydia trachomatis at Oropharynx & Urethra & Rectum |

**Table S8. 61.** Model 8’ output parameters with 95% confidence intervals

| **Mean** | **95%CI: lower** | **95%CI: upper** | **Parameters** |
| --- | --- | --- | --- |
| 44.06 | 38.16 | 55.83 | %, Consistent condom usage in anal sex in past 12 months |
| 86.99 | 83.21 | 91.21 | %, Condom efficacy in preventing transmission |
| - | - | - | Times, Frequency of kissing in the past 12 months |
| 15.19 | 5.81 | 22.62 | Times, Frequency of oral sex in the past 12 months |
| 43.36 | 24.11 | 70.06 | Times, Frequency of rimming in the past 12 months |
| 20.13 | 4.08 | 39.16 | Times, Frequency of anal sex in the past 12 months |
| 98.86 | 58.31 | 194.01 | weeks, Infection duration of chlamydia trachomatis at throat(asymptomatic infection) |
| 1.58 | 1.26 | 1.74 | weeks, Infection duration at urethral (symptomatic infection) |
| 86.79 | 83.27 | 89.34 | %, proportion of urethral infections that are asymptomatic |
| 84.24 | 69.91 | 92.99 | %, proportion of anal infections that are asymptomatic |
| 22.96 | 9.04 | 47.68 | weeks, Infection duration at urethral (asymptomatic infection) |
| 49.60 | 48.56 | 51.45 | weeks, Infection duration of chlamydia trachomatis at anus (asymptomatic infection) |
| 1.47 | 1.15 | 1.80 | weeks, Infection duration of chlamydia trachomatis at anus (symptomatic infection) |
| 78.26 | 67.61 | 89.25 | %, proportion of MSM received throat swab in the past 12 months |
| 71.41 | 67.09 | 78.94 | %, proportion of MSM received anal swab in the past 12 months |
| 83.45 | 68.24 | 91.45 | %, proportion of MSM received urine test in the past 12 months |
| 28.50 | 25.86 | 32.41 | %, proportion of 'oral sex and anal sex' in the same sex episode |
| 71.61 | 70.37 | 72.46 | %, proportion of 'oral sex and rimming' in the same sex episode |
| 69.45 | 67.49 | 70.52 | %, proportion of saliva use during anal sex |
| 80.00 | 80.00 | 80.00 | %, proportion of receptive oral sex followed by partner's insertive anal sex |
| 80.00 | 80.00 | 80.00 | %, proportion of insertive oral sex followed by partner's insertive rimming |
| 1.20 | 0.42 | 1.68 | %, Infection prevalence of chlamydia trachomatis only at Oropharynx |
| 2.85 | 1.79 | 4.02 | %, Infection prevalence of chlamydia trachomatis only at Urethral |
| 7.19 | 5.44 | 8.54 | %, Infection prevalence of chlamydia trachomatis only at Rectum |
| 0.00 | 0.00 | 0.00 | %, Infection prevalence of chlamydia trachomatis at Oropharynx & Urethra |
| 1.94 | 1.23 | 2.82 | %, Infection prevalence of chlamydia trachomatis at Oropharynx & Rectum |
| 1.43 | 0.64 | 2.01 | %, Infection prevalence of chlamydia trachomatis at Urethra & Rectum |
| 0.00 | 0.00 | 0.00 | %, Infection prevalence of chlamydia trachomatis at Oropharynx & Urethra & Rectum |

**Table S8. 62.** Model 9’ output parameters with 95% confidence intervals

| **Mean** | **95%CI: lower** | **95%CI: upper** | **Parameters** |
| --- | --- | --- | --- |
| 46.19 | 38.25 | 53.47 | %, Consistent condom usage in anal sex in past 12 months |
| 86.34 | 83.27 | 90.57 | %, Condom efficacy in preventing transmission |
| - | - | - | Times, Frequency of kissing in the past 12 months |
| 12.39 | 6.29 | 22.06 | Times, Frequency of oral sex in the past 12 months |
| 45.81 | 26.00 | 63.01 | Times, Frequency of rimming in the past 12 months |
| 20.01 | 7.99 | 33.11 | Times, Frequency of anal sex in the past 12 months |
| 123.07 | 61.86 | 174.01 | weeks, Infection duration of chlamydia trachomatis at throat(asymptomatic infection) |
| 1.53 | 1.28 | 1.71 | weeks, Infection duration at urethral (symptomatic infection) |
| 86.50 | 83.68 | 89.16 | %, proportion of urethral infections that are asymptomatic |
| 82.98 | 69.85 | 92.96 | %, proportion of anal infections that are asymptomatic |
| 30.66 | 11.21 | 46.76 | weeks, Infection duration at urethral (asymptomatic infection) |
| 50.12 | 48.87 | 51.19 | weeks, Infection duration of chlamydia trachomatis at anus (asymptomatic infection) |
| 1.47 | 1.22 | 1.78 | weeks, Infection duration of chlamydia trachomatis at anus (symptomatic infection) |
| 79.78 | 67.74 | 86.68 | %, proportion of MSM received throat swab in the past 12 months |
| 73.14 | 67.47 | 78.02 | %, proportion of MSM received anal swab in the past 12 months |
| 81.04 | 69.21 | 91.04 | %, proportion of MSM received urine test in the past 12 months |
| 29.52 | 26.19 | 32.21 | %, proportion of 'oral sex and anal sex' in the same sex episode |
| 71.42 | 70.42 | 72.20 | %, proportion of 'oral sex and rimming' in the same sex episode |
| 69.30 | 67.64 | 70.52 | %, proportion of saliva use during anal sex |
| 80.00 | 80.00 | 80.00 | %, proportion of receptive oral sex followed by partner's insertive anal sex |
| 80.00 | 80.00 | 80.00 | %, proportion of insertive oral sex followed by partner's insertive rimming |
| 1.13 | 0.53 | 1.55 | %, Infection prevalence of chlamydia trachomatis only at Oropharynx |
| 2.75 | 1.82 | 3.95 | %, Infection prevalence of chlamydia trachomatis only at Urethral |
| 7.50 | 5.71 | 8.49 | %, Infection prevalence of chlamydia trachomatis only at Rectum |
| 0.00 | 0.00 | 0.00 | %, Infection prevalence of chlamydia trachomatis at Oropharynx & Urethra |
| 1.94 | 1.26 | 2.67 | %, Infection prevalence of chlamydia trachomatis at Oropharynx & Rectum |
| 1.27 | 0.72 | 1.96 | %, Infection prevalence of chlamydia trachomatis at Urethra & Rectum |
| 0.00 | 0.00 | 0.00 | %, Infection prevalence of chlamydia trachomatis at Oropharynx & Urethra & Rectum |

**Table S8. 63.** Model 10’ output parameters with 95% confidence intervals

| **Mean** | **95%CI: lower** | **95%CI: upper** | **Parameters** |
| --- | --- | --- | --- |
| 49.03 | 40.29 | 52.95 | %, Consistent condom usage in anal sex in past 12 months |
| 85.85 | 83.41 | 89.82 | %, Condom efficacy in preventing transmission |
| - | - | - | Times, Frequency of kissing in the past 12 months |
| 13.55 | 7.23 | 20.65 | Times, Frequency of oral sex in the past 12 months |
| 46.52 | 28.90 | 59.72 | Times, Frequency of rimming in the past 12 months |
| 20.53 | 10.71 | 31.58 | Times, Frequency of anal sex in the past 12 months |
| 118.17 | 64.45 | 165.47 | weeks, Infection duration of chlamydia trachomatis at throat(asymptomatic infection) |
| 1.47 | 1.27 | 1.68 | weeks, Infection duration at urethral (symptomatic infection) |
| 86.80 | 84.33 | 88.90 | %, proportion of urethral infections that are asymptomatic |
| 79.15 | 70.84 | 91.61 | %, proportion of anal infections that are asymptomatic |
| 33.38 | 14.68 | 45.17 | weeks, Infection duration at urethral (asymptomatic infection) |
| 49.92 | 48.91 | 50.92 | weeks, Infection duration of chlamydia trachomatis at anus (asymptomatic infection) |
| 1.45 | 1.25 | 1.73 | weeks, Infection duration of chlamydia trachomatis at anus (symptomatic infection) |
| 77.69 | 68.96 | 85.46 | %, proportion of MSM received throat swab in the past 12 months |
| 73.19 | 67.85 | 77.68 | %, proportion of MSM received anal swab in the past 12 months |
| 82.44 | 70.40 | 90.51 | %, proportion of MSM received urine test in the past 12 months |
| 29.02 | 26.28 | 32.07 | %, proportion of 'oral sex and anal sex' in the same sex episode |
| 71.01 | 70.48 | 72.06 | %, proportion of 'oral sex and rimming' in the same sex episode |
| 69.03 | 67.66 | 70.26 | %, proportion of saliva use during anal sex |
| 80.00 | 80.00 | 80.00 | %, proportion of receptive oral sex followed by partner's insertive anal sex |
| 80.00 | 80.00 | 80.00 | %, proportion of insertive oral sex followed by partner's insertive rimming |
| 0.91 | 0.59 | 1.51 | %, Infection prevalence of chlamydia trachomatis only at Oropharynx |
| 2.79 | 2.08 | 3.80 | %, Infection prevalence of chlamydia trachomatis only at Urethral |
| 6.65 | 5.81 | 8.30 | %, Infection prevalence of chlamydia trachomatis only at Rectum |
| 0.00 | 0.00 | 0.00 | %, Infection prevalence of chlamydia trachomatis at Oropharynx & Urethra |
| 1.79 | 1.27 | 2.62 | %, Infection prevalence of chlamydia trachomatis at Oropharynx & Rectum |
| 1.37 | 0.78 | 1.91 | %, Infection prevalence of chlamydia trachomatis at Urethra & Rectum |
| 0.00 | 0.00 | 0.00 | %, Infection prevalence of chlamydia trachomatis at Oropharynx & Urethra & Rectum |

**Table S8. 64.** Model 11’ output parameters with 95% confidence intervals

| **Mean** | **95%CI: lower** | **95%CI: upper** | **Parameters** |
| --- | --- | --- | --- |
| 44.83 | 40.35 | 51.35 | %, Consistent condom usage in anal sex in past 12 months |
| 87.38 | 84.19 | 89.55 | %, Condom efficacy in preventing transmission |
| - | - | - | Times, Frequency of kissing in the past 12 months |
| 13.52 | 8.00 | 20.53 | Times, Frequency of oral sex in the past 12 months |
| 47.19 | 28.71 | 58.11 | Times, Frequency of rimming in the past 12 months |
| 20.70 | 12.28 | 28.74 | Times, Frequency of anal sex in the past 12 months |
| 114.96 | 67.04 | 157.36 | weeks, Infection duration of chlamydia trachomatis at throat(asymptomatic infection) |
| 1.51 | 1.28 | 1.67 | weeks, Infection duration at urethral (symptomatic infection) |
| 86.80 | 84.45 | 88.86 | %, proportion of urethral infections that are asymptomatic |
| 79.42 | 71.15 | 89.78 | %, proportion of anal infections that are asymptomatic |
| 30.21 | 17.42 | 43.39 | weeks, Infection duration at urethral (asymptomatic infection) |
| 50.18 | 49.10 | 50.75 | weeks, Infection duration of chlamydia trachomatis at anus (asymptomatic infection) |
| 1.46 | 1.27 | 1.67 | weeks, Infection duration of chlamydia trachomatis at anus (symptomatic infection) |
| 73.73 | 69.37 | 84.00 | %, proportion of MSM received throat swab in the past 12 months |
| 73.03 | 68.36 | 77.34 | %, proportion of MSM received anal swab in the past 12 months |
| 81.50 | 70.98 | 89.52 | %, proportion of MSM received urine test in the past 12 months |
| 29.71 | 26.49 | 31.97 | %, proportion of 'oral sex and anal sex' in the same sex episode |
| 71.19 | 70.56 | 72.04 | %, proportion of 'oral sex and rimming' in the same sex episode |
| 69.02 | 67.85 | 70.22 | %, proportion of saliva use during anal sex |
| 80.00 | 80.00 | 80.00 | %, proportion of receptive oral sex followed by partner's insertive anal sex |
| 80.00 | 80.00 | 80.00 | %, proportion of insertive oral sex followed by partner's insertive rimming |
| 1.04 | 0.67 | 1.47 | %, Infection prevalence of chlamydia trachomatis only at Oropharynx |
| 2.82 | 2.22 | 3.73 | %, Infection prevalence of chlamydia trachomatis only at Urethral |
| 7.23 | 6.14 | 8.24 | %, Infection prevalence of chlamydia trachomatis only at Rectum |
| 0.00 | 0.00 | 0.00 | %, Infection prevalence of chlamydia trachomatis at Oropharynx & Urethra |
| 1.84 | 1.33 | 2.36 | %, Infection prevalence of chlamydia trachomatis at Oropharynx & Rectum |
| 1.23 | 0.80 | 1.79 | %, Infection prevalence of chlamydia trachomatis at Urethra & Rectum |
| 0.00 | 0.00 | 0.00 | %, Infection prevalence of chlamydia trachomatis at Oropharynx & Urethra & Rectum |

**Table S8. 65.** Model 12’ output parameters with 95% confidence intervals

| **Mean** | **95%CI: lower** | **95%CI: upper** | **Parameters** |
| --- | --- | --- | --- |
| 45.03 | 40.79 | 50.54 | %, Consistent condom usage in anal sex in past 12 months |
| 86.51 | 84.42 | 89.17 | %, Condom efficacy in preventing transmission |
| - | - | - | Times, Frequency of kissing in the past 12 months |
| 13.99 | 8.46 | 19.71 | Times, Frequency of oral sex in the past 12 months |
| 41.80 | 29.49 | 56.07 | Times, Frequency of rimming in the past 12 months |
| 21.52 | 14.88 | 28.70 | Times, Frequency of anal sex in the past 12 months |
| 103.49 | 68.16 | 150.44 | weeks, Infection duration of chlamydia trachomatis at throat(asymptomatic infection) |
| 1.49 | 1.30 | 1.66 | weeks, Infection duration at urethral (symptomatic infection) |
| 86.72 | 84.94 | 88.53 | %, proportion of urethral infections that are asymptomatic |
| 77.79 | 72.24 | 88.87 | %, proportion of anal infections that are asymptomatic |
| 28.54 | 18.15 | 40.59 | weeks, Infection duration at urethral (asymptomatic infection) |
| 49.88 | 49.19 | 50.70 | weeks, Infection duration of chlamydia trachomatis at anus (asymptomatic infection) |
| 1.44 | 1.30 | 1.65 | weeks, Infection duration of chlamydia trachomatis at anus (symptomatic infection) |
| 78.21 | 69.93 | 83.43 | %, proportion of MSM received throat swab in the past 12 months |
| 71.03 | 68.17 | 77.05 | %, proportion of MSM received anal swab in the past 12 months |
| 78.88 | 71.74 | 88.69 | %, proportion of MSM received urine test in the past 12 months |
| 29.23 | 26.52 | 31.77 | %, proportion of 'oral sex and anal sex' in the same sex episode |
| 71.22 | 70.61 | 71.95 | %, proportion of 'oral sex and rimming' in the same sex episode |
| 69.02 | 67.92 | 69.88 | %, proportion of saliva use during anal sex |
| 80.00 | 80.00 | 80.00 | %, proportion of receptive oral sex followed by partner's insertive anal sex |
| 80.00 | 80.00 | 80.00 | %, proportion of insertive oral sex followed by partner's insertive rimming |
| 0.95 | 0.66 | 1.44 | %, Infection prevalence of chlamydia trachomatis only at Oropharynx |
| 2.94 | 2.39 | 3.71 | %, Infection prevalence of chlamydia trachomatis only at Urethral |
| 7.19 | 6.27 | 8.21 | %, Infection prevalence of chlamydia trachomatis only at Rectum |
| 0.00 | 0.00 | 0.00 | %, Infection prevalence of chlamydia trachomatis at Oropharynx & Urethra |
| 1.70 | 1.36 | 2.24 | %, Infection prevalence of chlamydia trachomatis at Oropharynx & Rectum |
| 1.34 | 0.88 | 1.73 | %, Infection prevalence of chlamydia trachomatis at Urethra & Rectum |
| 0.00 | 0.00 | 0.00 | %, Infection prevalence of chlamydia trachomatis at Oropharynx & Urethra & Rectum |

**Table S8. 66.** Model 13’ output parameters with 95% confidence intervals

| **Mean** | **95%CI: lower** | **95%CI: upper** | **Parameters** |
| --- | --- | --- | --- |
| 44.47 | 41.06 | 49.34 | %, Consistent condom usage in anal sex in past 12 months |
| 86.64 | 84.82 | 89.02 | %, Condom efficacy in preventing transmission |
| - | - | - | Times, Frequency of kissing in the past 12 months |
| 13.37 | 9.25 | 18.68 | Times, Frequency of oral sex in the past 12 months |
| 43.74 | 31.24 | 55.64 | Times, Frequency of rimming in the past 12 months |
| 21.33 | 15.01 | 28.14 | Times, Frequency of anal sex in the past 12 months |
| 91.34 | 71.21 | 145.02 | weeks, Infection duration of chlamydia trachomatis at throat(asymptomatic infection) |
| 1.42 | 1.35 | 1.64 | weeks, Infection duration at urethral (symptomatic infection) |
| 86.38 | 84.93 | 87.49 | %, proportion of urethral infections that are asymptomatic |
| 80.88 | 73.07 | 85.80 | %, proportion of anal infections that are asymptomatic |
| 29.30 | 19.47 | 39.44 | weeks, Infection duration at urethral (asymptomatic infection) |
| 49.90 | 49.22 | 50.60 | weeks, Infection duration of chlamydia trachomatis at anus (asymptomatic infection) |
| 1.47 | 1.31 | 1.63 | weeks, Infection duration of chlamydia trachomatis at anus (symptomatic infection) |
| 77.53 | 71.52 | 82.72 | %, proportion of MSM received throat swab in the past 12 months |
| 72.31 | 69.00 | 76.57 | %, proportion of MSM received anal swab in the past 12 months |
| 78.97 | 72.03 | 87.83 | %, proportion of MSM received urine test in the past 12 months |
| 29.71 | 27.35 | 31.59 | %, proportion of 'oral sex and anal sex' in the same sex episode |
| 71.38 | 70.72 | 71.85 | %, proportion of 'oral sex and rimming' in the same sex episode |
| 68.82 | 68.09 | 69.83 | %, proportion of saliva use during anal sex |
| 80.00 | 80.00 | 80.00 | %, proportion of receptive oral sex followed by partner's insertive anal sex |
| 80.00 | 80.00 | 80.00 | %, proportion of insertive oral sex followed by partner's insertive rimming |
| 1.08 | 0.79 | 1.42 | %, Infection prevalence of chlamydia trachomatis only at Oropharynx |
| 3.15 | 2.42 | 3.68 | %, Infection prevalence of chlamydia trachomatis only at Urethral |
| 7.40 | 6.40 | 8.15 | %, Infection prevalence of chlamydia trachomatis only at Rectum |
| 0.00 | 0.00 | 0.00 | %, Infection prevalence of chlamydia trachomatis at Oropharynx & Urethra |
| 1.82 | 1.42 | 2.22 | %, Infection prevalence of chlamydia trachomatis at Oropharynx & Rectum |
| 1.37 | 0.93 | 1.67 | %, Infection prevalence of chlamydia trachomatis at Urethra & Rectum |
| 0.00 | 0.00 | 0.00 | %, Infection prevalence of chlamydia trachomatis at Oropharynx & Urethra & Rectum |

Model output parameters (Published validation dataset3): Data from MSM surveillance data of all Dutch STI clinics

**Table S8. 67.** Model 2’ output parameters with 95% confidence intervals

| **Mean** | **95%CI: lower** | **95%CI: upper** | **Parameters** |
| --- | --- | --- | --- |
| 44.97 | 34.87 | 56.82 | %, Consistent condom usage in anal sex in past 12 months |
| 87.44 | 80.56 | 93.54 | %, Condom efficacy in preventing transmission |
| - | - | - | Times, Frequency of kissing in the past 12 months |
| 10.72 | 1.88 | 26.08 | Times, Frequency of oral sex in the past 12 months |
| 19.24 | 2.69 | 67.13 | Times, Frequency of rimming in the past 12 months |
| 12.82 | 2.12 | 48.88 | Times, Frequency of anal sex in the past 12 months |
| 100.44 | 31.72 | 236.78 | weeks, Infection duration of chlamydia trachomatis at throat(asymptomatic infection) |
| 1.53 | 1.14 | 1.96 | weeks, Infection duration at urethral (symptomatic infection) |
| 100.00 | 100.00 | 100.00 | %, proportion of urethral infections that are asymptomatic |
| 100.00 | 100.00 | 100.00 | %, proportion of anal infections that are asymptomatic |
| 29.41 | 14.12 | 50.45 | weeks, Infection duration at urethral (asymptomatic infection) |
| 50.08 | 48.25 | 51.73 | weeks, Infection duration of chlamydia trachomatis at anus (asymptomatic infection) |
| 1.52 | 1.13 | 1.91 | weeks, Infection duration of chlamydia trachomatis at anus (symptomatic infection) |
| 83.86 | 65.06 | 95.48 | %, proportion of MSM received throat swab in the past 12 months |
| 75.02 | 65.30 | 93.58 | %, proportion of MSM received anal swab in the past 12 months |
| 75.54 | 64.95 | 92.25 | %, proportion of MSM received urine test in the past 12 months |
| 28.43 | 25.49 | 33.51 | %, proportion of 'oral sex and anal sex' in the same sex episode |
| 71.35 | 70.08 | 72.67 | %, proportion of 'oral sex and rimming' in the same sex episode |
| 68.73 | 66.24 | 70.62 | %, proportion of saliva use during anal sex |
| 80.00 | 80.00 | 80.00 | %, proportion of receptive oral sex followed by partner's insertive anal sex |
| 80.00 | 80.00 | 80.00 | %, proportion of insertive oral sex followed by partner's insertive rimming |
| 0.50 | 0.49 | 0.53 | %, Infection prevalence of chlamydia trachomatis only at Oropharynx |
| 2.32 | 2.24 | 2.36 | %, Infection prevalence of chlamydia trachomatis only at Urethral |
| 6.25 | 6.17 | 6.38 | %, Infection prevalence of chlamydia trachomatis only at Rectum |
| 0.06 | 0.05 | 0.07 | %, Infection prevalence of chlamydia trachomatis at Oropharynx & Urethra |
| 0.54 | 0.51 | 0.58 | %, Infection prevalence of chlamydia trachomatis at Oropharynx & Rectum |
| 1.03 | 0.99 | 1.07 | %, Infection prevalence of chlamydia trachomatis at Urethra & Rectum |
| 0.10 | 0.09 | 0.12 | %, Infection prevalence of chlamydia trachomatis at Oropharynx & Urethra & Rectum |

**Table S8. 68.** Model 7’ output parameters with 95% confidence intervals

| **Mean** | **95%CI: lower** | **95%CI: upper** | **Parameters** |
| --- | --- | --- | --- |
| 46.58 | 35.93 | 55.70 | %, Consistent condom usage in anal sex in past 12 months |
| 84.68 | 81.40 | 91.84 | %, Condom efficacy in preventing transmission |
| - | - | - | Times, Frequency of kissing in the past 12 months |
| 11.73 | 2.51 | 24.86 | Times, Frequency of oral sex in the past 12 months |
| 50.42 | 11.99 | 66.58 | Times, Frequency of rimming in the past 12 months |
| 12.81 | 2.37 | 22.75 | Times, Frequency of anal sex in the past 12 months |
| 170.10 | 67.86 | 223.05 | weeks, Infection duration of chlamydia trachomatis at throat(asymptomatic infection) |
| 1.51 | 1.22 | 1.92 | weeks, Infection duration at urethral (symptomatic infection) |
| 100.00 | 100.00 | 100.00 | %, proportion of urethral infections that are asymptomatic |
| 100.00 | 100.00 | 100.00 | %, proportion of anal infections that are asymptomatic |
| 36.20 | 21.86 | 48.24 | weeks, Infection duration at urethral (asymptomatic infection) |
| 50.13 | 48.51 | 51.29 | weeks, Infection duration of chlamydia trachomatis at anus (asymptomatic infection) |
| 1.47 | 1.15 | 1.84 | weeks, Infection duration of chlamydia trachomatis at anus (symptomatic infection) |
| 75.29 | 66.60 | 94.25 | %, proportion of MSM received throat swab in the past 12 months |
| 79.68 | 70.21 | 91.69 | %, proportion of MSM received anal swab in the past 12 months |
| 79.60 | 69.25 | 90.16 | %, proportion of MSM received urine test in the past 12 months |
| 30.18 | 26.93 | 32.97 | %, proportion of 'oral sex and anal sex' in the same sex episode |
| 71.26 | 70.34 | 72.64 | %, proportion of 'oral sex and rimming' in the same sex episode |
| 68.82 | 67.05 | 70.09 | %, proportion of saliva use during anal sex |
| 80.00 | 80.00 | 80.00 | %, proportion of receptive oral sex followed by partner's insertive anal sex |
| 80.00 | 80.00 | 80.00 | %, proportion of insertive oral sex followed by partner's insertive rimming |
| 0.50 | 0.49 | 0.52 | %, Infection prevalence of chlamydia trachomatis only at Oropharynx |
| 2.29 | 2.24 | 2.35 | %, Infection prevalence of chlamydia trachomatis only at Urethral |
| 6.26 | 6.18 | 6.37 | %, Infection prevalence of chlamydia trachomatis only at Rectum |
| 0.06 | 0.05 | 0.07 | %, Infection prevalence of chlamydia trachomatis at Oropharynx & Urethra |
| 0.55 | 0.52 | 0.57 | %, Infection prevalence of chlamydia trachomatis at Oropharynx & Rectum |
| 1.03 | 0.99 | 1.06 | %, Infection prevalence of chlamydia trachomatis at Urethra & Rectum |
| 0.11 | 0.09 | 0.11 | %, Infection prevalence of chlamydia trachomatis at Oropharynx & Urethra & Rectum |

**Table S8. 69.** Model 8’ output parameters with 95% confidence intervals

| **Mean** | **95%CI: lower** | **95%CI: upper** | **Parameters** |
| --- | --- | --- | --- |
| 46.85 | 36.64 | 55.31 | %, Consistent condom usage in anal sex in past 12 months |
| 88.02 | 81.33 | 90.06 | %, Condom efficacy in preventing transmission |
| - | - | - | Times, Frequency of kissing in the past 12 months |
| 16.97 | 3.74 | 22.44 | Times, Frequency of oral sex in the past 12 months |
| 33.95 | 12.71 | 58.60 | Times, Frequency of rimming in the past 12 months |
| 15.25 | 4.87 | 22.21 | Times, Frequency of anal sex in the past 12 months |
| 139.02 | 87.40 | 211.59 | weeks, Infection duration of chlamydia trachomatis at throat(asymptomatic infection) |
| 1.52 | 1.24 | 1.86 | weeks, Infection duration at urethral (symptomatic infection) |
| 100.00 | 100.00 | 100.00 | %, proportion of urethral infections that are asymptomatic |
| 100.00 | 100.00 | 100.00 | %, proportion of anal infections that are asymptomatic |
| 31.30 | 22.67 | 44.57 | weeks, Infection duration at urethral (asymptomatic infection) |
| 49.40 | 48.63 | 50.91 | weeks, Infection duration of chlamydia trachomatis at anus (asymptomatic infection) |
| 1.47 | 1.18 | 1.82 | weeks, Infection duration of chlamydia trachomatis at anus (symptomatic infection) |
| 80.73 | 71.37 | 93.82 | %, proportion of MSM received throat swab in the past 12 months |
| 79.19 | 71.73 | 89.09 | %, proportion of MSM received anal swab in the past 12 months |
| 79.99 | 70.18 | 88.33 | %, proportion of MSM received urine test in the past 12 months |
| 30.11 | 27.40 | 32.79 | %, proportion of 'oral sex and anal sex' in the same sex episode |
| 71.61 | 70.54 | 72.57 | %, proportion of 'oral sex and rimming' in the same sex episode |
| 68.60 | 67.31 | 70.02 | %, proportion of saliva use during anal sex |
| 80.00 | 80.00 | 80.00 | %, proportion of receptive oral sex followed by partner's insertive anal sex |
| 80.00 | 80.00 | 80.00 | %, proportion of insertive oral sex followed by partner's insertive rimming |
| 0.51 | 0.49 | 0.52 | %, Infection prevalence of chlamydia trachomatis only at Oropharynx |
| 2.29 | 2.25 | 2.34 | %, Infection prevalence of chlamydia trachomatis only at Urethral |
| 6.27 | 6.19 | 6.36 | %, Infection prevalence of chlamydia trachomatis only at Rectum |
| 0.06 | 0.05 | 0.07 | %, Infection prevalence of chlamydia trachomatis at Oropharynx & Urethra |
| 0.54 | 0.52 | 0.56 | %, Infection prevalence of chlamydia trachomatis at Oropharynx & Rectum |
| 1.03 | 1.00 | 1.06 | %, Infection prevalence of chlamydia trachomatis at Urethra & Rectum |
| 0.10 | 0.10 | 0.11 | %, Infection prevalence of chlamydia trachomatis at Oropharynx & Urethra & Rectum |

**Table S8. 70.** Model 9’ output parameters with 95% confidence intervals

| **Mean** | **95%CI: lower** | **95%CI: upper** | **Parameters** |
| --- | --- | --- | --- |
| 45.35 | 37.61 | 53.14 | %, Consistent condom usage in anal sex in past 12 months |
| 85.14 | 81.78 | 89.65 | %, Condom efficacy in preventing transmission |
| - | - | - | Times, Frequency of kissing in the past 12 months |
| 13.61 | 4.51 | 21.85 | Times, Frequency of oral sex in the past 12 months |
| 31.85 | 19.05 | 57.89 | Times, Frequency of rimming in the past 12 months |
| 12.49 | 5.76 | 20.24 | Times, Frequency of anal sex in the past 12 months |
| 148.28 | 88.25 | 209.59 | weeks, Infection duration of chlamydia trachomatis at throat(asymptomatic infection) |
| 1.50 | 1.24 | 1.86 | weeks, Infection duration at urethral (symptomatic infection) |
| 100.00 | 100.00 | 100.00 | %, proportion of urethral infections that are asymptomatic |
| 100.00 | 100.00 | 100.00 | %, proportion of anal infections that are asymptomatic |
| 34.18 | 24.54 | 44.25 | weeks, Infection duration at urethral (asymptomatic infection) |
| 49.99 | 48.72 | 50.77 | weeks, Infection duration of chlamydia trachomatis at anus (asymptomatic infection) |
| 1.46 | 1.18 | 1.78 | weeks, Infection duration of chlamydia trachomatis at anus (symptomatic infection) |
| 83.19 | 72.33 | 92.93 | %, proportion of MSM received throat swab in the past 12 months |
| 78.84 | 72.25 | 87.31 | %, proportion of MSM received anal swab in the past 12 months |
| 76.11 | 70.06 | 88.14 | %, proportion of MSM received urine test in the past 12 months |
| 29.96 | 27.76 | 32.38 | %, proportion of 'oral sex and anal sex' in the same sex episode |
| 71.57 | 70.60 | 72.50 | %, proportion of 'oral sex and rimming' in the same sex episode |
| 68.36 | 67.39 | 69.78 | %, proportion of saliva use during anal sex |
| 80.00 | 80.00 | 80.00 | %, proportion of receptive oral sex followed by partner's insertive anal sex |
| 80.00 | 80.00 | 80.00 | %, proportion of insertive oral sex followed by partner's insertive rimming |
| 0.50 | 0.49 | 0.52 | %, Infection prevalence of chlamydia trachomatis only at Oropharynx |
| 2.30 | 2.25 | 2.33 | %, Infection prevalence of chlamydia trachomatis only at Urethral |
| 6.29 | 6.20 | 6.36 | %, Infection prevalence of chlamydia trachomatis only at Rectum |
| 0.06 | 0.05 | 0.06 | %, Infection prevalence of chlamydia trachomatis at Oropharynx & Urethra |
| 0.55 | 0.52 | 0.56 | %, Infection prevalence of chlamydia trachomatis at Oropharynx & Rectum |
| 1.04 | 1.00 | 1.06 | %, Infection prevalence of chlamydia trachomatis at Urethra & Rectum |
| 0.10 | 0.10 | 0.11 | %, Infection prevalence of chlamydia trachomatis at Oropharynx & Urethra & Rectum |

**Table S8. 71**. Model 10’ output parameters with 95% confidence intervals

| **Mean** | **95%CI: lower** | **95%CI: upper** | **Parameters** |
| --- | --- | --- | --- |
| 45.72 | 39.23 | 50.87 | %, Consistent condom usage in anal sex in past 12 months |
| 84.96 | 82.54 | 88.70 | %, Condom efficacy in preventing transmission |
| - | - | - | Times, Frequency of kissing in the past 12 months |
| 6.86 | 4.65 | 13.59 | Times, Frequency of oral sex in the past 12 months |
| 41.77 | 20.23 | 54.07 | Times, Frequency of rimming in the past 12 months |
| 15.66 | 8.75 | 18.89 | Times, Frequency of anal sex in the past 12 months |
| 158.92 | 93.17 | 207.98 | weeks, Infection duration of chlamydia trachomatis at throat(asymptomatic infection) |
| 1.55 | 1.28 | 1.80 | weeks, Infection duration at urethral (symptomatic infection) |
| 100.00 | 100.00 | 100.00 | %, proportion of urethral infections that are asymptomatic |
| 100.00 | 100.00 | 100.00 | %, proportion of anal infections that are asymptomatic |
| 35.54 | 27.31 | 43.75 | weeks, Infection duration at urethral (asymptomatic infection) |
| 49.93 | 49.12 | 50.71 | weeks, Infection duration of chlamydia trachomatis at anus (asymptomatic infection) |
| 1.56 | 1.29 | 1.75 | weeks, Infection duration of chlamydia trachomatis at anus (symptomatic infection) |
| 82.11 | 72.67 | 92.13 | %, proportion of MSM received throat swab in the past 12 months |
| 80.70 | 73.04 | 86.73 | %, proportion of MSM received anal swab in the past 12 months |
| 79.92 | 71.66 | 84.18 | %, proportion of MSM received urine test in the past 12 months |
| 31.08 | 29.13 | 32.35 | %, proportion of 'oral sex and anal sex' in the same sex episode |
| 71.33 | 70.72 | 72.43 | %, proportion of 'oral sex and rimming' in the same sex episode |
| 68.67 | 67.46 | 69.69 | %, proportion of saliva use during anal sex |
| 80.00 | 80.00 | 80.00 | %, proportion of receptive oral sex followed by partner's insertive anal sex |
| 80.00 | 80.00 | 80.00 | %, proportion of insertive oral sex followed by partner's insertive rimming |
| 0.50 | 0.49 | 0.51 | %, Infection prevalence of chlamydia trachomatis only at Oropharynx |
| 2.29 | 2.25 | 2.32 | %, Infection prevalence of chlamydia trachomatis only at Urethral |
| 6.28 | 6.21 | 6.35 | %, Infection prevalence of chlamydia trachomatis only at Rectum |
| 0.06 | 0.05 | 0.06 | %, Infection prevalence of chlamydia trachomatis at Oropharynx & Urethra |
| 0.54 | 0.52 | 0.56 | %, Infection prevalence of chlamydia trachomatis at Oropharynx & Rectum |
| 1.03 | 1.00 | 1.05 | %, Infection prevalence of chlamydia trachomatis at Urethra & Rectum |
| 0.10 | 0.10 | 0.11 | %, Infection prevalence of chlamydia trachomatis at Oropharynx & Urethra & Rectum |

**Table S8. 72.** Model 11’ output parameters with 95% confidence intervals

| **Mean** | **95%CI: lower** | **95%CI: upper** | **Parameters** |
| --- | --- | --- | --- |
| 45.43 | 39.35 | 50.24 | %, Consistent condom usage in anal sex in past 12 months |
| 85.61 | 82.85 | 88.26 | %, Condom efficacy in preventing transmission |
| - | - | - | Times, Frequency of kissing in the past 12 months |
| 9.02 | 4.83 | 13.47 | Times, Frequency of oral sex in the past 12 months |
| 36.50 | 24.21 | 53.39 | Times, Frequency of rimming in the past 12 months |
| 13.98 | 9.28 | 18.05 | Times, Frequency of anal sex in the past 12 months |
| 169.78 | 118.25 | 205.66 | weeks, Infection duration of chlamydia trachomatis at throat(asymptomatic infection) |
| 1.49 | 1.27 | 1.78 | weeks, Infection duration at urethral (symptomatic infection) |
| 100.00 | 100.00 | 100.00 | %, proportion of urethral infections that are asymptomatic |
| 100.00 | 100.00 | 100.00 | %, proportion of anal infections that are asymptomatic |
| 37.03 | 28.67 | 42.62 | weeks, Infection duration at urethral (asymptomatic infection) |
| 50.00 | 49.20 | 50.70 | weeks, Infection duration of chlamydia trachomatis at anus (asymptomatic infection) |
| 1.49 | 1.29 | 1.74 | weeks, Infection duration of chlamydia trachomatis at anus (symptomatic infection) |
| 81.30 | 73.10 | 88.35 | %, proportion of MSM received throat swab in the past 12 months |
| 81.67 | 75.27 | 86.39 | %, proportion of MSM received anal swab in the past 12 months |
| 77.06 | 71.31 | 83.67 | %, proportion of MSM received urine test in the past 12 months |
| 31.17 | 29.30 | 31.96 | %, proportion of 'oral sex and anal sex' in the same sex episode |
| 71.61 | 70.82 | 72.43 | %, proportion of 'oral sex and rimming' in the same sex episode |
| 68.18 | 67.57 | 69.54 | %, proportion of saliva use during anal sex |
| 80.00 | 80.00 | 80.00 | %, proportion of receptive oral sex followed by partner's insertive anal sex |
| 80.00 | 80.00 | 80.00 | %, proportion of insertive oral sex followed by partner's insertive rimming |
| 0.50 | 0.49 | 0.51 | %, Infection prevalence of chlamydia trachomatis only at Oropharynx |
| 2.29 | 2.25 | 2.32 | %, Infection prevalence of chlamydia trachomatis only at Urethral |
| 6.27 | 6.22 | 6.35 | %, Infection prevalence of chlamydia trachomatis only at Rectum |
| 0.06 | 0.05 | 0.06 | %, Infection prevalence of chlamydia trachomatis at Oropharynx & Urethra |
| 0.54 | 0.52 | 0.55 | %, Infection prevalence of chlamydia trachomatis at Oropharynx & Rectum |
| 1.02 | 1.00 | 1.04 | %, Infection prevalence of chlamydia trachomatis at Urethra & Rectum |
| 0.11 | 0.10 | 0.11 | %, Infection prevalence of chlamydia trachomatis at Oropharynx & Urethra & Rectum |

**Table S8. 73.** Model 12’ output parameters with 95% confidence intervals

| **Mean** | **95%CI: lower** | **95%CI: upper** | **Parameters** |
| --- | --- | --- | --- |
| 45.36 | 40.21 | 49.13 | %, Consistent condom usage in anal sex in past 12 months |
| 85.64 | 83.30 | 87.77 | %, Condom efficacy in preventing transmission |
| - | - | - | Times, Frequency of kissing in the past 12 months |
| 9.58 | 6.06 | 12.90 | Times, Frequency of oral sex in the past 12 months |
| 41.75 | 26.76 | 52.28 | Times, Frequency of rimming in the past 12 months |
| 13.20 | 9.67 | 17.92 | Times, Frequency of anal sex in the past 12 months |
| 163.89 | 140.80 | 193.44 | weeks, Infection duration of chlamydia trachomatis at throat(asymptomatic infection) |
| 1.46 | 1.30 | 1.68 | weeks, Infection duration at urethral (symptomatic infection) |
| 100.00 | 100.00 | 100.00 | %, proportion of urethral infections that are asymptomatic |
| 100.00 | 100.00 | 100.00 | %, proportion of anal infections that are asymptomatic |
| 34.81 | 29.04 | 41.57 | weeks, Infection duration at urethral (asymptomatic infection) |
| 50.06 | 49.27 | 50.65 | weeks, Infection duration of chlamydia trachomatis at anus (asymptomatic infection) |
| 1.54 | 1.36 | 1.71 | weeks, Infection duration of chlamydia trachomatis at anus (symptomatic infection) |
| 79.60 | 74.13 | 87.09 | %, proportion of MSM received throat swab in the past 12 months |
| 80.39 | 76.62 | 86.01 | %, proportion of MSM received anal swab in the past 12 months |
| 77.40 | 71.68 | 82.45 | %, proportion of MSM received urine test in the past 12 months |
| 30.51 | 29.68 | 31.49 | %, proportion of 'oral sex and anal sex' in the same sex episode |
| 71.55 | 70.85 | 72.08 | %, proportion of 'oral sex and rimming' in the same sex episode |
| 68.34 | 67.78 | 69.24 | %, proportion of saliva use during anal sex |
| 80.00 | 80.00 | 80.00 | %, proportion of receptive oral sex followed by partner's insertive anal sex |
| 80.00 | 80.00 | 80.00 | %, proportion of insertive oral sex followed by partner's insertive rimming |
| 0.50 | 0.50 | 0.51 | %, Infection prevalence of chlamydia trachomatis only at Oropharynx |
| 2.28 | 2.26 | 2.31 | %, Infection prevalence of chlamydia trachomatis only at Urethral |
| 6.29 | 6.24 | 6.35 | %, Infection prevalence of chlamydia trachomatis only at Rectum |
| 0.06 | 0.05 | 0.06 | %, Infection prevalence of chlamydia trachomatis at Oropharynx & Urethra |
| 0.53 | 0.53 | 0.55 | %, Infection prevalence of chlamydia trachomatis at Oropharynx & Rectum |
| 1.02 | 1.01 | 1.04 | %, Infection prevalence of chlamydia trachomatis at Urethra & Rectum |
| 0.11 | 0.10 | 0.11 | %, Infection prevalence of chlamydia trachomatis at Oropharynx & Urethra & Rectum |

**Table S8. 74.** Model 13’ output parameters with 95% confidence intervals

| **Mean** | **95%CI: lower** | **95%CI: upper** | **Parameters** |
| --- | --- | --- | --- |
| 44.76 | 41.40 | 48.79 | %, Consistent condom usage in anal sex in past 12 months |
| 85.52 | 83.47 | 87.48 | %, Condom efficacy in preventing transmission |
| - | - | - | Times, Frequency of kissing in the past 12 months |
| 6.88 | 6.16 | 11.01 | Times, Frequency of oral sex in the past 12 months |
| 40.80 | 27.29 | 49.79 | Times, Frequency of rimming in the past 12 months |
| 15.79 | 12.88 | 17.88 | Times, Frequency of anal sex in the past 12 months |
| 173.73 | 144.27 | 191.93 | weeks, Infection duration of chlamydia trachomatis at throat(asymptomatic infection) |
| 1.48 | 1.32 | 1.64 | weeks, Infection duration at urethral (symptomatic infection) |
| 100.00 | 100.00 | 100.00 | %, proportion of urethral infections that are asymptomatic |
| 100.00 | 100.00 | 100.00 | %, proportion of anal infections that are asymptomatic |
| 34.31 | 29.44 | 39.74 | weeks, Infection duration at urethral (asymptomatic infection) |
| 50.15 | 49.45 | 50.50 | weeks, Infection duration of chlamydia trachomatis at anus (asymptomatic infection) |
| 1.54 | 1.38 | 1.70 | weeks, Infection duration of chlamydia trachomatis at anus (symptomatic infection) |
| 81.71 | 74.94 | 86.22 | %, proportion of MSM received throat swab in the past 12 months |
| 81.64 | 76.79 | 85.85 | %, proportion of MSM received anal swab in the past 12 months |
| 76.32 | 72.54 | 81.59 | %, proportion of MSM received urine test in the past 12 months |
| 30.68 | 29.85 | 31.48 | %, proportion of 'oral sex and anal sex' in the same sex episode |
| 71.28 | 70.88 | 71.90 | %, proportion of 'oral sex and rimming' in the same sex episode |
| 68.49 | 67.82 | 69.09 | %, proportion of saliva use during anal sex |
| 80.00 | 80.00 | 80.00 | %, proportion of receptive oral sex followed by partner's insertive anal sex |
| 80.00 | 80.00 | 80.00 | %, proportion of insertive oral sex followed by partner's insertive rimming |
| 0.50 | 0.50 | 0.51 | %, Infection prevalence of chlamydia trachomatis only at Oropharynx |
| 2.28 | 2.26 | 2.31 | %, Infection prevalence of chlamydia trachomatis only at Urethral |
| 6.30 | 6.25 | 6.35 | %, Infection prevalence of chlamydia trachomatis only at Rectum |
| 0.06 | 0.06 | 0.06 | %, Infection prevalence of chlamydia trachomatis at Oropharynx & Urethra |
| 0.54 | 0.53 | 0.55 | %, Infection prevalence of chlamydia trachomatis at Oropharynx & Rectum |
| 1.02 | 1.01 | 1.04 | %, Infection prevalence of chlamydia trachomatis at Urethra & Rectum |
| 0.10 | 0.10 | 0.11 | %, Infection prevalence of chlamydia trachomatis at Oropharynx & Urethra & Rectum |

Model output parameters (Published validation dataset4): Data from 1,610 community MSM in Thailand

**Table S8. 75.** Model 2’ output parameters with 95% confidence intervals

| **Mean** | **95%CI: lower** | **95%CI: upper** | **Parameters** |
| --- | --- | --- | --- |
| 46.18 | 36.36 | 55.59 | %, Consistent condom usage in anal sex in past 12 months |
| 87.71 | 81.29 | 93.06 | %, Condom efficacy in preventing transmission |
| - | - | - | Times, Frequency of kissing in the past 12 months |
| 17.81 | 2.29 | 26.93 | Times, Frequency of oral sex in the past 12 months |
| 30.17 | 4.25 | 69.51 | Times, Frequency of rimming in the past 12 months |
| 33.51 | 2.31 | 51.95 | Times, Frequency of anal sex in the past 12 months |
| 146.84 | 33.42 | 238.52 | weeks, Infection duration of chlamydia trachomatis at throat(asymptomatic infection) |
| 1.58 | 1.06 | 1.90 | weeks, Infection duration at urethral (symptomatic infection) |
| 100.00 | 100.00 | 100.00 | %, proportion of urethral infections that are asymptomatic |
| 100.00 | 100.00 | 100.00 | %, proportion of anal infections that are asymptomatic |
| 22.18 | 3.95 | 47.21 | weeks, Infection duration at urethral (asymptomatic infection) |
| 50.33 | 48.21 | 51.71 | weeks, Infection duration of chlamydia trachomatis at anus (asymptomatic infection) |
| 1.44 | 1.15 | 1.95 | weeks, Infection duration of chlamydia trachomatis at anus (symptomatic infection) |
| 81.60 | 67.26 | 95.40 | %, proportion of MSM received throat swab in the past 12 months |
| 80.36 | 64.40 | 94.33 | %, proportion of MSM received anal swab in the past 12 months |
| 78.51 | 65.88 | 93.38 | %, proportion of MSM received urine test in the past 12 months |
| 30.07 | 25.41 | 33.61 | %, proportion of 'oral sex and anal sex' in the same sex episode |
| 71.16 | 69.79 | 72.74 | %, proportion of 'oral sex and rimming' in the same sex episode |
| 68.51 | 66.29 | 70.80 | %, proportion of saliva use during anal sex |
| 80.00 | 80.00 | 80.00 | %, proportion of receptive oral sex followed by partner's insertive anal sex |
| 80.00 | 80.00 | 80.00 | %, proportion of insertive oral sex followed by partner's insertive rimming |
| 1.68 | 1.19 | 2.47 | %, Infection prevalence of chlamydia trachomatis only at Oropharynx |
| 5.18 | 3.94 | 5.90 | %, Infection prevalence of chlamydia trachomatis only at Urethral |
| 11.90 | 10.44 | 12.88 | %, Infection prevalence of chlamydia trachomatis only at Rectum |
| 0.21 | 0.03 | 0.37 | %, Infection prevalence of chlamydia trachomatis at Oropharynx & Urethra |
| 1.31 | 0.75 | 1.64 | %, Infection prevalence of chlamydia trachomatis at Oropharynx & Rectum |
| 2.09 | 1.55 | 2.79 | %, Infection prevalence of chlamydia trachomatis at Urethra & Rectum |
| 0.22 | 0.05 | 0.38 | %, Infection prevalence of chlamydia trachomatis at Oropharynx & Urethra & Rectum |

**Table S8. 76.** Model 7’ output parameters with 95% confidence intervals

| **Mean** | **95%CI: lower** | **95%CI: upper** | **Parameters** |
| --- | --- | --- | --- |
| 47.61 | 36.78 | 54.62 | %, Consistent condom usage in anal sex in past 12 months |
| 87.30 | 81.63 | 92.45 | %, Condom efficacy in preventing transmission |
| - | - | - | Times, Frequency of kissing in the past 12 months |
| 13.96 | 3.44 | 26.51 | Times, Frequency of oral sex in the past 12 months |
| 37.81 | 6.68 | 66.14 | Times, Frequency of rimming in the past 12 months |
| 27.95 | 6.24 | 49.10 | Times, Frequency of anal sex in the past 12 months |
| 152.41 | 49.14 | 225.18 | weeks, Infection duration of chlamydia trachomatis at throat(asymptomatic infection) |
| 1.56 | 1.20 | 1.85 | weeks, Infection duration at urethral (symptomatic infection) |
| 100.00 | 100.00 | 100.00 | %, proportion of urethral infections that are asymptomatic |
| 100.00 | 100.00 | 100.00 | %, proportion of anal infections that are asymptomatic |
| 26.90 | 12.08 | 48.43 | weeks, Infection duration at urethral (asymptomatic infection) |
| 50.11 | 48.30 | 51.43 | weeks, Infection duration of chlamydia trachomatis at anus (asymptomatic infection) |
| 1.59 | 1.18 | 1.90 | weeks, Infection duration of chlamydia trachomatis at anus (symptomatic infection) |
| 81.34 | 71.87 | 93.33 | %, proportion of MSM received throat swab in the past 12 months |
| 76.52 | 66.07 | 86.69 | %, proportion of MSM received anal swab in the past 12 months |
| 78.21 | 68.64 | 92.32 | %, proportion of MSM received urine test in the past 12 months |
| 29.42 | 25.83 | 33.11 | %, proportion of 'oral sex and anal sex' in the same sex episode |
| 71.41 | 70.00 | 72.67 | %, proportion of 'oral sex and rimming' in the same sex episode |
| 68.40 | 66.35 | 70.26 | %, proportion of saliva use during anal sex |
| 80.00 | 80.00 | 80.00 | %, proportion of receptive oral sex followed by partner's insertive anal sex |
| 80.00 | 80.00 | 80.00 | %, proportion of insertive oral sex followed by partner's insertive rimming |
| 1.83 | 1.23 | 2.36 | %, Infection prevalence of chlamydia trachomatis only at Oropharynx |
| 4.76 | 4.14 | 5.72 | %, Infection prevalence of chlamydia trachomatis only at Urethral |
| 11.86 | 10.54 | 12.57 | %, Infection prevalence of chlamydia trachomatis only at Rectum |
| 0.23 | 0.06 | 0.33 | %, Infection prevalence of chlamydia trachomatis at Oropharynx & Urethra |
| 1.30 | 0.83 | 1.61 | %, Infection prevalence of chlamydia trachomatis at Oropharynx & Rectum |
| 2.10 | 1.64 | 2.70 | %, Infection prevalence of chlamydia trachomatis at Urethra & Rectum |
| 0.25 | 0.10 | 0.37 | %, Infection prevalence of chlamydia trachomatis at Oropharynx & Urethra & Rectum |

**Table S8. 77.** Model 8’ output parameters with 95% confidence intervals

| **Mean** | **95%CI: lower** | **95%CI: upper** | **Parameters** |
| --- | --- | --- | --- |
| 45.83 | 37.47 | 53.52 | %, Consistent condom usage in anal sex in past 12 months |
| 86.14 | 82.00 | 90.94 | %, Condom efficacy in preventing transmission |
| - | - | - | Times, Frequency of kissing in the past 12 months |
| 17.70 | 3.35 | 25.79 | Times, Frequency of oral sex in the past 12 months |
| 38.56 | 16.89 | 55.54 | Times, Frequency of rimming in the past 12 months |
| 28.65 | 8.26 | 41.29 | Times, Frequency of anal sex in the past 12 months |
| 143.64 | 71.06 | 217.06 | weeks, Infection duration of chlamydia trachomatis at throat(asymptomatic infection) |
| 1.51 | 1.20 | 1.81 | weeks, Infection duration at urethral (symptomatic infection) |
| 100.00 | 100.00 | 100.00 | %, proportion of urethral infections that are asymptomatic |
| 100.00 | 100.00 | 100.00 | %, proportion of anal infections that are asymptomatic |
| 26.51 | 12.09 | 48.28 | weeks, Infection duration at urethral (asymptomatic infection) |
| 49.85 | 48.51 | 51.39 | weeks, Infection duration of chlamydia trachomatis at anus (asymptomatic infection) |
| 1.54 | 1.23 | 1.86 | weeks, Infection duration of chlamydia trachomatis at anus (symptomatic infection) |
| 83.41 | 72.01 | 92.75 | %, proportion of MSM received throat swab in the past 12 months |
| 75.90 | 68.08 | 85.55 | %, proportion of MSM received anal swab in the past 12 months |
| 81.84 | 70.01 | 90.87 | %, proportion of MSM received urine test in the past 12 months |
| 29.71 | 26.25 | 32.83 | %, proportion of 'oral sex and anal sex' in the same sex episode |
| 71.24 | 70.11 | 72.40 | %, proportion of 'oral sex and rimming' in the same sex episode |
| 68.18 | 66.62 | 70.14 | %, proportion of saliva use during anal sex |
| 80.00 | 80.00 | 80.00 | %, proportion of receptive oral sex followed by partner's insertive anal sex |
| 80.00 | 80.00 | 80.00 | %, proportion of insertive oral sex followed by partner's insertive rimming |
| 1.78 | 1.27 | 2.25 | %, Infection prevalence of chlamydia trachomatis only at Oropharynx |
| 4.95 | 4.39 | 5.68 | %, Infection prevalence of chlamydia trachomatis only at Urethral |
| 11.57 | 10.59 | 12.40 | %, Infection prevalence of chlamydia trachomatis only at Rectum |
| 0.21 | 0.08 | 0.32 | %, Infection prevalence of chlamydia trachomatis at Oropharynx & Urethra |
| 1.25 | 0.87 | 1.48 | %, Infection prevalence of chlamydia trachomatis at Oropharynx & Rectum |
| 2.07 | 1.69 | 2.70 | %, Infection prevalence of chlamydia trachomatis at Urethra & Rectum |
| 0.24 | 0.12 | 0.34 | %, Infection prevalence of chlamydia trachomatis at Oropharynx & Urethra & Rectum |

**Table S8. 78.** Model 9’ output parameters with 95% confidence intervals

| **Mean** | **95%CI: lower** | **95%CI: upper** | **Parameters** |
| --- | --- | --- | --- |
| 45.76 | 37.83 | 52.74 | %, Consistent condom usage in anal sex in past 12 months |
| 86.09 | 82.49 | 90.20 | %, Condom efficacy in preventing transmission |
| - | - | - | Times, Frequency of kissing in the past 12 months |
| 13.62 | 4.09 | 22.50 | Times, Frequency of oral sex in the past 12 months |
| 39.29 | 17.77 | 52.98 | Times, Frequency of rimming in the past 12 months |
| 21.33 | 10.05 | 39.88 | Times, Frequency of anal sex in the past 12 months |
| 135.26 | 72.39 | 211.60 | weeks, Infection duration of chlamydia trachomatis at throat(asymptomatic infection) |
| 1.45 | 1.26 | 1.78 | weeks, Infection duration at urethral (symptomatic infection) |
| 100.00 | 100.00 | 100.00 | %, proportion of urethral infections that are asymptomatic |
| 100.00 | 100.00 | 100.00 | %, proportion of anal infections that are asymptomatic |
| 27.92 | 12.79 | 42.15 | weeks, Infection duration at urethral (asymptomatic infection) |
| 50.50 | 48.64 | 51.32 | weeks, Infection duration of chlamydia trachomatis at anus (asymptomatic infection) |
| 1.55 | 1.29 | 1.82 | weeks, Infection duration of chlamydia trachomatis at anus (symptomatic infection) |
| 83.88 | 73.54 | 92.05 | %, proportion of MSM received throat swab in the past 12 months |
| 77.07 | 68.77 | 84.18 | %, proportion of MSM received anal swab in the past 12 months |
| 78.69 | 71.71 | 90.43 | %, proportion of MSM received urine test in the past 12 months |
| 29.28 | 26.48 | 32.73 | %, proportion of 'oral sex and anal sex' in the same sex episode |
| 71.31 | 70.30 | 72.16 | %, proportion of 'oral sex and rimming' in the same sex episode |
| 68.69 | 66.92 | 69.96 | %, proportion of saliva use during anal sex |
| 80.00 | 80.00 | 80.00 | %, proportion of receptive oral sex followed by partner's insertive anal sex |
| 80.00 | 80.00 | 80.00 | %, proportion of insertive oral sex followed by partner's insertive rimming |
| 1.79 | 1.37 | 2.21 | %, Infection prevalence of chlamydia trachomatis only at Oropharynx |
| 5.08 | 4.66 | 5.59 | %, Infection prevalence of chlamydia trachomatis only at Urethral |
| 11.74 | 10.81 | 12.24 | %, Infection prevalence of chlamydia trachomatis only at Rectum |
| 0.21 | 0.10 | 0.32 | %, Infection prevalence of chlamydia trachomatis at Oropharynx & Urethra |
| 1.15 | 0.91 | 1.47 | %, Infection prevalence of chlamydia trachomatis at Oropharynx & Rectum |
| 2.08 | 1.76 | 2.67 | %, Infection prevalence of chlamydia trachomatis at Urethra & Rectum |
| 0.23 | 0.13 | 0.33 | %, Infection prevalence of chlamydia trachomatis at Oropharynx & Urethra & Rectum |

**Table S8. 79.** Model 10’ output parameters with 95% confidence intervals

| **Mean** | **95%CI: lower** | **95%CI: upper** | **Parameters** |
| --- | --- | --- | --- |
| 43.16 | 38.07 | 52.21 | %, Consistent condom usage in anal sex in past 12 months |
| 85.59 | 82.73 | 89.55 | %, Condom efficacy in preventing transmission |
| - | - | - | Times, Frequency of kissing in the past 12 months |
| 13.06 | 4.87 | 21.74 | Times, Frequency of oral sex in the past 12 months |
| 34.90 | 19.25 | 50.57 | Times, Frequency of rimming in the past 12 months |
| 25.90 | 12.93 | 36.89 | Times, Frequency of anal sex in the past 12 months |
| 125.93 | 75.57 | 199.34 | weeks, Infection duration of chlamydia trachomatis at throat(asymptomatic infection) |
| 1.49 | 1.28 | 1.77 | weeks, Infection duration at urethral (symptomatic infection) |
| 100.00 | 100.00 | 100.00 | %, proportion of urethral infections that are asymptomatic |
| 100.00 | 100.00 | 100.00 | %, proportion of anal infections that are asymptomatic |
| 28.06 | 16.92 | 40.72 | weeks, Infection duration at urethral (asymptomatic infection) |
| 50.20 | 48.88 | 51.15 | weeks, Infection duration of chlamydia trachomatis at anus (asymptomatic infection) |
| 1.58 | 1.30 | 1.77 | weeks, Infection duration of chlamydia trachomatis at anus (symptomatic infection) |
| 82.54 | 73.75 | 89.70 | %, proportion of MSM received throat swab in the past 12 months |
| 77.73 | 70.05 | 83.96 | %, proportion of MSM received anal swab in the past 12 months |
| 82.92 | 73.44 | 88.63 | %, proportion of MSM received urine test in the past 12 months |
| 29.73 | 26.82 | 32.25 | %, proportion of 'oral sex and anal sex' in the same sex episode |
| 70.94 | 70.37 | 72.09 | %, proportion of 'oral sex and rimming' in the same sex episode |
| 68.63 | 67.17 | 69.93 | %, proportion of saliva use during anal sex |
| 80.00 | 80.00 | 80.00 | %, proportion of receptive oral sex followed by partner's insertive anal sex |
| 80.00 | 80.00 | 80.00 | %, proportion of insertive oral sex followed by partner's insertive rimming |
| 1.88 | 1.47 | 2.17 | %, Infection prevalence of chlamydia trachomatis only at Oropharynx |
| 5.04 | 4.73 | 5.54 | %, Infection prevalence of chlamydia trachomatis only at Urethral |
| 11.30 | 10.85 | 12.14 | %, Infection prevalence of chlamydia trachomatis only at Rectum |
| 0.21 | 0.13 | 0.31 | %, Infection prevalence of chlamydia trachomatis at Oropharynx & Urethra |
| 1.22 | 0.93 | 1.45 | %, Infection prevalence of chlamydia trachomatis at Oropharynx & Rectum |
| 2.07 | 1.77 | 2.65 | %, Infection prevalence of chlamydia trachomatis at Urethra & Rectum |
| 0.25 | 0.14 | 0.33 | %, Infection prevalence of chlamydia trachomatis at Oropharynx & Urethra & Rectum |

**Table S8. 80.** Model 11’ output parameters with 95% confidence intervals

| **Mean** | **95%CI: lower** | **95%CI: upper** | **Parameters** |
| --- | --- | --- | --- |
| 45.68 | 38.55 | 51.79 | %, Consistent condom usage in anal sex in past 12 months |
| 85.37 | 83.09 | 88.36 | %, Condom efficacy in preventing transmission |
| - | - | - | Times, Frequency of kissing in the past 12 months |
| 11.79 | 5.44 | 21.08 | Times, Frequency of oral sex in the past 12 months |
| 37.74 | 20.55 | 49.49 | Times, Frequency of rimming in the past 12 months |
| 23.92 | 14.29 | 33.66 | Times, Frequency of anal sex in the past 12 months |
| 153.13 | 78.39 | 200.57 | weeks, Infection duration of chlamydia trachomatis at throat(asymptomatic infection) |
| 1.53 | 1.31 | 1.75 | weeks, Infection duration at urethral (symptomatic infection) |
| 100.00 | 100.00 | 100.00 | %, proportion of urethral infections that are asymptomatic |
| 100.00 | 100.00 | 100.00 | %, proportion of anal infections that are asymptomatic |
| 29.64 | 17.27 | 41.05 | weeks, Infection duration at urethral (asymptomatic infection) |
| 50.11 | 49.20 | 51.07 | weeks, Infection duration of chlamydia trachomatis at anus (asymptomatic infection) |
| 1.57 | 1.34 | 1.74 | weeks, Infection duration of chlamydia trachomatis at anus (symptomatic infection) |
| 78.78 | 73.80 | 87.04 | %, proportion of MSM received throat swab in the past 12 months |
| 75.10 | 71.03 | 79.74 | %, proportion of MSM received anal swab in the past 12 months |
| 80.10 | 73.86 | 87.87 | %, proportion of MSM received urine test in the past 12 months |
| 30.01 | 27.43 | 31.91 | %, proportion of 'oral sex and anal sex' in the same sex episode |
| 71.23 | 70.49 | 71.98 | %, proportion of 'oral sex and rimming' in the same sex episode |
| 68.44 | 67.22 | 69.75 | %, proportion of saliva use during anal sex |
| 80.00 | 80.00 | 80.00 | %, proportion of receptive oral sex followed by partner's insertive anal sex |
| 80.00 | 80.00 | 80.00 | %, proportion of insertive oral sex followed by partner's insertive rimming |
| 1.78 | 1.56 | 2.14 | %, Infection prevalence of chlamydia trachomatis only at Oropharynx |
| 5.02 | 4.73 | 5.46 | %, Infection prevalence of chlamydia trachomatis only at Urethral |
| 11.34 | 10.88 | 11.97 | %, Infection prevalence of chlamydia trachomatis only at Rectum |
| 0.22 | 0.13 | 0.31 | %, Infection prevalence of chlamydia trachomatis at Oropharynx & Urethra |
| 1.09 | 0.93 | 1.39 | %, Infection prevalence of chlamydia trachomatis at Oropharynx & Rectum |
| 2.29 | 1.78 | 2.59 | %, Infection prevalence of chlamydia trachomatis at Urethra & Rectum |
| 0.24 | 0.16 | 0.32 | %, Infection prevalence of chlamydia trachomatis at Oropharynx & Urethra & Rectum |

**Table S8. 81.** Model 12’ output parameters with 95% confidence intervals

| **Mean** | **95%CI: lower** | **95%CI: upper** | **Parameters** |
| --- | --- | --- | --- |
| 45.37 | 39.68 | 50.58 | %, Consistent condom usage in anal sex in past 12 months |
| 85.45 | 83.62 | 87.52 | %, Condom efficacy in preventing transmission |
| - | - | - | Times, Frequency of kissing in the past 12 months |
| 15.15 | 6.17 | 20.59 | Times, Frequency of oral sex in the past 12 months |
| 36.42 | 25.44 | 48.05 | Times, Frequency of rimming in the past 12 months |
| 26.16 | 16.56 | 33.87 | Times, Frequency of anal sex in the past 12 months |
| 135.86 | 80.33 | 185.16 | weeks, Infection duration of chlamydia trachomatis at throat(asymptomatic infection) |
| 1.53 | 1.33 | 1.69 | weeks, Infection duration at urethral (symptomatic infection) |
| 100.00 | 100.00 | 100.00 | %, proportion of urethral infections that are asymptomatic |
| 100.00 | 100.00 | 100.00 | %, proportion of anal infections that are asymptomatic |
| 29.71 | 20.65 | 37.68 | weeks, Infection duration at urethral (asymptomatic infection) |
| 50.09 | 49.37 | 51.04 | weeks, Infection duration of chlamydia trachomatis at anus (asymptomatic infection) |
| 1.51 | 1.35 | 1.68 | weeks, Infection duration of chlamydia trachomatis at anus (symptomatic infection) |
| 79.20 | 74.04 | 85.64 | %, proportion of MSM received throat swab in the past 12 months |
| 75.49 | 71.82 | 79.45 | %, proportion of MSM received anal swab in the past 12 months |
| 79.71 | 74.20 | 86.50 | %, proportion of MSM received urine test in the past 12 months |
| 30.51 | 27.87 | 31.54 | %, proportion of 'oral sex and anal sex' in the same sex episode |
| 71.13 | 70.48 | 71.88 | %, proportion of 'oral sex and rimming' in the same sex episode |
| 68.71 | 67.48 | 69.55 | %, proportion of saliva use during anal sex |
| 80.00 | 80.00 | 80.00 | %, proportion of receptive oral sex followed by partner's insertive anal sex |
| 80.00 | 80.00 | 80.00 | %, proportion of insertive oral sex followed by partner's insertive rimming |
| 1.90 | 1.65 | 2.11 | %, Infection prevalence of chlamydia trachomatis only at Oropharynx |
| 5.12 | 4.83 | 5.42 | %, Infection prevalence of chlamydia trachomatis only at Urethral |
| 11.41 | 10.99 | 11.85 | %, Infection prevalence of chlamydia trachomatis only at Rectum |
| 0.22 | 0.15 | 0.30 | %, Infection prevalence of chlamydia trachomatis at Oropharynx & Urethra |
| 1.13 | 1.00 | 1.36 | %, Infection prevalence of chlamydia trachomatis at Oropharynx & Rectum |
| 2.22 | 1.86 | 2.57 | %, Infection prevalence of chlamydia trachomatis at Urethra & Rectum |
| 0.23 | 0.18 | 0.30 | %, Infection prevalence of chlamydia trachomatis at Oropharynx & Urethra & Rectum |

**Table S8. 82.** Model 13’ output parameters with 95% confidence intervals

| **Mean** | **95%CI: lower** | **95%CI: upper** | **Parameters** |
| --- | --- | --- | --- |
| 45.42 | 41.08 | 49.94 | %, Consistent condom usage in anal sex in past 12 months |
| 86.07 | 84.41 | 87.25 | %, Condom efficacy in preventing transmission |
| - | - | - | Times, Frequency of kissing in the past 12 months |
| 10.37 | 6.68 | 17.74 | Times, Frequency of oral sex in the past 12 months |
| 38.62 | 27.23 | 46.75 | Times, Frequency of rimming in the past 12 months |
| 24.62 | 19.03 | 32.44 | Times, Frequency of anal sex in the past 12 months |
| 124.48 | 84.50 | 175.88 | weeks, Infection duration of chlamydia trachomatis at throat(asymptomatic infection) |
| 1.44 | 1.37 | 1.66 | weeks, Infection duration at urethral (symptomatic infection) |
| 100.00 | 100.00 | 100.00 | %, proportion of urethral infections that are asymptomatic |
| 100.00 | 100.00 | 100.00 | %, proportion of anal infections that are asymptomatic |
| 31.33 | 21.58 | 36.45 | weeks, Infection duration at urethral (asymptomatic infection) |
| 50.27 | 49.44 | 50.95 | weeks, Infection duration of chlamydia trachomatis at anus (asymptomatic infection) |
| 1.51 | 1.38 | 1.64 | weeks, Infection duration of chlamydia trachomatis at anus (symptomatic infection) |
| 79.36 | 74.74 | 84.80 | %, proportion of MSM received throat swab in the past 12 months |
| 77.09 | 72.95 | 79.43 | %, proportion of MSM received anal swab in the past 12 months |
| 80.30 | 74.53 | 85.14 | %, proportion of MSM received urine test in the past 12 months |
| 29.77 | 28.18 | 31.44 | %, proportion of 'oral sex and anal sex' in the same sex episode |
| 71.06 | 70.47 | 71.75 | %, proportion of 'oral sex and rimming' in the same sex episode |
| 68.38 | 67.74 | 69.43 | %, proportion of saliva use during anal sex |
| 80.00 | 80.00 | 80.00 | %, proportion of receptive oral sex followed by partner's insertive anal sex |
| 80.00 | 80.00 | 80.00 | %, proportion of insertive oral sex followed by partner's insertive rimming |
| 1.79 | 1.67 | 2.07 | %, Infection prevalence of chlamydia trachomatis only at Oropharynx |
| 5.12 | 4.85 | 5.39 | %, Infection prevalence of chlamydia trachomatis only at Urethral |
| 11.46 | 11.07 | 11.85 | %, Infection prevalence of chlamydia trachomatis only at Rectum |
| 0.21 | 0.15 | 0.27 | %, Infection prevalence of chlamydia trachomatis at Oropharynx & Urethra |
| 1.12 | 1.00 | 1.32 | %, Infection prevalence of chlamydia trachomatis at Oropharynx & Rectum |
| 2.02 | 1.90 | 2.49 | %, Infection prevalence of chlamydia trachomatis at Urethra & Rectum |
| 0.22 | 0.18 | 0.29 | %, Infection prevalence of chlamydia trachomatis at Oropharynx & Urethra & Rectum |

Model output parameters (Published validation dataset 5): Data from 179 MSM with HIV in the USA

**Table S8. 83.** Model 2’ output parameters with 95% confidence intervals

| **Mean** | **95%CI: lower** | **95%CI: upper** | **Parameters** |
| --- | --- | --- | --- |
| 44.46 | 35.06 | 56.26 | %, Consistent condom usage in anal sex in past 12 months |
| 86.07 | 80.48 | 93.98 | %, Condom efficacy in preventing transmission |
| - | - | - | Times, Frequency of kissing in the past 12 months |
| 10.95 | 2.66 | 27.30 | Times, Frequency of oral sex in the past 12 months |
| 29.17 | 2.56 | 76.63 | Times, Frequency of rimming in the past 12 months |
| 13.73 | 1.72 | 48.68 | Times, Frequency of anal sex in the past 12 months |
| 125.37 | 34.04 | 232.11 | weeks, Infection duration of chlamydia trachomatis at throat(asymptomatic infection) |
| 1.37 | 1.05 | 1.97 | weeks, Infection duration at urethral (symptomatic infection) |
| 84.05 | 80.31 | 89.60 | %, proportion of urethral infections that are asymptomatic |
| 83.31 | 67.07 | 94.44 | %, proportion of anal infections that are asymptomatic |
| 27.95 | 4.02 | 51.02 | weeks, Infection duration at urethral (asymptomatic infection) |
| 49.80 | 48.43 | 51.67 | weeks, Infection duration of chlamydia trachomatis at anus (asymptomatic infection) |
| 1.38 | 1.07 | 1.84 | weeks, Infection duration of chlamydia trachomatis at anus (symptomatic infection) |
| 83.28 | 64.94 | 95.31 | %, proportion of MSM received throat swab in the past 12 months |
| 79.54 | 67.32 | 94.19 | %, proportion of MSM received anal swab in the past 12 months |
| 79.23 | 64.86 | 92.58 | %, proportion of MSM received urine test in the past 12 months |
| 28.47 | 25.71 | 32.79 | %, proportion of 'oral sex and anal sex' in the same sex episode |
| 71.08 | 69.81 | 72.67 | %, proportion of 'oral sex and rimming' in the same sex episode |
| 69.27 | 66.54 | 70.85 | %, proportion of saliva use during anal sex |
| 80.00 | 80.00 | 80.00 | %, proportion of receptive oral sex followed by partner's insertive anal sex |
| 80.00 | 80.00 | 80.00 | %, proportion of insertive oral sex followed by partner's insertive rimming |
| 0.00 | 0.00 | 0.00 | %, Infection prevalence of chlamydia trachomatis only at Oropharynx |
| 2.97 | 0.66 | 4.77 | %, Infection prevalence of chlamydia trachomatis only at Urethral |
| 13.21 | 9.17 | 18.54 | %, Infection prevalence of chlamydia trachomatis only at Rectum |
| 0.00 | 0.00 | 0.00 | %, Infection prevalence of chlamydia trachomatis at Oropharynx & Urethra |
| 0.00 | 0.00 | 0.00 | %, Infection prevalence of chlamydia trachomatis at Oropharynx & Rectum |
| 2.36 | 0.82 | 4.09 | %, Infection prevalence of chlamydia trachomatis at Urethra & Rectum |
| 0.00 | 0.00 | 0.00 | %, Infection prevalence of chlamydia trachomatis at Oropharynx & Urethra & Rectum |

**Table S8. 84.** Model 7’ output parameters with 95% confidence intervals

| **Mean** | **95%CI: lower** | **95%CI: upper** | **Parameters** |
| --- | --- | --- | --- |
| 47.59 | 35.43 | 54.67 | %, Consistent condom usage in anal sex in past 12 months |
| 86.78 | 80.95 | 92.42 | %, Condom efficacy in preventing transmission |
| - | - | - | Times, Frequency of kissing in the past 12 months |
| 11.68 | 2.82 | 23.60 | Times, Frequency of oral sex in the past 12 months |
| 38.29 | 5.57 | 73.47 | Times, Frequency of rimming in the past 12 months |
| 13.30 | 2.30 | 42.30 | Times, Frequency of anal sex in the past 12 months |
| 142.31 | 58.87 | 226.91 | weeks, Infection duration of chlamydia trachomatis at throat(asymptomatic infection) |
| 1.43 | 1.06 | 1.94 | weeks, Infection duration at urethral (symptomatic infection) |
| 84.49 | 80.85 | 89.00 | %, proportion of urethral infections that are asymptomatic |
| 81.11 | 70.34 | 92.63 | %, proportion of anal infections that are asymptomatic |
| 30.34 | 7.00 | 51.01 | weeks, Infection duration at urethral (asymptomatic infection) |
| 50.60 | 48.48 | 51.51 | weeks, Infection duration of chlamydia trachomatis at anus (asymptomatic infection) |
| 1.45 | 1.21 | 1.81 | weeks, Infection duration of chlamydia trachomatis at anus (symptomatic infection) |
| 80.61 | 67.86 | 90.55 | %, proportion of MSM received throat swab in the past 12 months |
| 81.23 | 68.93 | 91.22 | %, proportion of MSM received anal swab in the past 12 months |
| 81.23 | 69.77 | 91.08 | %, proportion of MSM received urine test in the past 12 months |
| 28.61 | 26.10 | 31.79 | %, proportion of 'oral sex and anal sex' in the same sex episode |
| 71.57 | 70.25 | 72.53 | %, proportion of 'oral sex and rimming' in the same sex episode |
| 68.50 | 66.71 | 70.57 | %, proportion of saliva use during anal sex |
| 80.00 | 80.00 | 80.00 | %, proportion of receptive oral sex followed by partner's insertive anal sex |
| 80.00 | 80.00 | 80.00 | %, proportion of insertive oral sex followed by partner's insertive rimming |
| 0.00 | 0.00 | 0.00 | %, Infection prevalence of chlamydia trachomatis only at Oropharynx |
| 2.75 | 0.69 | 4.51 | %, Infection prevalence of chlamydia trachomatis only at Urethral |
| 12.37 | 9.43 | 17.89 | %, Infection prevalence of chlamydia trachomatis only at Rectum |
| 0.00 | 0.00 | 0.00 | %, Infection prevalence of chlamydia trachomatis at Oropharynx & Urethra |
| 0.00 | 0.00 | 0.00 | %, Infection prevalence of chlamydia trachomatis at Oropharynx & Rectum |
| 2.65 | 1.24 | 3.88 | %, Infection prevalence of chlamydia trachomatis at Urethra & Rectum |
| 0.00 | 0.00 | 0.00 | %, Infection prevalence of chlamydia trachomatis at Oropharynx & Urethra & Rectum |

**Table S8. 85.** Model 8’ output parameters with 95% confidence intervals

| **Mean** | **95%CI: lower** | **95%CI: upper** | **Parameters** |
| --- | --- | --- | --- |
| 46.56 | 38.86 | 54.00 | %, Consistent condom usage in anal sex in past 12 months |
| 86.05 | 81.27 | 92.24 | %, Condom efficacy in preventing transmission |
| - | - | - | Times, Frequency of kissing in the past 12 months |
| 16.81 | 4.05 | 23.31 | Times, Frequency of oral sex in the past 12 months |
| 36.95 | 9.47 | 71.97 | Times, Frequency of rimming in the past 12 months |
| 19.02 | 8.33 | 40.43 | Times, Frequency of anal sex in the past 12 months |
| 138.57 | 64.69 | 214.15 | weeks, Infection duration of chlamydia trachomatis at throat(asymptomatic infection) |
| 1.48 | 1.14 | 1.87 | weeks, Infection duration at urethral (symptomatic infection) |
| 84.96 | 80.98 | 88.95 | %, proportion of urethral infections that are asymptomatic |
| 82.30 | 72.47 | 91.90 | %, proportion of anal infections that are asymptomatic |
| 33.63 | 10.85 | 49.72 | weeks, Infection duration at urethral (asymptomatic infection) |
| 50.24 | 49.11 | 51.24 | weeks, Infection duration of chlamydia trachomatis at anus (asymptomatic infection) |
| 1.50 | 1.24 | 1.79 | weeks, Infection duration of chlamydia trachomatis at anus (symptomatic infection) |
| 75.78 | 69.85 | 90.63 | %, proportion of MSM received throat swab in the past 12 months |
| 77.77 | 72.12 | 88.85 | %, proportion of MSM received anal swab in the past 12 months |
| 79.42 | 70.68 | 89.73 | %, proportion of MSM received urine test in the past 12 months |
| 29.02 | 26.26 | 30.83 | %, proportion of 'oral sex and anal sex' in the same sex episode |
| 71.69 | 70.44 | 72.47 | %, proportion of 'oral sex and rimming' in the same sex episode |
| 68.82 | 66.84 | 70.40 | %, proportion of saliva use during anal sex |
| 80.00 | 80.00 | 80.00 | %, proportion of receptive oral sex followed by partner's insertive anal sex |
| 80.00 | 80.00 | 80.00 | %, proportion of insertive oral sex followed by partner's insertive rimming |
| 0.00 | 0.00 | 0.00 | %, Infection prevalence of chlamydia trachomatis only at Oropharynx |
| 2.58 | 0.73 | 4.11 | %, Infection prevalence of chlamydia trachomatis only at Urethral |
| 13.82 | 10.22 | 16.84 | %, Infection prevalence of chlamydia trachomatis only at Rectum |
| 0.00 | 0.00 | 0.00 | %, Infection prevalence of chlamydia trachomatis at Oropharynx & Urethra |
| 0.00 | 0.00 | 0.00 | %, Infection prevalence of chlamydia trachomatis at Oropharynx & Rectum |
| 2.58 | 1.62 | 3.61 | %, Infection prevalence of chlamydia trachomatis at Urethra & Rectum |
| 0.00 | 0.00 | 0.00 | %, Infection prevalence of chlamydia trachomatis at Oropharynx & Urethra & Rectum |

**Table S8. 86**. Model 9’ output parameters with 95% confidence intervals

| **Mean** | **95%CI: lower** | **95%CI: upper** | **Parameters** |
| --- | --- | --- | --- |
| 44.74 | 39.08 | 52.67 | %, Consistent condom usage in anal sex in past 12 months |
| 86.61 | 82.62 | 91.26 | %, Condom efficacy in preventing transmission |
| - | - | - | Times, Frequency of kissing in the past 12 months |
| 12.95 | 7.12 | 21.26 | Times, Frequency of oral sex in the past 12 months |
| 53.09 | 16.66 | 72.02 | Times, Frequency of rimming in the past 12 months |
| 12.29 | 8.85 | 22.92 | Times, Frequency of anal sex in the past 12 months |
| 118.76 | 80.72 | 207.86 | weeks, Infection duration of chlamydia trachomatis at throat(asymptomatic infection) |
| 1.57 | 1.32 | 1.85 | weeks, Infection duration at urethral (symptomatic infection) |
| 84.17 | 81.44 | 87.51 | %, proportion of urethral infections that are asymptomatic |
| 85.03 | 74.30 | 90.87 | %, proportion of anal infections that are asymptomatic |
| 25.44 | 9.01 | 43.17 | weeks, Infection duration at urethral (asymptomatic infection) |
| 50.07 | 49.15 | 51.13 | weeks, Infection duration of chlamydia trachomatis at anus (asymptomatic infection) |
| 1.49 | 1.26 | 1.75 | weeks, Infection duration of chlamydia trachomatis at anus (symptomatic infection) |
| 79.10 | 72.52 | 87.45 | %, proportion of MSM received throat swab in the past 12 months |
| 77.71 | 73.09 | 84.49 | %, proportion of MSM received anal swab in the past 12 months |
| 79.81 | 71.80 | 88.12 | %, proportion of MSM received urine test in the past 12 months |
| 28.84 | 26.48 | 30.35 | %, proportion of 'oral sex and anal sex' in the same sex episode |
| 71.62 | 70.73 | 72.33 | %, proportion of 'oral sex and rimming' in the same sex episode |
| 68.74 | 67.07 | 70.10 | %, proportion of saliva use during anal sex |
| 80.00 | 80.00 | 80.00 | %, proportion of receptive oral sex followed by partner's insertive anal sex |
| 80.00 | 80.00 | 80.00 | %, proportion of insertive oral sex followed by partner's insertive rimming |
| 0.00 | 0.00 | 0.00 | %, Infection prevalence of chlamydia trachomatis only at Oropharynx |
| 2.48 | 1.29 | 4.03 | %, Infection prevalence of chlamydia trachomatis only at Urethral |
| 13.22 | 11.15 | 16.67 | %, Infection prevalence of chlamydia trachomatis only at Rectum |
| 0.00 | 0.00 | 0.00 | %, Infection prevalence of chlamydia trachomatis at Oropharynx & Urethra |
| 0.00 | 0.00 | 0.00 | %, Infection prevalence of chlamydia trachomatis at Oropharynx & Rectum |
| 2.88 | 1.78 | 3.45 | %, Infection prevalence of chlamydia trachomatis at Urethra & Rectum |
| 0.00 | 0.00 | 0.00 | %, Infection prevalence of chlamydia trachomatis at Oropharynx & Urethra & Rectum |

**Table S8. 87.** Model 10’ output parameters with 95% confidence intervals

| **Mean** | **95%CI: lower** | **95%CI: upper** | **Parameters** |
| --- | --- | --- | --- |
| 46.28 | 40.56 | 51.84 | %, Consistent condom usage in anal sex in past 12 months |
| 85.70 | 83.15 | 90.33 | %, Condom efficacy in preventing transmission |
| - | - | - | Times, Frequency of kissing in the past 12 months |
| 14.82 | 9.35 | 20.65 | Times, Frequency of oral sex in the past 12 months |
| 47.58 | 22.73 | 69.12 | Times, Frequency of rimming in the past 12 months |
| 14.98 | 9.53 | 20.64 | Times, Frequency of anal sex in the past 12 months |
| 153.91 | 95.53 | 198.26 | weeks, Infection duration of chlamydia trachomatis at throat(asymptomatic infection) |
| 1.55 | 1.34 | 1.80 | weeks, Infection duration at urethral (symptomatic infection) |
| 84.01 | 81.73 | 86.13 | %, proportion of urethral infections that are asymptomatic |
| 84.48 | 74.83 | 89.45 | %, proportion of anal infections that are asymptomatic |
| 29.01 | 9.56 | 38.70 | weeks, Infection duration at urethral (asymptomatic infection) |
| 50.30 | 49.35 | 50.92 | weeks, Infection duration of chlamydia trachomatis at anus (asymptomatic infection) |
| 1.47 | 1.28 | 1.71 | weeks, Infection duration of chlamydia trachomatis at anus (symptomatic infection) |
| 80.59 | 75.21 | 86.86 | %, proportion of MSM received throat swab in the past 12 months |
| 78.86 | 73.59 | 83.59 | %, proportion of MSM received anal swab in the past 12 months |
| 80.16 | 73.66 | 87.01 | %, proportion of MSM received urine test in the past 12 months |
| 28.31 | 27.01 | 30.00 | %, proportion of 'oral sex and anal sex' in the same sex episode |
| 71.64 | 70.85 | 72.30 | %, proportion of 'oral sex and rimming' in the same sex episode |
| 68.90 | 67.60 | 69.97 | %, proportion of saliva use during anal sex |
| 80.00 | 80.00 | 80.00 | %, proportion of receptive oral sex followed by partner's insertive anal sex |
| 80.00 | 80.00 | 80.00 | %, proportion of insertive oral sex followed by partner's insertive rimming |
| 0.00 | 0.00 | 0.00 | %, Infection prevalence of chlamydia trachomatis only at Oropharynx |
| 2.51 | 1.54 | 3.62 | %, Infection prevalence of chlamydia trachomatis only at Urethral |
| 14.86 | 12.14 | 16.17 | %, Infection prevalence of chlamydia trachomatis only at Rectum |
| 0.00 | 0.00 | 0.00 | %, Infection prevalence of chlamydia trachomatis at Oropharynx & Urethra |
| 0.00 | 0.00 | 0.00 | %, Infection prevalence of chlamydia trachomatis at Oropharynx & Rectum |
| 2.79 | 1.91 | 3.29 | %, Infection prevalence of chlamydia trachomatis at Urethra & Rectum |
| 0.00 | 0.00 | 0.00 | %, Infection prevalence of chlamydia trachomatis at Oropharynx & Urethra & Rectum |

**Table S8. 88.** Model 11’ output parameters with 95% confidence intervals

| **Mean** | **95%CI: lower** | **95%CI: upper** | **Parameters** |
| --- | --- | --- | --- |
| 43.64 | 40.65 | 50.82 | %, Consistent condom usage in anal sex in past 12 months |
| 86.15 | 83.61 | 89.78 | %, Condom efficacy in preventing transmission |
| - | - | - | Times, Frequency of kissing in the past 12 months |
| 15.30 | 11.38 | 19.96 | Times, Frequency of oral sex in the past 12 months |
| 48.79 | 24.30 | 68.69 | Times, Frequency of rimming in the past 12 months |
| 12.97 | 9.61 | 18.47 | Times, Frequency of anal sex in the past 12 months |
| 139.65 | 98.06 | 191.93 | weeks, Infection duration of chlamydia trachomatis at throat(asymptomatic infection) |
| 1.51 | 1.38 | 1.79 | weeks, Infection duration at urethral (symptomatic infection) |
| 83.78 | 81.91 | 85.75 | %, proportion of urethral infections that are asymptomatic |
| 82.94 | 76.24 | 89.04 | %, proportion of anal infections that are asymptomatic |
| 20.66 | 9.62 | 37.56 | weeks, Infection duration at urethral (asymptomatic infection) |
| 50.18 | 49.36 | 50.76 | weeks, Infection duration of chlamydia trachomatis at anus (asymptomatic infection) |
| 1.47 | 1.29 | 1.68 | weeks, Infection duration of chlamydia trachomatis at anus (symptomatic infection) |
| 81.04 | 75.89 | 86.51 | %, proportion of MSM received throat swab in the past 12 months |
| 79.12 | 74.68 | 83.13 | %, proportion of MSM received anal swab in the past 12 months |
| 80.00 | 74.31 | 86.39 | %, proportion of MSM received urine test in the past 12 months |
| 28.36 | 27.02 | 29.75 | %, proportion of 'oral sex and anal sex' in the same sex episode |
| 71.83 | 70.98 | 72.21 | %, proportion of 'oral sex and rimming' in the same sex episode |
| 68.79 | 67.63 | 69.78 | %, proportion of saliva use during anal sex |
| 80.00 | 80.00 | 80.00 | %, proportion of receptive oral sex followed by partner's insertive anal sex |
| 80.00 | 80.00 | 80.00 | %, proportion of insertive oral sex followed by partner's insertive rimming |
| 0.00 | 0.00 | 0.00 | %, Infection prevalence of chlamydia trachomatis only at Oropharynx |
| 2.54 | 1.61 | 3.59 | %, Infection prevalence of chlamydia trachomatis only at Urethral |
| 14.02 | 12.86 | 15.50 | %, Infection prevalence of chlamydia trachomatis only at Rectum |
| 0.00 | 0.00 | 0.00 | %, Infection prevalence of chlamydia trachomatis at Oropharynx & Urethra |
| 0.00 | 0.00 | 0.00 | %, Infection prevalence of chlamydia trachomatis at Oropharynx & Rectum |
| 2.66 | 1.91 | 3.13 | %, Infection prevalence of chlamydia trachomatis at Urethra & Rectum |
| 0.00 | 0.00 | 0.00 | %, Infection prevalence of chlamydia trachomatis at Oropharynx & Urethra & Rectum |

**Table S8. 89.** Model 12’ output parameters with 95% confidence intervals

| **Mean** | **95%CI: lower** | **95%CI: upper** | **Parameters** |
| --- | --- | --- | --- |
| 43.72 | 40.94 | 49.65 | %, Consistent condom usage in anal sex in past 12 months |
| 86.22 | 83.97 | 89.23 | %, Condom efficacy in preventing transmission |
| - | - | - | Times, Frequency of kissing in the past 12 months |
| 17.15 | 12.11 | 19.55 | Times, Frequency of oral sex in the past 12 months |
| 43.72 | 27.90 | 68.01 | Times, Frequency of rimming in the past 12 months |
| 12.52 | 10.13 | 17.07 | Times, Frequency of anal sex in the past 12 months |
| 139.65 | 103.51 | 188.23 | weeks, Infection duration of chlamydia trachomatis at throat(asymptomatic infection) |
| 1.60 | 1.40 | 1.79 | weeks, Infection duration at urethral (symptomatic infection) |
| 83.34 | 82.13 | 85.24 | %, proportion of urethral infections that are asymptomatic |
| 84.44 | 79.08 | 88.70 | %, proportion of anal infections that are asymptomatic |
| 19.62 | 9.69 | 33.91 | weeks, Infection duration at urethral (asymptomatic infection) |
| 50.07 | 49.37 | 50.65 | weeks, Infection duration of chlamydia trachomatis at anus (asymptomatic infection) |
| 1.47 | 1.29 | 1.67 | weeks, Infection duration of chlamydia trachomatis at anus (symptomatic infection) |
| 81.65 | 76.76 | 86.29 | %, proportion of MSM received throat swab in the past 12 months |
| 78.00 | 74.84 | 82.22 | %, proportion of MSM received anal swab in the past 12 months |
| 79.69 | 74.70 | 85.72 | %, proportion of MSM received urine test in the past 12 months |
| 27.95 | 27.04 | 29.44 | %, proportion of 'oral sex and anal sex' in the same sex episode |
| 71.86 | 71.26 | 72.18 | %, proportion of 'oral sex and rimming' in the same sex episode |
| 68.82 | 67.70 | 69.61 | %, proportion of saliva use during anal sex |
| 80.00 | 80.00 | 80.00 | %, proportion of receptive oral sex followed by partner's insertive anal sex |
| 80.00 | 80.00 | 80.00 | %, proportion of insertive oral sex followed by partner's insertive rimming |
| 0.00 | 0.00 | 0.00 | %, Infection prevalence of chlamydia trachomatis only at Oropharynx |
| 2.59 | 1.83 | 3.49 | %, Infection prevalence of chlamydia trachomatis only at Urethral |
| 14.23 | 13.05 | 15.36 | %, Infection prevalence of chlamydia trachomatis only at Rectum |
| 0.00 | 0.00 | 0.00 | %, Infection prevalence of chlamydia trachomatis at Oropharynx & Urethra |
| 0.00 | 0.00 | 0.00 | %, Infection prevalence of chlamydia trachomatis at Oropharynx & Rectum |
| 2.48 | 1.98 | 3.08 | %, Infection prevalence of chlamydia trachomatis at Urethra & Rectum |
| 0.00 | 0.00 | 0.00 | %, Infection prevalence of chlamydia trachomatis at Oropharynx & Urethra & Rectum |

**Table S8. 90.** Model 13’ output parameters with 95% confidence intervals

| **Mean** | **95%CI: lower** | **95%CI: upper** | **Parameters** |
| --- | --- | --- | --- |
| 44.47 | 41.53 | 49.15 | %, Consistent condom usage in anal sex in past 12 months |
| 86.52 | 84.30 | 88.91 | %, Condom efficacy in preventing transmission |
| - | - | - | Times, Frequency of kissing in the past 12 months |
| 15.88 | 12.51 | 19.20 | Times, Frequency of oral sex in the past 12 months |
| 43.99 | 28.81 | 66.65 | Times, Frequency of rimming in the past 12 months |
| 12.63 | 10.54 | 16.53 | Times, Frequency of anal sex in the past 12 months |
| 142.77 | 110.19 | 179.86 | weeks, Infection duration of chlamydia trachomatis at throat(asymptomatic infection) |
| 1.62 | 1.42 | 1.78 | weeks, Infection duration at urethral (symptomatic infection) |
| 83.59 | 82.25 | 85.04 | %, proportion of urethral infections that are asymptomatic |
| 85.23 | 79.26 | 88.56 | %, proportion of anal infections that are asymptomatic |
| 22.98 | 10.61 | 31.93 | weeks, Infection duration at urethral (asymptomatic infection) |
| 50.06 | 49.41 | 50.60 | weeks, Infection duration of chlamydia trachomatis at anus (asymptomatic infection) |
| 1.46 | 1.30 | 1.65 | weeks, Infection duration of chlamydia trachomatis at anus (symptomatic infection) |
| 81.34 | 77.71 | 85.64 | %, proportion of MSM received throat swab in the past 12 months |
| 78.63 | 75.91 | 81.94 | %, proportion of MSM received anal swab in the past 12 months |
| 80.37 | 74.84 | 85.19 | %, proportion of MSM received urine test in the past 12 months |
| 28.23 | 27.12 | 29.33 | %, proportion of 'oral sex and anal sex' in the same sex episode |
| 71.70 | 71.33 | 72.14 | %, proportion of 'oral sex and rimming' in the same sex episode |
| 68.73 | 67.81 | 69.54 | %, proportion of saliva use during anal sex |
| 80.00 | 80.00 | 80.00 | %, proportion of receptive oral sex followed by partner's insertive anal sex |
| 80.00 | 80.00 | 80.00 | %, proportion of insertive oral sex followed by partner's insertive rimming |
| 0.00 | 0.00 | 0.00 | %, Infection prevalence of chlamydia trachomatis only at Oropharynx |
| 2.57 | 1.88 | 3.39 | %, Infection prevalence of chlamydia trachomatis only at Urethral |
| 14.08 | 13.32 | 14.85 | %, Infection prevalence of chlamydia trachomatis only at Rectum |
| 0.00 | 0.00 | 0.00 | %, Infection prevalence of chlamydia trachomatis at Oropharynx & Urethra |
| 0.00 | 0.00 | 0.00 | %, Infection prevalence of chlamydia trachomatis at Oropharynx & Rectum |
| 2.52 | 2.00 | 2.99 | %, Infection prevalence of chlamydia trachomatis at Urethra & Rectum |
| 0.00 | 0.00 | 0.00 | %, Infection prevalence of chlamydia trachomatis at Oropharynx & Urethra & Rectum |

‘Anal sex, oral sex, rimming, kissing and sequential sexual practices’ transmission models (Model 3,14-20)

Model output parameters (Calibration Dataset): Unpublished calibration data from 4888 MSM attending Melbourne Sexual Health Centre

**Table S8. 91.** Model 3’ output parameters with 95% confidence intervals

| **Mean** | **95%CI: lower** | **95%CI: upper** | **Parameters** |
| --- | --- | --- | --- |
| 45.36 | 35.44 | 56.03 | %, Consistent condom usage in anal sex in past 12 months |
| 89.18 | 80.65 | 93.70 | %, Condom efficacy in preventing transmission |
| 5.07 | 0.84 | 12.10 | Times, Frequency of kissing in the past 12 months |
| 15.14 | 2.49 | 27.82 | Times, Frequency of oral sex in the past 12 months |
| 30.29 | 3.04 | 75.07 | Times, Frequency of rimming in the past 12 months |
| 16.99 | 2.11 | 47.78 | Times, Frequency of anal sex in the past 12 months |
| 119.84 | 31.07 | 210.10 | weeks, Infection duration of chlamydia trachomatis at throat(asymptomatic infection) |
| 1.38 | 1.04 | 1.94 | weeks, Infection duration at urethral (symptomatic infection) |
| 84.52 | 81.26 | 89.33 | %, proportion of urethral infections that are asymptomatic |
| 81.53 | 66.81 | 95.69 | %, proportion of anal infections that are asymptomatic |
| 22.64 | 7.23 | 50.70 | weeks, Infection duration at urethral (asymptomatic infection) |
| 50.02 | 48.43 | 51.75 | weeks, Infection duration of chlamydia trachomatis at anus (asymptomatic infection) |
| 1.51 | 1.03 | 1.99 | weeks, Infection duration of chlamydia trachomatis at anus (symptomatic infection) |
| 81.74 | 65.60 | 94.05 | %, proportion of MSM received throat swab in the past 12 months |
| 77.50 | 64.47 | 92.09 | %, proportion of MSM received anal swab in the past 12 months |
| 80.67 | 67.25 | 92.54 | %, proportion of MSM received urine test in the past 12 months |
| 28.21 | 24.98 | 33.36 | %, proportion of 'oral sex and anal sex' in the same sex episode |
| 71.08 | 69.83 | 72.56 | %, proportion of 'oral sex and rimming' in the same sex episode |
| 68.72 | 66.88 | 70.73 | %, proportion of saliva use during anal sex |
| 80.00 | 80.00 | 80.00 | %, proportion of receptive oral sex followed by partner's insertive anal sex |
| 80.00 | 80.00 | 80.00 | %, proportion of insertive oral sex followed by partner's insertive rimming |
| 0.83 | 0.63 | 1.01 | %, Infection prevalence of chlamydia trachomatis only at Oropharynx |
| 1.87 | 1.54 | 2.20 | %, Infection prevalence of chlamydia trachomatis only at Urethral |
| 7.59 | 6.85 | 8.11 | %, Infection prevalence of chlamydia trachomatis only at Rectum |
| 0.00 | 0.00 | 0.00 | %, Infection prevalence of chlamydia trachomatis at Oropharynx & Urethra |
| 1.14 | 0.93 | 1.47 | %, Infection prevalence of chlamydia trachomatis at Oropharynx & Rectum |
| 1.16 | 0.94 | 1.46 | %, Infection prevalence of chlamydia trachomatis at Urethra & Rectum |
| 0.20 | 0.09 | 0.30 | %, Infection prevalence of chlamydia trachomatis at Oropharynx & Urethra & Rectum |

**Table S8. 92**. Model 14’ output parameters with 95% confidence intervals

| **Mean** | **95%CI: lower** | **95%CI: upper** | **Parameters** |
| --- | --- | --- | --- |
| 45.59 | 36.60 | 55.43 | %, Consistent condom usage in anal sex in past 12 months |
| 86.54 | 81.14 | 93.06 | %, Condom efficacy in preventing transmission |
| 4.95 | 1.53 | 11.09 | Times, Frequency of kissing in the past 12 months |
| 15.99 | 7.20 | 26.55 | Times, Frequency of oral sex in the past 12 months |
| 46.48 | 10.78 | 73.06 | Times, Frequency of rimming in the past 12 months |
| 8.49 | 3.24 | 21.94 | Times, Frequency of anal sex in the past 12 months |
| 144.60 | 65.20 | 200.17 | weeks, Infection duration of chlamydia trachomatis at throat(asymptomatic infection) |
| 1.61 | 1.20 | 1.91 | weeks, Infection duration at urethral (symptomatic infection) |
| 85.33 | 82.18 | 88.66 | %, proportion of urethral infections that are asymptomatic |
| 83.89 | 71.75 | 94.66 | %, proportion of anal infections that are asymptomatic |
| 16.54 | 8.36 | 41.67 | weeks, Infection duration at urethral (asymptomatic infection) |
| 49.91 | 48.54 | 51.62 | weeks, Infection duration of chlamydia trachomatis at anus (asymptomatic infection) |
| 1.56 | 1.08 | 1.96 | weeks, Infection duration of chlamydia trachomatis at anus (symptomatic infection) |
| 78.76 | 67.78 | 92.02 | %, proportion of MSM received throat swab in the past 12 months |
| 79.81 | 71.17 | 89.48 | %, proportion of MSM received anal swab in the past 12 months |
| 81.32 | 69.79 | 91.32 | %, proportion of MSM received urine test in the past 12 months |
| 29.26 | 25.47 | 32.81 | %, proportion of 'oral sex and anal sex' in the same sex episode |
| 71.75 | 70.10 | 72.49 | %, proportion of 'oral sex and rimming' in the same sex episode |
| 69.07 | 67.51 | 70.67 | %, proportion of saliva use during anal sex |
| 80.00 | 80.00 | 80.00 | %, proportion of receptive oral sex followed by partner's insertive anal sex |
| 80.00 | 80.00 | 80.00 | %, proportion of insertive oral sex followed by partner's insertive rimming |
| 0.79 | 0.64 | 0.95 | %, Infection prevalence of chlamydia trachomatis only at Oropharynx |
| 1.92 | 1.55 | 2.11 | %, Infection prevalence of chlamydia trachomatis only at Urethral |
| 7.69 | 6.97 | 8.00 | %, Infection prevalence of chlamydia trachomatis only at Rectum |
| 0.00 | 0.00 | 0.00 | %, Infection prevalence of chlamydia trachomatis at Oropharynx & Urethra |
| 1.13 | 0.99 | 1.45 | %, Infection prevalence of chlamydia trachomatis at Oropharynx & Rectum |
| 1.21 | 0.97 | 1.45 | %, Infection prevalence of chlamydia trachomatis at Urethra & Rectum |
| 0.18 | 0.11 | 0.30 | %, Infection prevalence of chlamydia trachomatis at Oropharynx & Urethra & Rectum |

**Table S8. 93.** Model 15’ output parameters with 95% confidence intervals

| **Mean** | **95%CI: lower** | **95%CI: upper** | **Parameters** |
| --- | --- | --- | --- |
| 48.20 | 39.71 | 55.20 | %, Consistent condom usage in anal sex in past 12 months |
| 88.92 | 83.58 | 92.45 | %, Condom efficacy in preventing transmission |
| 6.33 | 1.77 | 10.55 | Times, Frequency of kissing in the past 12 months |
| 17.04 | 7.79 | 24.87 | Times, Frequency of oral sex in the past 12 months |
| 38.76 | 13.35 | 66.49 | Times, Frequency of rimming in the past 12 months |
| 12.22 | 4.51 | 20.64 | Times, Frequency of anal sex in the past 12 months |
| 136.20 | 72.53 | 186.88 | weeks, Infection duration of chlamydia trachomatis at throat(asymptomatic infection) |
| 1.53 | 1.20 | 1.87 | weeks, Infection duration at urethral (symptomatic infection) |
| 85.19 | 82.44 | 88.26 | %, proportion of urethral infections that are asymptomatic |
| 84.45 | 73.79 | 93.32 | %, proportion of anal infections that are asymptomatic |
| 16.26 | 8.63 | 33.82 | weeks, Infection duration at urethral (asymptomatic infection) |
| 49.85 | 48.59 | 51.32 | weeks, Infection duration of chlamydia trachomatis at anus (asymptomatic infection) |
| 1.59 | 1.18 | 1.96 | weeks, Infection duration of chlamydia trachomatis at anus (symptomatic infection) |
| 83.19 | 68.84 | 91.82 | %, proportion of MSM received throat swab in the past 12 months |
| 80.02 | 71.63 | 88.72 | %, proportion of MSM received anal swab in the past 12 months |
| 82.46 | 72.38 | 91.39 | %, proportion of MSM received urine test in the past 12 months |
| 29.21 | 25.79 | 32.46 | %, proportion of 'oral sex and anal sex' in the same sex episode |
| 71.38 | 70.32 | 72.40 | %, proportion of 'oral sex and rimming' in the same sex episode |
| 68.75 | 67.60 | 70.53 | %, proportion of saliva use during anal sex |
| 80.00 | 80.00 | 80.00 | %, proportion of receptive oral sex followed by partner's insertive anal sex |
| 80.00 | 80.00 | 80.00 | %, proportion of insertive oral sex followed by partner's insertive rimming |
| 0.84 | 0.64 | 0.95 | %, Infection prevalence of chlamydia trachomatis only at Oropharynx |
| 1.79 | 1.58 | 2.08 | %, Infection prevalence of chlamydia trachomatis only at Urethral |
| 7.47 | 7.00 | 7.94 | %, Infection prevalence of chlamydia trachomatis only at Rectum |
| 0.00 | 0.00 | 0.00 | %, Infection prevalence of chlamydia trachomatis at Oropharynx & Urethra |
| 1.22 | 1.03 | 1.40 | %, Infection prevalence of chlamydia trachomatis at Oropharynx & Rectum |
| 1.31 | 0.99 | 1.44 | %, Infection prevalence of chlamydia trachomatis at Urethra & Rectum |
| 0.20 | 0.11 | 0.29 | %, Infection prevalence of chlamydia trachomatis at Oropharynx & Urethra & Rectum |

**Table S8. 94.** Model 16’ output parameters with 95% confidence intervals

| **Mean** | **95%CI: lower** | **95%CI: upper** | **Parameters** |
| --- | --- | --- | --- |
| 48.05 | 35.61 | 57.80 | %, Consistent condom usage in anal sex in past 12 months |
| 87.38 | 81.02 | 93.94 | %, Condom efficacy in preventing transmission |
| 6.91 | 1.31 | 12.16 | Times, Frequency of kissing in the past 12 months |
| 17.98 | 2.62 | 27.03 | Times, Frequency of oral sex in the past 12 months |
| 35.17 | 8.86 | 69.19 | Times, Frequency of rimming in the past 12 months |
| 18.43 | 3.73 | 44.03 | Times, Frequency of anal sex in the past 12 months |
| 129.12 | 41.33 | 227.92 | weeks, Infection duration of chlamydia trachomatis at throat(asymptomatic infection) |
| 1.58 | 1.23 | 1.96 | weeks, Infection duration at urethral (symptomatic infection) |
| 83.82 | 81.21 | 89.72 | %, proportion of urethral infections that are asymptomatic |
| 77.92 | 61.57 | 96.91 | %, proportion of anal infections that are asymptomatic |
| 25.00 | 4.88 | 44.87 | weeks, Infection duration at urethral (asymptomatic infection) |
| 50.23 | 48.24 | 51.79 | weeks, Infection duration of chlamydia trachomatis at anus (asymptomatic infection) |
| 1.49 | 1.04 | 1.86 | weeks, Infection duration of chlamydia trachomatis at anus (symptomatic infection) |
| 81.60 | 66.36 | 91.07 | %, proportion of MSM received throat swab in the past 12 months |
| 77.31 | 66.34 | 93.03 | %, proportion of MSM received anal swab in the past 12 months |
| 79.00 | 65.17 | 90.04 | %, proportion of MSM received urine test in the past 12 months |
| 28.37 | 25.86 | 32.78 | %, proportion of 'oral sex and anal sex' in the same sex episode |
| 71.52 | 69.97 | 72.71 | %, proportion of 'oral sex and rimming' in the same sex episode |
| 68.49 | 66.86 | 70.42 | %, proportion of saliva use during anal sex |
| 80.00 | 80.00 | 80.00 | %, proportion of receptive oral sex followed by partner's insertive anal sex |
| 80.00 | 80.00 | 80.00 | %, proportion of insertive oral sex followed by partner's insertive rimming |
| 0.78 | 0.58 | 1.02 | %, Infection prevalence of chlamydia trachomatis only at Oropharynx |
| 1.81 | 1.56 | 2.05 | %, Infection prevalence of chlamydia trachomatis only at Urethral |
| 7.35 | 6.92 | 8.20 | %, Infection prevalence of chlamydia trachomatis only at Rectum |
| 0.00 | 0.00 | 0.00 | %, Infection prevalence of chlamydia trachomatis at Oropharynx & Urethra |
| 1.17 | 0.95 | 1.45 | %, Infection prevalence of chlamydia trachomatis at Oropharynx & Rectum |
| 1.21 | 1.02 | 1.43 | %, Infection prevalence of chlamydia trachomatis at Urethra & Rectum |
| 0.19 | 0.09 | 0.28 | %, Infection prevalence of chlamydia trachomatis at Oropharynx & Urethra & Rectum |

**Table S8. 95.** Model 17’ output parameters with 95% confidence intervals

| **Mean** | **95%CI: lower** | **95%CI: upper** | **Parameters** |
| --- | --- | --- | --- |
| 43.80 | 37.24 | 54.89 | %, Consistent condom usage in anal sex in past 12 months |
| 87.65 | 82.64 | 93.44 | %, Condom efficacy in preventing transmission |
| 8.01 | 2.64 | 12.08 | Times, Frequency of kissing in the past 12 months |
| 17.06 | 5.07 | 25.19 | Times, Frequency of oral sex in the past 12 months |
| 43.72 | 14.12 | 65.73 | Times, Frequency of rimming in the past 12 months |
| 20.34 | 8.38 | 32.97 | Times, Frequency of anal sex in the past 12 months |
| 171.32 | 61.70 | 228.35 | weeks, Infection duration of chlamydia trachomatis at throat(asymptomatic infection) |
| 1.58 | 1.26 | 1.83 | weeks, Infection duration at urethral (symptomatic infection) |
| 86.94 | 81.68 | 89.72 | %, proportion of urethral infections that are asymptomatic |
| 80.13 | 63.43 | 96.40 | %, proportion of anal infections that are asymptomatic |
| 19.11 | 6.09 | 36.85 | weeks, Infection duration at urethral (asymptomatic infection) |
| 50.25 | 48.53 | 51.59 | weeks, Infection duration of chlamydia trachomatis at anus (asymptomatic infection) |
| 1.35 | 1.05 | 1.82 | weeks, Infection duration of chlamydia trachomatis at anus (symptomatic infection) |
| 76.58 | 66.91 | 89.92 | %, proportion of MSM received throat swab in the past 12 months |
| 78.24 | 67.36 | 92.35 | %, proportion of MSM received anal swab in the past 12 months |
| 76.55 | 68.84 | 88.18 | %, proportion of MSM received urine test in the past 12 months |
| 29.42 | 26.42 | 32.69 | %, proportion of 'oral sex and anal sex' in the same sex episode |
| 71.55 | 70.29 | 72.72 | %, proportion of 'oral sex and rimming' in the same sex episode |
| 68.70 | 66.98 | 70.25 | %, proportion of saliva use during anal sex |
| 80.00 | 80.00 | 80.00 | %, proportion of receptive oral sex followed by partner's insertive anal sex |
| 80.00 | 80.00 | 80.00 | %, proportion of insertive oral sex followed by partner's insertive rimming |
| 0.79 | 0.58 | 1.01 | %, Infection prevalence of chlamydia trachomatis only at Oropharynx |
| 1.87 | 1.58 | 1.99 | %, Infection prevalence of chlamydia trachomatis only at Urethral |
| 7.58 | 7.00 | 8.11 | %, Infection prevalence of chlamydia trachomatis only at Rectum |
| 0.00 | 0.00 | 0.00 | %, Infection prevalence of chlamydia trachomatis at Oropharynx & Urethra |
| 1.22 | 1.06 | 1.45 | %, Infection prevalence of chlamydia trachomatis at Oropharynx & Rectum |
| 1.24 | 1.07 | 1.41 | %, Infection prevalence of chlamydia trachomatis at Urethra & Rectum |
| 0.20 | 0.10 | 0.27 | %, Infection prevalence of chlamydia trachomatis at Oropharynx & Urethra & Rectum |

**Table S8. 96.** Model 18’ output parameters with 95% confidence intervals

| **Mean** | **95%CI: lower** | **95%CI: upper** | **Parameters** |
| --- | --- | --- | --- |
| 44.66 | 39.14 | 54.18 | %, Consistent condom usage in anal sex in past 12 months |
| 87.92 | 83.24 | 92.75 | %, Condom efficacy in preventing transmission |
| 7.69 | 4.38 | 11.54 | Times, Frequency of kissing in the past 12 months |
| 11.72 | 5.51 | 19.72 | Times, Frequency of oral sex in the past 12 months |
| 47.91 | 19.38 | 60.20 | Times, Frequency of rimming in the past 12 months |
| 15.80 | 11.00 | 28.43 | Times, Frequency of anal sex in the past 12 months |
| 173.86 | 111.66 | 215.61 | weeks, Infection duration of chlamydia trachomatis at throat(asymptomatic infection) |
| 1.55 | 1.36 | 1.79 | weeks, Infection duration at urethral (symptomatic infection) |
| 85.90 | 82.88 | 89.28 | %, proportion of urethral infections that are asymptomatic |
| 81.41 | 67.28 | 94.76 | %, proportion of anal infections that are asymptomatic |
| 20.40 | 7.23 | 32.90 | weeks, Infection duration at urethral (asymptomatic infection) |
| 50.24 | 48.88 | 51.55 | weeks, Infection duration of chlamydia trachomatis at anus (asymptomatic infection) |
| 1.44 | 1.08 | 1.68 | weeks, Infection duration of chlamydia trachomatis at anus (symptomatic infection) |
| 79.72 | 69.13 | 89.78 | %, proportion of MSM received throat swab in the past 12 months |
| 77.87 | 67.96 | 90.66 | %, proportion of MSM received anal swab in the past 12 months |
| 81.00 | 71.62 | 87.48 | %, proportion of MSM received urine test in the past 12 months |
| 30.56 | 27.09 | 32.13 | %, proportion of 'oral sex and anal sex' in the same sex episode |
| 71.58 | 70.32 | 72.64 | %, proportion of 'oral sex and rimming' in the same sex episode |
| 68.62 | 67.36 | 70.02 | %, proportion of saliva use during anal sex |
| 80.00 | 80.00 | 80.00 | %, proportion of receptive oral sex followed by partner's insertive anal sex |
| 80.00 | 80.00 | 80.00 | %, proportion of insertive oral sex followed by partner's insertive rimming |
| 0.70 | 0.60 | 0.94 | %, Infection prevalence of chlamydia trachomatis only at Oropharynx |
| 1.76 | 1.60 | 1.98 | %, Infection prevalence of chlamydia trachomatis only at Urethral |
| 7.60 | 7.11 | 8.06 | %, Infection prevalence of chlamydia trachomatis only at Rectum |
| 0.00 | 0.00 | 0.00 | %, Infection prevalence of chlamydia trachomatis at Oropharynx & Urethra |
| 1.20 | 1.06 | 1.37 | %, Infection prevalence of chlamydia trachomatis at Oropharynx & Rectum |
| 1.20 | 1.12 | 1.38 | %, Infection prevalence of chlamydia trachomatis at Urethra & Rectum |
| 0.16 | 0.10 | 0.26 | %, Infection prevalence of chlamydia trachomatis at Oropharynx & Urethra & Rectum |

**Table S8. 97.** Model 19’ output parameters with 95% confidence intervals

| **Mean** | **95%CI: lower** | **95%CI: upper** | **Parameters** |
| --- | --- | --- | --- |
| 47.93 | 40.84 | 54.10 | %, Consistent condom usage in anal sex in past 12 months |
| 88.70 | 84.99 | 91.87 | %, Condom efficacy in preventing transmission |
| 8.94 | 5.43 | 11.25 | Times, Frequency of kissing in the past 12 months |
| 10.00 | 6.30 | 17.03 | Times, Frequency of oral sex in the past 12 months |
| 39.35 | 20.95 | 50.25 | Times, Frequency of rimming in the past 12 months |
| 21.12 | 12.21 | 26.46 | Times, Frequency of anal sex in the past 12 months |
| 153.64 | 113.94 | 201.12 | weeks, Infection duration of chlamydia trachomatis at throat(asymptomatic infection) |
| 1.60 | 1.39 | 1.77 | weeks, Infection duration at urethral (symptomatic infection) |
| 86.28 | 83.30 | 88.76 | %, proportion of urethral infections that are asymptomatic |
| 83.50 | 70.09 | 93.53 | %, proportion of anal infections that are asymptomatic |
| 13.10 | 7.34 | 18.71 | weeks, Infection duration at urethral (asymptomatic infection) |
| 50.20 | 49.21 | 51.14 | weeks, Infection duration of chlamydia trachomatis at anus (asymptomatic infection) |
| 1.37 | 1.10 | 1.63 | weeks, Infection duration of chlamydia trachomatis at anus (symptomatic infection) |
| 80.70 | 69.34 | 88.82 | %, proportion of MSM received throat swab in the past 12 months |
| 77.37 | 70.25 | 89.14 | %, proportion of MSM received anal swab in the past 12 months |
| 78.79 | 72.38 | 86.02 | %, proportion of MSM received urine test in the past 12 months |
| 30.51 | 27.59 | 31.74 | %, proportion of 'oral sex and anal sex' in the same sex episode |
| 71.64 | 70.67 | 72.48 | %, proportion of 'oral sex and rimming' in the same sex episode |
| 68.92 | 67.60 | 69.89 | %, proportion of saliva use during anal sex |
| 80.00 | 80.00 | 80.00 | %, proportion of receptive oral sex followed by partner's insertive anal sex |
| 80.00 | 80.00 | 80.00 | %, proportion of insertive oral sex followed by partner's insertive rimming |
| 0.80 | 0.63 | 0.91 | %, Infection prevalence of chlamydia trachomatis only at Oropharynx |
| 1.77 | 1.63 | 1.94 | %, Infection prevalence of chlamydia trachomatis only at Urethral |
| 7.50 | 7.16 | 7.99 | %, Infection prevalence of chlamydia trachomatis only at Rectum |
| 0.00 | 0.00 | 0.00 | %, Infection prevalence of chlamydia trachomatis at Oropharynx & Urethra |
| 1.20 | 1.09 | 1.32 | %, Infection prevalence of chlamydia trachomatis at Oropharynx & Rectum |
| 1.23 | 1.15 | 1.36 | %, Infection prevalence of chlamydia trachomatis at Urethra & Rectum |
| 0.17 | 0.10 | 0.24 | %, Infection prevalence of chlamydia trachomatis at Oropharynx & Urethra & Rectum |

**Table S8. 98**. Model 20’ output parameters with 95% confidence intervals

| **Mean** | **95%CI: lower** | **95%CI: upper** | **Parameters** |
| --- | --- | --- | --- |
| 48.83 | 41.35 | 53.60 | %, Consistent condom usage in anal sex in past 12 months |
| 88.59 | 85.15 | 91.74 | %, Condom efficacy in preventing transmission |
| 7.88 | 5.46 | 10.25 | Times, Frequency of kissing in the past 12 months |
| 9.32 | 6.67 | 14.76 | Times, Frequency of oral sex in the past 12 months |
| 35.60 | 22.72 | 47.82 | Times, Frequency of rimming in the past 12 months |
| 20.05 | 15.53 | 26.06 | Times, Frequency of anal sex in the past 12 months |
| 159.31 | 117.03 | 192.05 | weeks, Infection duration of chlamydia trachomatis at throat(asymptomatic infection) |
| 1.57 | 1.42 | 1.77 | weeks, Infection duration at urethral (symptomatic infection) |
| 85.58 | 83.36 | 88.71 | %, proportion of urethral infections that are asymptomatic |
| 83.06 | 72.26 | 93.01 | %, proportion of anal infections that are asymptomatic |
| 13.21 | 7.65 | 17.84 | weeks, Infection duration at urethral (asymptomatic infection) |
| 50.33 | 49.25 | 51.08 | weeks, Infection duration of chlamydia trachomatis at anus (asymptomatic infection) |
| 1.38 | 1.11 | 1.60 | weeks, Infection duration of chlamydia trachomatis at anus (symptomatic infection) |
| 77.92 | 70.64 | 85.89 | %, proportion of MSM received throat swab in the past 12 months |
| 80.06 | 70.73 | 88.27 | %, proportion of MSM received anal swab in the past 12 months |
| 79.24 | 72.99 | 85.73 | %, proportion of MSM received urine test in the past 12 months |
| 30.08 | 27.93 | 31.62 | %, proportion of 'oral sex and anal sex' in the same sex episode |
| 71.71 | 70.68 | 72.47 | %, proportion of 'oral sex and rimming' in the same sex episode |
| 68.63 | 67.62 | 69.71 | %, proportion of saliva use during anal sex |
| 80.00 | 80.00 | 80.00 | %, proportion of receptive oral sex followed by partner's insertive anal sex |
| 80.00 | 80.00 | 80.00 | %, proportion of insertive oral sex followed by partner's insertive rimming |
| 0.79 | 0.64 | 0.90 | %, Infection prevalence of chlamydia trachomatis only at Oropharynx |
| 1.78 | 1.65 | 1.91 | %, Infection prevalence of chlamydia trachomatis only at Urethral |
| 7.60 | 7.18 | 7.98 | %, Infection prevalence of chlamydia trachomatis only at Rectum |
| 0.00 | 0.00 | 0.00 | %, Infection prevalence of chlamydia trachomatis at Oropharynx & Urethra |
| 1.22 | 1.10 | 1.33 | %, Infection prevalence of chlamydia trachomatis at Oropharynx & Rectum |
| 1.27 | 1.18 | 1.35 | %, Infection prevalence of chlamydia trachomatis at Urethra & Rectum |
| 0.17 | 0.11 | 0.23 | %, Infection prevalence of chlamydia trachomatis at Oropharynx & Urethra & Rectum |

Model output parameters (Published validation dataset 1): Data from 1,011 asymptomatic MSM attending Melbourne Sexual Health Centre

**Table S8. 99.** Model 3’ output parameters with 95% confidence intervals

| **Mean** | **95%CI: lower** | **95%CI: upper** | **Parameters** |
| --- | --- | --- | --- |
| 43.82 | 36.01 | 58.44 | %, Consistent condom usage in anal sex in past 12 months |
| 89.33 | 80.48 | 94.68 | %, Condom efficacy in preventing transmission |
| 7.04 | 1.54 | 11.63 | Times, Frequency of kissing in the past 12 months |
| 16.66 | 1.77 | 27.78 | Times, Frequency of oral sex in the past 12 months |
| 40.35 | 1.86 | 77.20 | Times, Frequency of rimming in the past 12 months |
| 30.07 | 2.70 | 51.56 | Times, Frequency of anal sex in the past 12 months |
| 130.69 | 46.76 | 238.73 | weeks, Infection duration of chlamydia trachomatis at throat(asymptomatic infection) |
| 1.45 | 1.09 | 1.95 | weeks, Infection duration at urethral (symptomatic infection) |
| 100.00 | 100.00 | 100.00 | %, proportion of urethral infections that are asymptomatic |
| 100.00 | 100.00 | 100.00 | %, proportion of anal infections that are asymptomatic |
| 25.16 | 4.76 | 46.03 | weeks, Infection duration at urethral (asymptomatic infection) |
| 49.94 | 48.06 | 51.70 | weeks, Infection duration of chlamydia trachomatis at anus (asymptomatic infection) |
| 1.44 | 1.12 | 1.97 | weeks, Infection duration of chlamydia trachomatis at anus (symptomatic infection) |
| 80.84 | 67.45 | 94.21 | %, proportion of MSM received throat swab in the past 12 months |
| 82.23 | 63.90 | 94.90 | %, proportion of MSM received anal swab in the past 12 months |
| 76.88 | 66.13 | 92.76 | %, proportion of MSM received urine test in the past 12 months |
| 28.14 | 24.99 | 32.84 | %, proportion of 'oral sex and anal sex' in the same sex episode |
| 71.28 | 69.79 | 72.57 | %, proportion of 'oral sex and rimming' in the same sex episode |
| 69.75 | 66.55 | 70.80 | %, proportion of saliva use during anal sex |
| 80.00 | 80.00 | 80.00 | %, proportion of receptive oral sex followed by partner's insertive anal sex |
| 80.00 | 80.00 | 80.00 | %, proportion of insertive oral sex followed by partner's insertive rimming |
| 1.39 | 0.43 | 2.45 | %, Infection prevalence of chlamydia trachomatis only at Oropharynx |
| 1.59 | 0.90 | 2.29 | %, Infection prevalence of chlamydia trachomatis only at Urethral |
| 6.98 | 5.78 | 8.61 | %, Infection prevalence of chlamydia trachomatis only at Rectum |
| 0.00 | 0.00 | 0.00 | %, Infection prevalence of chlamydia trachomatis at Oropharynx & Urethra |
| 2.09 | 1.18 | 3.90 | %, Infection prevalence of chlamydia trachomatis at Oropharynx & Rectum |
| 0.52 | 0.20 | 1.09 | %, Infection prevalence of chlamydia trachomatis at Urethra & Rectum |
| 0.00 | 0.00 | 0.00 | %, Infection prevalence of chlamydia trachomatis at Oropharynx & Urethra & Rectum |

**Table S8. 100.** Model 14’ output parameters with 95% confidence intervals

| **Mean** | **95%CI: lower** | **95%CI: upper** | **Parameters** |
| --- | --- | --- | --- |
| 47.82 | 36.72 | 57.19 | %, Consistent condom usage in anal sex in past 12 months |
| 88.29 | 81.45 | 94.19 | %, Condom efficacy in preventing transmission |
| 6.48 | 1.71 | 11.48 | Times, Frequency of kissing in the past 12 months |
| 13.56 | 4.12 | 27.42 | Times, Frequency of oral sex in the past 12 months |
| 34.36 | 6.70 | 73.19 | Times, Frequency of rimming in the past 12 months |
| 15.65 | 4.05 | 40.58 | Times, Frequency of anal sex in the past 12 months |
| 139.55 | 75.04 | 226.45 | weeks, Infection duration of chlamydia trachomatis at throat(asymptomatic infection) |
| 1.52 | 1.13 | 1.89 | weeks, Infection duration at urethral (symptomatic infection) |
| 100.00 | 100.00 | 100.00 | %, proportion of urethral infections that are asymptomatic |
| 100.00 | 100.00 | 100.00 | %, proportion of anal infections that are asymptomatic |
| 29.47 | 6.60 | 45.87 | weeks, Infection duration at urethral (asymptomatic infection) |
| 49.83 | 48.27 | 51.50 | weeks, Infection duration of chlamydia trachomatis at anus (asymptomatic infection) |
| 1.56 | 1.13 | 1.91 | weeks, Infection duration of chlamydia trachomatis at anus (symptomatic infection) |
| 80.80 | 68.91 | 91.21 | %, proportion of MSM received throat swab in the past 12 months |
| 77.96 | 65.46 | 91.32 | %, proportion of MSM received anal swab in the past 12 months |
| 81.58 | 67.48 | 91.40 | %, proportion of MSM received urine test in the past 12 months |
| 30.02 | 25.40 | 32.35 | %, proportion of 'oral sex and anal sex' in the same sex episode |
| 71.42 | 69.92 | 72.44 | %, proportion of 'oral sex and rimming' in the same sex episode |
| 68.69 | 66.99 | 70.69 | %, proportion of saliva use during anal sex |
| 80.00 | 80.00 | 80.00 | %, proportion of receptive oral sex followed by partner's insertive anal sex |
| 80.00 | 80.00 | 80.00 | %, proportion of insertive oral sex followed by partner's insertive rimming |
| 1.49 | 0.67 | 2.33 | %, Infection prevalence of chlamydia trachomatis only at Oropharynx |
| 1.74 | 1.13 | 2.19 | %, Infection prevalence of chlamydia trachomatis only at Urethral |
| 7.11 | 5.85 | 8.43 | %, Infection prevalence of chlamydia trachomatis only at Rectum |
| 0.00 | 0.00 | 0.00 | %, Infection prevalence of chlamydia trachomatis at Oropharynx & Urethra |
| 2.42 | 1.34 | 3.63 | %, Infection prevalence of chlamydia trachomatis at Oropharynx & Rectum |
| 0.63 | 0.31 | 1.04 | %, Infection prevalence of chlamydia trachomatis at Urethra & Rectum |
| 0.00 | 0.00 | 0.00 | %, Infection prevalence of chlamydia trachomatis at Oropharynx & Urethra & Rectum |

**Table S8. 101.** Model 15’ output parameters with 95% confidence intervals

| **Mean** | **95%CI: lower** | **95%CI: upper** | **Parameters** |
| --- | --- | --- | --- |
| 50.14 | 41.24 | 55.02 | %, Consistent condom usage in anal sex in past 12 months |
| 88.31 | 83.20 | 91.95 | %, Condom efficacy in preventing transmission |
| 7.31 | 2.92 | 10.30 | Times, Frequency of kissing in the past 12 months |
| 21.40 | 16.18 | 25.11 | Times, Frequency of oral sex in the past 12 months |
| 28.42 | 17.46 | 57.50 | Times, Frequency of rimming in the past 12 months |
| 26.50 | 12.02 | 39.25 | Times, Frequency of anal sex in the past 12 months |
| 148.45 | 105.84 | 201.32 | weeks, Infection duration of chlamydia trachomatis at throat(asymptomatic infection) |
| 1.50 | 1.17 | 1.69 | weeks, Infection duration at urethral (symptomatic infection) |
| 100.00 | 100.00 | 100.00 | %, proportion of urethral infections that are asymptomatic |
| 100.00 | 100.00 | 100.00 | %, proportion of anal infections that are asymptomatic |
| 13.22 | 7.69 | 25.34 | weeks, Infection duration at urethral (asymptomatic infection) |
| 49.67 | 48.56 | 50.80 | weeks, Infection duration of chlamydia trachomatis at anus (asymptomatic infection) |
| 1.63 | 1.35 | 1.83 | weeks, Infection duration of chlamydia trachomatis at anus (symptomatic infection) |
| 81.68 | 73.71 | 86.87 | %, proportion of MSM received throat swab in the past 12 months |
| 77.11 | 68.94 | 87.46 | %, proportion of MSM received anal swab in the past 12 months |
| 78.09 | 71.68 | 87.68 | %, proportion of MSM received urine test in the past 12 months |
| 28.59 | 26.64 | 31.30 | %, proportion of 'oral sex and anal sex' in the same sex episode |
| 71.43 | 70.28 | 72.17 | %, proportion of 'oral sex and rimming' in the same sex episode |
| 69.07 | 67.61 | 70.32 | %, proportion of saliva use during anal sex |
| 80.00 | 80.00 | 80.00 | %, proportion of receptive oral sex followed by partner's insertive anal sex |
| 80.00 | 80.00 | 80.00 | %, proportion of insertive oral sex followed by partner's insertive rimming |
| 1.56 | 0.83 | 2.16 | %, Infection prevalence of chlamydia trachomatis only at Oropharynx |
| 1.70 | 1.28 | 2.01 | %, Infection prevalence of chlamydia trachomatis only at Urethral |
| 7.52 | 6.61 | 8.25 | %, Infection prevalence of chlamydia trachomatis only at Rectum |
| 0.00 | 0.00 | 0.00 | %, Infection prevalence of chlamydia trachomatis at Oropharynx & Urethra |
| 2.52 | 2.00 | 3.11 | %, Infection prevalence of chlamydia trachomatis at Oropharynx & Rectum |
| 0.70 | 0.42 | 0.90 | %, Infection prevalence of chlamydia trachomatis at Urethra & Rectum |
| 0.00 | 0.00 | 0.00 | %, Infection prevalence of chlamydia trachomatis at Oropharynx & Urethra & Rectum |

**Table S8. 102.** Model 16’ output parameters with 95% confidence intervals

| **Mean** | **95%CI: lower** | **95%CI: upper** | **Parameters** |
| --- | --- | --- | --- |
| 46.89 | 36.75 | 58.26 | %, Consistent condom usage in anal sex in past 12 months |
| 85.37 | 80.70 | 93.04 | %, Condom efficacy in preventing transmission |
| 7.33 | 1.05 | 11.92 | Times, Frequency of kissing in the past 12 months |
| 13.72 | 3.87 | 27.15 | Times, Frequency of oral sex in the past 12 months |
| 27.51 | 7.80 | 73.33 | Times, Frequency of rimming in the past 12 months |
| 25.00 | 3.27 | 52.19 | Times, Frequency of anal sex in the past 12 months |
| 149.86 | 52.56 | 225.00 | weeks, Infection duration of chlamydia trachomatis at throat(asymptomatic infection) |
| 1.48 | 1.07 | 1.95 | weeks, Infection duration at urethral (symptomatic infection) |
| 100.00 | 100.00 | 100.00 | %, proportion of urethral infections that are asymptomatic |
| 100.00 | 100.00 | 100.00 | %, proportion of anal infections that are asymptomatic |
| 21.16 | 4.92 | 37.55 | weeks, Infection duration at urethral (asymptomatic infection) |
| 49.63 | 48.32 | 51.30 | weeks, Infection duration of chlamydia trachomatis at anus (asymptomatic infection) |
| 1.42 | 1.06 | 1.96 | weeks, Infection duration of chlamydia trachomatis at anus (symptomatic infection) |
| 77.44 | 66.66 | 94.25 | %, proportion of MSM received throat swab in the past 12 months |
| 81.88 | 67.54 | 94.35 | %, proportion of MSM received anal swab in the past 12 months |
| 79.78 | 65.12 | 90.70 | %, proportion of MSM received urine test in the past 12 months |
| 30.40 | 25.79 | 33.32 | %, proportion of 'oral sex and anal sex' in the same sex episode |
| 71.72 | 70.18 | 72.84 | %, proportion of 'oral sex and rimming' in the same sex episode |
| 68.76 | 66.61 | 70.46 | %, proportion of saliva use during anal sex |
| 80.00 | 80.00 | 80.00 | %, proportion of receptive oral sex followed by partner's insertive anal sex |
| 80.00 | 80.00 | 80.00 | %, proportion of insertive oral sex followed by partner's insertive rimming |
| 1.08 | 0.35 | 2.04 | %, Infection prevalence of chlamydia trachomatis only at Oropharynx |
| 1.52 | 0.84 | 2.26 | %, Infection prevalence of chlamydia trachomatis only at Urethral |
| 7.68 | 5.85 | 8.81 | %, Infection prevalence of chlamydia trachomatis only at Rectum |
| 0.00 | 0.00 | 0.00 | %, Infection prevalence of chlamydia trachomatis at Oropharynx & Urethra |
| 2.71 | 1.19 | 3.81 | %, Infection prevalence of chlamydia trachomatis at Oropharynx & Rectum |
| 0.76 | 0.34 | 1.07 | %, Infection prevalence of chlamydia trachomatis at Urethra & Rectum |
| 0.00 | 0.00 | 0.00 | %, Infection prevalence of chlamydia trachomatis at Oropharynx & Urethra & Rectum |

**Table S8. 103.** Model 17’ output parameters with 95% confidence intervals

| **Mean** | **95%CI: lower** | **95%CI: upper** | **Parameters** |
| --- | --- | --- | --- |
| 45.43 | 36.64 | 55.06 | %, Consistent condom usage in anal sex in past 12 months |
| 87.87 | 80.92 | 92.70 | %, Condom efficacy in preventing transmission |
| 8.65 | 2.84 | 11.38 | Times, Frequency of kissing in the past 12 months |
| 17.81 | 4.94 | 24.23 | Times, Frequency of oral sex in the past 12 months |
| 30.36 | 9.20 | 65.93 | Times, Frequency of rimming in the past 12 months |
| 26.94 | 6.69 | 48.84 | Times, Frequency of anal sex in the past 12 months |
| 136.68 | 56.90 | 217.62 | weeks, Infection duration of chlamydia trachomatis at throat(asymptomatic infection) |
| 1.58 | 1.13 | 1.85 | weeks, Infection duration at urethral (symptomatic infection) |
| 100.00 | 100.00 | 100.00 | %, proportion of urethral infections that are asymptomatic |
| 100.00 | 100.00 | 100.00 | %, proportion of anal infections that are asymptomatic |
| 15.29 | 6.01 | 36.90 | weeks, Infection duration at urethral (asymptomatic infection) |
| 49.77 | 48.37 | 51.14 | weeks, Infection duration of chlamydia trachomatis at anus (asymptomatic infection) |
| 1.45 | 1.14 | 1.88 | weeks, Infection duration of chlamydia trachomatis at anus (symptomatic infection) |
| 82.49 | 66.95 | 93.32 | %, proportion of MSM received throat swab in the past 12 months |
| 82.98 | 70.08 | 93.50 | %, proportion of MSM received anal swab in the past 12 months |
| 78.14 | 66.46 | 89.51 | %, proportion of MSM received urine test in the past 12 months |
| 29.56 | 26.13 | 32.75 | %, proportion of 'oral sex and anal sex' in the same sex episode |
| 71.47 | 70.28 | 72.58 | %, proportion of 'oral sex and rimming' in the same sex episode |
| 68.35 | 66.84 | 69.51 | %, proportion of saliva use during anal sex |
| 80.00 | 80.00 | 80.00 | %, proportion of receptive oral sex followed by partner's insertive anal sex |
| 80.00 | 80.00 | 80.00 | %, proportion of insertive oral sex followed by partner's insertive rimming |
| 1.38 | 0.39 | 2.00 | %, Infection prevalence of chlamydia trachomatis only at Oropharynx |
| 1.64 | 0.85 | 2.23 | %, Infection prevalence of chlamydia trachomatis only at Urethral |
| 7.14 | 5.88 | 8.46 | %, Infection prevalence of chlamydia trachomatis only at Rectum |
| 0.00 | 0.00 | 0.00 | %, Infection prevalence of chlamydia trachomatis at Oropharynx & Urethra |
| 2.65 | 1.24 | 3.74 | %, Infection prevalence of chlamydia trachomatis at Oropharynx & Rectum |
| 0.76 | 0.43 | 1.05 | %, Infection prevalence of chlamydia trachomatis at Urethra & Rectum |
| 0.00 | 0.00 | 0.00 | %, Infection prevalence of chlamydia trachomatis at Oropharynx & Urethra & Rectum |

**Table S8. 104.** Model 18’ output parameters with 95% confidence intervals

| **Mean** | **95%CI: lower** | **95%CI: upper** | **Parameters** |
| --- | --- | --- | --- |
| 44.25 | 36.98 | 53.97 | %, Consistent condom usage in anal sex in past 12 months |
| 85.58 | 81.53 | 91.98 | %, Condom efficacy in preventing transmission |
| 7.74 | 3.34 | 10.71 | Times, Frequency of kissing in the past 12 months |
| 12.54 | 6.39 | 23.35 | Times, Frequency of oral sex in the past 12 months |
| 40.49 | 14.57 | 57.99 | Times, Frequency of rimming in the past 12 months |
| 16.98 | 8.33 | 38.55 | Times, Frequency of anal sex in the past 12 months |
| 127.49 | 71.02 | 200.96 | weeks, Infection duration of chlamydia trachomatis at throat(asymptomatic infection) |
| 1.47 | 1.23 | 1.81 | weeks, Infection duration at urethral (symptomatic infection) |
| 100.00 | 100.00 | 100.00 | %, proportion of urethral infections that are asymptomatic |
| 100.00 | 100.00 | 100.00 | %, proportion of anal infections that are asymptomatic |
| 19.04 | 6.82 | 33.88 | weeks, Infection duration at urethral (asymptomatic infection) |
| 49.76 | 48.57 | 50.87 | weeks, Infection duration of chlamydia trachomatis at anus (asymptomatic infection) |
| 1.49 | 1.14 | 1.88 | weeks, Infection duration of chlamydia trachomatis at anus (symptomatic infection) |
| 75.27 | 68.58 | 92.22 | %, proportion of MSM received throat swab in the past 12 months |
| 83.29 | 71.00 | 91.93 | %, proportion of MSM received anal swab in the past 12 months |
| 78.73 | 67.57 | 88.81 | %, proportion of MSM received urine test in the past 12 months |
| 28.95 | 26.75 | 32.51 | %, proportion of 'oral sex and anal sex' in the same sex episode |
| 71.40 | 70.37 | 72.54 | %, proportion of 'oral sex and rimming' in the same sex episode |
| 68.20 | 66.92 | 69.30 | %, proportion of saliva use during anal sex |
| 80.00 | 80.00 | 80.00 | %, proportion of receptive oral sex followed by partner's insertive anal sex |
| 80.00 | 80.00 | 80.00 | %, proportion of insertive oral sex followed by partner's insertive rimming |
| 1.49 | 0.68 | 1.98 | %, Infection prevalence of chlamydia trachomatis only at Oropharynx |
| 1.29 | 0.89 | 2.18 | %, Infection prevalence of chlamydia trachomatis only at Urethral |
| 7.11 | 5.98 | 8.26 | %, Infection prevalence of chlamydia trachomatis only at Rectum |
| 0.00 | 0.00 | 0.00 | %, Infection prevalence of chlamydia trachomatis at Oropharynx & Urethra |
| 2.60 | 1.45 | 3.58 | %, Infection prevalence of chlamydia trachomatis at Oropharynx & Rectum |
| 0.72 | 0.45 | 1.02 | %, Infection prevalence of chlamydia trachomatis at Urethra & Rectum |
| 0.00 | 0.00 | 0.00 | %, Infection prevalence of chlamydia trachomatis at Oropharynx & Urethra & Rectum |

**Table S8. 105**. Model 19’ output parameters with 95% confidence intervals

| **Mean** | **95%CI: lower** | **95%CI: upper** | **Parameters** |
| --- | --- | --- | --- |
| 47.67 | 38.04 | 54.04 | %, Consistent condom usage in anal sex in past 12 months |
| 87.61 | 82.68 | 91.65 | %, Condom efficacy in preventing transmission |
| 6.88 | 4.36 | 10.44 | Times, Frequency of kissing in the past 12 months |
| 14.08 | 7.52 | 22.60 | Times, Frequency of oral sex in the past 12 months |
| 36.64 | 16.31 | 56.09 | Times, Frequency of rimming in the past 12 months |
| 28.51 | 10.32 | 37.74 | Times, Frequency of anal sex in the past 12 months |
| 145.02 | 72.78 | 192.66 | weeks, Infection duration of chlamydia trachomatis at throat(asymptomatic infection) |
| 1.57 | 1.26 | 1.79 | weeks, Infection duration at urethral (symptomatic infection) |
| 100.00 | 100.00 | 100.00 | %, proportion of urethral infections that are asymptomatic |
| 100.00 | 100.00 | 100.00 | %, proportion of anal infections that are asymptomatic |
| 11.77 | 7.65 | 22.48 | weeks, Infection duration at urethral (asymptomatic infection) |
| 49.58 | 48.75 | 50.47 | weeks, Infection duration of chlamydia trachomatis at anus (asymptomatic infection) |
| 1.48 | 1.21 | 1.83 | weeks, Infection duration of chlamydia trachomatis at anus (symptomatic infection) |
| 82.14 | 69.59 | 91.88 | %, proportion of MSM received throat swab in the past 12 months |
| 77.89 | 71.73 | 89.80 | %, proportion of MSM received anal swab in the past 12 months |
| 77.46 | 68.51 | 87.22 | %, proportion of MSM received urine test in the past 12 months |
| 30.19 | 26.89 | 32.31 | %, proportion of 'oral sex and anal sex' in the same sex episode |
| 71.49 | 70.57 | 72.41 | %, proportion of 'oral sex and rimming' in the same sex episode |
| 67.92 | 67.17 | 69.27 | %, proportion of saliva use during anal sex |
| 80.00 | 80.00 | 80.00 | %, proportion of receptive oral sex followed by partner's insertive anal sex |
| 80.00 | 80.00 | 80.00 | %, proportion of insertive oral sex followed by partner's insertive rimming |
| 1.12 | 0.69 | 1.91 | %, Infection prevalence of chlamydia trachomatis only at Oropharynx |
| 1.62 | 0.92 | 2.13 | %, Infection prevalence of chlamydia trachomatis only at Urethral |
| 7.29 | 6.08 | 8.04 | %, Infection prevalence of chlamydia trachomatis only at Rectum |
| 0.00 | 0.00 | 0.00 | %, Infection prevalence of chlamydia trachomatis at Oropharynx & Urethra |
| 2.79 | 1.60 | 3.55 | %, Infection prevalence of chlamydia trachomatis at Oropharynx & Rectum |
| 0.79 | 0.52 | 1.01 | %, Infection prevalence of chlamydia trachomatis at Urethra & Rectum |
| 0.00 | 0.00 | 0.00 | %, Infection prevalence of chlamydia trachomatis at Oropharynx & Urethra & Rectum |

**Table S8. 106.** Model 20’ output parameters with 95% confidence intervals

| **Mean** | **95%CI: lower** | **95%CI: upper** | **Parameters** |
| --- | --- | --- | --- |
| 43.61 | 38.53 | 52.93 | %, Consistent condom usage in anal sex in past 12 months |
| 86.87 | 83.64 | 90.30 | %, Condom efficacy in preventing transmission |
| 7.33 | 4.58 | 9.64 | Times, Frequency of kissing in the past 12 months |
| 16.59 | 8.55 | 20.42 | Times, Frequency of oral sex in the past 12 months |
| 36.37 | 20.08 | 50.24 | Times, Frequency of rimming in the past 12 months |
| 25.39 | 14.21 | 37.17 | Times, Frequency of anal sex in the past 12 months |
| 149.31 | 76.04 | 192.53 | weeks, Infection duration of chlamydia trachomatis at throat(asymptomatic infection) |
| 1.50 | 1.29 | 1.74 | weeks, Infection duration at urethral (symptomatic infection) |
| 100.00 | 100.00 | 100.00 | %, proportion of urethral infections that are asymptomatic |
| 100.00 | 100.00 | 100.00 | %, proportion of anal infections that are asymptomatic |
| 12.93 | 8.25 | 19.55 | weeks, Infection duration at urethral (asymptomatic infection) |
| 49.49 | 48.96 | 50.28 | weeks, Infection duration of chlamydia trachomatis at anus (asymptomatic infection) |
| 1.49 | 1.24 | 1.75 | weeks, Infection duration of chlamydia trachomatis at anus (symptomatic infection) |
| 76.80 | 71.87 | 89.93 | %, proportion of MSM received throat swab in the past 12 months |
| 78.06 | 72.22 | 87.13 | %, proportion of MSM received anal swab in the past 12 months |
| 79.39 | 71.77 | 85.25 | %, proportion of MSM received urine test in the past 12 months |
| 29.42 | 27.31 | 32.19 | %, proportion of 'oral sex and anal sex' in the same sex episode |
| 71.35 | 70.64 | 72.22 | %, proportion of 'oral sex and rimming' in the same sex episode |
| 68.40 | 67.33 | 69.06 | %, proportion of saliva use during anal sex |
| 80.00 | 80.00 | 80.00 | %, proportion of receptive oral sex followed by partner's insertive anal sex |
| 80.00 | 80.00 | 80.00 | %, proportion of insertive oral sex followed by partner's insertive rimming |
| 1.24 | 0.74 | 1.83 | %, Infection prevalence of chlamydia trachomatis only at Oropharynx |
| 1.61 | 1.01 | 2.09 | %, Infection prevalence of chlamydia trachomatis only at Urethral |
| 7.09 | 6.23 | 7.95 | %, Infection prevalence of chlamydia trachomatis only at Rectum |
| 0.00 | 0.00 | 0.00 | %, Infection prevalence of chlamydia trachomatis at Oropharynx & Urethra |
| 2.37 | 1.77 | 3.34 | %, Infection prevalence of chlamydia trachomatis at Oropharynx & Rectum |
| 0.76 | 0.55 | 0.98 | %, Infection prevalence of chlamydia trachomatis at Urethra & Rectum |
| 0.00 | 0.00 | 0.00 | %, Infection prevalence of chlamydia trachomatis at Oropharynx & Urethra & Rectum |

Model output parameters (Published validation dataset2 ): Data from 393 MSM attending STD & HIV care clinics in the USA

**Table S8. 107.** Model 3’ output parameters with 95% confidence intervals

| **Mean** | **95%CI: lower** | **95%CI: upper** | **Parameters** |
| --- | --- | --- | --- |
| 48.15 | 37.95 | 58.12 | %, Consistent condom usage in anal sex in past 12 months |
| 87.99 | 81.65 | 92.86 | %, Condom efficacy in preventing transmission |
| 5.82 | 1.10 | 12.47 | Times, Frequency of kissing in the past 12 months |
| 10.29 | 0.73 | 25.63 | Times, Frequency of oral sex in the past 12 months |
| 44.07 | 10.17 | 76.62 | Times, Frequency of rimming in the past 12 months |
| 16.73 | 1.84 | 42.71 | Times, Frequency of anal sex in the past 12 months |
| 166.22 | 31.94 | 237.92 | weeks, Infection duration of chlamydia trachomatis at throat(asymptomatic infection) |
| 1.46 | 1.04 | 1.95 | weeks, Infection duration at urethral (symptomatic infection) |
| 84.97 | 81.29 | 89.64 | %, proportion of urethral infections that are asymptomatic |
| 77.42 | 64.50 | 96.74 | %, proportion of anal infections that are asymptomatic |
| 32.67 | 5.66 | 51.79 | weeks, Infection duration at urethral (asymptomatic infection) |
| 49.55 | 48.22 | 51.86 | weeks, Infection duration of chlamydia trachomatis at anus (asymptomatic infection) |
| 1.53 | 1.16 | 1.94 | weeks, Infection duration of chlamydia trachomatis at anus (symptomatic infection) |
| 80.40 | 64.69 | 95.06 | %, proportion of MSM received throat swab in the past 12 months |
| 78.69 | 65.21 | 92.03 | %, proportion of MSM received anal swab in the past 12 months |
| 78.62 | 64.94 | 94.90 | %, proportion of MSM received urine test in the past 12 months |
| 30.44 | 25.27 | 33.44 | %, proportion of 'oral sex and anal sex' in the same sex episode |
| 70.95 | 69.87 | 72.54 | %, proportion of 'oral sex and rimming' in the same sex episode |
| 68.11 | 66.02 | 70.86 | %, proportion of saliva use during anal sex |
| 80.00 | 80.00 | 80.00 | %, proportion of receptive oral sex followed by partner's insertive anal sex |
| 80.00 | 80.00 | 80.00 | %, proportion of insertive oral sex followed by partner's insertive rimming |
| 0.76 | 0.14 | 1.96 | %, Infection prevalence of chlamydia trachomatis only at Oropharynx |
| 2.25 | 1.20 | 4.25 | %, Infection prevalence of chlamydia trachomatis only at Urethral |
| 6.91 | 4.44 | 9.11 | %, Infection prevalence of chlamydia trachomatis only at Rectum |
| 0.00 | 0.00 | 0.00 | %, Infection prevalence of chlamydia trachomatis at Oropharynx & Urethra |
| 2.03 | 0.76 | 3.31 | %, Infection prevalence of chlamydia trachomatis at Oropharynx & Rectum |
| 1.33 | 0.36 | 2.27 | %, Infection prevalence of chlamydia trachomatis at Urethra & Rectum |
| 0.00 | 0.00 | 0.00 | %, Infection prevalence of chlamydia trachomatis at Oropharynx & Urethra & Rectum |

**Table S8. 108.** Model 14’ output parameters with 95% confidence intervals

| **Mean** | **95%CI: lower** | **95%CI: upper** | **Parameters** |
| --- | --- | --- | --- |
| 48.28 | 41.43 | 52.98 | %, Consistent condom usage in anal sex in past 12 months |
| 86.52 | 83.27 | 92.11 | %, Condom efficacy in preventing transmission |
| 6.82 | 1.81 | 11.58 | Times, Frequency of kissing in the past 12 months |
| 11.62 | 1.06 | 22.98 | Times, Frequency of oral sex in the past 12 months |
| 45.67 | 24.25 | 67.44 | Times, Frequency of rimming in the past 12 months |
| 19.51 | 7.46 | 34.65 | Times, Frequency of anal sex in the past 12 months |
| 114.60 | 47.47 | 203.46 | weeks, Infection duration of chlamydia trachomatis at throat(asymptomatic infection) |
| 1.42 | 1.12 | 1.80 | weeks, Infection duration at urethral (symptomatic infection) |
| 86.26 | 82.16 | 88.96 | %, proportion of urethral infections that are asymptomatic |
| 76.78 | 66.59 | 92.76 | %, proportion of anal infections that are asymptomatic |
| 29.02 | 13.16 | 44.47 | weeks, Infection duration at urethral (asymptomatic infection) |
| 49.67 | 48.43 | 51.17 | weeks, Infection duration of chlamydia trachomatis at anus (asymptomatic infection) |
| 1.56 | 1.19 | 1.86 | weeks, Infection duration of chlamydia trachomatis at anus (symptomatic infection) |
| 83.68 | 70.59 | 93.37 | %, proportion of MSM received throat swab in the past 12 months |
| 78.86 | 69.70 | 88.06 | %, proportion of MSM received anal swab in the past 12 months |
| 78.87 | 68.22 | 88.91 | %, proportion of MSM received urine test in the past 12 months |
| 30.61 | 26.38 | 32.77 | %, proportion of 'oral sex and anal sex' in the same sex episode |
| 71.23 | 70.31 | 72.29 | %, proportion of 'oral sex and rimming' in the same sex episode |
| 68.67 | 66.97 | 70.27 | %, proportion of saliva use during anal sex |
| 80.00 | 80.00 | 80.00 | %, proportion of receptive oral sex followed by partner's insertive anal sex |
| 80.00 | 80.00 | 80.00 | %, proportion of insertive oral sex followed by partner's insertive rimming |
| 0.87 | 0.41 | 1.45 | %, Infection prevalence of chlamydia trachomatis only at Oropharynx |
| 2.86 | 1.55 | 3.76 | %, Infection prevalence of chlamydia trachomatis only at Urethral |
| 6.80 | 4.86 | 8.70 | %, Infection prevalence of chlamydia trachomatis only at Rectum |
| 0.00 | 0.00 | 0.00 | %, Infection prevalence of chlamydia trachomatis at Oropharynx & Urethra |
| 1.77 | 1.03 | 2.76 | %, Infection prevalence of chlamydia trachomatis at Oropharynx & Rectum |
| 1.30 | 0.58 | 2.03 | %, Infection prevalence of chlamydia trachomatis at Urethra & Rectum |
| 0.00 | 0.00 | 0.00 | %, Infection prevalence of chlamydia trachomatis at Oropharynx & Urethra & Rectum |

**Table S8. 109.** Model 15’ output parameters with 95% confidence intervals

| **Mean** | **95%CI: lower** | **95%CI: upper** | **Parameters** |
| --- | --- | --- | --- |
| 47.02 | 41.37 | 52.24 | %, Consistent condom usage in anal sex in past 12 months |
| 86.46 | 84.31 | 91.89 | %, Condom efficacy in preventing transmission |
| 5.17 | 2.17 | 10.75 | Times, Frequency of kissing in the past 12 months |
| 13.54 | 3.48 | 22.23 | Times, Frequency of oral sex in the past 12 months |
| 46.89 | 26.65 | 66.33 | Times, Frequency of rimming in the past 12 months |
| 20.31 | 10.55 | 34.08 | Times, Frequency of anal sex in the past 12 months |
| 146.63 | 55.05 | 197.78 | weeks, Infection duration of chlamydia trachomatis at throat(asymptomatic infection) |
| 1.43 | 1.14 | 1.77 | weeks, Infection duration at urethral (symptomatic infection) |
| 85.26 | 82.60 | 88.50 | %, proportion of urethral infections that are asymptomatic |
| 81.06 | 68.14 | 91.85 | %, proportion of anal infections that are asymptomatic |
| 22.19 | 13.49 | 36.77 | weeks, Infection duration at urethral (asymptomatic infection) |
| 49.93 | 48.49 | 51.10 | weeks, Infection duration of chlamydia trachomatis at anus (asymptomatic infection) |
| 1.56 | 1.20 | 1.80 | weeks, Infection duration of chlamydia trachomatis at anus (symptomatic infection) |
| 80.96 | 72.03 | 90.49 | %, proportion of MSM received throat swab in the past 12 months |
| 77.75 | 70.07 | 87.34 | %, proportion of MSM received anal swab in the past 12 months |
| 80.26 | 70.89 | 88.10 | %, proportion of MSM received urine test in the past 12 months |
| 29.95 | 26.49 | 32.36 | %, proportion of 'oral sex and anal sex' in the same sex episode |
| 71.65 | 70.30 | 72.27 | %, proportion of 'oral sex and rimming' in the same sex episode |
| 68.47 | 67.18 | 70.08 | %, proportion of saliva use during anal sex |
| 80.00 | 80.00 | 80.00 | %, proportion of receptive oral sex followed by partner's insertive anal sex |
| 80.00 | 80.00 | 80.00 | %, proportion of insertive oral sex followed by partner's insertive rimming |
| 1.04 | 0.49 | 1.41 | %, Infection prevalence of chlamydia trachomatis only at Oropharynx |
| 2.68 | 1.75 | 3.62 | %, Infection prevalence of chlamydia trachomatis only at Urethral |
| 6.95 | 5.09 | 8.46 | %, Infection prevalence of chlamydia trachomatis only at Rectum |
| 0.00 | 0.00 | 0.00 | %, Infection prevalence of chlamydia trachomatis at Oropharynx & Urethra |
| 1.78 | 1.10 | 2.60 | %, Infection prevalence of chlamydia trachomatis at Oropharynx & Rectum |
| 1.35 | 0.67 | 1.95 | %, Infection prevalence of chlamydia trachomatis at Urethra & Rectum |
| 0.00 | 0.00 | 0.00 | %, Infection prevalence of chlamydia trachomatis at Oropharynx & Urethra & Rectum |

**Table S8. 110.** Model 16’ output parameters with 95% confidence intervals

| **Mean** | **95%CI: lower** | **95%CI: upper** | **Parameters** |
| --- | --- | --- | --- |
| 46.24 | 42.48 | 52.12 | %, Consistent condom usage in anal sex in past 12 months |
| 87.46 | 84.72 | 91.24 | %, Condom efficacy in preventing transmission |
| 6.15 | 2.29 | 10.96 | Times, Frequency of kissing in the past 12 months |
| 12.69 | 5.54 | 20.66 | Times, Frequency of oral sex in the past 12 months |
| 42.06 | 30.19 | 63.89 | Times, Frequency of rimming in the past 12 months |
| 23.84 | 13.09 | 33.23 | Times, Frequency of anal sex in the past 12 months |
| 149.73 | 63.52 | 189.45 | weeks, Infection duration of chlamydia trachomatis at throat(asymptomatic infection) |
| 1.51 | 1.16 | 1.73 | weeks, Infection duration at urethral (symptomatic infection) |
| 85.41 | 82.76 | 87.57 | %, proportion of urethral infections that are asymptomatic |
| 79.63 | 69.39 | 90.76 | %, proportion of anal infections that are asymptomatic |
| 27.58 | 15.69 | 35.66 | weeks, Infection duration at urethral (asymptomatic infection) |
| 50.13 | 48.55 | 51.02 | weeks, Infection duration of chlamydia trachomatis at anus (asymptomatic infection) |
| 1.46 | 1.25 | 1.80 | weeks, Infection duration of chlamydia trachomatis at anus (symptomatic infection) |
| 82.26 | 74.12 | 89.34 | %, proportion of MSM received throat swab in the past 12 months |
| 78.52 | 70.52 | 86.16 | %, proportion of MSM received anal swab in the past 12 months |
| 80.58 | 71.36 | 86.45 | %, proportion of MSM received urine test in the past 12 months |
| 29.96 | 26.94 | 32.07 | %, proportion of 'oral sex and anal sex' in the same sex episode |
| 71.44 | 70.33 | 72.16 | %, proportion of 'oral sex and rimming' in the same sex episode |
| 68.82 | 67.22 | 69.92 | %, proportion of saliva use during anal sex |
| 80.00 | 80.00 | 80.00 | %, proportion of receptive oral sex followed by partner's insertive anal sex |
| 80.00 | 80.00 | 80.00 | %, proportion of insertive oral sex followed by partner's insertive rimming |
| 0.85 | 0.56 | 1.40 | %, Infection prevalence of chlamydia trachomatis only at Oropharynx |
| 2.62 | 1.78 | 3.52 | %, Infection prevalence of chlamydia trachomatis only at Urethral |
| 7.07 | 5.24 | 8.34 | %, Infection prevalence of chlamydia trachomatis only at Rectum |
| 0.00 | 0.00 | 0.00 | %, Infection prevalence of chlamydia trachomatis at Oropharynx & Urethra |
| 1.79 | 1.28 | 2.52 | %, Infection prevalence of chlamydia trachomatis at Oropharynx & Rectum |
| 1.48 | 0.68 | 1.91 | %, Infection prevalence of chlamydia trachomatis at Urethra & Rectum |
| 0.00 | 0.00 | 0.00 | %, Infection prevalence of chlamydia trachomatis at Oropharynx & Urethra & Rectum |

**Table S8. 111.** Model 17’ output parameters with 95% confidence intervals

| **Mean** | **95%CI: lower** | **95%CI: upper** | **Parameters** |
| --- | --- | --- | --- |
| 48.18 | 43.36 | 51.41 | %, Consistent condom usage in anal sex in past 12 months |
| 87.72 | 85.15 | 90.86 | %, Condom efficacy in preventing transmission |
| 6.67 | 3.26 | 10.30 | Times, Frequency of kissing in the past 12 months |
| 14.62 | 7.61 | 20.33 | Times, Frequency of oral sex in the past 12 months |
| 45.10 | 31.05 | 62.42 | Times, Frequency of rimming in the past 12 months |
| 22.17 | 13.85 | 30.43 | Times, Frequency of anal sex in the past 12 months |
| 142.60 | 77.33 | 184.44 | weeks, Infection duration of chlamydia trachomatis at throat(asymptomatic infection) |
| 1.50 | 1.25 | 1.72 | weeks, Infection duration at urethral (symptomatic infection) |
| 85.00 | 83.03 | 87.36 | %, proportion of urethral infections that are asymptomatic |
| 79.20 | 71.46 | 89.81 | %, proportion of anal infections that are asymptomatic |
| 25.58 | 16.47 | 34.17 | weeks, Infection duration at urethral (asymptomatic infection) |
| 49.92 | 48.79 | 51.01 | weeks, Infection duration of chlamydia trachomatis at anus (asymptomatic infection) |
| 1.55 | 1.30 | 1.76 | weeks, Infection duration of chlamydia trachomatis at anus (symptomatic infection) |
| 81.70 | 75.43 | 88.91 | %, proportion of MSM received throat swab in the past 12 months |
| 79.04 | 71.54 | 85.20 | %, proportion of MSM received anal swab in the past 12 months |
| 78.41 | 71.97 | 85.61 | %, proportion of MSM received urine test in the past 12 months |
| 29.60 | 27.23 | 31.98 | %, proportion of 'oral sex and anal sex' in the same sex episode |
| 71.15 | 70.63 | 72.05 | %, proportion of 'oral sex and rimming' in the same sex episode |
| 68.79 | 67.60 | 69.91 | %, proportion of saliva use during anal sex |
| 80.00 | 80.00 | 80.00 | %, proportion of receptive oral sex followed by partner's insertive anal sex |
| 80.00 | 80.00 | 80.00 | %, proportion of insertive oral sex followed by partner's insertive rimming |
| 0.80 | 0.59 | 1.35 | %, Infection prevalence of chlamydia trachomatis only at Oropharynx |
| 2.51 | 1.93 | 3.49 | %, Infection prevalence of chlamydia trachomatis only at Urethral |
| 6.71 | 5.28 | 8.10 | %, Infection prevalence of chlamydia trachomatis only at Rectum |
| 0.00 | 0.00 | 0.00 | %, Infection prevalence of chlamydia trachomatis at Oropharynx & Urethra |
| 1.75 | 1.36 | 2.46 | %, Infection prevalence of chlamydia trachomatis at Oropharynx & Rectum |
| 1.24 | 0.74 | 1.84 | %, Infection prevalence of chlamydia trachomatis at Urethra & Rectum |
| 0.00 | 0.00 | 0.00 | %, Infection prevalence of chlamydia trachomatis at Oropharynx & Urethra & Rectum |

**Table S8. 112.** Model 18’ output parameters with 95% confidence intervals

| **Mean** | **95%CI: lower** | **95%CI: upper** | **Parameters** |
| --- | --- | --- | --- |
| 47.94 | 44.13 | 51.09 | %, Consistent condom usage in anal sex in past 12 months |
| 87.60 | 85.59 | 90.07 | %, Condom efficacy in preventing transmission |
| 6.70 | 3.96 | 9.84 | Times, Frequency of kissing in the past 12 months |
| 15.01 | 8.77 | 20.06 | Times, Frequency of oral sex in the past 12 months |
| 46.75 | 31.75 | 59.62 | Times, Frequency of rimming in the past 12 months |
| 19.82 | 14.69 | 29.37 | Times, Frequency of anal sex in the past 12 months |
| 147.88 | 88.21 | 181.61 | weeks, Infection duration of chlamydia trachomatis at throat(asymptomatic infection) |
| 1.50 | 1.29 | 1.66 | weeks, Infection duration at urethral (symptomatic infection) |
| 84.64 | 83.24 | 86.70 | %, proportion of urethral infections that are asymptomatic |
| 80.89 | 72.30 | 89.29 | %, proportion of anal infections that are asymptomatic |
| 23.96 | 18.15 | 33.31 | weeks, Infection duration at urethral (asymptomatic infection) |
| 49.59 | 48.90 | 50.74 | weeks, Infection duration of chlamydia trachomatis at anus (asymptomatic infection) |
| 1.46 | 1.29 | 1.69 | weeks, Infection duration of chlamydia trachomatis at anus (symptomatic infection) |
| 82.07 | 76.57 | 87.31 | %, proportion of MSM received throat swab in the past 12 months |
| 79.11 | 72.52 | 84.16 | %, proportion of MSM received anal swab in the past 12 months |
| 76.46 | 72.62 | 84.73 | %, proportion of MSM received urine test in the past 12 months |
| 29.43 | 27.36 | 31.68 | %, proportion of 'oral sex and anal sex' in the same sex episode |
| 71.23 | 70.74 | 71.86 | %, proportion of 'oral sex and rimming' in the same sex episode |
| 68.41 | 67.68 | 69.66 | %, proportion of saliva use during anal sex |
| 80.00 | 80.00 | 80.00 | %, proportion of receptive oral sex followed by partner's insertive anal sex |
| 80.00 | 80.00 | 80.00 | %, proportion of insertive oral sex followed by partner's insertive rimming |
| 0.91 | 0.66 | 1.19 | %, Infection prevalence of chlamydia trachomatis only at Oropharynx |
| 2.77 | 1.98 | 3.36 | %, Infection prevalence of chlamydia trachomatis only at Urethral |
| 6.75 | 5.44 | 7.86 | %, Infection prevalence of chlamydia trachomatis only at Rectum |
| 0.00 | 0.00 | 0.00 | %, Infection prevalence of chlamydia trachomatis at Oropharynx & Urethra |
| 1.98 | 1.41 | 2.33 | %, Infection prevalence of chlamydia trachomatis at Oropharynx & Rectum |
| 1.40 | 0.91 | 1.74 | %, Infection prevalence of chlamydia trachomatis at Urethra & Rectum |
| 0.00 | 0.00 | 0.00 | %, Infection prevalence of chlamydia trachomatis at Oropharynx & Urethra & Rectum |

**Table S8. 113.** Model 19’ output parameters with 95% confidence intervals

| **Mean** | **95%CI: lower** | **95%CI: upper** | **Parameters** |
| --- | --- | --- | --- |
| 47.87 | 44.83 | 50.74 | %, Consistent condom usage in anal sex in past 12 months |
| 87.82 | 86.50 | 89.65 | %, Condom efficacy in preventing transmission |
| 6.55 | 4.21 | 9.46 | Times, Frequency of kissing in the past 12 months |
| 13.10 | 8.83 | 18.84 | Times, Frequency of oral sex in the past 12 months |
| 48.05 | 33.39 | 56.12 | Times, Frequency of rimming in the past 12 months |
| 21.40 | 16.01 | 28.99 | Times, Frequency of anal sex in the past 12 months |
| 148.76 | 102.90 | 179.15 | weeks, Infection duration of chlamydia trachomatis at throat(asymptomatic infection) |
| 1.46 | 1.29 | 1.63 | weeks, Infection duration at urethral (symptomatic infection) |
| 85.21 | 83.56 | 86.56 | %, proportion of urethral infections that are asymptomatic |
| 78.10 | 72.75 | 89.06 | %, proportion of anal infections that are asymptomatic |
| 23.03 | 18.64 | 33.13 | weeks, Infection duration at urethral (asymptomatic infection) |
| 49.98 | 48.93 | 50.73 | weeks, Infection duration of chlamydia trachomatis at anus (asymptomatic infection) |
| 1.47 | 1.31 | 1.64 | weeks, Infection duration of chlamydia trachomatis at anus (symptomatic infection) |
| 81.84 | 76.89 | 86.25 | %, proportion of MSM received throat swab in the past 12 months |
| 78.70 | 72.69 | 83.89 | %, proportion of MSM received anal swab in the past 12 months |
| 78.26 | 73.42 | 84.34 | %, proportion of MSM received urine test in the past 12 months |
| 30.44 | 27.57 | 31.68 | %, proportion of 'oral sex and anal sex' in the same sex episode |
| 71.40 | 70.85 | 71.82 | %, proportion of 'oral sex and rimming' in the same sex episode |
| 68.70 | 67.75 | 69.45 | %, proportion of saliva use during anal sex |
| 80.00 | 80.00 | 80.00 | %, proportion of receptive oral sex followed by partner's insertive anal sex |
| 80.00 | 80.00 | 80.00 | %, proportion of insertive oral sex followed by partner's insertive rimming |
| 0.94 | 0.68 | 1.18 | %, Infection prevalence of chlamydia trachomatis only at Oropharynx |
| 2.66 | 2.03 | 3.26 | %, Infection prevalence of chlamydia trachomatis only at Urethral |
| 6.96 | 5.78 | 7.88 | %, Infection prevalence of chlamydia trachomatis only at Rectum |
| 0.00 | 0.00 | 0.00 | %, Infection prevalence of chlamydia trachomatis at Oropharynx & Urethra |
| 1.83 | 1.50 | 2.24 | %, Infection prevalence of chlamydia trachomatis at Oropharynx & Rectum |
| 1.24 | 0.94 | 1.66 | %, Infection prevalence of chlamydia trachomatis at Urethra & Rectum |
| 0.00 | 0.00 | 0.00 | %, Infection prevalence of chlamydia trachomatis at Oropharynx & Urethra & Rectum |

**Table S8. 114.** Model 20’ output parameters with 95% confidence intervals

| **Mean** | **95%CI: lower** | **95%CI: upper** | **Parameters** |
| --- | --- | --- | --- |
| 47.57 | 45.26 | 50.15 | %, Consistent condom usage in anal sex in past 12 months |
| 88.19 | 86.65 | 89.45 | %, Condom efficacy in preventing transmission |
| 6.52 | 4.65 | 8.40 | Times, Frequency of kissing in the past 12 months |
| 12.96 | 9.56 | 17.42 | Times, Frequency of oral sex in the past 12 months |
| 44.62 | 33.83 | 54.81 | Times, Frequency of rimming in the past 12 months |
| 22.50 | 17.05 | 27.79 | Times, Frequency of anal sex in the past 12 months |
| 142.27 | 109.78 | 176.12 | weeks, Infection duration of chlamydia trachomatis at throat(asymptomatic infection) |
| 1.49 | 1.30 | 1.62 | weeks, Infection duration at urethral (symptomatic infection) |
| 85.08 | 83.80 | 86.51 | %, proportion of urethral infections that are asymptomatic |
| 79.91 | 72.86 | 88.18 | %, proportion of anal infections that are asymptomatic |
| 25.61 | 19.61 | 31.40 | weeks, Infection duration at urethral (asymptomatic infection) |
| 49.85 | 49.02 | 50.67 | weeks, Infection duration of chlamydia trachomatis at anus (asymptomatic infection) |
| 1.44 | 1.36 | 1.61 | weeks, Infection duration of chlamydia trachomatis at anus (symptomatic infection) |
| 81.09 | 77.23 | 85.21 | %, proportion of MSM received throat swab in the past 12 months |
| 77.64 | 73.70 | 83.55 | %, proportion of MSM received anal swab in the past 12 months |
| 77.84 | 74.10 | 82.86 | %, proportion of MSM received urine test in the past 12 months |
| 29.75 | 28.12 | 31.27 | %, proportion of 'oral sex and anal sex' in the same sex episode |
| 71.29 | 70.86 | 71.76 | %, proportion of 'oral sex and rimming' in the same sex episode |
| 68.53 | 67.94 | 69.44 | %, proportion of saliva use during anal sex |
| 80.00 | 80.00 | 80.00 | %, proportion of receptive oral sex followed by partner's insertive anal sex |
| 80.00 | 80.00 | 80.00 | %, proportion of insertive oral sex followed by partner's insertive rimming |
| 0.90 | 0.69 | 1.14 | %, Infection prevalence of chlamydia trachomatis only at Oropharynx |
| 2.72 | 2.05 | 3.25 | %, Infection prevalence of chlamydia trachomatis only at Urethral |
| 6.78 | 5.81 | 7.73 | %, Infection prevalence of chlamydia trachomatis only at Rectum |
| 0.00 | 0.00 | 0.00 | %, Infection prevalence of chlamydia trachomatis at Oropharynx & Urethra |
| 1.94 | 1.59 | 2.22 | %, Infection prevalence of chlamydia trachomatis at Oropharynx & Rectum |
| 1.25 | 0.96 | 1.59 | %, Infection prevalence of chlamydia trachomatis at Urethra & Rectum |
| 0.00 | 0.00 | 0.00 | %, Infection prevalence of chlamydia trachomatis at Oropharynx & Urethra & Rectum |

Model output parameters (Published validation dataset3): Data from MSM surveillance data of all Dutch STI clinics

**Table S8. 115.** Model 3’ output parameters with 95% confidence intervals

| **Mean** | **95%CI: lower** | **95%CI: upper** | **Parameters** |
| --- | --- | --- | --- |
| 48.95 | 35.37 | 58.33 | %, Consistent condom usage in anal sex in past 12 months |
| 86.49 | 80.19 | 93.86 | %, Condom efficacy in preventing transmission |
| 7.49 | 2.15 | 12.39 | Times, Frequency of kissing in the past 12 months |
| 7.69 | 1.04 | 23.11 | Times, Frequency of oral sex in the past 12 months |
| 17.77 | 1.91 | 75.23 | Times, Frequency of rimming in the past 12 months |
| 13.46 | 0.47 | 50.79 | Times, Frequency of anal sex in the past 12 months |
| 122.89 | 45.61 | 234.07 | weeks, Infection duration of chlamydia trachomatis at throat(asymptomatic infection) |
| 1.53 | 1.16 | 1.96 | weeks, Infection duration at urethral (symptomatic infection) |
| 100.00 | 100.00 | 100.00 | %, proportion of urethral infections that are asymptomatic |
| 100.00 | 100.00 | 100.00 | %, proportion of anal infections that are asymptomatic |
| 31.33 | 14.93 | 49.24 | weeks, Infection duration at urethral (asymptomatic infection) |
| 50.14 | 48.27 | 51.75 | weeks, Infection duration of chlamydia trachomatis at anus (asymptomatic infection) |
| 1.39 | 1.07 | 1.97 | weeks, Infection duration of chlamydia trachomatis at anus (symptomatic infection) |
| 77.57 | 66.36 | 94.77 | %, proportion of MSM received throat swab in the past 12 months |
| 77.07 | 64.78 | 93.65 | %, proportion of MSM received anal swab in the past 12 months |
| 76.06 | 65.04 | 92.27 | %, proportion of MSM received urine test in the past 12 months |
| 29.39 | 26.38 | 32.91 | %, proportion of 'oral sex and anal sex' in the same sex episode |
| 71.18 | 70.04 | 72.72 | %, proportion of 'oral sex and rimming' in the same sex episode |
| 68.48 | 66.26 | 70.12 | %, proportion of saliva use during anal sex |
| 80.00 | 80.00 | 80.00 | %, proportion of receptive oral sex followed by partner's insertive anal sex |
| 80.00 | 80.00 | 80.00 | %, proportion of insertive oral sex followed by partner's insertive rimming |
| 0.51 | 0.48 | 0.54 | %, Infection prevalence of chlamydia trachomatis only at Oropharynx |
| 2.31 | 2.24 | 2.36 | %, Infection prevalence of chlamydia trachomatis only at Urethral |
| 6.32 | 6.18 | 6.39 | %, Infection prevalence of chlamydia trachomatis only at Rectum |
| 0.06 | 0.05 | 0.07 | %, Infection prevalence of chlamydia trachomatis at Oropharynx & Urethra |
| 0.54 | 0.51 | 0.58 | %, Infection prevalence of chlamydia trachomatis at Oropharynx & Rectum |
| 1.03 | 0.98 | 1.06 | %, Infection prevalence of chlamydia trachomatis at Urethra & Rectum |
| 0.11 | 0.09 | 0.12 | %, Infection prevalence of chlamydia trachomatis at Oropharynx & Urethra & Rectum |

**Table S8. 116.** Model 14’ output parameters with 95% confidence intervals

| **Mean** | **95%CI: lower** | **95%CI: upper** | **Parameters** |
| --- | --- | --- | --- |
| 46.38 | 36.28 | 57.83 | %, Consistent condom usage in anal sex in past 12 months |
| 87.29 | 80.81 | 92.68 | %, Condom efficacy in preventing transmission |
| 7.54 | 3.39 | 11.77 | Times, Frequency of kissing in the past 12 months |
| 8.32 | 1.86 | 20.84 | Times, Frequency of oral sex in the past 12 months |
| 42.21 | 10.14 | 75.18 | Times, Frequency of rimming in the past 12 months |
| 8.87 | 0.70 | 26.63 | Times, Frequency of anal sex in the past 12 months |
| 186.54 | 77.88 | 230.52 | weeks, Infection duration of chlamydia trachomatis at throat(asymptomatic infection) |
| 1.54 | 1.21 | 1.93 | weeks, Infection duration at urethral (symptomatic infection) |
| 100.00 | 100.00 | 100.00 | %, proportion of urethral infections that are asymptomatic |
| 100.00 | 100.00 | 100.00 | %, proportion of anal infections that are asymptomatic |
| 36.22 | 17.39 | 48.69 | weeks, Infection duration at urethral (asymptomatic infection) |
| 49.92 | 48.29 | 51.51 | weeks, Infection duration of chlamydia trachomatis at anus (asymptomatic infection) |
| 1.47 | 1.13 | 1.92 | weeks, Infection duration of chlamydia trachomatis at anus (symptomatic infection) |
| 79.87 | 70.27 | 92.05 | %, proportion of MSM received throat swab in the past 12 months |
| 80.99 | 69.99 | 91.47 | %, proportion of MSM received anal swab in the past 12 months |
| 78.04 | 67.78 | 89.93 | %, proportion of MSM received urine test in the past 12 months |
| 30.59 | 26.55 | 32.54 | %, proportion of 'oral sex and anal sex' in the same sex episode |
| 71.33 | 70.36 | 72.61 | %, proportion of 'oral sex and rimming' in the same sex episode |
| 68.28 | 66.47 | 70.19 | %, proportion of saliva use during anal sex |
| 80.00 | 80.00 | 80.00 | %, proportion of receptive oral sex followed by partner's insertive anal sex |
| 80.00 | 80.00 | 80.00 | %, proportion of insertive oral sex followed by partner's insertive rimming |
| 0.51 | 0.49 | 0.54 | %, Infection prevalence of chlamydia trachomatis only at Oropharynx |
| 2.29 | 2.24 | 2.34 | %, Infection prevalence of chlamydia trachomatis only at Urethral |
| 6.29 | 6.19 | 6.38 | %, Infection prevalence of chlamydia trachomatis only at Rectum |
| 0.06 | 0.05 | 0.07 | %, Infection prevalence of chlamydia trachomatis at Oropharynx & Urethra |
| 0.55 | 0.51 | 0.58 | %, Infection prevalence of chlamydia trachomatis at Oropharynx & Rectum |
| 1.02 | 0.99 | 1.05 | %, Infection prevalence of chlamydia trachomatis at Urethra & Rectum |
| 0.11 | 0.09 | 0.12 | %, Infection prevalence of chlamydia trachomatis at Oropharynx & Urethra & Rectum |

**Table S8. 117.** Model 15’ output parameters with 95% confidence intervals

| **Mean** | **95%CI: lower** | **95%CI: upper** | **Parameters** |
| --- | --- | --- | --- |
| 47.91 | 37.94 | 55.20 | %, Consistent condom usage in anal sex in past 12 months |
| 87.27 | 81.58 | 91.89 | %, Condom efficacy in preventing transmission |
| 7.62 | 3.87 | 11.25 | Times, Frequency of kissing in the past 12 months |
| 9.00 | 3.66 | 18.68 | Times, Frequency of oral sex in the past 12 months |
| 25.84 | 10.58 | 64.28 | Times, Frequency of rimming in the past 12 months |
| 21.11 | 9.50 | 26.14 | Times, Frequency of anal sex in the past 12 months |
| 150.83 | 83.43 | 229.44 | weeks, Infection duration of chlamydia trachomatis at throat(asymptomatic infection) |
| 1.61 | 1.33 | 1.85 | weeks, Infection duration at urethral (symptomatic infection) |
| 100.00 | 100.00 | 100.00 | %, proportion of urethral infections that are asymptomatic |
| 100.00 | 100.00 | 100.00 | %, proportion of anal infections that are asymptomatic |
| 24.12 | 17.40 | 34.27 | weeks, Infection duration at urethral (asymptomatic infection) |
| 49.73 | 48.86 | 50.93 | weeks, Infection duration of chlamydia trachomatis at anus (asymptomatic infection) |
| 1.53 | 1.17 | 1.86 | weeks, Infection duration of chlamydia trachomatis at anus (symptomatic infection) |
| 81.95 | 74.60 | 90.44 | %, proportion of MSM received throat swab in the past 12 months |
| 77.27 | 70.88 | 89.02 | %, proportion of MSM received anal swab in the past 12 months |
| 77.75 | 69.30 | 85.99 | %, proportion of MSM received urine test in the past 12 months |
| 30.50 | 27.23 | 32.33 | %, proportion of 'oral sex and anal sex' in the same sex episode |
| 71.50 | 70.53 | 72.39 | %, proportion of 'oral sex and rimming' in the same sex episode |
| 68.32 | 66.61 | 69.81 | %, proportion of saliva use during anal sex |
| 80.00 | 80.00 | 80.00 | %, proportion of receptive oral sex followed by partner's insertive anal sex |
| 80.00 | 80.00 | 80.00 | %, proportion of insertive oral sex followed by partner's insertive rimming |
| 0.52 | 0.49 | 0.54 | %, Infection prevalence of chlamydia trachomatis only at Oropharynx |
| 2.29 | 2.25 | 2.33 | %, Infection prevalence of chlamydia trachomatis only at Urethral |
| 6.32 | 6.21 | 6.37 | %, Infection prevalence of chlamydia trachomatis only at Rectum |
| 0.06 | 0.05 | 0.07 | %, Infection prevalence of chlamydia trachomatis at Oropharynx & Urethra |
| 0.55 | 0.52 | 0.57 | %, Infection prevalence of chlamydia trachomatis at Oropharynx & Rectum |
| 1.03 | 0.99 | 1.05 | %, Infection prevalence of chlamydia trachomatis at Urethra & Rectum |
| 0.10 | 0.10 | 0.11 | %, Infection prevalence of chlamydia trachomatis at Oropharynx & Urethra & Rectum |

**Table S8. 118.** Model 16’ output parameters with 95% confidence intervals

| **Mean** | **95%CI: lower** | **95%CI: upper** | **Parameters** |
| --- | --- | --- | --- |
| 47.52 | 38.40 | 55.04 | %, Consistent condom usage in anal sex in past 12 months |
| 86.26 | 81.85 | 90.79 | %, Condom efficacy in preventing transmission |
| 7.43 | 4.00 | 10.01 | Times, Frequency of kissing in the past 12 months |
| 12.88 | 5.33 | 18.87 | Times, Frequency of oral sex in the past 12 months |
| 33.75 | 16.12 | 66.15 | Times, Frequency of rimming in the past 12 months |
| 14.51 | 10.64 | 19.20 | Times, Frequency of anal sex in the past 12 months |
| 162.03 | 95.91 | 225.76 | weeks, Infection duration of chlamydia trachomatis at throat(asymptomatic infection) |
| 1.53 | 1.36 | 1.80 | weeks, Infection duration at urethral (symptomatic infection) |
| 100.00 | 100.00 | 100.00 | %, proportion of urethral infections that are asymptomatic |
| 100.00 | 100.00 | 100.00 | %, proportion of anal infections that are asymptomatic |
| 26.60 | 19.36 | 33.75 | weeks, Infection duration at urethral (asymptomatic infection) |
| 50.02 | 48.93 | 50.89 | weeks, Infection duration of chlamydia trachomatis at anus (asymptomatic infection) |
| 1.45 | 1.20 | 1.85 | weeks, Infection duration of chlamydia trachomatis at anus (symptomatic infection) |
| 83.69 | 76.41 | 89.71 | %, proportion of MSM received throat swab in the past 12 months |
| 78.49 | 71.20 | 88.65 | %, proportion of MSM received anal swab in the past 12 months |
| 76.41 | 69.98 | 86.27 | %, proportion of MSM received urine test in the past 12 months |
| 28.88 | 27.53 | 31.02 | %, proportion of 'oral sex and anal sex' in the same sex episode |
| 71.29 | 70.54 | 72.32 | %, proportion of 'oral sex and rimming' in the same sex episode |
| 68.19 | 66.98 | 69.68 | %, proportion of saliva use during anal sex |
| 80.00 | 80.00 | 80.00 | %, proportion of receptive oral sex followed by partner's insertive anal sex |
| 80.00 | 80.00 | 80.00 | %, proportion of insertive oral sex followed by partner's insertive rimming |
| 0.51 | 0.50 | 0.53 | %, Infection prevalence of chlamydia trachomatis only at Oropharynx |
| 2.29 | 2.25 | 2.33 | %, Infection prevalence of chlamydia trachomatis only at Urethral |
| 6.28 | 6.21 | 6.33 | %, Infection prevalence of chlamydia trachomatis only at Rectum |
| 0.06 | 0.05 | 0.07 | %, Infection prevalence of chlamydia trachomatis at Oropharynx & Urethra |
| 0.54 | 0.53 | 0.56 | %, Infection prevalence of chlamydia trachomatis at Oropharynx & Rectum |
| 1.01 | 1.00 | 1.05 | %, Infection prevalence of chlamydia trachomatis at Urethra & Rectum |
| 0.11 | 0.10 | 0.11 | %, Infection prevalence of chlamydia trachomatis at Oropharynx & Urethra & Rectum |

**Table S8. 119.** Model 17’ output parameters with 95% confidence intervals

| **Mean** | **95%CI: lower** | **95%CI: upper** | **Parameters** |
| --- | --- | --- | --- |
| 47.18 | 41.21 | 54.90 | %, Consistent condom usage in anal sex in past 12 months |
| 86.56 | 83.09 | 89.64 | %, Condom efficacy in preventing transmission |
| 7.02 | 4.76 | 9.36 | Times, Frequency of kissing in the past 12 months |
| 8.56 | 5.60 | 14.40 | Times, Frequency of oral sex in the past 12 months |
| 42.64 | 23.05 | 62.12 | Times, Frequency of rimming in the past 12 months |
| 15.23 | 11.89 | 18.11 | Times, Frequency of anal sex in the past 12 months |
| 183.48 | 138.77 | 216.10 | weeks, Infection duration of chlamydia trachomatis at throat(asymptomatic infection) |
| 1.52 | 1.42 | 1.64 | weeks, Infection duration at urethral (symptomatic infection) |
| 100.00 | 100.00 | 100.00 | %, proportion of urethral infections that are asymptomatic |
| 100.00 | 100.00 | 100.00 | %, proportion of anal infections that are asymptomatic |
| 26.37 | 21.03 | 31.89 | weeks, Infection duration at urethral (asymptomatic infection) |
| 50.04 | 49.21 | 50.82 | weeks, Infection duration of chlamydia trachomatis at anus (asymptomatic infection) |
| 1.53 | 1.28 | 1.80 | weeks, Infection duration of chlamydia trachomatis at anus (symptomatic infection) |
| 82.72 | 77.18 | 87.84 | %, proportion of MSM received throat swab in the past 12 months |
| 81.56 | 72.95 | 86.72 | %, proportion of MSM received anal swab in the past 12 months |
| 76.93 | 71.72 | 85.02 | %, proportion of MSM received urine test in the past 12 months |
| 29.84 | 28.18 | 30.99 | %, proportion of 'oral sex and anal sex' in the same sex episode |
| 71.45 | 70.80 | 72.16 | %, proportion of 'oral sex and rimming' in the same sex episode |
| 67.89 | 67.17 | 68.94 | %, proportion of saliva use during anal sex |
| 80.00 | 80.00 | 80.00 | %, proportion of receptive oral sex followed by partner's insertive anal sex |
| 80.00 | 80.00 | 80.00 | %, proportion of insertive oral sex followed by partner's insertive rimming |
| 0.52 | 0.50 | 0.53 | %, Infection prevalence of chlamydia trachomatis only at Oropharynx |
| 2.29 | 2.26 | 2.32 | %, Infection prevalence of chlamydia trachomatis only at Urethral |
| 6.27 | 6.22 | 6.32 | %, Infection prevalence of chlamydia trachomatis only at Rectum |
| 0.06 | 0.05 | 0.07 | %, Infection prevalence of chlamydia trachomatis at Oropharynx & Urethra |
| 0.55 | 0.53 | 0.56 | %, Infection prevalence of chlamydia trachomatis at Oropharynx & Rectum |
| 1.02 | 1.00 | 1.04 | %, Infection prevalence of chlamydia trachomatis at Urethra & Rectum |
| 0.10 | 0.10 | 0.11 | %, Infection prevalence of chlamydia trachomatis at Oropharynx & Urethra & Rectum |

**Table S8. 120.** Model 18’ output parameters with 95% confidence intervals

| **Mean** | **95%CI: lower** | **95%CI: upper** | **Parameters** |
| --- | --- | --- | --- |
| 48.43 | 41.65 | 54.35 | %, Consistent condom usage in anal sex in past 12 months |
| 86.58 | 83.14 | 88.88 | %, Condom efficacy in preventing transmission |
| 6.78 | 4.88 | 9.07 | Times, Frequency of kissing in the past 12 months |
| 9.23 | 6.11 | 14.30 | Times, Frequency of oral sex in the past 12 months |
| 42.60 | 24.64 | 62.07 | Times, Frequency of rimming in the past 12 months |
| 14.40 | 12.29 | 16.88 | Times, Frequency of anal sex in the past 12 months |
| 175.04 | 141.00 | 202.26 | weeks, Infection duration of chlamydia trachomatis at throat(asymptomatic infection) |
| 1.53 | 1.42 | 1.63 | weeks, Infection duration at urethral (symptomatic infection) |
| 100.00 | 100.00 | 100.00 | %, proportion of urethral infections that are asymptomatic |
| 100.00 | 100.00 | 100.00 | %, proportion of anal infections that are asymptomatic |
| 27.68 | 21.96 | 31.19 | weeks, Infection duration at urethral (asymptomatic infection) |
| 49.92 | 49.28 | 50.78 | weeks, Infection duration of chlamydia trachomatis at anus (asymptomatic infection) |
| 1.56 | 1.30 | 1.78 | weeks, Infection duration of chlamydia trachomatis at anus (symptomatic infection) |
| 82.40 | 77.38 | 87.17 | %, proportion of MSM received throat swab in the past 12 months |
| 80.95 | 73.67 | 85.73 | %, proportion of MSM received anal swab in the past 12 months |
| 77.95 | 72.70 | 84.24 | %, proportion of MSM received urine test in the past 12 months |
| 29.54 | 28.56 | 30.73 | %, proportion of 'oral sex and anal sex' in the same sex episode |
| 71.51 | 70.88 | 72.13 | %, proportion of 'oral sex and rimming' in the same sex episode |
| 68.08 | 67.38 | 68.83 | %, proportion of saliva use during anal sex |
| 80.00 | 80.00 | 80.00 | %, proportion of receptive oral sex followed by partner's insertive anal sex |
| 80.00 | 80.00 | 80.00 | %, proportion of insertive oral sex followed by partner's insertive rimming |
| 0.51 | 0.50 | 0.52 | %, Infection prevalence of chlamydia trachomatis only at Oropharynx |
| 2.29 | 2.26 | 2.32 | %, Infection prevalence of chlamydia trachomatis only at Urethral |
| 6.27 | 6.22 | 6.31 | %, Infection prevalence of chlamydia trachomatis only at Rectum |
| 0.06 | 0.06 | 0.07 | %, Infection prevalence of chlamydia trachomatis at Oropharynx & Urethra |
| 0.55 | 0.54 | 0.56 | %, Infection prevalence of chlamydia trachomatis at Oropharynx & Rectum |
| 1.02 | 1.00 | 1.04 | %, Infection prevalence of chlamydia trachomatis at Urethra & Rectum |
| 0.11 | 0.10 | 0.11 | %, Infection prevalence of chlamydia trachomatis at Oropharynx & Urethra & Rectum |

**Table S8. 121.** Model 19’ output parameters with 95% confidence intervals

| **Mean** | **95%CI: lower** | **95%CI: upper** | **Parameters** |
| --- | --- | --- | --- |
| 49.93 | 43.00 | 53.94 | %, Consistent condom usage in anal sex in past 12 months |
| 86.84 | 83.45 | 88.63 | %, Condom efficacy in preventing transmission |
| 7.23 | 5.22 | 8.69 | Times, Frequency of kissing in the past 12 months |
| 8.56 | 6.34 | 12.17 | Times, Frequency of oral sex in the past 12 months |
| 35.11 | 24.49 | 58.64 | Times, Frequency of rimming in the past 12 months |
| 15.18 | 12.89 | 16.71 | Times, Frequency of anal sex in the past 12 months |
| 175.72 | 143.31 | 201.67 | weeks, Infection duration of chlamydia trachomatis at throat(asymptomatic infection) |
| 1.50 | 1.45 | 1.61 | weeks, Infection duration at urethral (symptomatic infection) |
| 100.00 | 100.00 | 100.00 | %, proportion of urethral infections that are asymptomatic |
| 100.00 | 100.00 | 100.00 | %, proportion of anal infections that are asymptomatic |
| 24.37 | 22.28 | 29.13 | weeks, Infection duration at urethral (asymptomatic infection) |
| 50.09 | 49.46 | 50.74 | weeks, Infection duration of chlamydia trachomatis at anus (asymptomatic infection) |
| 1.54 | 1.32 | 1.77 | weeks, Infection duration of chlamydia trachomatis at anus (symptomatic infection) |
| 83.38 | 77.58 | 87.16 | %, proportion of MSM received throat swab in the past 12 months |
| 78.09 | 74.03 | 84.93 | %, proportion of MSM received anal swab in the past 12 months |
| 77.87 | 73.20 | 83.52 | %, proportion of MSM received urine test in the past 12 months |
| 29.47 | 28.68 | 30.71 | %, proportion of 'oral sex and anal sex' in the same sex episode |
| 71.29 | 70.95 | 71.82 | %, proportion of 'oral sex and rimming' in the same sex episode |
| 68.00 | 67.39 | 68.77 | %, proportion of saliva use during anal sex |
| 80.00 | 80.00 | 80.00 | %, proportion of receptive oral sex followed by partner's insertive anal sex |
| 80.00 | 80.00 | 80.00 | %, proportion of insertive oral sex followed by partner's insertive rimming |
| 0.51 | 0.50 | 0.52 | %, Infection prevalence of chlamydia trachomatis only at Oropharynx |
| 2.30 | 2.27 | 2.32 | %, Infection prevalence of chlamydia trachomatis only at Urethral |
| 6.27 | 6.22 | 6.31 | %, Infection prevalence of chlamydia trachomatis only at Rectum |
| 0.06 | 0.06 | 0.07 | %, Infection prevalence of chlamydia trachomatis at Oropharynx & Urethra |
| 0.55 | 0.54 | 0.56 | %, Infection prevalence of chlamydia trachomatis at Oropharynx & Rectum |
| 1.02 | 1.00 | 1.04 | %, Infection prevalence of chlamydia trachomatis at Urethra & Rectum |
| 0.11 | 0.10 | 0.11 | %, Infection prevalence of chlamydia trachomatis at Oropharynx & Urethra & Rectum |

**Table S8. 122.** Model 20’ output parameters with 95% confidence intervals

| **Mean** | **95%CI: lower** | **95%CI: upper** | **Parameters** |
| --- | --- | --- | --- |
| 48.32 | 44.99 | 53.07 | %, Consistent condom usage in anal sex in past 12 months |
| 86.43 | 84.09 | 88.12 | %, Condom efficacy in preventing transmission |
| 6.82 | 5.64 | 8.19 | Times, Frequency of kissing in the past 12 months |
| 7.14 | 6.48 | 8.70 | Times, Frequency of oral sex in the past 12 months |
| 36.31 | 25.10 | 51.50 | Times, Frequency of rimming in the past 12 months |
| 15.53 | 13.97 | 16.61 | Times, Frequency of anal sex in the past 12 months |
| 181.14 | 157.47 | 196.74 | weeks, Infection duration of chlamydia trachomatis at throat(asymptomatic infection) |
| 1.51 | 1.46 | 1.60 | weeks, Infection duration at urethral (symptomatic infection) |
| 100.00 | 100.00 | 100.00 | %, proportion of urethral infections that are asymptomatic |
| 100.00 | 100.00 | 100.00 | %, proportion of anal infections that are asymptomatic |
| 25.58 | 22.67 | 29.06 | weeks, Infection duration at urethral (asymptomatic infection) |
| 50.17 | 49.61 | 50.67 | weeks, Infection duration of chlamydia trachomatis at anus (asymptomatic infection) |
| 1.55 | 1.36 | 1.75 | weeks, Infection duration of chlamydia trachomatis at anus (symptomatic infection) |
| 82.74 | 78.45 | 86.42 | %, proportion of MSM received throat swab in the past 12 months |
| 80.22 | 74.84 | 84.77 | %, proportion of MSM received anal swab in the past 12 months |
| 78.28 | 73.86 | 83.13 | %, proportion of MSM received urine test in the past 12 months |
| 29.72 | 28.90 | 30.67 | %, proportion of 'oral sex and anal sex' in the same sex episode |
| 71.37 | 71.09 | 71.71 | %, proportion of 'oral sex and rimming' in the same sex episode |
| 67.93 | 67.42 | 68.67 | %, proportion of saliva use during anal sex |
| 80.00 | 80.00 | 80.00 | %, proportion of receptive oral sex followed by partner's insertive anal sex |
| 80.00 | 80.00 | 80.00 | %, proportion of insertive oral sex followed by partner's insertive rimming |
| 0.51 | 0.51 | 0.52 | %, Infection prevalence of chlamydia trachomatis only at Oropharynx |
| 2.29 | 2.27 | 2.31 | %, Infection prevalence of chlamydia trachomatis only at Urethral |
| 6.27 | 6.23 | 6.31 | %, Infection prevalence of chlamydia trachomatis only at Rectum |
| 0.06 | 0.06 | 0.07 | %, Infection prevalence of chlamydia trachomatis at Oropharynx & Urethra |
| 0.55 | 0.54 | 0.55 | %, Infection prevalence of chlamydia trachomatis at Oropharynx & Rectum |
| 1.02 | 1.01 | 1.03 | %, Infection prevalence of chlamydia trachomatis at Urethra & Rectum |
| 0.11 | 0.10 | 0.11 | %, Infection prevalence of chlamydia trachomatis at Oropharynx & Urethra & Rectum |

Model output parameters (Published validation dataset4): Data from 1,610 community MSM in Thailand

**Table S8. 123.** Model 3’ output parameters with 95% confidence intervals

| **Mean** | **95%CI: lower** | **95%CI: upper** | **Parameters** |
| --- | --- | --- | --- |
| 48.71 | 36.37 | 58.50 | %, Consistent condom usage in anal sex in past 12 months |
| 88.76 | 80.42 | 93.61 | %, Condom efficacy in preventing transmission |
| 6.21 | 2.51 | 12.65 | Times, Frequency of kissing in the past 12 months |
| 10.18 | 1.63 | 27.70 | Times, Frequency of oral sex in the past 12 months |
| 36.43 | 2.50 | 73.10 | Times, Frequency of rimming in the past 12 months |
| 29.01 | 3.52 | 52.91 | Times, Frequency of anal sex in the past 12 months |
| 122.43 | 30.00 | 235.88 | weeks, Infection duration of chlamydia trachomatis at throat(asymptomatic infection) |
| 1.49 | 1.03 | 1.96 | weeks, Infection duration at urethral (symptomatic infection) |
| 100.00 | 100.00 | 100.00 | %, proportion of urethral infections that are asymptomatic |
| 100.00 | 100.00 | 100.00 | %, proportion of anal infections that are asymptomatic |
| 26.38 | 8.34 | 51.11 | weeks, Infection duration at urethral (asymptomatic infection) |
| 49.99 | 48.16 | 51.53 | weeks, Infection duration of chlamydia trachomatis at anus (asymptomatic infection) |
| 1.50 | 1.03 | 1.93 | weeks, Infection duration of chlamydia trachomatis at anus (symptomatic infection) |
| 78.27 | 66.85 | 94.51 | %, proportion of MSM received throat swab in the past 12 months |
| 82.84 | 64.67 | 94.93 | %, proportion of MSM received anal swab in the past 12 months |
| 80.26 | 65.38 | 90.25 | %, proportion of MSM received urine test in the past 12 months |
| 28.67 | 25.51 | 33.17 | %, proportion of 'oral sex and anal sex' in the same sex episode |
| 70.83 | 69.84 | 72.65 | %, proportion of 'oral sex and rimming' in the same sex episode |
| 69.21 | 66.18 | 70.85 | %, proportion of saliva use during anal sex |
| 80.00 | 80.00 | 80.00 | %, proportion of receptive oral sex followed by partner's insertive anal sex |
| 80.00 | 80.00 | 80.00 | %, proportion of insertive oral sex followed by partner's insertive rimming |
| 1.98 | 1.47 | 2.49 | %, Infection prevalence of chlamydia trachomatis only at Oropharynx |
| 4.65 | 3.91 | 5.89 | %, Infection prevalence of chlamydia trachomatis only at Urethral |
| 11.88 | 10.43 | 13.11 | %, Infection prevalence of chlamydia trachomatis only at Rectum |
| 0.23 | 0.05 | 0.38 | %, Infection prevalence of chlamydia trachomatis at Oropharynx & Urethra |
| 1.07 | 0.72 | 1.74 | %, Infection prevalence of chlamydia trachomatis at Oropharynx & Rectum |
| 1.92 | 1.45 | 2.64 | %, Infection prevalence of chlamydia trachomatis at Urethra & Rectum |
| 0.17 | 0.01 | 0.38 | %, Infection prevalence of chlamydia trachomatis at Oropharynx & Urethra & Rectum |

**Table S8. 124.** Model 14’ output parameters with 95% confidence intervals

| **Mean** | **95%CI: lower** | **95%CI: upper** | **Parameters** |
| --- | --- | --- | --- |
| 49.05 | 39.65 | 57.08 | %, Consistent condom usage in anal sex in past 12 months |
| 86.78 | 82.58 | 92.48 | %, Condom efficacy in preventing transmission |
| 8.01 | 3.88 | 11.99 | Times, Frequency of kissing in the past 12 months |
| 12.77 | 2.70 | 26.22 | Times, Frequency of oral sex in the past 12 months |
| 37.12 | 7.20 | 68.32 | Times, Frequency of rimming in the past 12 months |
| 27.24 | 6.12 | 42.40 | Times, Frequency of anal sex in the past 12 months |
| 145.93 | 39.37 | 223.98 | weeks, Infection duration of chlamydia trachomatis at throat(asymptomatic infection) |
| 1.56 | 1.17 | 1.90 | weeks, Infection duration at urethral (symptomatic infection) |
| 100.00 | 100.00 | 100.00 | %, proportion of urethral infections that are asymptomatic |
| 100.00 | 100.00 | 100.00 | %, proportion of anal infections that are asymptomatic |
| 29.63 | 12.80 | 44.46 | weeks, Infection duration at urethral (asymptomatic infection) |
| 49.77 | 48.40 | 51.29 | weeks, Infection duration of chlamydia trachomatis at anus (asymptomatic infection) |
| 1.65 | 1.12 | 1.88 | weeks, Infection duration of chlamydia trachomatis at anus (symptomatic infection) |
| 80.55 | 68.18 | 90.10 | %, proportion of MSM received throat swab in the past 12 months |
| 77.88 | 66.28 | 91.33 | %, proportion of MSM received anal swab in the past 12 months |
| 76.81 | 67.41 | 87.90 | %, proportion of MSM received urine test in the past 12 months |
| 29.70 | 26.19 | 32.47 | %, proportion of 'oral sex and anal sex' in the same sex episode |
| 71.26 | 70.11 | 72.59 | %, proportion of 'oral sex and rimming' in the same sex episode |
| 68.84 | 66.72 | 70.75 | %, proportion of saliva use during anal sex |
| 80.00 | 80.00 | 80.00 | %, proportion of receptive oral sex followed by partner's insertive anal sex |
| 80.00 | 80.00 | 80.00 | %, proportion of insertive oral sex followed by partner's insertive rimming |
| 1.90 | 1.57 | 2.33 | %, Infection prevalence of chlamydia trachomatis only at Oropharynx |
| 4.90 | 4.02 | 5.65 | %, Infection prevalence of chlamydia trachomatis only at Urethral |
| 11.45 | 10.56 | 12.75 | %, Infection prevalence of chlamydia trachomatis only at Rectum |
| 0.26 | 0.07 | 0.36 | %, Infection prevalence of chlamydia trachomatis at Oropharynx & Urethra |
| 1.27 | 0.81 | 1.62 | %, Infection prevalence of chlamydia trachomatis at Oropharynx & Rectum |
| 2.14 | 1.57 | 2.49 | %, Infection prevalence of chlamydia trachomatis at Urethra & Rectum |
| 0.25 | 0.05 | 0.37 | %, Infection prevalence of chlamydia trachomatis at Oropharynx & Urethra & Rectum |

**Table S8. 125.** Model 15’ output parameters with 95% confidence intervals

| **Mean** | **95%CI: lower** | **95%CI: upper** | **Parameters** |
| --- | --- | --- | --- |
| 48.70 | 39.66 | 56.31 | %, Consistent condom usage in anal sex in past 12 months |
| 87.93 | 82.59 | 92.39 | %, Condom efficacy in preventing transmission |
| 9.25 | 4.62 | 11.43 | Times, Frequency of kissing in the past 12 months |
| 16.37 | 3.73 | 24.80 | Times, Frequency of oral sex in the past 12 months |
| 29.74 | 7.77 | 66.20 | Times, Frequency of rimming in the past 12 months |
| 27.54 | 9.57 | 40.53 | Times, Frequency of anal sex in the past 12 months |
| 118.26 | 48.16 | 196.08 | weeks, Infection duration of chlamydia trachomatis at throat(asymptomatic infection) |
| 1.50 | 1.20 | 1.83 | weeks, Infection duration at urethral (symptomatic infection) |
| 100.00 | 100.00 | 100.00 | %, proportion of urethral infections that are asymptomatic |
| 100.00 | 100.00 | 100.00 | %, proportion of anal infections that are asymptomatic |
| 27.90 | 15.92 | 40.65 | weeks, Infection duration at urethral (asymptomatic infection) |
| 50.28 | 48.54 | 51.26 | weeks, Infection duration of chlamydia trachomatis at anus (asymptomatic infection) |
| 1.53 | 1.20 | 1.86 | weeks, Infection duration of chlamydia trachomatis at anus (symptomatic infection) |
| 81.53 | 71.59 | 88.96 | %, proportion of MSM received throat swab in the past 12 months |
| 77.45 | 66.34 | 90.82 | %, proportion of MSM received anal swab in the past 12 months |
| 75.99 | 69.98 | 85.48 | %, proportion of MSM received urine test in the past 12 months |
| 29.02 | 26.22 | 31.99 | %, proportion of 'oral sex and anal sex' in the same sex episode |
| 71.02 | 70.23 | 72.45 | %, proportion of 'oral sex and rimming' in the same sex episode |
| 68.25 | 66.90 | 70.43 | %, proportion of saliva use during anal sex |
| 80.00 | 80.00 | 80.00 | %, proportion of receptive oral sex followed by partner's insertive anal sex |
| 80.00 | 80.00 | 80.00 | %, proportion of insertive oral sex followed by partner's insertive rimming |
| 1.99 | 1.59 | 2.30 | %, Infection prevalence of chlamydia trachomatis only at Oropharynx |
| 4.66 | 4.06 | 5.42 | %, Infection prevalence of chlamydia trachomatis only at Urethral |
| 11.87 | 10.65 | 12.62 | %, Infection prevalence of chlamydia trachomatis only at Rectum |
| 0.23 | 0.09 | 0.32 | %, Infection prevalence of chlamydia trachomatis at Oropharynx & Urethra |
| 1.21 | 0.83 | 1.55 | %, Infection prevalence of chlamydia trachomatis at Oropharynx & Rectum |
| 2.05 | 1.58 | 2.40 | %, Infection prevalence of chlamydia trachomatis at Urethra & Rectum |
| 0.20 | 0.05 | 0.33 | %, Infection prevalence of chlamydia trachomatis at Oropharynx & Urethra & Rectum |

**Table S8. 126.** Model 16’ output parameters with 95% confidence intervals

| **Mean** | **95%CI: lower** | **95%CI: upper** | **Parameters** |
| --- | --- | --- | --- |
| 47.23 | 41.16 | 55.80 | %, Consistent condom usage in anal sex in past 12 months |
| 88.07 | 83.00 | 91.28 | %, Condom efficacy in preventing transmission |
| 7.00 | 4.77 | 11.33 | Times, Frequency of kissing in the past 12 months |
| 13.18 | 5.34 | 23.45 | Times, Frequency of oral sex in the past 12 months |
| 36.15 | 10.02 | 62.22 | Times, Frequency of rimming in the past 12 months |
| 22.58 | 11.48 | 38.16 | Times, Frequency of anal sex in the past 12 months |
| 119.67 | 51.13 | 192.61 | weeks, Infection duration of chlamydia trachomatis at throat(asymptomatic infection) |
| 1.44 | 1.21 | 1.77 | weeks, Infection duration at urethral (symptomatic infection) |
| 100.00 | 100.00 | 100.00 | %, proportion of urethral infections that are asymptomatic |
| 100.00 | 100.00 | 100.00 | %, proportion of anal infections that are asymptomatic |
| 27.52 | 18.64 | 39.69 | weeks, Infection duration at urethral (asymptomatic infection) |
| 49.76 | 48.77 | 51.12 | weeks, Infection duration of chlamydia trachomatis at anus (asymptomatic infection) |
| 1.61 | 1.28 | 1.83 | weeks, Infection duration of chlamydia trachomatis at anus (symptomatic infection) |
| 78.56 | 71.68 | 86.77 | %, proportion of MSM received throat swab in the past 12 months |
| 76.36 | 68.46 | 88.18 | %, proportion of MSM received anal swab in the past 12 months |
| 80.67 | 70.85 | 84.80 | %, proportion of MSM received urine test in the past 12 months |
| 29.60 | 26.67 | 31.88 | %, proportion of 'oral sex and anal sex' in the same sex episode |
| 71.24 | 70.33 | 72.28 | %, proportion of 'oral sex and rimming' in the same sex episode |
| 68.59 | 67.12 | 70.22 | %, proportion of saliva use during anal sex |
| 80.00 | 80.00 | 80.00 | %, proportion of receptive oral sex followed by partner's insertive anal sex |
| 80.00 | 80.00 | 80.00 | %, proportion of insertive oral sex followed by partner's insertive rimming |
| 1.94 | 1.69 | 2.24 | %, Infection prevalence of chlamydia trachomatis only at Oropharynx |
| 4.85 | 4.10 | 5.30 | %, Infection prevalence of chlamydia trachomatis only at Urethral |
| 11.91 | 10.68 | 12.59 | %, Infection prevalence of chlamydia trachomatis only at Rectum |
| 0.19 | 0.11 | 0.31 | %, Infection prevalence of chlamydia trachomatis at Oropharynx & Urethra |
| 1.25 | 0.98 | 1.52 | %, Infection prevalence of chlamydia trachomatis at Oropharynx & Rectum |
| 1.92 | 1.67 | 2.38 | %, Infection prevalence of chlamydia trachomatis at Urethra & Rectum |
| 0.19 | 0.08 | 0.31 | %, Infection prevalence of chlamydia trachomatis at Oropharynx & Urethra & Rectum |

**Table S8. 127.** Model 17’ output parameters with 95% confidence intervals

| **Mean** | **95%CI: lower** | **95%CI: upper** | **Parameters** |
| --- | --- | --- | --- |
| 50.88 | 42.63 | 55.27 | %, Consistent condom usage in anal sex in past 12 months |
| 87.14 | 83.60 | 90.73 | %, Condom efficacy in preventing transmission |
| 8.57 | 5.67 | 11.05 | Times, Frequency of kissing in the past 12 months |
| 15.23 | 5.85 | 23.40 | Times, Frequency of oral sex in the past 12 months |
| 36.38 | 10.07 | 57.12 | Times, Frequency of rimming in the past 12 months |
| 23.18 | 12.18 | 35.43 | Times, Frequency of anal sex in the past 12 months |
| 145.31 | 58.53 | 187.35 | weeks, Infection duration of chlamydia trachomatis at throat(asymptomatic infection) |
| 1.52 | 1.27 | 1.74 | weeks, Infection duration at urethral (symptomatic infection) |
| 100.00 | 100.00 | 100.00 | %, proportion of urethral infections that are asymptomatic |
| 100.00 | 100.00 | 100.00 | %, proportion of anal infections that are asymptomatic |
| 29.36 | 20.70 | 37.12 | weeks, Infection duration at urethral (asymptomatic infection) |
| 49.81 | 48.87 | 51.08 | weeks, Infection duration of chlamydia trachomatis at anus (asymptomatic infection) |
| 1.49 | 1.26 | 1.79 | weeks, Infection duration of chlamydia trachomatis at anus (symptomatic infection) |
| 77.90 | 72.91 | 84.67 | %, proportion of MSM received throat swab in the past 12 months |
| 79.00 | 71.11 | 87.62 | %, proportion of MSM received anal swab in the past 12 months |
| 77.32 | 71.33 | 84.01 | %, proportion of MSM received urine test in the past 12 months |
| 29.51 | 27.40 | 31.78 | %, proportion of 'oral sex and anal sex' in the same sex episode |
| 71.30 | 70.47 | 72.08 | %, proportion of 'oral sex and rimming' in the same sex episode |
| 68.68 | 67.47 | 69.97 | %, proportion of saliva use during anal sex |
| 80.00 | 80.00 | 80.00 | %, proportion of receptive oral sex followed by partner's insertive anal sex |
| 80.00 | 80.00 | 80.00 | %, proportion of insertive oral sex followed by partner's insertive rimming |
| 1.97 | 1.73 | 2.23 | %, Infection prevalence of chlamydia trachomatis only at Oropharynx |
| 4.62 | 4.10 | 5.21 | %, Infection prevalence of chlamydia trachomatis only at Urethral |
| 11.49 | 10.85 | 12.51 | %, Infection prevalence of chlamydia trachomatis only at Rectum |
| 0.21 | 0.11 | 0.29 | %, Infection prevalence of chlamydia trachomatis at Oropharynx & Urethra |
| 1.32 | 1.00 | 1.47 | %, Infection prevalence of chlamydia trachomatis at Oropharynx & Rectum |
| 2.05 | 1.70 | 2.30 | %, Infection prevalence of chlamydia trachomatis at Urethra & Rectum |
| 0.17 | 0.08 | 0.29 | %, Infection prevalence of chlamydia trachomatis at Oropharynx & Urethra & Rectum |

**Table S8.128.** Model 18’ output parameters with 95% confidence intervals

| **Mean** | **95%CI: lower** | **95%CI: upper** | **Parameters** |
| --- | --- | --- | --- |
| 48.32 | 43.44 | 54.37 | %, Consistent condom usage in anal sex in past 12 months |
| 86.24 | 83.75 | 90.53 | %, Condom efficacy in preventing transmission |
| 8.65 | 6.18 | 10.88 | Times, Frequency of kissing in the past 12 months |
| 13.39 | 6.60 | 22.79 | Times, Frequency of oral sex in the past 12 months |
| 31.97 | 13.29 | 54.68 | Times, Frequency of rimming in the past 12 months |
| 21.41 | 12.74 | 31.31 | Times, Frequency of anal sex in the past 12 months |
| 139.13 | 70.18 | 173.33 | weeks, Infection duration of chlamydia trachomatis at throat(asymptomatic infection) |
| 1.54 | 1.27 | 1.70 | weeks, Infection duration at urethral (symptomatic infection) |
| 100.00 | 100.00 | 100.00 | %, proportion of urethral infections that are asymptomatic |
| 100.00 | 100.00 | 100.00 | %, proportion of anal infections that are asymptomatic |
| 29.74 | 23.03 | 36.42 | weeks, Infection duration at urethral (asymptomatic infection) |
| 49.83 | 48.99 | 50.96 | weeks, Infection duration of chlamydia trachomatis at anus (asymptomatic infection) |
| 1.56 | 1.30 | 1.77 | weeks, Infection duration of chlamydia trachomatis at anus (symptomatic infection) |
| 76.88 | 72.88 | 83.06 | %, proportion of MSM received throat swab in the past 12 months |
| 80.43 | 72.55 | 86.63 | %, proportion of MSM received anal swab in the past 12 months |
| 78.81 | 71.87 | 83.98 | %, proportion of MSM received urine test in the past 12 months |
| 30.21 | 27.73 | 31.55 | %, proportion of 'oral sex and anal sex' in the same sex episode |
| 70.95 | 70.51 | 71.73 | %, proportion of 'oral sex and rimming' in the same sex episode |
| 68.81 | 67.72 | 69.83 | %, proportion of saliva use during anal sex |
| 80.00 | 80.00 | 80.00 | %, proportion of receptive oral sex followed by partner's insertive anal sex |
| 80.00 | 80.00 | 80.00 | %, proportion of insertive oral sex followed by partner's insertive rimming |
| 1.96 | 1.76 | 2.18 | %, Infection prevalence of chlamydia trachomatis only at Oropharynx |
| 4.75 | 4.24 | 5.08 | %, Infection prevalence of chlamydia trachomatis only at Urethral |
| 11.53 | 10.93 | 12.47 | %, Infection prevalence of chlamydia trachomatis only at Rectum |
| 0.20 | 0.13 | 0.29 | %, Infection prevalence of chlamydia trachomatis at Oropharynx & Urethra |
| 1.25 | 1.03 | 1.44 | %, Infection prevalence of chlamydia trachomatis at Oropharynx & Rectum |
| 1.90 | 1.72 | 2.22 | %, Infection prevalence of chlamydia trachomatis at Urethra & Rectum |
| 0.15 | 0.09 | 0.26 | %, Infection prevalence of chlamydia trachomatis at Oropharynx & Urethra & Rectum |

**Table S8. 129.** Model 19’ output parameters with 95% confidence intervals

| **Mean** | **95%CI: lower** | **95%CI: upper** | **Parameters** |
| --- | --- | --- | --- |
| 49.24 | 44.76 | 53.00 | %, Consistent condom usage in anal sex in past 12 months |
| 87.91 | 84.11 | 90.20 | %, Condom efficacy in preventing transmission |
| 7.93 | 6.30 | 10.76 | Times, Frequency of kissing in the past 12 months |
| 14.29 | 8.10 | 22.54 | Times, Frequency of oral sex in the past 12 months |
| 34.95 | 12.64 | 53.19 | Times, Frequency of rimming in the past 12 months |
| 23.85 | 14.62 | 31.20 | Times, Frequency of anal sex in the past 12 months |
| 128.77 | 71.37 | 165.03 | weeks, Infection duration of chlamydia trachomatis at throat(asymptomatic infection) |
| 1.51 | 1.31 | 1.65 | weeks, Infection duration at urethral (symptomatic infection) |
| 100.00 | 100.00 | 100.00 | %, proportion of urethral infections that are asymptomatic |
| 100.00 | 100.00 | 100.00 | %, proportion of anal infections that are asymptomatic |
| 29.98 | 23.82 | 35.53 | weeks, Infection duration at urethral (asymptomatic infection) |
| 49.98 | 49.09 | 50.69 | weeks, Infection duration of chlamydia trachomatis at anus (asymptomatic infection) |
| 1.60 | 1.34 | 1.74 | weeks, Infection duration of chlamydia trachomatis at anus (symptomatic infection) |
| 77.20 | 73.50 | 82.57 | %, proportion of MSM received throat swab in the past 12 months |
| 80.26 | 73.73 | 85.10 | %, proportion of MSM received anal swab in the past 12 months |
| 78.72 | 72.40 | 83.58 | %, proportion of MSM received urine test in the past 12 months |
| 30.12 | 27.94 | 31.39 | %, proportion of 'oral sex and anal sex' in the same sex episode |
| 71.25 | 70.56 | 71.69 | %, proportion of 'oral sex and rimming' in the same sex episode |
| 68.84 | 67.76 | 69.74 | %, proportion of saliva use during anal sex |
| 80.00 | 80.00 | 80.00 | %, proportion of receptive oral sex followed by partner's insertive anal sex |
| 80.00 | 80.00 | 80.00 | %, proportion of insertive oral sex followed by partner's insertive rimming |
| 2.01 | 1.77 | 2.14 | %, Infection prevalence of chlamydia trachomatis only at Oropharynx |
| 4.55 | 4.26 | 5.04 | %, Infection prevalence of chlamydia trachomatis only at Urethral |
| 11.75 | 11.20 | 12.44 | %, Infection prevalence of chlamydia trachomatis only at Rectum |
| 0.23 | 0.15 | 0.28 | %, Infection prevalence of chlamydia trachomatis at Oropharynx & Urethra |
| 1.28 | 1.09 | 1.43 | %, Infection prevalence of chlamydia trachomatis at Oropharynx & Rectum |
| 2.01 | 1.76 | 2.19 | %, Infection prevalence of chlamydia trachomatis at Urethra & Rectum |
| 0.16 | 0.09 | 0.26 | %, Infection prevalence of chlamydia trachomatis at Oropharynx & Urethra & Rectum |

**Table S8. 130.** Model 20’ output parameters with 95% confidence intervals

| **Mean** | **95%CI: lower** | **95%CI: upper** | **Parameters** |
| --- | --- | --- | --- |
| 47.95 | 44.89 | 52.16 | %, Consistent condom usage in anal sex in past 12 months |
| 87.48 | 84.46 | 90.12 | %, Condom efficacy in preventing transmission |
| 8.49 | 6.43 | 10.29 | Times, Frequency of kissing in the past 12 months |
| 14.17 | 8.69 | 21.97 | Times, Frequency of oral sex in the past 12 months |
| 33.72 | 15.11 | 50.68 | Times, Frequency of rimming in the past 12 months |
| 25.00 | 15.93 | 31.24 | Times, Frequency of anal sex in the past 12 months |
| 114.80 | 74.31 | 160.79 | weeks, Infection duration of chlamydia trachomatis at throat(asymptomatic infection) |
| 1.50 | 1.37 | 1.61 | weeks, Infection duration at urethral (symptomatic infection) |
| 100.00 | 100.00 | 100.00 | %, proportion of urethral infections that are asymptomatic |
| 100.00 | 100.00 | 100.00 | %, proportion of anal infections that are asymptomatic |
| 31.10 | 24.82 | 35.39 | weeks, Infection duration at urethral (asymptomatic infection) |
| 49.72 | 49.17 | 50.44 | weeks, Infection duration of chlamydia trachomatis at anus (asymptomatic infection) |
| 1.55 | 1.34 | 1.71 | weeks, Infection duration of chlamydia trachomatis at anus (symptomatic infection) |
| 77.10 | 73.49 | 81.97 | %, proportion of MSM received throat swab in the past 12 months |
| 80.64 | 75.41 | 84.70 | %, proportion of MSM received anal swab in the past 12 months |
| 78.45 | 72.67 | 82.66 | %, proportion of MSM received urine test in the past 12 months |
| 29.85 | 28.13 | 31.29 | %, proportion of 'oral sex and anal sex' in the same sex episode |
| 71.04 | 70.63 | 71.64 | %, proportion of 'oral sex and rimming' in the same sex episode |
| 68.58 | 67.88 | 69.56 | %, proportion of saliva use during anal sex |
| 80.00 | 80.00 | 80.00 | %, proportion of receptive oral sex followed by partner's insertive anal sex |
| 80.00 | 80.00 | 80.00 | %, proportion of insertive oral sex followed by partner's insertive rimming |
| 1.94 | 1.79 | 2.11 | %, Infection prevalence of chlamydia trachomatis only at Oropharynx |
| 4.63 | 4.26 | 4.95 | %, Infection prevalence of chlamydia trachomatis only at Urethral |
| 11.82 | 11.25 | 12.39 | %, Infection prevalence of chlamydia trachomatis only at Rectum |
| 0.21 | 0.16 | 0.26 | %, Infection prevalence of chlamydia trachomatis at Oropharynx & Urethra |
| 1.24 | 1.08 | 1.43 | %, Infection prevalence of chlamydia trachomatis at Oropharynx & Rectum |
| 1.93 | 1.79 | 2.17 | %, Infection prevalence of chlamydia trachomatis at Urethra & Rectum |
| 0.17 | 0.10 | 0.25 | %, Infection prevalence of chlamydia trachomatis at Oropharynx & Urethra & Rectum |

Model output parameters (Published validation dataset5): Data from 179 MSM with HIV in the USA

**Table S8. 131.** Model 3’ output parameters with 95% confidence intervals

| **Mean** | **95%CI: lower** | **95%CI: upper** | **Parameters** |
| --- | --- | --- | --- |
| 44.72 | 34.86 | 57.44 | %, Consistent condom usage in anal sex in past 12 months |
| 85.81 | 80.51 | 94.01 | %, Condom efficacy in preventing transmission |
| 6.16 | 0.81 | 12.43 | Times, Frequency of kissing in the past 12 months |
| 14.21 | 1.30 | 27.72 | Times, Frequency of oral sex in the past 12 months |
| 29.43 | 6.87 | 76.83 | Times, Frequency of rimming in the past 12 months |
| 11.17 | 2.57 | 50.14 | Times, Frequency of anal sex in the past 12 months |
| 103.34 | 28.69 | 224.19 | weeks, Infection duration of chlamydia trachomatis at throat(asymptomatic infection) |
| 1.50 | 1.11 | 1.97 | weeks, Infection duration at urethral (symptomatic infection) |
| 84.67 | 80.42 | 88.74 | %, proportion of urethral infections that are asymptomatic |
| 83.16 | 64.12 | 96.40 | %, proportion of anal infections that are asymptomatic |
| 32.33 | 4.80 | 50.09 | weeks, Infection duration at urethral (asymptomatic infection) |
| 50.10 | 48.13 | 51.70 | weeks, Infection duration of chlamydia trachomatis at anus (asymptomatic infection) |
| 1.52 | 1.05 | 1.97 | weeks, Infection duration of chlamydia trachomatis at anus (symptomatic infection) |
| 84.16 | 67.99 | 94.41 | %, proportion of MSM received throat swab in the past 12 months |
| 77.88 | 65.87 | 91.62 | %, proportion of MSM received anal swab in the past 12 months |
| 77.49 | 64.87 | 93.20 | %, proportion of MSM received urine test in the past 12 months |
| 28.26 | 25.09 | 33.61 | %, proportion of 'oral sex and anal sex' in the same sex episode |
| 71.61 | 70.18 | 72.60 | %, proportion of 'oral sex and rimming' in the same sex episode |
| 67.91 | 66.28 | 70.70 | %, proportion of saliva use during anal sex |
| 80.00 | 80.00 | 80.00 | %, proportion of receptive oral sex followed by partner's insertive anal sex |
| 80.00 | 80.00 | 80.00 | %, proportion of insertive oral sex followed by partner's insertive rimming |
| 0.00 | 0.00 | 0.00 | %, Infection prevalence of chlamydia trachomatis only at Oropharynx |
| 2.83 | 0.58 | 4.84 | %, Infection prevalence of chlamydia trachomatis only at Urethral |
| 12.52 | 8.73 | 17.74 | %, Infection prevalence of chlamydia trachomatis only at Rectum |
| 0.00 | 0.00 | 0.00 | %, Infection prevalence of chlamydia trachomatis at Oropharynx & Urethra |
| 0.00 | 0.00 | 0.00 | %, Infection prevalence of chlamydia trachomatis at Oropharynx & Rectum |
| 2.41 | 0.72 | 4.37 | %, Infection prevalence of chlamydia trachomatis at Urethra & Rectum |
| 0.00 | 0.00 | 0.00 | %, Infection prevalence of chlamydia trachomatis at Oropharynx & Urethra & Rectum |

**Table S8. 132.** Model 14’ output parameters with 95% confidence intervals

| **Mean** | **95%CI: lower** | **95%CI: upper** | **Parameters** |
| --- | --- | --- | --- |
| 45.18 | 36.88 | 56.22 | %, Consistent condom usage in anal sex in past 12 months |
| 85.69 | 81.36 | 92.59 | %, Condom efficacy in preventing transmission |
| 8.23 | 0.97 | 12.12 | Times, Frequency of kissing in the past 12 months |
| 15.63 | 3.72 | 27.53 | Times, Frequency of oral sex in the past 12 months |
| 24.59 | 8.37 | 76.23 | Times, Frequency of rimming in the past 12 months |
| 14.26 | 3.90 | 42.74 | Times, Frequency of anal sex in the past 12 months |
| 112.42 | 34.32 | 204.86 | weeks, Infection duration of chlamydia trachomatis at throat(asymptomatic infection) |
| 1.54 | 1.22 | 1.91 | weeks, Infection duration at urethral (symptomatic infection) |
| 85.01 | 81.08 | 88.33 | %, proportion of urethral infections that are asymptomatic |
| 79.92 | 67.42 | 94.72 | %, proportion of anal infections that are asymptomatic |
| 29.06 | 8.25 | 49.68 | weeks, Infection duration at urethral (asymptomatic infection) |
| 49.61 | 48.26 | 51.55 | weeks, Infection duration of chlamydia trachomatis at anus (asymptomatic infection) |
| 1.55 | 1.09 | 1.95 | weeks, Infection duration of chlamydia trachomatis at anus (symptomatic infection) |
| 77.91 | 69.36 | 91.62 | %, proportion of MSM received throat swab in the past 12 months |
| 78.68 | 67.83 | 89.00 | %, proportion of MSM received anal swab in the past 12 months |
| 75.70 | 66.52 | 92.29 | %, proportion of MSM received urine test in the past 12 months |
| 29.07 | 26.03 | 32.89 | %, proportion of 'oral sex and anal sex' in the same sex episode |
| 71.53 | 70.52 | 72.53 | %, proportion of 'oral sex and rimming' in the same sex episode |
| 68.20 | 66.55 | 70.49 | %, proportion of saliva use during anal sex |
| 80.00 | 80.00 | 80.00 | %, proportion of receptive oral sex followed by partner's insertive anal sex |
| 80.00 | 80.00 | 80.00 | %, proportion of insertive oral sex followed by partner's insertive rimming |
| 0.00 | 0.00 | 0.00 | %, Infection prevalence of chlamydia trachomatis only at Oropharynx |
| 3.02 | 1.35 | 4.62 | %, Infection prevalence of chlamydia trachomatis only at Urethral |
| 13.66 | 9.46 | 17.16 | %, Infection prevalence of chlamydia trachomatis only at Rectum |
| 0.00 | 0.00 | 0.00 | %, Infection prevalence of chlamydia trachomatis at Oropharynx & Urethra |
| 0.00 | 0.00 | 0.00 | %, Infection prevalence of chlamydia trachomatis at Oropharynx & Rectum |
| 2.51 | 1.20 | 3.93 | %, Infection prevalence of chlamydia trachomatis at Urethra & Rectum |
| 0.00 | 0.00 | 0.00 | %, Infection prevalence of chlamydia trachomatis at Oropharynx & Urethra & Rectum |

**Table S8. 133.** Model 15’ output parameters with 95% confidence intervals

| **Mean** | **95%CI: lower** | **95%CI: upper** | **Parameters** |
| --- | --- | --- | --- |
| 45.99 | 37.01 | 54.72 | %, Consistent condom usage in anal sex in past 12 months |
| 87.54 | 81.66 | 91.99 | %, Condom efficacy in preventing transmission |
| 5.36 | 1.19 | 11.53 | Times, Frequency of kissing in the past 12 months |
| 18.84 | 10.36 | 26.86 | Times, Frequency of oral sex in the past 12 months |
| 44.30 | 10.93 | 75.92 | Times, Frequency of rimming in the past 12 months |
| 19.71 | 6.17 | 39.15 | Times, Frequency of anal sex in the past 12 months |
| 117.13 | 42.28 | 190.49 | weeks, Infection duration of chlamydia trachomatis at throat(asymptomatic infection) |
| 1.63 | 1.24 | 1.90 | weeks, Infection duration at urethral (symptomatic infection) |
| 84.75 | 81.63 | 87.87 | %, proportion of urethral infections that are asymptomatic |
| 80.45 | 69.81 | 93.54 | %, proportion of anal infections that are asymptomatic |
| 27.01 | 10.55 | 47.03 | weeks, Infection duration at urethral (asymptomatic infection) |
| 50.40 | 48.97 | 51.41 | weeks, Infection duration of chlamydia trachomatis at anus (asymptomatic infection) |
| 1.58 | 1.09 | 1.89 | weeks, Infection duration of chlamydia trachomatis at anus (symptomatic infection) |
| 78.93 | 71.35 | 90.89 | %, proportion of MSM received throat swab in the past 12 months |
| 79.98 | 68.51 | 86.61 | %, proportion of MSM received anal swab in the past 12 months |
| 79.18 | 67.59 | 91.34 | %, proportion of MSM received urine test in the past 12 months |
| 30.13 | 26.21 | 32.73 | %, proportion of 'oral sex and anal sex' in the same sex episode |
| 71.70 | 70.64 | 72.33 | %, proportion of 'oral sex and rimming' in the same sex episode |
| 68.35 | 66.68 | 69.80 | %, proportion of saliva use during anal sex |
| 80.00 | 80.00 | 80.00 | %, proportion of receptive oral sex followed by partner's insertive anal sex |
| 80.00 | 80.00 | 80.00 | %, proportion of insertive oral sex followed by partner's insertive rimming |
| 0.00 | 0.00 | 0.00 | %, Infection prevalence of chlamydia trachomatis only at Oropharynx |
| 3.37 | 1.67 | 4.50 | %, Infection prevalence of chlamydia trachomatis only at Urethral |
| 13.64 | 10.07 | 16.77 | %, Infection prevalence of chlamydia trachomatis only at Rectum |
| 0.00 | 0.00 | 0.00 | %, Infection prevalence of chlamydia trachomatis at Oropharynx & Urethra |
| 0.00 | 0.00 | 0.00 | %, Infection prevalence of chlamydia trachomatis at Oropharynx & Rectum |
| 2.15 | 1.22 | 3.80 | %, Infection prevalence of chlamydia trachomatis at Urethra & Rectum |
| 0.00 | 0.00 | 0.00 | %, Infection prevalence of chlamydia trachomatis at Oropharynx & Urethra & Rectum |

**Table S8. 134.** Model 16’ output parameters with 95% confidence intervals

| **Mean** | **95%CI: lower** | **95%CI: upper** | **Parameters** |
| --- | --- | --- | --- |
| 44.68 | 37.37 | 54.55 | %, Consistent condom usage in anal sex in past 12 months |
| 86.26 | 82.01 | 90.93 | %, Condom efficacy in preventing transmission |
| 6.54 | 2.16 | 11.33 | Times, Frequency of kissing in the past 12 months |
| 16.63 | 10.19 | 25.32 | Times, Frequency of oral sex in the past 12 months |
| 48.28 | 13.90 | 75.97 | Times, Frequency of rimming in the past 12 months |
| 10.55 | 5.90 | 27.19 | Times, Frequency of anal sex in the past 12 months |
| 92.28 | 48.01 | 174.31 | weeks, Infection duration of chlamydia trachomatis at throat(asymptomatic infection) |
| 1.50 | 1.28 | 1.83 | weeks, Infection duration at urethral (symptomatic infection) |
| 84.52 | 82.21 | 87.19 | %, proportion of urethral infections that are asymptomatic |
| 85.23 | 70.47 | 92.13 | %, proportion of anal infections that are asymptomatic |
| 30.64 | 14.58 | 45.27 | weeks, Infection duration at urethral (asymptomatic infection) |
| 50.18 | 48.97 | 51.30 | weeks, Infection duration of chlamydia trachomatis at anus (asymptomatic infection) |
| 1.52 | 1.13 | 1.85 | weeks, Infection duration of chlamydia trachomatis at anus (symptomatic infection) |
| 80.72 | 71.82 | 88.14 | %, proportion of MSM received throat swab in the past 12 months |
| 77.58 | 69.27 | 84.65 | %, proportion of MSM received anal swab in the past 12 months |
| 82.59 | 69.93 | 90.31 | %, proportion of MSM received urine test in the past 12 months |
| 29.90 | 27.08 | 32.06 | %, proportion of 'oral sex and anal sex' in the same sex episode |
| 71.57 | 70.86 | 72.22 | %, proportion of 'oral sex and rimming' in the same sex episode |
| 67.62 | 66.83 | 68.90 | %, proportion of saliva use during anal sex |
| 80.00 | 80.00 | 80.00 | %, proportion of receptive oral sex followed by partner's insertive anal sex |
| 80.00 | 80.00 | 80.00 | %, proportion of insertive oral sex followed by partner's insertive rimming |
| 0.00 | 0.00 | 0.00 | %, Infection prevalence of chlamydia trachomatis only at Oropharynx |
| 3.25 | 1.75 | 4.17 | %, Infection prevalence of chlamydia trachomatis only at Urethral |
| 13.51 | 10.14 | 16.66 | %, Infection prevalence of chlamydia trachomatis only at Rectum |
| 0.00 | 0.00 | 0.00 | %, Infection prevalence of chlamydia trachomatis at Oropharynx & Urethra |
| 0.00 | 0.00 | 0.00 | %, Infection prevalence of chlamydia trachomatis at Oropharynx & Rectum |
| 2.70 | 1.79 | 3.85 | %, Infection prevalence of chlamydia trachomatis at Urethra & Rectum |
| 0.00 | 0.00 | 0.00 | %, Infection prevalence of chlamydia trachomatis at Oropharynx & Urethra & Rectum |

**Table S8. 135.** Model 17’ output parameters with 95% confidence intervals

| **Mean** | **95%CI: lower** | **95%CI: upper** | **Parameters** |
| --- | --- | --- | --- |
| 44.78 | 37.69 | 52.37 | %, Consistent condom usage in anal sex in past 12 months |
| 86.20 | 82.36 | 90.81 | %, Condom efficacy in preventing transmission |
| 6.16 | 2.47 | 11.05 | Times, Frequency of kissing in the past 12 months |
| 19.91 | 11.53 | 25.41 | Times, Frequency of oral sex in the past 12 months |
| 38.23 | 17.27 | 69.14 | Times, Frequency of rimming in the past 12 months |
| 13.36 | 7.12 | 23.75 | Times, Frequency of anal sex in the past 12 months |
| 114.16 | 54.28 | 169.45 | weeks, Infection duration of chlamydia trachomatis at throat(asymptomatic infection) |
| 1.59 | 1.29 | 1.82 | weeks, Infection duration at urethral (symptomatic infection) |
| 84.85 | 82.32 | 87.06 | %, proportion of urethral infections that are asymptomatic |
| 82.27 | 71.20 | 90.14 | %, proportion of anal infections that are asymptomatic |
| 24.85 | 15.90 | 44.17 | weeks, Infection duration at urethral (asymptomatic infection) |
| 50.31 | 49.08 | 51.16 | weeks, Infection duration of chlamydia trachomatis at anus (asymptomatic infection) |
| 1.50 | 1.12 | 1.82 | weeks, Infection duration of chlamydia trachomatis at anus (symptomatic infection) |
| 82.01 | 73.27 | 87.77 | %, proportion of MSM received throat swab in the past 12 months |
| 77.98 | 69.53 | 84.26 | %, proportion of MSM received anal swab in the past 12 months |
| 78.38 | 70.82 | 89.72 | %, proportion of MSM received urine test in the past 12 months |
| 28.64 | 27.24 | 31.68 | %, proportion of 'oral sex and anal sex' in the same sex episode |
| 71.53 | 70.93 | 72.14 | %, proportion of 'oral sex and rimming' in the same sex episode |
| 68.00 | 66.96 | 68.81 | %, proportion of saliva use during anal sex |
| 80.00 | 80.00 | 80.00 | %, proportion of receptive oral sex followed by partner's insertive anal sex |
| 80.00 | 80.00 | 80.00 | %, proportion of insertive oral sex followed by partner's insertive rimming |
| 0.00 | 0.00 | 0.00 | %, Infection prevalence of chlamydia trachomatis only at Oropharynx |
| 2.90 | 1.79 | 3.88 | %, Infection prevalence of chlamydia trachomatis only at Urethral |
| 13.99 | 10.87 | 16.23 | %, Infection prevalence of chlamydia trachomatis only at Rectum |
| 0.00 | 0.00 | 0.00 | %, Infection prevalence of chlamydia trachomatis at Oropharynx & Urethra |
| 0.00 | 0.00 | 0.00 | %, Infection prevalence of chlamydia trachomatis at Oropharynx & Rectum |
| 2.72 | 1.99 | 3.76 | %, Infection prevalence of chlamydia trachomatis at Urethra & Rectum |
| 0.00 | 0.00 | 0.00 | %, Infection prevalence of chlamydia trachomatis at Oropharynx & Urethra & Rectum |

**Table S8. 136.** Model 18’ output parameters with 95% confidence intervals

| **Mean** | **95%CI: lower** | **95%CI: upper** | **Parameters** |
| --- | --- | --- | --- |
| 42.43 | 38.03 | 48.63 | %, Consistent condom usage in anal sex in past 12 months |
| 86.11 | 83.01 | 90.32 | %, Condom efficacy in preventing transmission |
| 5.36 | 2.79 | 10.38 | Times, Frequency of kissing in the past 12 months |
| 19.29 | 12.28 | 25.15 | Times, Frequency of oral sex in the past 12 months |
| 48.10 | 21.50 | 68.72 | Times, Frequency of rimming in the past 12 months |
| 14.41 | 7.89 | 20.80 | Times, Frequency of anal sex in the past 12 months |
| 90.02 | 55.20 | 149.84 | weeks, Infection duration of chlamydia trachomatis at throat(asymptomatic infection) |
| 1.53 | 1.36 | 1.77 | weeks, Infection duration at urethral (symptomatic infection) |
| 84.48 | 82.33 | 86.82 | %, proportion of urethral infections that are asymptomatic |
| 83.19 | 71.29 | 88.76 | %, proportion of anal infections that are asymptomatic |
| 32.06 | 18.78 | 43.16 | weeks, Infection duration at urethral (asymptomatic infection) |
| 50.03 | 49.26 | 51.14 | weeks, Infection duration of chlamydia trachomatis at anus (asymptomatic infection) |
| 1.47 | 1.14 | 1.79 | weeks, Infection duration of chlamydia trachomatis at anus (symptomatic infection) |
| 80.95 | 73.64 | 87.51 | %, proportion of MSM received throat swab in the past 12 months |
| 77.60 | 71.26 | 84.32 | %, proportion of MSM received anal swab in the past 12 months |
| 80.70 | 71.79 | 90.00 | %, proportion of MSM received urine test in the past 12 months |
| 29.52 | 27.41 | 31.11 | %, proportion of 'oral sex and anal sex' in the same sex episode |
| 71.50 | 70.96 | 72.08 | %, proportion of 'oral sex and rimming' in the same sex episode |
| 67.64 | 67.10 | 68.55 | %, proportion of saliva use during anal sex |
| 80.00 | 80.00 | 80.00 | %, proportion of receptive oral sex followed by partner's insertive anal sex |
| 80.00 | 80.00 | 80.00 | %, proportion of insertive oral sex followed by partner's insertive rimming |
| 0.00 | 0.00 | 0.00 | %, Infection prevalence of chlamydia trachomatis only at Oropharynx |
| 2.81 | 1.82 | 3.76 | %, Infection prevalence of chlamydia trachomatis only at Urethral |
| 12.52 | 11.01 | 16.08 | %, Infection prevalence of chlamydia trachomatis only at Rectum |
| 0.00 | 0.00 | 0.00 | %, Infection prevalence of chlamydia trachomatis at Oropharynx & Urethra |
| 0.00 | 0.00 | 0.00 | %, Infection prevalence of chlamydia trachomatis at Oropharynx & Rectum |
| 2.70 | 2.05 | 3.52 | %, Infection prevalence of chlamydia trachomatis at Urethra & Rectum |
| 0.00 | 0.00 | 0.00 | %, Infection prevalence of chlamydia trachomatis at Oropharynx & Urethra & Rectum |

**Table S8. 137.** Model 19’ output parameters with 95% confidence intervals

| **Mean** | **95%CI: lower** | **95%CI: upper** | **Parameters** |
| --- | --- | --- | --- |
| 43.31 | 38.42 | 47.43 | %, Consistent condom usage in anal sex in past 12 months |
| 86.31 | 83.27 | 89.95 | %, Condom efficacy in preventing transmission |
| 5.99 | 2.99 | 10.30 | Times, Frequency of kissing in the past 12 months |
| 19.06 | 14.39 | 24.65 | Times, Frequency of oral sex in the past 12 months |
| 42.50 | 24.69 | 68.15 | Times, Frequency of rimming in the past 12 months |
| 12.12 | 8.51 | 19.53 | Times, Frequency of anal sex in the past 12 months |
| 80.49 | 56.76 | 128.73 | weeks, Infection duration of chlamydia trachomatis at throat(asymptomatic infection) |
| 1.54 | 1.37 | 1.73 | weeks, Infection duration at urethral (symptomatic infection) |
| 84.59 | 82.60 | 86.81 | %, proportion of urethral infections that are asymptomatic |
| 82.84 | 72.03 | 88.12 | %, proportion of anal infections that are asymptomatic |
| 30.54 | 20.94 | 41.95 | weeks, Infection duration at urethral (asymptomatic infection) |
| 50.47 | 49.34 | 51.06 | weeks, Infection duration of chlamydia trachomatis at anus (asymptomatic infection) |
| 1.46 | 1.15 | 1.69 | weeks, Infection duration of chlamydia trachomatis at anus (symptomatic infection) |
| 82.15 | 74.33 | 87.61 | %, proportion of MSM received throat swab in the past 12 months |
| 78.31 | 73.11 | 83.63 | %, proportion of MSM received anal swab in the past 12 months |
| 83.47 | 73.06 | 88.17 | %, proportion of MSM received urine test in the past 12 months |
| 29.29 | 27.51 | 30.94 | %, proportion of 'oral sex and anal sex' in the same sex episode |
| 71.62 | 71.06 | 72.02 | %, proportion of 'oral sex and rimming' in the same sex episode |
| 67.77 | 67.16 | 68.47 | %, proportion of saliva use during anal sex |
| 80.00 | 80.00 | 80.00 | %, proportion of receptive oral sex followed by partner's insertive anal sex |
| 80.00 | 80.00 | 80.00 | %, proportion of insertive oral sex followed by partner's insertive rimming |
| 0.00 | 0.00 | 0.00 | %, Infection prevalence of chlamydia trachomatis only at Oropharynx |
| 2.69 | 1.90 | 3.48 | %, Infection prevalence of chlamydia trachomatis only at Urethral |
| 13.50 | 11.37 | 15.82 | %, Infection prevalence of chlamydia trachomatis only at Rectum |
| 0.00 | 0.00 | 0.00 | %, Infection prevalence of chlamydia trachomatis at Oropharynx & Urethra |
| 0.00 | 0.00 | 0.00 | %, Infection prevalence of chlamydia trachomatis at Oropharynx & Rectum |
| 2.66 | 2.10 | 3.49 | %, Infection prevalence of chlamydia trachomatis at Urethra & Rectum |
| 0.00 | 0.00 | 0.00 | %, Infection prevalence of chlamydia trachomatis at Oropharynx & Urethra & Rectum |

**Table S8. 138.** Model 20’ output parameters with 95% confidence intervals

| **Mean** | **95%CI: lower** | **95%CI: upper** | **Parameters** |
| --- | --- | --- | --- |
| 44.20 | 39.39 | 47.38 | %, Consistent condom usage in anal sex in past 12 months |
| 86.78 | 83.99 | 89.46 | %, Condom efficacy in preventing transmission |
| 6.56 | 3.04 | 9.87 | Times, Frequency of kissing in the past 12 months |
| 19.81 | 15.10 | 23.77 | Times, Frequency of oral sex in the past 12 months |
| 37.90 | 25.21 | 59.18 | Times, Frequency of rimming in the past 12 months |
| 14.51 | 9.18 | 18.61 | Times, Frequency of anal sex in the past 12 months |
| 80.36 | 57.88 | 122.76 | weeks, Infection duration of chlamydia trachomatis at throat(asymptomatic infection) |
| 1.61 | 1.39 | 1.70 | weeks, Infection duration at urethral (symptomatic infection) |
| 84.74 | 82.68 | 86.48 | %, proportion of urethral infections that are asymptomatic |
| 80.84 | 72.13 | 86.81 | %, proportion of anal infections that are asymptomatic |
| 27.03 | 20.85 | 37.03 | weeks, Infection duration at urethral (asymptomatic infection) |
| 50.30 | 49.48 | 51.04 | weeks, Infection duration of chlamydia trachomatis at anus (asymptomatic infection) |
| 1.44 | 1.22 | 1.67 | weeks, Infection duration of chlamydia trachomatis at anus (symptomatic infection) |
| 80.84 | 74.47 | 87.18 | %, proportion of MSM received throat swab in the past 12 months |
| 78.62 | 74.32 | 83.26 | %, proportion of MSM received anal swab in the past 12 months |
| 79.13 | 72.73 | 86.96 | %, proportion of MSM received urine test in the past 12 months |
| 29.02 | 27.89 | 30.53 | %, proportion of 'oral sex and anal sex' in the same sex episode |
| 71.61 | 71.11 | 71.98 | %, proportion of 'oral sex and rimming' in the same sex episode |
| 67.88 | 67.27 | 68.42 | %, proportion of saliva use during anal sex |
| 80.00 | 80.00 | 80.00 | %, proportion of receptive oral sex followed by partner's insertive anal sex |
| 80.00 | 80.00 | 80.00 | %, proportion of insertive oral sex followed by partner's insertive rimming |
| 0.00 | 0.00 | 0.00 | %, Infection prevalence of chlamydia trachomatis only at Oropharynx |
| 2.49 | 1.98 | 3.45 | %, Infection prevalence of chlamydia trachomatis only at Urethral |
| 13.07 | 11.39 | 15.43 | %, Infection prevalence of chlamydia trachomatis only at Rectum |
| 0.00 | 0.00 | 0.00 | %, Infection prevalence of chlamydia trachomatis at Oropharynx & Urethra |
| 0.00 | 0.00 | 0.00 | %, Infection prevalence of chlamydia trachomatis at Oropharynx & Rectum |
| 2.69 | 2.17 | 3.39 | %, Infection prevalence of chlamydia trachomatis at Urethra & Rectum |
| 0.00 | 0.00 | 0.00 | %, Infection prevalence of chlamydia trachomatis at Oropharynx & Urethra & Rectum |

Sensitivity analysis of model 2, 7-13 : 4888 MSM attending Melbourne Sexual Health Centre

Model output parameters of sensitivity analysis: half-length of asymptomatic urethral and anal duration

**Table S8. 139.** Model 2’ output parameters with 95% confidence intervals

| **Mean** | **95%CI: lower** | **95%CI: upper** | **Parameters** |
| --- | --- | --- | --- |
| 45.36 | 34.95 | 57.20 | %, Consistent condom usage in anal sex in past 12 months |
| 87.25 | 81.12 | 94.37 | %, Condom efficacy in preventing transmission |
| - | - | - | Times, Frequency of kissing in the past 12 months |
| 14.66 | 2.77 | 26.25 | Times, Frequency of oral sex in the past 12 months |
| 27.09 | 4.51 | 59.51 | Times, Frequency of rimming in the past 12 months |
| 14.37 | 0.68 | 48.66 | Times, Frequency of anal sex in the past 12 months |
| 74.55 | 35.43 | 117.24 | weeks, Infection duration of chlamydia trachomatis at throat(asymptomatic infection) |
| 1.44 | 1.09 | 1.95 | weeks, Infection duration at urethral (symptomatic infection) |
| 84.27 | 81.07 | 88.14 | %, proportion of urethral infections that are asymptomatic |
| 85.23 | 62.16 | 96.77 | %, proportion of anal infections that are asymptomatic |
| 11.98 | 3.09 | 25.20 | weeks, Infection duration at urethral (asymptomatic infection) |
| 25.10 | 24.13 | 25.89 | weeks, Infection duration of chlamydia trachomatis at anus (asymptomatic infection) |
| 1.44 | 1.02 | 1.97 | weeks, Infection duration of chlamydia trachomatis at anus (symptomatic infection) |
| 80.52 | 66.27 | 93.93 | %, proportion of MSM received throat swab in the past 12 months |
| 79.86 | 66.31 | 91.60 | %, proportion of MSM received anal swab in the past 12 months |
| 78.80 | 65.77 | 92.05 | %, proportion of MSM received urine test in the past 12 months |
| 29.60 | 25.38 | 32.94 | %, proportion of 'oral sex and anal sex' in the same sex episode |
| 71.04 | 70.00 | 72.74 | %, proportion of 'oral sex and rimming' in the same sex episode |
| 68.95 | 66.58 | 70.43 | %, proportion of saliva use during anal sex |
| 80.00 | 80.00 | 80.00 | %, proportion of receptive oral sex followed by partner's insertive anal sex |
| 80.00 | 80.00 | 80.00 | %, proportion of insertive oral sex followed by partner's insertive rimming |
| 0.79 | 0.60 | 1.04 | %, Infection prevalence of chlamydia trachomatis only at Oropharynx |
| 1.93 | 1.55 | 2.19 | %, Infection prevalence of chlamydia trachomatis only at Urethral |
| 7.56 | 6.85 | 8.25 | %, Infection prevalence of chlamydia trachomatis only at Rectum |
| 0.00 | 0.00 | 0.00 | %, Infection prevalence of chlamydia trachomatis at Oropharynx & Urethra |
| 1.23 | 1.00 | 1.47 | %, Infection prevalence of chlamydia trachomatis at Oropharynx & Rectum |
| 1.11 | 0.93 | 1.54 | %, Infection prevalence of chlamydia trachomatis at Urethra & Rectum |
| 0.22 | 0.08 | 0.32 | %, Infection prevalence of chlamydia trachomatis at Oropharynx & Urethra & Rectum |

**Table S8. 140.** Model 7’ output parameters with 95% confidence intervals

| **Mean** | **95%CI: lower** | **95%CI: upper** | **Parameters** |
| --- | --- | --- | --- |
| 41.94 | 34.79 | 54.38 | %, Consistent condom usage in anal sex in past 12 months |
| 86.89 | 82.05 | 93.01 | %, Condom efficacy in preventing transmission |
| - | - | - | Times, Frequency of kissing in the past 12 months |
| 15.10 | 6.55 | 24.42 | Times, Frequency of oral sex in the past 12 months |
| 35.32 | 12.42 | 57.03 | Times, Frequency of rimming in the past 12 months |
| 6.08 | 1.47 | 12.93 | Times, Frequency of anal sex in the past 12 months |
| 80.17 | 53.75 | 115.21 | weeks, Infection duration of chlamydia trachomatis at throat(asymptomatic infection) |
| 1.69 | 1.24 | 1.92 | weeks, Infection duration at urethral (symptomatic infection) |
| 83.92 | 81.60 | 87.23 | %, proportion of urethral infections that are asymptomatic |
| 83.35 | 65.97 | 96.32 | %, proportion of anal infections that are asymptomatic |
| 12.79 | 5.06 | 22.91 | weeks, Infection duration at urethral (asymptomatic infection) |
| 25.09 | 24.42 | 25.74 | weeks, Infection duration of chlamydia trachomatis at anus (asymptomatic infection) |
| 1.54 | 1.07 | 1.91 | weeks, Infection duration of chlamydia trachomatis at anus (symptomatic infection) |
| 80.36 | 69.58 | 92.17 | %, proportion of MSM received throat swab in the past 12 months |
| 80.06 | 69.21 | 89.67 | %, proportion of MSM received anal swab in the past 12 months |
| 81.16 | 68.70 | 87.24 | %, proportion of MSM received urine test in the past 12 months |
| 28.78 | 25.72 | 30.79 | %, proportion of 'oral sex and anal sex' in the same sex episode |
| 71.18 | 70.28 | 72.59 | %, proportion of 'oral sex and rimming' in the same sex episode |
| 68.51 | 66.98 | 70.12 | %, proportion of saliva use during anal sex |
| 80.00 | 80.00 | 80.00 | %, proportion of receptive oral sex followed by partner's insertive anal sex |
| 80.00 | 80.00 | 80.00 | %, proportion of insertive oral sex followed by partner's insertive rimming |
| 0.79 | 0.65 | 1.01 | %, Infection prevalence of chlamydia trachomatis only at Oropharynx |
| 1.88 | 1.63 | 2.14 | %, Infection prevalence of chlamydia trachomatis only at Urethral |
| 7.48 | 6.93 | 8.20 | %, Infection prevalence of chlamydia trachomatis only at Rectum |
| 0.00 | 0.00 | 0.00 | %, Infection prevalence of chlamydia trachomatis at Oropharynx & Urethra |
| 1.24 | 1.04 | 1.47 | %, Infection prevalence of chlamydia trachomatis at Oropharynx & Rectum |
| 1.20 | 0.98 | 1.47 | %, Infection prevalence of chlamydia trachomatis at Urethra & Rectum |
| 0.18 | 0.09 | 0.31 | %, Infection prevalence of chlamydia trachomatis at Oropharynx & Urethra & Rectum |

**Table S8. 141.** Model 8’ output parameters with 95% confidence intervals

| **Mean** | **95%CI: lower** | **95%CI: upper** | **Parameters** |
| --- | --- | --- | --- |
| 46.72 | 38.34 | 53.77 | %, Consistent condom usage in anal sex in past 12 months |
| 87.85 | 82.44 | 93.11 | %, Condom efficacy in preventing transmission |
| - | - | - | Times, Frequency of kissing in the past 12 months |
| 16.26 | 9.42 | 23.59 | Times, Frequency of oral sex in the past 12 months |
| 34.85 | 12.33 | 55.71 | Times, Frequency of rimming in the past 12 months |
| 6.66 | 1.92 | 11.64 | Times, Frequency of anal sex in the past 12 months |
| 82.35 | 55.28 | 113.67 | weeks, Infection duration of chlamydia trachomatis at throat(asymptomatic infection) |
| 1.57 | 1.30 | 1.88 | weeks, Infection duration at urethral (symptomatic infection) |
| 84.59 | 81.72 | 86.99 | %, proportion of urethral infections that are asymptomatic |
| 80.92 | 68.03 | 96.21 | %, proportion of anal infections that are asymptomatic |
| 9.86 | 5.75 | 20.47 | weeks, Infection duration at urethral (asymptomatic infection) |
| 25.14 | 24.52 | 25.74 | weeks, Infection duration of chlamydia trachomatis at anus (asymptomatic infection) |
| 1.47 | 1.08 | 1.88 | weeks, Infection duration of chlamydia trachomatis at anus (symptomatic infection) |
| 82.85 | 72.84 | 91.37 | %, proportion of MSM received throat swab in the past 12 months |
| 81.46 | 69.56 | 89.32 | %, proportion of MSM received anal swab in the past 12 months |
| 77.97 | 69.75 | 85.85 | %, proportion of MSM received urine test in the past 12 months |
| 28.29 | 26.04 | 30.58 | %, proportion of 'oral sex and anal sex' in the same sex episode |
| 71.29 | 70.47 | 72.44 | %, proportion of 'oral sex and rimming' in the same sex episode |
| 68.52 | 67.05 | 69.82 | %, proportion of saliva use during anal sex |
| 80.00 | 80.00 | 80.00 | %, proportion of receptive oral sex followed by partner's insertive anal sex |
| 80.00 | 80.00 | 80.00 | %, proportion of insertive oral sex followed by partner's insertive rimming |
| 0.84 | 0.65 | 1.01 | %, Infection prevalence of chlamydia trachomatis only at Oropharynx |
| 1.99 | 1.68 | 2.13 | %, Infection prevalence of chlamydia trachomatis only at Urethral |
| 7.56 | 7.00 | 8.17 | %, Infection prevalence of chlamydia trachomatis only at Rectum |
| 0.00 | 0.00 | 0.00 | %, Infection prevalence of chlamydia trachomatis at Oropharynx & Urethra |
| 1.22 | 1.04 | 1.46 | %, Infection prevalence of chlamydia trachomatis at Oropharynx & Rectum |
| 1.20 | 0.99 | 1.46 | %, Infection prevalence of chlamydia trachomatis at Urethra & Rectum |
| 0.20 | 0.10 | 0.30 | %, Infection prevalence of chlamydia trachomatis at Oropharynx & Urethra & Rectum |

**Table S8. 142.** Model 9’ output parameters with 95% confidence intervals

| **Mean** | **95%CI: lower** | **95%CI: upper** | **Parameters** |
| --- | --- | --- | --- |
| 45.85 | 40.02 | 52.52 | %, Consistent condom usage in anal sex in past 12 months |
| 90.23 | 84.78 | 92.76 | %, Condom efficacy in preventing transmission |
| - | - | - | Times, Frequency of kissing in the past 12 months |
| 14.67 | 11.12 | 20.80 | Times, Frequency of oral sex in the past 12 months |
| 27.57 | 12.61 | 53.20 | Times, Frequency of rimming in the past 12 months |
| 7.02 | 2.92 | 9.91 | Times, Frequency of anal sex in the past 12 months |
| 81.49 | 59.91 | 107.05 | weeks, Infection duration of chlamydia trachomatis at throat(asymptomatic infection) |
| 1.63 | 1.39 | 1.84 | weeks, Infection duration at urethral (symptomatic infection) |
| 84.24 | 82.15 | 86.83 | %, proportion of urethral infections that are asymptomatic |
| 81.46 | 69.67 | 92.62 | %, proportion of anal infections that are asymptomatic |
| 12.99 | 6.36 | 18.96 | weeks, Infection duration at urethral (asymptomatic infection) |
| 25.17 | 24.67 | 25.64 | weeks, Infection duration of chlamydia trachomatis at anus (asymptomatic infection) |
| 1.46 | 1.13 | 1.85 | weeks, Infection duration of chlamydia trachomatis at anus (symptomatic infection) |
| 82.94 | 74.77 | 90.19 | %, proportion of MSM received throat swab in the past 12 months |
| 79.02 | 71.28 | 88.31 | %, proportion of MSM received anal swab in the past 12 months |
| 80.06 | 71.37 | 85.21 | %, proportion of MSM received urine test in the past 12 months |
| 28.57 | 26.37 | 30.60 | %, proportion of 'oral sex and anal sex' in the same sex episode |
| 71.65 | 70.82 | 72.27 | %, proportion of 'oral sex and rimming' in the same sex episode |
| 68.59 | 67.39 | 69.56 | %, proportion of saliva use during anal sex |
| 80.00 | 80.00 | 80.00 | %, proportion of receptive oral sex followed by partner's insertive anal sex |
| 80.00 | 80.00 | 80.00 | %, proportion of insertive oral sex followed by partner's insertive rimming |
| 0.84 | 0.68 | 0.99 | %, Infection prevalence of chlamydia trachomatis only at Oropharynx |
| 1.90 | 1.68 | 2.08 | %, Infection prevalence of chlamydia trachomatis only at Urethral |
| 7.48 | 7.14 | 7.87 | %, Infection prevalence of chlamydia trachomatis only at Rectum |
| 0.00 | 0.00 | 0.00 | %, Infection prevalence of chlamydia trachomatis at Oropharynx & Urethra |
| 1.16 | 1.06 | 1.43 | %, Infection prevalence of chlamydia trachomatis at Oropharynx & Rectum |
| 1.20 | 1.01 | 1.38 | %, Infection prevalence of chlamydia trachomatis at Urethra & Rectum |
| 0.20 | 0.11 | 0.29 | %, Infection prevalence of chlamydia trachomatis at Oropharynx & Urethra & Rectum |

**Table S8. 143.** Model 10’ output parameters with 95% confidence intervals

| **Mean** | **95%CI: lower** | **95%CI: upper** | **Parameters** |
| --- | --- | --- | --- |
| 47.68 | 41.40 | 51.64 | %, Consistent condom usage in anal sex in past 12 months |
| 88.06 | 85.31 | 90.97 | %, Condom efficacy in preventing transmission |
| - | - | - | Times, Frequency of kissing in the past 12 months |
| 15.83 | 12.10 | 19.46 | Times, Frequency of oral sex in the past 12 months |
| 33.61 | 14.21 | 51.37 | Times, Frequency of rimming in the past 12 months |
| 7.63 | 4.10 | 9.87 | Times, Frequency of anal sex in the past 12 months |
| 82.20 | 64.24 | 102.12 | weeks, Infection duration of chlamydia trachomatis at throat(asymptomatic infection) |
| 1.59 | 1.40 | 1.80 | weeks, Infection duration at urethral (symptomatic infection) |
| 83.95 | 82.32 | 86.52 | %, proportion of urethral infections that are asymptomatic |
| 77.34 | 69.42 | 91.76 | %, proportion of anal infections that are asymptomatic |
| 12.55 | 7.37 | 17.34 | weeks, Infection duration at urethral (asymptomatic infection) |
| 25.21 | 24.85 | 25.50 | weeks, Infection duration of chlamydia trachomatis at anus (asymptomatic infection) |
| 1.59 | 1.19 | 1.78 | weeks, Infection duration of chlamydia trachomatis at anus (symptomatic infection) |
| 81.80 | 75.36 | 89.80 | %, proportion of MSM received throat swab in the past 12 months |
| 81.95 | 72.76 | 87.10 | %, proportion of MSM received anal swab in the past 12 months |
| 78.77 | 73.69 | 84.48 | %, proportion of MSM received urine test in the past 12 months |
| 28.73 | 26.53 | 30.43 | %, proportion of 'oral sex and anal sex' in the same sex episode |
| 71.41 | 70.90 | 72.13 | %, proportion of 'oral sex and rimming' in the same sex episode |
| 68.40 | 67.47 | 69.33 | %, proportion of saliva use during anal sex |
| 80.00 | 80.00 | 80.00 | %, proportion of receptive oral sex followed by partner's insertive anal sex |
| 80.00 | 80.00 | 80.00 | %, proportion of insertive oral sex followed by partner's insertive rimming |
| 0.86 | 0.70 | 0.96 | %, Infection prevalence of chlamydia trachomatis only at Oropharynx |
| 1.86 | 1.71 | 2.05 | %, Infection prevalence of chlamydia trachomatis only at Urethral |
| 7.47 | 7.21 | 7.79 | %, Infection prevalence of chlamydia trachomatis only at Rectum |
| 0.00 | 0.00 | 0.00 | %, Infection prevalence of chlamydia trachomatis at Oropharynx & Urethra |
| 1.27 | 1.10 | 1.42 | %, Infection prevalence of chlamydia trachomatis at Oropharynx & Rectum |
| 1.13 | 1.03 | 1.36 | %, Infection prevalence of chlamydia trachomatis at Urethra & Rectum |
| 0.19 | 0.12 | 0.26 | %, Infection prevalence of chlamydia trachomatis at Oropharynx & Urethra & Rectum |

**Table S8. 144.** Model 11’ output parameters with 95% confidence intervals

| **Mean** | **95%CI: lower** | **95%CI: upper** | **Parameters** |
| --- | --- | --- | --- |
| 46.40 | 42.50 | 50.54 | %, Consistent condom usage in anal sex in past 12 months |
| 87.88 | 86.12 | 90.47 | %, Condom efficacy in preventing transmission |
| - | - | - | Times, Frequency of kissing in the past 12 months |
| 15.78 | 12.82 | 18.90 | Times, Frequency of oral sex in the past 12 months |
| 32.37 | 18.29 | 49.36 | Times, Frequency of rimming in the past 12 months |
| 6.47 | 4.44 | 9.19 | Times, Frequency of anal sex in the past 12 months |
| 91.04 | 82.24 | 100.43 | weeks, Infection duration of chlamydia trachomatis at throat(asymptomatic infection) |
| 1.56 | 1.42 | 1.74 | weeks, Infection duration at urethral (symptomatic infection) |
| 84.33 | 82.79 | 86.03 | %, proportion of urethral infections that are asymptomatic |
| 80.28 | 70.42 | 91.53 | %, proportion of anal infections that are asymptomatic |
| 11.97 | 8.09 | 14.87 | weeks, Infection duration at urethral (asymptomatic infection) |
| 25.17 | 24.93 | 25.41 | weeks, Infection duration of chlamydia trachomatis at anus (asymptomatic infection) |
| 1.42 | 1.30 | 1.72 | weeks, Infection duration of chlamydia trachomatis at anus (symptomatic infection) |
| 82.13 | 76.57 | 88.01 | %, proportion of MSM received throat swab in the past 12 months |
| 81.04 | 74.01 | 86.37 | %, proportion of MSM received anal swab in the past 12 months |
| 78.77 | 75.11 | 83.41 | %, proportion of MSM received urine test in the past 12 months |
| 28.95 | 27.27 | 30.41 | %, proportion of 'oral sex and anal sex' in the same sex episode |
| 71.46 | 71.01 | 71.96 | %, proportion of 'oral sex and rimming' in the same sex episode |
| 68.35 | 67.63 | 69.15 | %, proportion of saliva use during anal sex |
| 80.00 | 80.00 | 80.00 | %, proportion of receptive oral sex followed by partner's insertive anal sex |
| 80.00 | 80.00 | 80.00 | %, proportion of insertive oral sex followed by partner's insertive rimming |
| 0.84 | 0.71 | 0.94 | %, Infection prevalence of chlamydia trachomatis only at Oropharynx |
| 1.89 | 1.75 | 2.03 | %, Infection prevalence of chlamydia trachomatis only at Urethral |
| 7.58 | 7.28 | 7.73 | %, Infection prevalence of chlamydia trachomatis only at Rectum |
| 0.00 | 0.00 | 0.00 | %, Infection prevalence of chlamydia trachomatis at Oropharynx & Urethra |
| 1.26 | 1.15 | 1.35 | %, Infection prevalence of chlamydia trachomatis at Oropharynx & Rectum |
| 1.18 | 1.05 | 1.33 | %, Infection prevalence of chlamydia trachomatis at Urethra & Rectum |
| 0.19 | 0.14 | 0.25 | %, Infection prevalence of chlamydia trachomatis at Oropharynx & Urethra & Rectum |

**Table S8. 145**. Model 12’ output parameters with 95% confidence intervals

| **Mean** | **95%CI: lower** | **95%CI: upper** | **Parameters** |
| --- | --- | --- | --- |
| 47.20 | 43.17 | 49.96 | %, Consistent condom usage in anal sex in past 12 months |
| 88.35 | 86.52 | 89.85 | %, Condom efficacy in preventing transmission |
| - | - | - | Times, Frequency of kissing in the past 12 months |
| 15.92 | 13.35 | 18.14 | Times, Frequency of oral sex in the past 12 months |
| 32.62 | 19.71 | 42.04 | Times, Frequency of rimming in the past 12 months |
| 6.98 | 4.86 | 8.71 | Times, Frequency of anal sex in the past 12 months |
| 90.81 | 83.31 | 99.59 | weeks, Infection duration of chlamydia trachomatis at throat(asymptomatic infection) |
| 1.56 | 1.48 | 1.69 | weeks, Infection duration at urethral (symptomatic infection) |
| 84.41 | 82.92 | 85.75 | %, proportion of urethral infections that are asymptomatic |
| 82.98 | 73.83 | 90.96 | %, proportion of anal infections that are asymptomatic |
| 11.67 | 8.93 | 14.28 | weeks, Infection duration at urethral (asymptomatic infection) |
| 25.07 | 24.96 | 25.30 | weeks, Infection duration of chlamydia trachomatis at anus (asymptomatic infection) |
| 1.51 | 1.35 | 1.69 | weeks, Infection duration of chlamydia trachomatis at anus (symptomatic infection) |
| 82.69 | 77.08 | 87.42 | %, proportion of MSM received throat swab in the past 12 months |
| 79.02 | 74.34 | 84.47 | %, proportion of MSM received anal swab in the past 12 months |
| 79.99 | 75.75 | 82.82 | %, proportion of MSM received urine test in the past 12 months |
| 28.53 | 27.39 | 30.31 | %, proportion of 'oral sex and anal sex' in the same sex episode |
| 71.51 | 71.06 | 71.87 | %, proportion of 'oral sex and rimming' in the same sex episode |
| 68.65 | 67.83 | 68.99 | %, proportion of saliva use during anal sex |
| 80.00 | 80.00 | 80.00 | %, proportion of receptive oral sex followed by partner's insertive anal sex |
| 80.00 | 80.00 | 80.00 | %, proportion of insertive oral sex followed by partner's insertive rimming |
| 0.84 | 0.73 | 0.92 | %, Infection prevalence of chlamydia trachomatis only at Oropharynx |
| 1.88 | 1.75 | 1.99 | %, Infection prevalence of chlamydia trachomatis only at Urethral |
| 7.49 | 7.32 | 7.72 | %, Infection prevalence of chlamydia trachomatis only at Rectum |
| 0.00 | 0.00 | 0.00 | %, Infection prevalence of chlamydia trachomatis at Oropharynx & Urethra |
| 1.24 | 1.16 | 1.34 | %, Infection prevalence of chlamydia trachomatis at Oropharynx & Rectum |
| 1.20 | 1.10 | 1.32 | %, Infection prevalence of chlamydia trachomatis at Urethra & Rectum |
| 0.20 | 0.14 | 0.25 | %, Infection prevalence of chlamydia trachomatis at Oropharynx & Urethra & Rectum |

**Table S8. 146.** Model 13’ output parameters with 95% confidence intervals

| **Mean** | **95%CI: lower** | **95%CI: upper** | **Parameters** |
| --- | --- | --- | --- |
| 46.26 | 43.08 | 49.80 | %, Consistent condom usage in anal sex in past 12 months |
| 88.46 | 86.69 | 89.70 | %, Condom efficacy in preventing transmission |
| - | - | - | Times, Frequency of kissing in the past 12 months |
| 15.38 | 13.45 | 17.91 | Times, Frequency of oral sex in the past 12 months |
| 29.22 | 20.19 | 38.79 | Times, Frequency of rimming in the past 12 months |
| 7.51 | 5.03 | 8.59 | Times, Frequency of anal sex in the past 12 months |
| 91.62 | 83.56 | 99.10 | weeks, Infection duration of chlamydia trachomatis at throat(asymptomatic infection) |
| 1.56 | 1.48 | 1.67 | weeks, Infection duration at urethral (symptomatic infection) |
| 84.52 | 82.97 | 85.56 | %, proportion of urethral infections that are asymptomatic |
| 81.92 | 74.07 | 87.53 | %, proportion of anal infections that are asymptomatic |
| 12.09 | 9.44 | 13.92 | weeks, Infection duration at urethral (asymptomatic infection) |
| 25.16 | 24.99 | 25.25 | weeks, Infection duration of chlamydia trachomatis at anus (asymptomatic infection) |
| 1.49 | 1.36 | 1.68 | weeks, Infection duration of chlamydia trachomatis at anus (symptomatic infection) |
| 83.59 | 77.70 | 87.06 | %, proportion of MSM received throat swab in the past 12 months |
| 78.08 | 74.69 | 84.28 | %, proportion of MSM received anal swab in the past 12 months |
| 79.28 | 75.84 | 82.62 | %, proportion of MSM received urine test in the past 12 months |
| 28.51 | 27.46 | 30.01 | %, proportion of 'oral sex and anal sex' in the same sex episode |
| 71.56 | 71.11 | 71.82 | %, proportion of 'oral sex and rimming' in the same sex episode |
| 68.39 | 67.91 | 68.98 | %, proportion of saliva use during anal sex |
| 80.00 | 80.00 | 80.00 | %, proportion of receptive oral sex followed by partner's insertive anal sex |
| 80.00 | 80.00 | 80.00 | %, proportion of insertive oral sex followed by partner's insertive rimming |
| 0.84 | 0.73 | 0.90 | %, Infection prevalence of chlamydia trachomatis only at Oropharynx |
| 1.87 | 1.76 | 1.97 | %, Infection prevalence of chlamydia trachomatis only at Urethral |
| 7.50 | 7.36 | 7.70 | %, Infection prevalence of chlamydia trachomatis only at Rectum |
| 0.00 | 0.00 | 0.00 | %, Infection prevalence of chlamydia trachomatis at Oropharynx & Urethra |
| 1.25 | 1.17 | 1.31 | %, Infection prevalence of chlamydia trachomatis at Oropharynx & Rectum |
| 1.19 | 1.11 | 1.30 | %, Infection prevalence of chlamydia trachomatis at Urethra & Rectum |
| 0.19 | 0.15 | 0.23 | %, Infection prevalence of chlamydia trachomatis at Oropharynx & Urethra & Rectum |

Model output parameters of sensitivity analysis: double days of sexual practices

**Table S8. 147.** Model 2’ output parameters with 95% confidence intervals

| **Mean** | **95%CI: lower** | **95%CI: upper** | **Parameters** |
| --- | --- | --- | --- |
| 46.61 | 36.44 | 56.10 | %, Consistent condom usage in anal sex in past 12 months |
| 86.12 | 80.22 | 94.13 | %, Condom efficacy in preventing transmission |
| - | - | - | Times, Frequency of kissing in the past 12 months |
| 31.13 | 3.93 | 55.40 | Times, Frequency of oral sex in the past 12 months |
| 44.44 | 2.72 | 148.95 | Times, Frequency of rimming in the past 12 months |
| 26.75 | 4.79 | 69.09 | Times, Frequency of anal sex in the past 12 months |
[truncated: 193,134 more chars]
