# Supplementary material for: Chlamydia trachomatis transmission between the oropharynx, urethra and anorectum in men who have sex with men: a mathematical model
Source: BMC Med. 2020 Nov 17;18:326. doi: 10.1186/s12916-020-01796-3 (PMC7670797; doi:10.1186/s12916-020-01796-3)
Supplement: Supplementary file 4 — Additional file 4. Additional results: model calibration, estimating the composition of incidence, and sensitivity analysis. Figs. S2a-32c. [file 12916_2020_1796_MOESM4_ESM.docx]

**Supplementary results: Transmission models without considering sequential sexual practices (model 1-3)**

**Unpublished data from 4888 MSM attending Melbourne Sexual Health Centre for model (model 1-3) calibration**


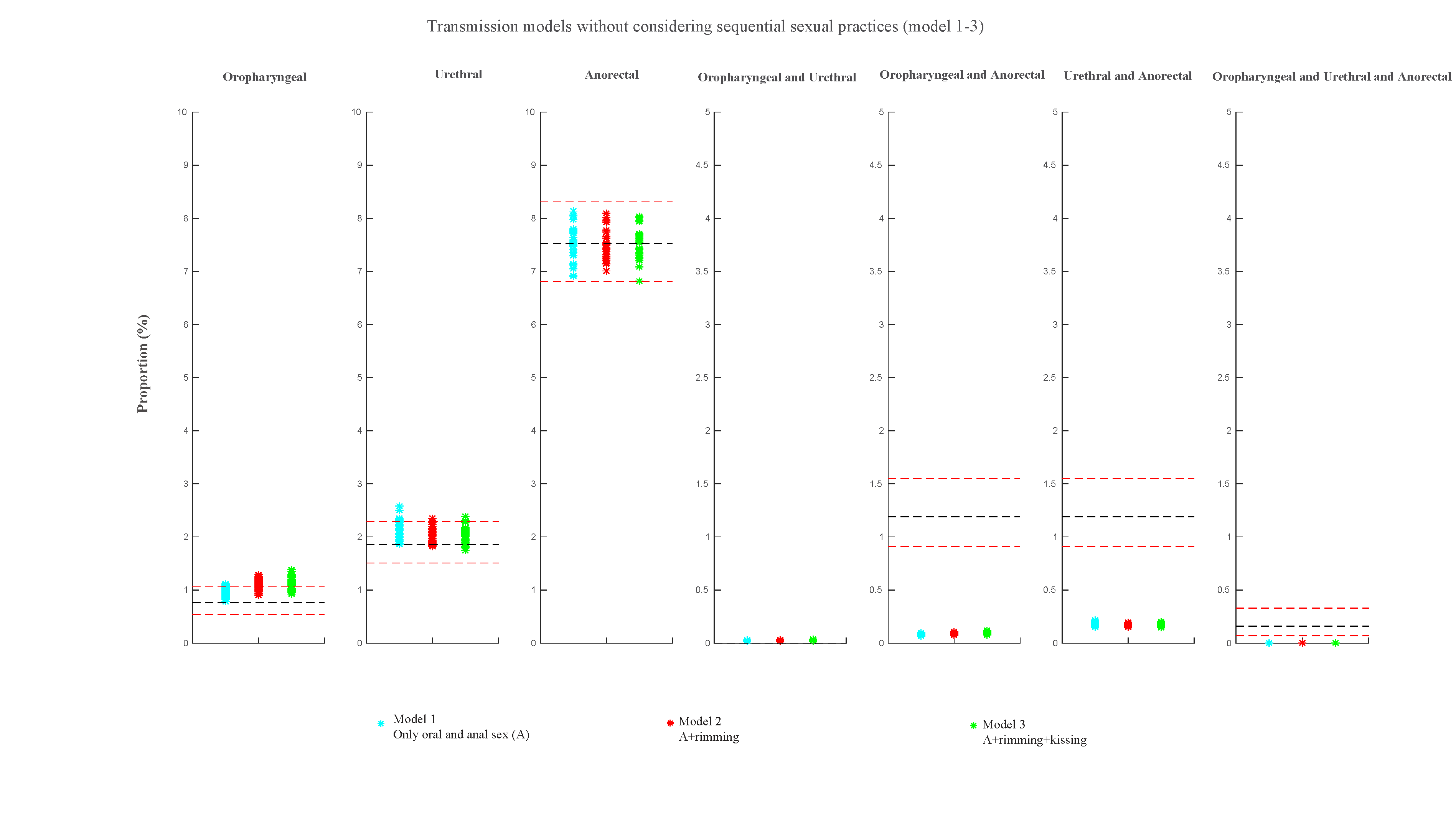


**Figure S2a.** Estimates of the three models for the percentage of specific anatomical sites positive for *Chlamydia trachomatis* for the three models (model 1-3) and the 95% confidence intervals for the observed site-specific positivity among 4888 MSM attending Melbourne Sexual Health Centre in 2018 and 2019


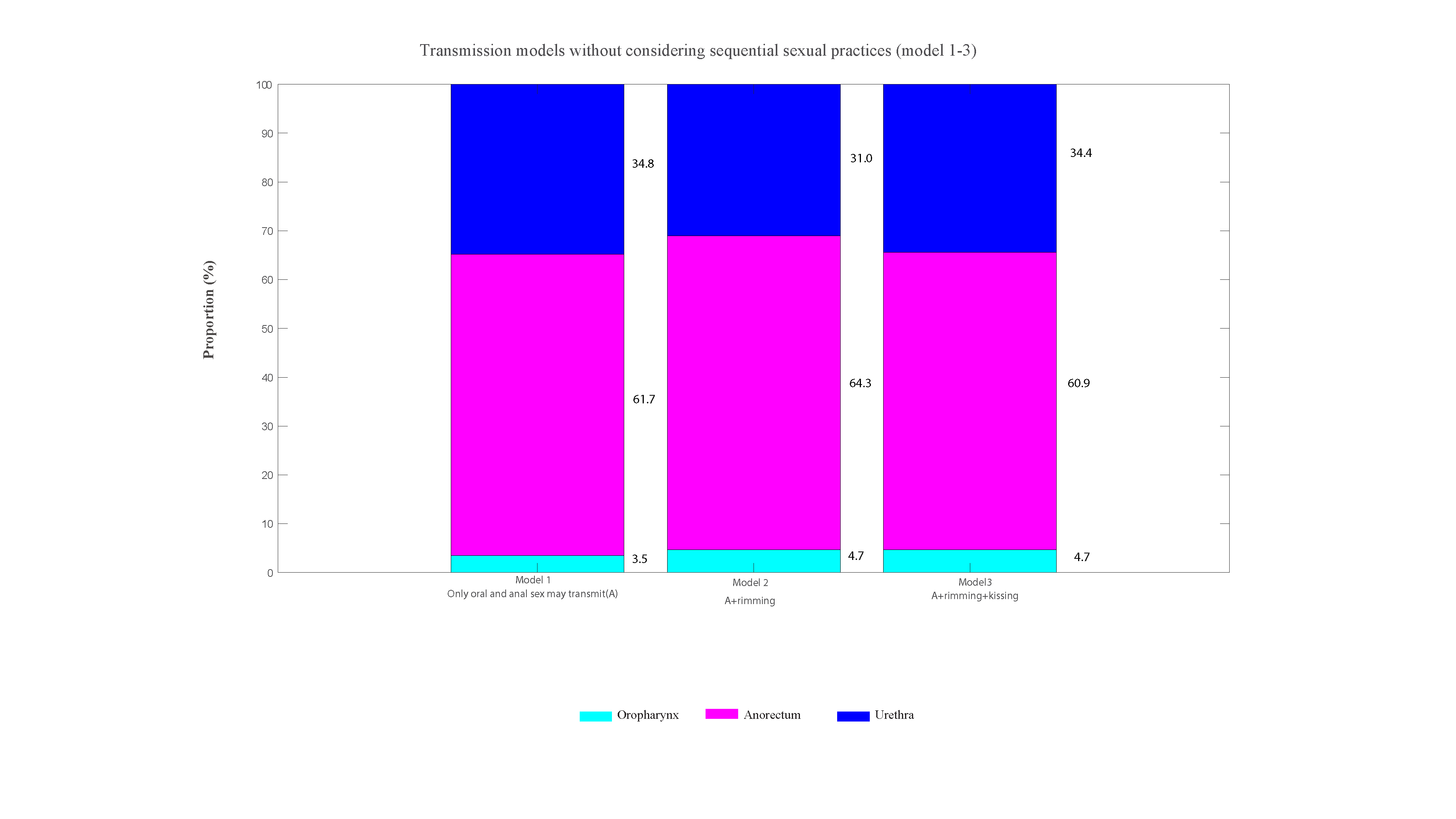


**Figure S2b.** Estimated proportion of incident *Chlamydia trachomatis* cases that occur at the oropharynx, anorectum or urethra in MSM from the three models (model 1-3) among 4888 MSM attending Melbourne Sexual Health Centre in 2018 and 2019


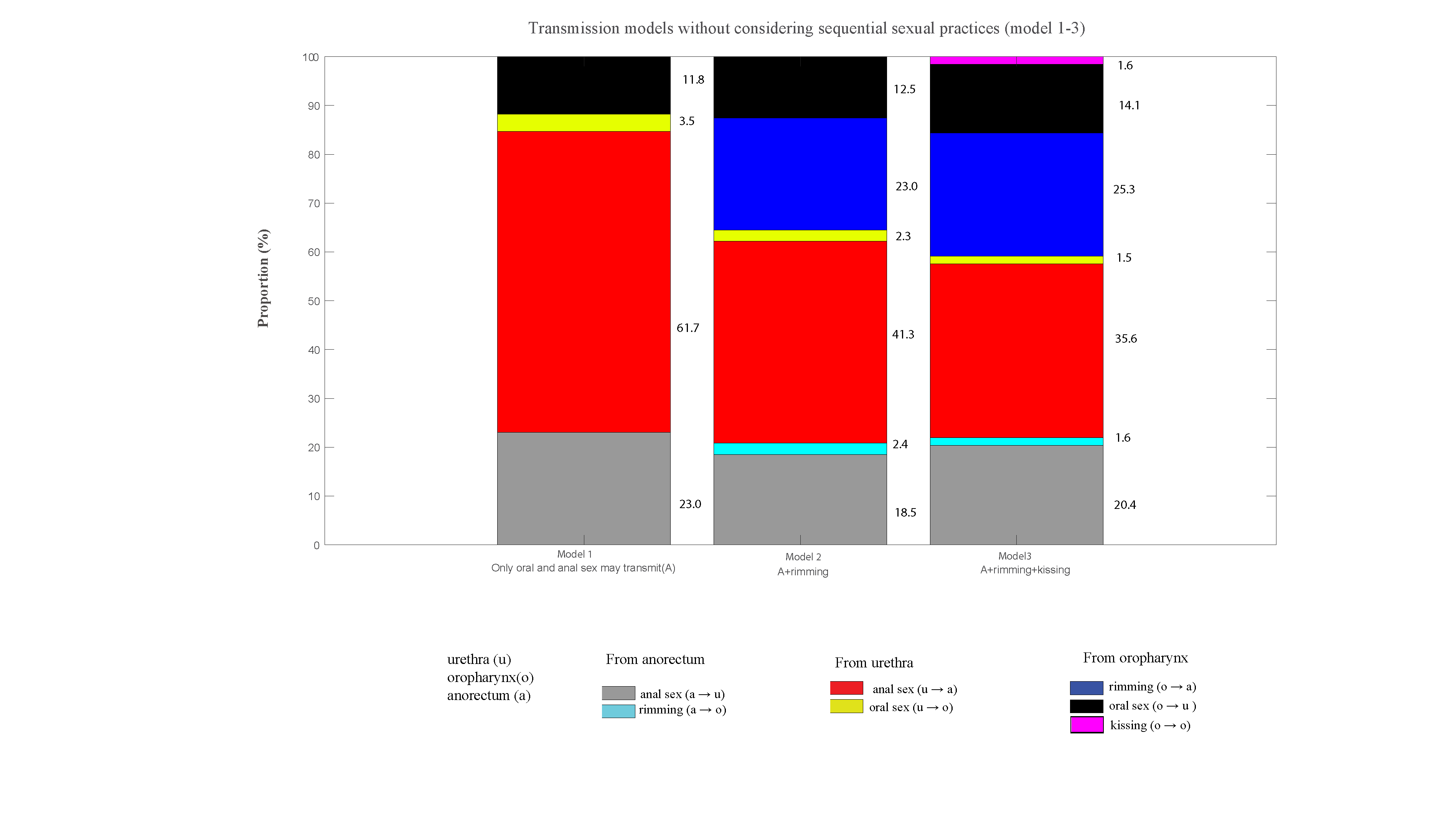


**Figure S2c.** Estimated proportion of incident *Chlamydia trachomatis* cases caused by sexual practices in MSM from the three models (model 1-3) among 4888 MSM attending Melbourne Sexual Health Centre in 2018 and 2019

**Validation of Results (Dataset 1): Published** **validation data from 1,011 asymptomatic MSM attending Melbourne Sexual Health Centre**

**
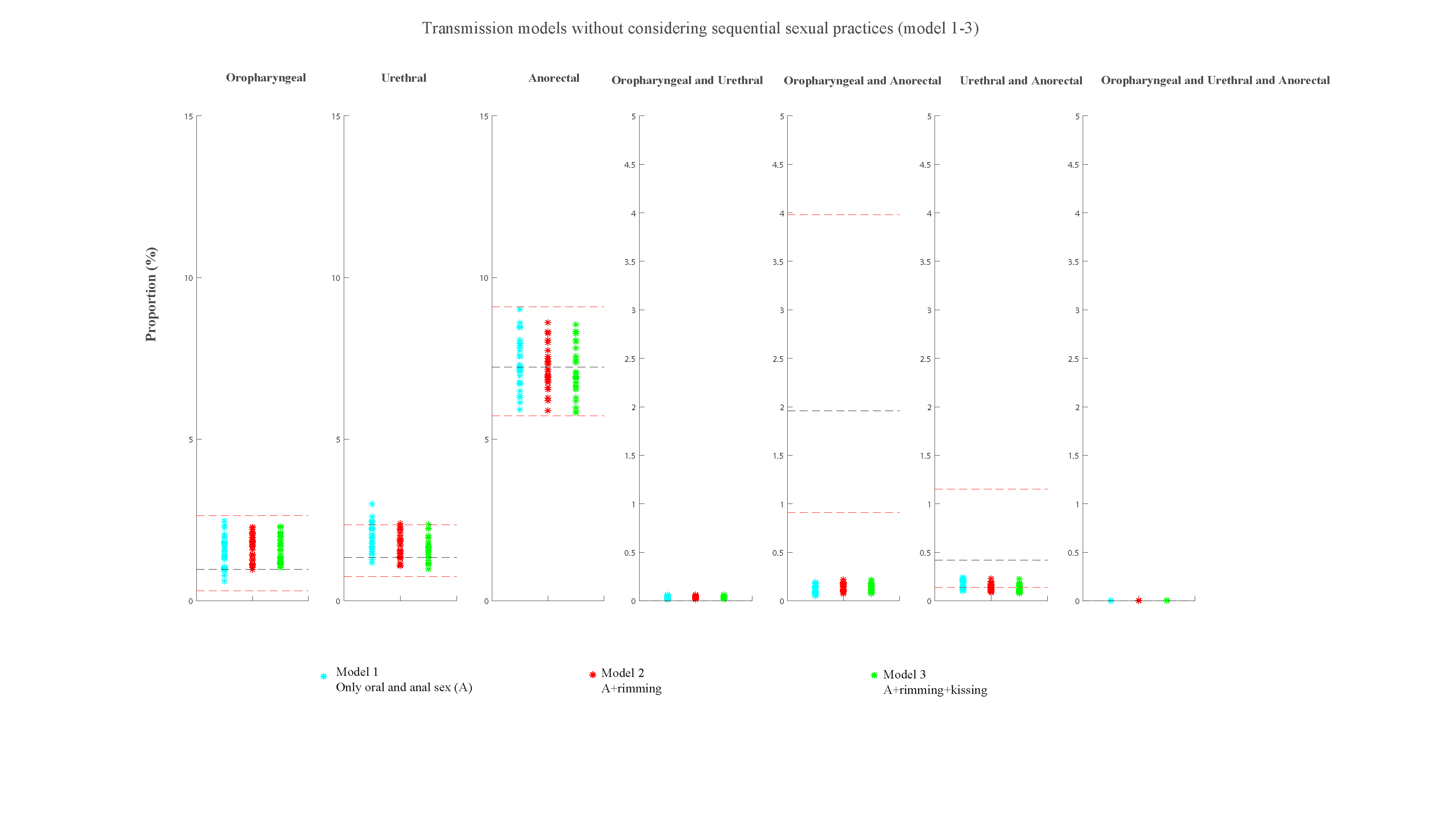
**

**Figure S3a.** Estimates of the three models for the percentage of specific anatomical sites positive for *Chlamydia trachomatis* for the three models (model 1-3) and the 95% confidence intervals for the observed site-specific positivity among 1,011 asymptomatic MSM attending MSHC during 2016–2017

**
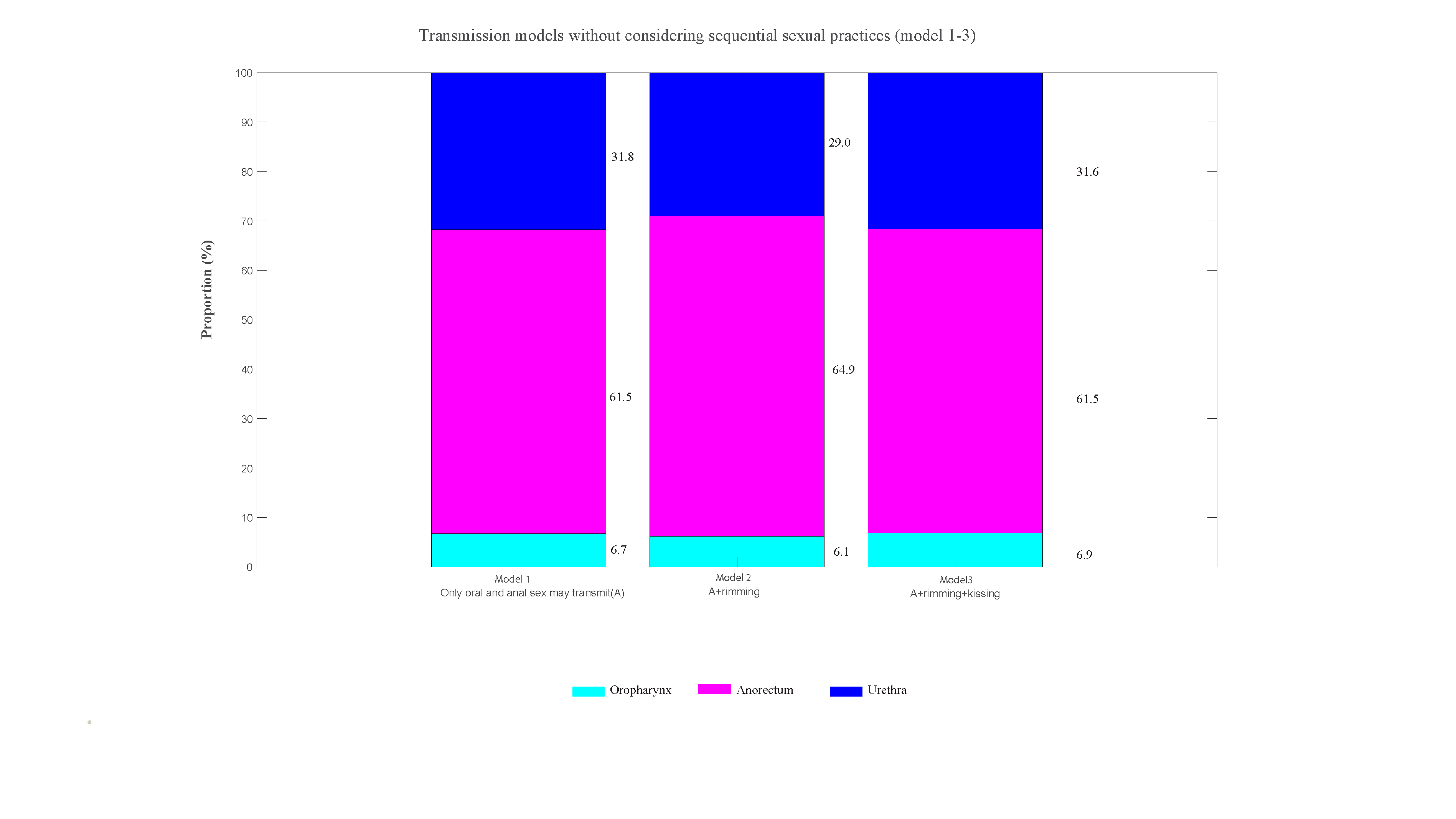
**

**Figure S3b.** Estimated proportion of incident *Chlamydia trachomatis* cases that occur at the oropharynx, anorectum or urethra in MSM from the three models (model 1-3) among 1,011 asymptomatic MSM attending MSHC during 2016–2017

**
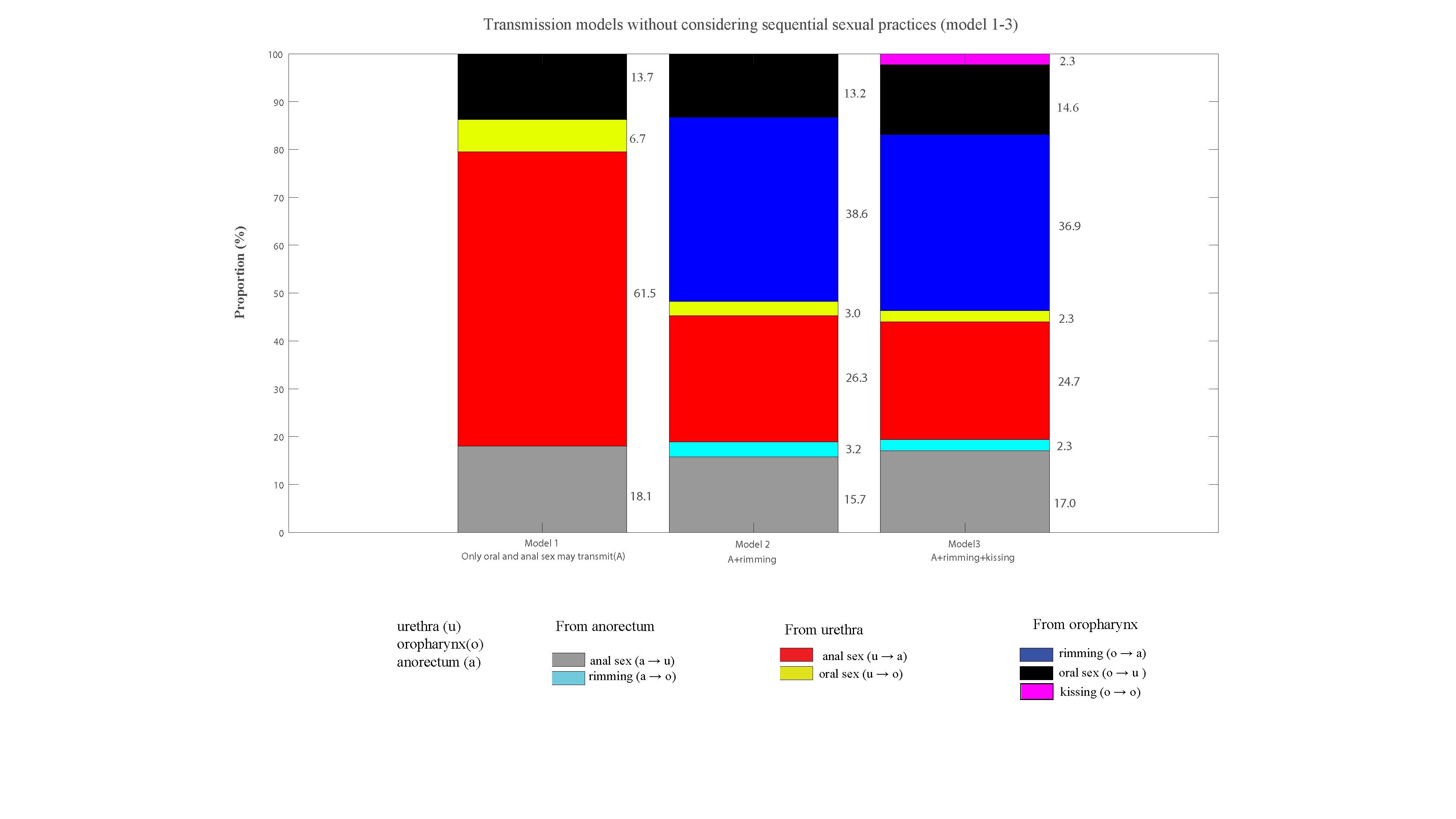
**

**Figure S3c.** Estimated proportion of incident *Chlamydia trachomatis* cases caused by sexual practices in MSM from the three models (model 1-3) among 1,011 asymptomatic MSM attending MSHC during 2016–2017

**Validation of Results (Dataset 2): Published validation data from 393 MSM attending STD & HIV care clinics in the USA**


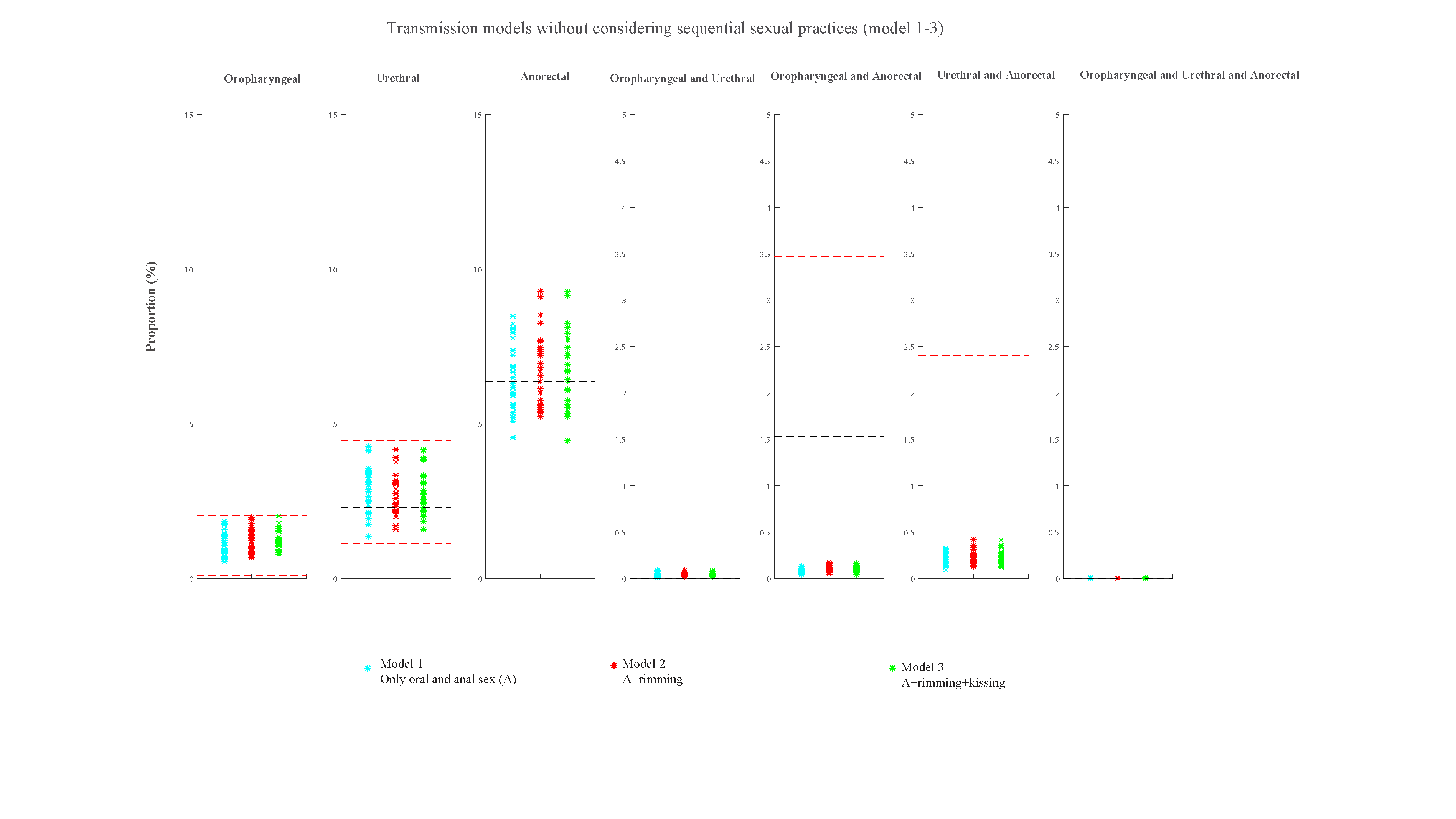


**Figure S4a.** Estimates of the three models for the percentage of specific anatomical sites positive for *Chlamydia trachomatis* for the three models (model 1-3) and the 95% confidence intervals for the observed site-specific positivity among 393MSM attending STD & HIV care clinics in the USA


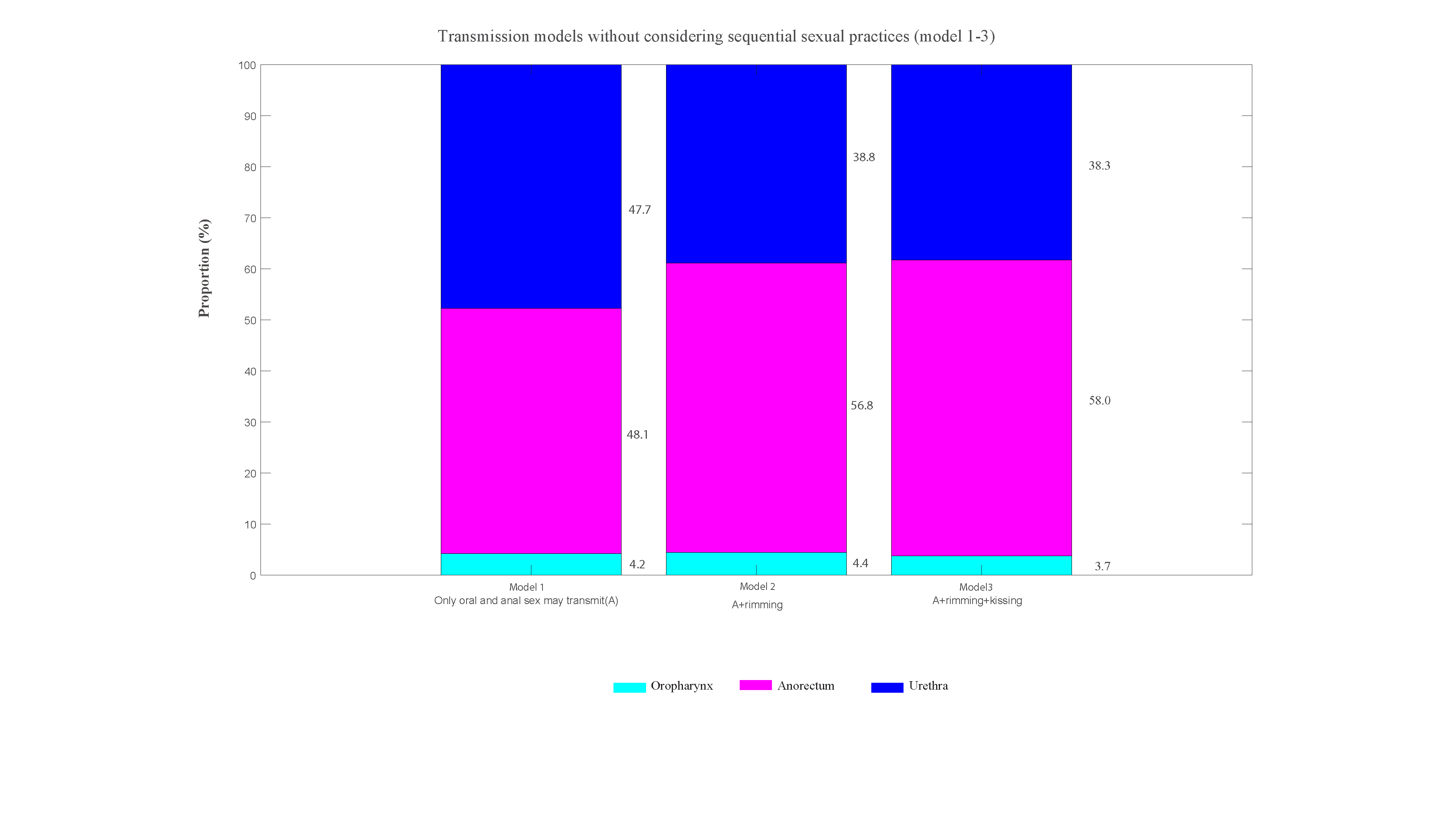


**Figure S4b.** Estimated proportion of incident *Chlamydia trachomatis* cases that occur at the oropharynx, anorectum or urethra in MSM from the three models (model 1-3) among 393MSM attending STD & HIV care clinics in the USA


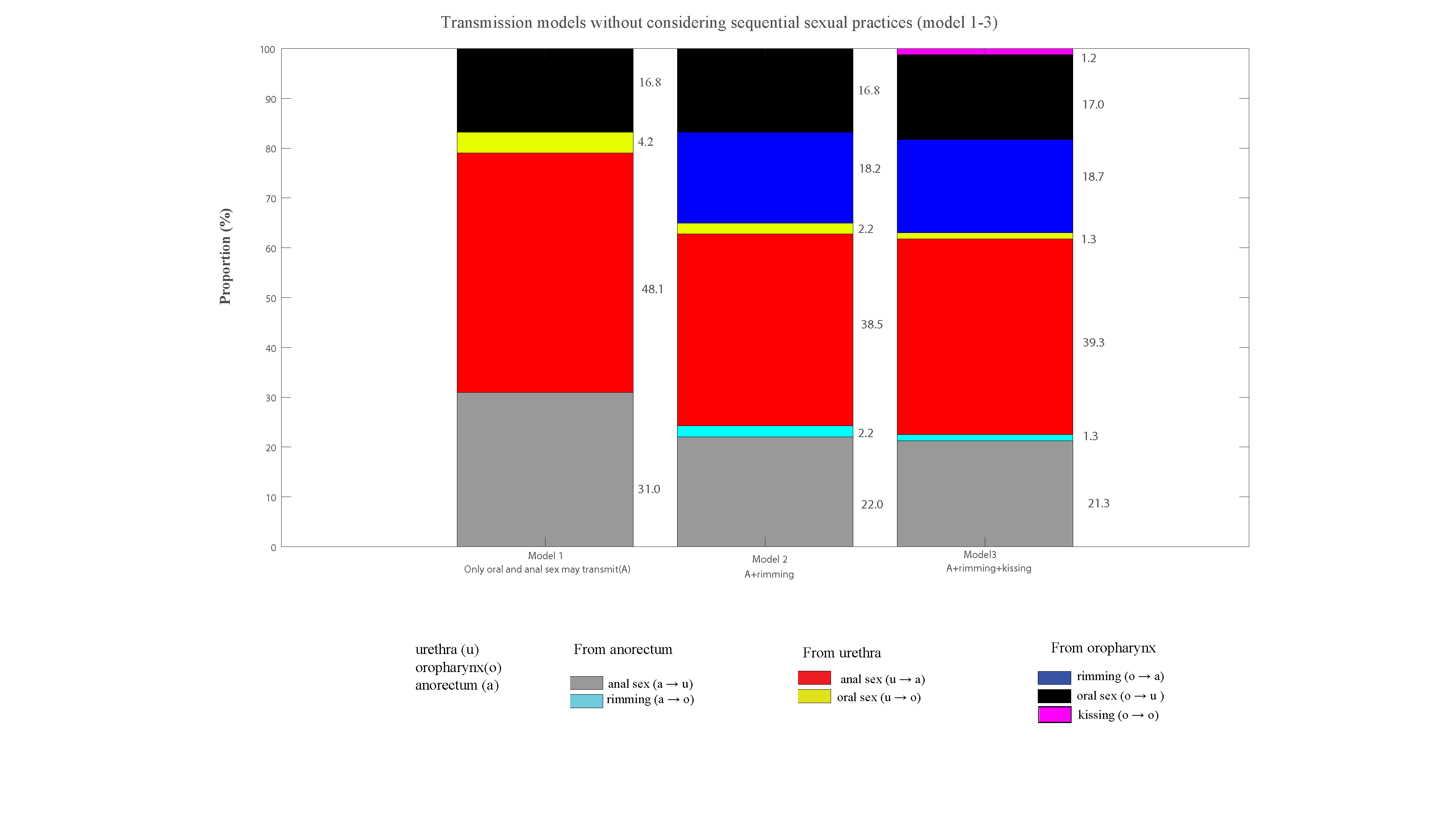


**Figure S4c**. Estimated proportion of incident *Chlamydia trachomatis* cases caused by sexual practices in MSM from the three models (model 1-3) among 393MSM attending STD & HIV care clinics in the USA

Validation of Results (Dataset 3): Published validation data from MSM surveillance data of all Dutch STI clinics

**
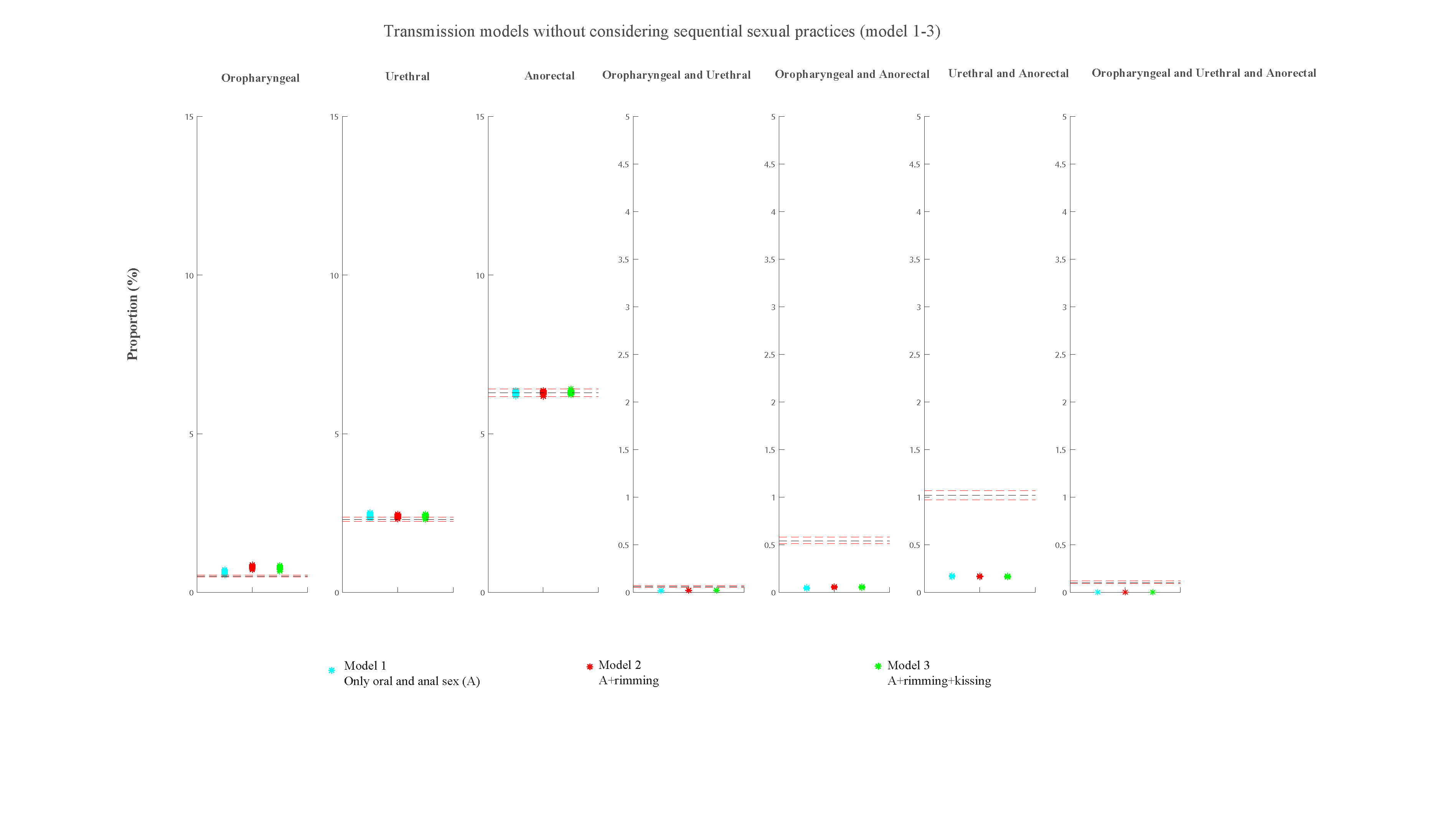
**

**Figure S5a**. Estimates of the three models for the percentage of specific anatomical sites positive for *Chlamydia trachomatis* for the three models (model 1-3) and the 95% confidence intervals for the observed site-specific positivity among MSM surveillance data (271, 242 consultations) from all Dutch STI clinics during 2008-2017

**
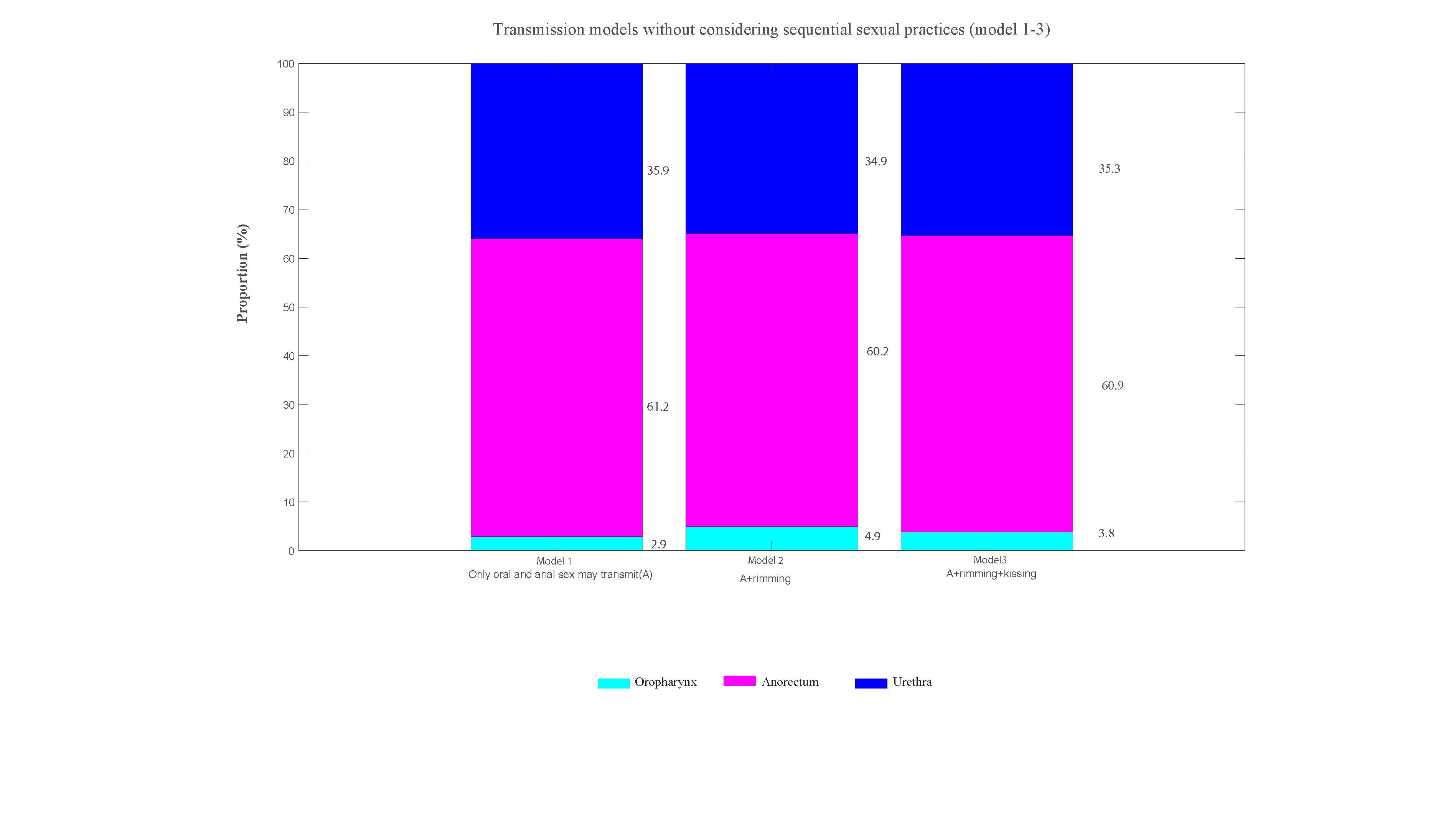
**

**Figure S5b.** Estimated proportion of incident *Chlamydia trachomatis* cases that occur at the oropharynx, anorectum or urethra in MSM from the three models (model 1-3) among MSM surveillance data (271, 242 consultations) from all Dutch STI clinics during 2008-2017

**
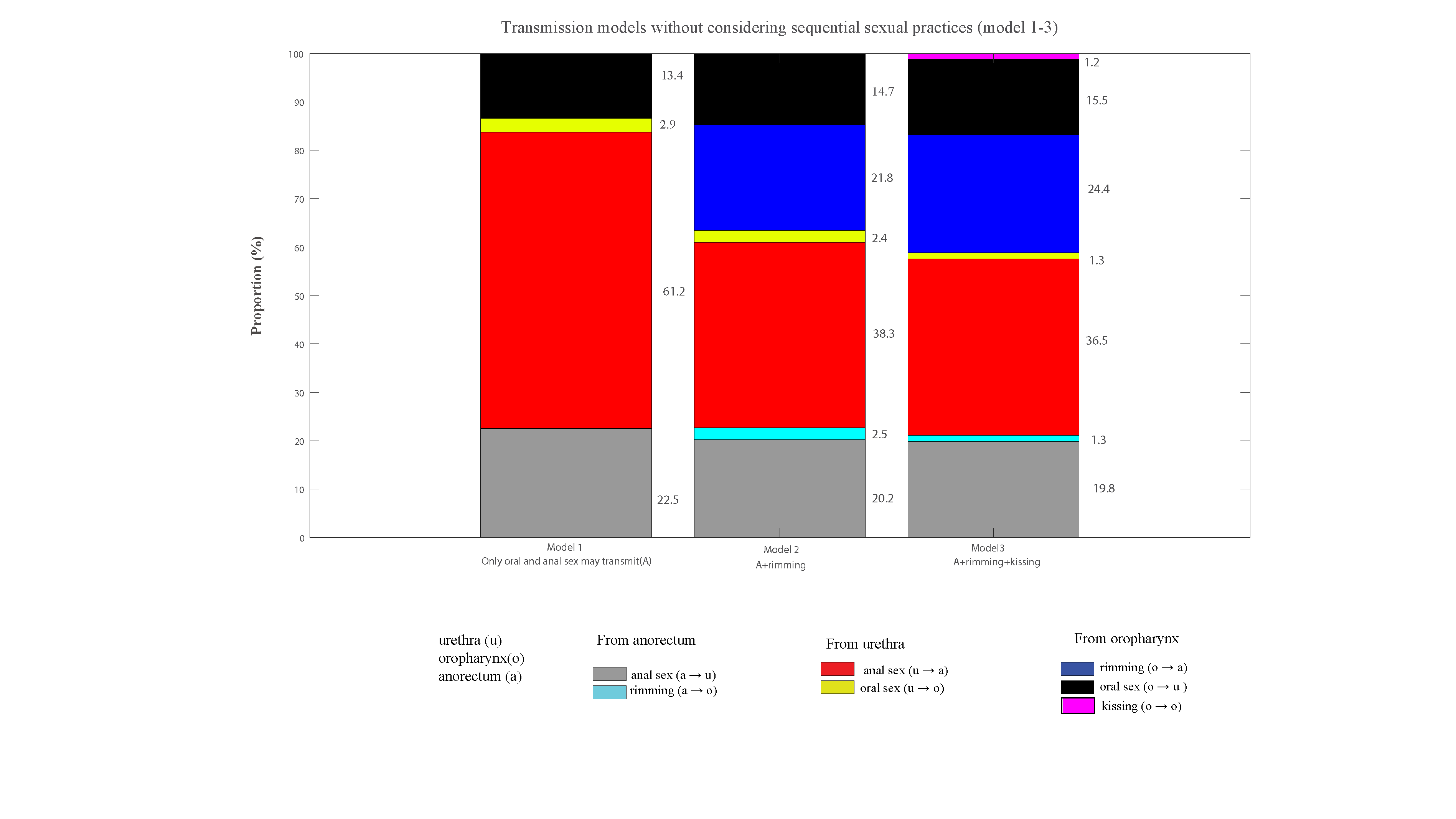
**

**Figure S5c**. Estimated proportion of incident *Chlamydia trachomatis* cases caused by sexual practices in MSM from the three models (model 1-3) among MSM surveillance data (271, 242 consultations) from all Dutch STI clinics during 2008-2017

**Validation of Results (Dataset 4): Published validation data from 1,610 community MSM in Thailand**


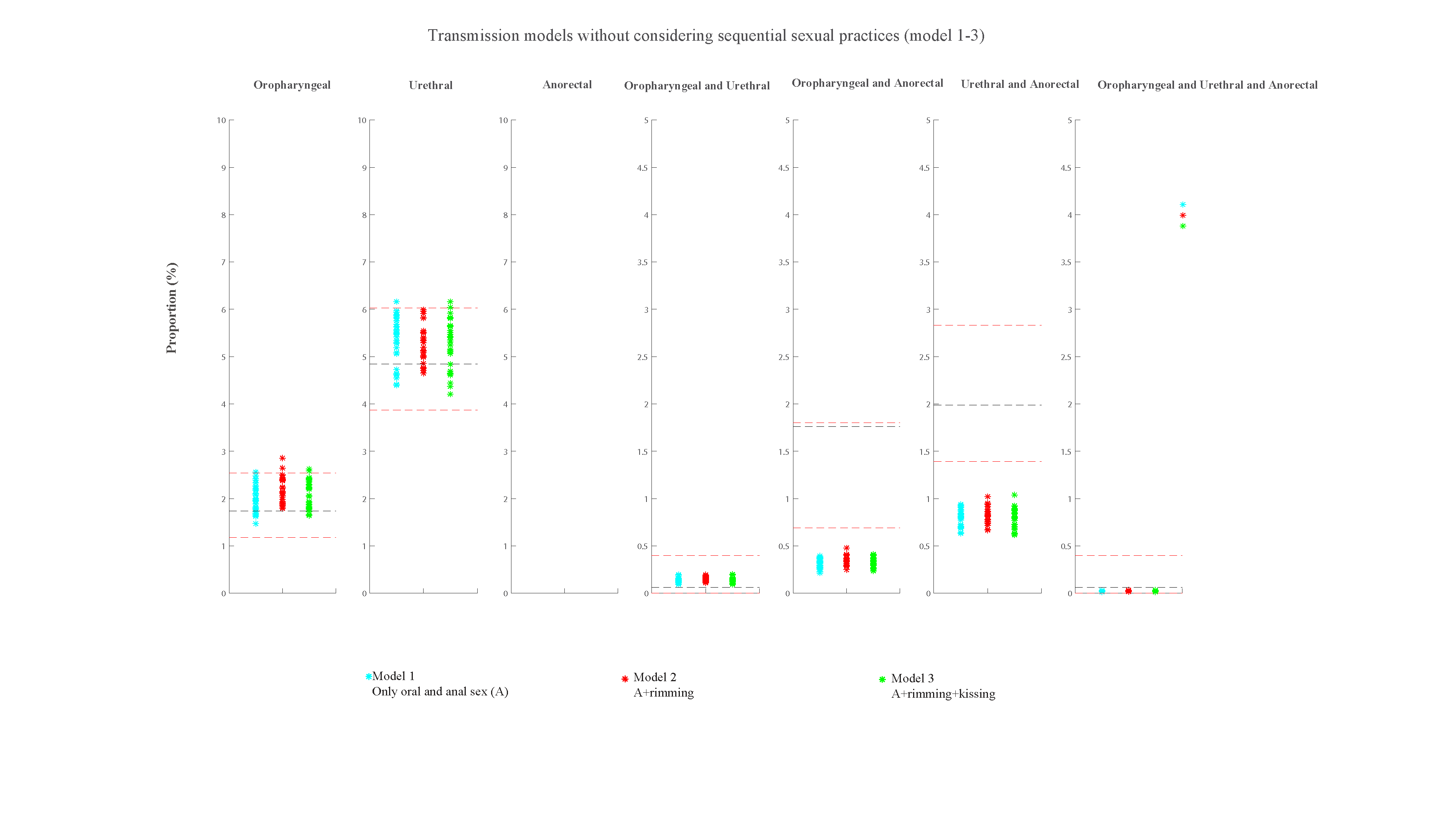


**Figure S6a**. Estimates of the three models for the percentage of specific anatomical sites positive for *Chlamydia trachomatis* for the three models (model 1-3) and the 95% confidence intervals for the observed site-specific positivity among 1,610 MSM attending a community-led test and treat cohort in Thailand between October 2015 and October 2016


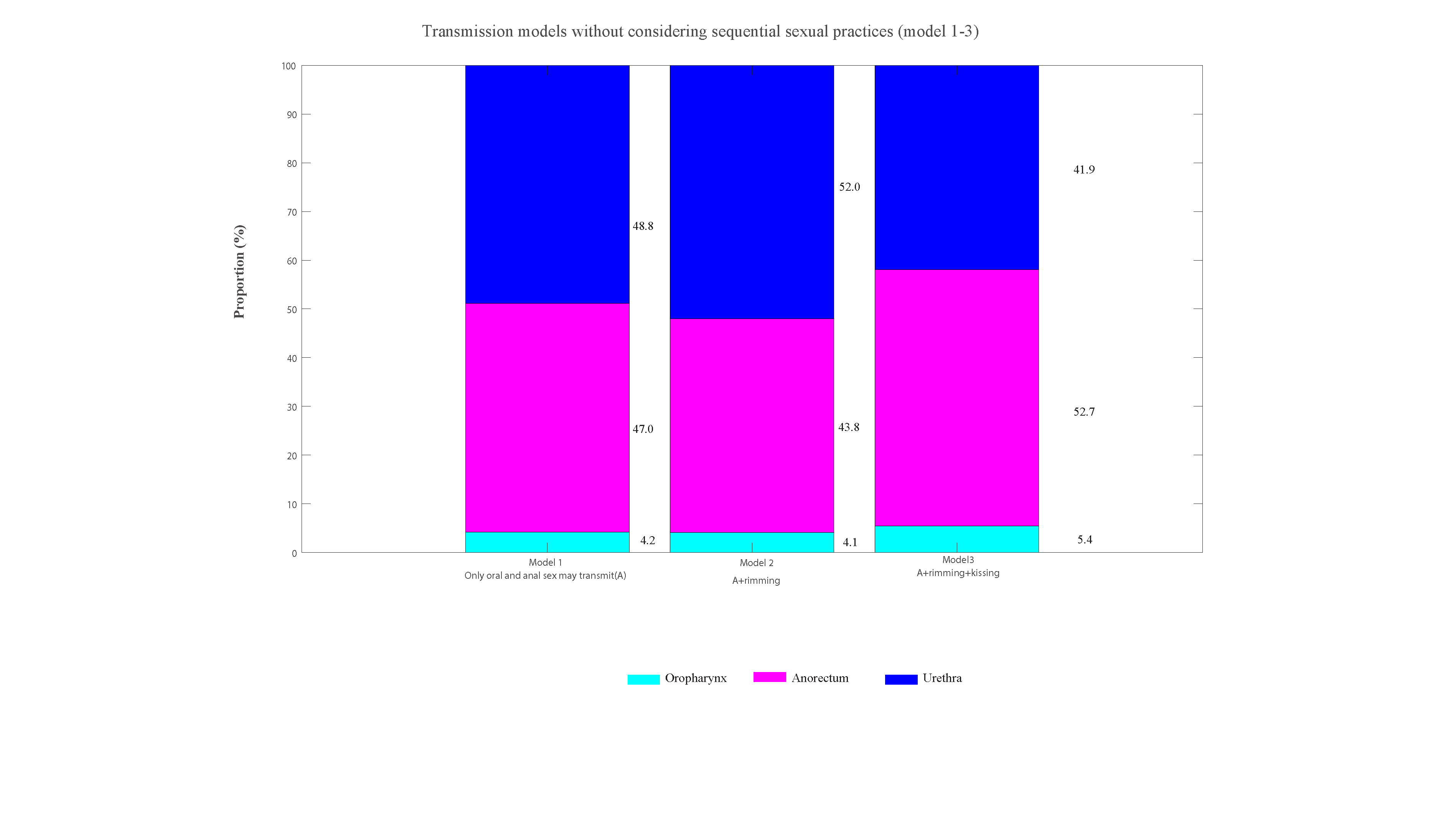


**Figure S6b.** Estimated proportion of incident *Chlamydia trachomatis* cases that occur at the oropharynx, anorectum or urethra in MSM from the three models (model 1-3) among 1,610 MSM attending a community-led test and treat cohort in Thailand between October 2015 and October 2016


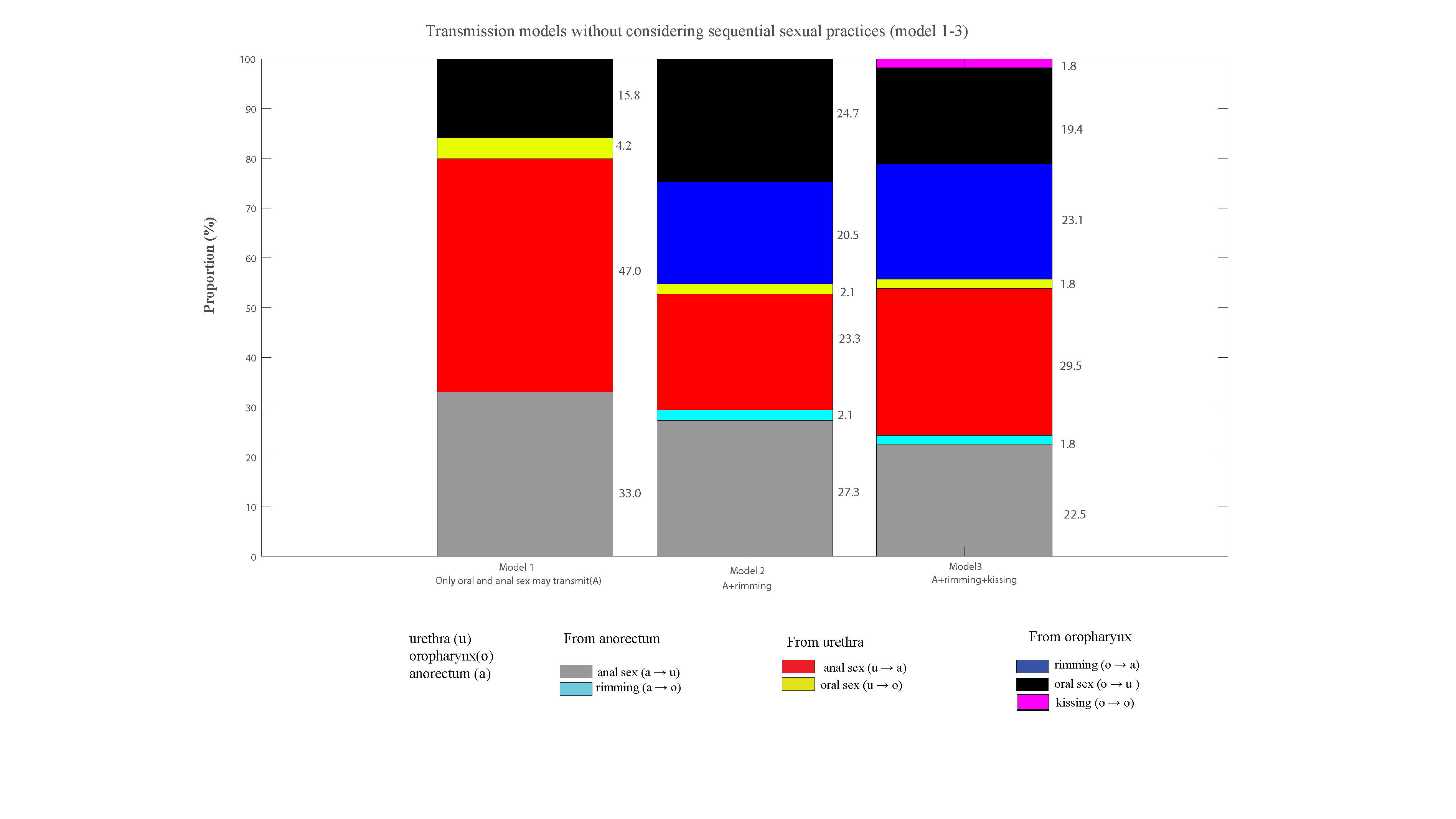


**Figure S6c.** Estimated proportion of incident *Chlamydia trachomatis* cases caused by sexual practices in MSM from the three models (model 1-3) among 1,610 MSM attending a community-led test and treat cohort in Thailand between October 2015 and October 2016

**Validation of Results (Dataset 5): Published validation data from 179 MSM with HIV in the USA**


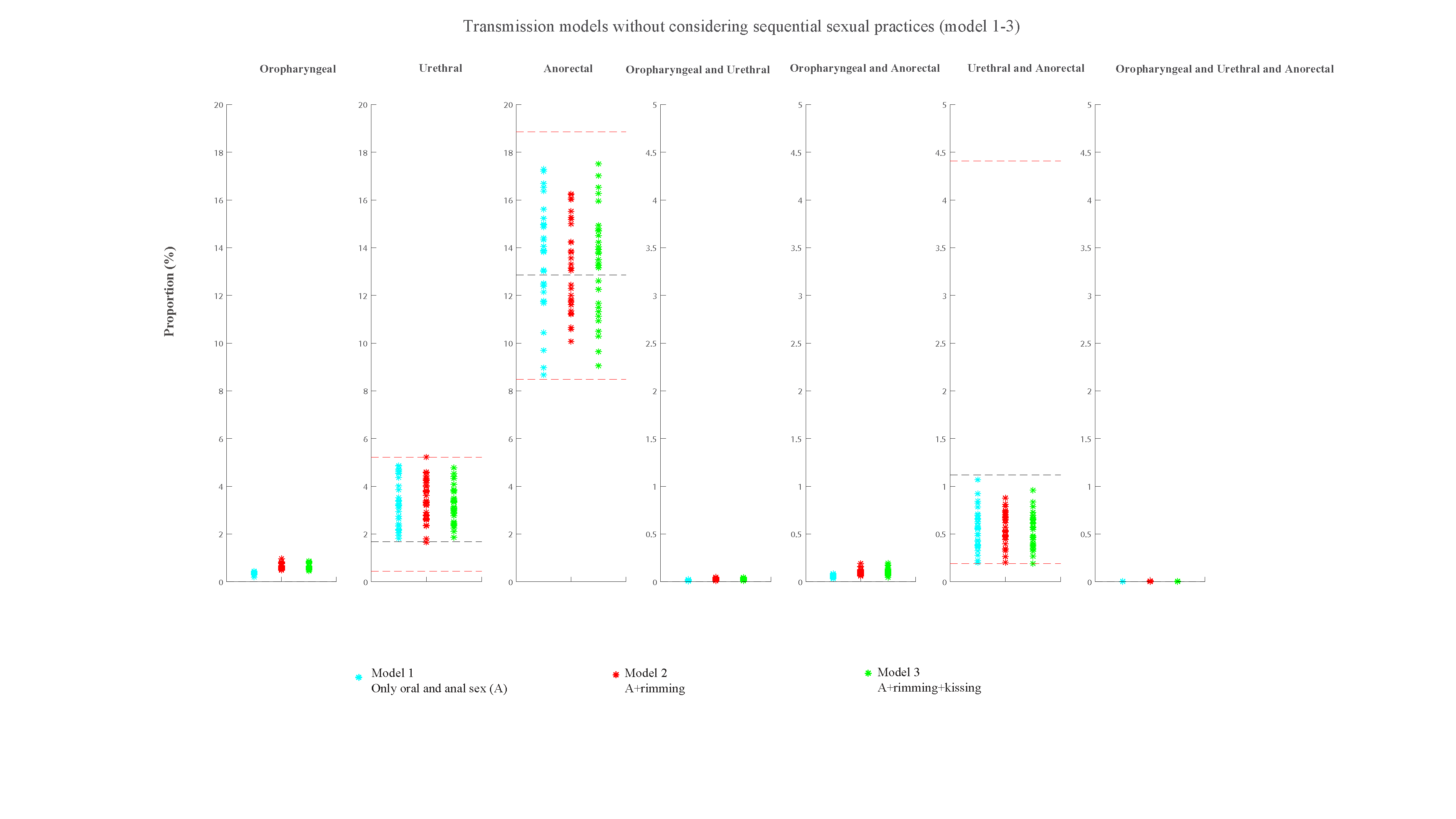


**Figure S7a.** Estimates of the three models for the percentage of specific anatomical sites positive for *Chlamydia trachomatis* for the three models (model 1-3) and the 95% confidence intervals for the observed site-specific positivity among179 MSM with HIV


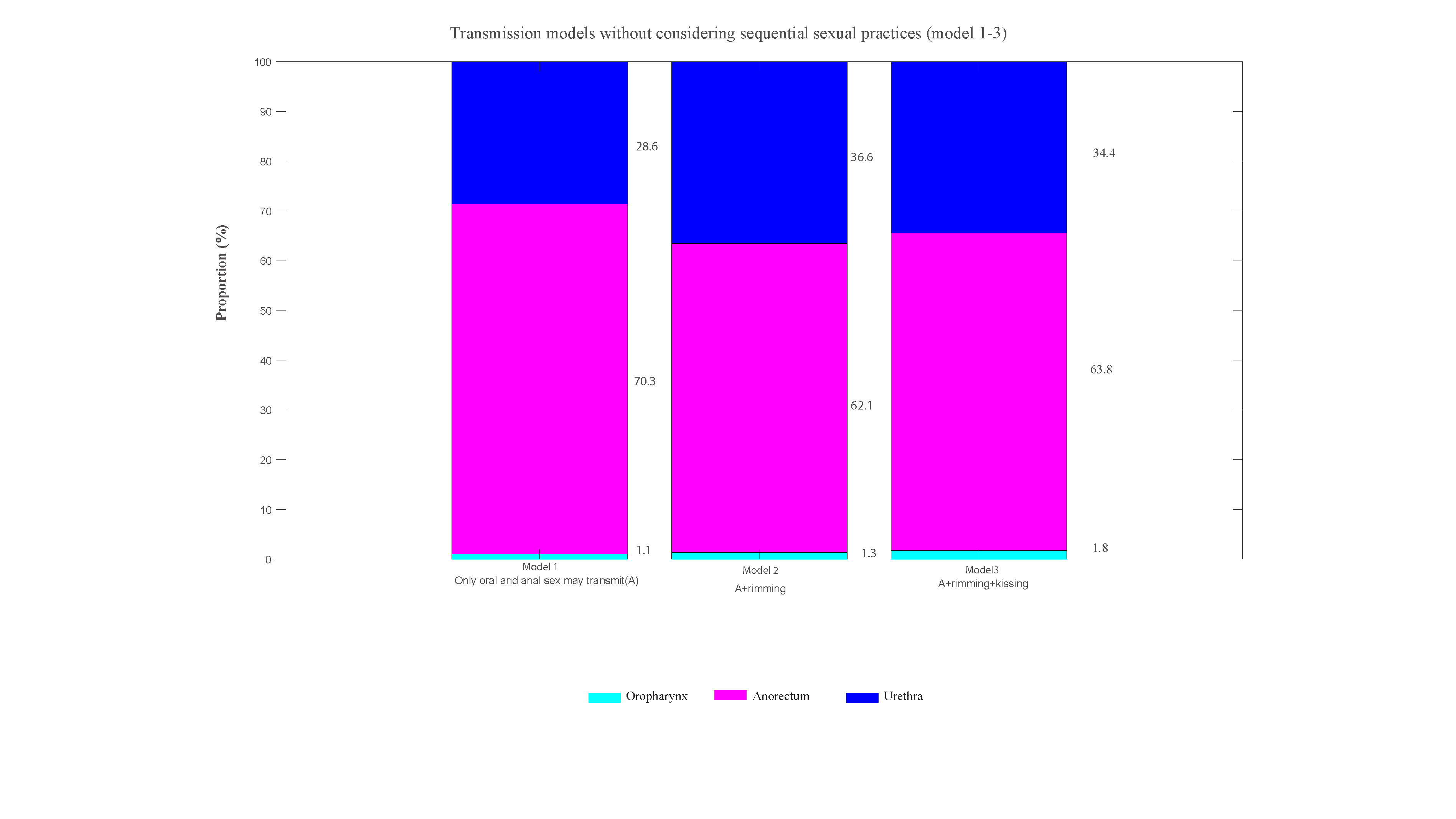


**Figure S7b**. Estimated proportion of incident *Chlamydia trachomatis* cases that occur at the oropharynx, anorectum or urethra in MSM from the three models (model 1-3) among179 MSM with HIV


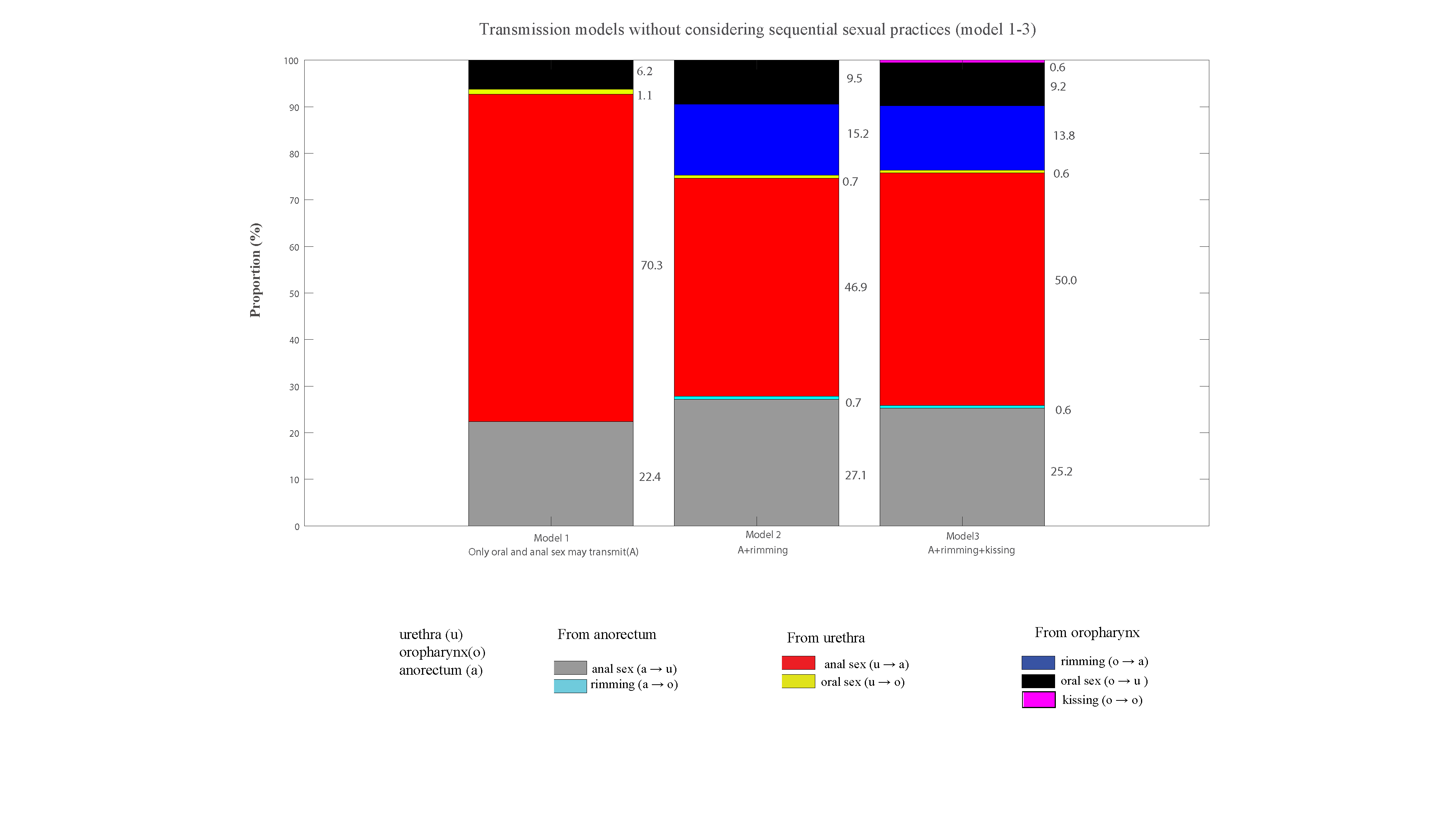


**Figure S7c.** Estimated proportion of incident *Chlamydia trachomatis* cases caused by sexual practices in MSM from the three models (model 1-3) among179 MSM with HIV

**Supplementary results: ‘Anal sex, oral sex and sequential sexual practices’ transmission models (Model 1, 4-6)**

**Unpublished data from 4888 MSM attending Melbourne Sexual Health Centre for model (model 1, 4-6) calibration**


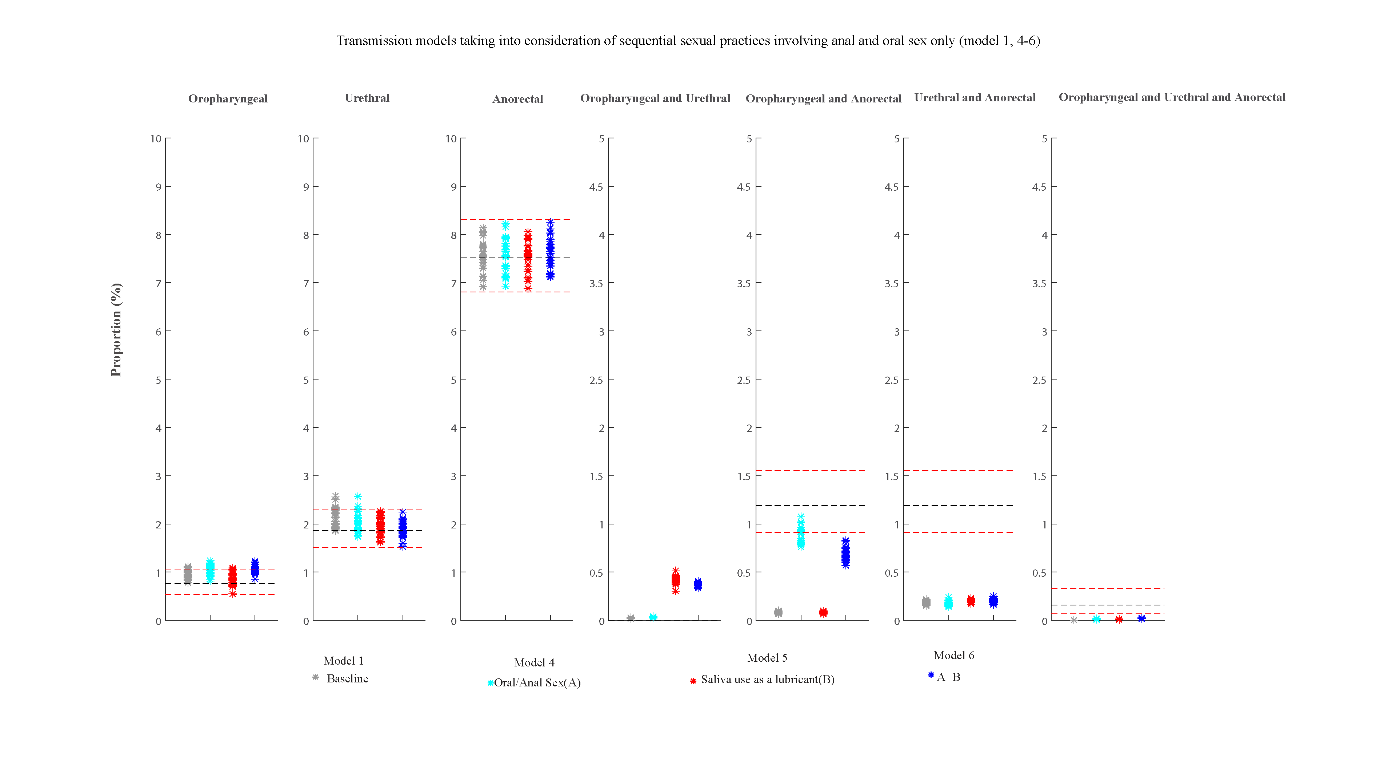


**Figure S8a.** Estimates of the eight models for the percentage of specific anatomical sites positive for *Chlamydia trachomatis* for the four models (model 1, 4-6) and the 95% confidence intervals for the observed site-specific positivity among 4888 MSM attending Melbourne Sexual Health Centre in 2018 and 2019


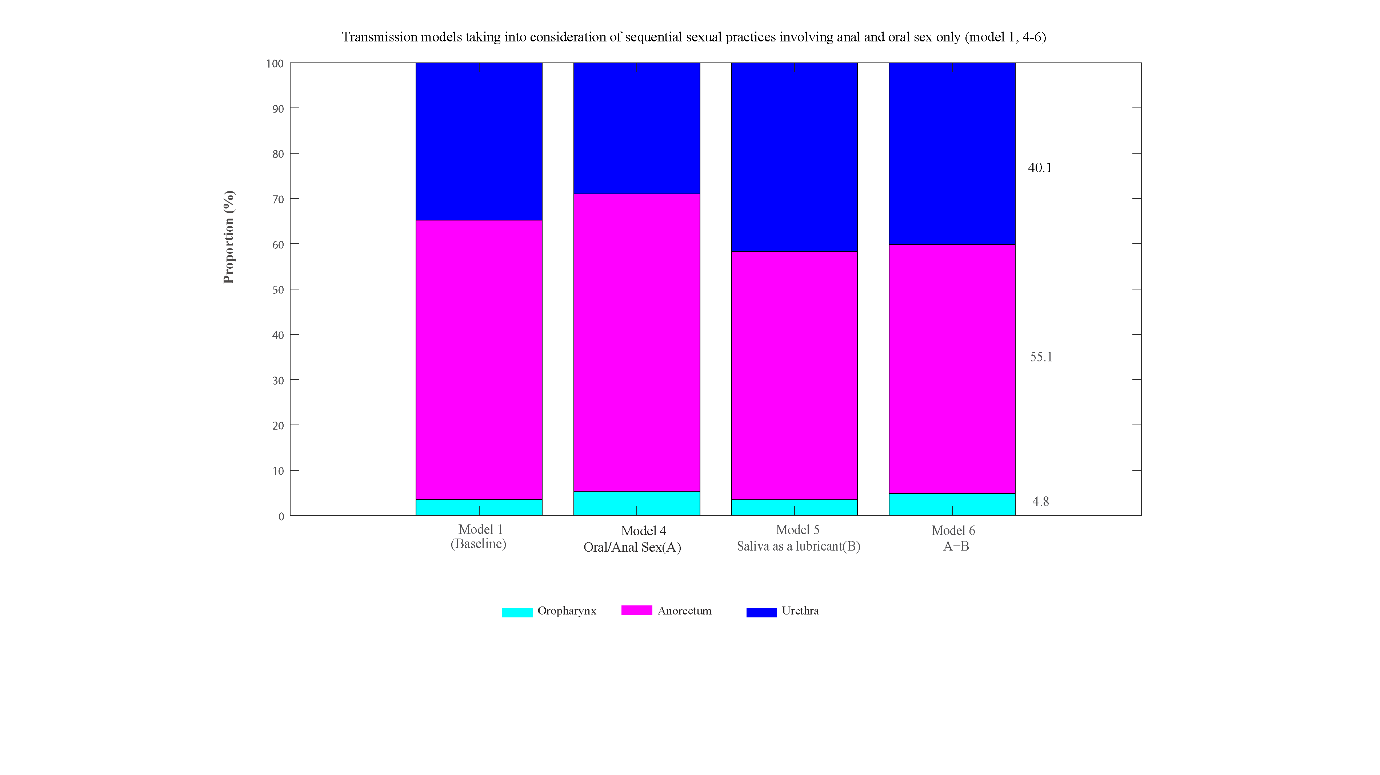


**Figure S8b.** Estimated proportion of incident *Chlamydia trachomatis* cases that occur at the oropharynx, anorectum or urethra in MSM from the four models (model 1, 4-6) among 4888 MSM attending Melbourne Sexual Health Centre in 2018 and 2019

**
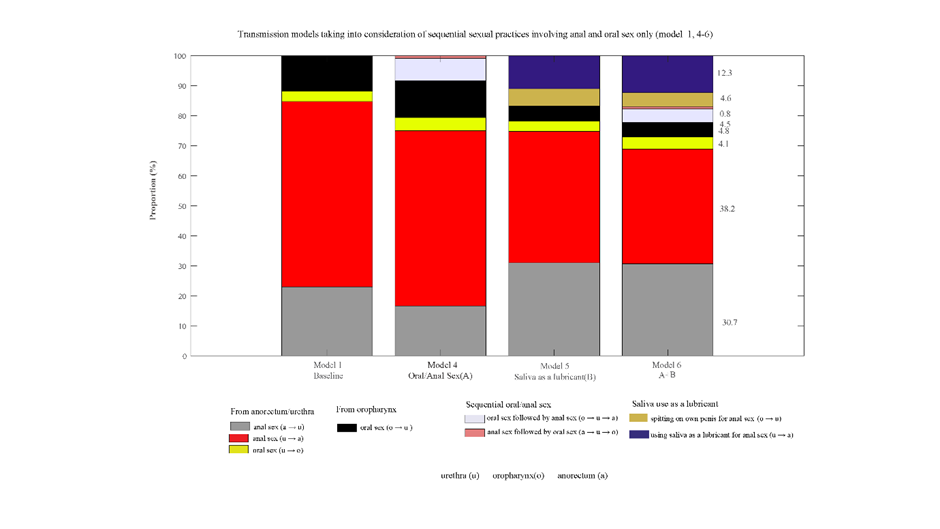
**

**Figure S8c.** Estimated proportion of incident *Chlamydia trachomatis* cases caused by sexual practices in MSM from the four models (model 1, 4-6) among 4888 MSM attending Melbourne Sexual Health Centre in 2018 and 2019

**Validation of Results (Dataset 1): Published validation data from 1,011 asymptomatic MSM attending Melbourne Sexual Health Centre**


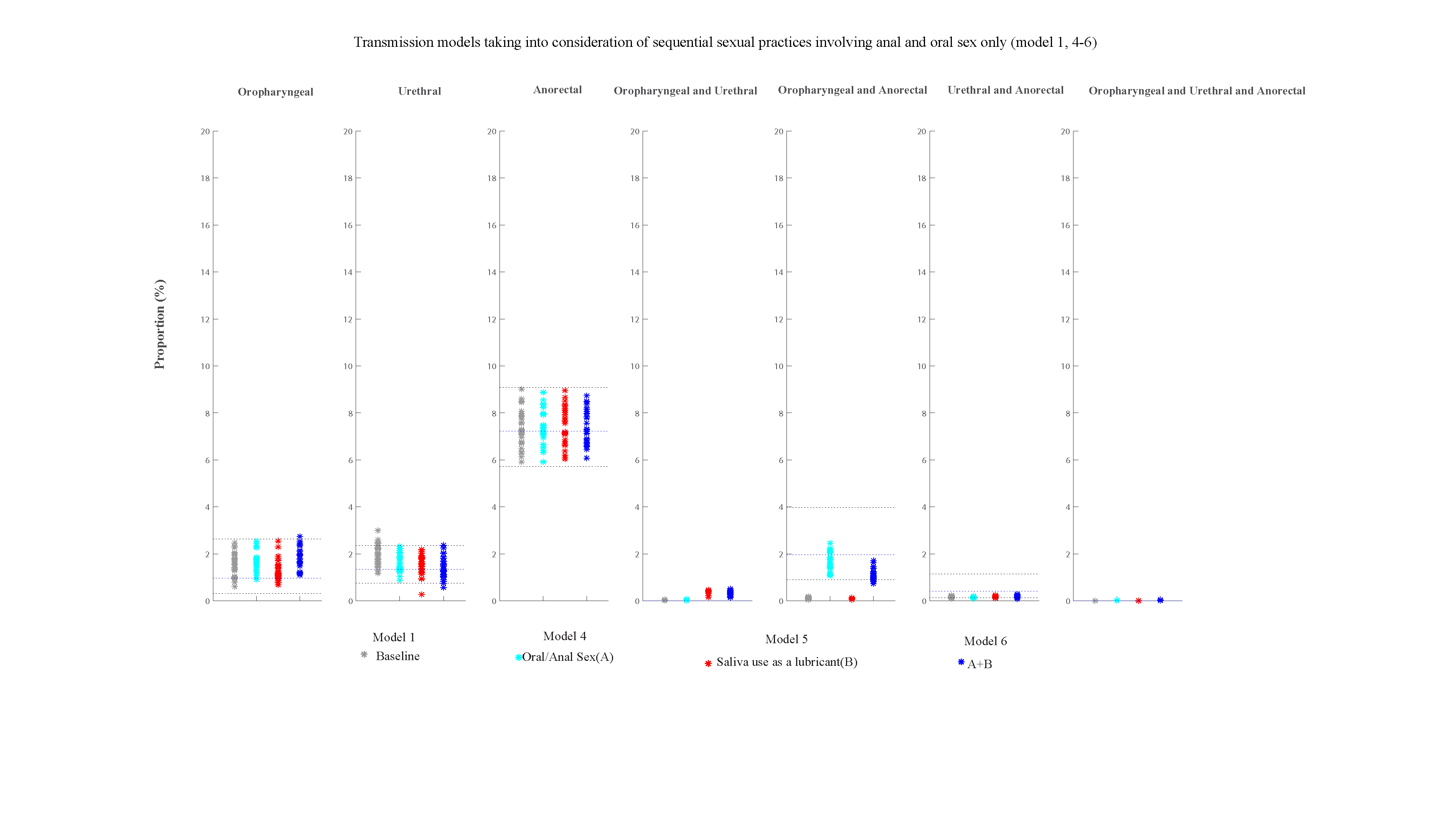


**Figure S9a**. Estimates of the eight models for the percentage of specific anatomical sites positive for *Chlamydia trachomatis* for the four models (model 1, 4-6) and the 95% confidence intervals for the observed site-specific positivity among 1,011 asymptomatic MSM attending MSHC during 2016–2017


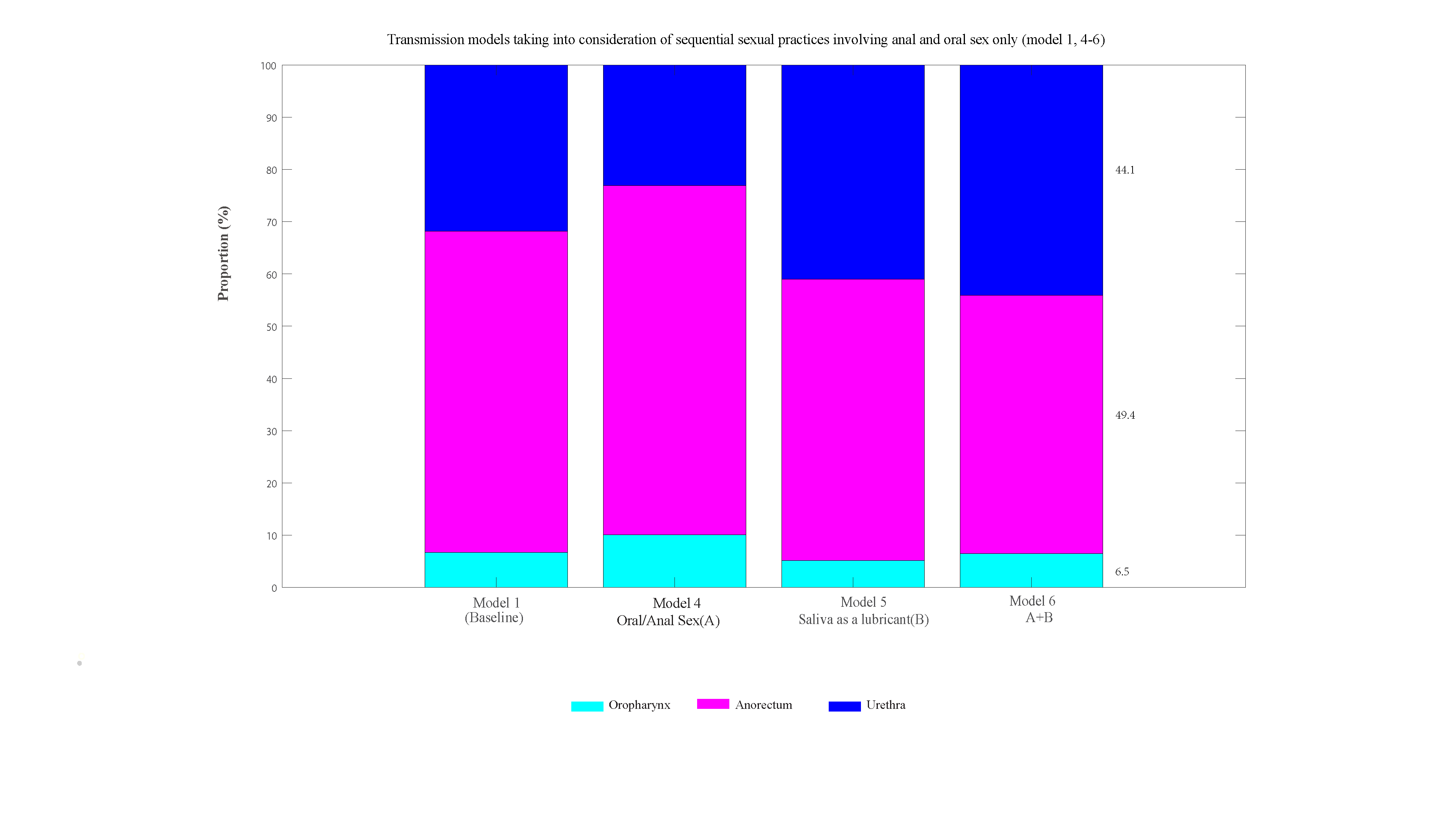


**Figure S9b.** Estimated proportion of incident *Chlamydia trachomatis* cases that occur at the oropharynx, anorectum or urethra in MSM from the four models (model 1, 4-6) among 1,011 asymptomatic MSM attending MSHC during 2016–2017


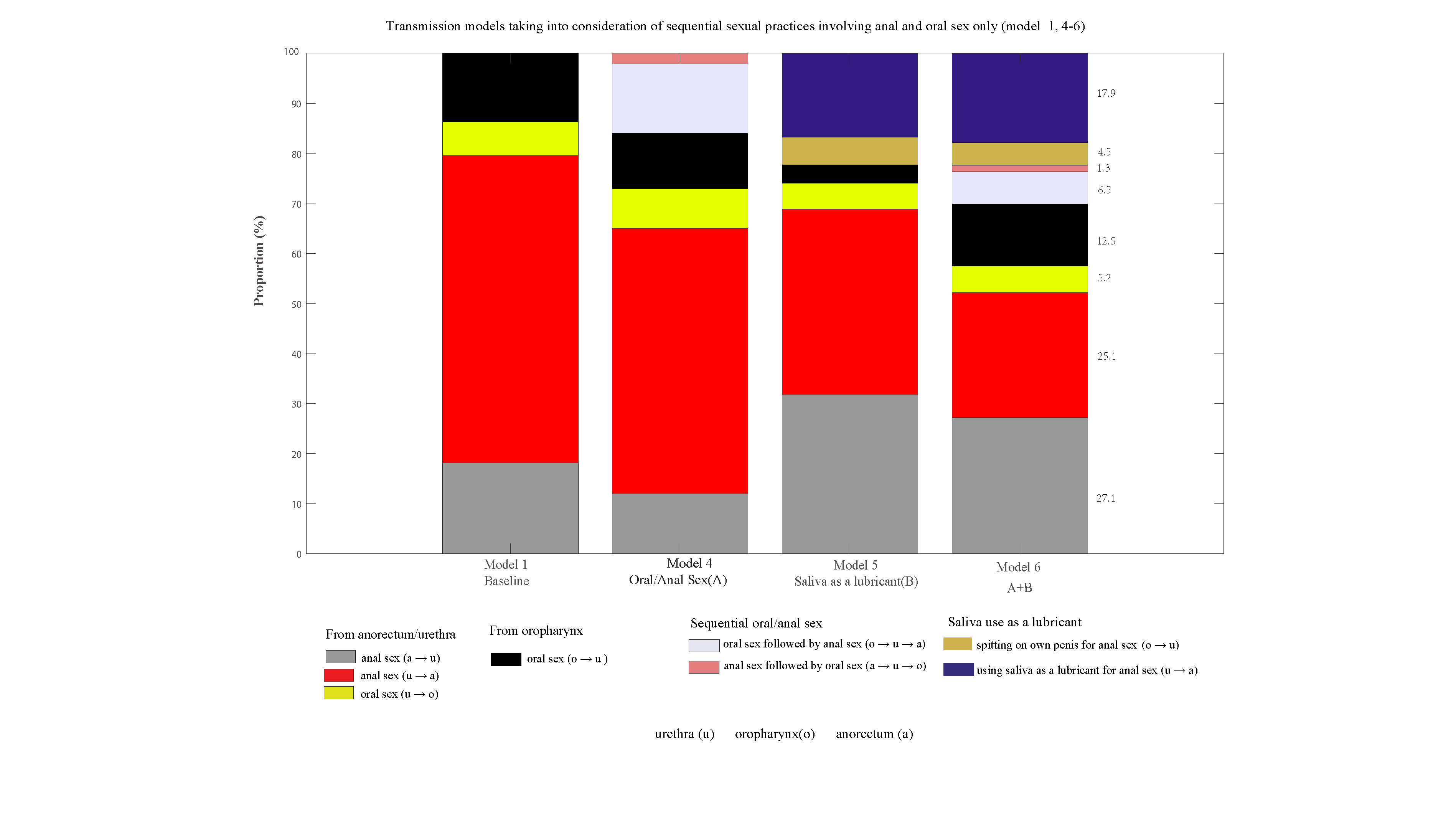


**Figure S9c.** Estimated proportion of incident *Chlamydia trachomatis* cases caused by sexual practices in MSM from the four models (model 1, 4-6) among 1,011 asymptomatic MSM attending MSHC during 2016–2017

**Validation of Results (Dataset 2): Published validation data from 393MSM attending STD & HIV care clinics in the USA**


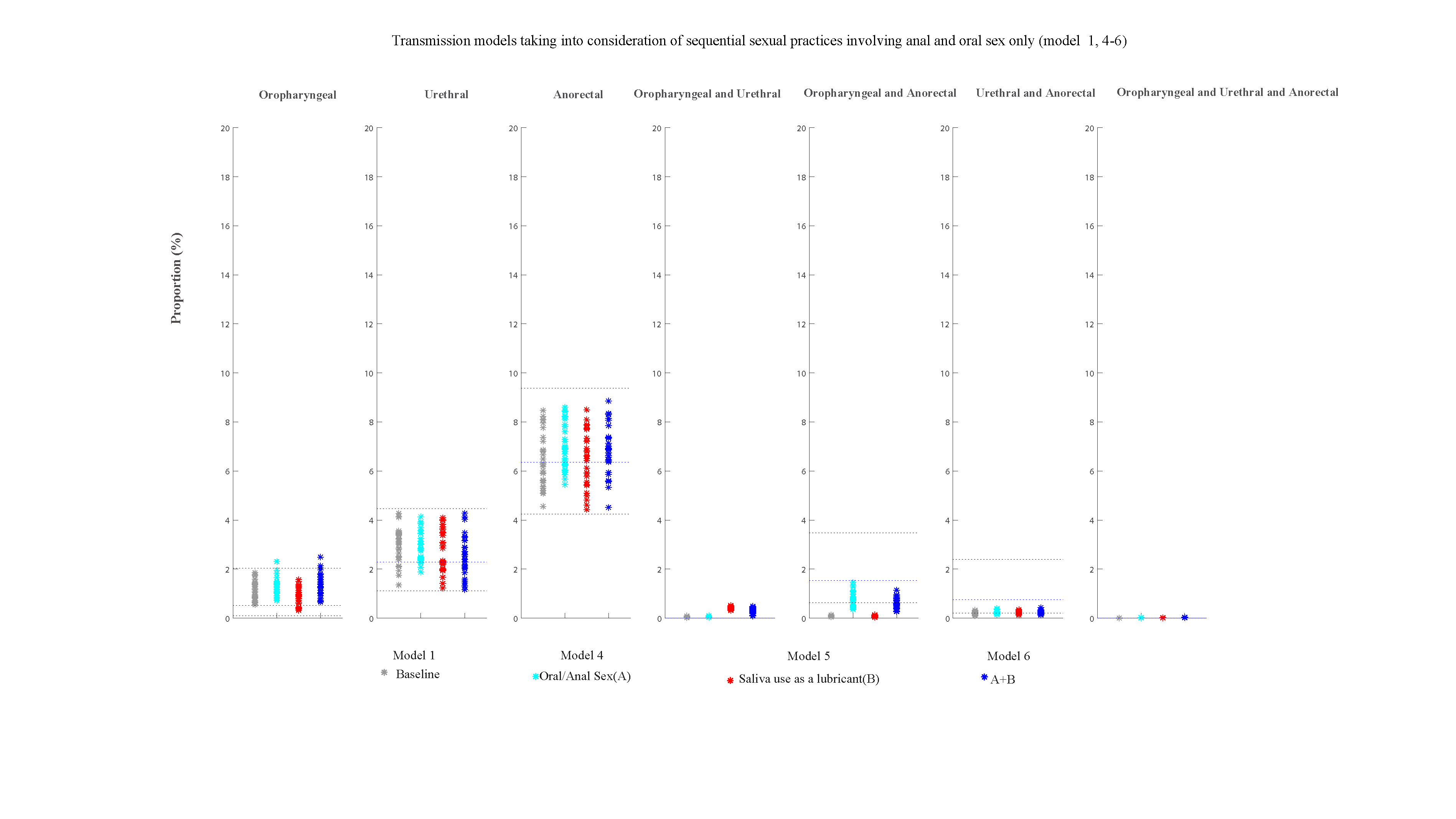


**Figure S10a.** Estimates of the eight models for the percentage of specific anatomical sites positive for *Chlamydia trachomatis* for the four models (model 1, 4-6) and the 95% confidence intervals for the observed site-specific positivity among 393MSM attending STD & HIV care clinics in the USA


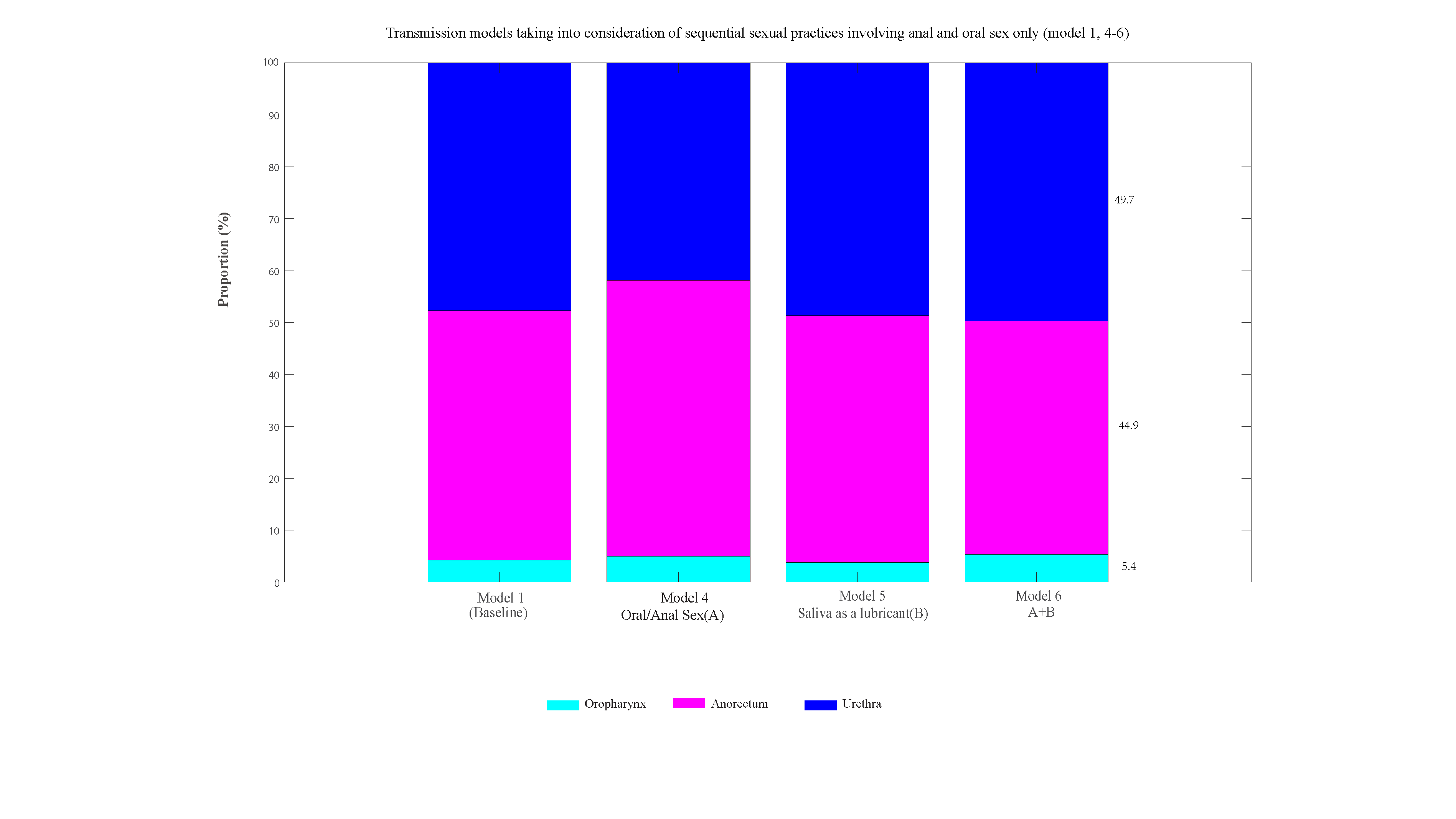


**Figure S10b.** Estimated proportion of incident *Chlamydia trachomatis* cases that occur at the oropharynx, anorectum or urethra in MSM from the four models (model 1, 4-6) among 393MSM attending STD & HIV care clinics in the USA


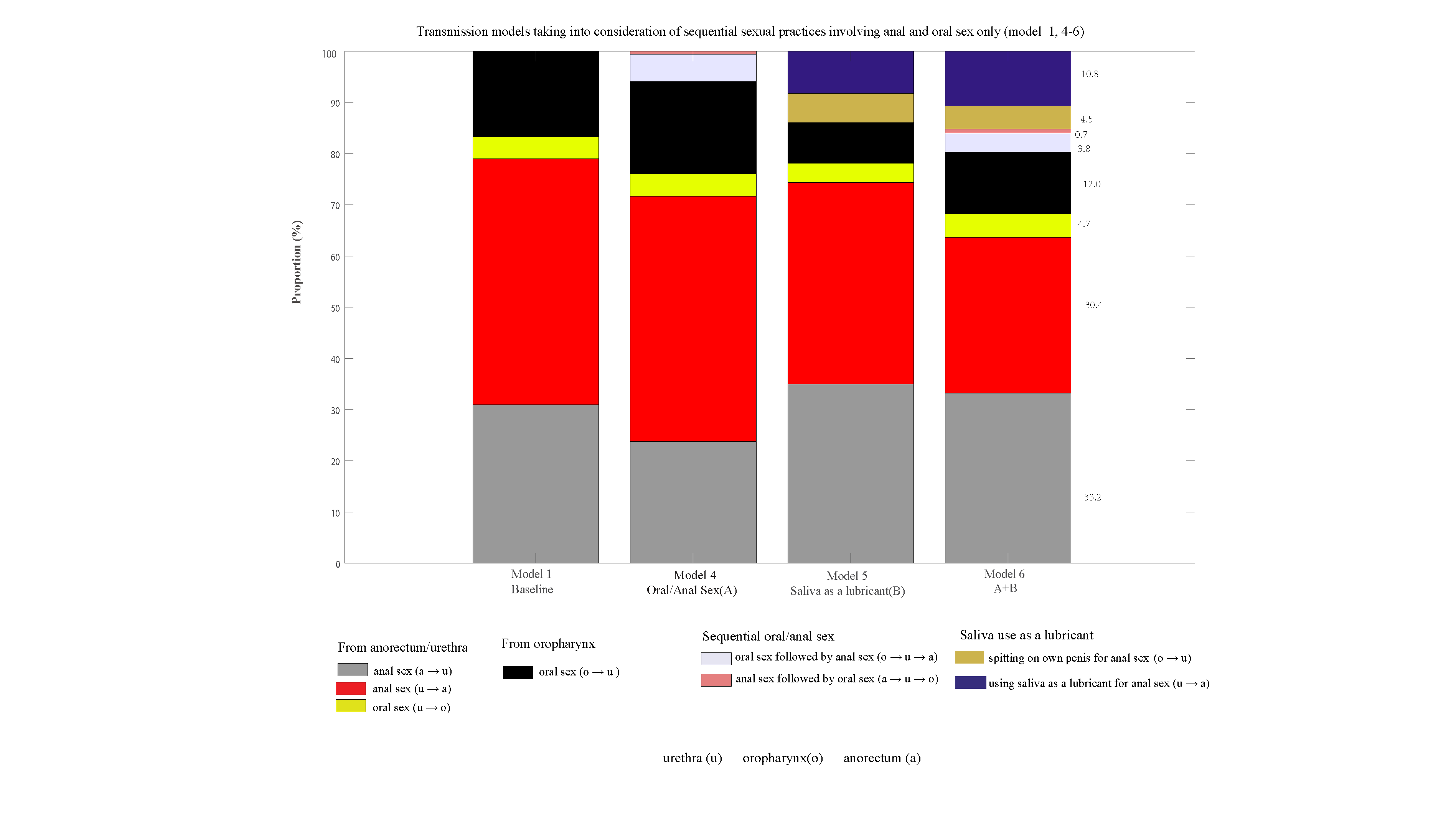


**Figure S10c.** Estimated proportion of incident *Chlamydia trachomatis* cases caused by sexual practices in MSM from the four models (model 1, 4-6) among 393MSM attending STD & HIV care clinics in the USA

**Validation of Results (Dataset 3): Published validation data from MSM surveillance data of all Dutch STI clinics**

**
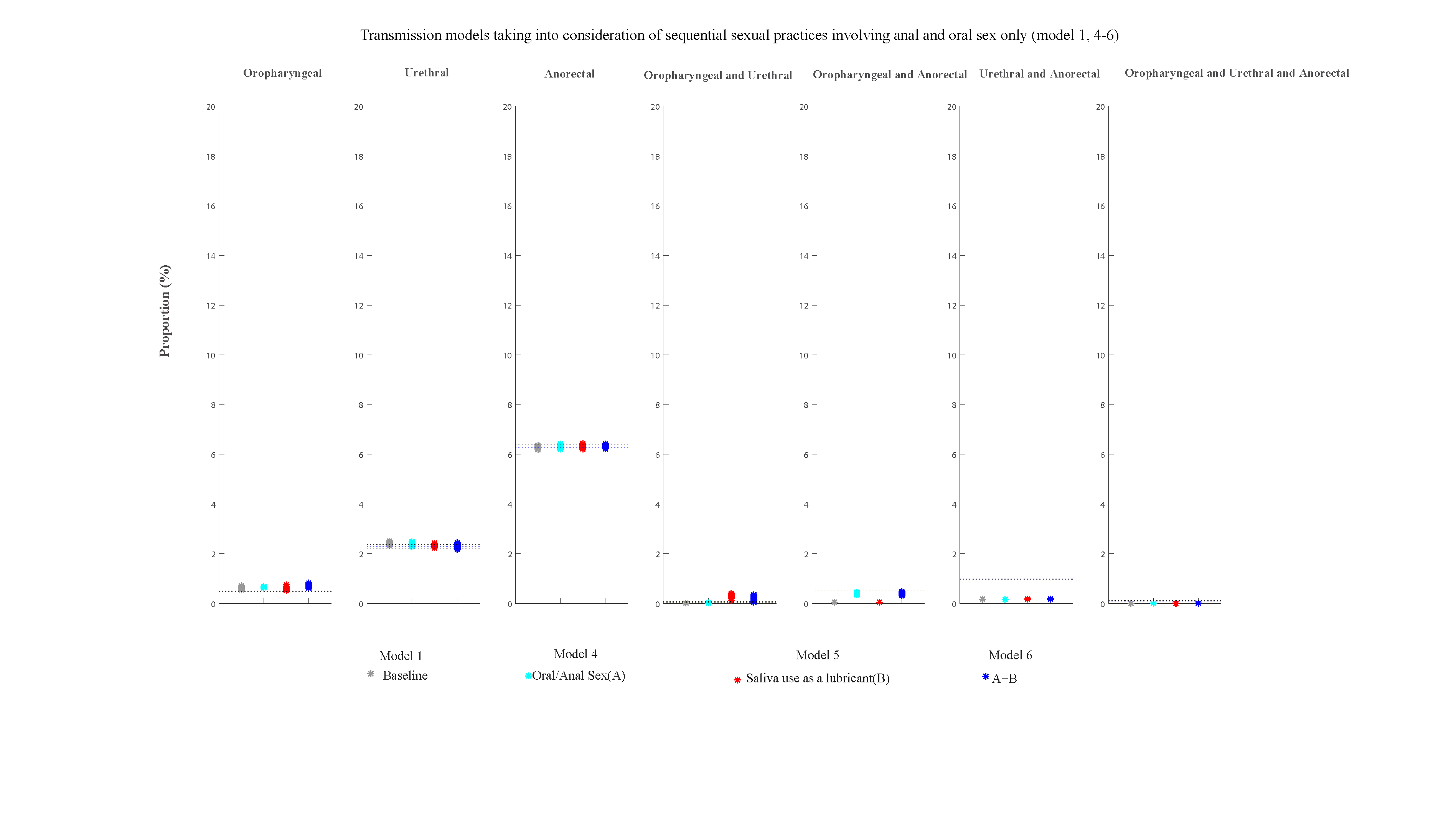
**

**Figure S11a.** Estimates of the eight models for the percentage of specific anatomical sites positive for *Chlamydia trachomatis* for the four models (model 1, 4-6) and the 95% confidence intervals for the observed site-specific positivity among MSM surveillance data (271, 242 consultations) from all Dutch STI clinics during 2008-2017

**
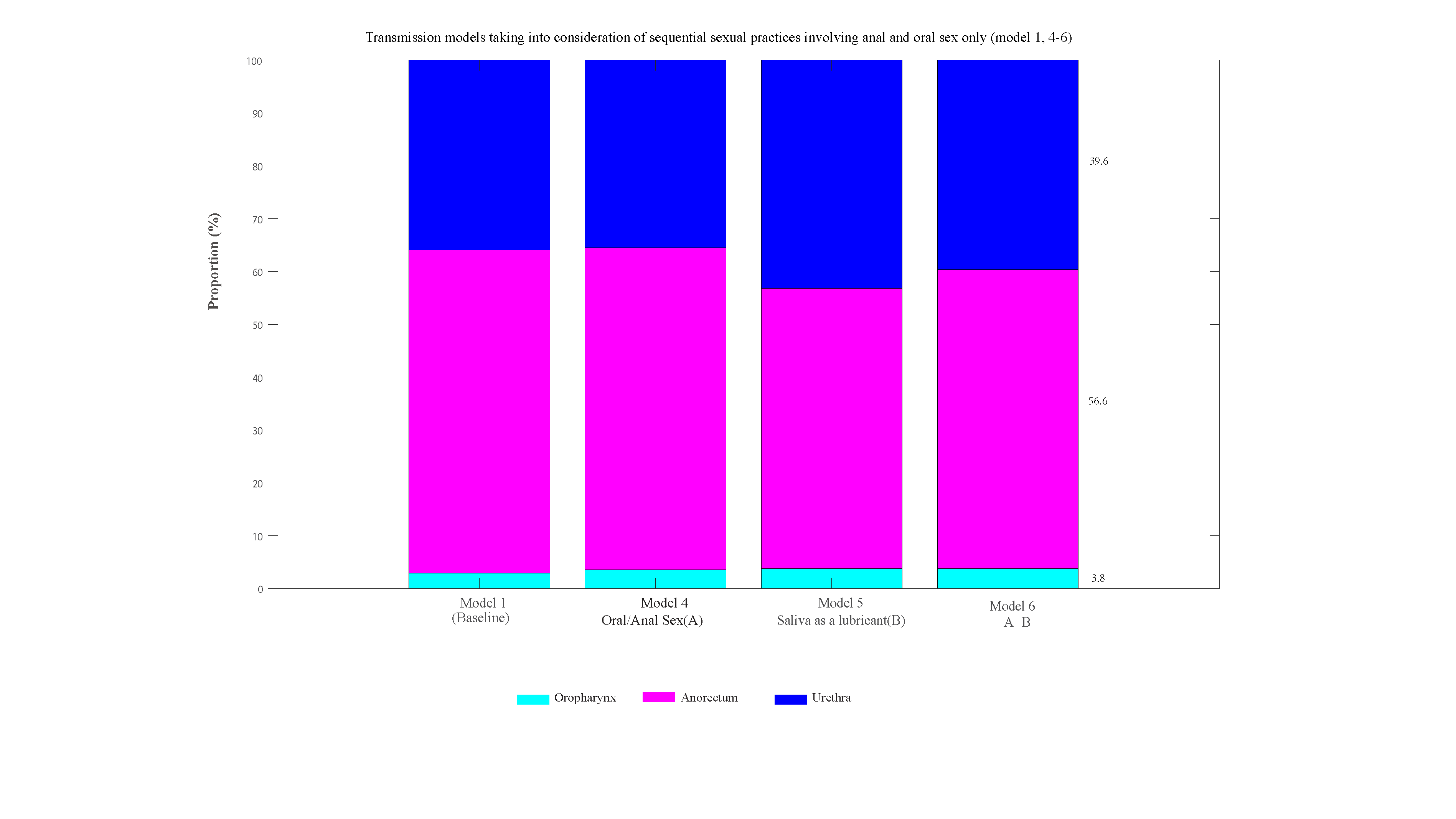
**

**Figure S11b.** Estimated proportion of incident *Chlamydia trachomatis* cases that occur at the oropharynx, anorectum or urethra in MSM from the four models (model 1, 4-6) among MSM surveillance data (271, 242 consultations) from all Dutch STI clinics during 2008-2017

**
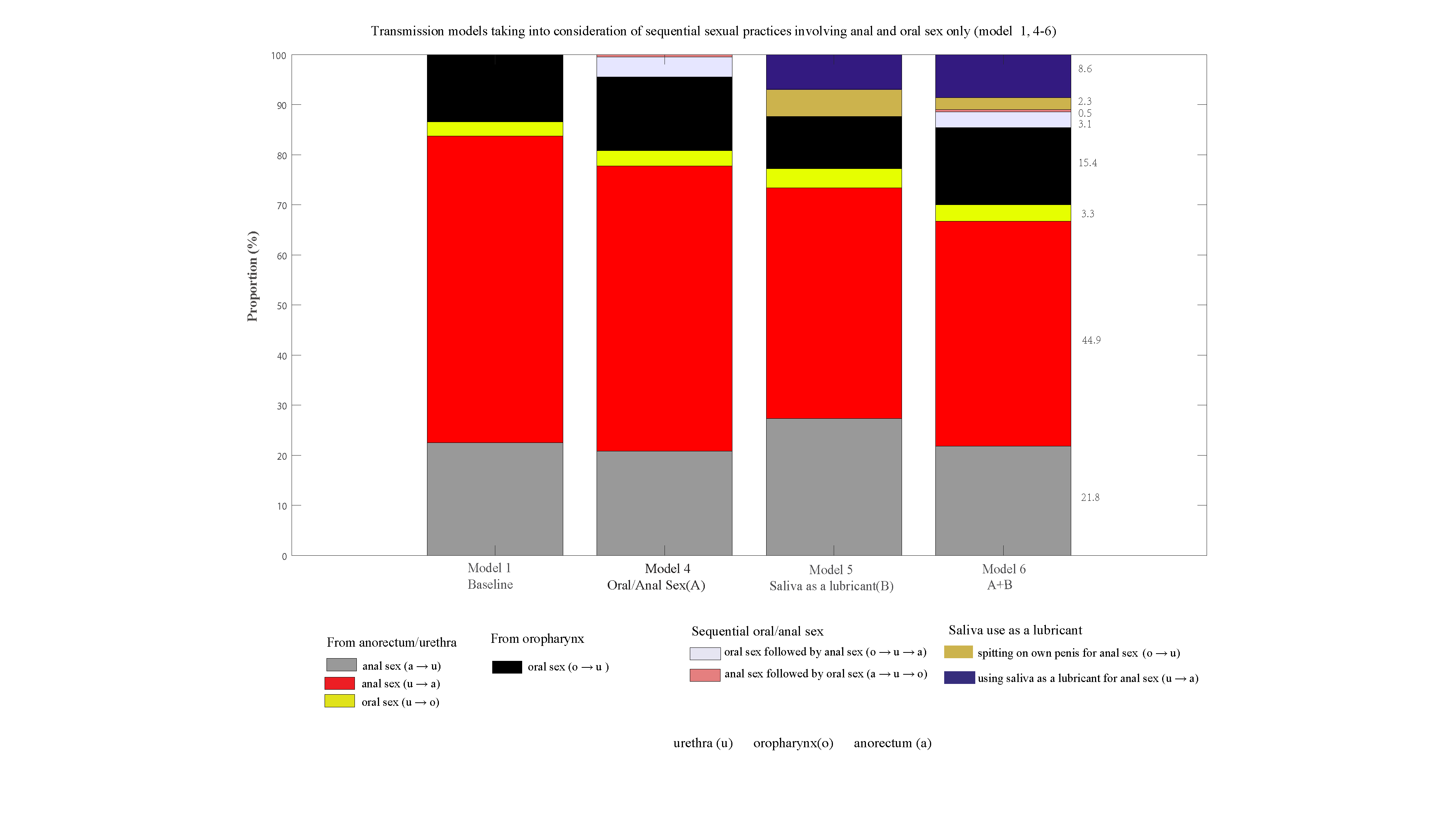
**

**Figure S11c.** Estimated proportion of incident *Chlamydia trachomatis* cases caused by sexual practices in MSM from the four models (model 1, 4-6) among MSM surveillance data (271, 242 consultations) from all Dutch STI clinics during 2008-2017

**Validation of Results (Dataset 4): Published validation data from 1,610 community MSM in Thailand**


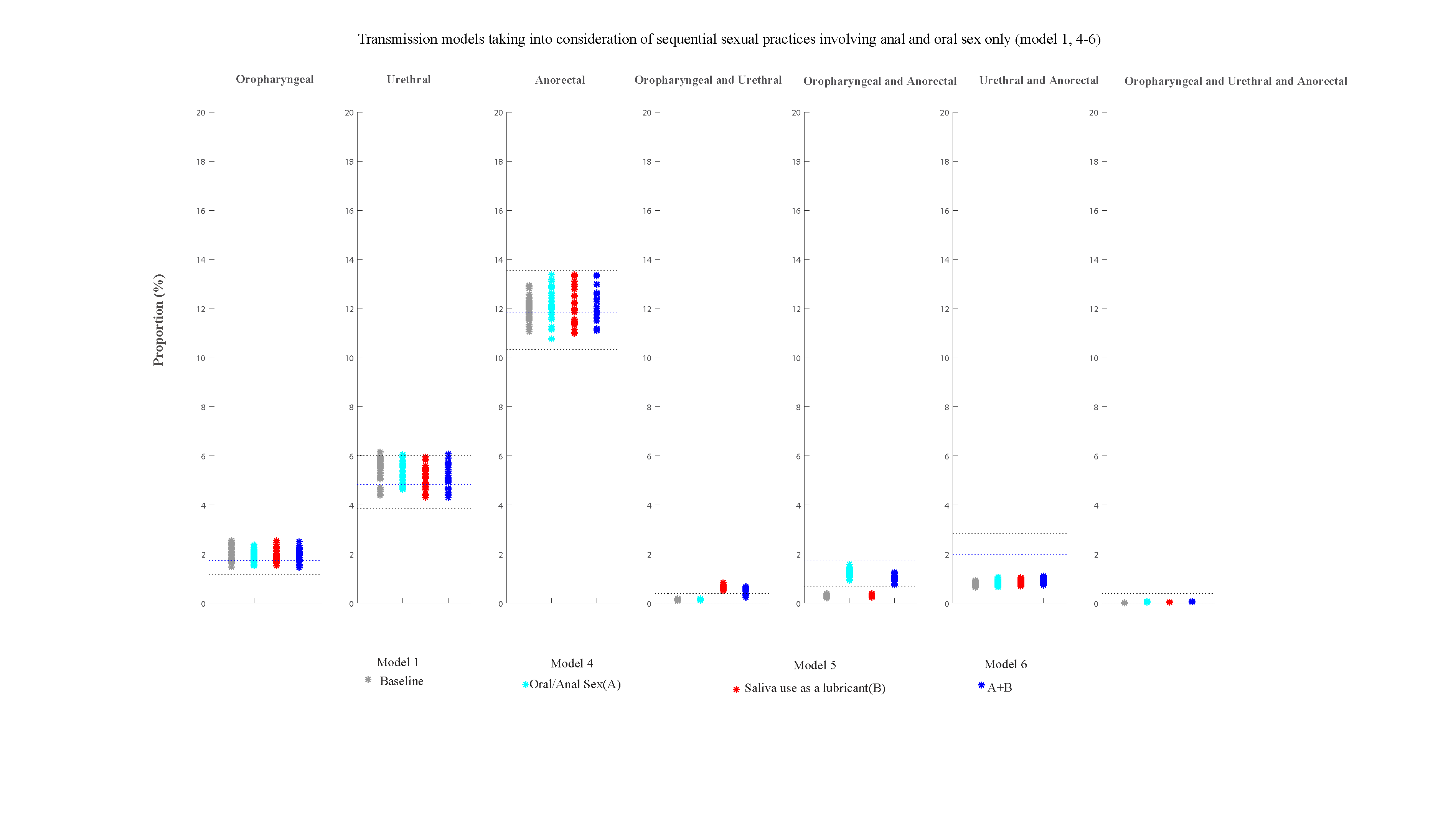


**Figure S12a.** Estimates of the eight models for the percentage of specific anatomical sites positive for *Chlamydia trachomatis* for the four models (model 1, 4-6) and the 95% confidence intervals for the observed site-specific positivity among 1,610 MSM attending a community-led test and treat cohort in Thailand between October 2015 and October 2016


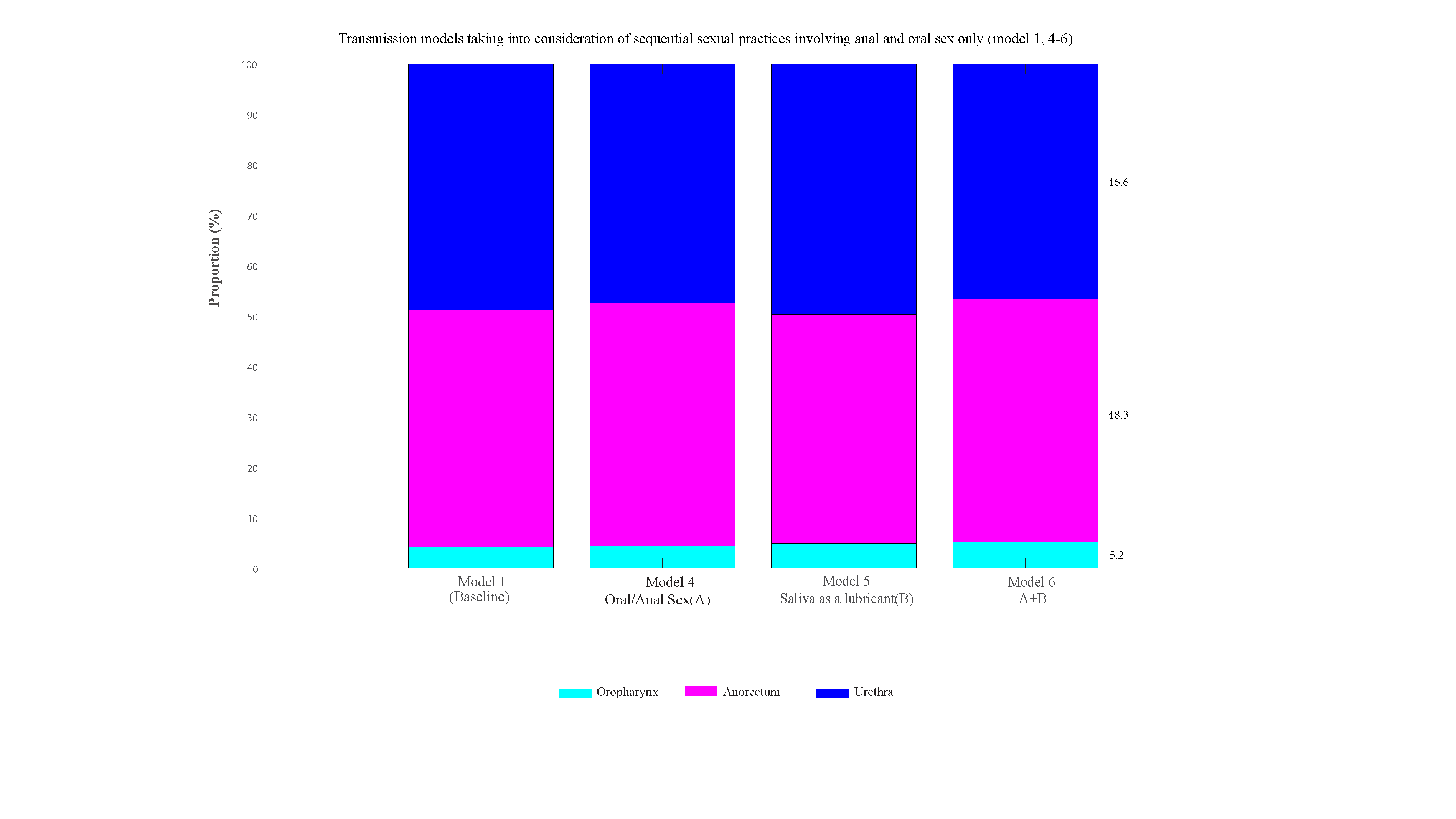


**Figure S12b.** Estimated proportion of incident *Chlamydia trachomatis* cases that occur at the oropharynx, anorectum or urethra in MSM from the four models (model 1, 4-6) among 1,610 MSM attending a community-led test and treat cohort in Thailand between October 2015 and October 2016


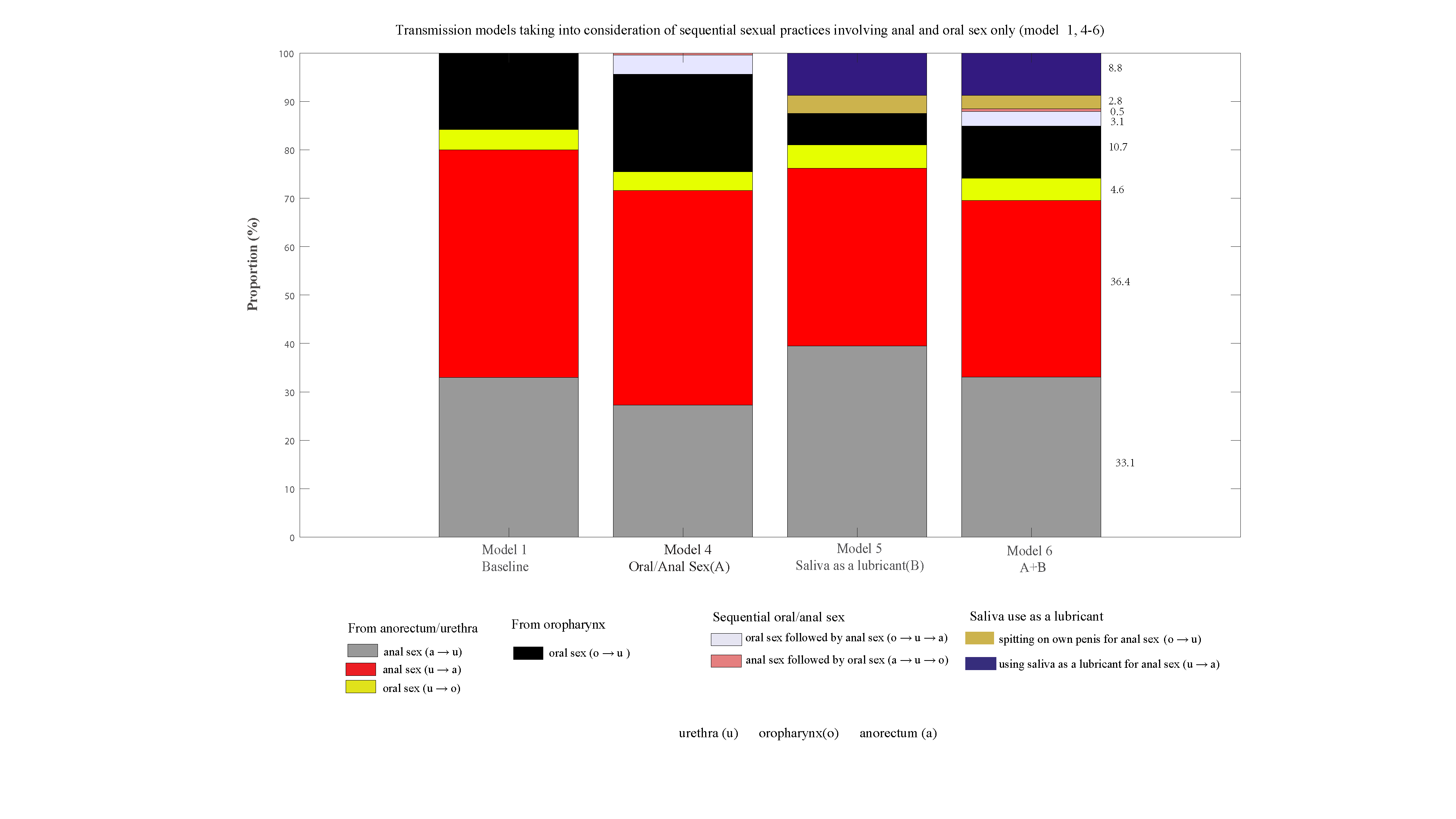


**Figure S12c**. Estimated proportion of incident *Chlamydia trachomatis* cases caused by sexual practices in MSM from the four models (model 1, 4-6) among 1,610 MSM attending a community-led test and treat cohort in Thailand between October 2015 and October 2016

**Validation of Results (Dataset 5): Published validation data from 179 MSM with HIV in the USA**


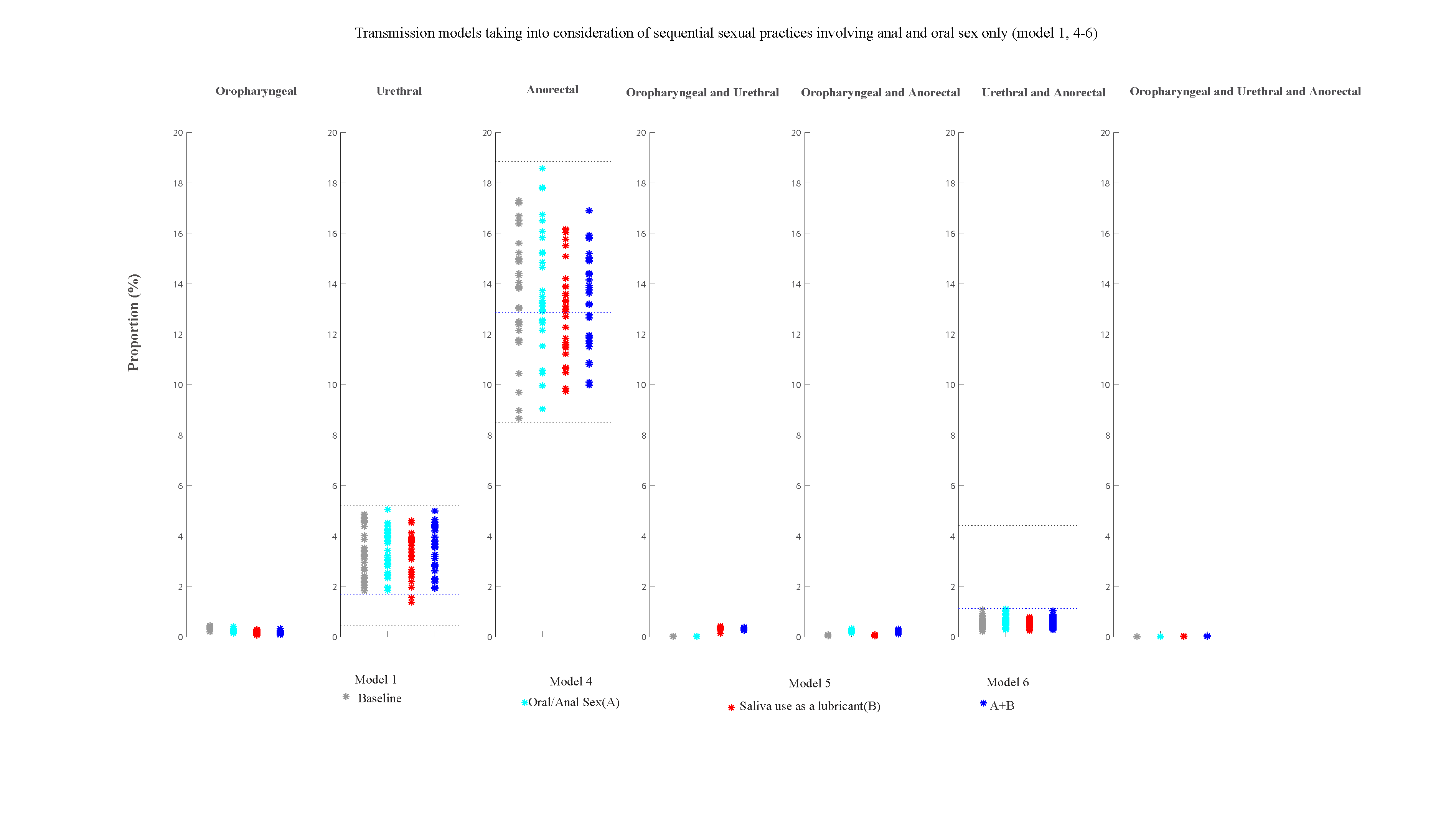


**Figure S13a.** Estimates of the eight models for the percentage of specific anatomical sites positive for *Chlamydia trachomatis* for the four models (model 1, 4-6) and the 95% confidence intervals for the observed site-specific positivity among179 MSM with HIV


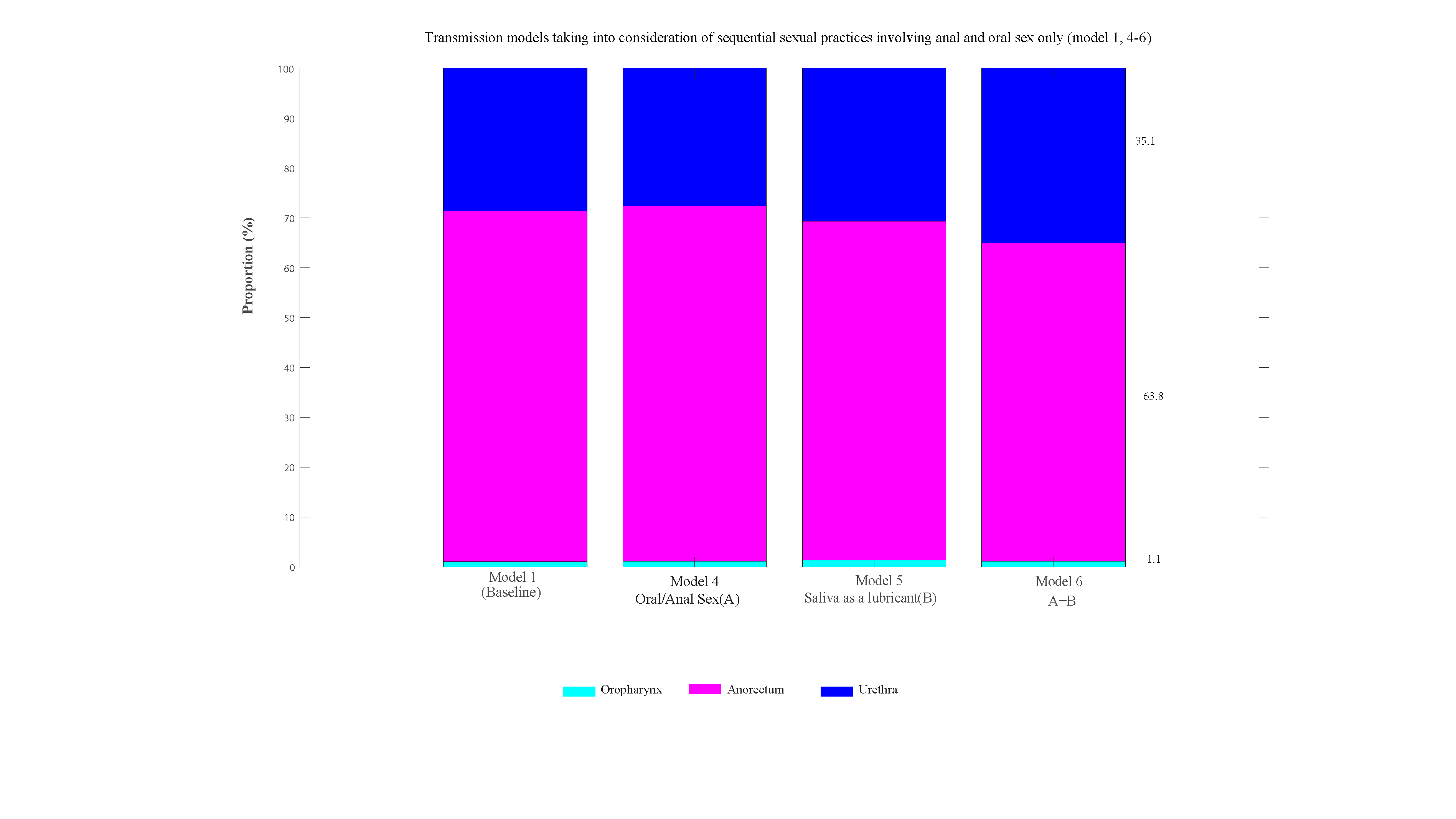


**Figure S13b**. Estimated proportion of incident *Chlamydia trachomatis* cases that occur at the oropharynx, anorectum or urethra in MSM from the four models (model 1, 4-6) among179 MSM with HIV


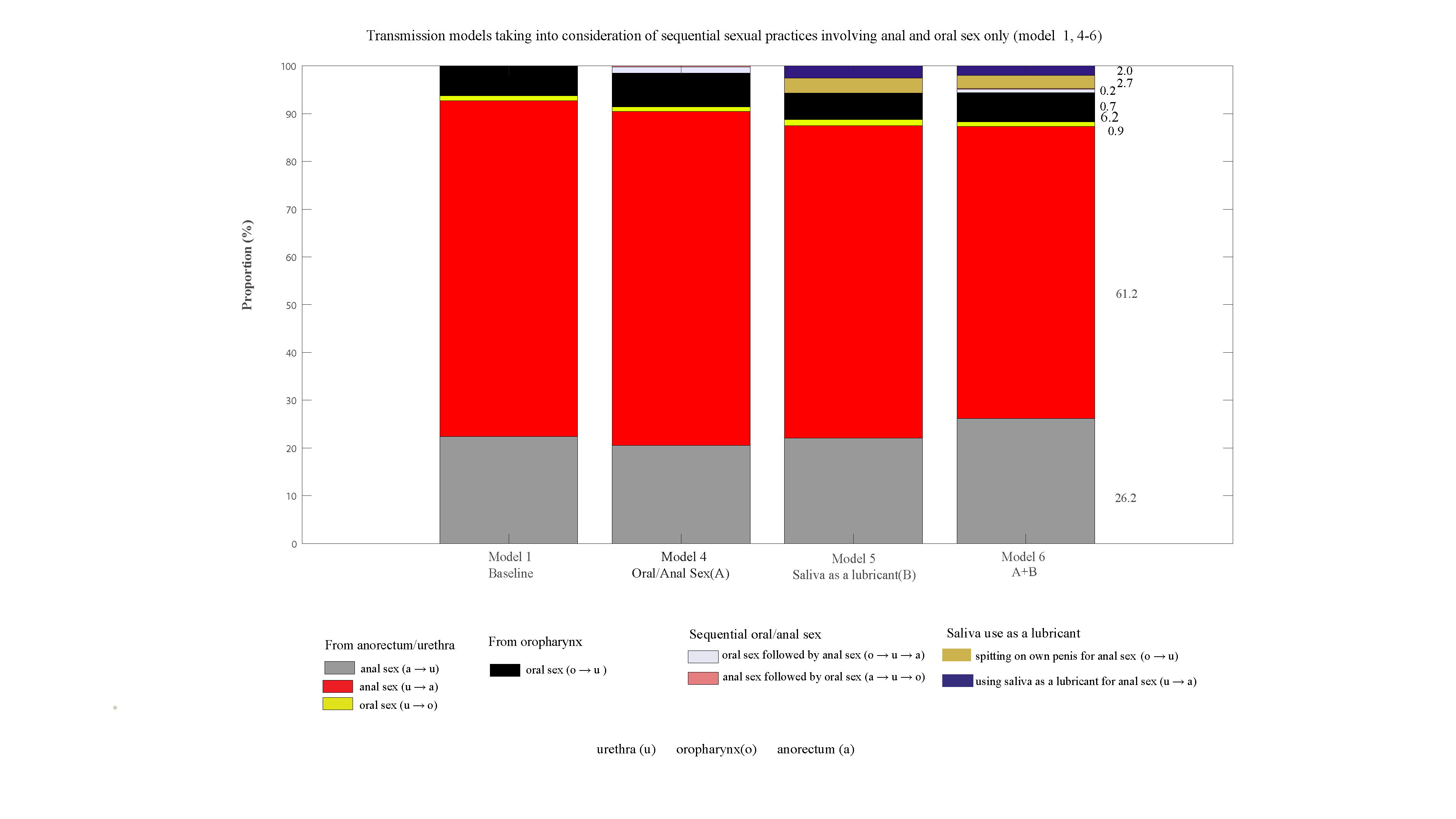


**Figure S13c.** Estimated proportion of incident *Chlamydia trachomatis* cases caused by sexual practices in MSM from the four models (model 1, 4-6) among179 MSM with HIV

**Supplementary results: ‘Anal sex, oral sex, rimming and sequential sexual practices’ transmission models (Model 2, 7-13)**

**Unpublished data from 4888 MSM attending Melbourne Sexual Health Centre for model (model 2, 7-13) calibration**


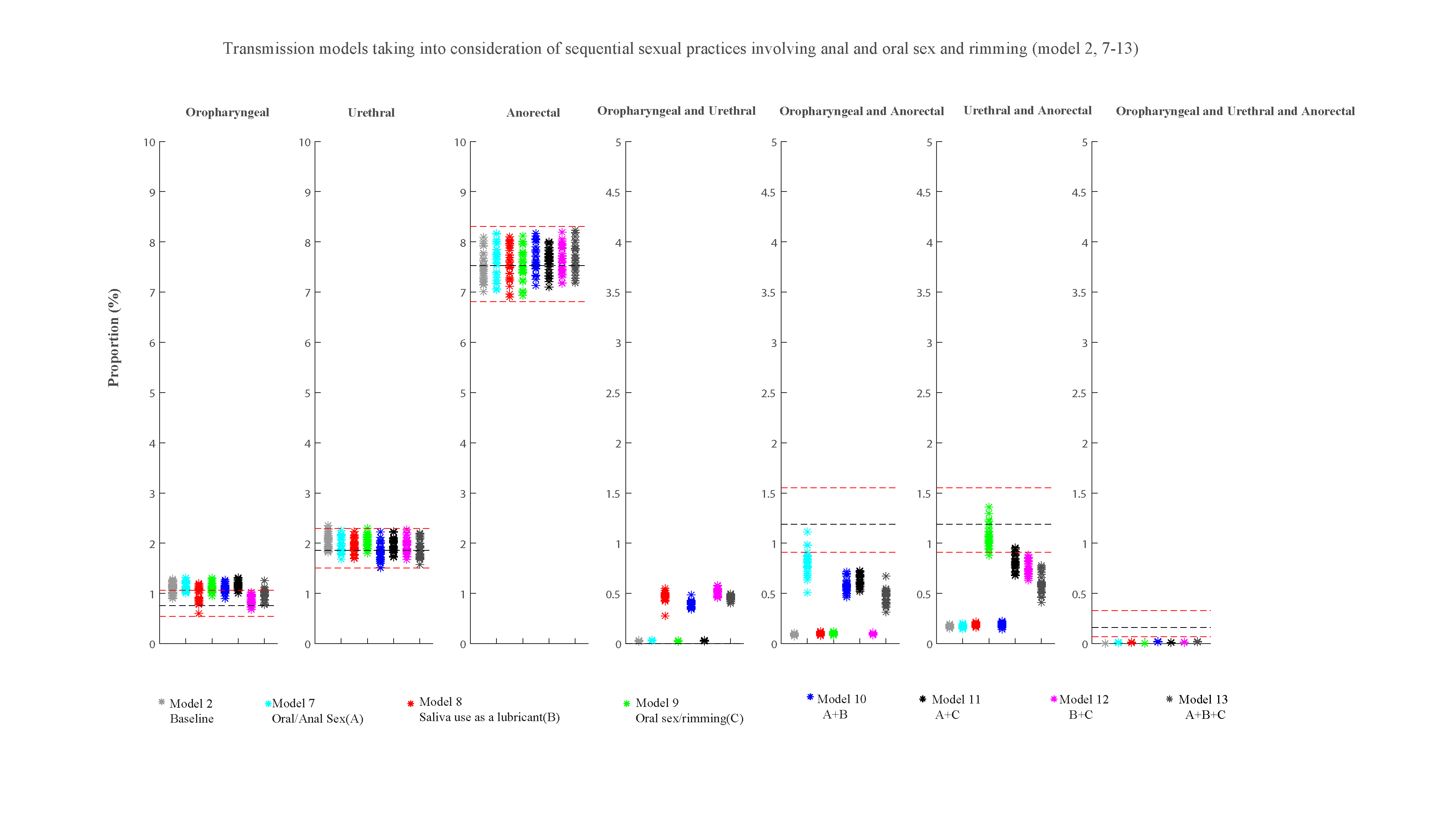


**Figure S14a.** Estimates of the eight models for the percentage of specific anatomical sites positive for *Chlamydia trachomatis* for the 8 models (model 2, 7-13) and the 95% confidence intervals for the observed site-specific positivity among 4888 MSM attending Melbourne Sexual Health Centre in 2018 and 2019


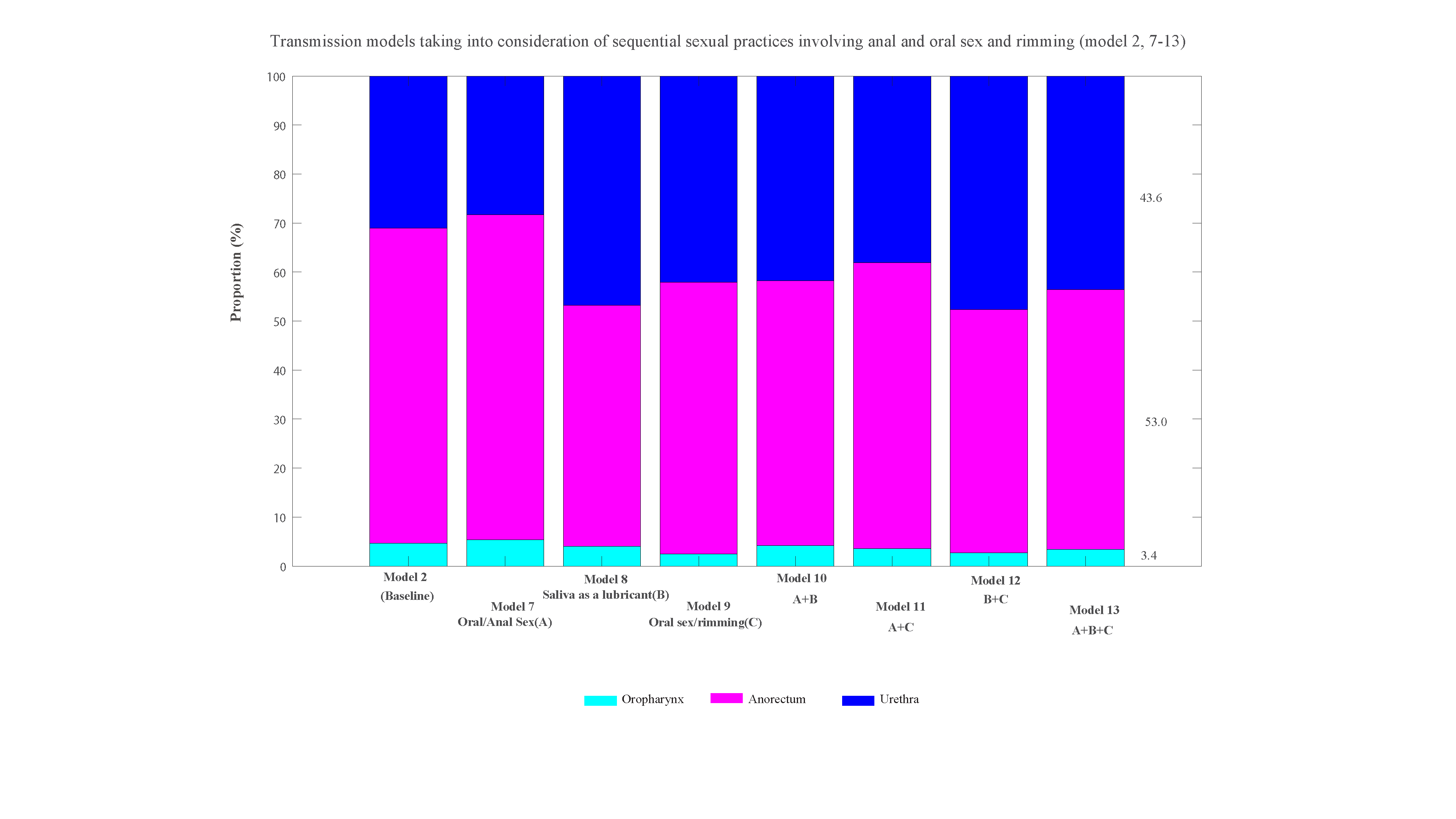


**Figure S14b.** Estimated proportion of incident *Chlamydia trachomatis* cases that occur at the oropharynx, anorectum or urethra in MSM from the eight models (model 2, 7-13) among 4888 MSM attending Melbourne Sexual Health Centre in 2018 and 2019


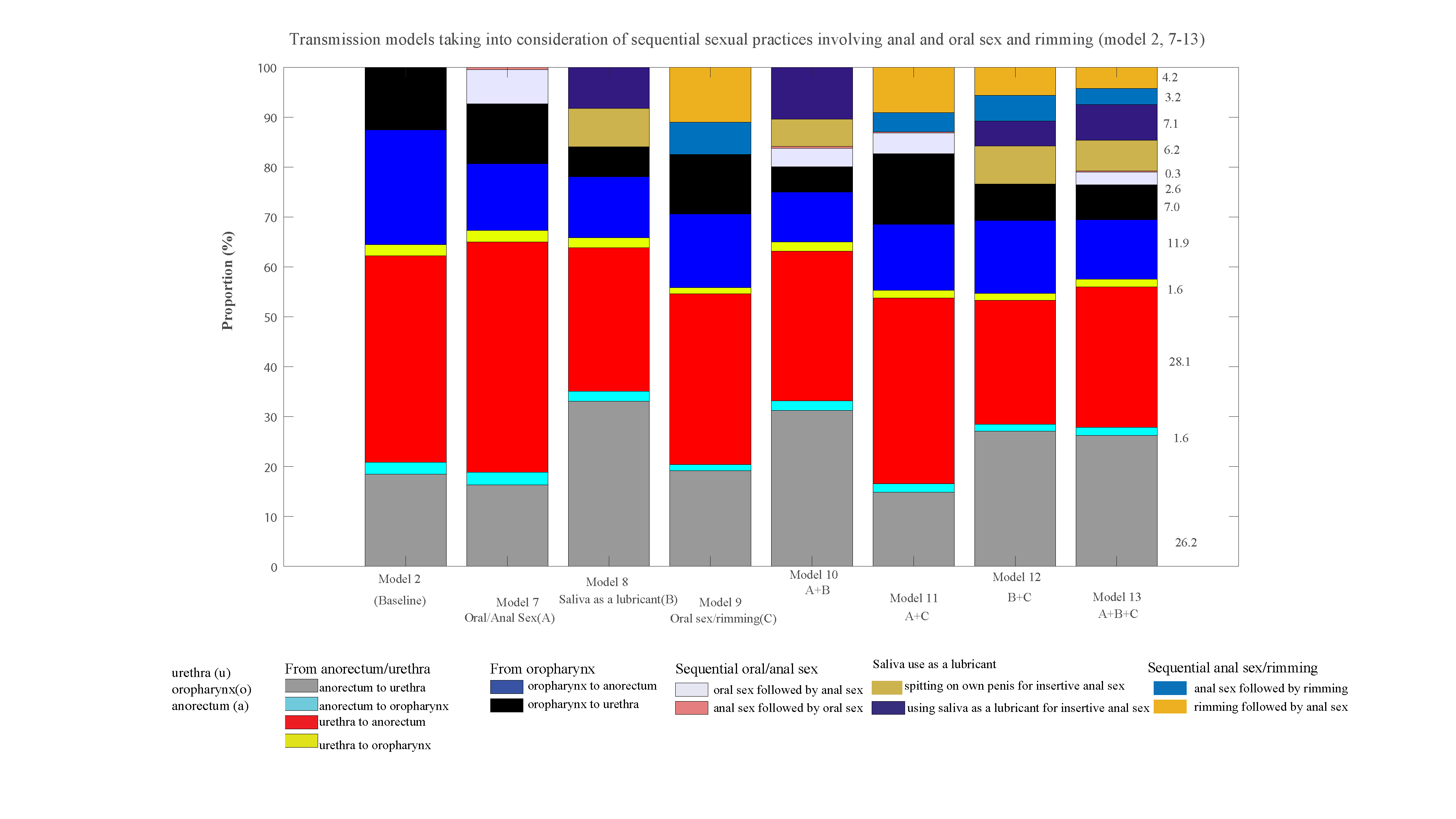


**Figure S14c.** Estimated proportion of incident *Chlamydia trachomatis* cases caused by sexual practices in MSM from the eight models (model 2, 7-13) among 4888 MSM attending Melbourne Sexual Health Centre in 2018 and 2019

**Validation of Results (Dataset 1): Published validation data from 1,011 asymptomatic MSM attending Melbourne Sexual Health Centre**

**
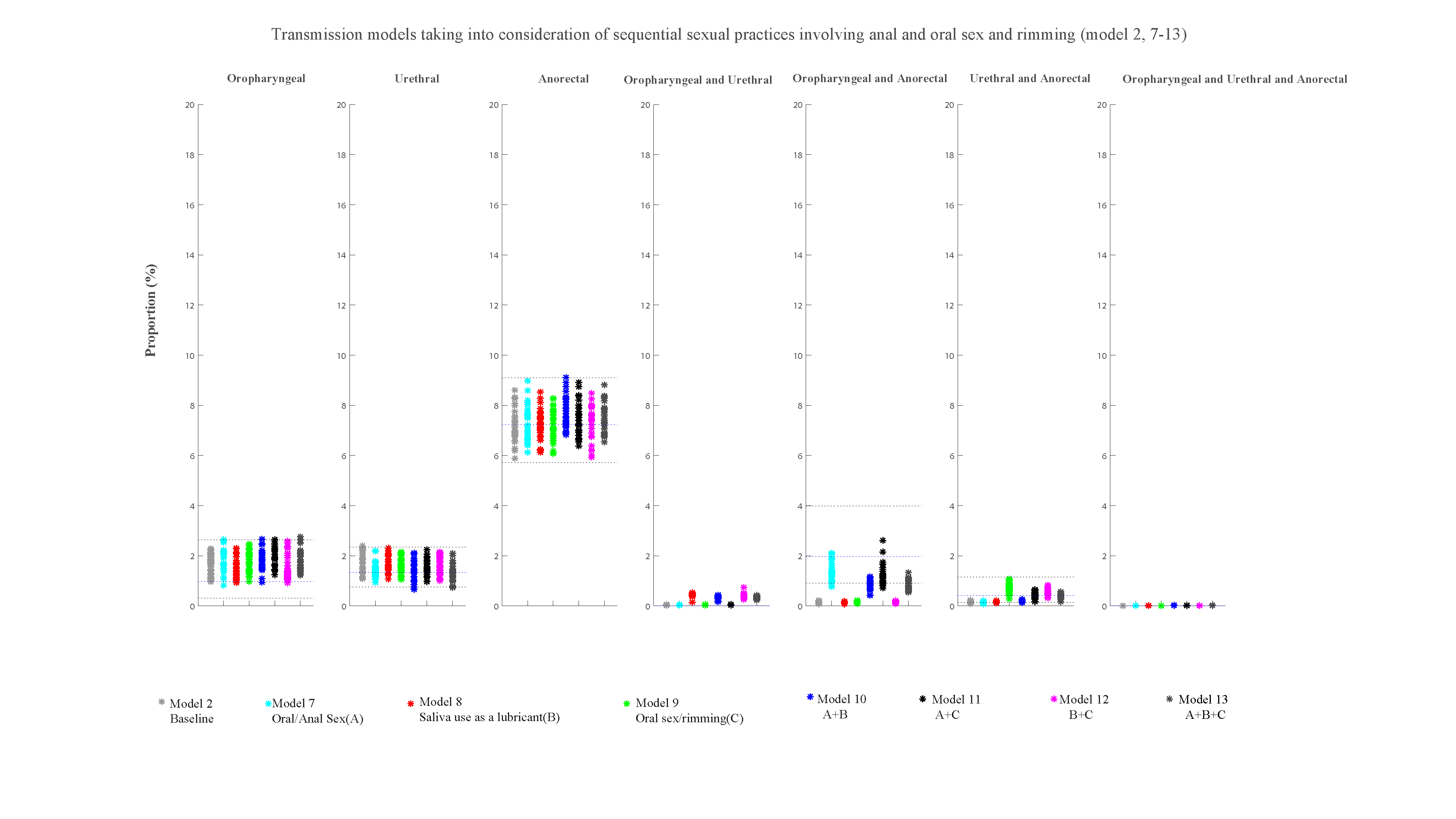
**

**Figure S15a.** Estimates of the eight models for the percentage of specific anatomical sites positive for *Chlamydia trachomatis* for the 8 models (model 2, 7-13) and the 95% confidence intervals for the observed site-specific positivity among 1,011 asymptomatic MSM attending MSHC during 2016–2017

**
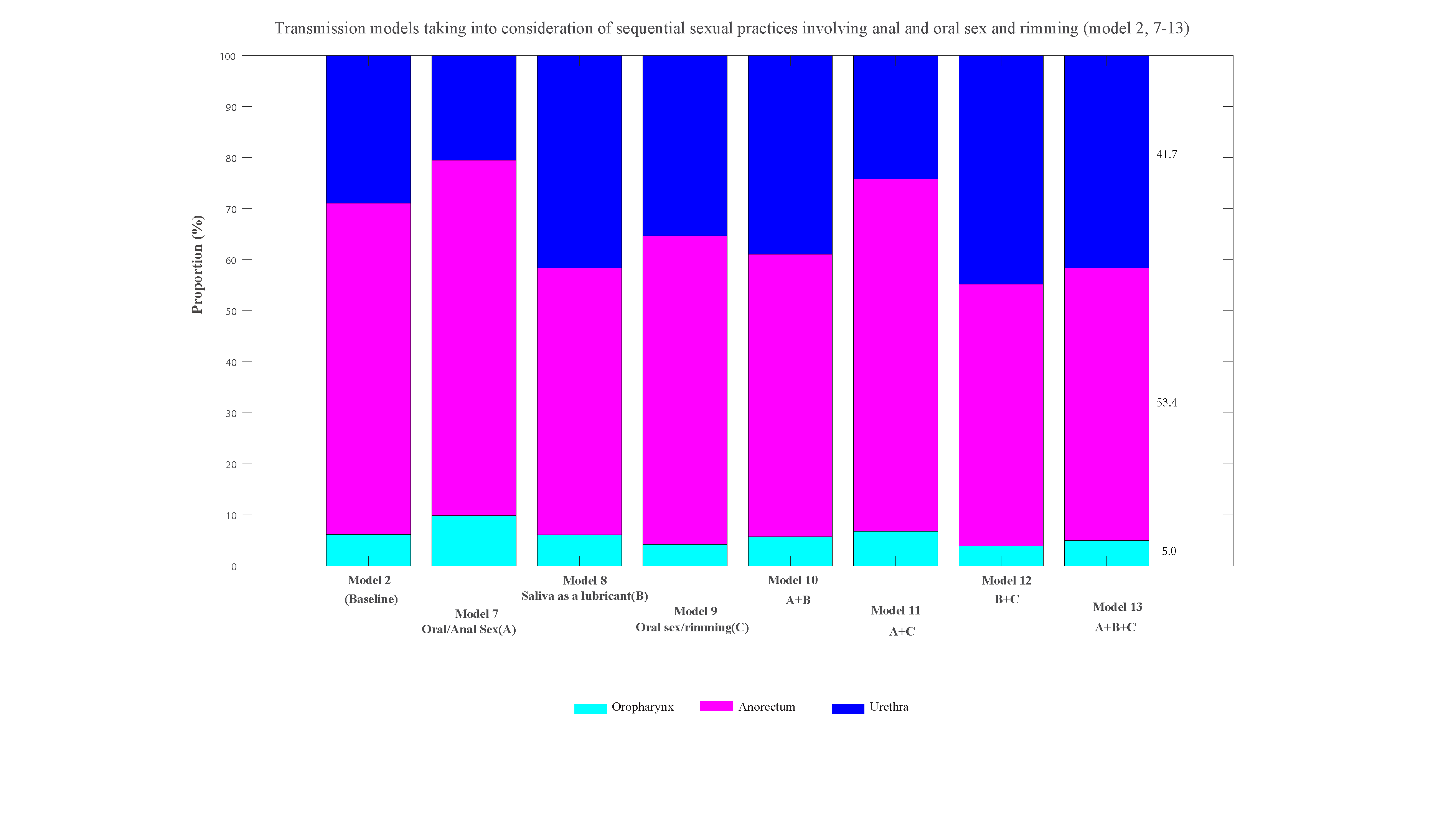
**

**Figure S15b.** Estimated proportion of incident *Chlamydia trachomatis* cases that occur at the oropharynx, anorectum or urethra in MSM from the eight models (model 2, 7-13) among 1,011 asymptomatic MSM attending MSHC during 2016–2017

**
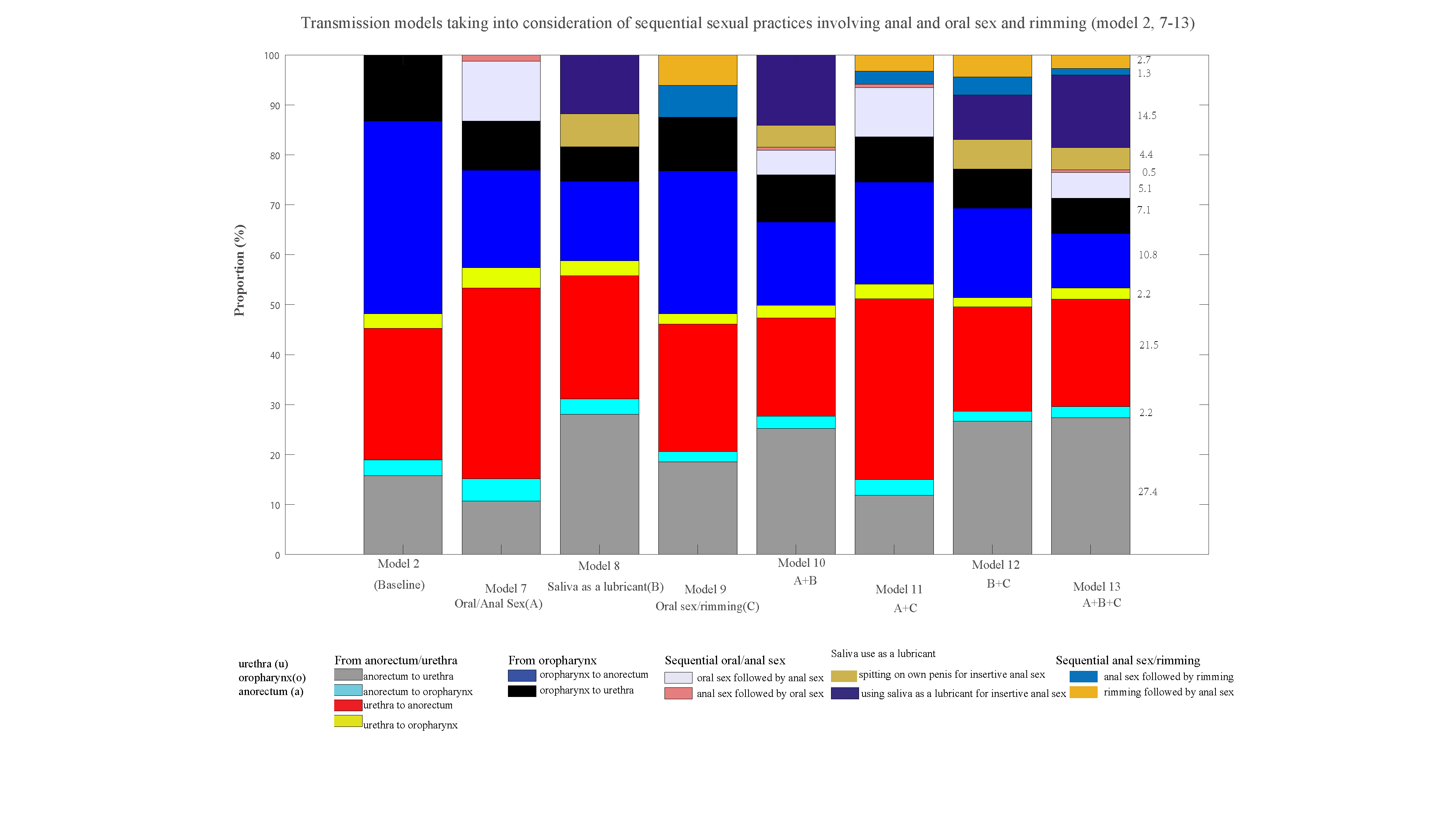
**

**Figure S15c.** Estimated proportion of incident *Chlamydia trachomatis* cases caused by sexual practices in MSM from the eight models (model 2, 7-13) among 1,011 asymptomatic MSM attending MSHC during 2016–2017

**Validation of Results (Dataset 2): Published validation data from 393MSM attending STD & HIV care clinics in the USA**

**
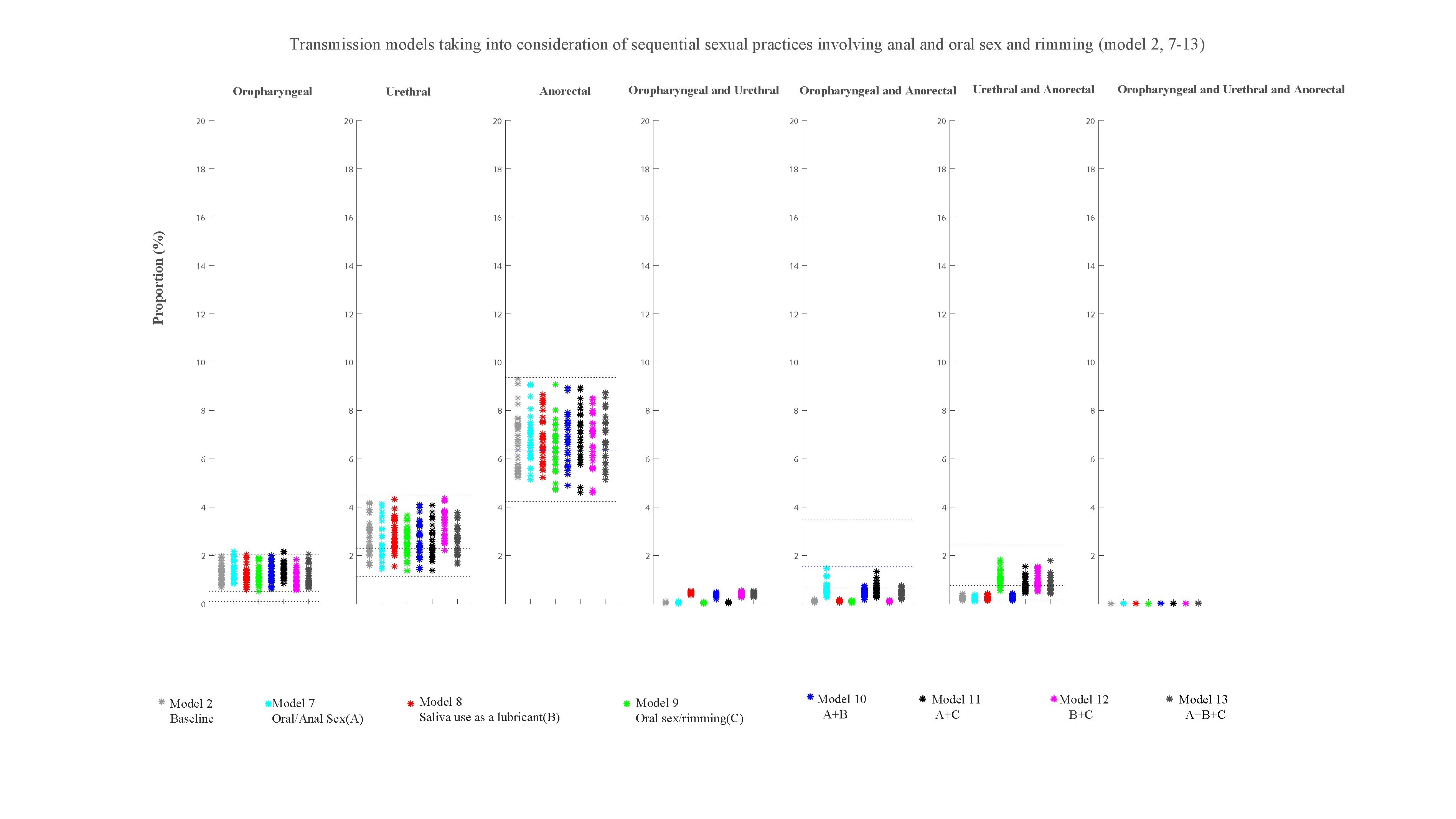
**

**Figure S16a.** Estimates of the eight models for the percentage of specific anatomical sites positive for *Chlamydia trachomatis* for the 8 models (model 2, 7-13) and the 95% confidence intervals for the observed site-specific positivity among 393MSM attending STD & HIV care clinics in the USA

**
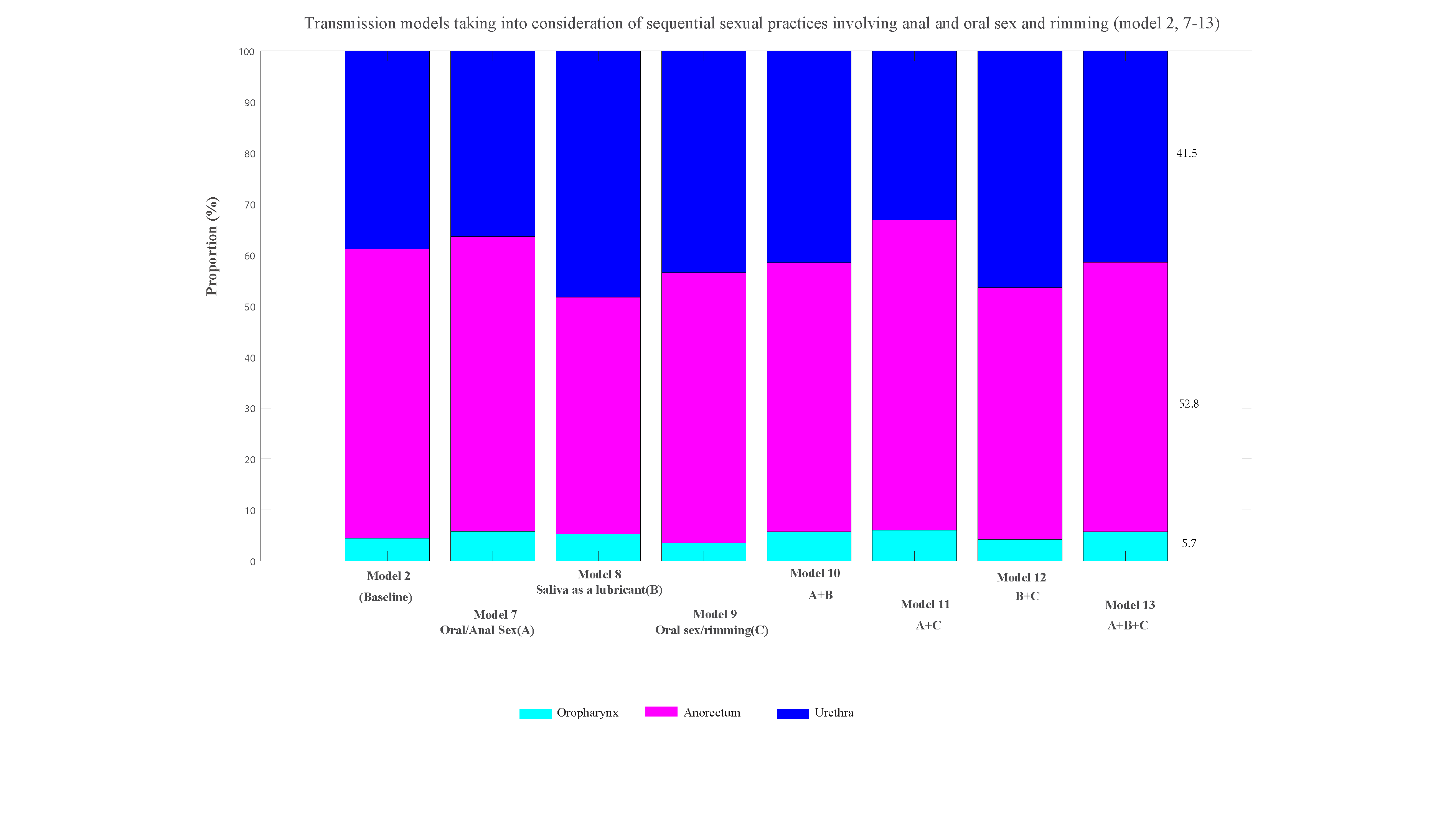
**

**Figure S16b.** Estimated proportion of incident *Chlamydia trachomatis* cases that occur at the oropharynx, anorectum or urethra in MSM from the eight models (model 2, 7-13) among 393MSM attending STD & HIV care clinics in the USA

**
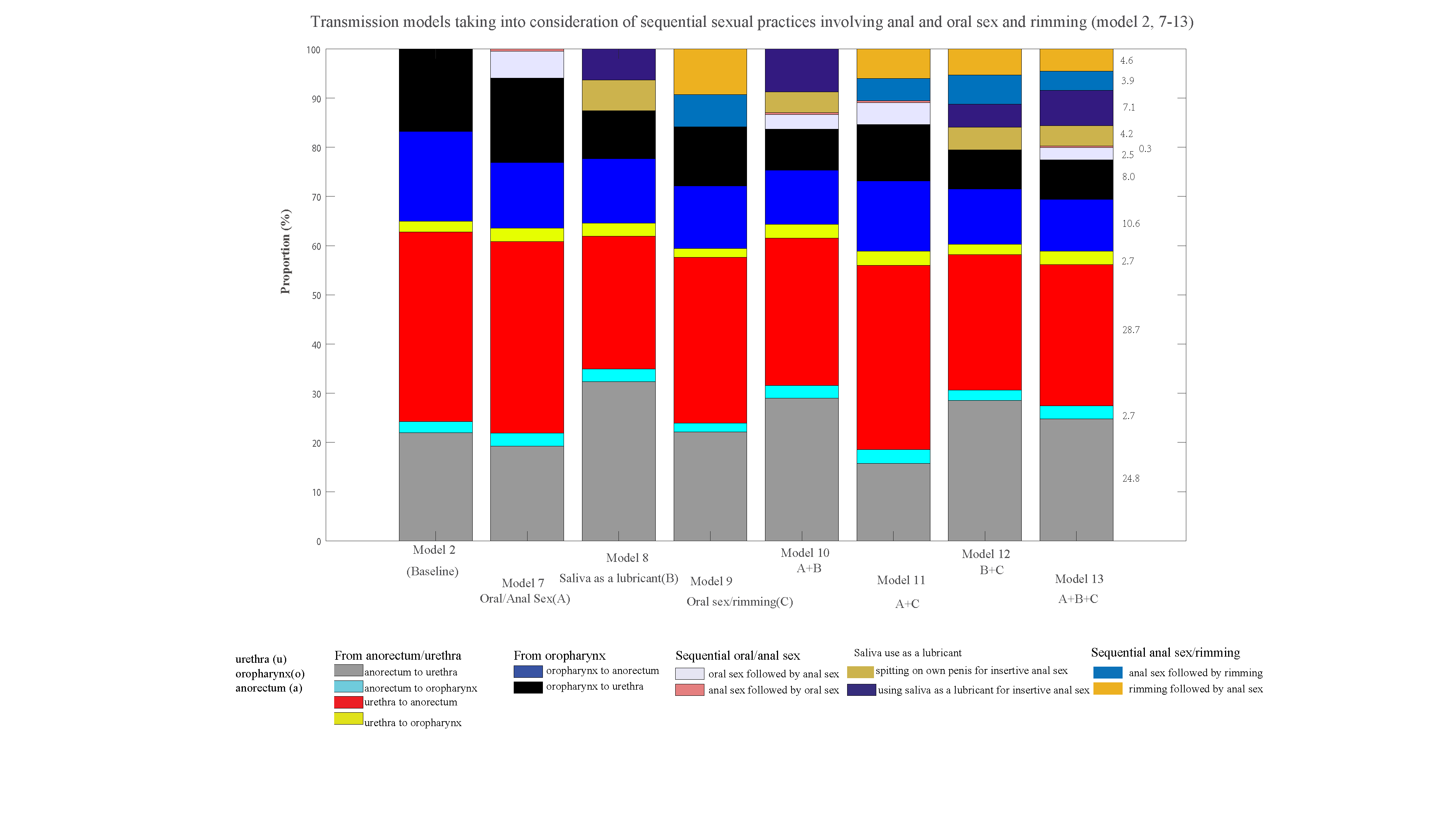
**

**Figure S16c.** Estimated proportion of incident *Chlamydia trachomatis* cases caused by sexual practices in MSM from the eight models (model 2, 7-13) among 393MSM attending STD & HIV care clinics in the USA

**Validation of Results (Dataset 3): Published validation data from MSM surveillance data of all Dutch STI clinics**


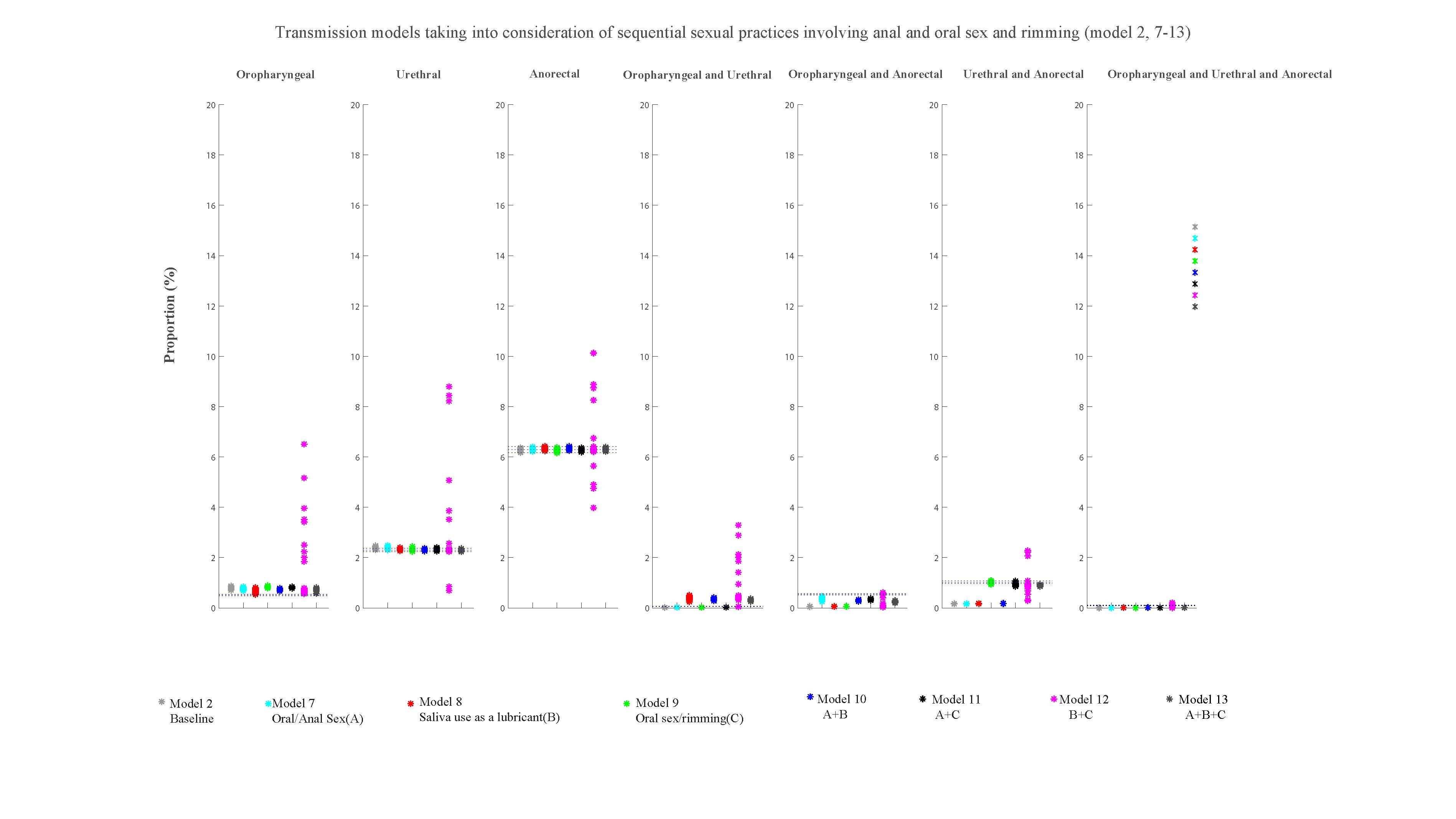


**Figure S17a.** Estimates of the eight models for the percentage of specific anatomical sites positive for *Chlamydia trachomatis* for the 8 models (model 2, 7-13) and the 95% confidence intervals for the observed site-specific positivity among MSM surveillance data (271, 242 consultations) from all Dutch STI clinics during 2008-2017


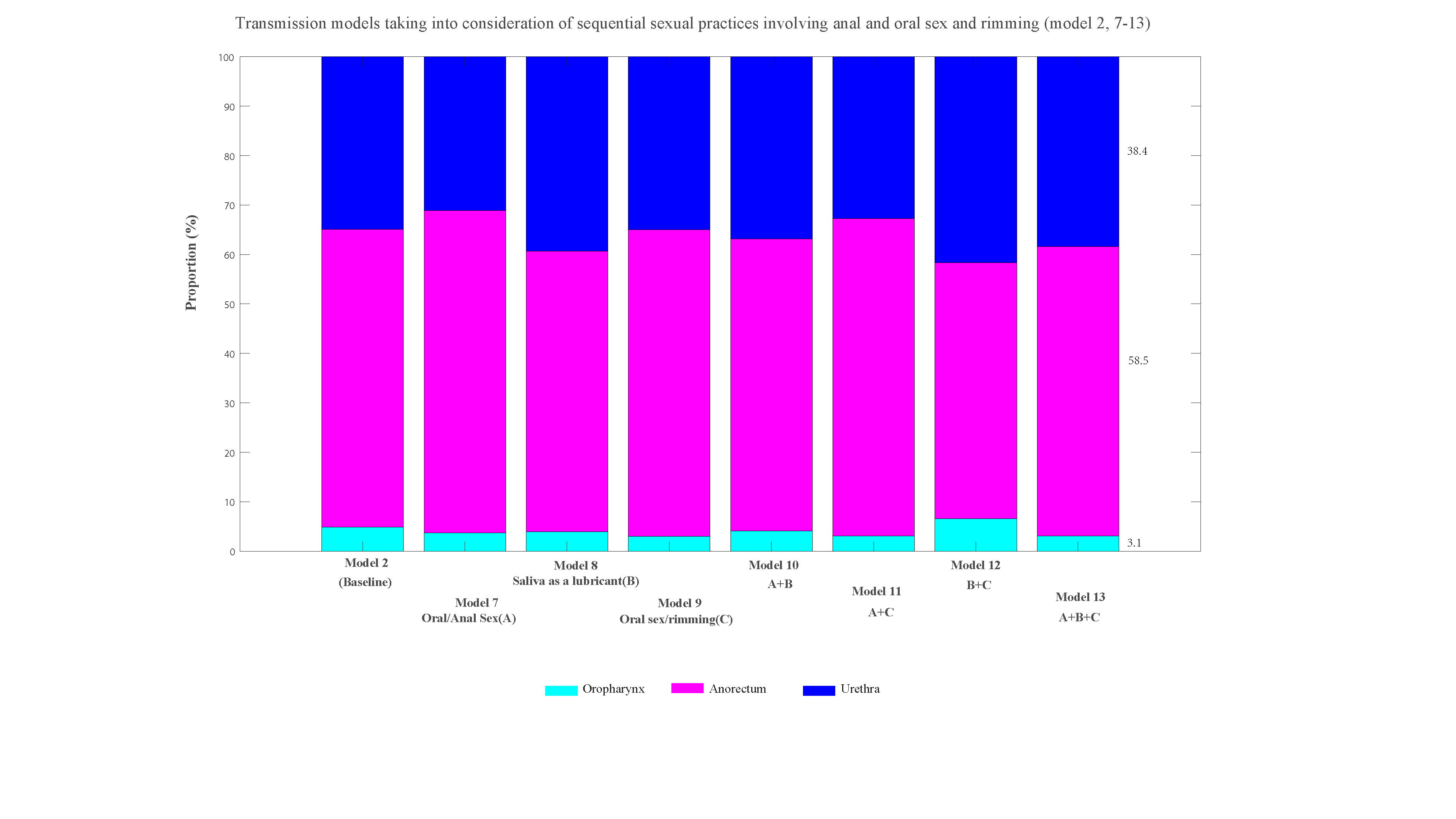


**Figure S17b.** Estimated proportion of incident *Chlamydia trachomatis* cases that occur at the oropharynx, anorectum or urethra in MSM from the eight models (model 2, 7-13) among MSM surveillance data (271, 242 consultations) from all Dutch STI clinics during 2008-2017


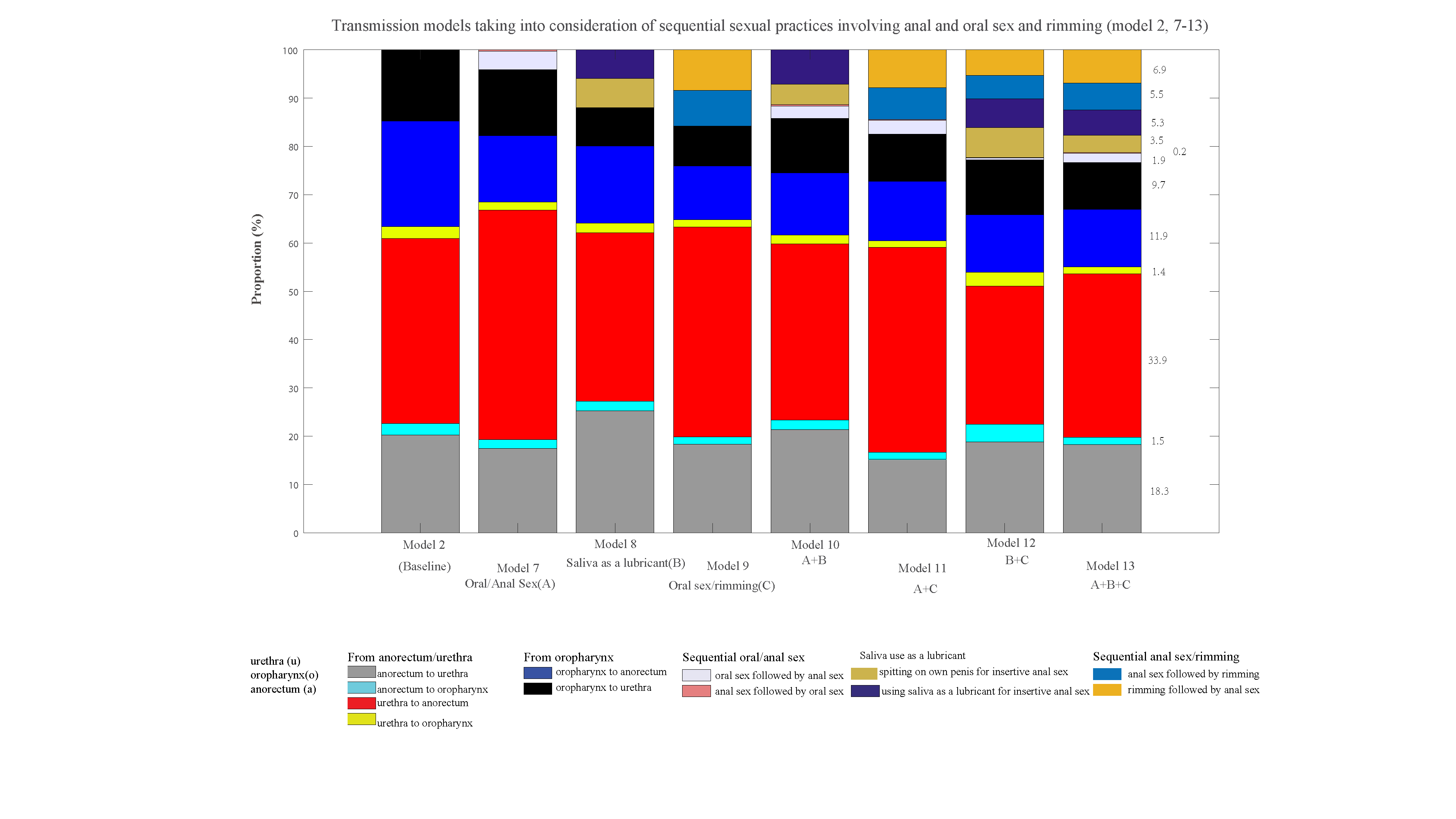


**Figure S17c.** Estimated proportion of incident *Chlamydia trachomatis* cases caused by sexual practices in MSM from the eight models (model 2, 7-13) among MSM surveillance data (271, 242 consultations) from all Dutch STI clinics during 2008-2017

**Validation of Results (Dataset 4): Published validation data from 1,610 community MSM in Thailand**


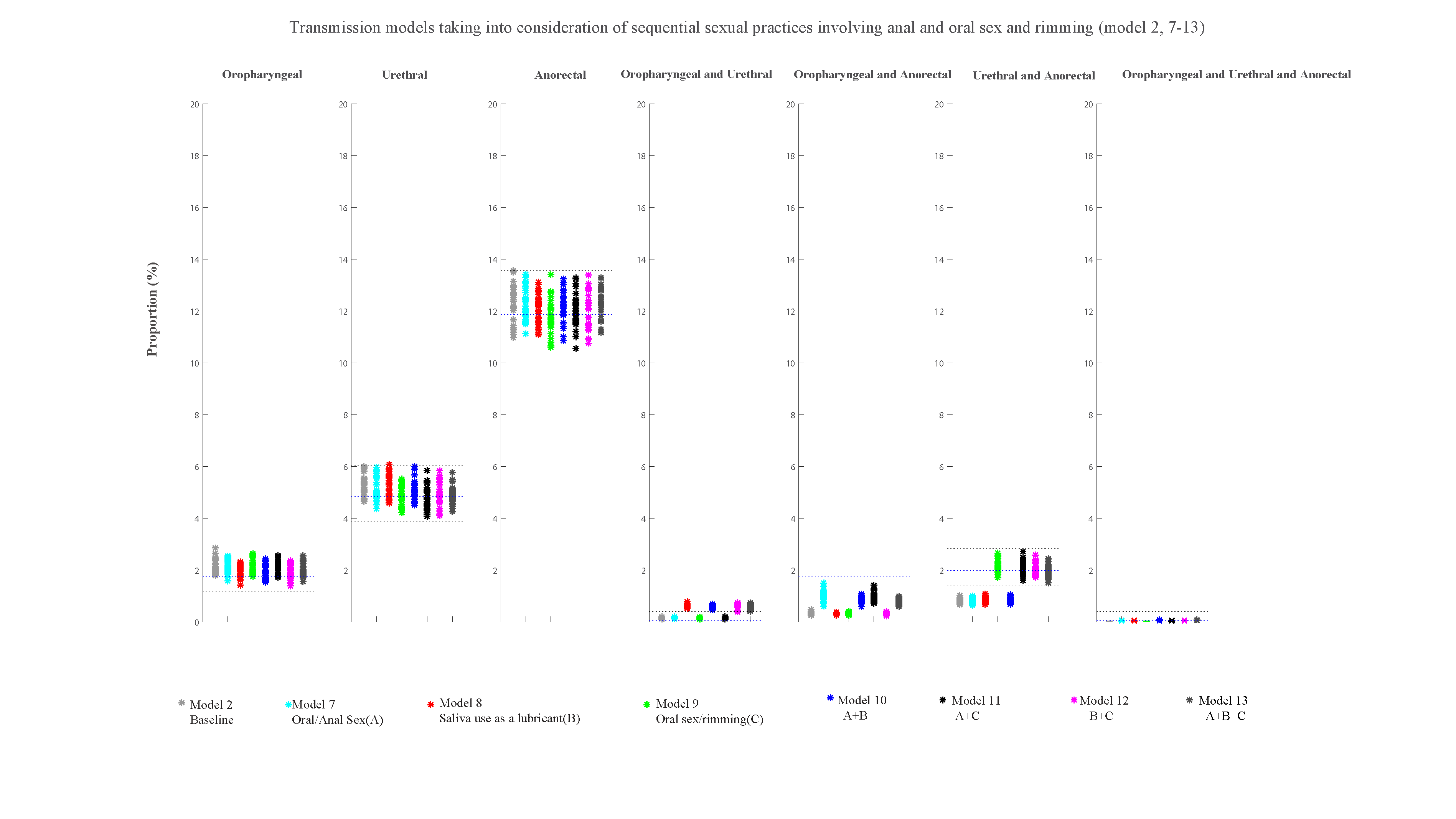


**Figure S18a.** Estimates of the eight models for the percentage of specific anatomical sites positive for *Chlamydia trachomatis* for the 8 models (model 2, 7-13) and the 95% confidence intervals for the observed site-specific positivity among 1,610 MSM attending a community-led test and treat cohort in Thailand between October 2015 and October 2016


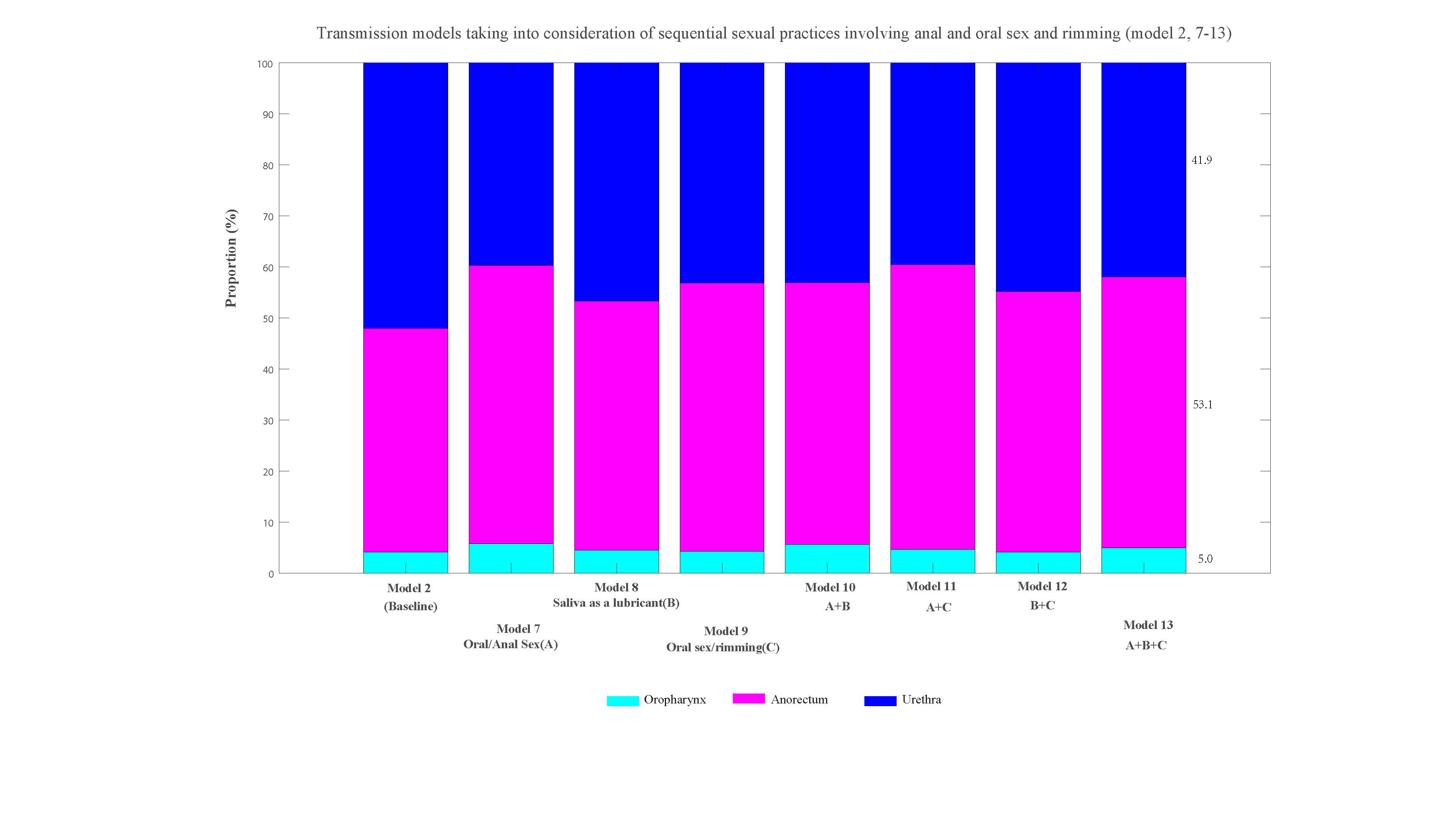


**Figure S18b.** Estimated proportion of incident *Chlamydia trachomatis* cases that occur at the oropharynx, anorectum or urethra in MSM from the eight models (model 2, 7-13) among 1,610 MSM attending a community-led test and treat cohort in Thailand between October 2015 and October 2016


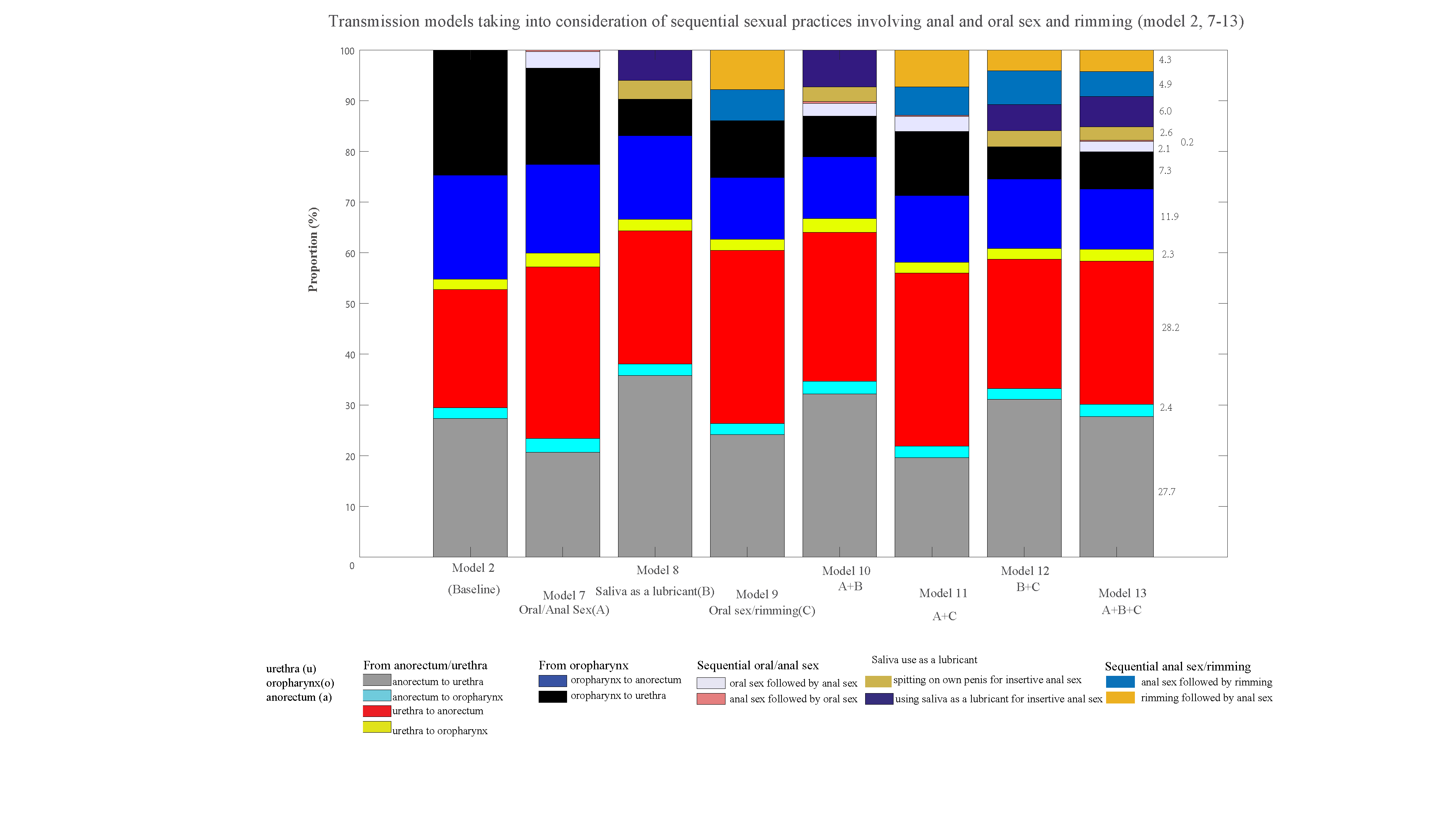


**Figure S18c.** Estimated proportion of incident *Chlamydia trachomatis* cases caused by sexual practices in MSM from the eight models (model 2, 7-13) among 1,610 MSM attending a community-led test and treat cohort in Thailand between October 2015 and October 2016

**Validation of Results (Dataset 5): Published validation data from 179 MSM with HIV in the USA**


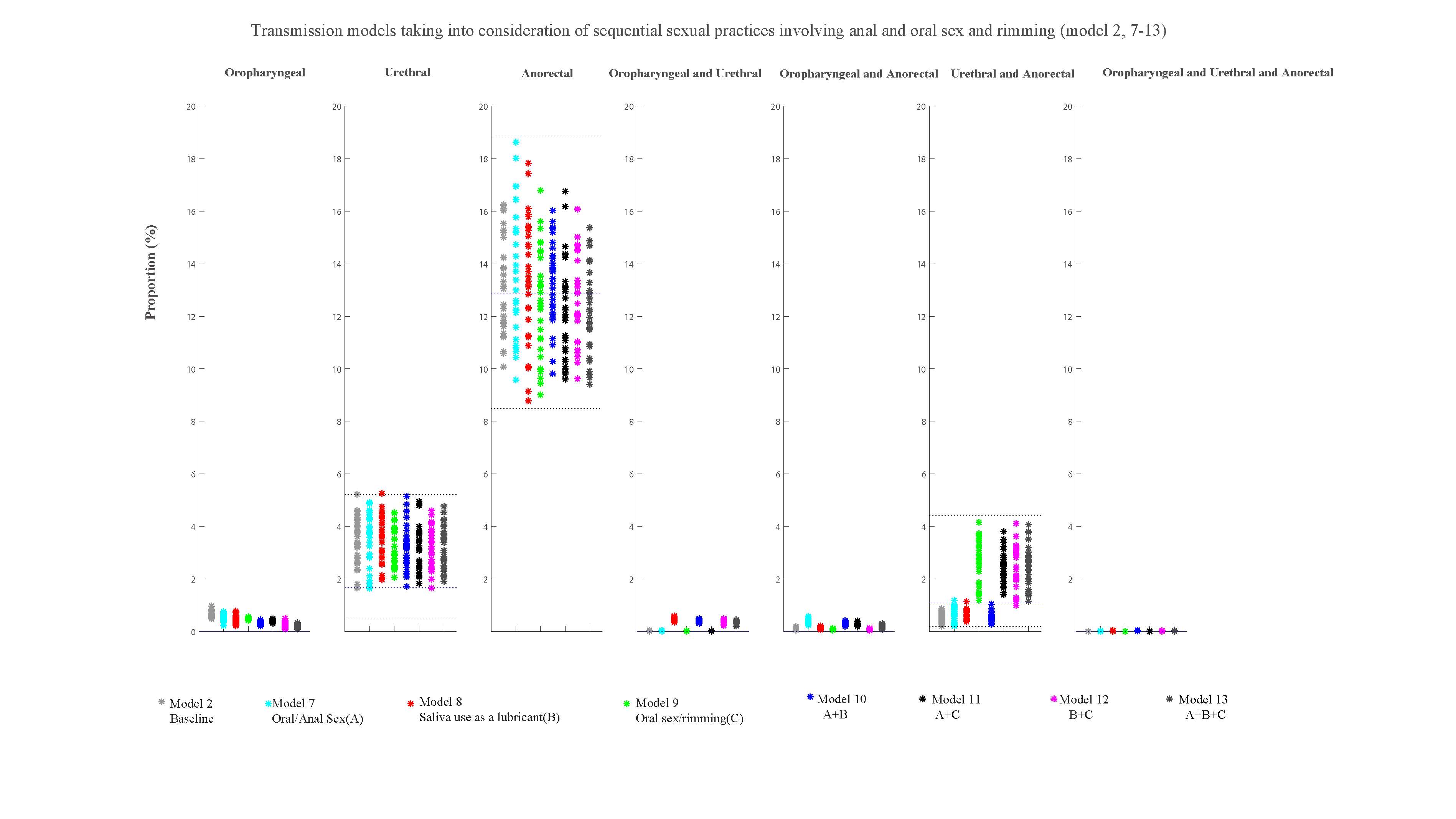


**Figure S19a.** Estimates of the eight models for the percentage of specific anatomical sites positive for *Chlamydia trachomatis* for the 8 models (model 2, 7-13) and the 95% confidence intervals for the observed site-specific positivity among179 MSM with HIV


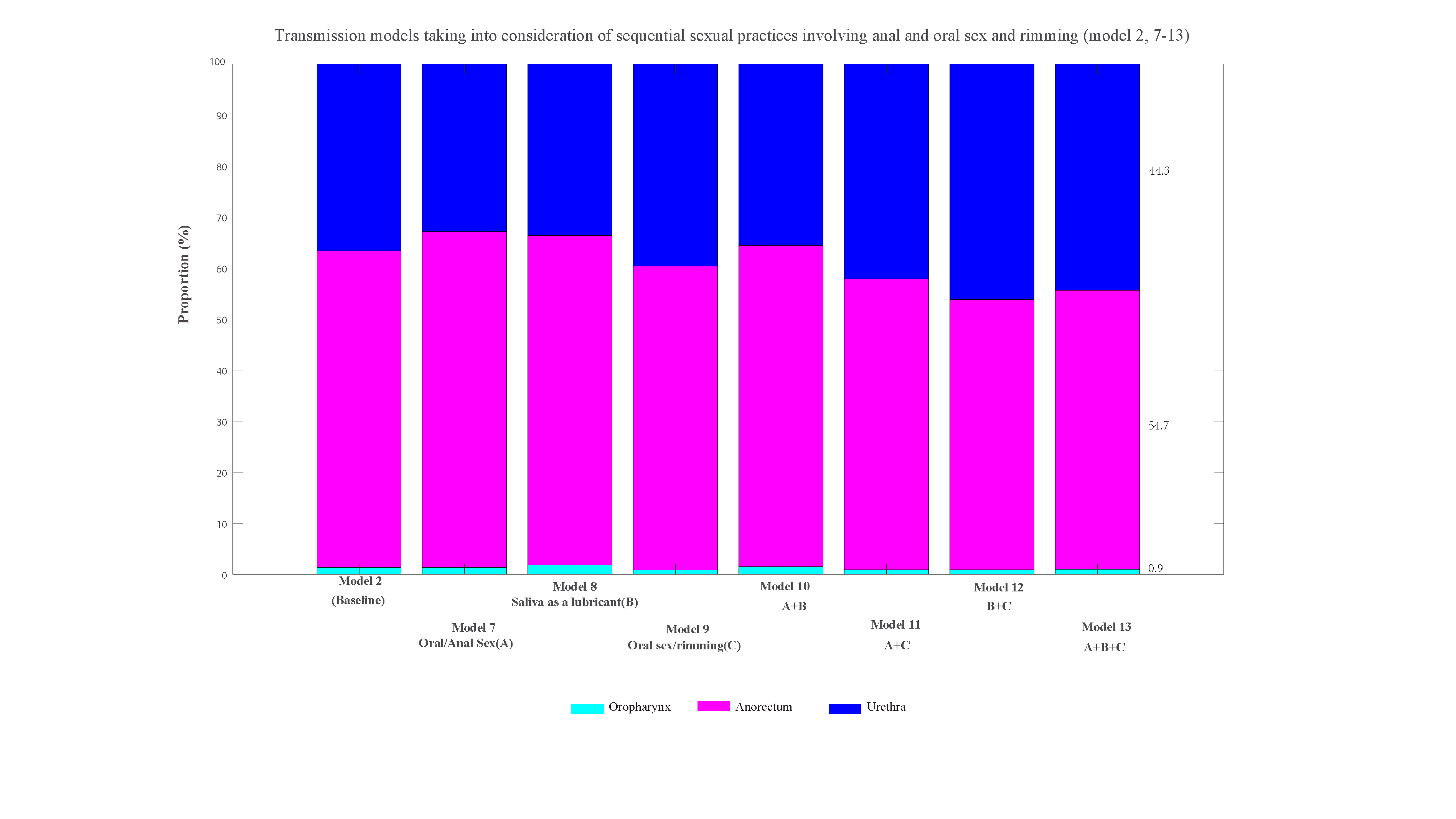


**Figure S19b.** Estimated proportion of incident *Chlamydia trachomatis* cases that occur at the oropharynx, anorectum or urethra in MSM from the eight models (model 2, 7-13) among179 MSM with HIV


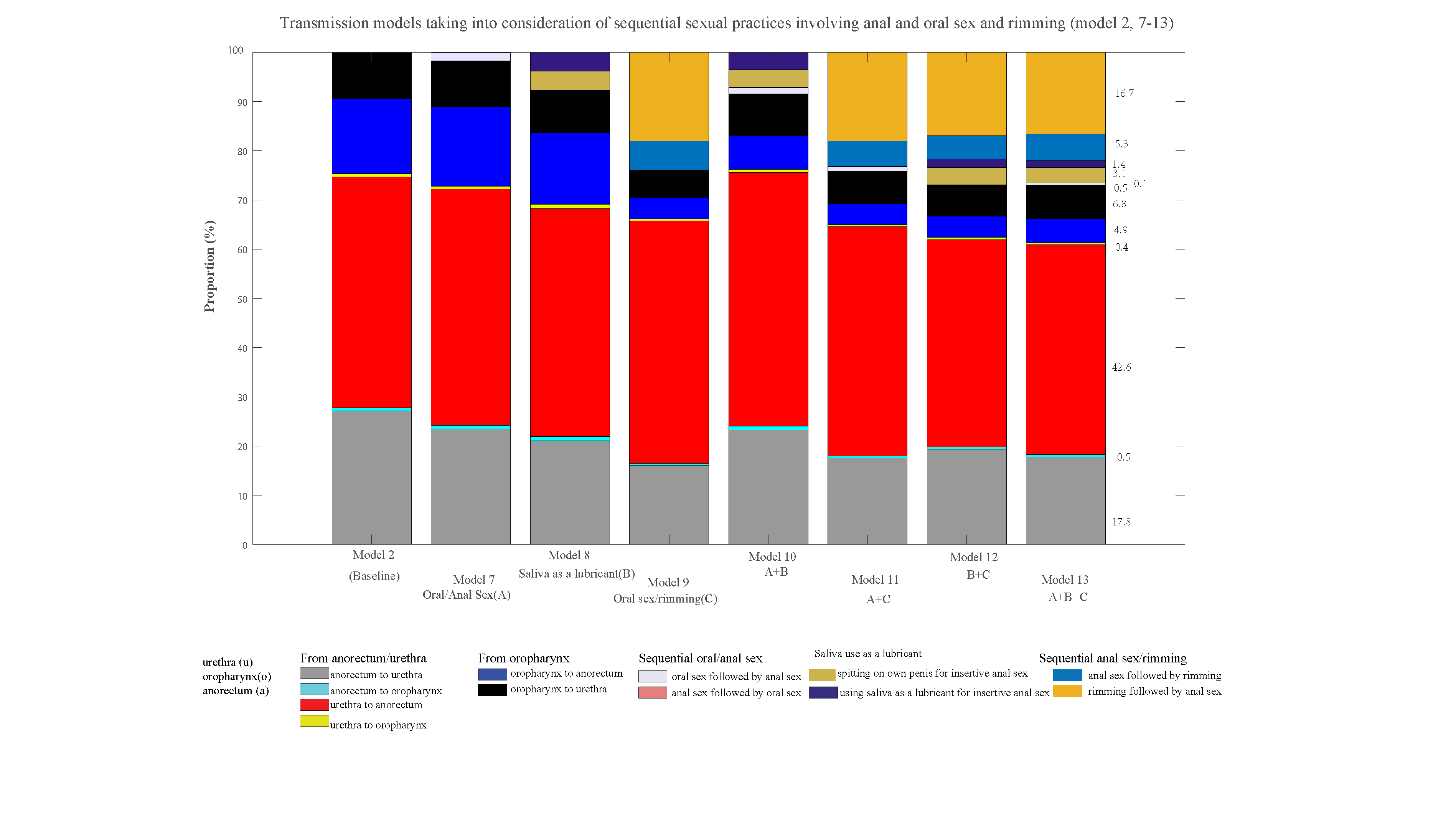


**Figure S19c.** Estimated proportion of incident *Chlamydia trachomatis* cases caused by sexual practices in MSM from the eight models (model 2, 7-13) among179 MSM with HIV

**Supplementary results: ‘Anal sex, oral sex, rimming, kissing and sequential sexual practices’ transmission models (Model 3,14-20)**

**Unpublished data from 4888 MSM attending Melbourne Sexual Health Centre for model (model 3,14-20) calibration**

**
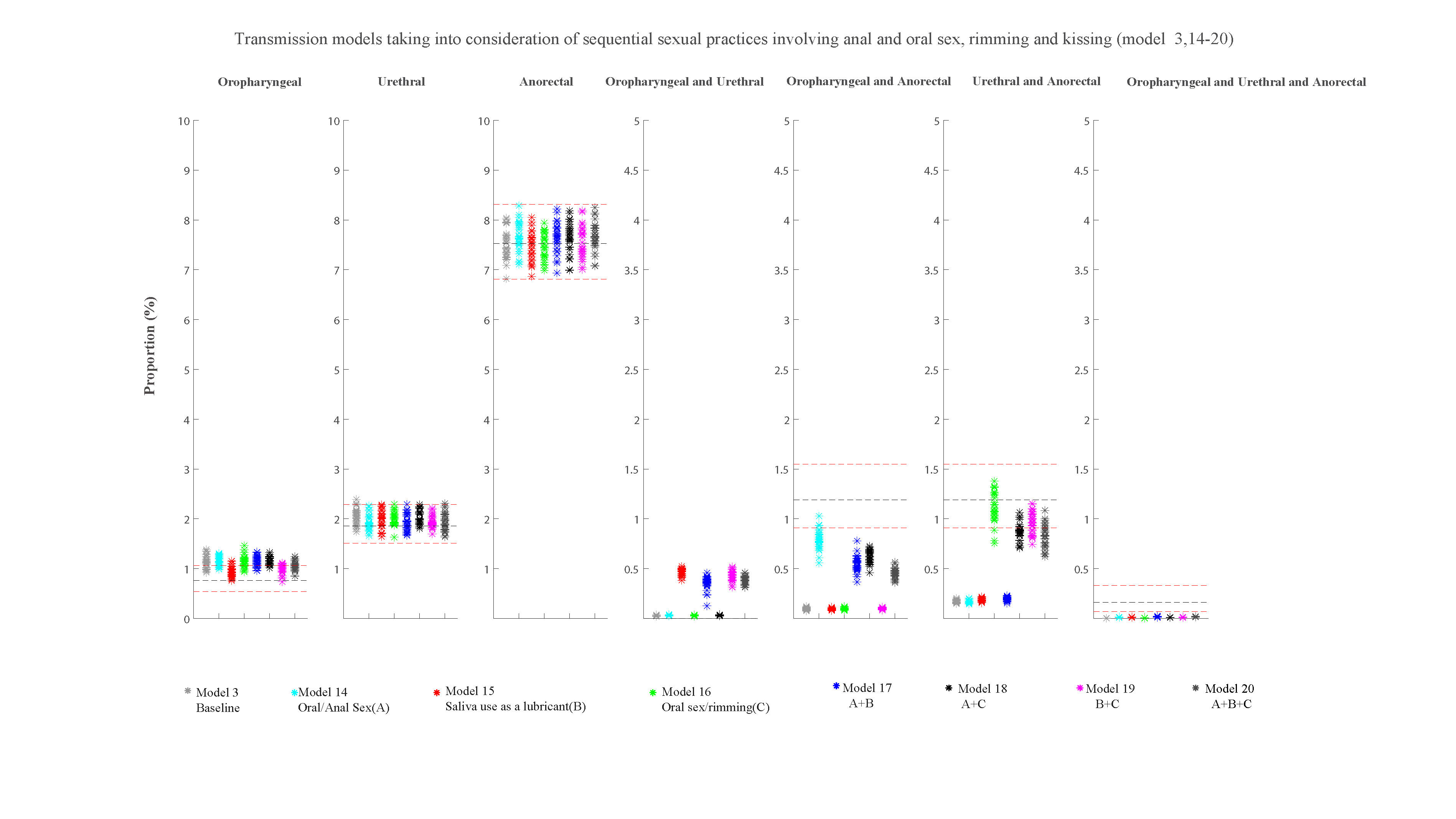
**

**Figure S20a.** Estimates of the eight models for the percentage of specific anatomical sites positive for *Chlamydia trachomatis* for the 8 models (model 3, 14-20) and the 95% confidence intervals for the observed site-specific positivity among 4888 MSM attending Melbourne Sexual Health Centre in 2018 and 2019

**
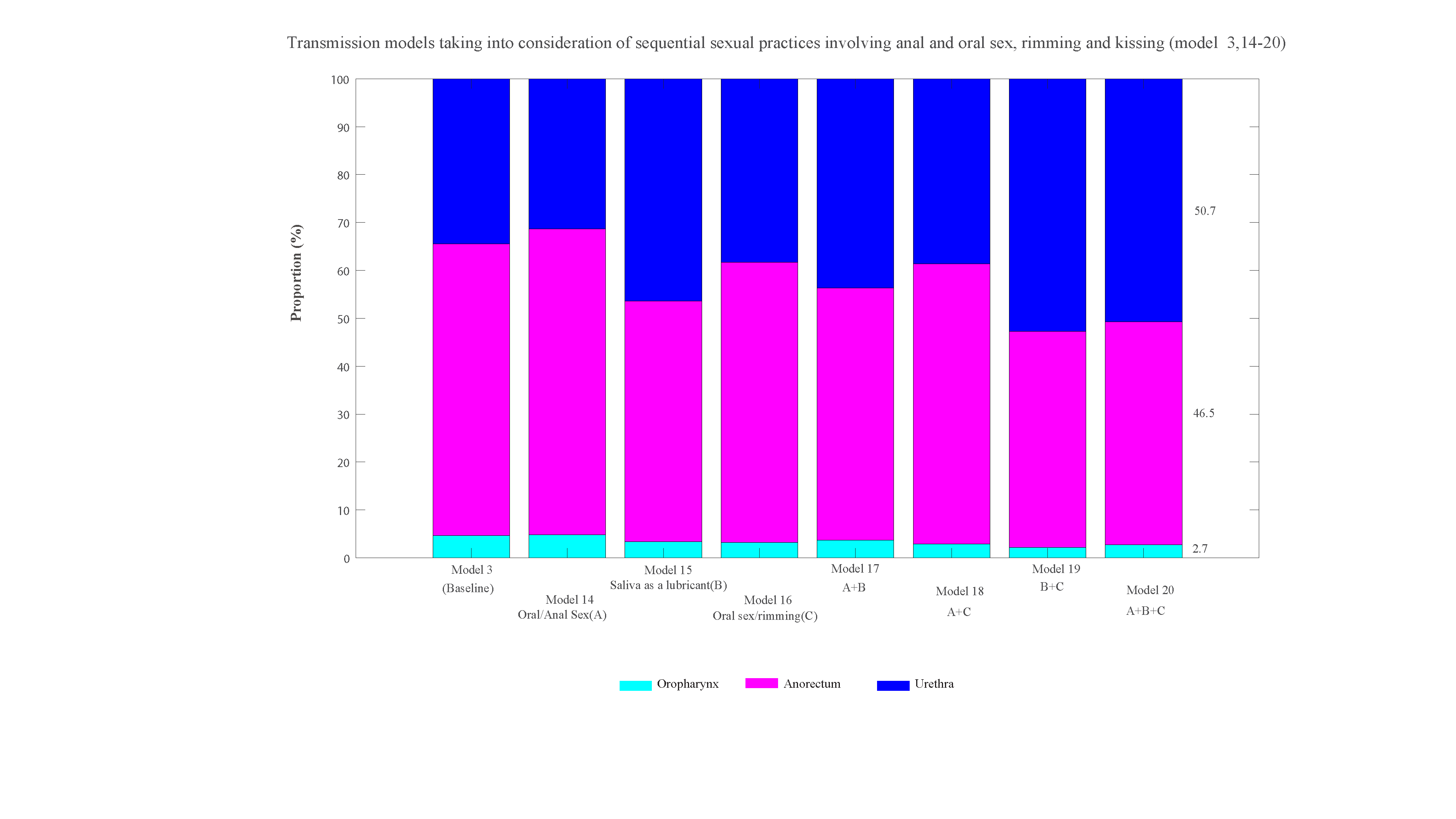
**

**Figure S20b**. Estimated proportion of incident *Chlamydia trachomatis* cases that occur at the oropharynx, anorectum or urethra in MSM from the 8 models (model 3, 14-20) among 4888 MSM attending Melbourne Sexual Health Centre in 2018 and 2019

**
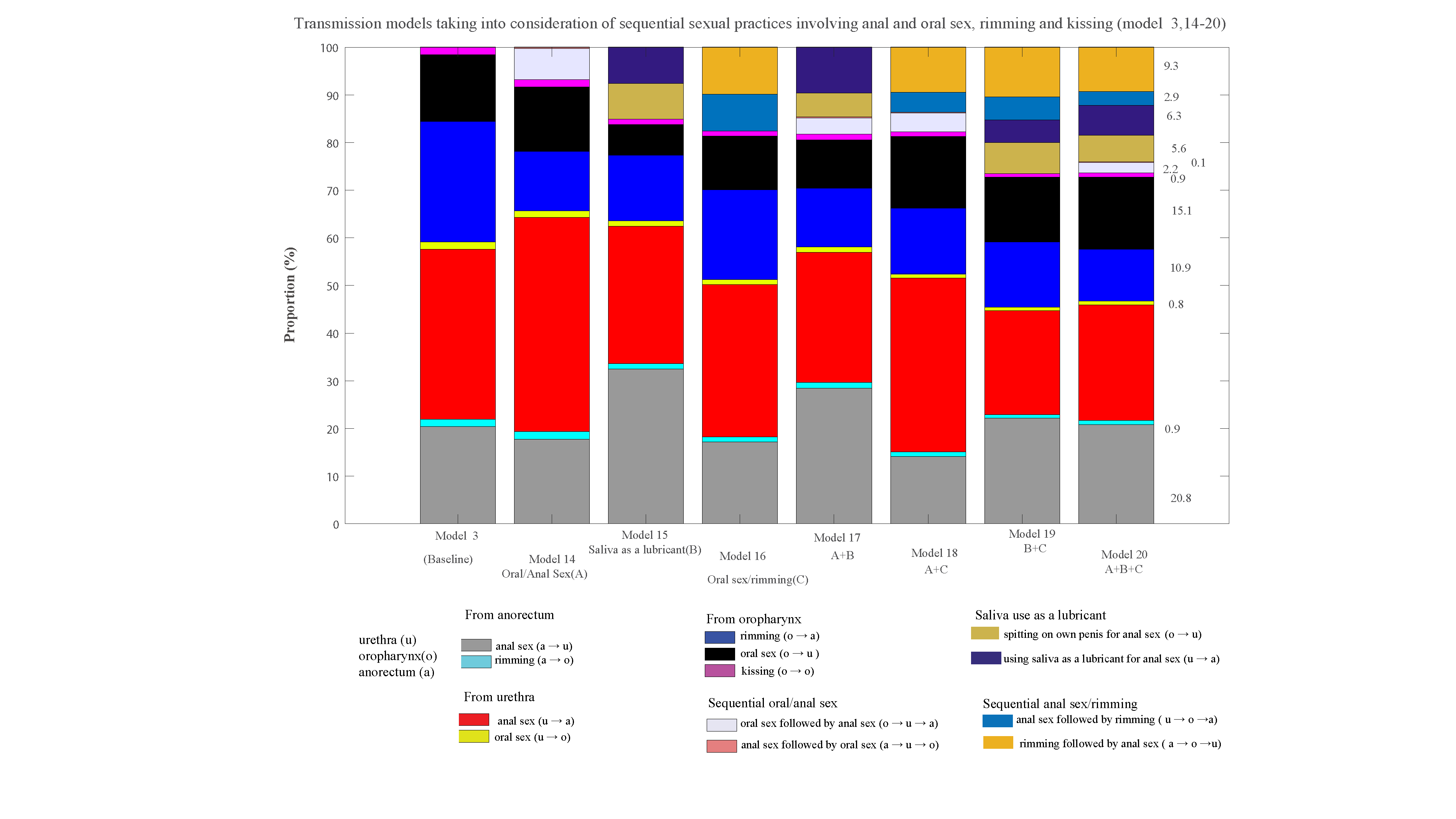
**

**Figure S20c**. Estimated proportion of incident *Chlamydia trachomatis* cases caused by sexual practices in MSM from the 8 models (model 3,14-20) among 4888 MSM attending Melbourne Sexual Health Centre in 2018 and 2019

**Validation of Results (Dataset 1): Published validation data from 1,011 asymptomatic MSM attending Melbourne Sexual Health Centre**

**
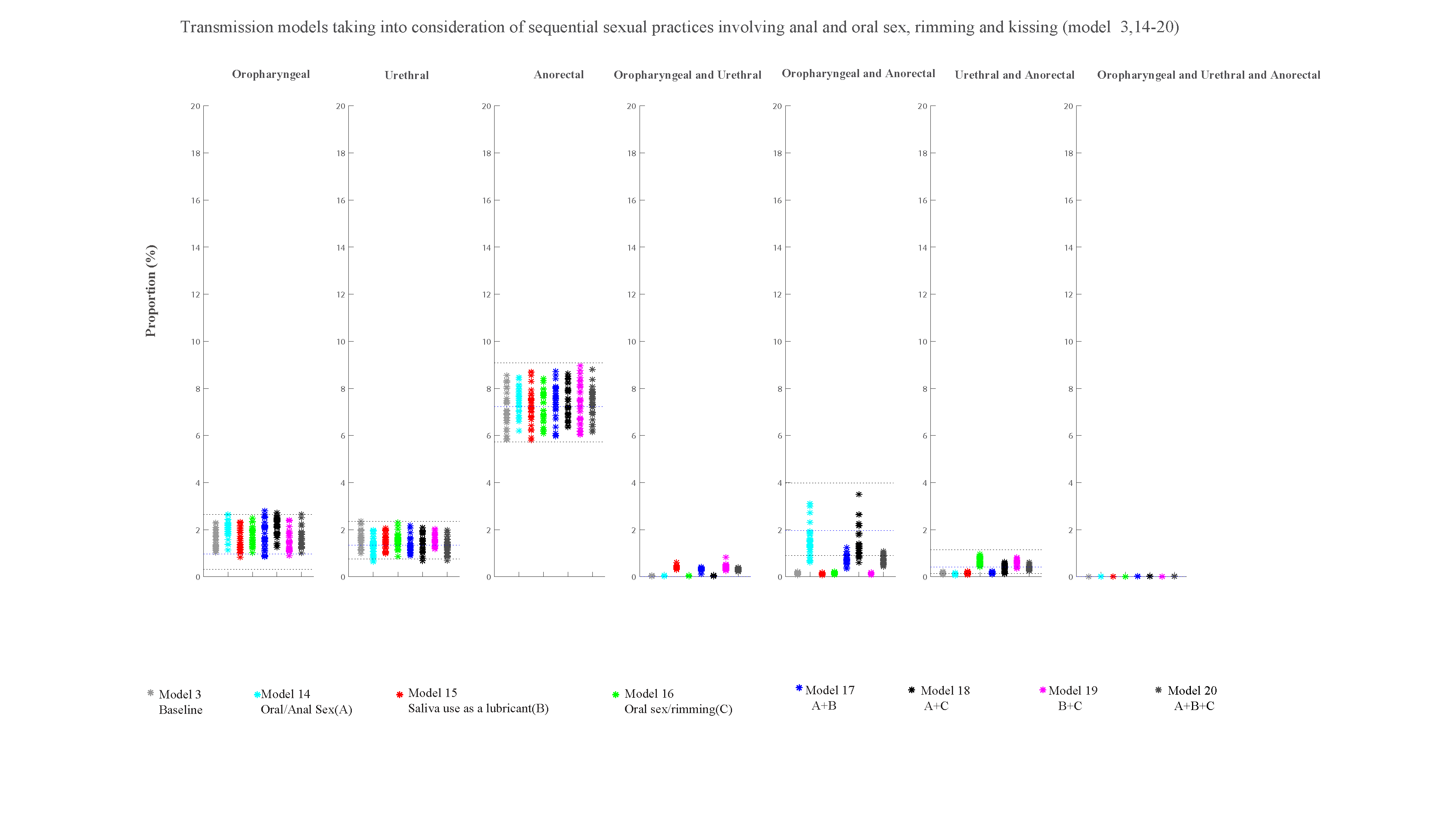
**

**Figure S21a.** Estimates of the eight models for the percentage of specific anatomical sites positive for *Chlamydia trachomatis* for the 8 models (model 3, 14-20) and the 95% confidence intervals for the observed site-specific positivity among 1,011 asymptomatic MSM attending MSHC during 2016–2017

**
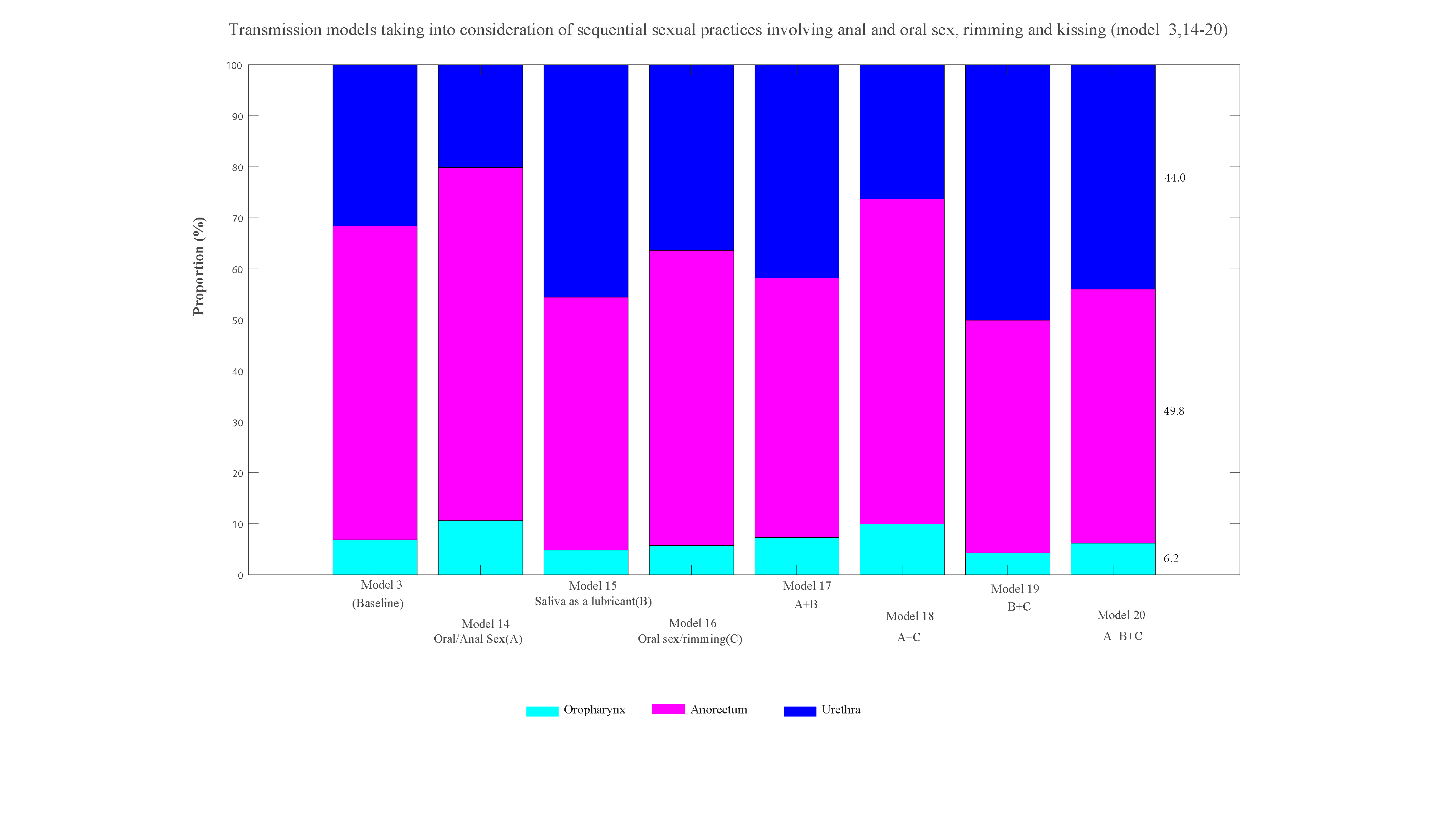
**

**Figure S21b.** Estimated proportion of incident *Chlamydia trachomatis* cases that occur at the oropharynx, anorectum or urethra in MSM from the 8 models (model 3, 14-20) among 1,011 asymptomatic MSM attending MSHC during 2016–2017

**
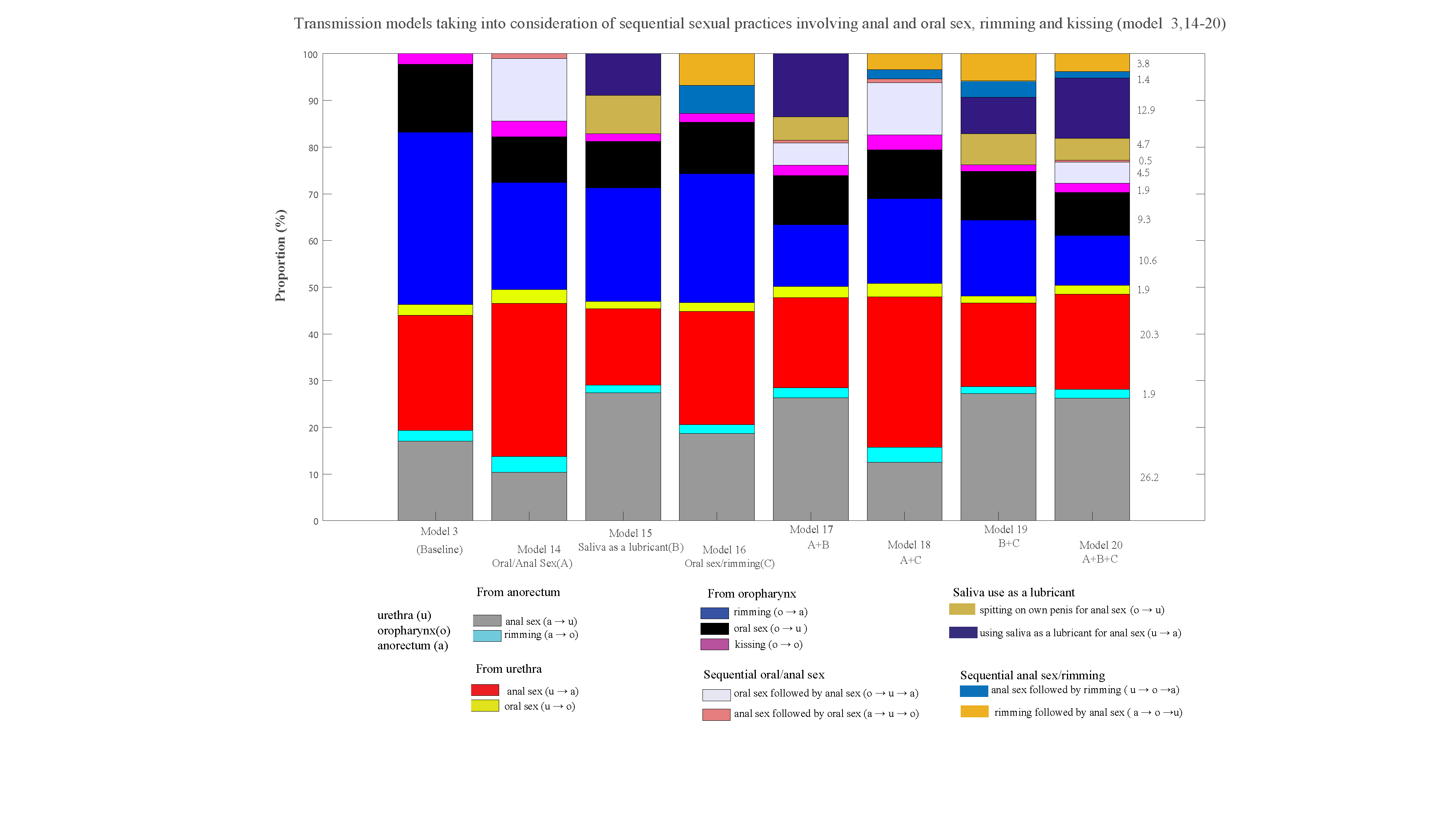
**

**Figure S21c.** Estimated proportion of incident *Chlamydia trachomatis* cases caused by sexual practices in MSM from the 8 models (model 3, 14-20) among 1,011 asymptomatic MSM attending MSHC during 2016–2017

Validation of Results (Dataset 2): Published validation data from 393MSM attending STD & HIV care clinics in the USA

**
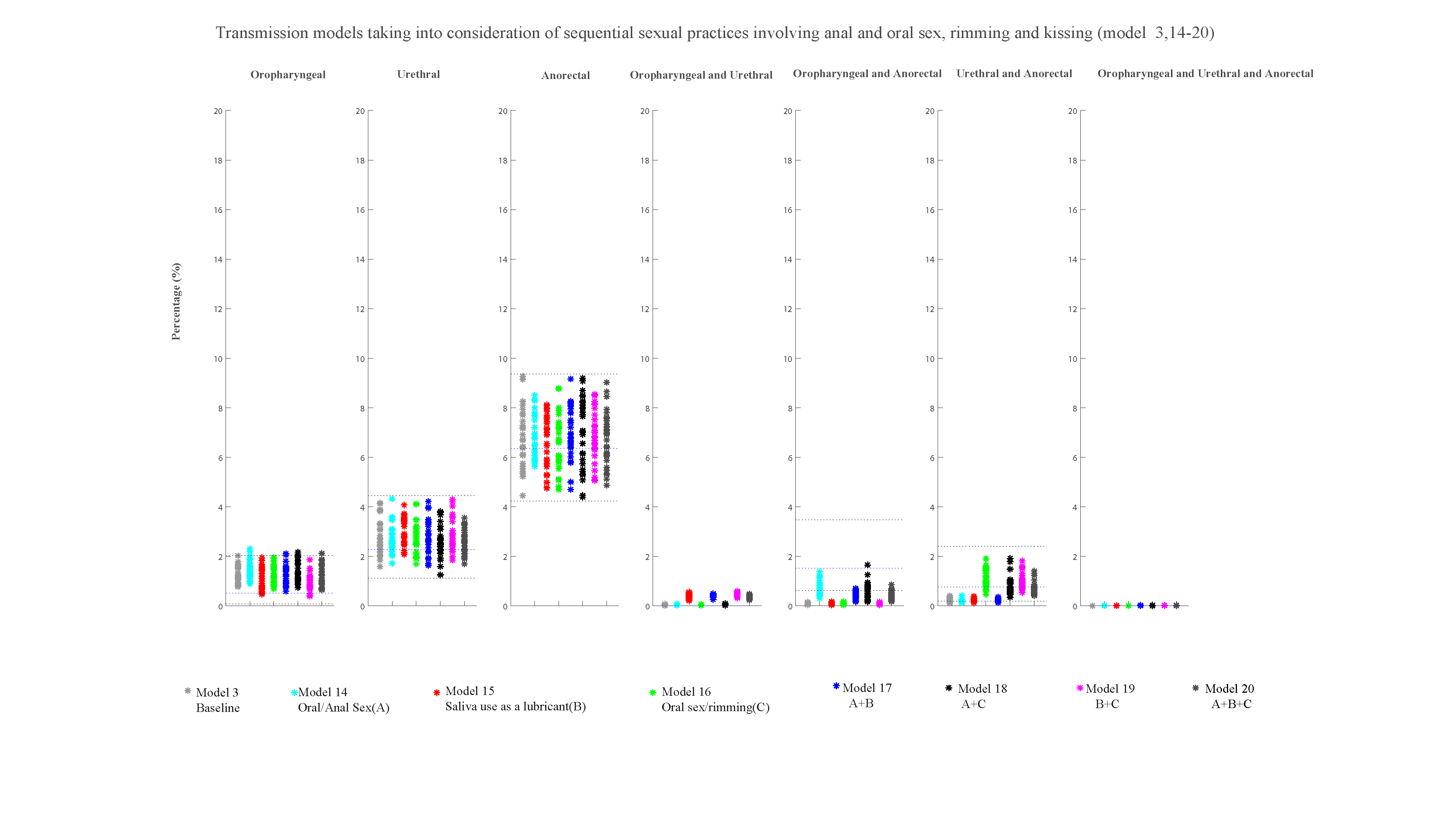
**

**Figure S22a.** Estimates of the eight models for the percentage of specific anatomical sites positive for *Chlamydia trachomatis* for the 8 models (model 3,14-20) and the 95% confidence intervals for the observed site-specific positivity among 393MSM attending STD & HIV care clinics in the USA

**
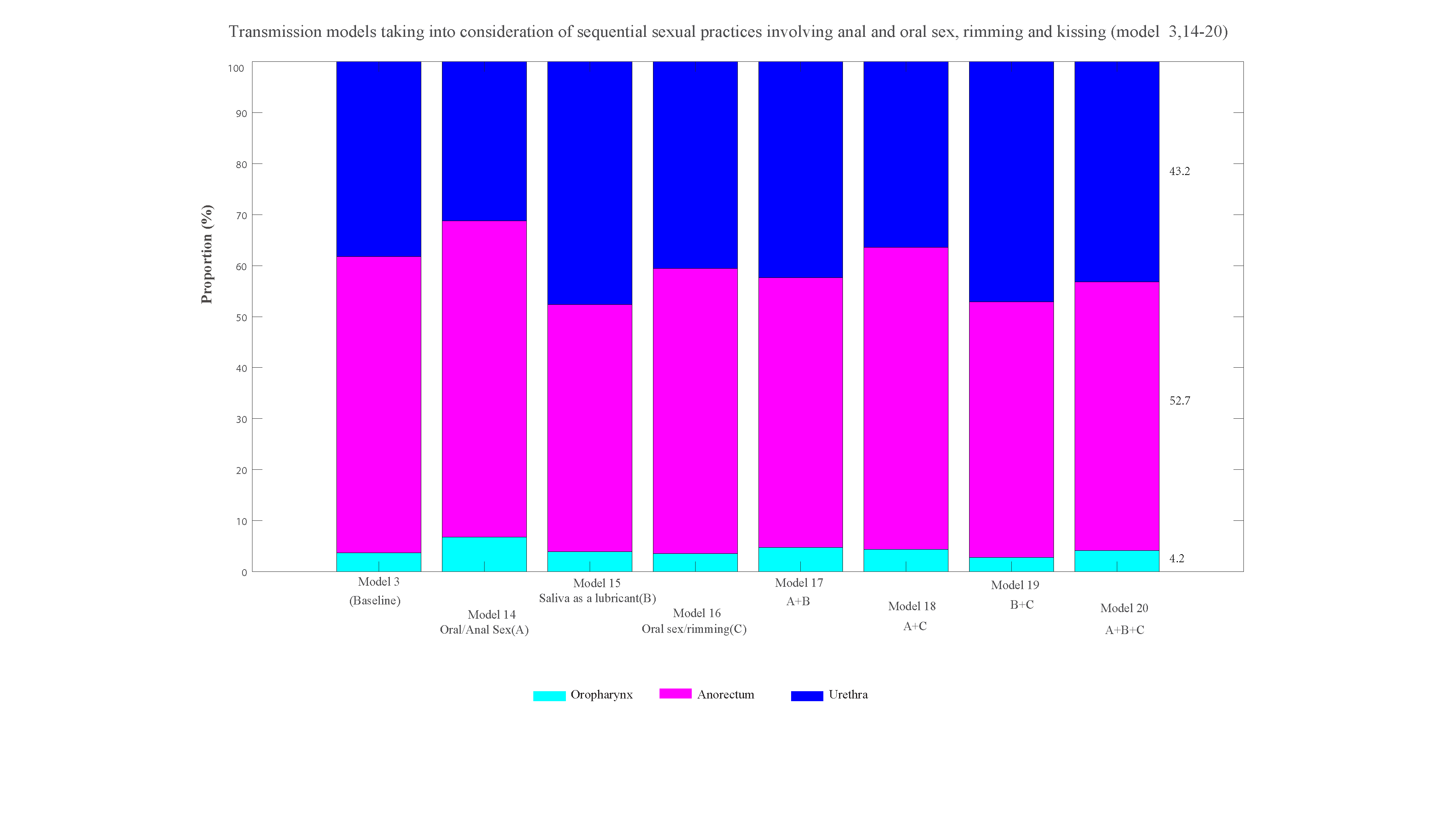
**

**Figure S22b.** Estimated proportion of incident *Chlamydia trachomatis* cases that occur at the oropharynx, anorectum or urethra in MSM from the 8 models (model 3,14-20) among 393MSM attending STD & HIV care clinics in the USA

**
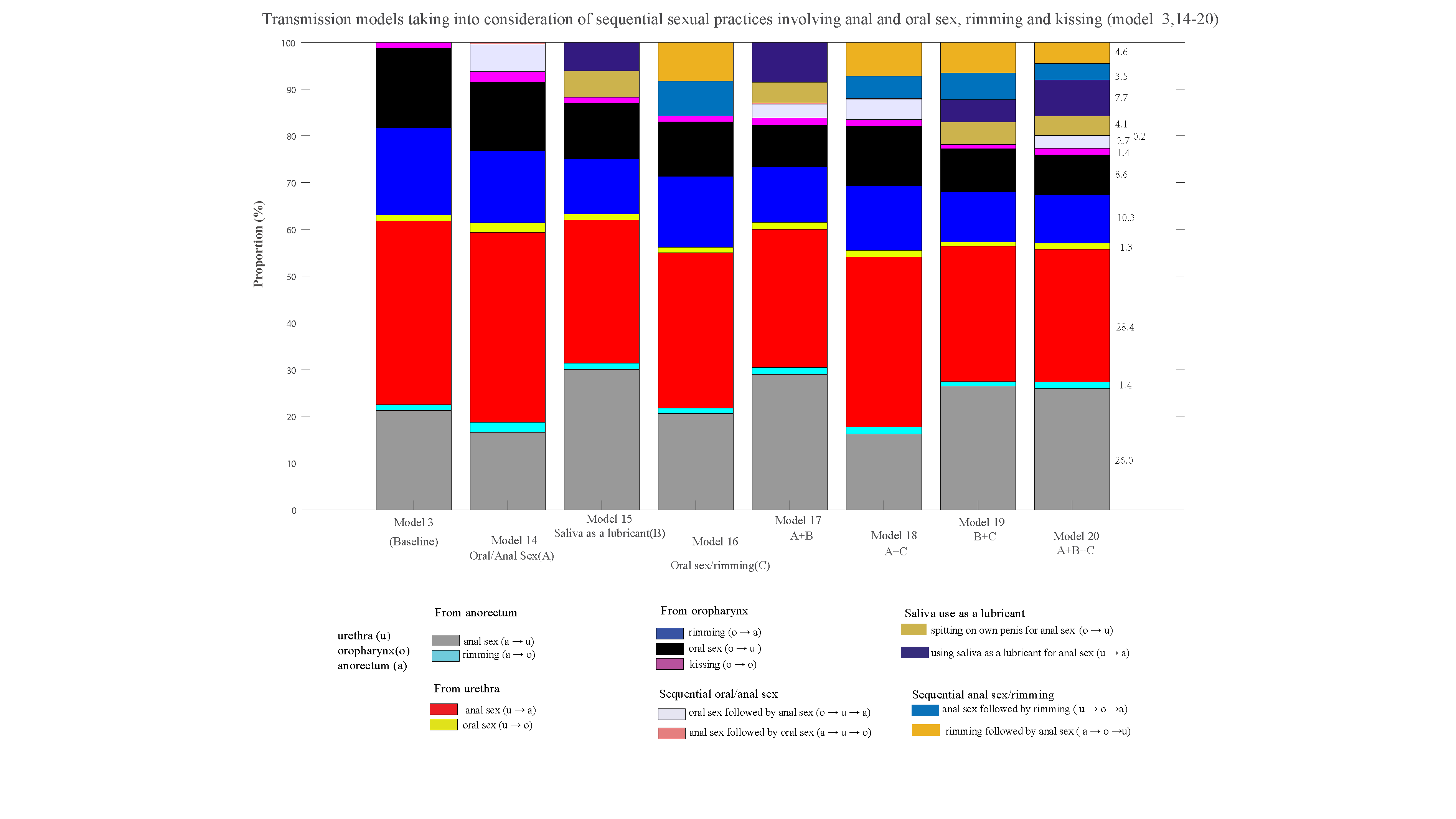
**

**Figure S22c.** Estimated proportion of incident *Chlamydia trachomatis* cases caused by sexual practices in MSM from the 8 models (model 3,14-20) among 393MSM attending STD & HIV care clinics in the USA

**Validation of Results (Dataset 3): Published validation data from MSM surveillance data of all Dutch STI clinics**

**
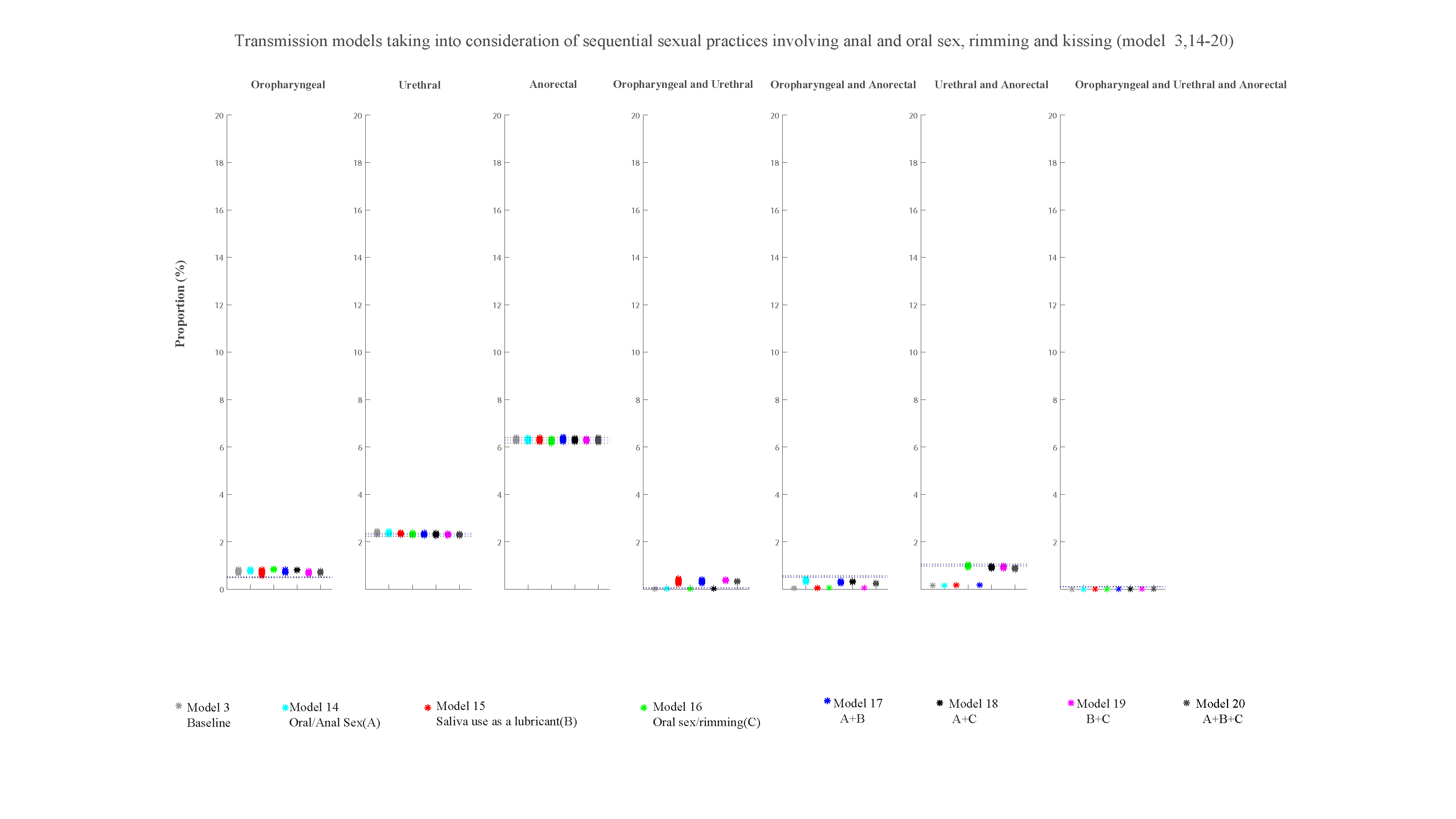
**

**Figure S23a.** Estimates of the eight models for the percentage of specific anatomical sites positive for *Chlamydia trachomatis* for the 8 models (model 3,14-20) and the 95% confidence intervals for the observed site-specific positivity among MSM surveillance data (271, 242 consultations) from all Dutch STI clinics during 2008-2017

**
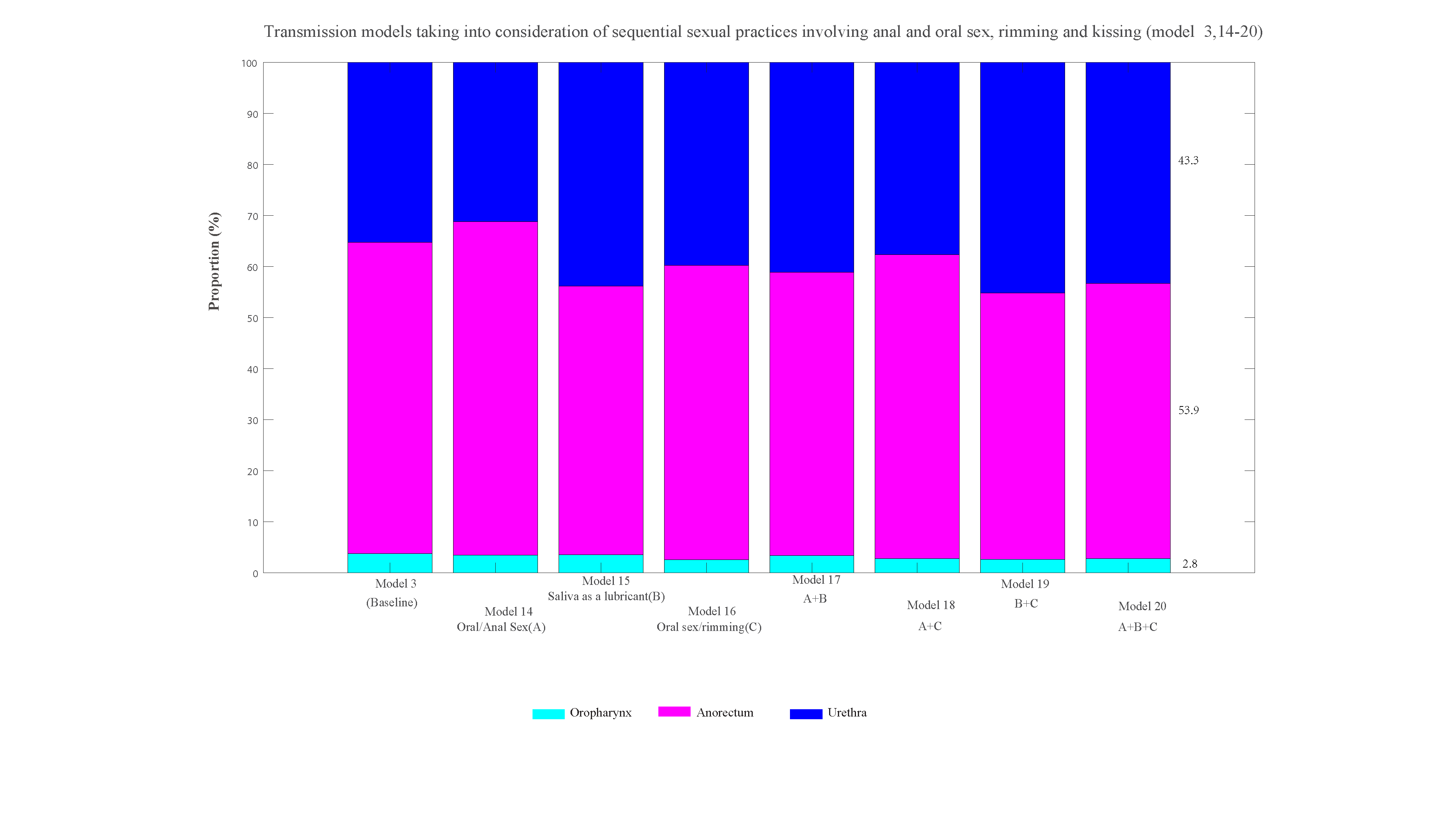
**

**Figure S23b.** Estimated proportion of incident *Chlamydia trachomatis* cases that occur at the oropharynx, anorectum or urethra in MSM from the 8 models (model 3, 14-20) among MSM surveillance data (271, 242 consultations) from all Dutch STI clinics during 2008-2017

**
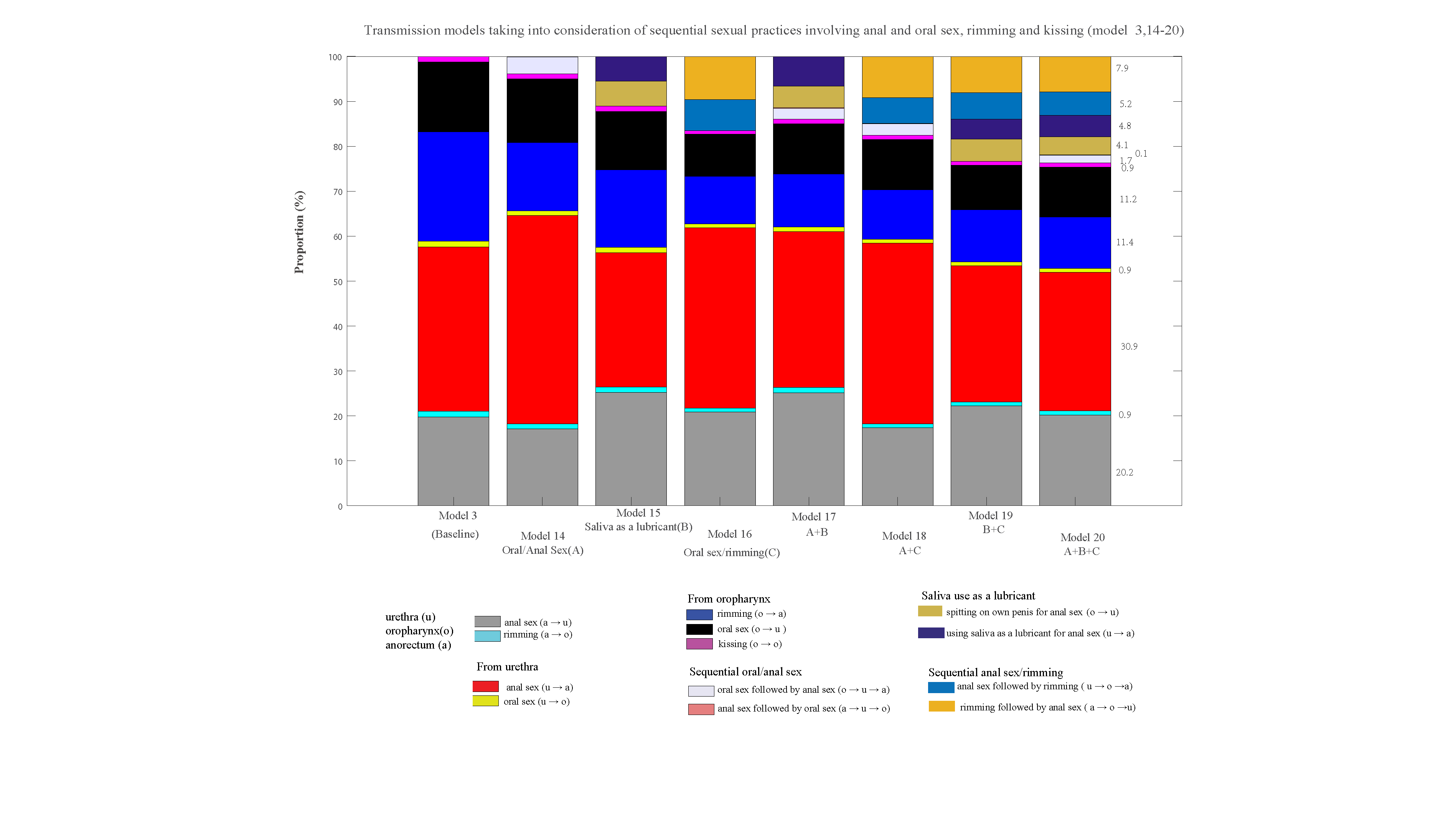
**

**Figure S23c.** Estimated proportion of incident *Chlamydia trachomatis* cases caused by sexual practices in MSM from the 8 models (model 3, 14-20) among MSM surveillance data (271, 242 consultations) from all Dutch STI clinics during 2008-2017

**Validation of Results (Dataset 4): Published validation data from 1,610 community MSM in Thailand**


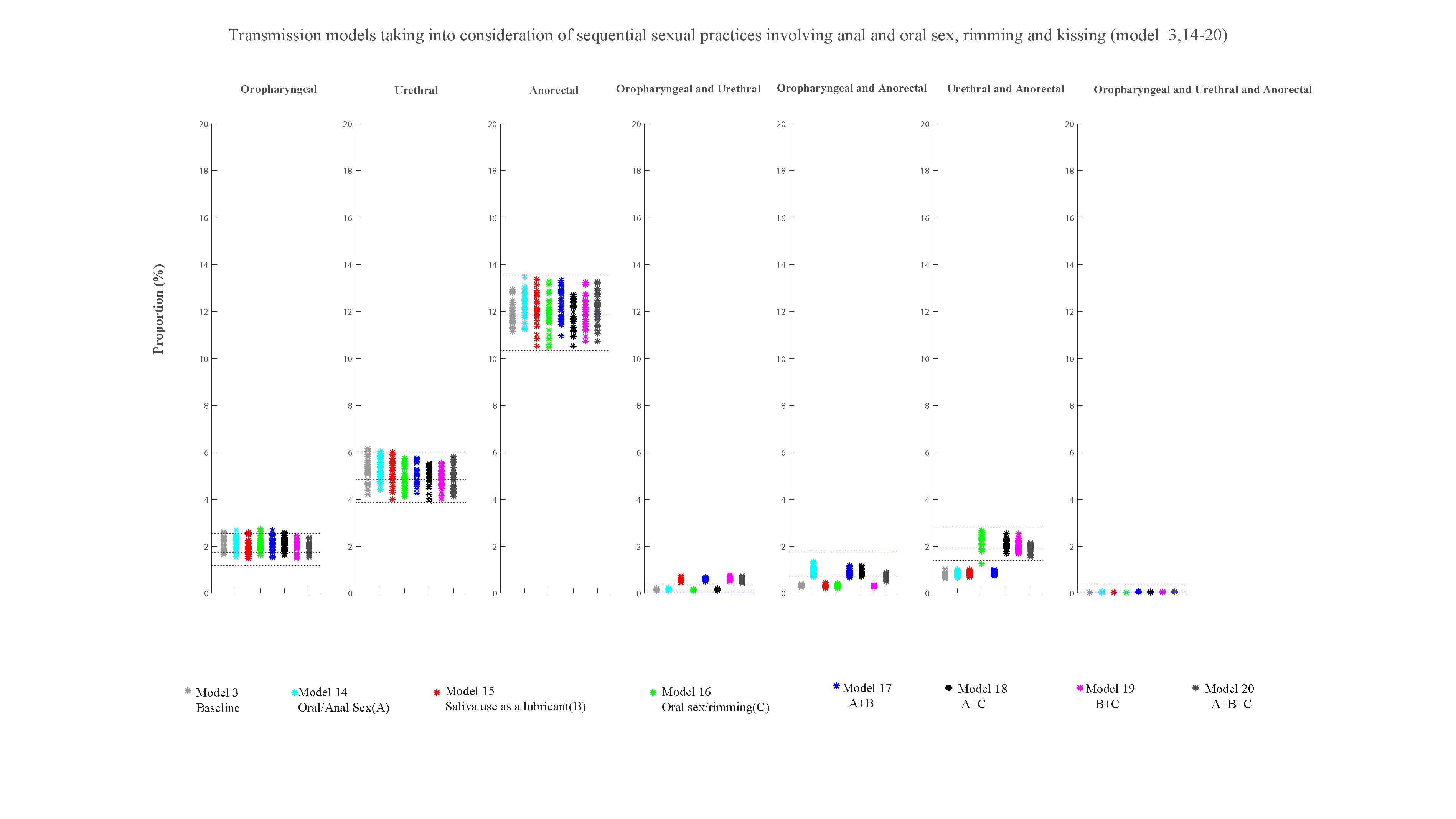


**Figure S24a.** Estimates of the eight models for the percentage of specific anatomical sites positive for *Chlamydia trachomatis* for the 8 models (model 3, 14-20) and the 95% confidence intervals for the observed site-specific positivity among 1,610 MSM attending a community-led test and treat cohort in Thailand between October 2015 and October 2016


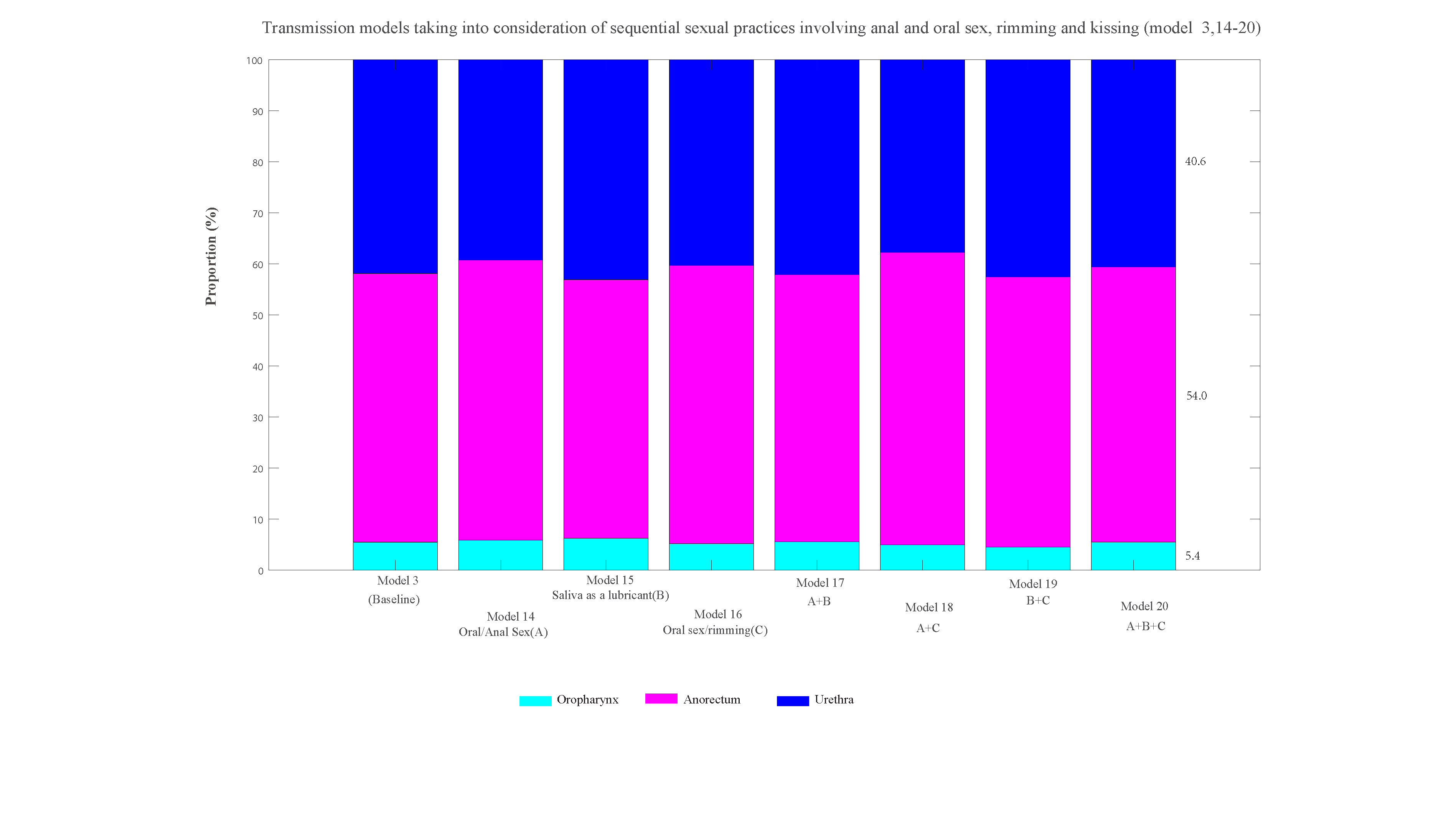


**Figure S24b.** Estimated proportion of incident *Chlamydia trachomatis* cases that occur at the oropharynx, anorectum or urethra in MSM from the 8 models (model 3, 14-20) among 1,610 MSM attending a community-led test and treat cohort in Thailand between October 2015 and October 2016


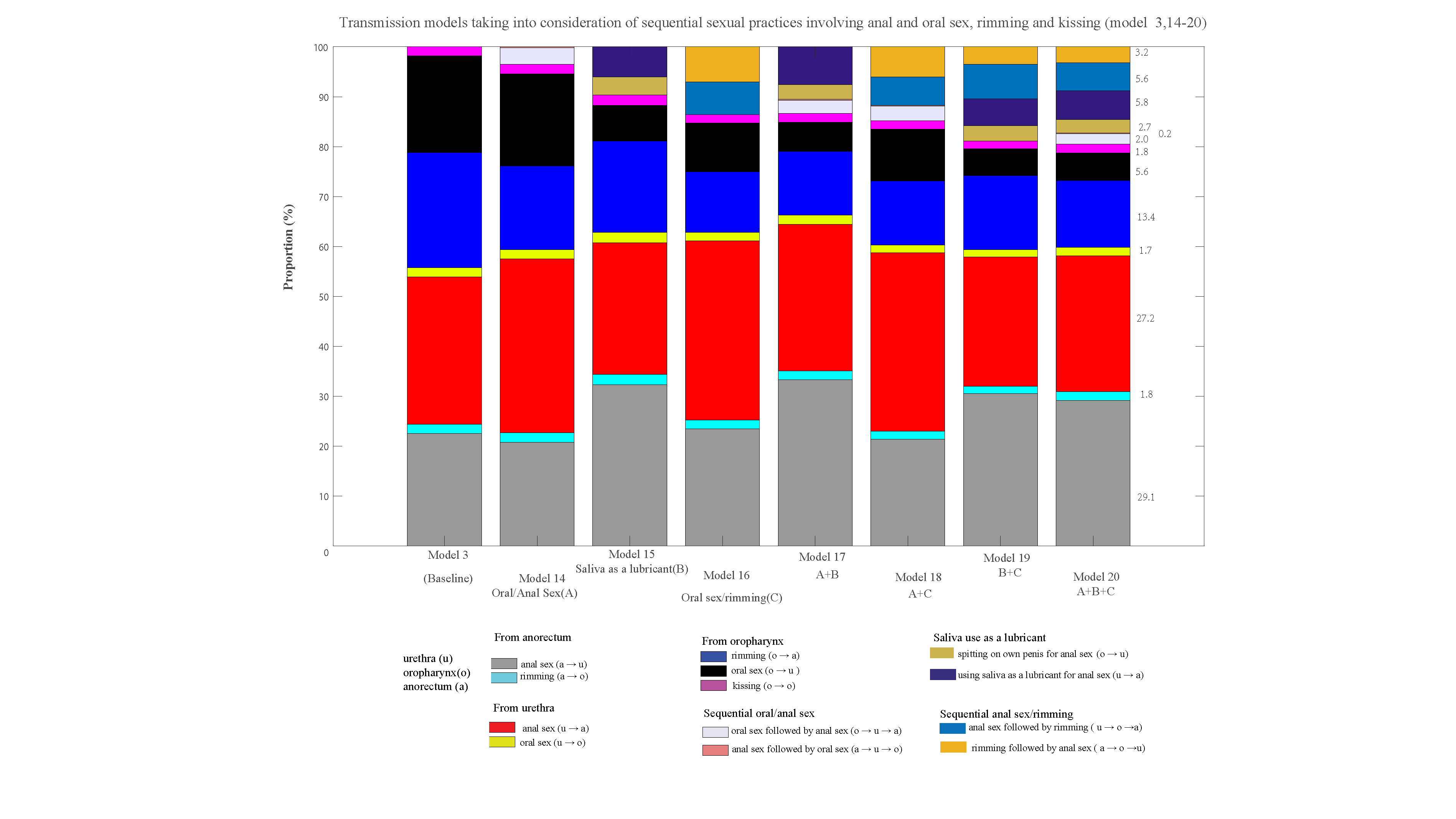


**Figure S24c.** Estimated proportion of incident *Chlamydia trachomatis* cases caused by sexual practices in MSM from the 8 models (model 3, 14-20) among 1,610 MSM attending a community-led test and treat cohort in Thailand between October 2015 and October 2016

**Validation of Results (Dataset 5): Published validation data from 179 MSM with HIV in the USA**

**
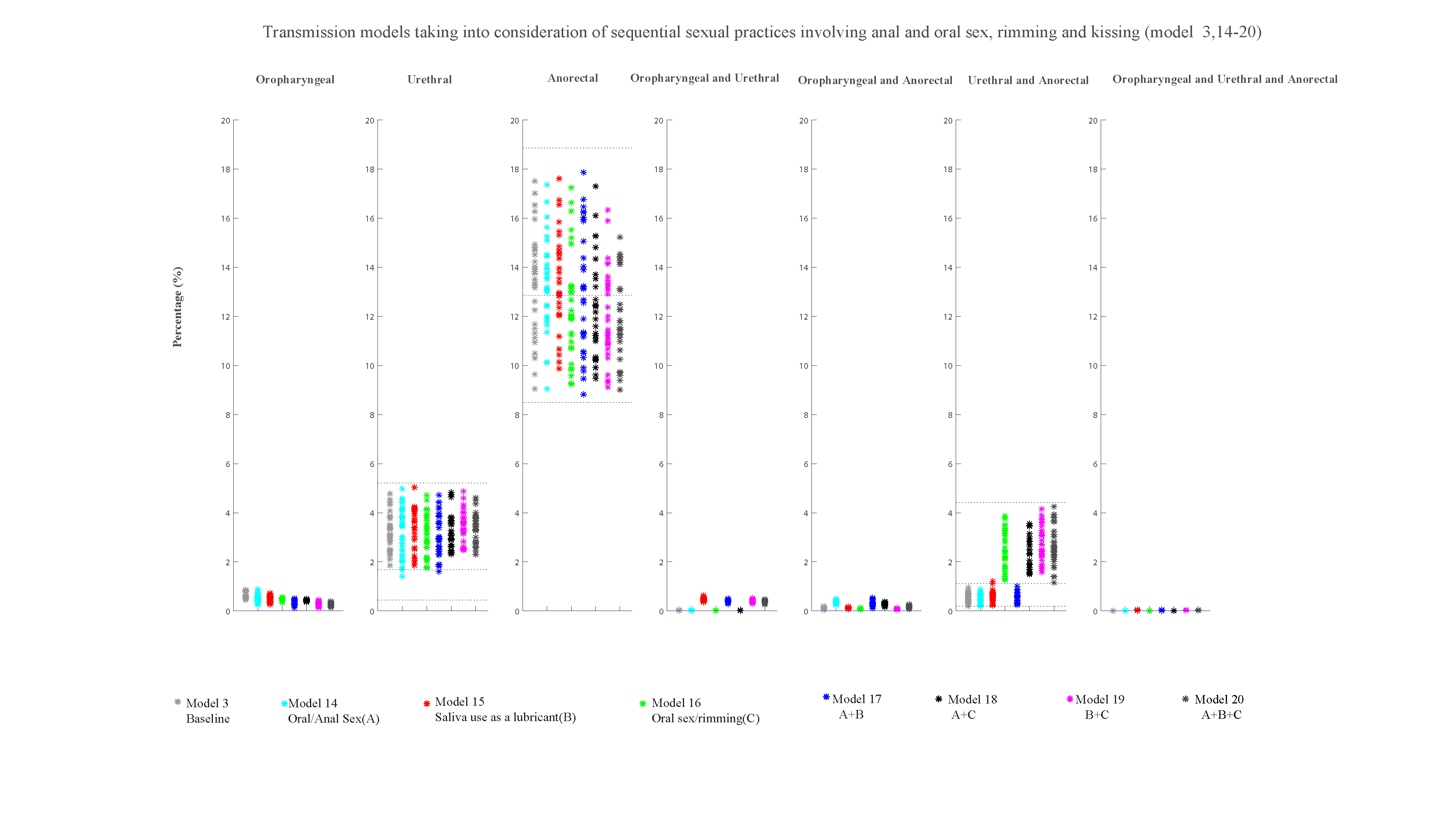
**

**Figure S25a.** Estimates of the eight models for the percentage of specific anatomical sites positive for *Chlamydia trachomatis* for the 8 models (model 3,14-20) and the 95% confidence intervals for the observed site-specific positivity among179 MSM with HIV

**
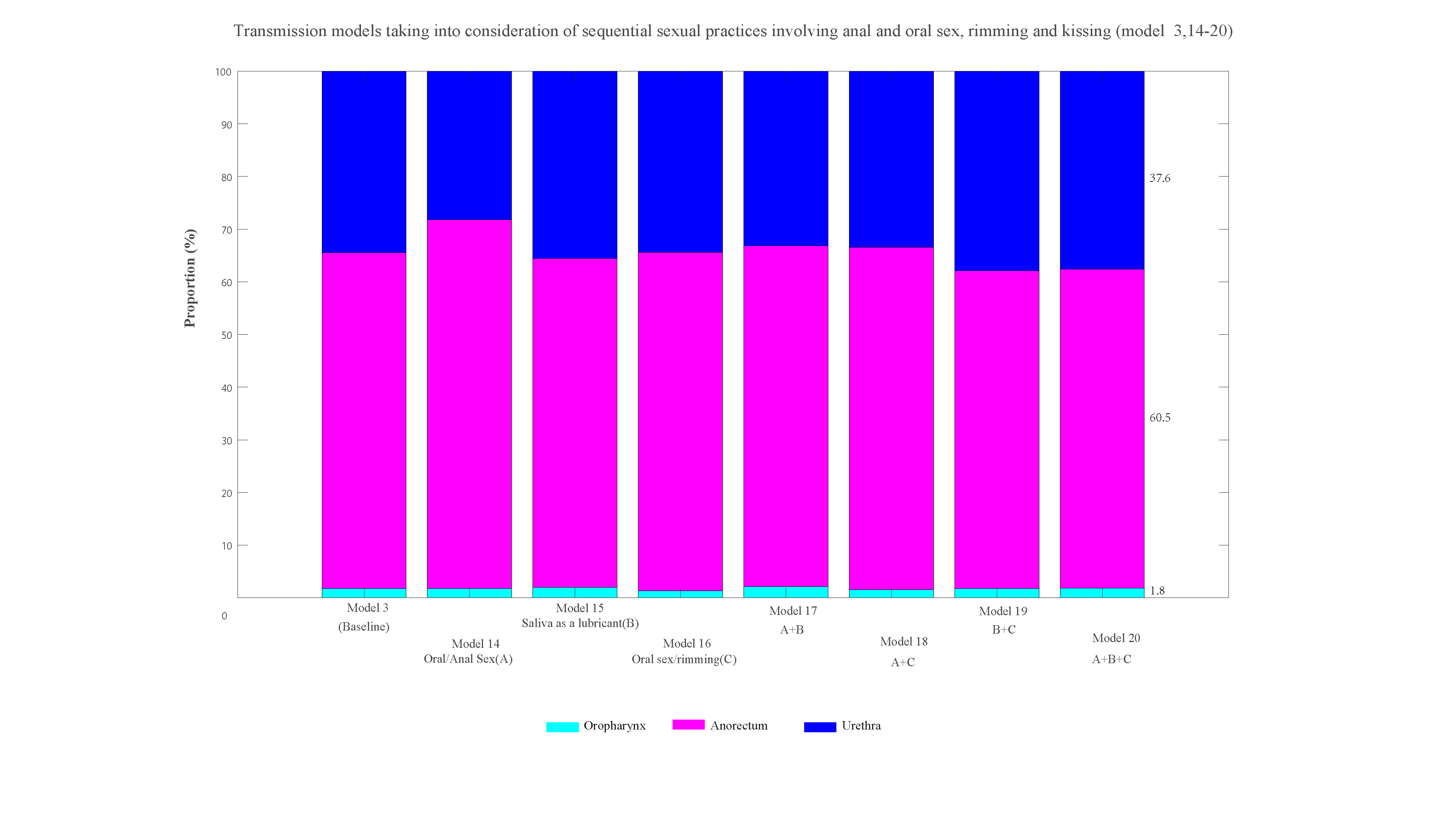
**

**Figure S25b.** Estimated proportion of incident *Chlamydia trachomatis* cases that occur at the oropharynx, anorectum or urethra in MSM from the 8 models (model 3, 14-20) among179 MSM with HIV

**
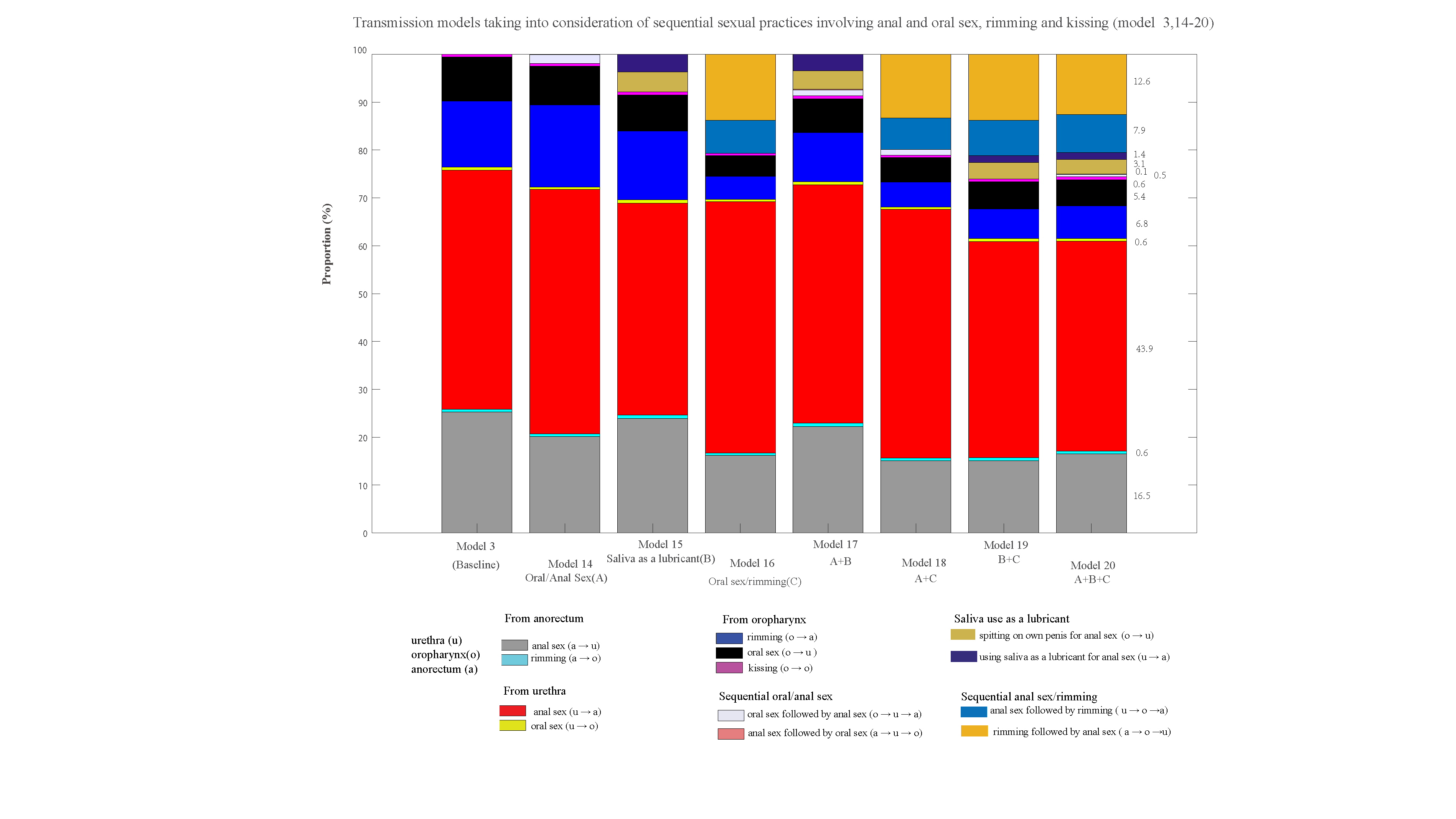
**

**Figure S25c.** Estimated proportion of incident *Chlamydia trachomatis* cases caused by sexual practices in MSM from the 8 models (model 3, 14-20) among179 MSM with HIV

**Supplementary results: Sensitivity analysis**

**Sensitivity analysis: half-length of asymptomatic urethral and anal duration**


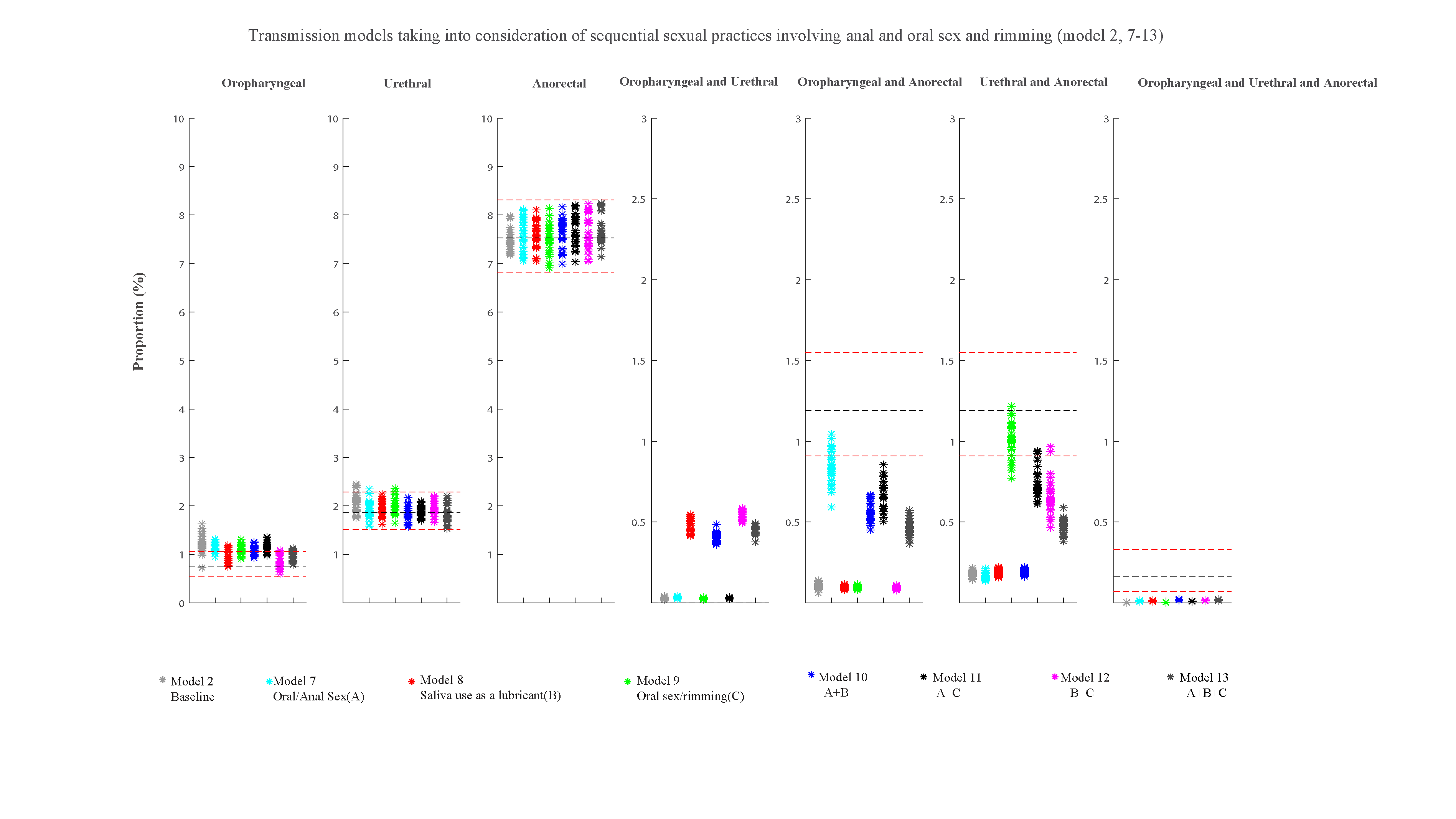


**Figure S26a.** Estimates of the eight models for the percentage of specific anatomical sites positive for *Chlamydia trachomatis* for the eight models (model 2, 7-13) and the 95% confidence intervals for the observed site-specific positivity among 4888 MSM attending Melbourne Sexual Health Centre in 2018 and 2019: half-length of asymptomatic urethral and anal duration


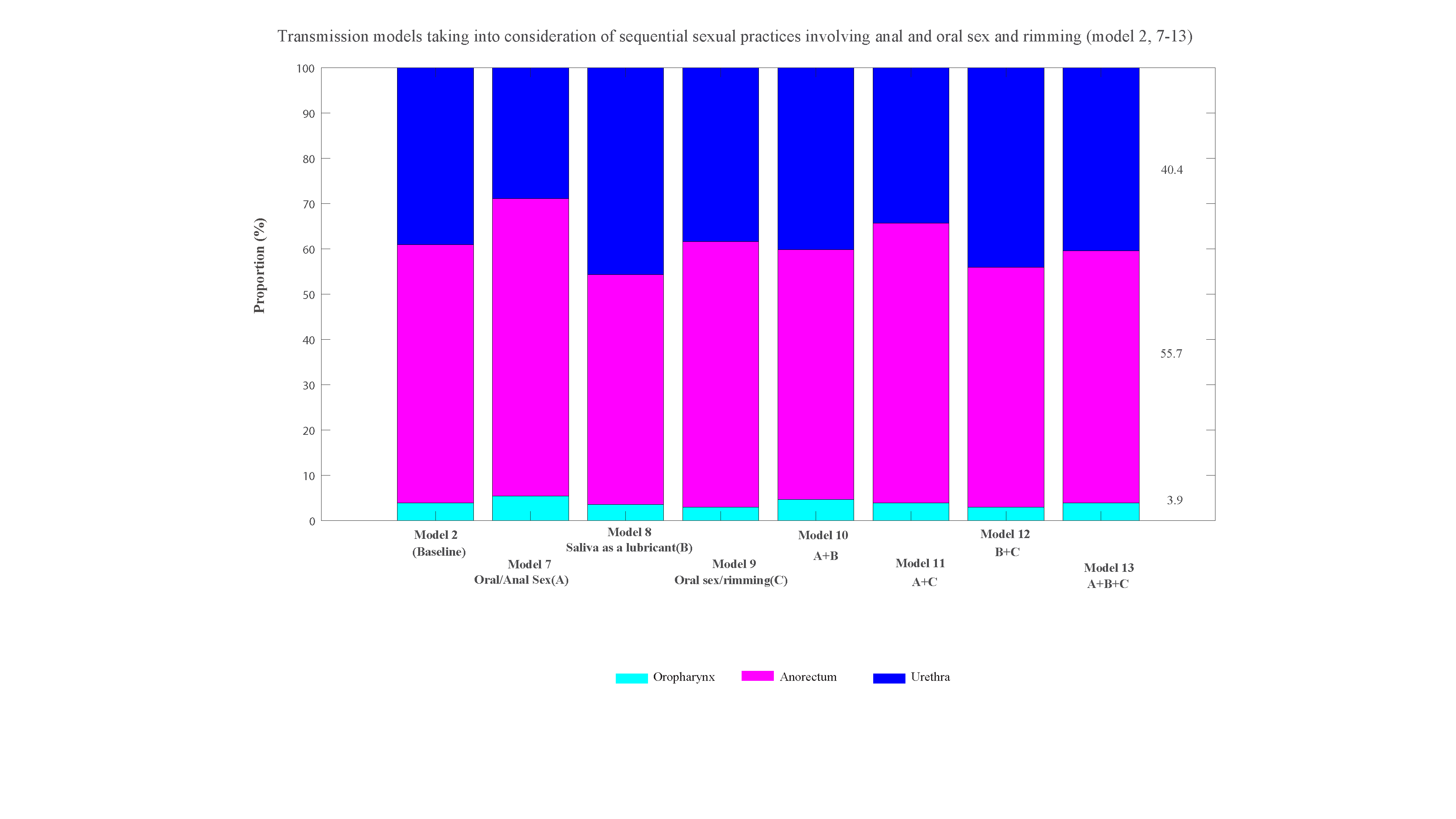


**Figure S26b.** Estimated proportion of incident *Chlamydia trachomatis* cases that occur at the oropharynx, anorectum or urethra in MSM from the eight models (model 2, 7-13) among 4888 MSM attending Melbourne Sexual Health Centre in 2018 and 2019: half-length of asymptomatic urethral and anal duration


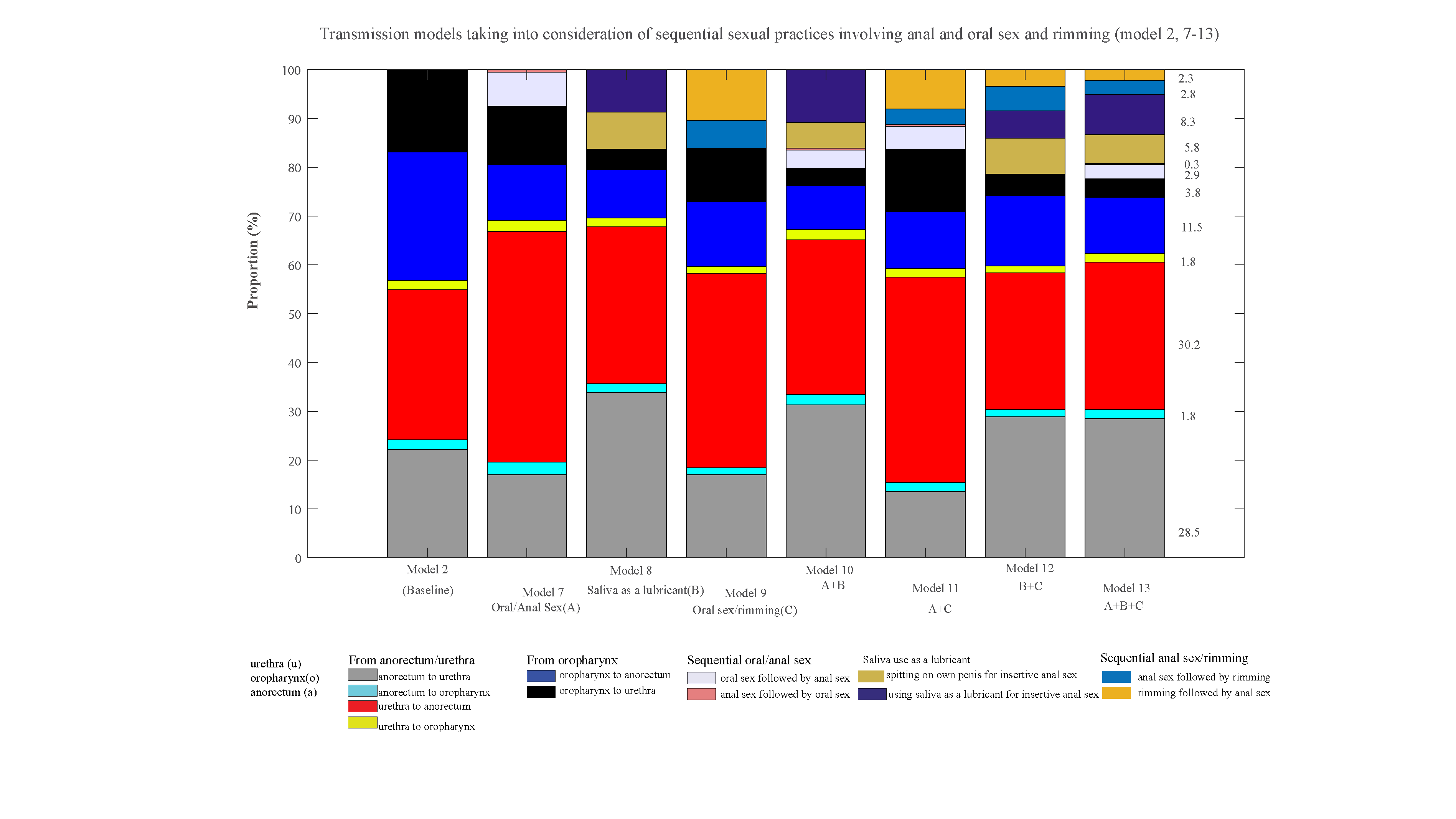


**Figure S26c.** Estimated proportion of incident *Chlamydia trachomatis* cases caused by sexual practices in MSM from the eight models (model 2, 7-13) among 4888 MSM attending Melbourne Sexual Health Centre in 2018 and 2019: half-length of asymptomatic urethral and anal duration

Sensitivity analysis: double days of sexual practices


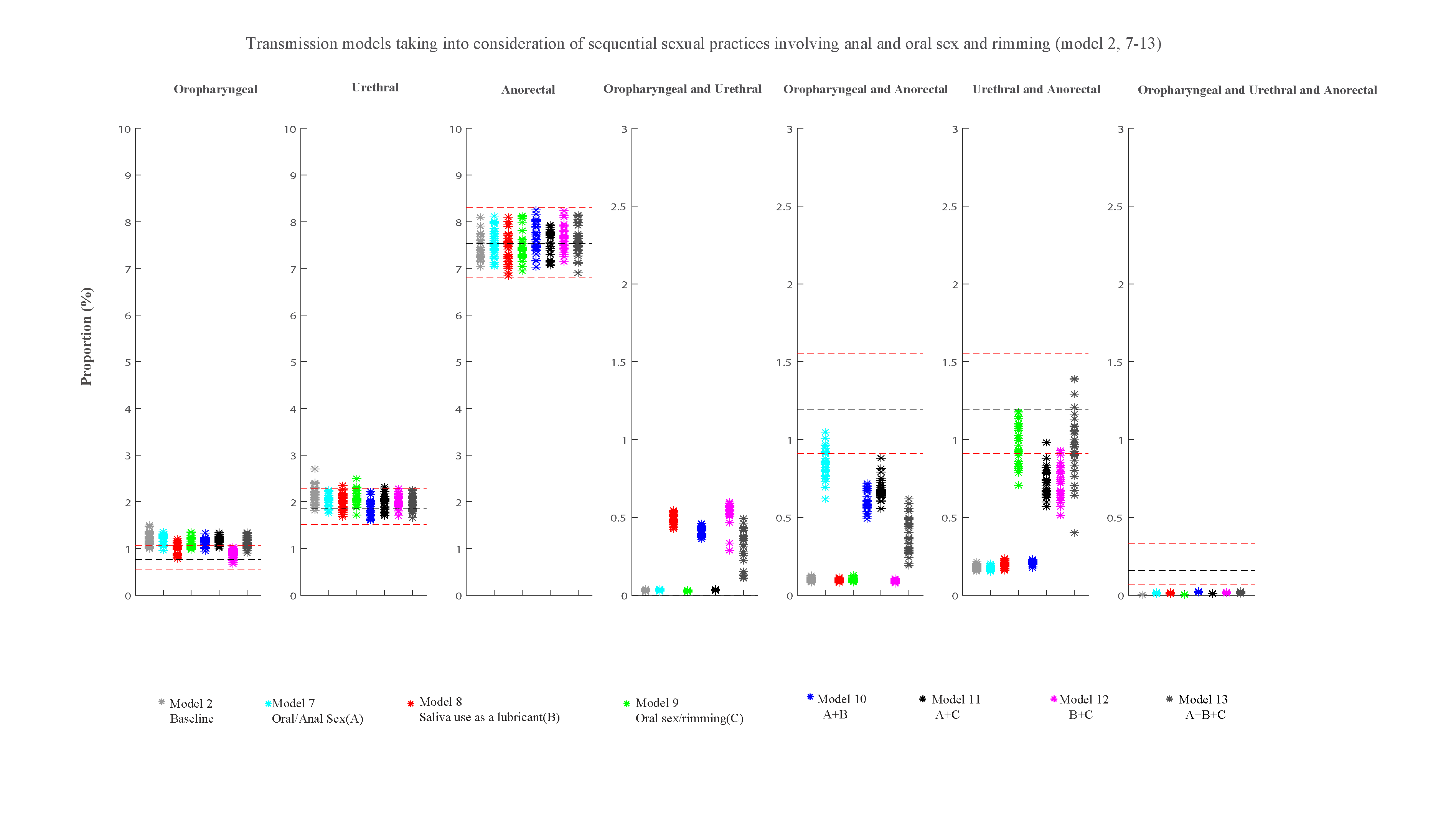


**Figure S27a.** Estimates of the eight models for the percentage of specific anatomical sites positive for *Chlamydia trachomatis* for the eight models (model 2, 7-13) and the 95% confidence intervals for the observed site-specific positivity among 4888 MSM attending Melbourne Sexual Health Centre in 2018 and 2019: double days of sexual practices


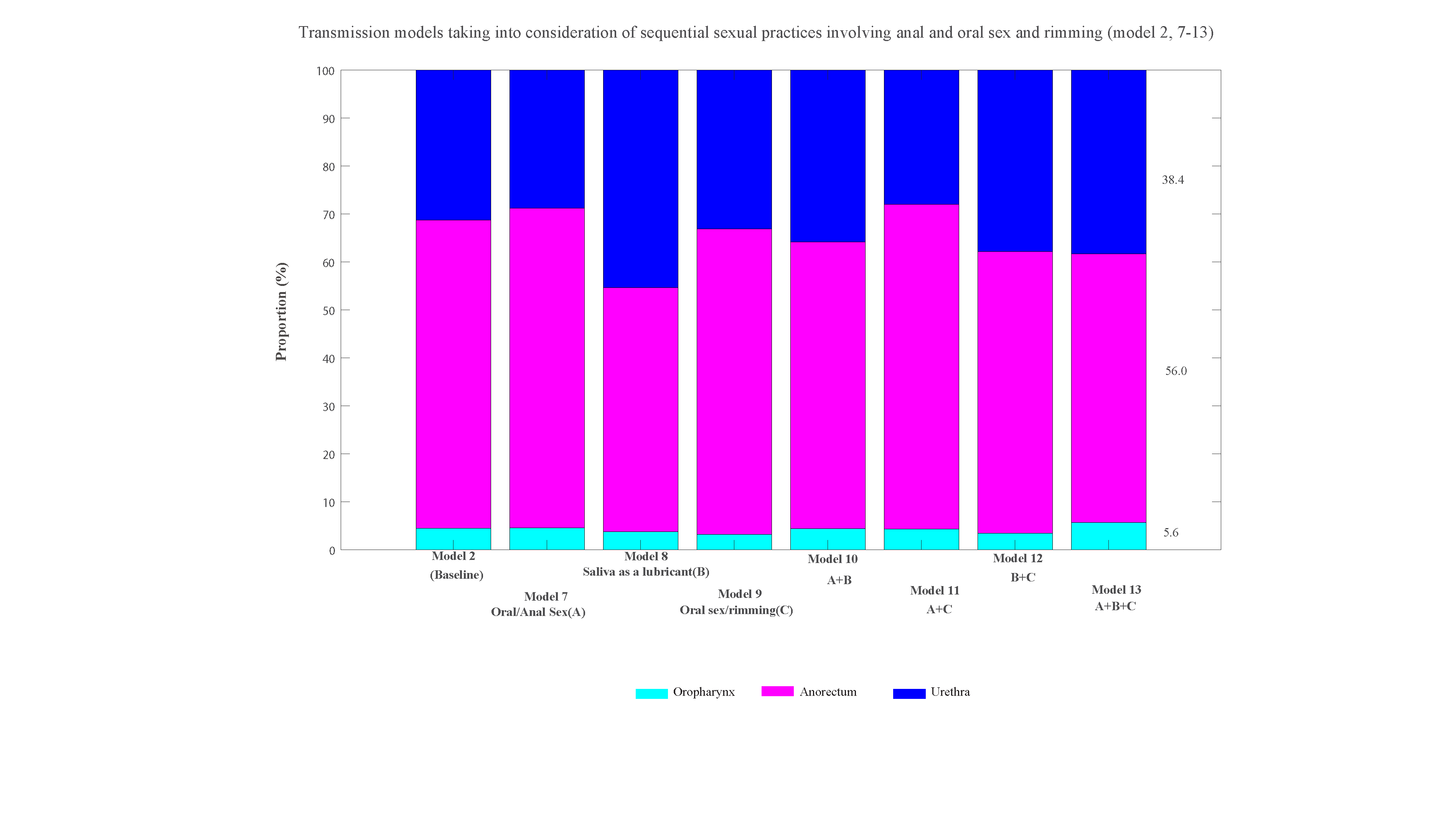


**Figure S27b.** Estimated proportion of incident *Chlamydia trachomatis* cases that occur at the oropharynx, anorectum or urethra in MSM from the eight models (model 2, 7-13) among 4888 MSM attending Melbourne Sexual Health Centre in 2018 and 2019：double days of sexual practices


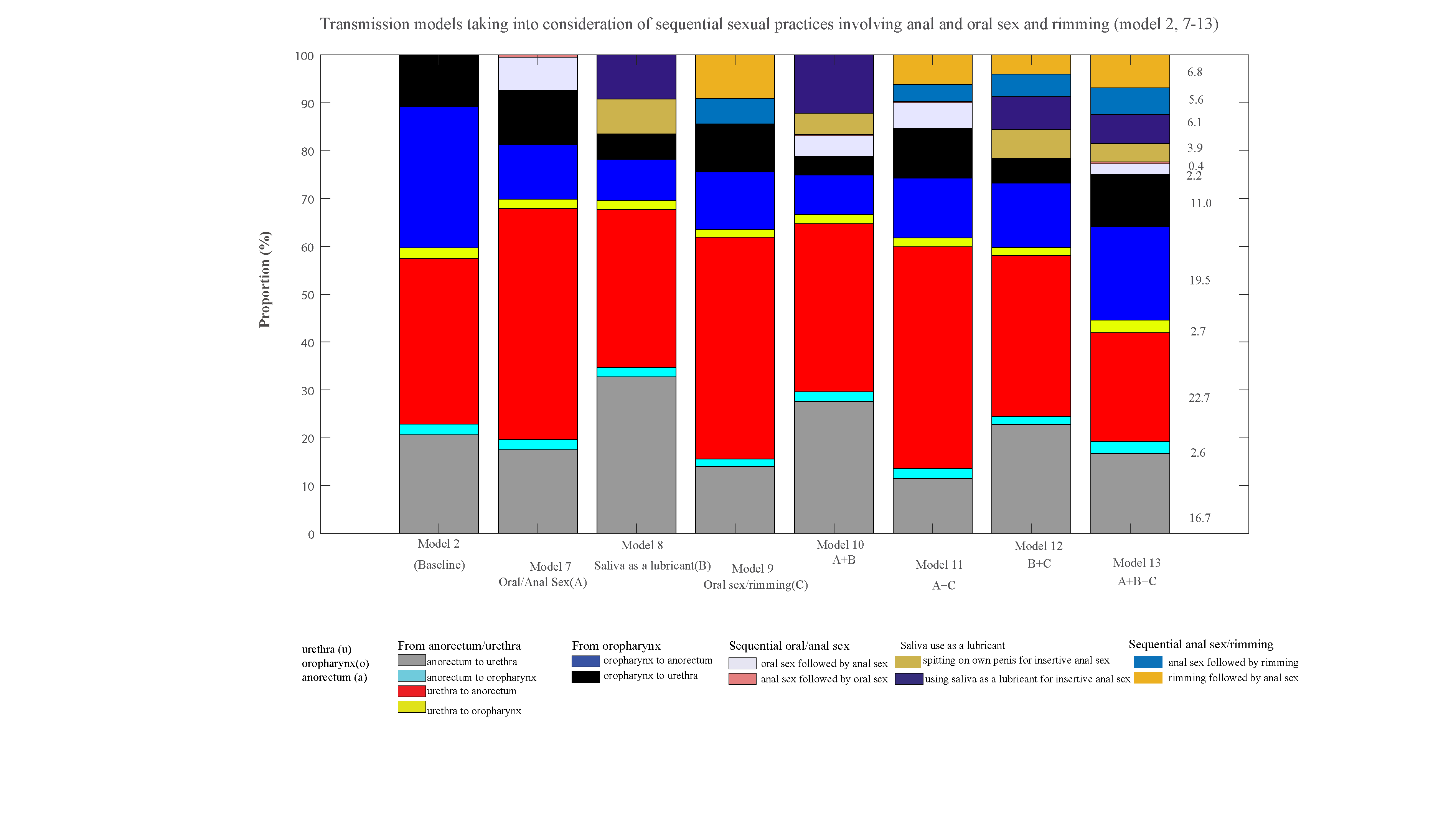


**Figure S27c**. Estimated proportion of incident *Chlamydia trachomatis* cases caused by sexual practices in MSM from the eight models (model 2, 7-13) among 4888 MSM attending Melbourne Sexual Health Centre in 2018 and 2019：double days of sexual practices

**Sensitivity analysis: half days of sexual practices**


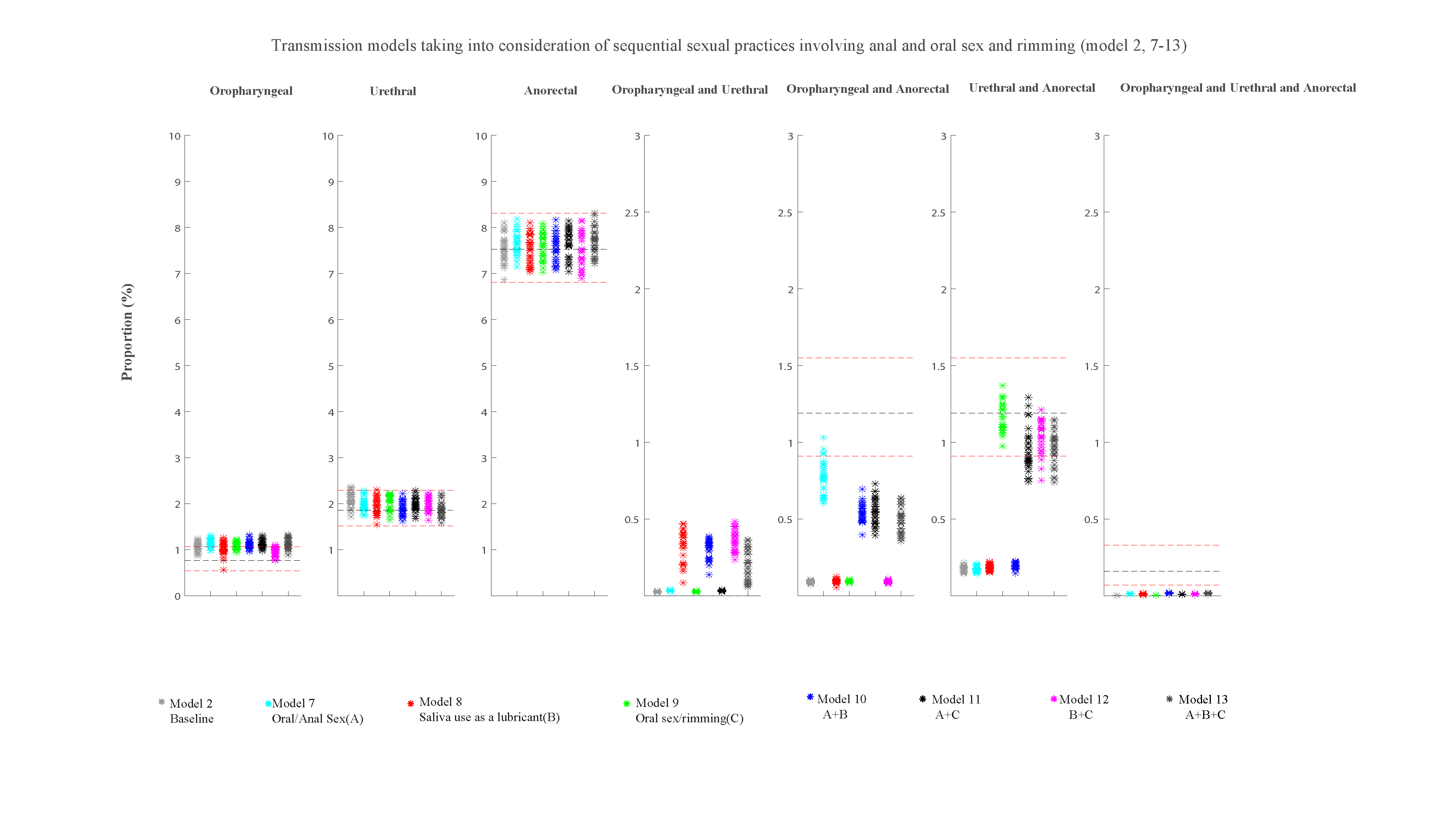


**Figure S28a.** Estimates of the eight models for the percentage of specific anatomical sites positive for *Chlamydia trachomatis* for the eight models (model 2, 7-13) and the 95% confidence intervals for the observed site-specific positivity among 4888 MSM attending Melbourne Sexual Health Centre in 2018 and 2019: half days of sexual practices


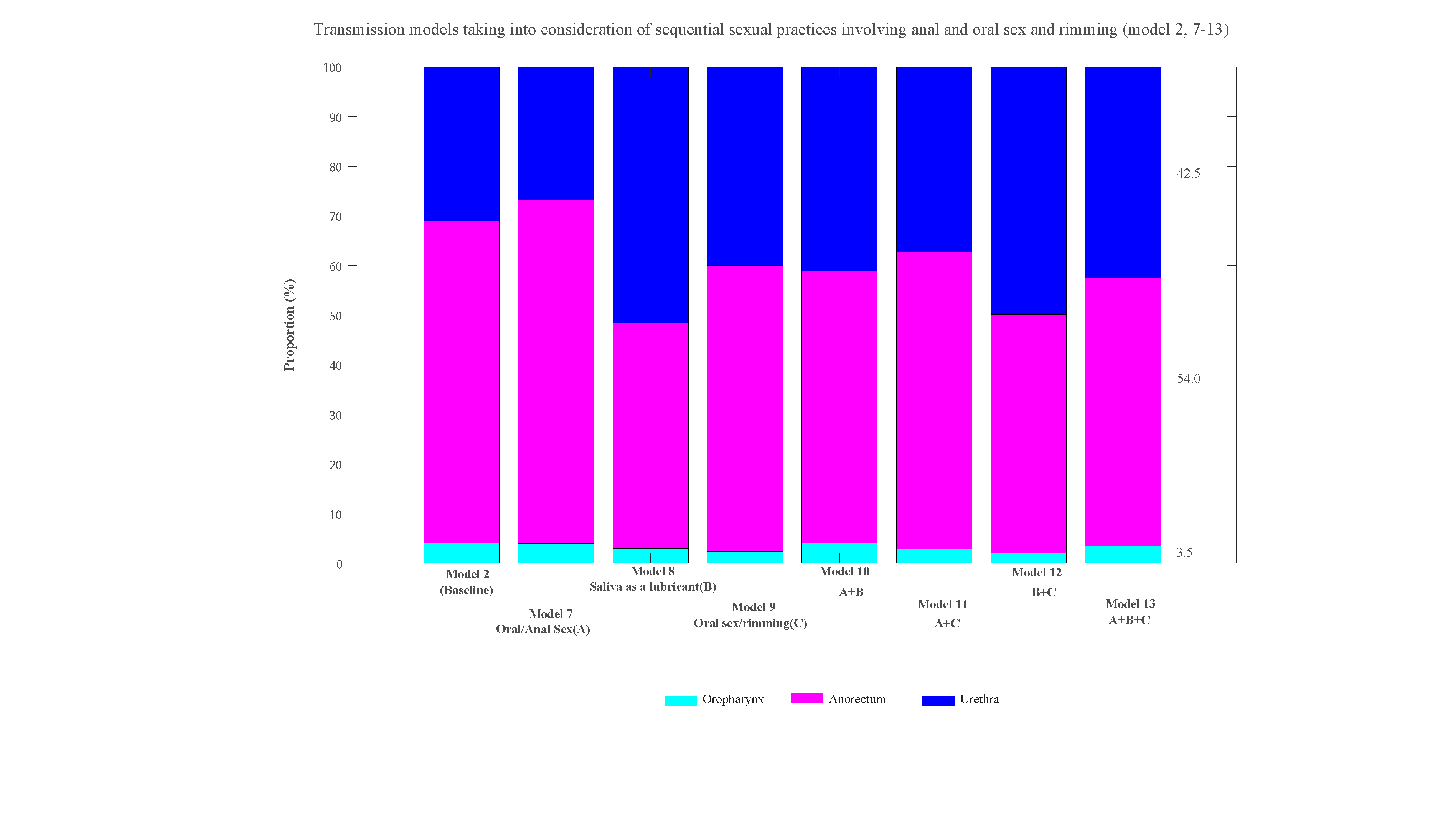


**Figure S28b**. Estimated proportion of incident *Chlamydia trachomatis* cases that occur at the oropharynx, anorectum or urethra in MSM from the eight models (model 2, 7-13) among 4888 MSM attending Melbourne Sexual Health Centre in 2018 and 2019：half days of sexual practices


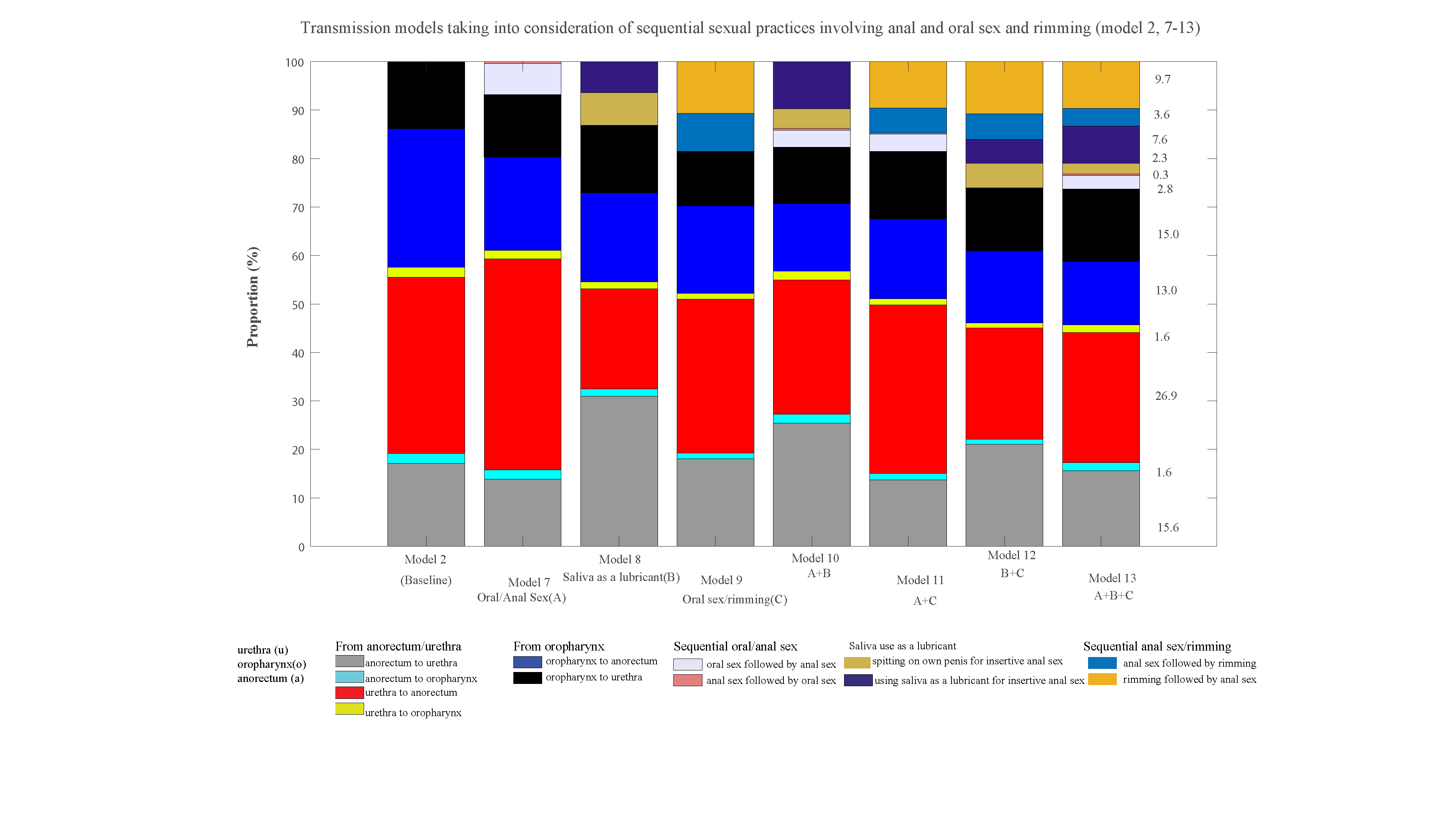


**Figure S28c.** Estimated proportion of incident *Chlamydia trachomatis* cases caused by sexual practices in MSM from the eight models (model 2, 7-13) among 4888 MSM attending Melbourne Sexual Health Centre in 2018 and 2019：half days of sexual practices

Sensitivity analysis: The proportion of sequential sexual practices = 0%


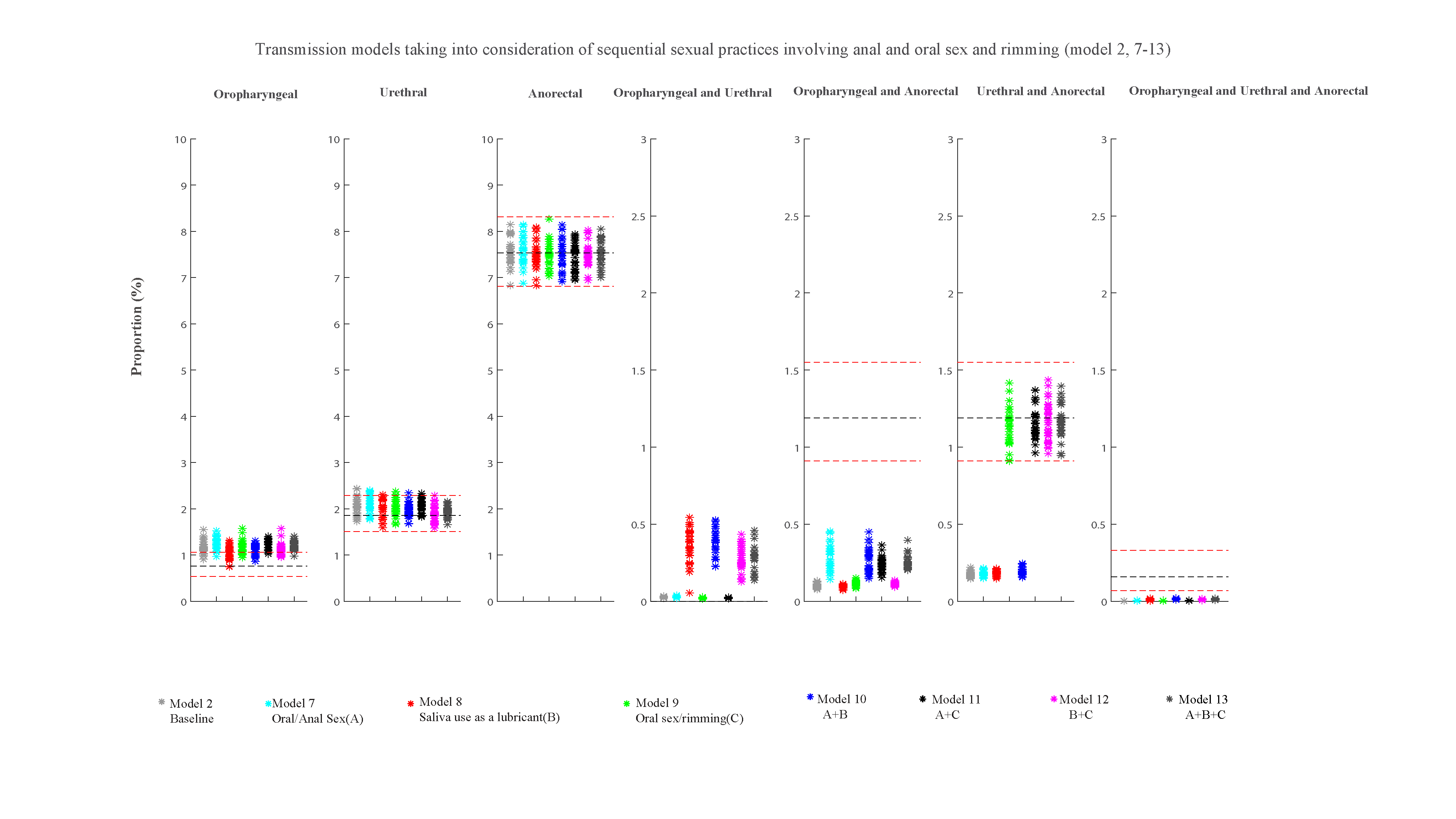


**Figure S29a.** Estimates of the eight models for the percentage of specific anatomical sites positive for *Chlamydia trachomatis* for the eight models (model 2, 7-13) and the 95% confidence intervals for the observed site-specific positivity among 4888 MSM attending Melbourne Sexual Health Centre in 2018 and 2019: the proportion of sequential sexual practices = 0%


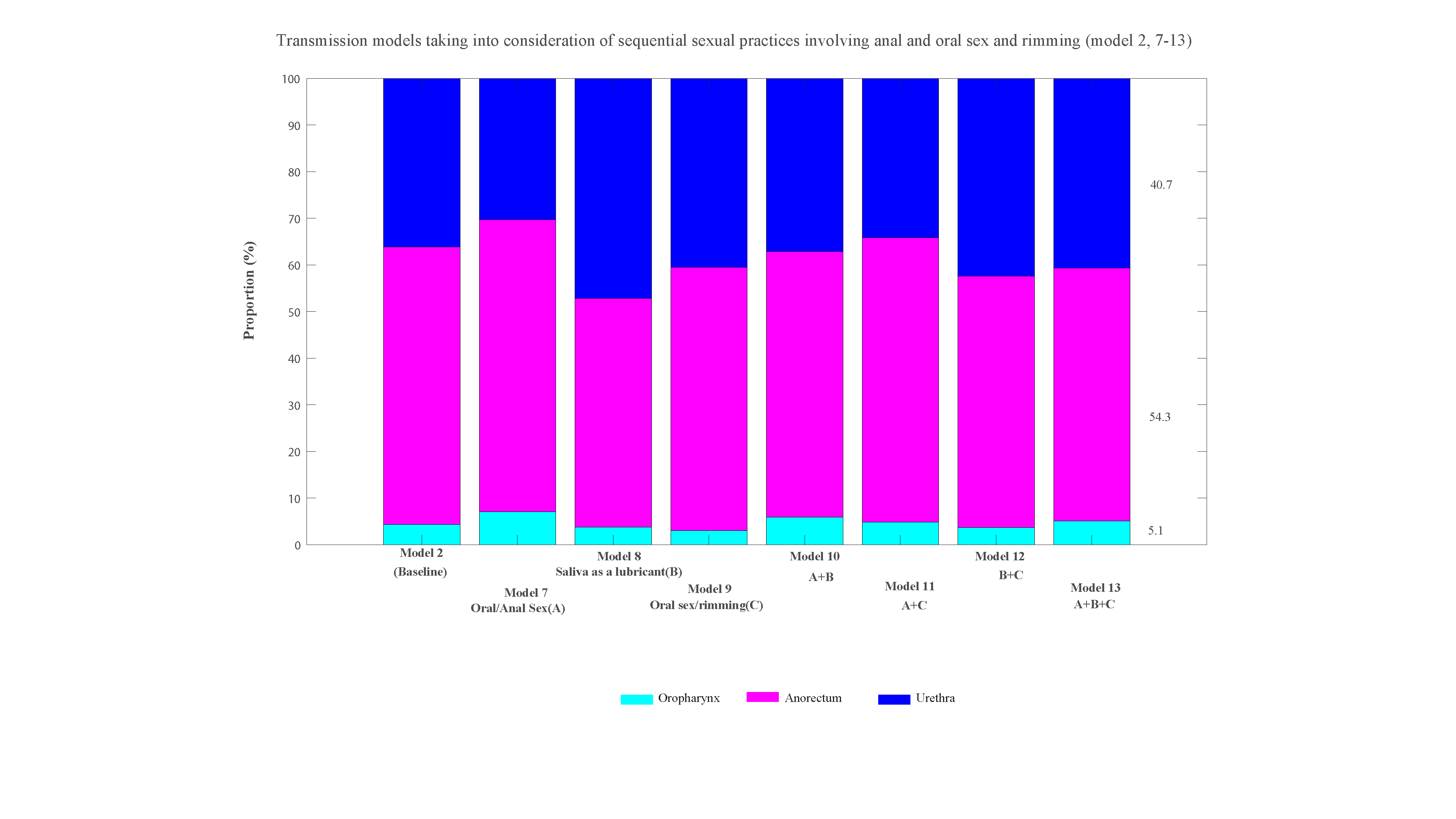


**Figure S29b.** Estimated proportion of incident *Chlamydia trachomatis* cases that occur at the oropharynx, anorectum or urethra in MSM from the eight models (model 2, 7-13) among 4888 MSM attending Melbourne Sexual Health Centre in 2018 and 2019：the proportion of sequential sexual practices = 0%


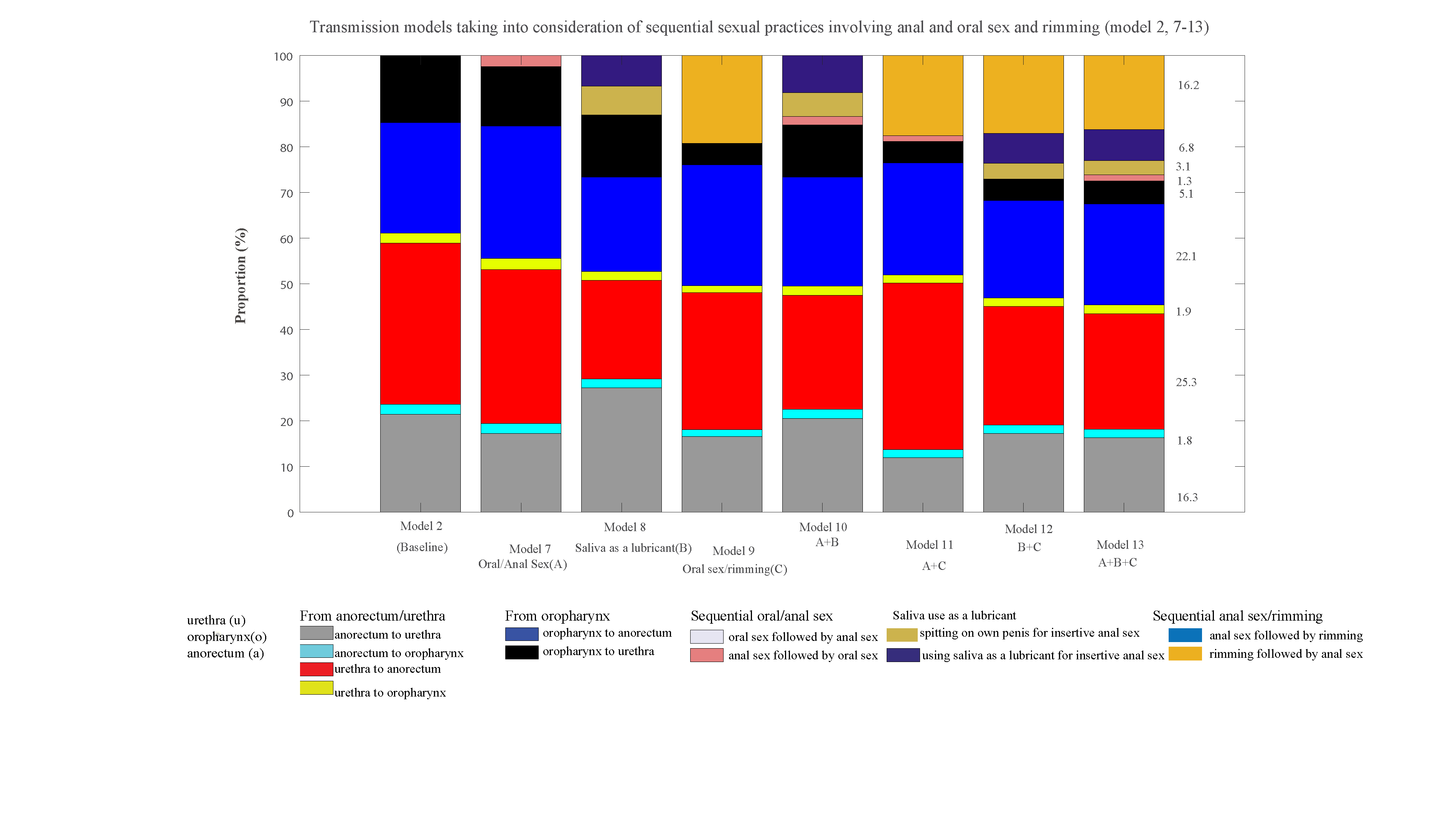


**Figure S29c.** Estimated proportion of incident *Chlamydia trachomatis* cases caused by sexual practices in MSM from the eight models (model 2, 7-13) among 4888 MSM attending Melbourne Sexual Health Centre in 2018 and 2019：the proportion of sequential sexual practices = 0%

Sensitivity analysis: The proportion of sequential sexual practices = 95%


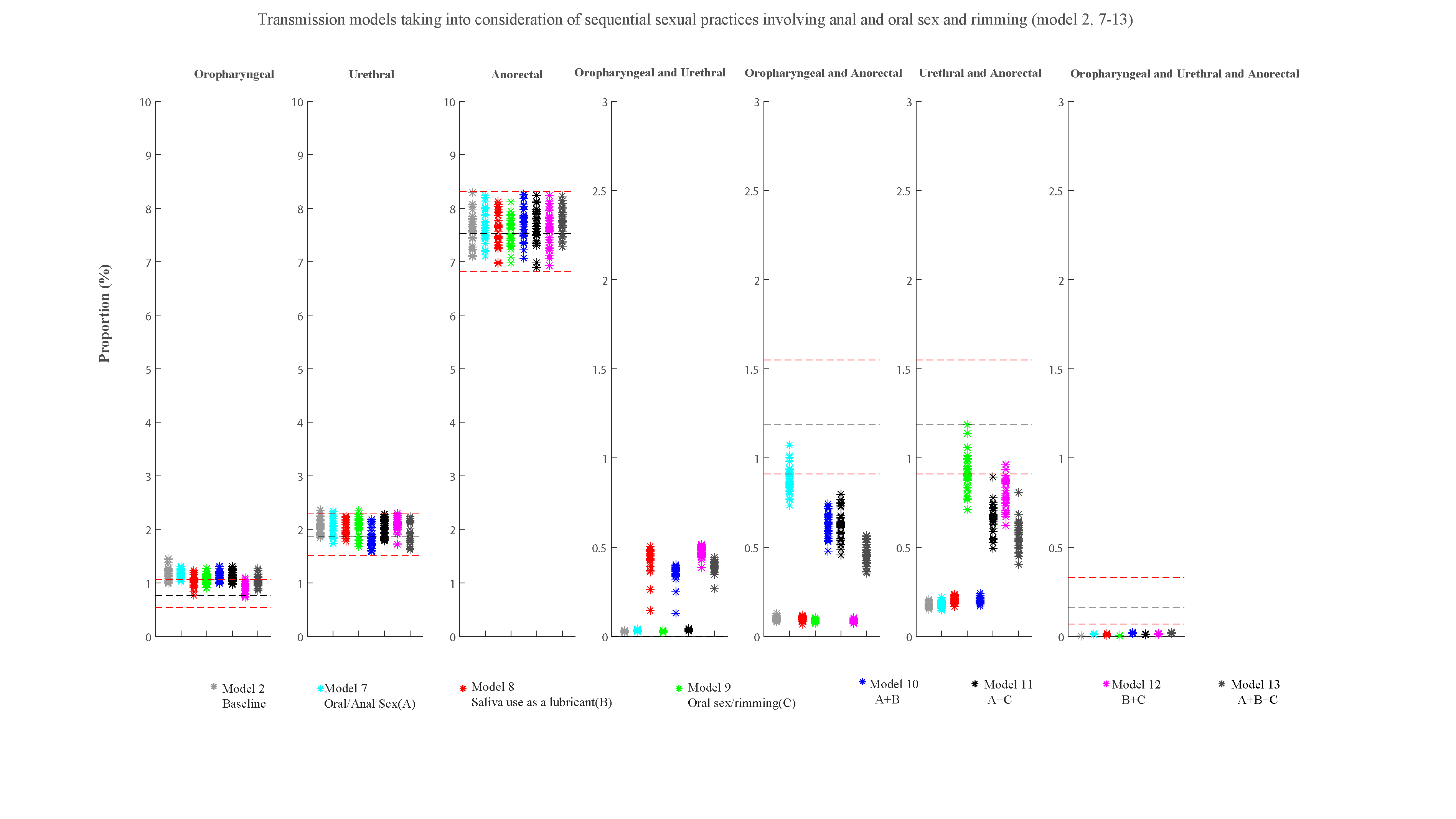


**Figure S30a.** Estimates of the eight models for the percentage of specific anatomical sites positive for *Chlamydia trachomatis* for the eight models (model 2, 7-13) and the 95% confidence intervals for the observed site-specific positivity among 4888 MSM attending Melbourne Sexual Health Centre in 2018 and 2019: the proportion of sequential sexual practices = 95%


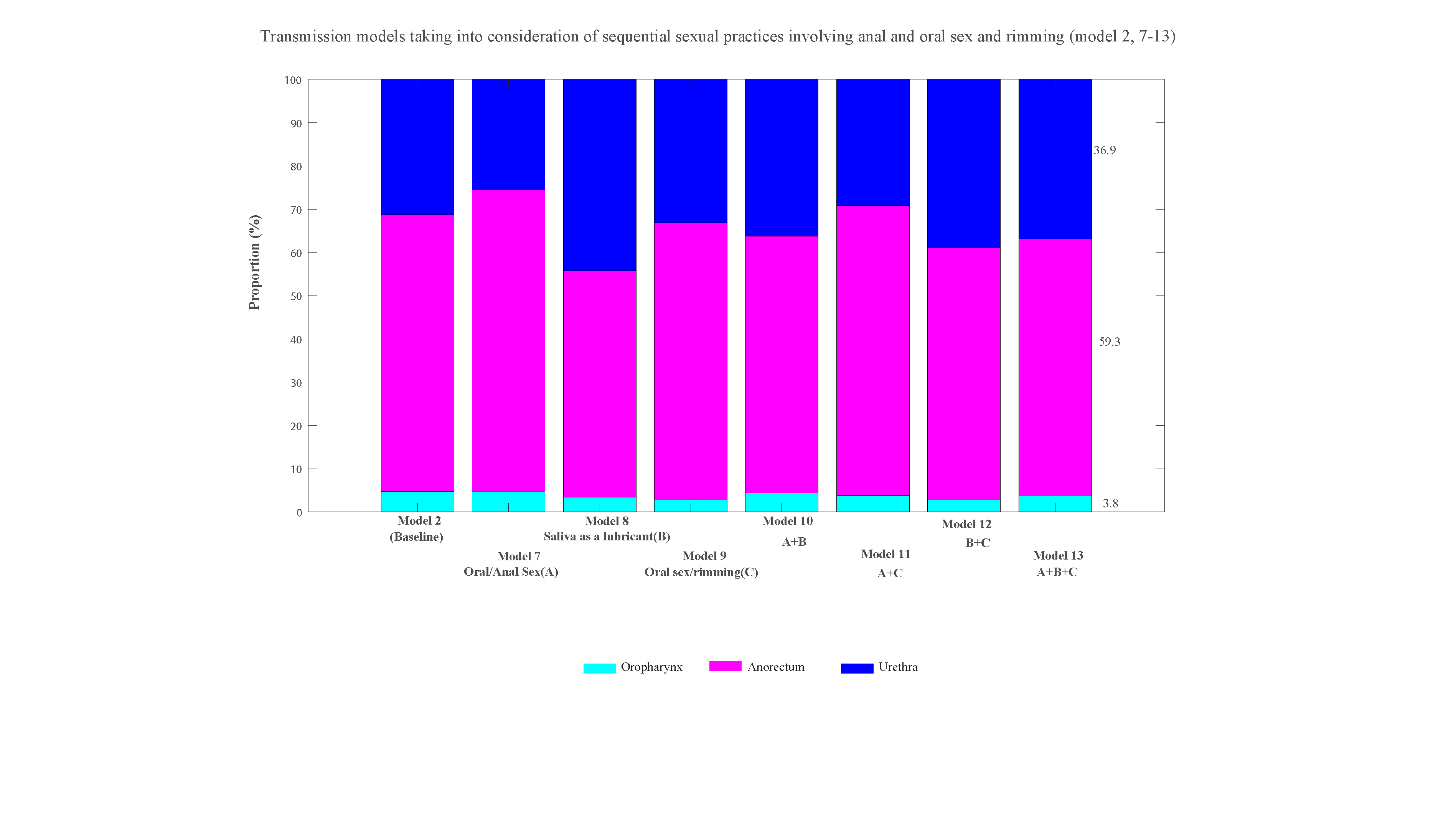


**Figure S30b.** Estimated proportion of incident *Chlamydia trachomatis* cases that occur at the oropharynx, anorectum or urethra in MSM from the eight models (model 2, 7-13) among 4888 MSM attending Melbourne Sexual Health Centre in 2018 and 2019: the proportion of sequential sexual practices = 95%


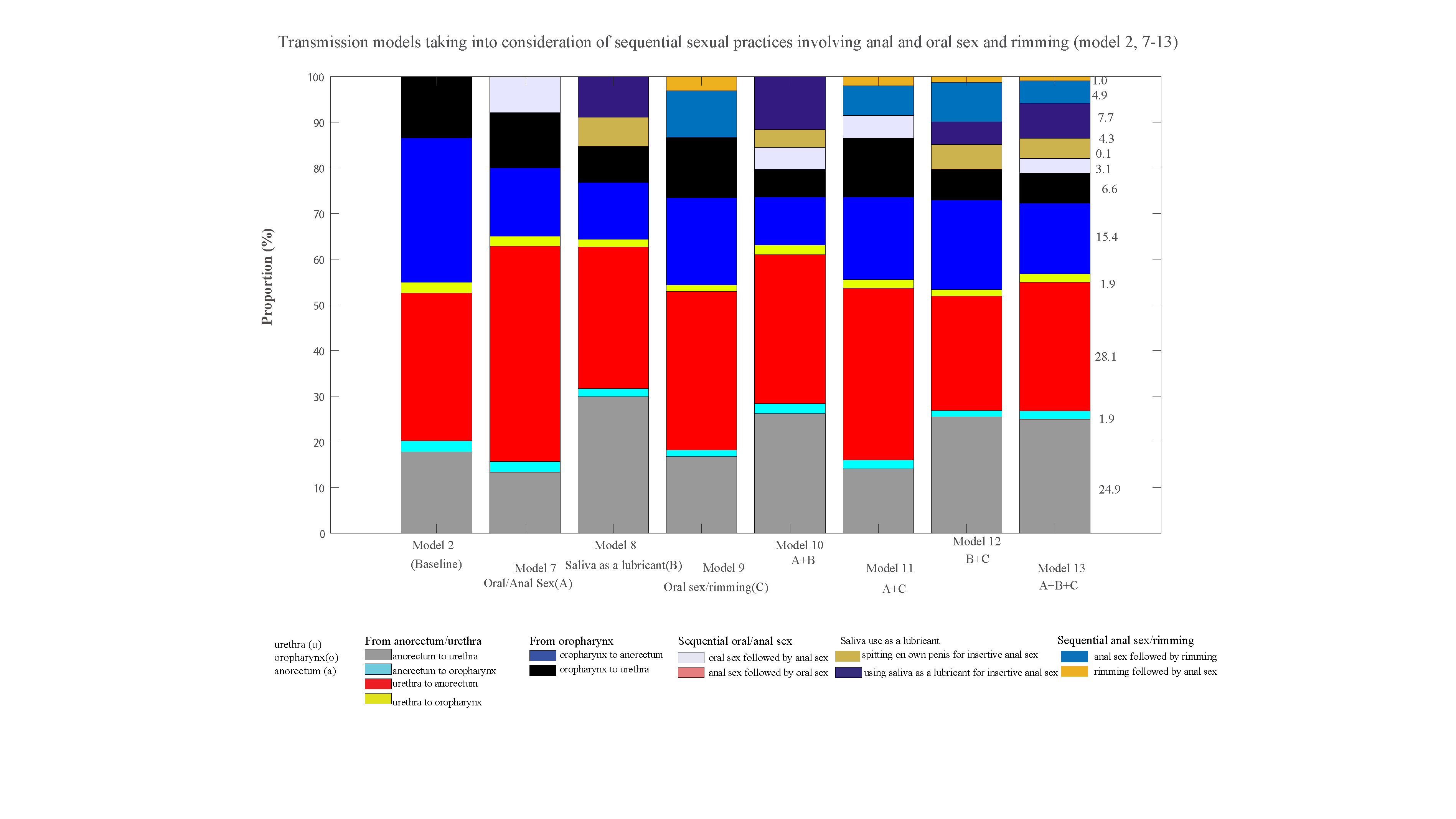


**Figure S30c.** Estimated proportion of incident *Chlamydia trachomatis* cases caused by sexual practices in MSM from the eight models (model 2, 7-13) among 4888 MSM attending Melbourne Sexual Health Centre in 2018 and 2019：the proportion of sequential sexual practices = 95%

**Sensitivity analysis: The proportion of sequential sexual practices = 98%**


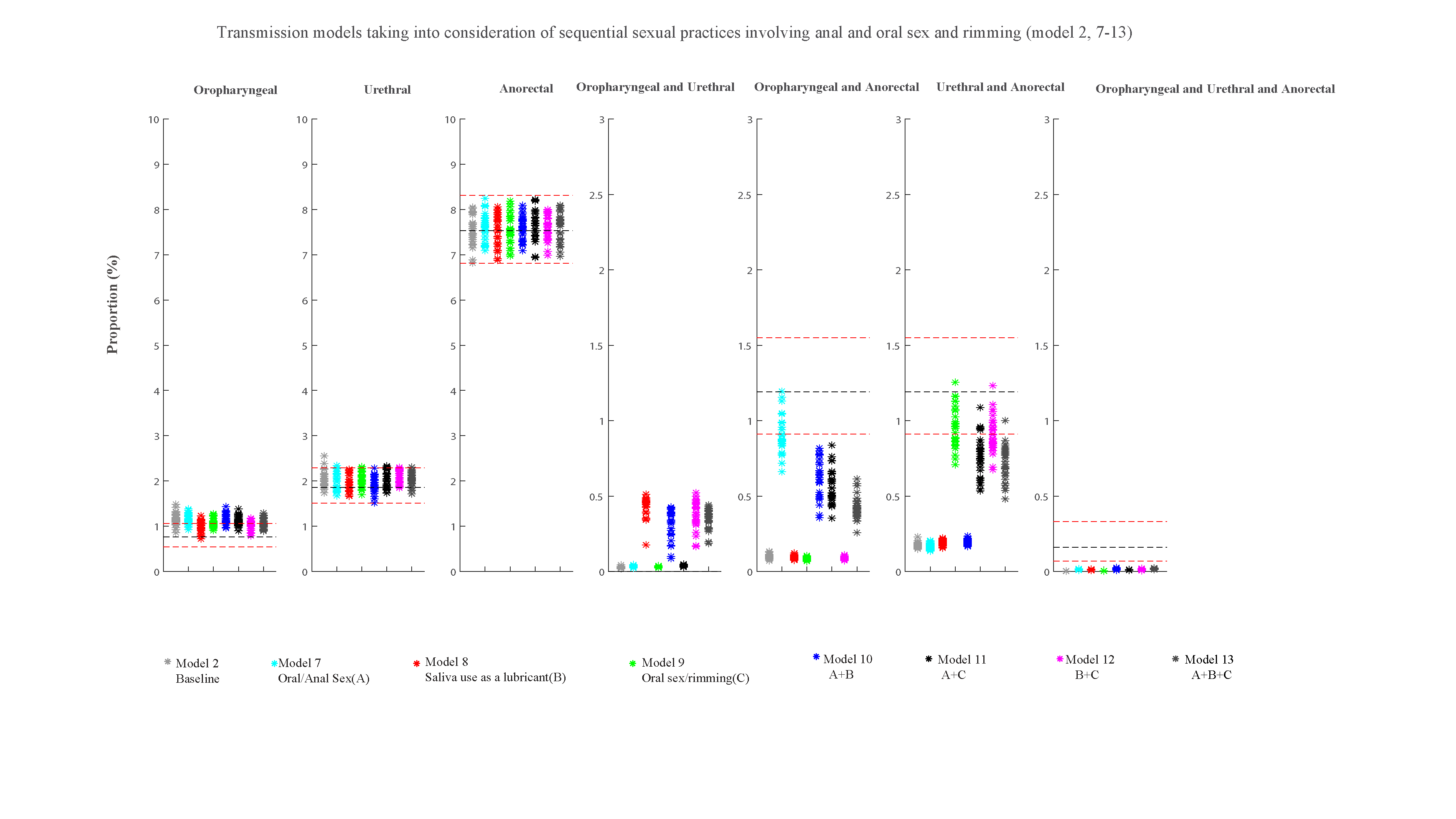


**Figure S31a.** Estimates of the eight models for the percentage of specific anatomical sites positive for *Chlamydia trachomatis* for the eight models (model 2, 7-13) and the 95% confidence intervals for the observed site-specific positivity among 4888 MSM attending Melbourne Sexual Health Centre in 2018 and 2019: the proportion of sequential sexual practices = 98%


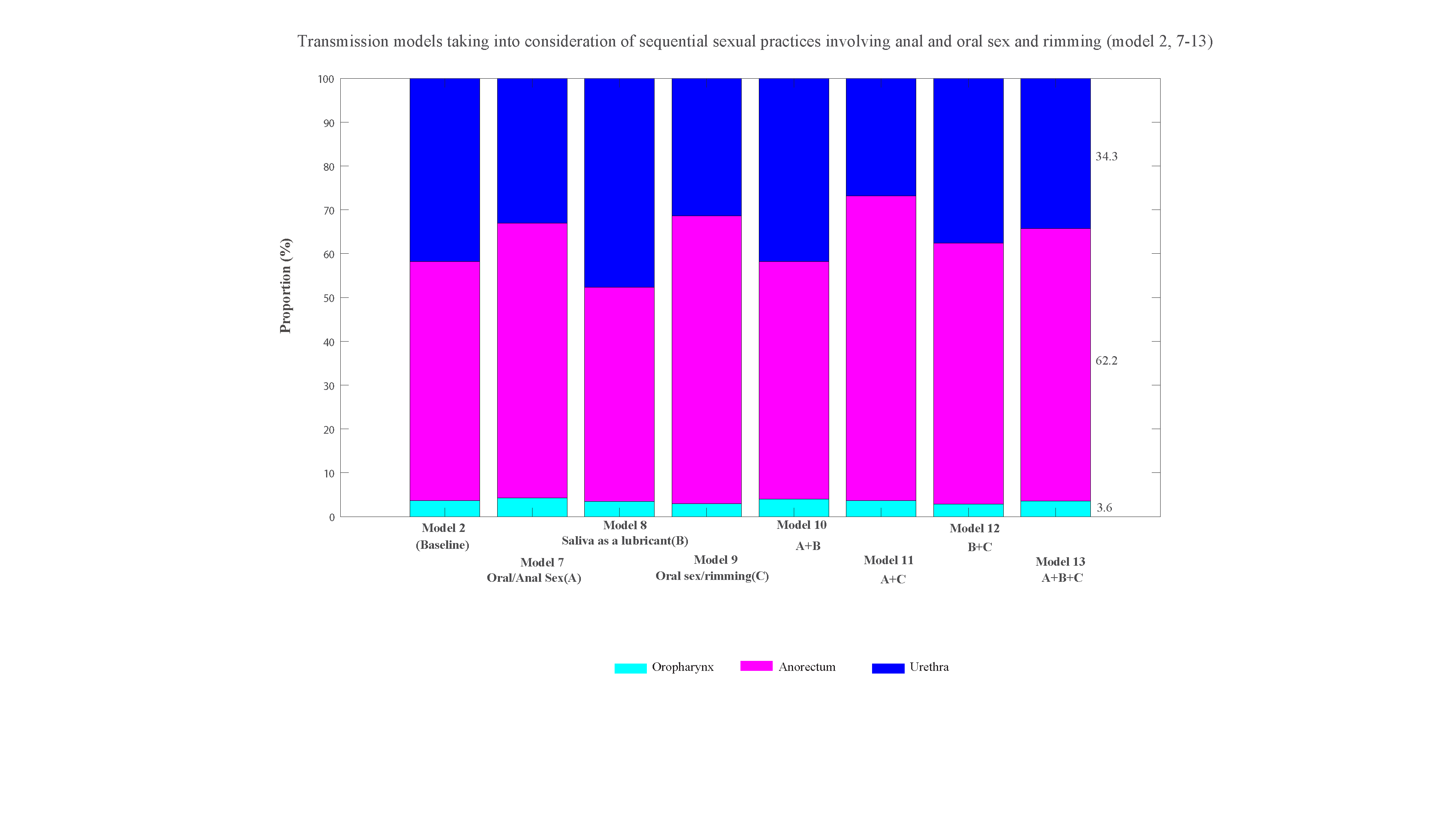


**Figure S31b.** Estimated proportion of incident *Chlamydia trachomatis* cases that occur at the oropharynx, anorectum or urethra in MSM from the eight models (model 2, 7-13) among 4888 MSM attending Melbourne Sexual Health Centre in 2018 and 2019: the proportion of sequential sexual practices = 98%


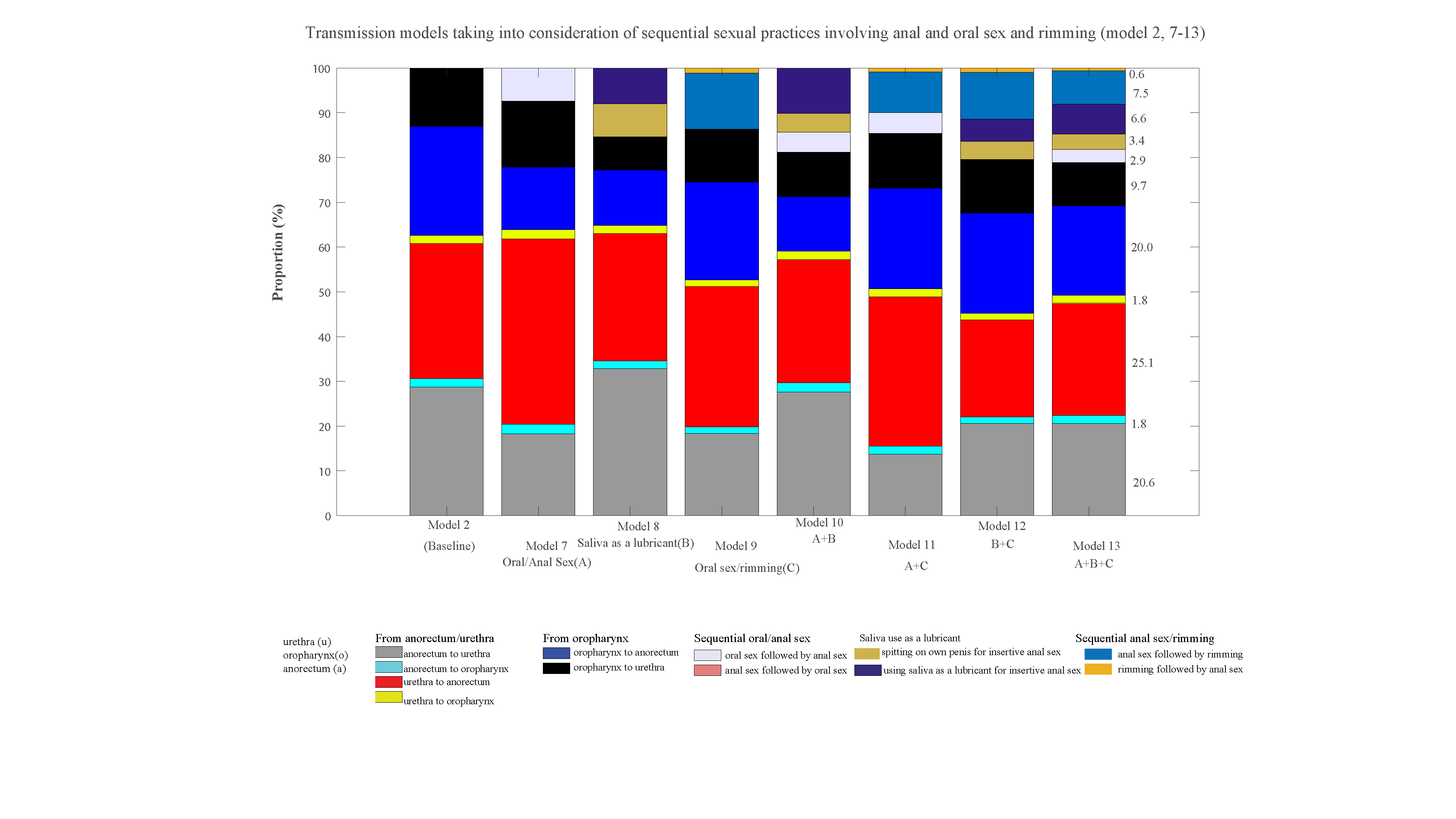


**Figure S31c.** Estimated proportion of incident *Chlamydia trachomatis* cases caused by sexual practices in MSM from the eight models (model 2, 7-13) among 4888 MSM attending Melbourne Sexual Health Centre in 2018 and 2019：the proportion of sequential sexual practices = 98%

**Sensitivity analysis: The proportion of sequential sexual practices = 100%**


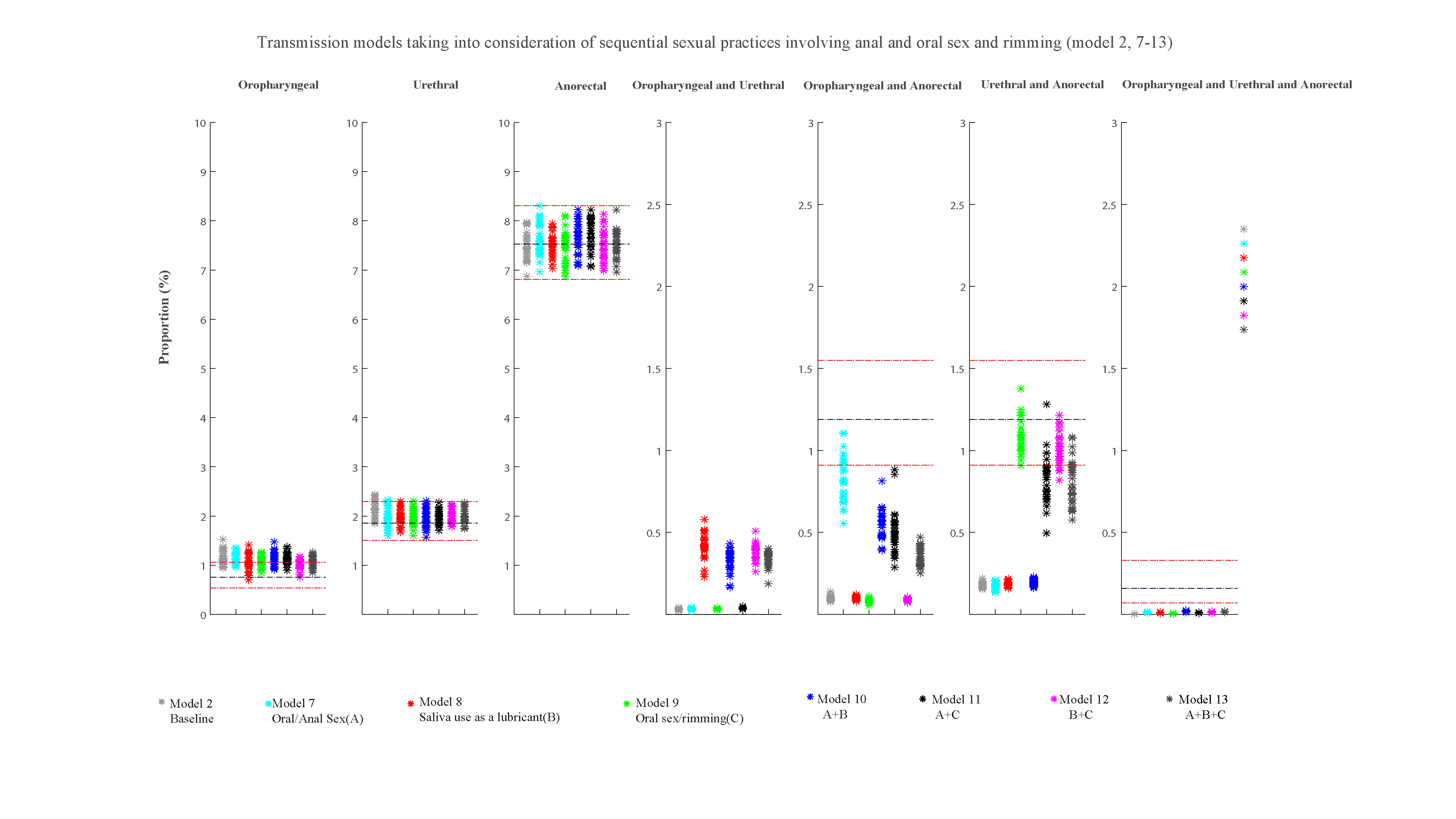


**Figure S32a.** Estimates of the eight models for the percentage of specific anatomical sites positive for *Chlamydia trachomatis* for the eight models (model 2, 7-13) and the 95% confidence intervals for the observed site-specific positivity among 4888 MSM attending Melbourne Sexual Health Centre in 2018 and 2019: the proportion of sequential sexual practices = 100%


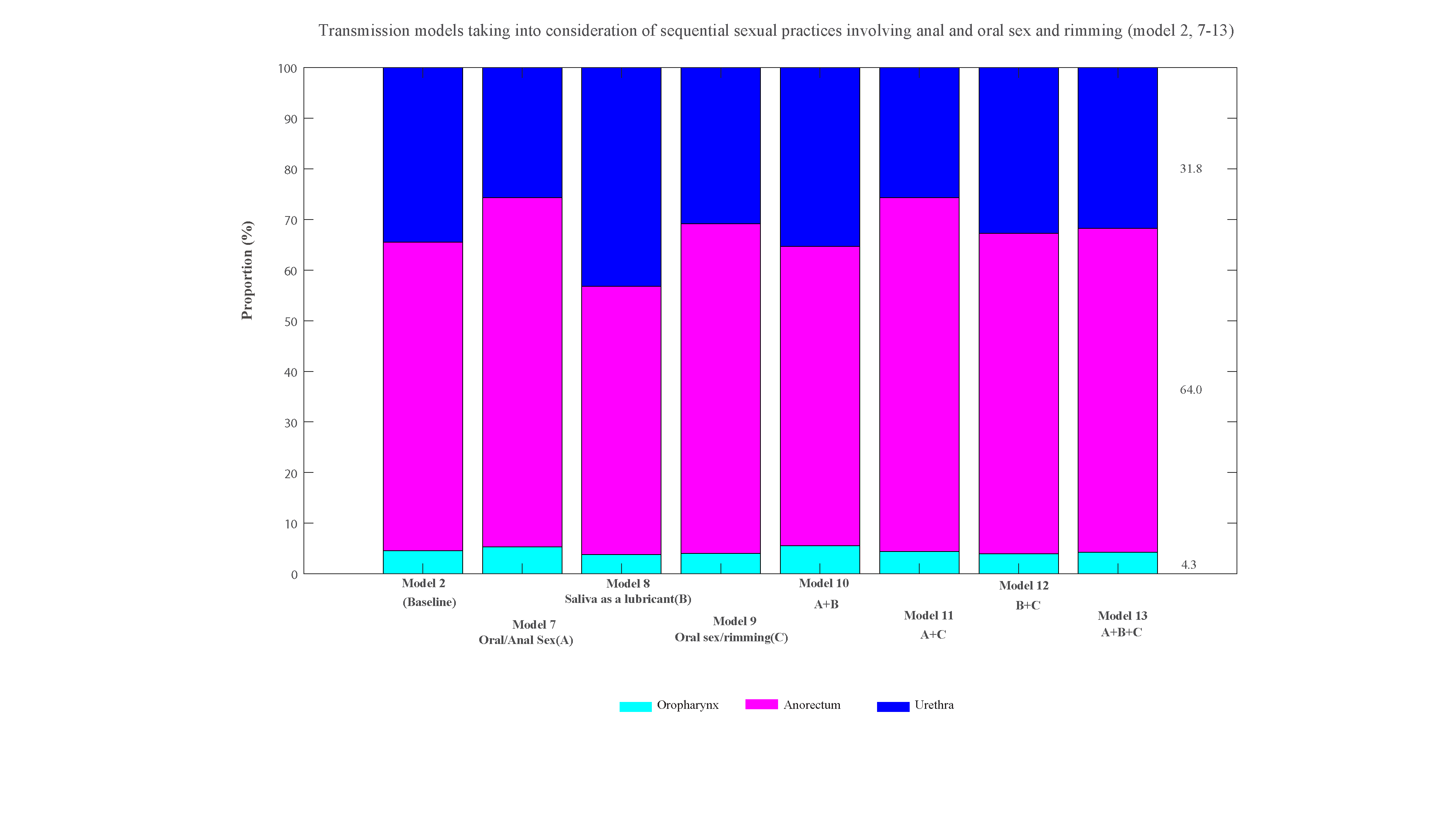


**Figure S32b.** Estimated proportion of incident *Chlamydia trachomatis* cases that occur at the oropharynx, anorectum or urethra in MSM from the eight models (model 2, 7-13) among 4888 MSM attending Melbourne Sexual Health Centre in 2018 and 2019：the proportion of sequential sexual practices = 100%


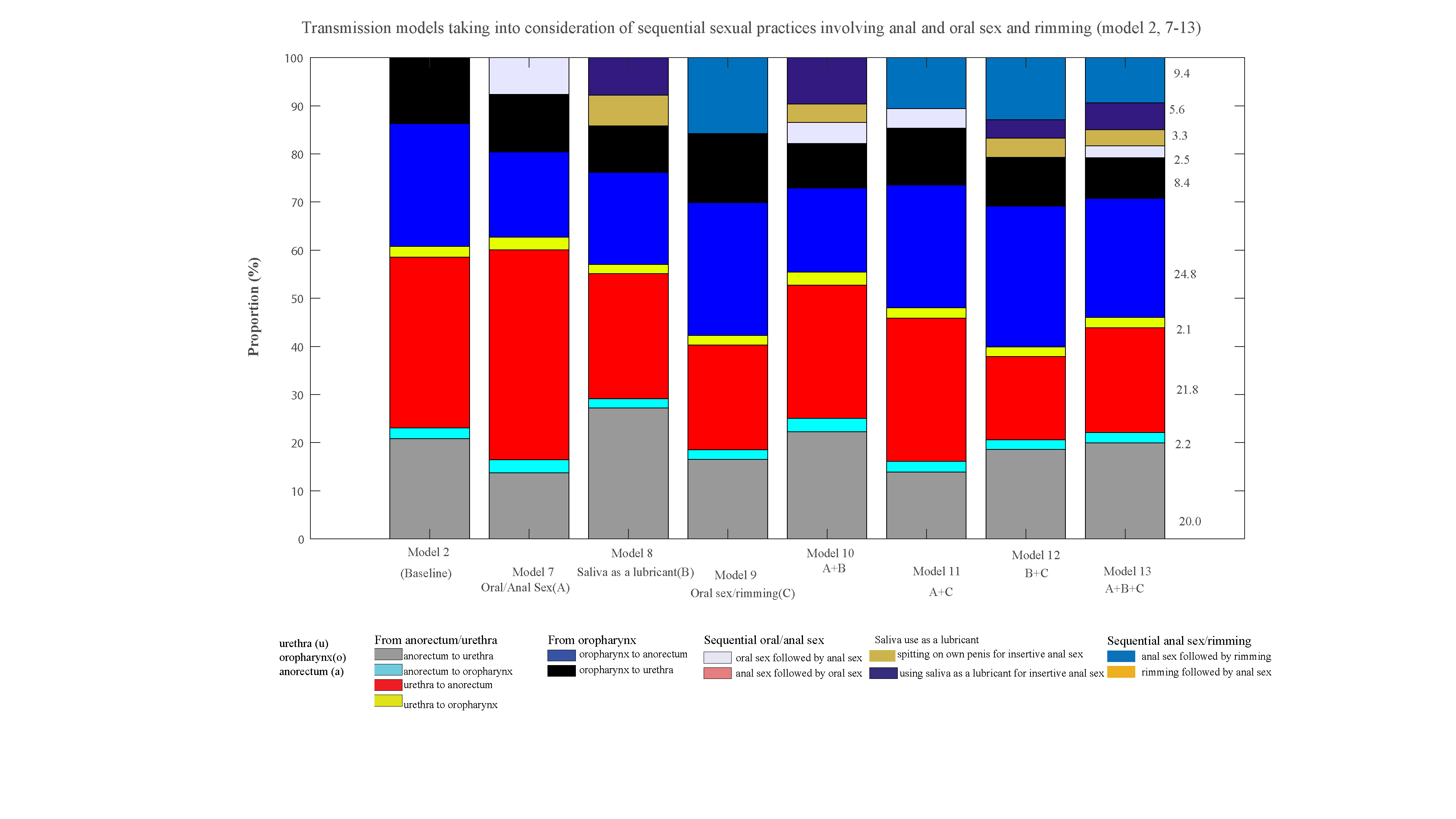


**Figure S32c.** Estimated proportion of incident *Chlamydia trachomatis* cases caused by sexual practices in MSM from the eight models (model 2, 7-13) among 4888 MSM attending Melbourne Sexual Health Centre in 2018 and 2019：the proportion of sequential sexual practices = 100%
